# Cross-species comparison significantly improves genome-wide prediction of cis-regulatory modules in Drosophila

**DOI:** 10.1186/1471-2105-5-129

**Published:** 2004-09-09

**Authors:** Saurabh Sinha, Mark D Schroeder, Ulrich Unnerstall, Ulrike Gaul, Eric D Siggia

**Affiliations:** 1 Center for Studies in Physics and Biology, The Rockefeller University, 1230 York Ave, New York, NY10021, USA; 2 Laboratory of Developmental Neurogenetics, The Rockefeller University, 1230 York Ave, New York, NY10021, USA

## Abstract

**Background:**

The discovery of cis-regulatory modules in metazoan genomes is crucial for understanding the connection between genes and organism diversity. It is important to quantify how comparative genomics can improve computational detection of such modules.

**Results:**

We run the Stubb software on the entire D. melanogaster genome, to obtain predictions of modules involved in segmentation of the embryo. Stubb uses a probabilistic model to score sequences for clustering of transcription factor binding sites, and can exploit multiple species data within the same probabilistic framework. The predictions are evaluated using publicly available gene expression data for thousands of genes, after careful manual annotation. We demonstrate that the use of a second genome (D. pseudoobscura) for cross-species comparison significantly improves the prediction accuracy of Stubb, and is a more sensitive approach than intersecting the results of separate runs over the two genomes. The entire list of predictions is made available online.

**Conclusion:**

Evolutionary conservation of modules serves as a filter to improve their detection *in silico*. The future availability of additional fruitfly genomes therefore carries the prospect of highly specific genome-wide predictions using Stubb.

## Background

Several computational approaches to the problem of predicting cis-regulatory modules ('CRM's) have been reported recently. Berman *et al*. [1], Markstein *et al*. [2] and Halfon *et al*. [3] predicted CRM's involved in body patterning in the fly, and experimentally verified their predictions. The underlying principle in these algorithms was to detect dense clusters of binding sites, as determined by matches (above some threshold) to catalogued transcription factor weight matrices. The algorithm of Rajewsky *et al*. [4], called Ahab, avoided the use of thresholds on weight matrix matches by a probabilistic modeling of CRM's. Ahab predictions within the segmentation gene network were subjected to extensive experimental validation, with excellent overall success (Schroeder *et al*. [5]). Most predicted CRM's, when placed upstream of a reporter gene, faithfully reproduce one or more aspects of the endogenous gene expression pattern. Moreover, an analysis of binding site composition over the entire set of validated modules reveals that Ahab's prediction of binding sites correlates well with expression patterns produced by the modules and suggests basic rules governing module composition.

The Stubb algorithm (Sinha *et al*. [6]) extended Ahab's approach by incorporating the use of two-species sequence information. Stubb also allows the option of scoring positional correlations between binding sites, but this option was not exercised in this study. For each sequence window analyzed, Stubb first computes the homologous sequence in the second species and aligns them using LAGAN (Brudno *et al*. [7]). The sequence is then partitioned into "blocks" (contiguous ungapped aligned regions of high percent identity) and non-blocks (sequence fragments between consecutive blocks, in either species). Putative binding sites in blocks are scored under an assumption of common evolutionary descent, using a probabilistic model of binding site evolution. Thus a "weak" site that is well conserved will score higher, while a "strong" site that is poorly conserved will have its score down-weighted. The score of the sequence window includes contributions from binding sites in blocks as well as in non-blocks. Stubb is implemented so that it can be run either on single species or two species data. In the single species mode, it is practically identical to the Ahab program. The Stubb software is available for download from 

In this paper, we present evidence that the exploitation of cross-species comparison (between *D. melanogaster *and *D. pseudoobscura*) using Stubb can lead to a significant improvement in the accuracy of genome-wide CRM prediction. To our knowledge, this is the first direct evaluation of the effect of cross-species comparison on CRM prediction on a genome-wide scale. Another important contribution of this paper is to present a benchmark for evaluating genome-wide CRM prediction tools, collected from the BDGP database and the literature, and curated by manual inspection of several hundred expression patterns. Using the same benchmark, we evaluate the effect of varying how background sequence information is incorporated in the algorithm, since this is the only tunable parameter in the Stubb program, other than the module length. We are thus able to suggest the optimal parameter settings for genome-wide CRM prediction using Stubb. Finally, we report all genome-wide predictions for cis-regulatory modules involved in anterior-posterior patterning in the early fly embryo, using both single-species and two-species Stubb, many of which make a strong case for experimental validation.

### Segmentation gene network

The transcription control paradigm we use as our test system is the segmentation of the anterior-posterior (ap) axis during early *Drosophila *embryogenesis, which has long been one of the preferred arenas for studying transcription control *in vivo*. The segmentation genes form a hierarchical network that, in a process of stepwise refinement, translates broad, overlapping expression gradients into periodic patterns of 14 discrete stripes, which prefigure the 14 segments of the larva (for reviews see St Johnston & Nusslein-Volhard [8]; Rivera-Pomar & Jackle [9]; Furriols & Casanova [10]). The maternal factors form gradients stretching along the entire ap axis of the embryo, the zygotic "gap" factors are expressed in one or more broad slightly overlapping domains; together they generate the 7-stripe patterns of the pair-rule genes; finally, the segment-polarity genes are expressed in 14 stripes. The regulation within the segmentation gene hierarchy is almost entirely transcriptional, and most of the participating genes are transcription factors themselves, activating (in the case of the maternal factors) or repressing (most gap factors) the transcription of genes at the same level or below. In most cases, the relevant binding sites are clustered within a small interval of 0.5–1 kb; these CRM's typically contain binding sites for multiple transcription factors and multiple binding sites for each factor. The clustering and the combinatorial and redundant nature of the input facilitate the computational search for segmentation control elements. Since the expression patterns of the segmentation genes are typically complex, their control regions often contain multiple separate CRM's controlling different aspects of the pattern.

The segmentation paradigm has been used as a test system for the computational detection of CRMs by us and others (Rajewsky *et al*. [4], Schroeder *et al*. [5], Berman *et al*. [1], Grad *et al*. [11]). Here, as before (Schroeder *et al*. [5]), we use the maternal and zygotic gap factors Bicoid, Hunchback, Caudal, Knirps, Krüppel, Giant, Tailless, Dstat, and the TorRE binding factor as input to Stubb. The binding site specificity of each factor is characterized by a position weight matrix that is based on a collection of experimentally verified binding sites.

### Evaluation methodology

The complete genomes of two fruitflies, *D. melanogaster *and *D. pseudobscura *have been sequenced, and Stubb was used to predict CRM's in the *D. melanogaster *genome. This was done in two modes – (i) STUBBSS, where Stubb is run on *D. melanogaster *genomic sequence alone, and (ii) STUBBMS, where Stubb uses orthologous sequence data from *D. pseudobscura *to help predict CRM's in *D. melanogaster*. For each mode of execution, we obtain a separate list of predicted CRM's, sorted in order of confidence in the prediction. The ideal test for our purpose would be to compare the accuracy of these two sorted lists. However, the set of experimentally verified CRM's involved in this system is sparse compared to the size of the system – roughly 50 CRM's are known (including the 15 new modules from Schroeder *et al*. [5]), while the number of target genes is several hundreds, by our estimate. Hence, direct evaluation of the success-rate of predictions is not feasible, and we use an alternative source of information to evaluate predictions, as described next.

A functional CRM directs the expression of a gene, by definition, and typically this gene is located in close proximity to the CRM. Hence, we may *map *the list of predicted CRM's to a list of predicted blastoderm-patterned genes – for each CRM predicted by Stubb, the *nearest *gene is identified, and if this gene is less than a threshold distance of 20 Kbp away, it is predicted to be a blastoderm-patterned gene. The resulting list of predicted "patterned genes" may now be evaluated for accuracy. (Any duplicates in the list are removed before evaluation.) The Berkeley Drosophila Genome Project (BDGP) has catalogued the expression patterns of a large number of genes in *D. melanogaster*, at various stages of development. We considered such a catalogue of 2167 genes, obtained from BDGP and from the literature. (See Test Genes [Additional File 1].) Visual inspection of the expression patterns of these genes revealed that 286 of them can be classified as having patterned expression along the anterior-posterior axis. (See Materials and Methods; also Patterned Genes [Additional File 2].) Hence, our benchmark is the entire set of 2167 genes, the "positive" set is the 286 ap-patterned genes, the remaining 1881 forming the "negative" set. This enables us to evaluate the accuracy of lists of patterned genes predicted by STUBBSS and STUBBMS, and compare their performance.

We note that some accuracy is lost in the translation of a list of predicted CRM's to the predicted genes it is mapped to, as per the mapping defined above. For instance, it is known that CRM's may control a gene located at large distances, i.e., further than the distance threshold of 20 Kb used in the mapping procedure. Also, it is possible that a CRM is located close to two genes, and directs the expression of both genes, or only of the farther gene, being somehow insulated from the nearer one. To address these concerns, we repeat our evaluation with a slightly different mapping from the one described above. A caveat that remains is that there may be genomic sequences that are functional, in the sense that they are capable of directing a specific blastoderm pattern in reporter gene constructs, but whose activity is 'silenced' in native genomic context and does not translate to patterning of any gene. Also, the CRM may direct expression of the gene only at post-blastodermal stages, so that the gene is not included in the "positive" test set of blastoderm patterned genes. Conversely, it may also happen that a predicted CRM lies close to a patterned gene, thereby being counted as a true positive, but the predicted CRM is not the sequence responsible for the gene's regulation. We assume that such effects are not biased against either algorithm.

## Results

### *STUBBMS *performs significantly better than *STUBBSS*

**Figure 1:**
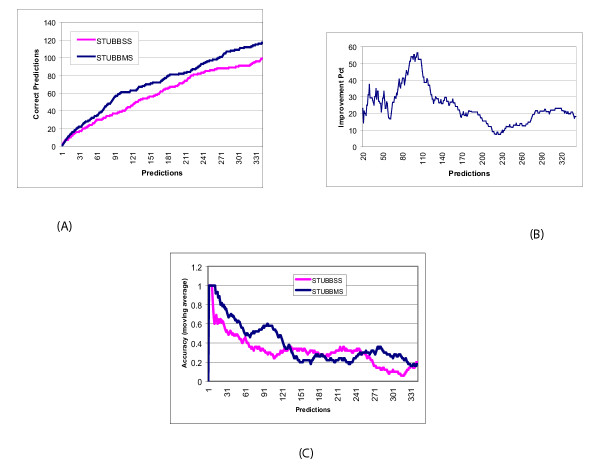
(a) Effect of two-species data on performance. The x-axis is the number of unique genes predicted to be ap patterned in the blastoderm, restricted to those for which we have expression information. The y-axis is the number of predictions that are actually blastoderm patterned. STUBBSS and STUBBMS represent the single-species and two-species Stubb runs respectively. (b) The increase in number of correct predictions from STUBBSS to STUBBMS, as a percentage of the correct predictions made by STUBBSS. (c) The specificity values for a moving window of 50 predictions. The y-axis is the number of correct predictions as a fraction of the number of predictions, which is 50 for each window.

Figure 1a shows the results of our evaluation procedure on STUBBSS and STUBBMS. These results are for the best choice of parameters for each algorithm – local, 1^st ^order background for STUBBSS and global, 2^nd ^order background for STUBBMS. (The meanings of these parameter values are explained later in this section.) The x-axis is the number of unique genes that are predicted by the algorithm (by progressively decreasing its score threshold) *and *are in the set of 2167 genes with expression information. On the y-axis we plot how many of those predicted genes are in the "positive" set (i.e., have an ap blastoderm pattern.) Thus, the y-axis is the specificity of the algorithm. We observe that STUBBMS performs significantly better than STUBBSS. For instance, to predict 100 genes correctly, STUBBSS has to make 343 predictions, while STUBBMS only has to make 267 predictions. Figure 1b plots the difference in the number of correct predictions as a fraction of the number of correct STUBBSS predictions, i.e., the percentage change in specificity for the same number of predictions made by either algorithm. We find a typical improvement of over 20%, even when over 300 overall predictions are made by each algorithm. Figure 1c shows the progression of each algorithm's prediction specificity in a moving window of 50 predictions. We find that STUBBMS has a significantly higher hit rate for the first ~120 predictions, after which both algorithms perform comparably. Even for the lower ranked predictions (i.e., those below rank 120), we find a specificity of 20 – 35% with STUBBMS, which is roughly twice the random expectation of 13% based on 286 positives in 2167 genes.

In order to further scrutinize the difference in predictions made by the two modes of Stubb, we focused on the points where their difference is most pronounced. Thus, in the top 102 unique gene predictions (for which we have information), STUBBSS reports 39 positives, while STUBBMS scores 61 hits, an improvement of over 56%. In comparison, the random expectation is ~13.5 hits. Thus the predictions of both STUBBSS and STUBBMS are significantly enriched in patterned genes (P < 10^-12 ^and <10^-37 ^respectively, Binomial Proportions test). Further examination of the top 102 gene predictions made by each algorithm revealed that 24 true positives are common to both lists. STUBBMS reports 37 true positives not discovered by STUBBSS, while the latter reports 15 true positives not found by the former. Similar results are seen for the top 311 predictions (another peak in Figure 1b): 70 correct predictions were common to both algorithms, 42 were predicted by STUBBMS only, and 21 by STUBBSS only. Thus there is substantial exclusivity in the sets of true positives of each algorithm.

We next examined separately the following three sets of genes: (i) INTERSECTION (predicted by both algorithms in the top 311) (ii) MS-ONLY (predicted only by STUBBMS) and (iii) SS-ONLY (predicted only by STUBBSS). Table 1 shows the break-down of these sets in terms of the strength of expression of their member genes. Overall, 124 of the 286 patterned genes, i.e., about 43%, are strongly expressed. We find in Table 1 that the sets INTERSECTION and MS-ONLY have more strongly expressed genes than weak and intermediate ones, and the opposite trend is seen in the set SS-ONLY.

One possible strategy that uses two-species sequence is to make predictions using STUBBSS on each of the two genomes separately and then intersect the respective lists. We found this strategy to be very restrictive – for instance, with a particular score threshold, STUBBSS predicts 205 unique genes in *D. melanogaster*, but intersecting these predictions with a similar number of top predictions in *D. pseudoobscura *gives only 68 unique genes, 33 of which are patterned. Of the top 68 predictions made in *D. melanogaster *alone, 29 are patterned. Thus the "intersection" strategy yields only a modest improvement over the single-species search, and does so at the price of significantly reducing the total number of predictions. Similar results were obtained when intersecting modules instead of gene predictions.

**Figure 2:**
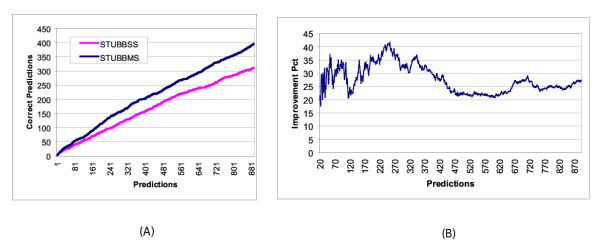
(a) Effect of two-species data on performance, using a less stringent evaluation method than in Figure 1. The x-axis is the number of predicted CRM's, restricted to those for which at least one of the two nearest genes has expression information. A prediction is deemed "correct" if either of its two nearest genes has a blastoderm-specific pattern. (b) The increase in number of correct predictions from STUBBSS to STUBBMS, as a percentage of the correct predictions made by STUBBSS.

We have noted above that the evaluation method is influenced by the way we map the predicted CRM's to predicted genes. To offset potential biases induced by this mapping, we repeated our analysis with a slightly different evaluation procedure, borrowing from the approach of Grad *et al*. [11]. We now traverse the sorted list of CRM's and count a CRM as a prediction if either of its two flanking genes has expression information. Furthermore, we designate a prediction to be "correct" if either of the two flanking genes has a blastoderm pattern. The assumption, as in Grad *et al*. [11], is that any predicted CRM near a blastoderm-patterned gene is a functional CRM responsible for some aspect of the pattern. Also, we are now counting modules rather than genes, i.e. we are allowing for multiple hits to the same gene. Figure 2a plots the results of STUBBSS and STUBBMS as per this new method of counting predictions and hits. We again notice a significant improvement in STUBBMS. For instance, in the top 300 CRM predictions for which a neighboring gene has expression information, STUBBMS makes 160 correct predictions while STUBBSS scores 121 hits. The gap between STUBBSS and STUBBMS increases as more predictions are considered, so that the improvement consistently stays above 20%, as seen in Figure 2b. For the remainder of this section, our evaluation method will use the more stringent mapping described earlier, wherein the nearest gene is predicted. Since our test data is in the form of lists of genes, we adhere to the evaluation strategy that counts genes. It is clear from Figures 1 and 2 that counting modules rather than genes improves the prediction accuracy, due to multiple CRM predictions for some blastoderm-patterned genes.

The default mapping from CRM's to genes used in our evaluations predicts a gene to be patterned only if its proximal end is less than 20 Kb from the CRM. Schroeder *et al*. [5] studied the range of locations of experimentally verified CRM's relative to the gene. They found that while there is a clustering of CRM's within the proximal 5 Kb region upstream, downstream or intronic of a gene, it is not unusual to have CRM's more than 10 Kb away from the regulated gene. Nelson *et al*. [12] observe that for D. melanogaster, the intergenic space on either side of a gene has a mean of 2 Kb – 10 Kb, depending on the complexity of the gene's function. We repeated our evaluation with different values of the distance threshold, and found that lower thresholds (5 Kb, 10 Kb) decrease the recovery rate, while higher thresholds (50 Kb) do not affect performance. (Data not shown.)

**Figure 3:**
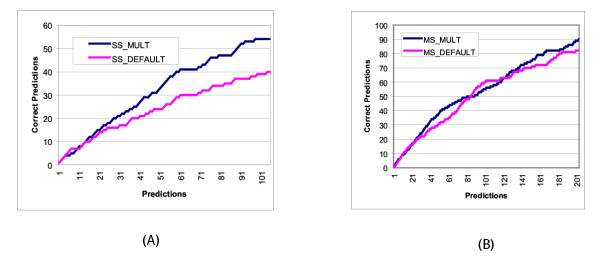
Prediction accuracy under the requirement that each predicted gene be evidenced by at least two predicted CRM's. "MULT" refers to this strategy, while "DEFAULT" refers to the default mapping of CRM's, without this restriction. (a) STUBBSS (b) STUBBMS.

Genes in the segmentation hierarchy often have multiple aspects to their expression pattern, with more than one CRM regulating them. We therefore measured how the Stubb predictions fare if we required that each predicted gene be evidenced by at least two predicted CRM's. This heuristic improves the performance of STUBBSS more prominently than that of STUBBMS, though much fewer predictions are made by either algorithm. (See Figure 3.) While 342 unique gene predictions were made by STUBBSS (Figure 1), we now observe that only 105 predictions are made using the same score threshold and the new way of counting predictions. Thus, it appears that STUBBSS performance is open to considerable improvement in the top ~100 predictions, by using either the multiple CRM restriction or the second species' sequence data. The two-species strategy however is able to increase specificity without loss of sensitivity.

**Figure 4:**
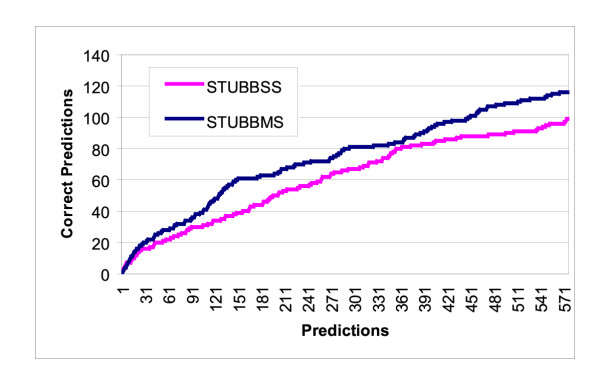
Effect of two-species data on performance, using a more complete data set that includes genes with ubiquitous, maternal-only, or no expression. The axes are as in Figure 1a. (The x-axis has a greater range than in Figure 1a.)

We have, in all tests reported in this paper, used as input a set of 2167 genes whose expression patterns are available either from BDGP or from the literature. BDGP has a supplementary list of 2065 genes for which only textual annotation has been made public, since these genes have been found to be either (i) ubiquitously expressed at all developmental stages, (ii) not expressed at any stage, or (iii) only maternally expressed. (See Additional Genes [Additional File 3].) Inclusion of these supplementary genes in our data set would approximately halve the overall fraction of patterned genes. When we examine the performance curves of STUBBSS and STUBBMS for this pattern-diluted data set (Figure 4), we find that STUBBMS shows an improvement over STUBBSS similar in proportion to that in the default data set, even though the prediction specificity of both programs suffers a drop (as compared to that in Figure 1a), typically in the range of 10–30%. Note, however, that this is substantially lower than the 50% drop one would expect by chance, given that the total number of genes has almost doubled, while the number of patterned genes remains constant.

### Characteristics of genes predicted by *STUBBMS*

**Figure 5:**
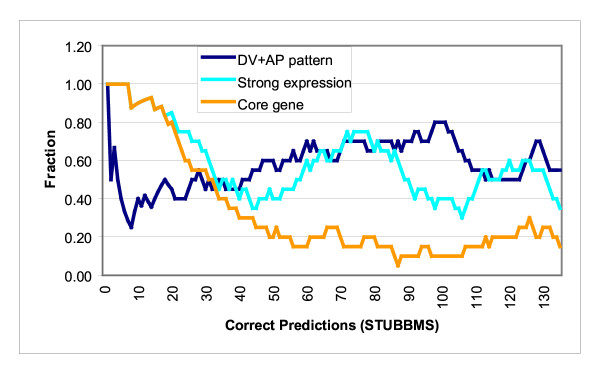
Annotation of blastoderm patterned genes predicted by STUBBMS. "DV+AP pattern": Genes with dorso-ventral aspect of pattern, in addition to anterior-posterior pattern. "Strong expression": Genes with strong expression. "Core gene": A set of 48 genes experimentally known to be required for segmentation. The x-axis represents correctly predicted genes. Each point represents, for a moving window of 20 correctly predicted genes, the fraction of genes in the appropriate category.

Our annotations of the blastoderm patterned genes also include whether the gene expression is strong, weak or of intermediate strength; if it has a dorsal-ventral (dv) modulation in addition to the primary anterior-posterior pattern; and if the gene belongs to a "core" set of 48 genes that have been shown experimentally to be required for the segmentation of the embryo (Schroeder *et al*. [5]). We were therefore able to examine the characteristics of the genes correctly predicted by Stubb, along these axes of information. The top 135 correct (gene) predictions made by STUBBMS were examined progressively, 20 predictions at a time. (That is, the correct predictions ranked *I *to *I+19 *were examined, with *I *being incremented in steps.) In each step, we computed the fraction of the 20 genes that belonged to the following three non-exclusive categories: (i) genes with dv (in addition to ap) modulation, (ii) genes with strong expression pattern, and (iii) genes in the "core" set of 48 genes. These values are reported in Figure 5. We find that

1. Genes with dorsal-ventral aspects to their blastoderm pattern are more frequent at lower ranks of prediction; i.e., the top predictions are enriched in genes with anterior-posterior patterns only.

2. Core genes are predominantly found in the top predictions.

3. Genes found at higher ranks are somewhat more likely to be strongly expressed.

The first two observations imply that the genes more directly involved in the ap axis formation are recovered at better ranks, and that the lower rank genome-wide predictions are richer in derivative patterns characteristic of genes with more complex regulatory inputs (pair-rule factors, dv factors etc.). The same trends were found for the correct predictions made by STUBBSS. (Data not shown.)

### Optimal parameter settings for Stubb

We next evaluate the effect of varying how background sequence information is incorporated in the Stubb algorithm. This is the only configurable aspect of the program, other than the module length. (In a separate test, we ran Stubb with a module length of 700 instead of the default value of 500, and found no significant difference in the prediction specificity curve.) One important parameter is the "Markov order" of background. A value of *k *for this parameter means that local correlations are assumed to be present at the level of (*k+1*)-mers, i.e., the random probability of seeing a particular base at a position depends on the bases seen at the previous *k *positions. (For readers familiar with the studies of Rajewsky *et al*. [4] and Schroeder *et al*. [5], "background *k*" in those studies is the same as a *(k-1)*^th ^order background in the terminology of this paper.) We vary this parameter to take the values *k = 1 *and *k = 2*, in different runs. The other parameter is the actual sequence used by Stubb to measure background nucleotide frequencies. Here the two options are (i) to use the current sequence window as background, or (ii) to use a pre-specified sequence (or collection of sequences) as background. We call these two the "local" and "global" background models respectively. For the "global" model, we input into Stubb 150 Kb of sequence from non-coding regions of the D. melanogaster genome, collected from the five chromosome arms 2L, 2R, 3L, 3R, and X.

**Figure 6:**
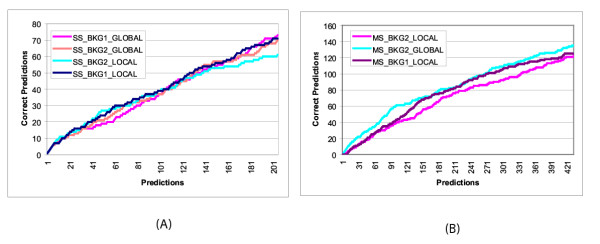
Effect of different parameter settings in Stubb, on prediction accuracy. (a: Single species Stubb, b: two-species Stubb.) The axes have the same interpretation as Figure 1a. Terminology in legend: "SS" – STUBBSS; "MS" – STUBBMS; "BKG1" and "BKG2": 1^st ^and 2^nd ^order background respectively; "LOCAL" and "GLOBAL": local and global background models respectively. (The axes are not to the same scale in (a) and (b).)

Figure 6 plots the specificity curves (as in Figure 1a) for each combination of parameter values tested. Figure 6a reports on different variants of STUBBSS, while Figure 6b plots the performance of STUBBMS. We find that STUBBSS performs best with a local, 1^st ^order background, though the other parameter values produce only slightly different results. On the other hand, the effect of background parameters on two-species Stubb is more pronounced, with the best choice being a global, 2^nd ^order background. Using a global 1^st ^background order gives almost identical results (data not shown), hence we infer that a global background is the optimal choice for STUBBMS.

**Figure 7:**
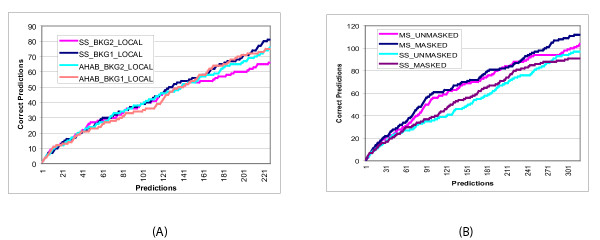
(a) Comparison between Ahab and Stubb, local background of orders 1 and 2 in different runs. All runs are on D. melanogaster genome only. (b) Effect of tandem repeat masking of input sequences. "MS_MASKED" and "MS_UNMASKED": STUBBMS, with global background model of order 2, on masked and unmasked sequence respectively. "SS_MASKED" and "SS_UNMASKED": STUBBSS, with local background model of order 1, on masked and unmasked sequence respectively.

As mentioned earlier, the STUBBSS program implements the same class of algorithm as the Ahab algorithm of Rajewsky *et al*. [4], with some technical differences, and therefore the two programs should produce similar results. We sought to verify this claim by running the Ahab program (with 1^st ^and 2^nd ^order Markov backgrounds), and comparing its performance to that of Stubb. (Ahab can only be run in the local background mode.) Figure 7a shows that there is not a significant difference between Stubb and Ahab CRM predictions.

All the above runs were on genomic sequence with tandem repeats masked by the Tandem Repeats Finder program of Benson [13]. We have found that this heuristic improves genome-wide CRM prediction by Stubb. To substantiate this claim, we ran STUBBSS and STUBBMS on raw (unmasked) genomic sequence. Figure 7b plots the results. We find that both STUBBSS and STUBBMS perform better on masked data than on unmasked data. However, when Stubb is used to analyze shorter sequences (such as the upstream and downstream regions of a gene of interest), we have found unmasked sequence to be more useful, since false positives are less of a concern.

## Discussion

The Stubb program is an extension of Ahab, with the important feature that it can handle two-species data within its probabilistic framework. The two programs differ in their underlying optimization method, with Stubb using an Expectation-Maximization approach in contrast to Ahab's conjugate gradient method. Performance evaluation of the two programs shows little difference between them, implying that the algorithm is robust to the actual optimization method used. Another technical difference between Ahab and Stubb is in the manner that orientation of binding sites is treated. While Stubb assumes a uniform prior on the orientation of a binding site, Ahab picks the best orientation for each site, with the caveat that probabilities are not strictly normalized.

An important component of Stubb is the alignment step where the two species are aligned (using LAGAN) and blocks of high sequence similarity are extracted. (See Methods.) The parameters used in LAGAN runs were obtained from Emberly *et al*. [14], who derived the alignment parameters that maximize the overlap between experimentally verified binding sites and blocks of sequence conservation. They also studied the effect of changing the alignment algorithm (LAGAN from Brudno *et al*. [7]versus SMASH from Zavolan *et al*. [15]) for CRM's in the two fly species, and found no significant difference. Finally, the similarity thresholds we use for defining conserved blocks (10 bp or longer, with >70% identity) were obtained by trying a broad range of values, and choosing those that produced the best results, as per our genome-wide evaluation.

Tandem repeat masking is a common pre-processing step for many sequence analysis applications involving binding sites. These repeats are short locally duplicated sequences, that may or may not be related to binding sites. It is not clear *a priori *how tandem repeats should affect module detection – repeats similar to binding sites of the system may improve sensitivity when they occur in CRM's; but if repeats resembling binding sites occur by chance in non-functional regions, prediction specificity may suffer. The occurrence of tandem repeats marks statistical deviation from Stubb's probabilistic model of sequence generation. In our tests, we found that repeats distract the algorithm more than they help, as manifested in better performance on repeat-masked sequence. (See Figure 7b.) This may be because two of the weight matrices in our collection (Hunchback and Caudal) resemble a poly-T stretch. Therefore, the poly-A or poly-T tandem repeats that occur promiscuously in the genome may be confused with sites of these two weight matrices.

A recently published tool for genome-wide CRM prediction, called PFR-Searcher (Grad *et al*. [11]), first identifies "phylogenetically footprinted regions" or "PFR"s, that are sequences conserved between the two fly species, and then searches for a subset of these that are most similar in content to an input set of promoters. Their approach differs from Stubb in the nature of prior information input to the algorithm. While Stubb uses an input set of weight matrices, the training data for PFR-Searcher is a set of CRM's which, in their approach, is itself provided by a similarity search among PFR's of co-regulated genes. PFR-Searcher therefore has the advantage of not requiring knowledge of the transcription factor weight matrices relevant to the system. However, its ability to predict the binding site composition of potential CRM's is therefore more limited as compared to Stubb. (The Stubb program computes an average "parse" of the predicted module into its constituent binding sites for various transcription factors.) Grad *et al*. [11]report an evaluation of their algorithm on a test system very similar to ours, but with enough minor differences to make a direct comparison of performance impossible. For instance, the entire list of CRM's predicted in their evaluation corresponds, as per our CRM → gene mapping, to a set of only 46 unique genes, of which 31 are patterned. Twenty of these 31 correct predictions are also found in the top 46 gene predictions of STUBBMS, indicating a good degree of overlap between the two methods, at least in their highest ranked predictions. A fair and comprehensive comparison of the predictive power of these two algorithms is an interesting topic for future work, and it will be even more interesting to run STUBBMS only on PFR's detected by their criteria.

Regarding the recovery of patterned genes by Stubb, several observations can be made. Of the 286 genes with ap patterns, we recover roughly half at a score cut-off of 10, using STUBBMS. Why is the other half not found? While it is obvious that lowering the cut-off will detect more patterned genes, there are other reasons why a patterned gene may be missed by Stubb. Some genes are likely to be lost due to the distance filter we have imposed (CRM to nearest gene <20 kb), since the regulatory regions of some genes (e.g., homeotic genes) are likely to be larger than that. More importantly, most of the patterned genes that are not part of the core transcriptional machinery have derivative patterns that reflect a more complex input (binding sites for pair rule factors, d-v factors etc.) and thus will only be recovered to the extent their input has a solid maternal/gap component. Conversely, there are at least two reasons for reporting false positives (roughly two thirds at a score cutoff of 10). The presence of an insulator could prevent the interaction between a CRM and its nearest basal promoter. More likely is a scenario where the predicted CRM's do drive expression but at post-blastoderm stages. All gap factors are active in multiple tissues in later development and therefore CRM's with dominant or exclusive gap input may well be active in these later contexts. These caveats affect all current CRM detection algorithms, and accounting for such additional axes of information as genomic context and module composition rules will be a difficult but important challenge for the future.

A very interesting observation comes from the analysis in Table 1: Genes predicted by STUBBSS only, and not by STUBBMS, have weak or intermediate expression pattern more often than strong expression. This means that the CRM's that are not well-conserved between the two species (and hence not picked up by STUBBMS) typically correspond to weakly expressed genes. This ties in with previous studies (e.g., Domazet-Loso & Tautz [16]) that found fast evolving genes in Drosophila to be expressed relatively weakly.

The Stubb program not only predicts cis-regulatory modules genome-wide, it additionally outputs the binding site profile of each predicted CRM, i.e., the locations and probabilities of binding sites in the CRM. Schroeder et al [5] use the corresponding feature in Ahab for a systematic analysis of the composition of all known or validated segmentation CRMs. The use of STUBBMS improves such binding site predictions. It is easy to adapt the program to take as input orthologous CRM's from the two species, and highlight the *changes *in terms of their binding site compositions. This leads to a powerful bioinformatic tool to predict regulatory changes between the two fly species. We can thus obtain hypotheses about changes in expression patterns, which can be verified experimentally. We have examined a representative collection of CRM's, and experimentally verified several of the changes predicted by Stubb, thereby building a catalogue of the different modes of cis-regulatory evolution. The results of this study will be reported in the near future.

## Conclusions

We have seen that the use of a second fly genome significantly improves genome-wide module prediction. Since STUBBMS uses a natural "two-species" extension of the algorithm of STUBBSS, this finding is largely a statement about the inherent potential of cross-species comparison as a paradigm for improving functional genomics. The STUBBMS program also has a natural extension to incorporate more than two genomes, and it will be very interesting to see how much of a difference a third genome makes. The genome of D. yakuba is expected to be sequenced soon, and since this species is closer to D. melanogaster, it may help better discriminate conserved regulatory modules.

## Methods

### Alignment of D. melanogaster and D. pseudobscura

*D. melanogaster *sequences were obtained from Flybase Release 3. The analysis was limited to the five chromosome arms 2L, 2R, 3L, 3R, and X. *D. pseudobscura *contigs were obtained from  (February 2003 Release). Based on Blast results, we created a mapping, called "CONTIGMAP", between regions of the *D. melanogaster *genome and *D. pseudobscura *contigs, each region typically being tens of Kb long. This mapping is many to many, i.e., different regions of *D. melanogaster *may map to the same contig, and the same (or overlapping) region in *D. melanogaster *may map to two or more *D. pseudobscura *contigs. For each entry (M, P) in CONTIGMAP, where M is the *D. melanogaster *region and P is the *D. pseudobscura *contig, the LAGAN alignment program (Brudno *et al*. [7]) was run, with parameters gap start = -6, gap extension = 0, match = 1, and mismatch = -2, and all contiguous ungapped blocks of alignment, with length 10 bp or more and 70% identity or more, were extracted. In cases where the same region in *D. melanogaster *was mapped to multiple contigs, the density of LAGAN blocks was then used to choose exactly one mapping contig.

### Stubb runs

Tandem repeats in the input sequences were masked with the Tandem Repeat Finder program of Benson [13], with parameter settings: (match = 2, mismatch = 5, indel = 5, match probability = 0.75, indel probability = 0.2, minimum score = 20, maximum period = 500). STUBBSS was run on the *D. melanogaster *genome with a sliding window of length 500 bp, in shifts of 50 bp. The input weight matrices for the maternal and gap transcription factors Bcd, Hb, Cad, Kni, Kr, Tll, Dstat and the torRE binding factor were obtained from Rajewsky *et al*. [4] and Schroeder *et al*. [5]. A weight matrix for the transcription factor Gt was constructed from known functional sites collected from the literature. STUBBMS was run on each entry (M, P) in CONTIGMAP, using a sliding window of length 500 bp on the *D. melanogaster *sequence M, in shifts of 50 bp. Thus, STUBBMS was not run on regions of *D. melanogaster *that are not aligned with some *D. pseudoobscura *contig. The weight matrices used were the same as in STUBBSS runs. The locations of the blocks computed in the alignment step (above) were input to STUBBMS, and the input value of the neutral mutation rate was 0.5, the value being chosen due to its better performance over alternatives tested.

Each genome-wide run of Stubb produces, for each starting position of the sliding window, a score that measures the likelihood of the sequence having a cluster of binding sites. The next step is to extract the coordinates of each window that scores better than all other windows overlapping it. Such windows correspond to local "peaks" in the score profile along the genome. All such "peak" windows with scores above a certain threshold are sorted in decreasing order of their score, to produce a sorted list of predicted CRM's. Each window in this list is annotated with useful information including the identity and relative location of its neighboring genes. The list is then filtered to retain only those predicted CRM's where Stubb predicts occurrences of at least two weight matrices. This is a heuristic that incorporates the combinatorial nature of CRM's, i.e., their tendency to have sites for multiple transcription factors (activators as well as repressors.) Finally, any predicted CRM that overlaps with an exon is removed from the list before evaluation. The predictions made by STUBBMS and STUBBSS are listed in the files "Predicted CRM's – two species" (Additional File 4) and "Predicted CRM's – single species" (Additional File 5), respectively.

### Annotation of gene expression database

The 792 genes which the BDGP expression database  lists as showing expression during blastoderm (embryonic stages 4–6) were visually inspected. From this list, we removed genes with ubiquitous expression (426; this also removes the presumably very small number of genes whose ubiquitous expression is controlled by separate "regional" modules), extremely faint or irreproducible expression (31), or expression in pole cells or yolk nuclei only (64), as well as genes whose expression is modulated along the dv axis only (13). The remaining 258 genes show patterned expression in the somatic portion along the ap axis of the blastoderm embryo; 28 known segmentation genes not captured in the BDGP expression database were added to the list, for a total of 286 genes showing ap patterned blastoderm expression. These genes were further categorized by expression level (strong, intermediate, weak) and type of pattern (ap, ap+dv, dv+ap). ap includes gap, pair rule and segment polarity-like patterns (e.g., *Kr*, *fkh*, *eve*); ap+dv denotes ap pattern with some dv modulation (e.g., *kni*, *so*, *en*); dv+ap denotes dv pattern with some ap modulation (e.g., *neur*).

## Authors' contributions

SS and EDS worked out the details of genome-wide Stubb runs. SS performed the Stubb runs, collected all statistics from the runs, and drafted the manuscript. EDS suggested several analyses reported. MDS, UU, and UG annotated the genes for expression pattern, suggested many of the analyses in the Results and Discussion sections, and wrote parts of the manuscript. All authors read and approved the final manuscript.

## Note added in proof

A recently published paper (Berman *et al*: *Genome Biol *2004, 5:R61, published 20 August 2004.) also evaluates the effect of cross-species comparison on CRM prediction in Drosophila.

## Supplementary Material

Additional File 1 Test Genes List of 2167 genes with expression information. (Source BDGP + literature.) Click here for file

Additional File 2 Patterned Genes List of 286 genes with blastoderm pattern, as determined by manual inspection of in-situ expression pattern. (Source BDGP + literature.) Click here for file

Additional File 3 Additional Genes List of 2065 genes that are ubiquitous, maternal only, or not expressed. (Source BDGP.) Click here for file

Additional File 4 Predicted CRM's – two species List of predicted CRM's using two-species Stubb with a global, 2^nd ^order background, sorted in decreasing order of Stubb score. Click here for file

Additional File 5 Predicted CRM's – single species List of predicted CRM's using single-species Stubb with a local, 1^st ^order background, sorted in decreasing order of Stubb score. This list of predictions, as well as that in Additional File 4 (above), is meant to be as inclusive as possible; therefore, the specificity of the lowest ranked predictions may be poor. Click here for file

## Figures and Tables

**Table 1:** Expression patterns of predicted genes. Top 311 genes predicted as being patterned, by STUBBSS and STUBBMS. "INTERSECTION": Genes correctly predicted by both methods. "MS-ONLY": Genes correctly predicted by STUBBMS and not by STUBBSS. "SS-ONLY": Genes correctly predicted by STUBBSS and not by STUBBMS.

	STRONGLY EXPRESSED	WEAK + INTERMEDIATE
INTERSECTION	41	29
MS-ONLY	23	19
SS-ONLY	7	14
