# Supplementary material for: Cross-species comparison significantly improves genome-wide prediction of cis-regulatory modules in Drosophila
Source: BMC Bioinformatics. 2004 Sep 9;5:129. doi: 10.1186/1471-2105-5-129 (PMC521067; doi:10.1186/1471-2105-5-129)
Supplement: Additional File 4 — Predicted CRM's – two species List of predicted CRM's using two-species Stubb with a global, 2nd order background, sorted in decreasing order of Stubb score. [file 1471-2105-5-129-S4.html]

```

```

```

```

```

```

```

```

```
*********************** EXPLANATIONS ************************
** List of predicted CRM's using STUBBMS, global 2nd order background.
** "Score" refers to StubbMS score.
** List is sorted in decreasing order of score.
** "GBROWSE" link displays the CRM on genome browser.
** For each predicted CRM, two nearest genes are reported.
** "insitu" at the beginning of a gene line means the gene has insitu information.
** "highlight" at the beginning of a gene line means the gene is blastoderm patterned.
** Coordinates of a module are with respect to D.melanogaster Release 3.
** For each CRM, its coordinates are followed by its binding site contents.
** For example, "hunchback   0.014827    5.389006" means there are 5.389006
** predicted occurrences of hunchback. (Ignore the number in second column.)
**
** To use Gbrowse : Click on "GBROWSE" link; Under "Dump, Searches and other Operations",
** choose "Annotate Fast Function Plot" and click "Configure"; Click "on" and the tracks
** you want to see, and click "Configure"; 
*************************************************************

*********************** Rank 1 [Score 39.921265]   GBROWSE*******************

insitu CG8112 in-situ | CG8112 | + | -181 | 14105 | INTRAGENIC | intron:CG8112-RA:1 | CG8112-RA | "-" | CG8112-RB | "-"
 CG9790 in-situ | CG9790 | - | 14693 | 14037 | DOWNSTREAM | CG9790-RA | "-"


note: overlaps known module hb_central_posterior_stripes by 500 bases (module coords: 4526535-4527557)

*********************** Rank 2 [Score 35.766846]   GBROWSE*******************

insitu CG32139 in-situ | Sox21b | - | -41946 | -60887 | UPSTREAM | CG32139-RA | "-"
insitu highlight CG5893 in-situ | D | - | 5350 | 2331 | DOWNSTREAM | CG5893-RA | "-"

*********************** Rank 3 [Score 35.064392]   GBROWSE*******************

 CG12134 in-situ | CG12134 | + | -4418 | -2784 | DOWNSTREAM | CG12134-RB | "-" | CG12134-RA | "-"
insitu highlight CG2328 in-situ | eve | + | 2024 | 5212 | UPSTREAM | CG2328-RA | "-"


note: overlaps known module eve_stripe3_7 by 445 bases (module coords: 5036134-5036644)

*********************** Rank 4 [Score 34.835205]   GBROWSE*******************

insitu highlight CG4717 in-situ | kni | - | -1199 | -4232 | UPSTREAM | CG4717-RA | "-"
insitu CG13253 in-situ | CG13253 | - | 22636 | 20233 | DOWNSTREAM | CG13253-RA | "-"


note: overlaps known module kni_kd by 500 bases (module coords: 20615078-20615954)
note: overlaps known module kni_no_name by 358 bases (module coords: 20613881-20615457)

*********************** Rank 5 [Score 34.408325]   GBROWSE*******************

 CG6486 in-situ | CG6486 | + | -21666 | -20474 | DOWNSTREAM | CG6486-RA | "-"
insitu highlight CG6494 in-situ | h | + | 4769 | 8049 | UPSTREAM | CG6494-RA | "-"

note: overlaps known module h_rescue by 500 bases (module coords: 8620516-8642090)
note: overlaps known module h_stripe1_1990 by 500 bases (module coords: 8629353-8630638)
note: overlaps known module h_stripe1_1991 by 500 bases (module coords: 8629635-8630510)
note: overlaps known module h_stripe2_1991 by 500 bases (module coords: 8625021-8630510)
note: overlaps known module h_stripe5_1990a by 500 bases (module coords: 8627668-8630638)
note: overlaps known module h_stripe1_5_1991 by 500 bases (module coords: 8627680-8630510)

*********************** Rank 6 [Score 33.137817]   GBROWSE*******************

insitu highlight CG2328 in-situ | eve | + | -8526 | -5338 | DOWNSTREAM | CG2328-RA | "-"
 CG2331 in-situ | TER94 | + | 3044 | 7450 | UPSTREAM | CG2331-RA | "-" | CG2331-RB | "-"

note: overlaps known module eve_stripe1 by 500 bases (module coords: 5046568-5047368)

*********************** Rank 7 [Score 32.919922]   GBROWSE*******************

insitu highlight CG16738 in-situ | slp1 | + | -8151 | -6694 | DOWNSTREAM | CG16738-RA | "-"
insitu highlight CG2939 in-situ | slp2 | + | 3011 | 5370 | UPSTREAM | CG2939-RA | "-"

*********************** Rank 8 [Score 32.488525]   GBROWSE*******************

 CG11692 in-situ | CG11692 | - | -14122 | -14678 | UPSTREAM | CG11692-RA | "-"
 CG1829 in-situ | Cyp6v1 | + | 5034 | 8503 | UPSTREAM | CG1829-RA | "-"


*********************** Rank 9 [Score 32.429504]   GBROWSE*******************

 CG14947 in-situ | CG14947 | + | -4256 | -3444 | DOWNSTREAM | CG14947-RA | "-"
insitu highlight CG6716 in-situ | prd | - | 5377 | 1918 | DOWNSTREAM | CG6716-RB | "-" | CG6716-RA | "-"


note: overlaps known module prd_rescue by 500 bases (module coords: 12066868-12085327)

*********************** Rank 10 [Score 32.068237]   GBROWSE*******************

 CG6486 in-situ | CG6486 | + | -17166 | -15974 | DOWNSTREAM | CG6486-RA | "-"
insitu highlight CG6494 in-situ | h | + | 9269 | 12549 | UPSTREAM | CG6494-RA | "-"


note: overlaps known module h_rescue by 500 bases (module coords: 8620516-8642090)
note: overlaps known module h_stripe2_6 by 500 bases (module coords: 8625021-8627680)
note: overlaps known module h_stripe2_1990 by 500 bases (module coords: 8625021-8626101)
note: overlaps known module h_stripe2_1991 by 500 bases (module coords: 8625021-8630510)
note: overlaps known module h_stripe6_1991 by 500 bases (module coords: 8625021-8627680)
note: overlaps known module h_stripe6_1990 by 334 bases (module coords: 8625566-8626101)

*********************** Rank 11 [Score 32.025146]   GBROWSE*******************

insitu highlight CG7952 in-situ | gt | - | -9615 | -11471 | UPSTREAM | CG7952-RB | "-"
 CG7925 in-situ | tko | - | 5398 | 3729 | DOWNSTREAM | CG7925-RB | "-"

*********************** Rank 12 [Score 31.793457]   GBROWSE*******************

 CG6486 in-situ | CG6486 | + | -16116 | -14924 | DOWNSTREAM | CG6486-RA | "-"
insitu highlight CG6494 in-situ | h | + | 10319 | 13599 | UPSTREAM | CG6494-RA | "-"


note: overlaps known module h_rescue by 500 bases (module coords: 8620516-8642090)
note: overlaps known module h_stripe7_1990a by 500 bases (module coords: 8623548-8625021)
note: overlaps known module h_stripe7_1997 by 370 bases (module coords: 8623787-8624719)
note: overlaps known module h_stripe7_1990b by 500 bases (module coords: 8623984-8625021)

*********************** Rank 13 [Score 31.288330]   GBROWSE*******************

 CG9380 in-situ | CG9380 | - | -33993 | -39201 | UPSTREAM | CG9380-RA | "-" | CG9380-RB | "-"
insitu highlight CG3340 in-situ | Kr | + | 3785 | 6704 | UPSTREAM | CG3340-RA | "-"


note: overlaps known module Kr_730 by 500 bases (module coords: 20266189-20266919)
note: overlaps known module Kr_AS2_MP2 by 278 bases (module coords: 20266622-20267163)
note: overlaps known module Kr_CD1 by 500 bases (module coords: 20266189-20267347)

*********************** Rank 14 [Score 30.593750]   GBROWSE*******************

 CG15541 in-situ | CG15541 | + | -29495 | -22951 | DOWNSTREAM | CG15541-RA | "-"
 CG1342 in-situ | CG1342 | + | 11982 | 14106 | UPSTREAM | CG1342-RA | "-"

*********************** Rank 15 [Score 28.753418]   GBROWSE*******************

insitu CG12075 in-situ | CG12075 | + | -13888 | -5866 | DOWNSTREAM | CG12075-RA | "-"
insitu highlight CG10701 in-situ | Moe | - | 13800 | -4398 | INTRAGENIC | intron:CG10701-RD:2 | intron:CG10701-RB:1 | CG10701-RD | "-" | CG10701-RB | "-" | CG10701-RA | "-" | CG10701-RC | "-"

*********************** Rank 16 [Score 28.446716]   GBROWSE*******************

 CG12109 in-situ | Caf1-180 | - | -37028 | -40893 | UPSTREAM | CG12109-RB | "-" | CG12109-RA | "-"
insitu highlight CG12154 in-situ | oc | - | 7396 | -11914 | INTRAGENIC | intron:CG12154-RA:1 | CG12154-RA | "-"

*********************** Rank 17 [Score 28.425049]   GBROWSE*******************

 CG14678 in-situ | CG14678 | - | -44923 | -47724 | UPSTREAM | CG14678-RA | "-"
 CG11373 in-situ | CG11373 | - | 28604 | 27942 | DOWNSTREAM | CG11373-RA | "-"

*********************** Rank 18 [Score 28.111328]   GBROWSE*******************

insitu highlight CG7952 in-situ | gt | - | -1665 | -3521 | UPSTREAM | CG7952-RB | "-"
 CG7925 in-situ | tko | - | 13348 | 11679 | DOWNSTREAM | CG7925-RB | "-"

note: overlaps known module gt_posterior by 461 bases (module coords: 2187439-2188383)

*********************** Rank 19 [Score 28.001709]   GBROWSE*******************

insitu highlight CG2047 in-situ | ftz | + | -3453 | -1549 | DOWNSTREAM | CG2047-RA | "-"
 CG31488 in-situ | CG31488 | + | 89957 | 90586 | UPSTREAM | CG31488-RA | "-"


note: overlaps known module ftz_3prime_element by 500 bases (module coords: 2692342-2694036)
note: overlaps known module ftz_rescue_construct by 500 bases (module coords: 2683630-2694036)

*********************** Rank 20 [Score 27.463623]   GBROWSE*******************

 CG3587 in-situ | EG:39E1.2 | + | -18642 | -17337 | DOWNSTREAM | CG3587-RA | "-"
 CG32802 in-situ | CG32802 | - | 31251 | 29874 | DOWNSTREAM | CG32802-RA | "-"

*********************** Rank 21 [Score 27.240479]   GBROWSE*******************

insitu CG31216 in-situ | CG31216 | + | -13900 | 13189 | INTRAGENIC | intron:CG31216-RA:1 | CG31216-RA | "-"
 CG7535 in-situ | GluClalpha | + | 45598 | 90084 | UPSTREAM | CG7535-RA | "-" | CG7535-RB | "-"

*********************** Rank 22 [Score 27.116821]   GBROWSE*******************

 CG4021 in-situ | CG4021 | + | -6454 | -4974 | DOWNSTREAM | CG4021-RA | "-"
 CG4402 in-situ | lox2 | - | 11856 | 9817 | DOWNSTREAM | CG4402-RA | "-"

*********************** Rank 23 [Score 26.975220]   GBROWSE*******************

 CG10417 in-situ | CG10417 | - | -27427 | -30204 | UPSTREAM | CG10417-RA | "-" | CG10417-RB | "-"
 CG30437 in-situ | CG30437 | + | 23207 | 63622 | UPSTREAM | CG30437-RA | "-" | CG30437-RC | "-" | CG30437-RB | "-"

*********************** Rank 24 [Score 26.875366]   GBROWSE*******************

insitu CG15270 in-situ | BG:DS04929.1 | + | -28520 | -7250 | DOWNSTREAM | CG15270-RA | "-"
 CG15269 in-situ | BG:DS04929.3 | + | 5420 | 8749 | UPSTREAM | CG15269-RA | "-"


*********************** Rank 25 [Score 26.607666]   GBROWSE*******************

 CG1338 in-situ | CG1338 | - | -2094 | -6506 | UPSTREAM | CG1338-RA | "-" | CG1338-RB | "-"
insitu highlight CG1849 in-situ | run | + | 17106 | 19991 | UPSTREAM | CG1849-RA | "-"


*********************** Rank 26 [Score 26.600342]   GBROWSE*******************

insitu CG5594 in-situ | BEST:CK01510 | + | -16254 | 714 | INTRAGENIC | intron:CG5594-RA:17 | intron:CG5594-RD:18 | intron:CG5594-RB:17 | intron:CG5594-RC:18 | CG5594-RA | "-" | CG5594-RD | "-" | CG5594-RB | "-" | CG5594-RC | "-"
insitu CG2812 in-situ | CG2812 | + | 857 | 2528 | UPSTREAM | CG2812-RA | "-"


*********************** Rank 27 [Score 26.545532]   GBROWSE*******************

 CG1338 in-situ | CG1338 | - | -10094 | -14506 | UPSTREAM | CG1338-RA | "-" | CG1338-RB | "-"
insitu highlight CG1849 in-situ | run | + | 9106 | 11991 | UPSTREAM | CG1849-RA | "-"

note: overlaps known module run_stripe3 by 174 bases (module coords: 20355609-20357923)
note: overlaps known module run_stripe7 by 263 bases (module coords: 20352427-20358012)

*********************** Rank 28 [Score 26.528931]   GBROWSE*******************

insitu highlight CG4717 in-situ | kni | - | -1699 | -4732 | UPSTREAM | CG4717-RA | "-"
insitu CG13253 in-situ | CG13253 | - | 22136 | 19733 | DOWNSTREAM | CG13253-RA | "-"

note: overlaps known module kni_223_1995 by 147 bases (module coords: 20615953-20616109)
note: overlaps known module kni_64_1995 by 67 bases (module coords: 20615887-20615953)
note: overlaps known module kni_kd by 355 bases (module coords: 20615078-20615954)

*********************** Rank 29 [Score 26.304443]   GBROWSE*******************

insitu highlight CG3851 in-situ | odd | - | -2783 | -5309 | UPSTREAM | CG3851-RA | "-"
 CG2788 in-situ | Dot | + | 9761 | 11640 | UPSTREAM | CG2788-RA | "-"

*********************** Rank 30 [Score 26.230957]   GBROWSE*******************

 CG1338 in-situ | CG1338 | - | -15844 | -20256 | UPSTREAM | CG1338-RA | "-" | CG1338-RB | "-"
insitu highlight CG1849 in-situ | run | + | 3356 | 6241 | UPSTREAM | CG1849-RA | "-"

note: overlaps known module run_7stripes by 500 bases (module coords: 20361765-20366856)

*********************** Rank 31 [Score 26.227844]   GBROWSE*******************

 CG12623 in-situ | CG12623 | - | -4836 | -5546 | UPSTREAM | CG12623-RA | "-"
 CG1641 in-situ | sisA | - | 6335 | 5568 | DOWNSTREAM | CG1641-RA | "-"

*********************** Rank 32 [Score 26.213440]   GBROWSE*******************

 CG18363 in-situ | CG18363 | - | -16500 | -17608 | UPSTREAM | CG18363-RA | "-"
 CG12477 in-situ | CG12477 | - | 8356 | 7559 | DOWNSTREAM | CG12477-RA | "-"

*********************** Rank 33 [Score 25.851807]   GBROWSE*******************

 CG14926 in-situ | CG14926 | - | -13666 | -14616 | UPSTREAM | CG14926-RA | "-"
 CG4881 in-situ | salr | + | 8376 | 16468 | UPSTREAM | CG4881-RA | "-" | CG4881-RB | "-"

*********************** Rank 34 [Score 25.767090]   GBROWSE*******************

 CG15544 in-situ | CG15544 | + | -21862 | -7678 | DOWNSTREAM | CG15544-RA | "-"
insitu highlight CG1378 in-situ | tll | + | 2656 | 4661 | UPSTREAM | CG1378-RA | "-"

note: overlaps known module tll_K11_post by 311 bases (module coords: 26664631-26665110)
note: overlaps known module tll_rescue by 500 bases (module coords: 26661256-26671461)

*********************** Rank 35 [Score 25.692505]   GBROWSE*******************

 CG14116 in-situ | CG14116 | + | -11124 | -9074 | DOWNSTREAM | CG14116-RA | "-"
 CG17300 in-situ | CG17300 | - | 14286 | 13384 | DOWNSTREAM | CG17300-RA | "-"

*********************** Rank 36 [Score 25.377197]   GBROWSE*******************

 CG14678 in-situ | CG14678 | - | -35873 | -38674 | UPSTREAM | CG14678-RA | "-"
 CG11373 in-situ | CG11373 | - | 37654 | 36992 | DOWNSTREAM | CG11373-RA | "-"

*********************** Rank 37 [Score 25.366333]   GBROWSE*******************

insitu highlight CG6634 in-situ | CG6634 | + | -36544 | -30576 | DOWNSTREAM | CG6634-RA | "-"
 CG14020 in-situ | CG14020 | + | 19657 | 20637 | UPSTREAM | CG14020-RA | "-"

*********************** Rank 38 [Score 25.362427]   GBROWSE*******************

 CG17111 in-situ | CG17111 | - | -14469 | -17947 | UPSTREAM | CG17111-RA | "-"
insitu CG6726 in-situ | CG6726 | + | 11005 | 12742 | UPSTREAM | CG6726-RB | "-" | CG6726-RA | "-"

*********************** Rank 39 [Score 25.275085]   GBROWSE*******************

insitu highlight CG13475 in-situ | HGTX | - | -2802 | -19155 | UPSTREAM | CG13475-RA | "-"
insitu CG13478 in-situ | Cyp314a1 | + | 17460 | 19475 | UPSTREAM | CG13478-RA | "-"


*********************** Rank 40 [Score 25.256836]   GBROWSE*******************

 CG15321 in-situ | CG15321 | - | -6694 | -7401 | UPSTREAM | CG15321-RA | "-"
insitu highlight CG12653 in-situ | btd | + | 3822 | 7207 | UPSTREAM | CG12653-RA | "-"

note: overlaps known module btd_head by 500 bases (module coords: 9429057-9430856)

*********************** Rank 41 [Score 25.192139]   GBROWSE*******************

 CG17835 in-situ | inv | + | -34172 | -1454 | DOWNSTREAM | CG17835-RA | "-" | CG17835-RD | "-" | CG17835-RB | "-" | CG17835-RC | "-"
 CG30034 in-situ | CG30034 | - | 13411 | 7542 | DOWNSTREAM | CG30034-RA | "-"


*********************** Rank 42 [Score 24.831421]   GBROWSE*******************

 CG10032 in-situ | CG10032 | + | -6317 | -5400 | DOWNSTREAM | CG10032-RA | "-"
 CG2595 in-situ | RacGAP84C | + | 7024 | 9723 | UPSTREAM | CG2595-RA | "-" | CG2595-RB | "-"

*********************** Rank 43 [Score 24.762207]   GBROWSE*******************

 CG7345 in-situ | Sox21a | - | -17246 | -20066 | UPSTREAM | CG7345-RA | "-"
insitu CG32139 in-situ | Sox21b | - | 7004 | -11937 | INTRAGENIC | intron:CG32139-RA:3 | CG32139-RA | "-"

*********************** Rank 44 [Score 24.596375]   GBROWSE*******************

 CG4659 in-situ | Srp54k | + | -3622 | -1412 | DOWNSTREAM | CG4659-RA | "-"
 CG10677 in-situ | CG10677 | + | 26732 | 28660 | UPSTREAM | CG10677-RA | "-"

*********************** Rank 45 [Score 24.412231]   GBROWSE*******************

insitu highlight CG3851 in-situ | odd | - | -4283 | -6809 | UPSTREAM | CG3851-RA | "-"
 CG2788 in-situ | Dot | + | 8261 | 10140 | UPSTREAM | CG2788-RA | "-"


*********************** Rank 46 [Score 24.356018]   GBROWSE*******************

 CG5557 in-situ | sqz | + | -10240 | -3887 | DOWNSTREAM | CG5557-RA | "-"
 CG14282 in-situ | CG14282 | + | 2539 | 3472 | UPSTREAM | CG14282-RA | "-"

*********************** Rank 47 [Score 24.283020]   GBROWSE*******************

 CG4429 in-situ | Rbp2 | + | -6382 | -2905 | DOWNSTREAM | CG4429-RA | "-" | CG4429-RB | "-"
 CG9906 in-situ | CG9906 | - | 70141 | 68222 | DOWNSTREAM | CG9906-RA | "-"

*********************** Rank 48 [Score 24.257935]   GBROWSE*******************

 CG6486 in-situ | CG6486 | + | -4166 | -2974 | DOWNSTREAM | CG6486-RA | "-"
insitu highlight CG6494 in-situ | h | + | 22269 | 25549 | UPSTREAM | CG6494-RA | "-"

*********************** Rank 49 [Score 24.179199]   GBROWSE*******************

insitu highlight CG2328 in-situ | eve | + | -9176 | -5988 | DOWNSTREAM | CG2328-RA | "-"
 CG2331 in-situ | TER94 | + | 2394 | 6800 | UPSTREAM | CG2331-RA | "-" | CG2331-RB | "-"

note: overlaps known module eve_stripe5 by 500 bases (module coords: 5047275-5048074)

*********************** Rank 50 [Score 24.137024]   GBROWSE*******************

 CG32115 in-situ | CG32115 | - | -33257 | -34863 | UPSTREAM | CG32115-RA | "-"
 CG10752 in-situ | CG10752 | - | 7659 | 5780 | DOWNSTREAM | CG10752-RA | "-"

*********************** Rank 51 [Score 24.086731]   GBROWSE*******************

 CG5488 in-situ | B-H2 | + | -69760 | -60179 | DOWNSTREAM | CG5488-RA | "-"
insitu CG5529 in-situ | B-H1 | + | 13160 | 18938 | UPSTREAM | CG5529-RA | "-"

*********************** Rank 52 [Score 23.975708]   GBROWSE*******************

 CG5732 in-situ | CG5732 | + | -8290 | -3418 | DOWNSTREAM | CG5732-RA | "-"
 CG7084 in-situ | CG7084 | + | 78702 | 83717 | UPSTREAM | CG7084-RB | "-" | CG7084-RA | "-"

*********************** Rank 53 [Score 23.884521]   GBROWSE*******************

insitu CG2819 in-situ | Pph13 | + | -8797 | -6736 | DOWNSTREAM | CG2819-RA | "-"
insitu highlight CG2851 in-situ | Gsc | - | 8402 | -2745 | INTRAGENIC | intron:CG2851-RA:2 | CG2851-RA | "-"

*********************** Rank 54 [Score 23.582153]   GBROWSE*******************

 CG5790 in-situ | CG5790 | + | -10813 | -8440 | DOWNSTREAM | CG5790-RA | "-"
 CG5803 in-situ | Fas3 | + | 14384 | 86873 | UPSTREAM | CG5803-RA | "-" | CG5803-RB | "-"


*********************** Rank 55 [Score 23.516235]   GBROWSE*******************

 CG1031 in-situ | alpha-Est1 | - | -1237 | -4632 | UPSTREAM | CG1031-RA | "-"
 CG32465 in-situ | CG32465 | - | 30906 | 20931 | DOWNSTREAM | CG32465-RB | "-"

*********************** Rank 56 [Score 23.494019]   GBROWSE*******************

 CG10734 in-situ | CG10734 | - | -2181 | -3564 | UPSTREAM | CG10734-RA | "-"
 CG8434 in-situ | CG8434 | + | 3372 | 8545 | UPSTREAM | CG8434-RA | "-"

*********************** Rank 57 [Score 23.489258]   GBROWSE*******************

 CG6717 in-situ | CG6717 | + | -4134 | -2836 | DOWNSTREAM | CG6717-RA | "-"
 CG6730 in-situ | Cyp4d21 | + | 19531 | 21634 | UPSTREAM | CG6730-RA | "-"

*********************** Rank 58 [Score 23.178833]   GBROWSE*******************

 CG4429 in-situ | Rbp2 | + | -22482 | -19005 | DOWNSTREAM | CG4429-RA | "-" | CG4429-RB | "-"
 CG9906 in-situ | CG9906 | - | 54041 | 52122 | DOWNSTREAM | CG9906-RA | "-"

*********************** Rank 59 [Score 23.135742]   GBROWSE*******************

insitu CG10710 in-situ | CG10710 | - | -66281 | -69609 | UPSTREAM | CG10710-RA | "-"
 CG12478 in-situ | bru-3 | - | 80867 | -47964 | INTRAGENIC | intron:CG12478-RA:5 | intron:CG12478-RB:3 | CG12478-RA | "-" | CG12478-RB | "-"

*********************** Rank 60 [Score 23.083740]   GBROWSE*******************

 CG12425 in-situ | CG12425 | + | -5288 | -3733 | DOWNSTREAM | CG12425-RA | "-"
 CG4787 in-situ | CG4787 | + | 82987 | 85273 | UPSTREAM | CG4787-RA | "-"

*********************** Rank 61 [Score 23.045898]   GBROWSE*******************

 CG15631 in-situ | CG15631 | - | -19823 | -21872 | UPSTREAM | CG15631-RA | "-"
 CG15630 in-situ | CG15630 | - | 39761 | -18734 | INTRAGENIC | intron:CG15630-RA:1 | CG15630-RA | "-"

*********************** Rank 62 [Score 23.015137]   GBROWSE*******************

insitu highlight CG4807 in-situ | ab | + | -10460 | 36402 | INTRAGENIC | intron:CG4807-RB:2 | intron:CG4807-RA:2 | CG4807-RB | "-" | CG4807-RA | "-"
 CG32830 in-situ | CG32830 | + | 29895 | 31898 | UPSTREAM | CG32830-RA | "-"

*********************** Rank 63 [Score 22.909790]   GBROWSE*******************

 CG4161 in-situ | BG:DS03023.2 | + | -6603 | -4715 | DOWNSTREAM | CG4161-RA | "-"
insitu CG3956 in-situ | sna | - | 38303 | 36627 | DOWNSTREAM | CG3956-RA | "-"

*********************** Rank 64 [Score 22.827881]   GBROWSE*******************

 CG17927 in-situ | Mhc | + | -1787 | 18400 | INTRAGENIC | intron:CG17927-RG:2 | intron:CG17927-RA:2 | intron:CG17927-RB:2 | intron:CG17927-RC:2 | intron:CG17927-RE:2 | intron:CG17927-RD:2 | intron:CG17927-RF:2 | intron:CG17927-RH:2 | intron:CG17927-RI:2 | intron:CG17927-RJ:2 | intron:CG17927-RL:2 | intron:CG17927-RK:2 | intron:CG17927-RM:2 | CG17927-RG | "-" | CG17927-RA | "-" | CG17927-RB | "-" | CG17927-RC | "-" | CG17927-RE | "-" | CG17927-RD | "-" | CG17927-RF | "-" | CG17927-RH | "-" | CG17927-RI | "-" | CG17927-RJ | "-" | CG17927-RL | "-" | CG17927-RK | "-" | CG17927-RM | "-"
insitu CG13279 in-situ | Cyt-b5-r | - | 20352 | 18491 | DOWNSTREAM | CG13279-RA | "-"


*********************** Rank 65 [Score 22.820251]   GBROWSE*******************

 CG2684 in-situ | lds | + | -21329 | -17274 | DOWNSTREAM | CG2684-RA | "-"
insitu CG11094 in-situ | dsx | - | 25645 | -17440 | INTRAGENIC | intron:CG11094-RA:2 | intron:CG11094-RB:2 | intron:CG11094-RC:2 | CG11094-RA | "-" | CG11094-RB | "-" | CG11094-RC | "-"


*********************** Rank 66 [Score 22.791992]   GBROWSE*******************

 CG15125 in-situ | CG15125 | + | -3947 | -2143 | DOWNSTREAM | CG15125-RA | "-"
 CG11018 in-situ | CG11018 | + | 706 | 2008 | UPSTREAM | CG11018-RA | "-"

*********************** Rank 67 [Score 22.752563]   GBROWSE*******************

 CG15544 in-situ | CG15544 | + | -22962 | -8778 | DOWNSTREAM | CG15544-RA | "-"
insitu highlight CG1378 in-situ | tll | + | 1556 | 3561 | UPSTREAM | CG1378-RA | "-"

note: overlaps known module tll_CD1_anter by 257 bases (module coords: 26666143-26666638)
note: overlaps known module tll_rescue by 500 bases (module coords: 26661256-26671461)

*********************** Rank 68 [Score 22.667969]   GBROWSE*******************

insitu CG3380 in-situ | CG3380 | - | -3210 | -7768 | UPSTREAM | CG3380-RA | "-"
insitu CG5799 in-situ | dve | + | 12514 | 54969 | UPSTREAM | CG5799-RA | "-" | CG5799-RD | "-" | CG5799-RB | "-" | CG5799-RC | "-"


*********************** Rank 69 [Score 22.663879]   GBROWSE*******************

 CG12682 in-situ | CG12682 | + | -5933 | -5220 | DOWNSTREAM | CG12682-RA | "-"
 CG12681 in-situ | CG12681 | + | 43745 | 45208 | UPSTREAM | CG12681-RA | "-"


*********************** Rank 70 [Score 22.401733]   GBROWSE*******************

 CG8517 in-situ | CG8517 | + | -54254 | -53457 | DOWNSTREAM | CG8517-RA | "-"
 CG12501 in-situ | Or56a | - | 2896 | 1124 | DOWNSTREAM | CG12501-RA | "-"

*********************** Rank 71 [Score 22.374146]   GBROWSE*******************

 CG4645 in-situ | CG4645 | + | -18087 | -16125 | DOWNSTREAM | CG4645-RA | "-"
 CG4396 in-situ | fne | + | 14560 | 17763 | UPSTREAM | CG4396-RA | "-"

*********************** Rank 72 [Score 22.286255]   GBROWSE*******************

 CG12478 in-situ | bru-3 | - | -11683 | -140514 | UPSTREAM | CG12478-RA | "-" | CG12478-RB | "-"
 CG8757 in-situ | CG8757 | - | 155120 | 154154 | DOWNSTREAM | CG8757-RA | "-"

*********************** Rank 73 [Score 22.243042]   GBROWSE*******************

 CG15000 in-situ | CG15000 | - | -28 | -2682 | UPSTREAM | CG15000-RA | "-"
 CG15001 in-situ | CG15001 | - | 4125 | 3799 | DOWNSTREAM | CG15001-RA | "-"

*********************** Rank 74 [Score 22.216919]   GBROWSE*******************

 CG13716 in-situ | CG13716 | - | -170 | -523 | UPSTREAM | CG13716-RA | "-"
 CG13715 in-situ | CG13715 | - | 2786 | 2475 | DOWNSTREAM | CG13715-RA | "-"


*********************** Rank 75 [Score 22.199036]   GBROWSE*******************

 CG14362 in-situ | CG14362 | - | -15260 | -16048 | UPSTREAM | CG14362-RA | "-"
 CG9930 in-situ | E5 | - | 3470 | -3211 | INTRAGENIC | intron:CG9930-RA:1 | CG9930-RA | "-"

*********************** Rank 76 [Score 22.197266]   GBROWSE*******************

 CG3587 in-situ | EG:39E1.2 | + | -17492 | -16187 | DOWNSTREAM | CG3587-RA | "-"
 CG32802 in-situ | CG32802 | - | 32401 | 31024 | DOWNSTREAM | CG32802-RA | "-"

*********************** Rank 77 [Score 22.023438]   GBROWSE*******************

 CG12676 in-situ | ed | + | -77802 | 5878 | INTRAGENIC | intron:CG12676-RA:8 | CG12676-RA | "-"
 CG31962 in-situ | Sr-CIII | + | 11182 | 12261 | UPSTREAM | CG31962-RA | "-"

*********************** Rank 78 [Score 22.016113]   GBROWSE*******************

 CG30111 in-situ | CG30111 | + | -15380 | -12001 | DOWNSTREAM | CG30111-RA | "-"
 CG11430 in-situ | olf186-F | + | 13720 | 30845 | UPSTREAM | CG11430-RB | "-" | CG11430-RC | "-" | CG11430-RA | "-"


*********************** Rank 79 [Score 21.932678]   GBROWSE*******************

insitu CG6847 in-situ | CG6847 | + | -41578 | -28875 | DOWNSTREAM | CG6847-RA | "-"
 CG32494 in-situ | CG32494 | + | 46950 | 48187 | UPSTREAM | CG32494-RA | "-"


*********************** Rank 80 [Score 21.829224]   GBROWSE*******************

 CG16898 in-situ | CG16898 | - | -39901 | -41269 | UPSTREAM | CG16898-RA | "-"
 CG8896 in-situ | 18w | + | 66623 | 72044 | UPSTREAM | CG8896-RA | "-"

*********************** Rank 81 [Score 21.800781]   GBROWSE*******************

 CG6633 in-situ | Ugt86Dd | - | -5770 | -7510 | UPSTREAM | CG6633-RA | "-"
 CG4706 in-situ | CG4706 | + | 2967 | 5540 | UPSTREAM | CG4706-RA | "-"

*********************** Rank 82 [Score 21.618225]   GBROWSE*******************

 CG10417 in-situ | CG10417 | - | -26227 | -29004 | UPSTREAM | CG10417-RA | "-" | CG10417-RB | "-"
 CG30437 in-situ | CG30437 | + | 24407 | 64822 | UPSTREAM | CG30437-RA | "-" | CG30437-RC | "-" | CG30437-RB | "-"

*********************** Rank 83 [Score 21.538635]   GBROWSE*******************

insitu highlight CG8704 in-situ | dpn | - | -1026 | -4382 | UPSTREAM | CG8704-RA | "-"
 CG8705 in-situ | pnut | + | 3916 | 6977 | UPSTREAM | CG8705-RB | "-" | CG8705-RA | "-"

*********************** Rank 84 [Score 21.514648]   GBROWSE*******************

 CG31759 in-situ | CG31759 | + | -127402 | -124910 | DOWNSTREAM | CG31759-RB | "-" | CG31759-RA | "-"
 CG31862 in-situ | CG31862 | + | 3841 | 4389 | UPSTREAM | CG31862-RA | "-"

*********************** Rank 85 [Score 21.507812]   GBROWSE*******************

 CG10881 in-situ | CG10881 | + | -14446 | -13563 | DOWNSTREAM | CG10881-RA | "-"
 CG17208 in-situ | CG17208 | - | 15213 | 14878 | DOWNSTREAM | CG17208-RA | "-"


*********************** Rank 86 [Score 21.507446]   GBROWSE*******************

 CG15001 in-situ | CG15001 | - | -375 | -701 | UPSTREAM | CG15001-RA | "-"
 CG15002 in-situ | mas | - | 6437 | 630 | DOWNSTREAM | CG15002-RB | "-"

*********************** Rank 87 [Score 21.349121]   GBROWSE*******************

insitu highlight CG2047 in-situ | ftz | + | -3953 | -2049 | DOWNSTREAM | CG2047-RA | "-"
 CG31488 in-situ | CG31488 | + | 89457 | 90086 | UPSTREAM | CG31488-RA | "-"

note: overlaps known module ftz_3prime_element by 37 bases (module coords: 2692342-2694036)
note: overlaps known module ftz_rescue_construct by 37 bases (module coords: 2683630-2694036)

*********************** Rank 88 [Score 21.308533]   GBROWSE*******************

insitu highlight CG32423 in-situ | CG32423 | - | -44148 | -161416 | UPSTREAM | CG32423-RA | "-" | CG32423-RD | "-" | CG32423-RB | "-" | CG32423-RC | "-"
 CG10645 in-situ | lama | - | 33831 | 21523 | DOWNSTREAM | CG10645-RC | "-" | CG10645-RB | "-" | CG10645-RA | "-"

*********************** Rank 89 [Score 21.307861]   GBROWSE*******************

insitu CG10710 in-situ | CG10710 | - | -20281 | -23609 | UPSTREAM | CG10710-RA | "-"
 CG12478 in-situ | bru-3 | - | 126867 | -1964 | INTRAGENIC | intron:CG12478-RA:10 | intron:CG12478-RB:8 | CG12478-RA | "-" | CG12478-RB | "-"

*********************** Rank 90 [Score 21.299805]   GBROWSE*******************

 CG11692 in-situ | CG11692 | - | -13272 | -13828 | UPSTREAM | CG11692-RA | "-"
 CG1829 in-situ | Cyp6v1 | + | 5884 | 9353 | UPSTREAM | CG1829-RA | "-"

*********************** Rank 91 [Score 21.261230]   GBROWSE*******************

 CG31769 in-situ | CG31769 | - | -9809 | -10957 | UPSTREAM | CG31769-RA | "-"
 CG15292 in-situ | CG15292 | - | 6617 | 6447 | DOWNSTREAM | CG15292-RA | "-"


*********************** Rank 92 [Score 21.233032]   GBROWSE*******************

 CG1829 in-situ | Cyp6v1 | + | -5166 | -1697 | DOWNSTREAM | CG1829-RA | "-"
 CG1835 in-situ | CG1835 | + | 5372 | 7506 | UPSTREAM | CG1835-RA | "-" | CG1835-RB | "-"


*********************** Rank 93 [Score 21.210938]   GBROWSE*******************

 CG12493 in-situ | CG12493 | - | -3491 | -4816 | UPSTREAM | CG12493-RA | "-"
 CG13711 in-situ | CG13711 | + | 820 | 1203 | UPSTREAM | CG13711-RA | "-"

*********************** Rank 94 [Score 21.173279]   GBROWSE*******************

 CG12835 in-situ | CG12835 | + | -8606 | -7620 | DOWNSTREAM | CG12835-RA | "-"
 CG4485 in-situ | Cyp9b1 | - | 14259 | 12289 | DOWNSTREAM | CG4485-RA | "-"

*********************** Rank 95 [Score 21.143555]   GBROWSE*******************

 CG10349 in-situ | CG10349 | + | -12085 | -6703 | DOWNSTREAM | CG10349-RA | "-" | CG10349-RB | "-"
 CG31270 in-situ | CG31270 | - | 31972 | 30856 | DOWNSTREAM | CG31270-RA | "-"

*********************** Rank 96 [Score 21.125793]   GBROWSE*******************

 CG3811 in-situ | CG3811 | + | -6556 | 12274 | INTRAGENIC | intron:CG3811-RA:2 | intron:CG3811-RB:2 | CG3811-RA | "-" | CG3811-RB | "-"
 CG31883 in-situ | CG31883 | + | 752 | 1877 | UPSTREAM | CG31883-RA | "-"

*********************** Rank 97 [Score 21.123596]   GBROWSE*******************

 CG4481 in-situ | Glu-RIB | + | -14966 | 18071 | INTRAGENIC | intron:CG4481-RA:7 | CG4481-RA | "-"
 CG32042 in-situ | PGRP-LA | + | 66943 | 70548 | UPSTREAM | CG32042-RB | "-" | CG32042-RA | "-"

*********************** Rank 98 [Score 21.113647]   GBROWSE*******************

 CG6604 in-situ | H15 | + | -40293 | -28707 | DOWNSTREAM | CG6604-RA | "-"
 CG31647 in-situ | CG31647 | - | 4567 | -9056 | INTRAGENIC | intron:CG31647-RA:3 | CG31647-RA | "-" | CG31647-RB | "-"

*********************** Rank 99 [Score 21.105652]   GBROWSE*******************

 CG3647 in-situ | stc | + | -48133 | -43545 | DOWNSTREAM | CG3647-RB | "-" | CG3647-RA | "-"
 CG4168 in-situ | BG:DS03192.2 | - | 16705 | 492 | DOWNSTREAM | CG4168-RA | "-"

*********************** Rank 100 [Score 20.973816]   GBROWSE*******************

insitu CG12701 in-situ | CG12701 | - | -18362 | -25303 | UPSTREAM | CG12701-RB | "-" | CG12701-RA | "-"
 CG12700 in-situ | skpD | + | 11439 | 12098 | UPSTREAM | CG12700-RA | "-"


*********************** Rank 101 [Score 20.971680]   GBROWSE*******************

insitu highlight CG9786 in-situ | hb | - | -1345 | -7847 | UPSTREAM | CG9786-RB | "-" | CG9786-RA | "-"
insitu CG8112 in-situ | CG8112 | + | 1819 | 16105 | UPSTREAM | CG8112-RA | "-" | CG8112-RB | "-"

*********************** Rank 102 [Score 20.944458]   GBROWSE*******************

 CG17686 in-situ | DIP1 | - | -20232 | -25594 | UPSTREAM | CG17686-RA | "-" | CG17686-RB | "-" | CG17686-RC | "-" | CG17686-RD | "-"
 CG14621 in-situ | CG14621 | + | 55319 | 57746 | UPSTREAM | CG14621-RA | "-"

*********************** Rank 103 [Score 20.857056]   GBROWSE*******************

 CG17839 in-situ | CG17839 | + | -23318 | 53163 | INTRAGENIC | intron:CG17839-RA:2 | CG17839-RA | "-"
 CG13467 in-situ | CG13467 | + | 75543 | 76566 | UPSTREAM | CG13467-RA | "-"

*********************** Rank 104 [Score 20.850830]   GBROWSE*******************

 CG31031 in-situ | CG31031 | + | -15784 | -15320 | DOWNSTREAM | CG31031-RA | "-"
 CG18682 in-situ | CG18682 | - | 45873 | 43192 | DOWNSTREAM | CG18682-RA | "-"

*********************** Rank 105 [Score 20.842651]   GBROWSE*******************

 CG15405 in-situ | CG15405 | - | -4845 | -9194 | UPSTREAM | CG15405-RA | "-"
 CG3347 in-situ | CG3347 | + | 62071 | 73965 | UPSTREAM | CG3347-RA | "-"


*********************** Rank 106 [Score 20.804932]   GBROWSE*******************

 CG5103 in-situ | CG5103 | - | -354 | -2604 | UPSTREAM | CG5103-RA | "-"
 CG13700 in-situ | CG13700 | - | 6539 | 4166 | DOWNSTREAM | CG13700-RA | "-"

*********************** Rank 107 [Score 20.731995]   GBROWSE*******************

 CG2616 in-situ | CG2616 | + | -10646 | -8645 | DOWNSTREAM | CG2616-RA | "-"
 CG3027 in-situ | pyd3 | - | 17013 | 14850 | DOWNSTREAM | CG3027-RA | "-"

*********************** Rank 108 [Score 20.711304]   GBROWSE*******************

insitu CG4145 in-situ | Cg25C | + | -2814 | 4685 | INTRAGENIC | intron:CG4145-RC:5 | intron:CG4145-RB:5 | intron:CG4145-RA:5 | CG4145-RC | "-" | CG4145-RB | "-" | CG4145-RA | "-"
insitu CG14041 in-situ | SP555 | + | 5624 | 8389 | UPSTREAM | CG14041-RA | "-" | CG14041-RB | "-"

*********************** Rank 109 [Score 20.703003]   GBROWSE*******************

 CG32725 in-situ | CG32725 | - | -27962 | -28689 | UPSTREAM | CG32725-RA | "-"
 CG1958 in-situ | CG1958 | + | 781 | 1758 | UPSTREAM | CG1958-RA | "-"

*********************** Rank 110 [Score 20.605713]   GBROWSE*******************

 CG9445 in-situ | CG9445 | - | -9871 | -10724 | UPSTREAM | CG9445-RA | "-"
 CG9446 in-situ | coro | - | 23971 | 13203 | DOWNSTREAM | CG9446-RA | "-" | CG9446-RB | "-"

*********************** Rank 111 [Score 20.589111]   GBROWSE*******************

insitu CG5799 in-situ | dve | + | -33786 | 8669 | INTRAGENIC | intron:CG5799-RA:2 | intron:CG5799-RD:2 | intron:CG5799-RC:3 | CG5799-RA | "-" | CG5799-RD | "-" | CG5799-RB | "-" | CG5799-RC | "-"
insitu CG5819 in-situ | CG5819 | + | 15710 | 19209 | UPSTREAM | CG5819-RA | "-" | CG5819-RB | "-"

*********************** Rank 112 [Score 20.586487]   GBROWSE*******************

 CG12110 in-situ | Pld | + | -92211 | -78969 | DOWNSTREAM | CG12110-RB | "-" | CG12110-RC | "-" | CG12110-RA | "-" | CG12110-RE | "-" | CG12110-RD | "-"
 CG9397 in-situ | 1.28 | + | 90970 | 91920 | UPSTREAM | CG9397-RA | "-"


*********************** Rank 113 [Score 20.570312]   GBROWSE*******************

 CG16758 in-situ | CG16758 | - | -2587 | -7467 | UPSTREAM | CG16758-RB | "-" | CG16758-RD | "-" | CG16758-RC | "-"
 CG32295 in-situ | CG32295 | - | 20801 | 19646 | DOWNSTREAM | CG32295-RA | "-"

*********************** Rank 114 [Score 20.567993]   GBROWSE*******************

 CG7320 in-situ | CG7320 | + | -41332 | -39605 | DOWNSTREAM | CG7320-RA | "-"
 CG7313 in-situ | CG7313 | + | 8926 | 9675 | UPSTREAM | CG7313-RA | "-"

*********************** Rank 115 [Score 20.491394]   GBROWSE*******************

 CG6604 in-situ | H15 | + | -9593 | 1993 | INTRAGENIC | intron:CG6604-RA:4 | CG6604-RA | "-"
 CG31647 in-situ | CG31647 | - | 35267 | 21644 | DOWNSTREAM | CG31647-RA | "-" | CG31647-RB | "-"


*********************** Rank 116 [Score 20.484924]   GBROWSE*******************

 CG1867 in-situ | Or98b | + | -8270 | -6892 | DOWNSTREAM | CG1867-RA | "-"
 CG14064 in-situ | beat-VI | + | 57692 | 112697 | UPSTREAM | CG14064-RA | "-"

*********************** Rank 117 [Score 20.463623]   GBROWSE*******************

 CG14362 in-situ | CG14362 | - | -4160 | -4948 | UPSTREAM | CG14362-RA | "-"
 CG9930 in-situ | E5 | - | 14570 | 7889 | DOWNSTREAM | CG9930-RA | "-"

*********************** Rank 118 [Score 20.437378]   GBROWSE*******************

 CG17697 in-situ | fz | + | -15273 | 79028 | INTRAGENIC | intron:CG17697-RB:1 | intron:CG17697-RA:1 | CG17697-RB | "-" | CG17697-RA | "-"
 CG13482 in-situ | CG13482 | + | 39817 | 40125 | UPSTREAM | CG13482-RA | "-"

*********************** Rank 119 [Score 20.427795]   GBROWSE*******************

 CG5646 in-situ | CG5646 | - | -5763 | -7690 | UPSTREAM | CG5646-RA | "-"
 CG5643 in-situ | BcDNA:LD34343 | - | 15176 | -2025 | INTRAGENIC | intron:CG5643-RB:4 | intron:CG5643-RD:5 | intron:CG5643-RF:4 | intron:CG5643-RG:5 | intron:CG5643-RA:4 | intron:CG5643-RC:4 | intron:CG5643-RE:5 | CG5643-RB | "-" | CG5643-RD | "-" | CG5643-RF | "-" | CG5643-RG | "-" | CG5643-RA | "-" | CG5643-RC | "-" | CG5643-RE | "-"


*********************** Rank 120 [Score 20.423340]   GBROWSE*******************

 CG4168 in-situ | BG:DS03192.2 | - | -35195 | -51408 | UPSTREAM | CG4168-RA | "-"
 CG3994 in-situ | BG:DS07295.1 | - | 34317 | 15214 | DOWNSTREAM | CG3994-RA | "-" | CG3994-RB | "-"

*********************** Rank 121 [Score 20.414917]   GBROWSE*******************

insitu CG7011 in-situ | CG7011 | - | -36297 | -39052 | UPSTREAM | CG7011-RA | "-"
 CG6888 in-situ | CG6888 | + | 7923 | 8671 | UPSTREAM | CG6888-RA | "-"

*********************** Rank 122 [Score 20.402954]   GBROWSE*******************

 CG2684 in-situ | lds | + | -11079 | -7024 | DOWNSTREAM | CG2684-RA | "-"
insitu CG11094 in-situ | dsx | - | 35895 | -7190 | INTRAGENIC | intron:CG11094-RA:3 | CG11094-RA | "-" | CG11094-RB | "-" | CG11094-RC | "-"


*********************** Rank 123 [Score 20.401733]   GBROWSE*******************

 CG12535 in-situ | CG12535 | - | -19748 | -20533 | UPSTREAM | CG12535-RB | "-" | CG12535-RA | "-"
 CG14269 in-situ | CG14269 | - | 22605 | 21926 | DOWNSTREAM | CG14269-RA | "-"


*********************** Rank 124 [Score 20.389038]   GBROWSE*******************

 CG13723 in-situ | CG13723 | + | -19520 | -19188 | DOWNSTREAM | CG13723-RA | "-"
 CG6485 in-situ | CG6485 | - | 36790 | 35941 | DOWNSTREAM | CG6485-RA | "-"


*********************** Rank 125 [Score 20.370361]   GBROWSE*******************

 CG5034 in-situ | GATAd | + | -3027 | 1515 | INTRAGENIC | intron:CG5034-RA:3 | CG5034-RA | "-"
 CG5029 in-situ | SamDC | + | 2519 | 4564 | UPSTREAM | CG5029-RA | "-" | CG5029-RB | "-"

*********************** Rank 126 [Score 20.360107]   GBROWSE*******************

 CG5406 in-situ | sif | + | -26322 | 53897 | INTRAGENIC | intron:CG5406-RB:12 | intron:CG5406-RA:1 | CG5406-RB | "-" | CG5406-RA | "-"
 CG32414 in-situ | CG32414 | + | 46483 | 47906 | UPSTREAM | CG32414-RA | "-"


*********************** Rank 127 [Score 20.296509]   GBROWSE*******************

 CG30448 in-situ | Obp56i | + | -4071 | -3591 | DOWNSTREAM | CG30448-RA | "-"
 CG13872 in-situ | CG13872 | - | 8733 | 6641 | DOWNSTREAM | CG13872-RA | "-"

*********************** Rank 128 [Score 20.257141]   GBROWSE*******************

 CG5971 in-situ | CG5971 | - | -2209 | -4781 | UPSTREAM | CG5971-RA | "-"
insitu CG32029 in-situ | CG32029 | + | 4184 | 7289 | UPSTREAM | CG32029-RA | "-"


*********************** Rank 129 [Score 20.249268]   GBROWSE*******************

 CG13952 in-situ | CG13952 | + | -3119 | -1623 | DOWNSTREAM | CG13952-RA | "-"
 CG13953 in-situ | CG13953 | + | 6022 | 6786 | UPSTREAM | CG13953-RA | "-"


*********************** Rank 130 [Score 20.211609]   GBROWSE*******************

 CG10037 in-situ | vvl | + | -91111 | -86735 | DOWNSTREAM | CG10037-RA | "-"
insitu CG10078 in-situ | Prat2 | - | 33587 | 30722 | DOWNSTREAM | CG10078-RB | "-" | CG10078-RA | "-"

*********************** Rank 131 [Score 20.197144]   GBROWSE*******************

 CG15186 in-situ | CG15186 | + | -3781 | 8456 | INTRAGENIC | intron:CG15186-RA:1 | CG15186-RA | "-" | CG15186-RB | "-"
 CG15185 in-situ | CG15185 | + | 40952 | 41980 | UPSTREAM | CG15185-RA | "-"


*********************** Rank 132 [Score 20.188660]   GBROWSE*******************

 CG6486 in-situ | CG6486 | + | -14966 | -13774 | DOWNSTREAM | CG6486-RA | "-"
insitu highlight CG6494 in-situ | h | + | 11469 | 14749 | UPSTREAM | CG6494-RA | "-"

note: overlaps known module h_rescue by 500 bases (module coords: 8620516-8642090)
note: overlaps known module h_stripe3_4_1991 by 349 bases (module coords: 8622071-8623548)
note: overlaps known module h_stripe3_4_1990 by 500 bases (module coords: 8622240-8623984)
note: overlaps known module h_stripe3 by 349 bases (module coords: 8623073-8623548)
note: overlaps known module h_stripe4 by 500 bases (module coords: 8623073-8623984)
note: overlaps known module h_stripe7_1990a by 152 bases (module coords: 8623548-8625021)

*********************** Rank 133 [Score 20.178955]   GBROWSE*******************

 CG31934 in-situ | CG31934 | - | -18119 | -18803 | UPSTREAM | CG31934-RA | "-"
 CG17158 in-situ | cpb | + | 15469 | 17344 | UPSTREAM | CG17158-RA | "-"

*********************** Rank 134 [Score 20.151123]   GBROWSE*******************

 CG31081 in-situ | CG31081 | + | -1139 | -96 | DOWNSTREAM | CG31081-RA | "-"
 CG14242 in-situ | CG14242 | + | 701 | 1447 | UPSTREAM | CG14242-RA | "-"


*********************** Rank 135 [Score 20.127686]   GBROWSE*******************

 CG7000 in-situ | CG7000 | - | -42533 | -44947 | UPSTREAM | CG7000-RA | "-"
 CG5862 in-situ | CG5862 | + | 4411 | 5567 | UPSTREAM | CG5862-RA | "-"


*********************** Rank 136 [Score 20.112061]   GBROWSE*******************

 CG2014 in-situ | CG2014 | + | -7217 | -6579 | DOWNSTREAM | CG2014-RA | "-"
insitu highlight CG1897 in-situ | Dr | + | 33325 | 42223 | UPSTREAM | CG1897-RA | "-"


*********************** Rank 137 [Score 20.088745]   GBROWSE*******************

insitu CG3619 in-situ | Dl | - | -40648 | -64130 | UPSTREAM | CG3619-RA | "-" | CG3619-RB | "-"
 CG3581 in-situ | CG3581 | - | 7888 | 6896 | DOWNSTREAM | CG3581-RA | "-"


*********************** Rank 138 [Score 20.077026]   GBROWSE*******************

insitu highlight CG2102 in-situ | cas | - | -7981 | -12339 | UPSTREAM | CG2102-RA | "-" | CG2102-RB | "-"
 CG1239 in-situ | CG1239 | + | 8260 | 9421 | UPSTREAM | CG1239-RA | "-"

*********************** Rank 139 [Score 20.044922]   GBROWSE*******************

 CG6559 in-situ | CG6559 | - | -61758 | -77996 | UPSTREAM | CG6559-RA | "-"
 CG12362 in-situ | CG12362 | + | 38345 | 40306 | UPSTREAM | CG12362-RB | "-" | CG12362-RA | "-"

*********************** Rank 140 [Score 20.033447]   GBROWSE*******************

insitu CG10191 in-situ | CG10191 | - | -2344 | -4039 | UPSTREAM | CG10191-RA | "-"
 CG32120 in-situ | Ly | - | 8793 | 3897 | DOWNSTREAM | CG32120-RA | "-"

*********************** Rank 141 [Score 19.968872]   GBROWSE*******************

 CG8279 in-situ | CG8279 | - | -21430 | -37369 | UPSTREAM | CG8279-RA | "-"
 CG14854 in-situ | CG14854 | - | 9711 | 8001 | DOWNSTREAM | CG14854-RA | "-"

*********************** Rank 142 [Score 19.939331]   GBROWSE*******************

 CG31832 in-situ | CG31832 | - | -895 | -1459 | UPSTREAM | CG31832-RA | "-"
 CG4182 in-situ | yellow-c | + | 2856 | 5227 | UPSTREAM | CG4182-RA | "-"


*********************** Rank 143 [Score 19.914185]   GBROWSE*******************

 CG12478 in-situ | bru-3 | - | -4683 | -133514 | UPSTREAM | CG12478-RA | "-" | CG12478-RB | "-"
 CG8757 in-situ | CG8757 | - | 162120 | 161154 | DOWNSTREAM | CG8757-RA | "-"

*********************** Rank 144 [Score 19.880737]   GBROWSE*******************

 CG14677 in-situ | CG14677 | - | -6434 | -10831 | UPSTREAM | CG14677-RA | "-"
 CG14678 in-situ | CG14678 | - | 627 | -2174 | INTRAGENIC | intron:CG14678-RA:3 | CG14678-RA | "-"

*********************** Rank 145 [Score 19.868286]   GBROWSE*******************

 CG8705 in-situ | pnut | + | -15584 | -12523 | DOWNSTREAM | CG8705-RB | "-" | CG8705-RA | "-"
 CG14760 in-situ | CG14760 | + | 1403 | 3883 | UPSTREAM | CG14760-RA | "-"


*********************** Rank 146 [Score 19.841064]   GBROWSE*******************

 CG3473 in-situ | BG:DS01486.1 | - | -27522 | -28176 | UPSTREAM | CG3473-RA | "-"
insitu CG32954 in-situ | CG32954 | + | 24307 | 27654 | UPSTREAM | CG32954-RA | "-" | CG32954-RB | "-" | CG32954-RC | "-" | CG32954-RG | "-" | CG32954-RH | "-" | CG32954-RF | "-" | CG32954-RD | "-" | CG32954-RE | "-"

*********************** Rank 147 [Score 19.835205]   GBROWSE*******************

 CG9397 in-situ | 1.28 | + | -57430 | -56480 | DOWNSTREAM | CG9397-RA | "-"
 CG15233 in-situ | CG15233 | - | 40780 | 39707 | DOWNSTREAM | CG15233-RA | "-"

*********************** Rank 148 [Score 19.780029]   GBROWSE*******************

 CG15636 in-situ | CG15636 | - | -56028 | -56348 | UPSTREAM | CG15636-RA | "-"
 CG15635 in-situ | CG15635 | + | 8985 | 12341 | UPSTREAM | CG15635-RA | "-"

*********************** Rank 149 [Score 19.778564]   GBROWSE*******************

 CG30111 in-situ | CG30111 | + | -9680 | -6301 | DOWNSTREAM | CG30111-RA | "-"
 CG11430 in-situ | olf186-F | + | 19420 | 36545 | UPSTREAM | CG11430-RB | "-" | CG11430-RC | "-" | CG11430-RA | "-"

*********************** Rank 150 [Score 19.765747]   GBROWSE*******************

 CG3606 in-situ | caz | + | -5985 | -1317 | DOWNSTREAM | CG3606-RB | "-" | CG3606-RA | "-"
 CG3632 in-situ | CG3632 | + | 1883 | 8487 | UPSTREAM | CG3632-RD | "-" | CG3632-RC | "-" | CG3632-RA | "-"


*********************** Rank 151 [Score 19.720703]   GBROWSE*******************

 CG4328 in-situ | CG4328 | - | -27841 | -29767 | UPSTREAM | CG4328-RA | "-"
insitu highlight CG32105 in-situ | CG32105 | + | 8433 | 15103 | UPSTREAM | CG32105-RB | "-"

*********************** Rank 152 [Score 19.698914]   GBROWSE*******************

 CG12538 in-situ | CG12538 | - | -11424 | -12008 | UPSTREAM | CG12538-RA | "-"
insitu CG31337 in-situ | CG31337 | - | 10425 | 9013 | DOWNSTREAM | CG31337-RA | "-"


*********************** Rank 153 [Score 19.681519]   GBROWSE*******************

 CG14061 in-situ | CG14061 | - | -9628 | -10728 | UPSTREAM | CG14061-RA | "-"
 CG12558 in-situ | CG12558 | + | 22660 | 23599 | UPSTREAM | CG12558-RA | "-"


*********************** Rank 154 [Score 19.651428]   GBROWSE*******************

 CG18405 in-situ | Sema-1a | + | -38423 | 65210 | INTRAGENIC | intron:CG18405-RA:1 | CG18405-RA | "-"
 CG9280 in-situ | Glt | + | 69824 | 74592 | UPSTREAM | CG9280-RC | "-" | CG9280-RB | "-" | CG9280-RA | "-"


*********************** Rank 155 [Score 19.630493]   GBROWSE*******************

 CG8454 in-situ | CG8454 | + | -40480 | -37276 | DOWNSTREAM | CG8454-RA | "-"
 CG8120 in-situ | CG8120 | + | 14314 | 14838 | UPSTREAM | CG8120-RA | "-"

*********************** Rank 156 [Score 19.616577]   GBROWSE*******************

 CG5897 in-situ | CG5897 | - | -15430 | -18217 | UPSTREAM | CG5897-RA | "-"
 CG11588 in-situ | CG11588 | + | 15464 | 16349 | UPSTREAM | CG11588-RA | "-"

*********************** Rank 157 [Score 19.601074]   GBROWSE*******************

insitu CG4999 in-situ | Tsp66E | + | -15141 | 4425 | INTRAGENIC | intron:CG4999-RB:4 | intron:CG4999-RA:4 | intron:CG4999-RC:4 | CG4999-RB | "-" | CG4999-RA | "-" | CG4999-RC | "-"
 CG5741 in-situ | CG5741 | - | 9173 | 4590 | DOWNSTREAM | CG5741-RA | "-"

*********************** Rank 158 [Score 19.564148]   GBROWSE*******************

 CG8519 in-situ | CG8519 | + | -3272 | -2274 | DOWNSTREAM | CG8519-RA | "-"
insitu CG10118 in-situ | ple | - | 8303 | 2134 | DOWNSTREAM | CG10118-RA | "-" | CG10118-RB | "-"

*********************** Rank 159 [Score 19.536987]   GBROWSE*******************

 CG32666 in-situ | CG32666 | + | -40191 | 21685 | INTRAGENIC | intron:CG32666-RB:2 | CG32666-RB | "-"
 CG1572 in-situ | CG1572 | - | 25274 | 21920 | DOWNSTREAM | CG1572-RA | "-" | CG1572-RB | "-"

*********************** Rank 160 [Score 19.536377]   GBROWSE*******************

 CG9116 in-situ | LysP | - | -7575 | -8261 | UPSTREAM | CG9116-RA | "-"
 CG1165 in-situ | LysS | + | 1476 | 1975 | UPSTREAM | CG1165-RA | "-"

*********************** Rank 161 [Score 19.503540]   GBROWSE*******************

insitu highlight CG4717 in-situ | kni | - | -4649 | -7682 | UPSTREAM | CG4717-RA | "-"
insitu CG13253 in-situ | CG13253 | - | 19186 | 16783 | DOWNSTREAM | CG13253-RA | "-"


*********************** Rank 162 [Score 19.502441]   GBROWSE*******************

insitu CG7891 in-situ | CG7891 | + | -9449 | -7217 | DOWNSTREAM | CG7891-RA | "-"
 CG9656 in-situ | grn | - | 45379 | 11287 | DOWNSTREAM | CG9656-RA | "-"

*********************** Rank 163 [Score 19.499023]   GBROWSE*******************

 CG1338 in-situ | CG1338 | - | -3644 | -8056 | UPSTREAM | CG1338-RA | "-" | CG1338-RB | "-"
insitu highlight CG1849 in-situ | run | + | 15556 | 18441 | UPSTREAM | CG1849-RA | "-"

*********************** Rank 164 [Score 19.492065]   GBROWSE*******************

 CG4959 in-situ | BG:DS04095.3 | - | -22503 | -25309 | UPSTREAM | CG4959-RB | "-"
 CG4894 in-situ | Ca-alpha1D | + | 10641 | 30070 | UPSTREAM | CG4894-RA | "-" | CG4894-RB | "-" | CG4894-RC | "-" | CG4894-RD | "-"

*********************** Rank 165 [Score 19.474670]   GBROWSE*******************

 CG32080 in-situ | CG32080 | + | -16466 | -15705 | DOWNSTREAM | CG32080-RA | "-"
 CG12296 in-situ | klu | - | 16756 | -10342 | INTRAGENIC | intron:CG12296-RA:2 | CG12296-RA | "-"

*********************** Rank 166 [Score 19.471802]   GBROWSE*******************

 CG31209 in-situ | CG31209 | + | -11296 | 8168 | INTRAGENIC | intron:CG31209-RA:3 | CG31209-RA | "-"
 CG5060 in-situ | CG5060 | + | 16272 | 54607 | UPSTREAM | CG5060-RA | "-"

*********************** Rank 167 [Score 19.427185]   GBROWSE*******************

 CG9922 in-situ | CG9922 | - | -9044 | -10193 | UPSTREAM | CG9922-RA | "-"
 CG3143 in-situ | foxo | + | 11763 | 42191 | UPSTREAM | CG3143-RA | "-"

*********************** Rank 168 [Score 19.415710]   GBROWSE*******************

insitu highlight CG31670 in-situ | CG31670 | - | -3666 | -7950 | UPSTREAM | CG31670-RA | "-"
 CG10908 in-situ | CG10908 | + | 15960 | 17237 | UPSTREAM | CG10908-RA | "-"

*********************** Rank 169 [Score 19.414124]   GBROWSE*******************

 CG5462 in-situ | scrib | + | -26117 | 33727 | INTRAGENIC | intron:CG5462-RA:5 | intron:CG5462-RB:5 | intron:CG5462-RC:5 | intron:CG5462-RD:5 | CG5462-RA | "-" | CG5462-RB | "-" | CG5462-RC | "-" | CG5462-RD | "-"
 CG31082 in-situ | CG31082 | + | 21897 | 24338 | UPSTREAM | CG31082-RA | "-"


*********************** Rank 170 [Score 19.414124]   GBROWSE*******************

 CG8454 in-situ | CG8454 | + | -41630 | -38426 | DOWNSTREAM | CG8454-RA | "-"
 CG8120 in-situ | CG8120 | + | 13164 | 13688 | UPSTREAM | CG8120-RA | "-"


*********************** Rank 171 [Score 19.407593]   GBROWSE*******************

insitu CG5799 in-situ | dve | + | -44336 | -1881 | DOWNSTREAM | CG5799-RA | "-" | CG5799-RD | "-" | CG5799-RB | "-" | CG5799-RC | "-"
insitu CG5819 in-situ | CG5819 | + | 5160 | 8659 | UPSTREAM | CG5819-RA | "-" | CG5819-RB | "-"

*********************** Rank 172 [Score 19.378723]   GBROWSE*******************

 CG11755 in-situ | CG11755 | + | -6178 | -5714 | DOWNSTREAM | CG11755-RA | "-"
insitu highlight CG9786 in-situ | hb | - | 13355 | 6853 | DOWNSTREAM | CG9786-RB | "-" | CG9786-RA | "-"


*********************** Rank 173 [Score 19.338196]   GBROWSE*******************

 CG8853 in-situ | CG8853 | + | -7428 | -5805 | DOWNSTREAM | CG8853-RA | "-"
insitu highlight CG10016 in-situ | drm | + | 920 | 9755 | UPSTREAM | CG10016-RB | "-" | CG10016-RA | "-"

*********************** Rank 174 [Score 19.324097]   GBROWSE*******************

 CG14506 in-situ | CG14506 | - | -8783 | -10193 | UPSTREAM | CG14506-RA | "-"
 CG11958 in-situ | Cnx99A | - | 13632 | 9239 | DOWNSTREAM | CG11958-RA | "-" | CG11958-RB | "-"

*********************** Rank 175 [Score 19.305542]   GBROWSE*******************

insitu CG7018 in-situ | Ets65A | + | -18120 | 7688 | INTRAGENIC | intron:CG7018-RA:7 | intron:CG7018-RB:4 | CG7018-RA | "-" | CG7018-RB | "-"
 CG12755 in-situ | l(3)mbn | - | 13191 | 7644 | DOWNSTREAM | CG12755-RA | "-" | CG12755-RB | "-"

*********************** Rank 176 [Score 19.305176]   GBROWSE*******************

 CG6154 in-situ | CG6154 | + | -40601 | -33075 | DOWNSTREAM | CG6154-RA | "-" | CG6154-RB | "-"
 CG14559 in-situ | CG14559 | + | 15095 | 28384 | UPSTREAM | CG14559-RA | "-"

*********************** Rank 177 [Score 19.291748]   GBROWSE*******************

 CG11368 in-situ | CG11368 | + | -6415 | -5960 | DOWNSTREAM | CG11368-RA | "-"
 CG32719 in-situ | CG32719 | - | 54220 | 50268 | DOWNSTREAM | CG32719-RA | "-"

*********************** Rank 178 [Score 19.291382]   GBROWSE*******************

 CG8896 in-situ | 18w | + | -26127 | -20706 | DOWNSTREAM | CG8896-RA | "-"
 CG11041 in-situ | CG11041 | + | 8509 | 9591 | UPSTREAM | CG11041-RA | "-"

*********************** Rank 179 [Score 19.288025]   GBROWSE*******************

 CG13785 in-situ | CG13785 | + | -15286 | -14389 | DOWNSTREAM | CG13785-RA | "-"
insitu highlight CG4889 in-situ | wg | + | 9342 | 18436 | UPSTREAM | CG4889-RA | "-" | CG4889-RB | "-"

*********************** Rank 180 [Score 19.283081]   GBROWSE*******************

 CG10869 in-situ | CG10869 | - | -10764 | -13176 | UPSTREAM | CG10869-RA | "-"
 CG31935 in-situ | CG31935 | - | 13091 | -2991 | INTRAGENIC | intron:CG31935-RA:2 | CG31935-RA | "-"

*********************** Rank 181 [Score 19.275635]   GBROWSE*******************

 CG14925 in-situ | CG14925 | - | -10026 | -11043 | UPSTREAM | CG14925-RA | "-"
 CG14926 in-situ | CG14926 | - | 47634 | 46684 | DOWNSTREAM | CG14926-RA | "-"


*********************** Rank 182 [Score 19.249023]   GBROWSE*******************

 CG1338 in-situ | CG1338 | - | -5444 | -9856 | UPSTREAM | CG1338-RA | "-" | CG1338-RB | "-"
insitu highlight CG1849 in-situ | run | + | 13756 | 16641 | UPSTREAM | CG1849-RA | "-"

note: overlaps known module run_stripe1 by 500 bases (module coords: 20352427-20354043)
note: overlaps known module run_stripe7 by 500 bases (module coords: 20352427-20358012)

*********************** Rank 183 [Score 19.232910]   GBROWSE*******************

 CG5462 in-situ | scrib | + | -25067 | 34777 | INTRAGENIC | intron:CG5462-RA:5 | intron:CG5462-RB:5 | intron:CG5462-RC:5 | intron:CG5462-RD:5 | CG5462-RA | "-" | CG5462-RB | "-" | CG5462-RC | "-" | CG5462-RD | "-"
 CG31082 in-situ | CG31082 | + | 22947 | 25388 | UPSTREAM | CG31082-RA | "-"

*********************** Rank 184 [Score 19.213806]   GBROWSE*******************

 CG1373 in-situ | CecC | + | -7228 | -6775 | DOWNSTREAM | CG1373-RA | "-"
 CG9737 in-situ | CG9737 | - | 19598 | 17551 | DOWNSTREAM | CG9737-RA | "-"

*********************** Rank 185 [Score 19.201050]   GBROWSE*******************

 CG6621 in-situ | CG6621 | - | -37130 | -40892 | UPSTREAM | CG6621-RA | "-"
 CG4683 in-situ | CG4683 | + | 19659 | 21014 | UPSTREAM | CG4683-RA | "-"

*********************** Rank 186 [Score 19.157959]   GBROWSE*******************

 CG15483 in-situ | CG15483 | - | -12690 | -14074 | UPSTREAM | CG15483-RA | "-"
 CG12283 in-situ | kek1 | - | 48257 | 44400 | DOWNSTREAM | CG12283-RA | "-"


*********************** Rank 187 [Score 19.156921]   GBROWSE*******************

 CG15167 in-situ | CG15167 | + | -6807 | -6457 | DOWNSTREAM | CG15167-RA | "-"
 CG31753 in-situ | CG31753 | - | 29157 | 16277 | DOWNSTREAM | CG31753-RA | "-"


*********************** Rank 188 [Score 19.145752]   GBROWSE*******************

 CG12899 in-situ | CG12899 | - | -17153 | -17437 | UPSTREAM | CG12899-RA | "-"
 CG12898 in-situ | CG12898 | - | 1878 | 1408 | DOWNSTREAM | CG12898-RA | "-"


*********************** Rank 189 [Score 19.125366]   GBROWSE*******************

 CG32431 in-situ | CG32431 | + | -6870 | -6163 | DOWNSTREAM | CG32431-RA | "-"
insitu highlight CG4717 in-situ | kni | - | 751 | -2282 | INTRAGENIC | intron:CG4717-RA:1 | CG4717-RA | "-"


*********************** Rank 190 [Score 19.101501]   GBROWSE*******************

 CG10093 in-situ | Cyp313a3 | - | -8500 | -10330 | UPSTREAM | CG10093-RA | "-"
 CG3942 in-situ | CG3942 | + | 6578 | 8983 | UPSTREAM | CG3942-RA | "-"

*********************** Rank 191 [Score 19.095154]   GBROWSE*******************

 CG15498 in-situ | CG15498 | - | -2315 | -3945 | UPSTREAM | CG15498-RA | "-"
 CG18402 in-situ | InR | - | 9065 | 1035 | DOWNSTREAM | CG18402-RA | "-"

*********************** Rank 192 [Score 19.083008]   GBROWSE*******************

 CG9138 in-situ | SP1070 | - | -25406 | -36889 | UPSTREAM | CG9138-RA | "-"
 CG13776 in-situ | CG13776 | + | 3077 | 4226 | UPSTREAM | CG13776-RA | "-"


*********************** Rank 193 [Score 19.072754]   GBROWSE*******************

 CG14363 in-situ | CG14363 | - | -20530 | -23507 | UPSTREAM | CG14363-RA | "-"
 CG17956 in-situ | Mst87F | - | 16201 | 15636 | DOWNSTREAM | CG17956-RA | "-"

*********************** Rank 194 [Score 19.062378]   GBROWSE*******************

 CG32655 in-situ | CG32655 | - | -60911 | -61969 | UPSTREAM | CG32655-RA | "-"
 CG2577 in-situ | CG2577 | + | 76571 | 77811 | UPSTREAM | CG2577-RA | "-"

*********************** Rank 195 [Score 19.048340]   GBROWSE*******************

 CG14532 in-situ | CG14532 | - | -13284 | -13877 | UPSTREAM | CG14532-RA | "-"
 CG7233 in-situ | CG7233 | - | 62871 | 61855 | DOWNSTREAM | CG7233-RA | "-"

*********************** Rank 196 [Score 19.037659]   GBROWSE*******************

insitu CG14334 in-situ | beat-IIa | - | -9996 | -56114 | UPSTREAM | CG14334-RA | "-"
 CG14333 in-situ | CG14333 | + | 8539 | 8847 | UPSTREAM | CG14333-RA | "-"

*********************** Rank 197 [Score 18.998962]   GBROWSE*******************

insitu CG6847 in-situ | CG6847 | + | -39678 | -26975 | DOWNSTREAM | CG6847-RA | "-"
 CG32494 in-situ | CG32494 | + | 48850 | 50087 | UPSTREAM | CG32494-RA | "-"


*********************** Rank 198 [Score 18.998779]   GBROWSE*******************

 CG14506 in-situ | CG14506 | - | -14833 | -16243 | UPSTREAM | CG14506-RA | "-"
 CG11958 in-situ | Cnx99A | - | 7582 | 3189 | DOWNSTREAM | CG11958-RA | "-" | CG11958-RB | "-"

*********************** Rank 199 [Score 18.990112]   GBROWSE*******************

 CG13712 in-situ | CG13712 | - | -13853 | -14206 | UPSTREAM | CG13712-RA | "-"
 CG12493 in-situ | CG12493 | - | 30009 | 28684 | DOWNSTREAM | CG12493-RA | "-"

*********************** Rank 200 [Score 18.984131]   GBROWSE*******************

 CG14985 in-situ | CG14985 | + | -13799 | -10645 | DOWNSTREAM | CG14985-RA | "-"
insitu highlight CG1132 in-situ | fd64A | + | 1823 | 3908 | UPSTREAM | CG1132-RA | "-"


*********************** Rank 201 [Score 18.982666]   GBROWSE*******************

 CG13826 in-situ | CG13826 | - | -1063 | -5607 | UPSTREAM | CG13826-RA | "-"
 CG4467 in-situ | CG4467 | - | 12278 | 2290 | DOWNSTREAM | CG4467-RA | "-"

*********************** Rank 202 [Score 18.964661]   GBROWSE*******************

insitu CG12094 in-situ | CG12094 | + | -58209 | -6027 | DOWNSTREAM | CG12094-RA | "-"
 CG12102 in-situ | CG12102 | + | 1444 | 2630 | UPSTREAM | CG12102-RA | "-"

*********************** Rank 203 [Score 18.964661]   GBROWSE*******************

 CG15014 in-situ | CG15014 | - | -13044 | -14169 | UPSTREAM | CG15014-RA | "-"
 CG15015 in-situ | Cip4 | - | 27913 | -11740 | INTRAGENIC | intron:CG15015-RA:4 | CG15015-RA | "-"

*********************** Rank 204 [Score 18.964172]   GBROWSE*******************

insitu highlight CG10917 in-situ | fj | + | -42003 | -38452 | DOWNSTREAM | CG10917-RA | "-"
 CG5581 in-situ | Ote | - | 2427 | 837 | DOWNSTREAM | CG5581-RA | "-"

*********************** Rank 205 [Score 18.916016]   GBROWSE*******************

 CG18371 in-situ | CG18371 | + | -32568 | -32080 | DOWNSTREAM | CG18371-RA | "-"
 CG13353 in-situ | CG13353 | + | 29190 | 30541 | UPSTREAM | CG13353-RA | "-"

*********************** Rank 206 [Score 18.874268]   GBROWSE*******************

 CG4841 in-situ | CG4841 | + | -13288 | -6914 | DOWNSTREAM | CG4841-RA | "-"
 CG33179 in-situ | beat-IIIb | + | 60689 | 69076 | UPSTREAM | CG33179-RA | "-"

*********************** Rank 207 [Score 18.860718]   GBROWSE*******************

insitu highlight CG6716 in-situ | prd | - | -2573 | -6032 | UPSTREAM | CG6716-RB | "-" | CG6716-RA | "-"
 CG5325 in-situ | CG5325 | + | 4742 | 6223 | UPSTREAM | CG5325-RA | "-" | CG5325-RB | "-"

note: overlaps known module prd_Pstripe by 464 bases (module coords: 12077663-12078363)
note: overlaps known module prd_rescue by 500 bases (module coords: 12066868-12085327)

*********************** Rank 208 [Score 18.853027]   GBROWSE*******************

 CG2595 in-situ | RacGAP84C | + | -12526 | -9827 | DOWNSTREAM | CG2595-RA | "-" | CG2595-RB | "-"
 CG32467 in-situ | CG32467 | - | 4713 | 4258 | DOWNSTREAM | CG32467-RA | "-"

*********************** Rank 209 [Score 18.838501]   GBROWSE*******************

 CG5462 in-situ | scrib | + | -24217 | 35627 | INTRAGENIC | intron:CG5462-RA:5 | intron:CG5462-RB:5 | intron:CG5462-RC:5 | intron:CG5462-RD:5 | CG5462-RA | "-" | CG5462-RB | "-" | CG5462-RC | "-" | CG5462-RD | "-"
 CG31082 in-situ | CG31082 | + | 23797 | 26238 | UPSTREAM | CG31082-RA | "-"


*********************** Rank 210 [Score 18.825134]   GBROWSE*******************

 CG31217 in-situ | CG31217 | - | -4243 | -7564 | UPSTREAM | CG31217-RA | "-"
 CG31498 in-situ | CG31498 | + | 4895 | 5728 | UPSTREAM | CG31498-RA | "-"

*********************** Rank 211 [Score 18.819702]   GBROWSE*******************

insitu highlight CG7952 in-situ | gt | - | -5315 | -7171 | UPSTREAM | CG7952-RB | "-"
 CG7925 in-situ | tko | - | 9698 | 8029 | DOWNSTREAM | CG7925-RB | "-"

*********************** Rank 212 [Score 18.811157]   GBROWSE*******************

 CG31749 in-situ | CG31749 | + | -50203 | -49413 | DOWNSTREAM | CG31749-RA | "-"
 CG10305 in-situ | RpS26 | - | 6720 | 5981 | DOWNSTREAM | CG10305-RA | "-" | CG10305-RB | "-" | CG10305-RC | "-"

*********************** Rank 213 [Score 18.810974]   GBROWSE*******************

insitu CG11453 in-situ | CG11453 | + | -5738 | -3731 | DOWNSTREAM | CG11453-RA | "-"
insitu CG4608 in-situ | bnl | - | 47556 | 4812 | DOWNSTREAM | CG4608-RA | "-" | CG4608-RB | "-"

*********************** Rank 214 [Score 18.802002]   GBROWSE*******************

 CG17839 in-situ | CG17839 | + | -74518 | 1963 | INTRAGENIC | intron:CG17839-RA:10 | CG17839-RA | "-"
 CG13467 in-situ | CG13467 | + | 24343 | 25366 | UPSTREAM | CG13467-RA | "-"

*********************** Rank 215 [Score 18.799805]   GBROWSE*******************

 CG1088 in-situ | Vha26 | + | -7200 | -4224 | DOWNSTREAM | CG1088-RB | "-" | CG1088-RA | "-"
 CG2922 in-situ | eIF-5C | - | 2238 | -3567 | INTRAGENIC | intron:CG2922-RA:3 | intron:CG2922-RG:3 | intron:CG2922-RC:4 | intron:CG2922-RF:3 | intron:CG2922-RD:3 | intron:CG2922-RB:4 | intron:CG2922-RE:2 | CG2922-RA | "-" | CG2922-RG | "-" | CG2922-RC | "-" | CG2922-RF | "-" | CG2922-RD | "-" | CG2922-RB | "-" | CG2922-RE | "-"


*********************** Rank 216 [Score 18.789917]   GBROWSE*******************

insitu highlight CG6464 in-situ | salm | - | -9826 | -21118 | UPSTREAM | CG6464-RA | "-"
insitu highlight CG4922 in-situ | sala | + | 30760 | 31526 | UPSTREAM | CG4922-RA | "-"

note: overlaps known module salm_anterior by 278 bases (module coords: 11444511-11444788)
note: overlaps known module salm_early_enhancer by 292 bases (module coords: 11444508-11445023)
note: overlaps known module salm_posterior by 12 bases (module coords: 11444788-11445026)

*********************** Rank 217 [Score 18.787109]   GBROWSE*******************

 CG1395 in-situ | stg | - | -32832 | -36794 | UPSTREAM | CG1395-RA | "-"
 CG14506 in-situ | CG14506 | - | 8617 | 7207 | DOWNSTREAM | CG14506-RA | "-"

*********************** Rank 218 [Score 18.778625]   GBROWSE*******************

 CG12623 in-situ | CG12623 | - | -3936 | -4646 | UPSTREAM | CG12623-RA | "-"
 CG1641 in-situ | sisA | - | 7235 | 6468 | DOWNSTREAM | CG1641-RA | "-"


*********************** Rank 219 [Score 18.776917]   GBROWSE*******************

 CG6890 in-situ | Tollo | + | -35030 | -27839 | DOWNSTREAM | CG6890-RA | "-"
 CG7259 in-situ | Best4 | + | 28630 | 30221 | UPSTREAM | CG7259-RA | "-"

*********************** Rank 220 [Score 18.774536]   GBROWSE*******************

 CG4841 in-situ | CG4841 | + | -54438 | -48064 | DOWNSTREAM | CG4841-RA | "-"
 CG33179 in-situ | beat-IIIb | + | 19539 | 27926 | UPSTREAM | CG33179-RA | "-"

*********************** Rank 221 [Score 18.760376]   GBROWSE*******************

 CG13785 in-situ | CG13785 | + | -17686 | -16789 | DOWNSTREAM | CG13785-RA | "-"
insitu highlight CG4889 in-situ | wg | + | 6942 | 16036 | UPSTREAM | CG4889-RA | "-" | CG4889-RB | "-"

*********************** Rank 222 [Score 18.753418]   GBROWSE*******************

 CG15159 in-situ | CG15159 | - | -9702 | -10571 | UPSTREAM | CG15159-RA | "-"
 CG10231 in-situ | CG10231 | - | 9355 | -21089 | INTRAGENIC | intron:CG10231-RA:1 | CG10231-RA | "-"

*********************** Rank 223 [Score 18.750916]   GBROWSE*******************

 CG4021 in-situ | CG4021 | + | -3054 | -1574 | DOWNSTREAM | CG4021-RA | "-"
 CG4402 in-situ | lox2 | - | 15256 | 13217 | DOWNSTREAM | CG4402-RA | "-"

*********************** Rank 224 [Score 18.709351]   GBROWSE*******************

 CG15596 in-situ | CG15596 | - | -8840 | -9748 | UPSTREAM | CG15596-RA | "-"
 CG1154 in-situ | CG1154 | + | 1003 | 4967 | UPSTREAM | CG1154-RA | "-"

*********************** Rank 225 [Score 18.694824]   GBROWSE*******************

insitu CG9184 in-situ | CG9184 | - | -2969 | -3989 | UPSTREAM | CG9184-RA | "-" | CG9184-RB | "-"
 CG9160 in-situ | mtacp1 | + | 4035 | 5862 | UPSTREAM | CG9160-RA | "-" | CG9160-RB | "-"


*********************** Rank 226 [Score 18.678345]   GBROWSE*******************

 CG31438 in-situ | CG31438 | + | -12287 | -11554 | DOWNSTREAM | CG31438-RA | "-"
 CG6570 in-situ | lbl | - | 12709 | -11437 | INTRAGENIC | intron:CG6570-RA:1 | CG6570-RA | "-"

*********************** Rank 227 [Score 18.657349]   GBROWSE*******************

 CG17738 in-situ | CG17738 | + | -21739 | -21407 | DOWNSTREAM | CG17738-RA | "-"
 CG4066 in-situ | CG4066 | + | 18694 | 20460 | UPSTREAM | CG4066-RA | "-"

*********************** Rank 228 [Score 18.628662]   GBROWSE*******************

insitu highlight CG10052 in-situ | Rx | + | -5126 | 15126 | INTRAGENIC | intron:CG10052-RA:2 | CG10052-RA | "-"
insitu CG10067 in-situ | Act57B | + | 22122 | 24534 | UPSTREAM | CG10067-RA | "-"


*********************** Rank 229 [Score 18.619629]   GBROWSE*******************

 CG1428 in-situ | CG1428 | + | -7085 | -5042 | DOWNSTREAM | CG1428-RA | "-"
 CG31600 in-situ | CG31600 | - | 1928 | 1066 | DOWNSTREAM | CG31600-RA | "-"

*********************** Rank 230 [Score 18.609375]   GBROWSE*******************

insitu highlight CG10325 in-situ | abd-A | - | -34912 | -57338 | UPSTREAM | CG10325-RA | "-" | CG10325-RB | "-"
 CG10349 in-situ | CG10349 | + | 14765 | 20147 | UPSTREAM | CG10349-RA | "-" | CG10349-RB | "-"

*********************** Rank 231 [Score 18.593750]   GBROWSE*******************

 CG5406 in-situ | sif | + | -33972 | 46247 | INTRAGENIC | intron:CG5406-RB:12 | intron:CG5406-RA:2 | CG5406-RB | "-" | CG5406-RA | "-"
 CG32414 in-situ | CG32414 | + | 38833 | 40256 | UPSTREAM | CG32414-RA | "-"


*********************** Rank 232 [Score 18.587341]   GBROWSE*******************

 CG30358 in-situ | CG30358 | + | -13256 | -12563 | DOWNSTREAM | CG30358-RA | "-"
 CG14755 in-situ | CG14755 | + | 14436 | 15290 | UPSTREAM | CG14755-RA | "-"

*********************** Rank 233 [Score 18.571533]   GBROWSE*******************

 CG9266 in-situ | CG9266 | - | -67215 | -70639 | UPSTREAM | CG9266-RB | "-"
insitu CG1762 in-situ | betaInt-nu | + | 16676 | 21888 | UPSTREAM | CG1762-RA | "-"

*********************** Rank 234 [Score 18.567261]   GBROWSE*******************

 CG31066 in-situ | CG31066 | + | -3896 | -3275 | DOWNSTREAM | CG31066-RA | "-"
 CG6127 in-situ | Ser | - | 4331 | -17567 | INTRAGENIC | intron:CG6127-RA:2 | CG6127-RA | "-"

*********************** Rank 235 [Score 18.560547]   GBROWSE*******************

 CG6380 in-situ | CG6380 | - | -70810 | -71847 | UPSTREAM | CG6380-RA | "-"
 CG31804 in-situ | CG31804 | + | 478 | 1218 | UPSTREAM | CG31804-RA | "-"

*********************** Rank 236 [Score 18.537354]   GBROWSE*******************

 CG7596 in-situ | Sgs5 | + | -2173 | -1519 | DOWNSTREAM | CG7596-RA | "-"
 CG7623 in-situ | sll | + | 8539 | 10981 | UPSTREAM | CG7623-RA | "-"


*********************** Rank 237 [Score 18.487915]   GBROWSE*******************

 CG14560 in-situ | msopa | + | -59864 | -59369 | DOWNSTREAM | CG14560-RA | "-"
 CG15374 in-situ | CG15374 | + | 22755 | 23249 | UPSTREAM | CG15374-RA | "-"

*********************** Rank 238 [Score 18.476624]   GBROWSE*******************

 CG30428 in-situ | CG30428 | + | 20221734 | 20222897 | UPSTREAM | CG30428-RA | "-"
 CG2981 in-situ | TpnC41C | - | 133834 | 130011 | DOWNSTREAM | CG2981-RA | "-"


*********************** Rank 239 [Score 18.465942]   GBROWSE*******************

 CG14061 in-situ | CG14061 | - | -10328 | -11428 | UPSTREAM | CG14061-RA | "-"
 CG12558 in-situ | CG12558 | + | 21960 | 22899 | UPSTREAM | CG12558-RA | "-"


*********************** Rank 240 [Score 18.456299]   GBROWSE*******************

 CG14698 in-situ | CG14698 | + | -8765 | -8239 | DOWNSTREAM | CG14698-RA | "-"
 CG4695 in-situ | CG4695 | + | 712 | 4940 | UPSTREAM | CG4695-RA | "-"

*********************** Rank 241 [Score 18.455933]   GBROWSE*******************

 CG14111 in-situ | CG14111 | + | -1940 | -852 | DOWNSTREAM | CG14111-RA | "-"
insitu CG14107 in-situ | CG14107 | - | 111 | -878 | INTRAGENIC | intron:CG14107-RA:1 | CG14107-RA | "-"


*********************** Rank 242 [Score 18.451050]   GBROWSE*******************

 CG8472 in-situ | Cam | + | -9579 | 5638 | INTRAGENIC | intron:CG8472-RA:3 | intron:CG8472-RB:2 | CG8472-RA | "-" | CG8472-RB | "-"
 CG13165 in-situ | CG13165 | + | 24430 | 28726 | UPSTREAM | CG13165-RA | "-"

*********************** Rank 243 [Score 18.450806]   GBROWSE*******************

 CG17952 in-situ | CG17952 | + | -27602 | -24040 | DOWNSTREAM | CG17952-RB | "-" | CG17952-RA | "-" | CG17952-RC | "-"
 CG7975 in-situ | Grx-1 | + | 9328 | 9879 | UPSTREAM | CG7975-RA | "-"

*********************** Rank 244 [Score 18.429077]   GBROWSE*******************

 CG7313 in-situ | CG7313 | + | -17774 | -17025 | DOWNSTREAM | CG7313-RA | "-"
 CG5103 in-situ | CG5103 | - | 24496 | 22246 | DOWNSTREAM | CG5103-RA | "-"

*********************** Rank 245 [Score 18.421875]   GBROWSE*******************

 CG15483 in-situ | CG15483 | - | -36340 | -37724 | UPSTREAM | CG15483-RA | "-"
 CG12283 in-situ | kek1 | - | 24607 | 20750 | DOWNSTREAM | CG12283-RA | "-"

*********************** Rank 246 [Score 18.403381]   GBROWSE*******************

 CG17134 in-situ | CG17134 | + | -3431 | -2173 | DOWNSTREAM | CG17134-RA | "-"
insitu CG6713 in-situ | Nos | - | 13369 | -19872 | INTRAGENIC | intron:CG6713-RA:5 | CG6713-RA | "-"

*********************** Rank 247 [Score 18.393555]   GBROWSE*******************

insitu CG10067 in-situ | Act57B | + | -13078 | -10666 | DOWNSTREAM | CG10067-RA | "-"
 CG33152 in-situ | hbn | - | 3803 | -2442 | INTRAGENIC | intron:CG33152-RA:3 | CG33152-RA | "-"


*********************** Rank 248 [Score 18.380615]   GBROWSE*******************

 CG6486 in-situ | CG6486 | + | -20466 | -19274 | DOWNSTREAM | CG6486-RA | "-"
insitu highlight CG6494 in-situ | h | + | 5969 | 9249 | UPSTREAM | CG6494-RA | "-"


note: overlaps known module h_rescue by 500 bases (module coords: 8620516-8642090)
note: overlaps known module h_stripe2_1991 by 500 bases (module coords: 8625021-8630510)
note: overlaps known module h_stripe5_1990a by 500 bases (module coords: 8627668-8630638)
note: overlaps known module h_stripe5_1990b by 500 bases (module coords: 8627680-8629353)
note: overlaps known module h_stripe1_5_1991 by 500 bases (module coords: 8627680-8630510)
note: overlaps known module h_stripe5_1993 by 410 bases (module coords: 8628790-8629353)

*********************** Rank 249 [Score 18.340820]   GBROWSE*******************

 CG31394 in-situ | CG31394 | - | -49654 | -50392 | UPSTREAM | CG31394-RA | "-"
insitu highlight CG17117 in-situ | hth | - | 59854 | -68970 | INTRAGENIC | intron:CG17117-RB:6 | intron:CG17117-RC:6 | intron:CG17117-RA:5 | CG17117-RD | "-" | CG17117-RB | "-" | CG17117-RC | "-" | CG17117-RA | "-"


*********************** Rank 250 [Score 18.336060]   GBROWSE*******************

insitu highlight CG7952 in-situ | gt | - | -2165 | -4021 | UPSTREAM | CG7952-RB | "-"
 CG7925 in-situ | tko | - | 12848 | 11179 | DOWNSTREAM | CG7925-RB | "-"


note: overlaps known module gt_posterior by 484 bases (module coords: 2187439-2188383)

*********************** Rank 251 [Score 18.316650]   GBROWSE*******************

 CG12358 in-situ | Paip2 | + | -4313 | -736 | DOWNSTREAM | CG12358-RA | "-"
 CG31342 in-situ | CG31342 | + | 705 | 6184 | UPSTREAM | CG31342-RA | "-"

*********************** Rank 252 [Score 18.315918]   GBROWSE*******************

 CG16850 in-situ | CG16850 | + | -9777 | -7977 | DOWNSTREAM | CG16850-RA | "-"
 CG31847 in-situ | CG31847 | + | 11086 | 15425 | UPSTREAM | CG31847-RA | "-"

*********************** Rank 253 [Score 18.313843]   GBROWSE*******************

 CG11071 in-situ | CG11071 | - | -6917 | -9942 | UPSTREAM | CG11071-RA | "-"
 CG32611 in-situ | CG32611 | - | 21471 | 5341 | DOWNSTREAM | CG32611-RB | "-"

*********************** Rank 254 [Score 18.310425]   GBROWSE*******************

 CG10037 in-situ | vvl | + | -95861 | -91485 | DOWNSTREAM | CG10037-RA | "-"
insitu CG10078 in-situ | Prat2 | - | 28837 | 25972 | DOWNSTREAM | CG10078-RB | "-" | CG10078-RA | "-"

*********************** Rank 255 [Score 18.295410]   GBROWSE*******************

insitu CG17325 in-situ | CG17325 | + | -4604 | -3676 | DOWNSTREAM | CG17325-RA | "-"
 CG17324 in-situ | BEST:GH06505 | + | 1727 | 4956 | UPSTREAM | CG17324-RA | "-"


*********************** Rank 256 [Score 18.277222]   GBROWSE*******************

 CG15452 in-situ | CG15452 | - | -27136 | -28014 | UPSTREAM | CG15452-RA | "-"
 CG32507 in-situ | CG32507 | - | 8068 | 7487 | DOWNSTREAM | CG32507-RA | "-"


*********************** Rank 257 [Score 18.267700]   GBROWSE*******************

 CG13344 in-situ | CG13344 | + | -3594 | -1528 | DOWNSTREAM | CG13344-RA | "-"
 CG18076 in-situ | shot | - | 76424 | -1461 | INTRAGENIC | intron:CG18076-RB:30 | intron:CG18076-RG:30 | intron:CG18076-RE:26 | intron:CG18076-RA:30 | intron:CG18076-RC:23 | intron:CG18076-RH:27 | CG18076-RB | "-" | CG18076-RG | "-" | CG18076-RE | "-" | CG18076-RA | "-" | CG18076-RC | "-" | CG18076-RH | "-"

*********************** Rank 258 [Score 18.259644]   GBROWSE*******************

insitu highlight CG4807 in-situ | ab | + | -37960 | 8902 | INTRAGENIC | intron:CG4807-RB:7 | intron:CG4807-RA:7 | CG4807-RB | "-" | CG4807-RA | "-"
 CG32830 in-situ | CG32830 | + | 2395 | 4398 | UPSTREAM | CG32830-RA | "-"

*********************** Rank 259 [Score 18.258179]   GBROWSE*******************

insitu CG12701 in-situ | CG12701 | - | -19062 | -26003 | UPSTREAM | CG12701-RB | "-" | CG12701-RA | "-"
 CG12700 in-situ | skpD | + | 10739 | 11398 | UPSTREAM | CG12700-RA | "-"


*********************** Rank 260 [Score 18.244690]   GBROWSE*******************

insitu CG8740 in-situ | BcDNA:GH05582 | + | -53134 | -29363 | DOWNSTREAM | CG8740-RB | "-" | CG8740-RC | "-" | CG8740-RA | "-"
 CG8746 in-situ | CG8746 | + | 3680 | 4824 | UPSTREAM | CG8746-RA | "-"

*********************** Rank 261 [Score 18.238770]   GBROWSE*******************

insitu CG7891 in-situ | CG7891 | + | -33999 | -31767 | DOWNSTREAM | CG7891-RA | "-"
 CG9656 in-situ | grn | - | 20829 | -13263 | INTRAGENIC | intron:CG9656-RA:5 | CG9656-RA | "-"


*********************** Rank 262 [Score 18.220703]   GBROWSE*******************

 CG11368 in-situ | CG11368 | + | -34765 | -34310 | DOWNSTREAM | CG11368-RA | "-"
 CG32719 in-situ | CG32719 | - | 25870 | 21918 | DOWNSTREAM | CG32719-RA | "-"


*********************** Rank 263 [Score 18.220703]   GBROWSE*******************

 CG9078 in-situ | ifc | + | -3861 | -1327 | DOWNSTREAM | CG9078-RA | "-"
insitu CG9088 in-situ | lid | - | 9298 | 256 | DOWNSTREAM | CG9088-RA | "-" | CG9088-RB | "-"

*********************** Rank 264 [Score 18.214600]   GBROWSE*******************

insitu highlight CG10325 in-situ | abd-A | - | -16262 | -38688 | UPSTREAM | CG10325-RA | "-" | CG10325-RB | "-"
 CG10349 in-situ | CG10349 | + | 33415 | 38797 | UPSTREAM | CG10349-RA | "-" | CG10349-RB | "-"

*********************** Rank 265 [Score 18.206543]   GBROWSE*******************

insitu CG6883 in-situ | trh | - | -48504 | -59297 | UPSTREAM | CG6883-RA | "-"
 CG13891 in-situ | CG13891 | - | 8070 | 7409 | DOWNSTREAM | CG13891-RA | "-"

*********************** Rank 266 [Score 18.201660]   GBROWSE*******************

 CG7520 in-situ | CG7520 | + | -20215 | -9344 | DOWNSTREAM | CG7520-RA | "-"
 CG7515 in-situ | CG7515 | + | 2730 | 4313 | UPSTREAM | CG7515-RA | "-"


*********************** Rank 267 [Score 18.200623]   GBROWSE*******************

 CG31394 in-situ | CG31394 | - | -25154 | -25892 | UPSTREAM | CG31394-RA | "-"
insitu highlight CG17117 in-situ | hth | - | 84354 | -44470 | INTRAGENIC | intron:CG17117-RB:7 | intron:CG17117-RC:7 | intron:CG17117-RA:6 | CG17117-RD | "-" | CG17117-RB | "-" | CG17117-RC | "-" | CG17117-RA | "-"

*********************** Rank 268 [Score 18.149658]   GBROWSE*******************

 CG31909 in-situ | CG31909 | + | -15048 | -14504 | DOWNSTREAM | CG31909-RA | "-"
insitu CG4698 in-situ | Wnt4 | - | 4549 | -17159 | INTRAGENIC | intron:CG4698-RA:1 | CG4698-RA | "-"

*********************** Rank 269 [Score 18.121704]   GBROWSE*******************

 CG4162 in-situ | lace | + | -27406 | -21918 | DOWNSTREAM | CG4162-RA | "-"
 CG15256 in-situ | BG:DS04862.2 | + | 23956 | 36734 | UPSTREAM | CG15256-RA | "-"


*********************** Rank 270 [Score 18.105469]   GBROWSE*******************

 CG12944 in-situ | Obp47a | + | -13348 | -12786 | DOWNSTREAM | CG12944-RA | "-"
 CG7722 in-situ | CG7722 | - | 18395 | 16914 | DOWNSTREAM | CG7722-RA | "-"

*********************** Rank 271 [Score 18.083252]   GBROWSE*******************

insitu CG2083 in-situ | CG2083 | - | -46341 | -58349 | UPSTREAM | CG2083-RA | "-"
 CG14952 in-situ | CG14952 | - | 1424 | 1041 | DOWNSTREAM | CG14952-RA | "-"

*********************** Rank 272 [Score 18.073914]   GBROWSE*******************

 CG13056 in-situ | CG13056 | + | -4681 | -4469 | DOWNSTREAM | CG13056-RA | "-"
 CG4998 in-situ | CG4998 | + | 828 | 7077 | UPSTREAM | CG4998-RA | "-"


*********************** Rank 273 [Score 18.051636]   GBROWSE*******************

 CG12425 in-situ | CG12425 | + | -12788 | -11233 | DOWNSTREAM | CG12425-RA | "-"
 CG4787 in-situ | CG4787 | + | 75487 | 77773 | UPSTREAM | CG4787-RA | "-"

*********************** Rank 274 [Score 18.038391]   GBROWSE*******************

insitu CG5799 in-situ | dve | + | -23886 | 18569 | INTRAGENIC | intron:CG5799-RA:2 | intron:CG5799-RD:2 | intron:CG5799-RC:2 | CG5799-RA | "-" | CG5799-RD | "-" | CG5799-RB | "-" | CG5799-RC | "-"
insitu CG5819 in-situ | CG5819 | + | 25610 | 29109 | UPSTREAM | CG5819-RA | "-" | CG5819-RB | "-"

*********************** Rank 275 [Score 18.032349]   GBROWSE*******************

 CG8213 in-situ | CG8213 | - | -5195 | -21417 | UPSTREAM | CG8213-RA | "-"
 CG11824 in-situ | CG11824 | - | 3722 | 2789 | DOWNSTREAM | CG11824-RA | "-"


*********************** Rank 276 [Score 18.023682]   GBROWSE*******************

 CG10029 in-situ | CG10029 | - | -8640 | -9976 | UPSTREAM | CG10029-RA | "-"
 CG31496 in-situ | CG31496 | + | 19404 | 20469 | UPSTREAM | CG31496-RA | "-"

*********************** Rank 277 [Score 18.008606]   GBROWSE*******************

 CG4090 in-situ | CG4090 | - | -15465 | -21893 | UPSTREAM | CG4090-RA | "-"
 CG31262 in-situ | CG31262 | - | 44019 | 41926 | DOWNSTREAM | CG31262-RA | "-"


*********************** Rank 278 [Score 18.007324]   GBROWSE*******************

insitu CG5973 in-situ | CG5973 | + | -4713 | 3050 | INTRAGENIC | intron:CG5973-RA:1 | intron:CG5973-RB:1 | intron:CG5973-RC:1 | CG5973-RA | "-" | CG5973-RB | "-" | CG5973-RC | "-"
 CG12789 in-situ | BEST:CK01577 | - | 8946 | 3126 | DOWNSTREAM | CG12789-RA | "-" | CG12789-RB | "-"

*********************** Rank 279 [Score 17.997070]   GBROWSE*******************

 CG2267 in-situ | CG2267 | - | -13234 | -15022 | UPSTREAM | CG2267-RA | "-"
 CG31013 in-situ | CG31013 | - | 3571 | 1619 | DOWNSTREAM | CG31013-RA | "-"

*********************** Rank 280 [Score 17.994324]   GBROWSE*******************

 CG13344 in-situ | CG13344 | + | -2094 | -28 | DOWNSTREAM | CG13344-RA | "-"
 CG18076 in-situ | shot | - | 77924 | 39 | DOWNSTREAM | CG18076-RB | "-" | CG18076-RG | "-" | CG18076-RE | "-" | CG18076-RA | "-" | CG18076-RC | "-" | CG18076-RH | "-"

*********************** Rank 281 [Score 17.991455]   GBROWSE*******************

 CG13417 in-situ | Gr93a | + | -2911 | -1401 | DOWNSTREAM | CG13417-RA | "-"
 CG13416 in-situ | CG13416 | + | 5491 | 6496 | UPSTREAM | CG13416-RA | "-"

*********************** Rank 282 [Score 17.989868]   GBROWSE*******************

 CG12377 in-situ | CG12377 | - | -30989 | -33228 | UPSTREAM | CG12377-RA | "-"
 CG11404 in-situ | CG11404 | + | 12215 | 13085 | UPSTREAM | CG11404-RA | "-"

*********************** Rank 283 [Score 17.988464]   GBROWSE*******************

 CG15198 in-situ | CG15198 | + | -48852 | -48484 | DOWNSTREAM | CG15198-RA | "-"
 CG12624 in-situ | CG12624 | - | 12547 | 11272 | DOWNSTREAM | CG12624-RA | "-"


*********************** Rank 284 [Score 17.987183]   GBROWSE*******************

 CG32083 in-situ | CG32083 | - | -1341 | -2002 | UPSTREAM | CG32083-RA | "-"
 CG6175 in-situ | CG6175 | - | 12619 | 5754 | DOWNSTREAM | CG6175-RB | "-"

*********************** Rank 285 [Score 17.977112]   GBROWSE*******************

 CG32632 in-situ | CG32632 | + | -17661 | -4500 | DOWNSTREAM | CG32632-RB | "-"
insitu CG7107 in-situ | up | - | 7412 | -1522 | INTRAGENIC | intron:CG7107-RA:7 | intron:CG7107-RB:6 | intron:CG7107-RD:4 | CG7107-RA | "-" | CG7107-RB | "-" | CG7107-RD | "-"


*********************** Rank 286 [Score 17.954102]   GBROWSE*******************

insitu highlight CG1133 in-situ | opa | + | -1234 | 15908 | INTRAGENIC | intron:CG1133-RA:1 | CG1133-RA | "-"
 CG14660 in-situ | CG14660 | - | 24454 | 21926 | DOWNSTREAM | CG14660-RA | "-"

*********************** Rank 287 [Score 17.932983]   GBROWSE*******************

 CG31190 in-situ | CG31190 | - | -10146 | -40948 | UPSTREAM | CG31190-RA | "-"
 CG5823 in-situ | CG5823 | - | 18762 | 16506 | DOWNSTREAM | CG5823-RA | "-"

*********************** Rank 288 [Score 17.932861]   GBROWSE*******************

insitu CG5799 in-situ | dve | + | -4536 | 37919 | INTRAGENIC | intron:CG5799-RA:1 | intron:CG5799-RD:1 | intron:CG5799-RB:1 | intron:CG5799-RC:1 | CG5799-RA | "-" | CG5799-RD | "-" | CG5799-RB | "-" | CG5799-RC | "-"
insitu CG5819 in-situ | CG5819 | + | 44960 | 48459 | UPSTREAM | CG5819-RA | "-" | CG5819-RB | "-"

*********************** Rank 289 [Score 17.932556]   GBROWSE*******************

 CG8398 in-situ | CG8398 | + | -12696 | -3836 | DOWNSTREAM | CG8398-RA | "-" | CG8398-RB | "-" | CG8398-RC | "-"
insitu highlight CG8442 in-situ | Glu-RI | + | 2013 | 13045 | UPSTREAM | CG8442-RA | "-"

*********************** Rank 290 [Score 17.929321]   GBROWSE*******************

 CG9650 in-situ | CG9650 | + | -3598 | 43747 | INTRAGENIC | intron:CG9650-RA:1 | CG9650-RA | "-" | CG9650-RB | "-" | CG9650-RC | "-"
 CG32725 in-situ | CG32725 | - | 41488 | 40761 | DOWNSTREAM | CG32725-RA | "-"

*********************** Rank 291 [Score 17.918335]   GBROWSE*******************

 CG31638 in-situ | CG31638 | + | -1362 | 3636 | INTRAGENIC | intron:CG31638-RA:5 | CG31638-RA | "-"
 CG9547 in-situ | CG9547 | - | 2043 | 488 | DOWNSTREAM | CG9547-RA | "-"

*********************** Rank 292 [Score 17.917480]   GBROWSE*******************

 CG32773 in-situ | CG32773 | - | -14833 | -15252 | UPSTREAM | CG32773-RA | "-"
 CG3578 in-situ | bi | + | 9772 | 81380 | UPSTREAM | CG3578-RA | "-"

*********************** Rank 293 [Score 17.916748]   GBROWSE*******************

insitu highlight CG4345 in-situ | grim | - | -91407 | -93102 | UPSTREAM | CG4345-RA | "-"
 CG4319 in-situ | rpr | - | 2582 | 1732 | DOWNSTREAM | CG4319-RA | "-"


*********************** Rank 294 [Score 17.872620]   GBROWSE*******************

insitu CG6051 in-situ | CG6051 | - | -18020 | -22461 | UPSTREAM | CG6051-RA | "-"
 CG5889 in-situ | Mdh | + | 4428 | 10393 | UPSTREAM | CG5889-RA | "-"

*********************** Rank 295 [Score 17.872437]   GBROWSE*******************

 CG32030 in-situ | CG32030 | + | -11896 | 20385 | INTRAGENIC | intron:CG32030-RA:2 | intron:CG32030-RB:2 | CG32030-RA | "-" | CG32030-RB | "-"
insitu CG5804 in-situ | CG5804 | - | 10200 | 9802 | DOWNSTREAM | CG5804-RA | "-"

*********************** Rank 296 [Score 17.868225]   GBROWSE*******************

 CG14477 in-situ | mm | + | -17131 | 7644 | INTRAGENIC | intron:CG14477-RA:4 | CG14477-RA | "-"
 CG10939 in-situ | Sip1 | + | 26371 | 36948 | UPSTREAM | CG10939-RA | "-"

*********************** Rank 297 [Score 17.866455]   GBROWSE*******************

 CG15696 in-situ | CG15696 | + | -1800 | -1261 | DOWNSTREAM | CG15696-RA | "-"
 CG15697 in-situ | CG15697 | + | 1638 | 2523 | UPSTREAM | CG15697-RA | "-" | CG15697-RB | "-"


*********************** Rank 298 [Score 17.858215]   GBROWSE*******************

insitu CG5000 in-situ | msps | + | -14787 | -4929 | DOWNSTREAM | CG5000-RA | "-"
 CG10185 in-situ | CG10185 | - | 1902 | -4923 | INTRAGENIC | intron:CG10185-RA:5 | CG10185-RA | "-"


*********************** Rank 299 [Score 17.852600]   GBROWSE*******************

 CG30111 in-situ | CG30111 | + | -19980 | -16601 | DOWNSTREAM | CG30111-RA | "-"
 CG11430 in-situ | olf186-F | + | 9120 | 26245 | UPSTREAM | CG11430-RB | "-" | CG11430-RC | "-" | CG11430-RA | "-"


*********************** Rank 300 [Score 17.840393]   GBROWSE*******************

 CG7720 in-situ | CG7720 | - | -45107 | -81027 | UPSTREAM | CG7720-RB | "-" | CG7720-RA | "-"
 CG18208 in-situ | CG18208 | - | 8130 | -34110 | INTRAGENIC | intron:CG18208-RA:1 | CG18208-RA | "-"

*********************** Rank 301 [Score 17.838989]   GBROWSE*******************

 CG4478 in-situ | Mst35Bb | - | -10451 | -12065 | UPSTREAM | CG4478-RA | "-"
 CG15277 in-situ | CG15277 | - | 15601 | 14355 | DOWNSTREAM | CG15277-RA | "-"


*********************** Rank 302 [Score 17.819397]   GBROWSE*******************

insitu CG7724 in-situ | CG7724 | + | -20552 | -17637 | DOWNSTREAM | CG7724-RA | "-"
 CG32169 in-situ | CG32169 | - | 2031 | -9438 | INTRAGENIC | intron:CG32169-RA:2 | CG32169-RA | "-"

*********************** Rank 303 [Score 17.807922]   GBROWSE*******************

 CG1867 in-situ | Or98b | + | -53720 | -52342 | DOWNSTREAM | CG1867-RA | "-"
 CG14064 in-situ | beat-VI | + | 12242 | 67247 | UPSTREAM | CG14064-RA | "-"

*********************** Rank 304 [Score 17.804321]   GBROWSE*******************

 CG15214 in-situ | CG15214 | + | -38646 | -34650 | DOWNSTREAM | CG15214-RA | "-"
 CG4835 in-situ | CG4835 | + | 5894 | 9421 | UPSTREAM | CG4835-RA | "-"


*********************** Rank 305 [Score 17.790283]   GBROWSE*******************

 CG15283 in-situ | BG:DS08340.1 | - | -31232 | -34671 | UPSTREAM | CG15283-RA | "-"
insitu highlight CG4491 in-situ | noc | + | 8814 | 11971 | UPSTREAM | CG4491-RA | "-"


*********************** Rank 306 [Score 17.787720]   GBROWSE*******************

 CG32063 in-situ | CG32063 | + | -20834 | -17541 | DOWNSTREAM | CG32063-RB | "-" | CG32063-RA | "-"
 CG32064 in-situ | CG32064 | + | 4840 | 6914 | UPSTREAM | CG32064-RA | "-"

*********************** Rank 307 [Score 17.787048]   GBROWSE*******************

 CG14520 in-situ | CG14520 | + | -1594 | -997 | DOWNSTREAM | CG14520-RA | "-"
 CG31047 in-situ | CG31047 | - | 7057 | 6274 | DOWNSTREAM | CG31047-RA | "-"

*********************** Rank 308 [Score 17.782715]   GBROWSE*******************

insitu CG3619 in-situ | Dl | - | -38848 | -62330 | UPSTREAM | CG3619-RA | "-" | CG3619-RB | "-"
 CG3581 in-situ | CG3581 | - | 9688 | 8696 | DOWNSTREAM | CG3581-RA | "-"

*********************** Rank 309 [Score 17.781616]   GBROWSE*******************

 CG14551 in-situ | CG14551 | - | -21134 | -22051 | UPSTREAM | CG14551-RA | "-"
 CG32474 in-situ | cranky | - | 13182 | -5941 | INTRAGENIC | intron:CG32474-RA:4 | CG32474-RA | "-"

*********************** Rank 310 [Score 17.773621]   GBROWSE*******************

 CG12600 in-situ | CG12600 | - | -16951 | -21576 | UPSTREAM | CG12600-RA | "-"
 CG7552 in-situ | CG7552 | - | 3796 | 2847 | DOWNSTREAM | CG7552-RA | "-"

*********************** Rank 311 [Score 17.769531]   GBROWSE*******************

insitu CG31547 in-situ | CG31547 | - | -9529 | -22726 | UPSTREAM | CG31547-RB | "-" | CG31547-RA | "-"
 CG1063 in-situ | Itp-r83A | + | 4909 | 26631 | UPSTREAM | CG1063-RA | "-" | CG1063-RB | "-"


*********************** Rank 312 [Score 17.769287]   GBROWSE*******************

 CG10862 in-situ | CG10862 | - | -91300 | -92606 | UPSTREAM | CG10862-RA | "-"
 CG10858 in-situ | CG10858 | - | 9130 | 2454 | DOWNSTREAM | CG10858-RA | "-"

*********************** Rank 313 [Score 17.748901]   GBROWSE*******************

 CG13109 in-situ | tai | + | -6667 | 72921 | INTRAGENIC | intron:CG13109-RA:1 | CG13109-RA | "-"
 CG17009 in-situ | CG17009 | - | 77543 | 76827 | DOWNSTREAM | CG17009-RA | "-"

*********************** Rank 314 [Score 17.733154]   GBROWSE*******************

 CG4898 in-situ | Tm1 | + | -778 | 25683 | INTRAGENIC | intron:CG4898-RB:1 | intron:CG4898-RJ:1 | intron:CG4898-RD:1 | intron:CG4898-RE:1 | intron:CG4898-RG:1 | CG4898-RB | "-" | CG4898-RJ | "-" | CG4898-RD | "-" | CG4898-RE | "-" | CG4898-RG | "-" | CG4898-RH | "-" | CG4898-RC | "-" | CG4898-RI | "-" | CG4898-RA | "-"
insitu CG4843 in-situ | Tm2 | + | 27883 | 32802 | UPSTREAM | CG4843-RA | "-" | CG4843-RB | "-"

*********************** Rank 315 [Score 17.715210]   GBROWSE*******************

 CG31421 in-situ | CG31421 | - | -24693 | -26325 | UPSTREAM | CG31421-RA | "-"
 CG31194 in-situ | CG31194 | - | 17816 | 13543 | DOWNSTREAM | CG31194-RA | "-"

*********************** Rank 316 [Score 17.705444]   GBROWSE*******************

insitu CG3619 in-situ | Dl | - | -13698 | -37180 | UPSTREAM | CG3619-RA | "-" | CG3619-RB | "-"
 CG3581 in-situ | CG3581 | - | 34838 | 33846 | DOWNSTREAM | CG3581-RA | "-"

*********************** Rank 317 [Score 17.704468]   GBROWSE*******************

insitu CG10118 in-situ | ple | - | -347 | -6516 | UPSTREAM | CG10118-RA | "-" | CG10118-RB | "-"
 CG32394 in-situ | CG32394 | - | 19775 | 10287 | DOWNSTREAM | CG32394-RA | "-"

*********************** Rank 318 [Score 17.700012]   GBROWSE*******************

 CG11348 in-situ | nAcRbeta-64B | + | -2687 | 3942 | INTRAGENIC | intron:CG11348-RA:2 | CG11348-RA | "-" | CG11348-RB | "-"
 CG15021 in-situ | CG15021 | + | 4664 | 6263 | UPSTREAM | CG15021-RA | "-"

*********************** Rank 319 [Score 17.694031]   GBROWSE*******************

 CG8517 in-situ | CG8517 | + | -53604 | -52807 | DOWNSTREAM | CG8517-RA | "-"
 CG12501 in-situ | Or56a | - | 3546 | 1774 | DOWNSTREAM | CG12501-RA | "-"

*********************** Rank 320 [Score 17.693237]   GBROWSE*******************

 CG15145 in-situ | CG15145 | - | -13572 | -15500 | UPSTREAM | CG15145-RA | "-"
 CG7094 in-situ | CG7094 | - | 10798 | 9386 | DOWNSTREAM | CG7094-RA | "-"

*********************** Rank 321 [Score 17.678955]   GBROWSE*******************

 CG9523 in-situ | CG9523 | + | -8998 | -6962 | DOWNSTREAM | CG9523-RA | "-"
insitu CG9527 in-situ | CG9527 | - | 14682 | 11736 | DOWNSTREAM | CG9527-RA | "-"


*********************** Rank 322 [Score 17.673828]   GBROWSE*******************

insitu CG12214 in-situ | CG12214 | - | -33787 | -36297 | UPSTREAM | CG12214-RB | "-" | CG12214-RA | "-"
insitu CG33183 in-situ | Hr46 | - | 40724 | 12769 | DOWNSTREAM | CG33183-RC | "-" | CG33183-RB | "-" | CG33183-RA | "-"

*********************** Rank 323 [Score 17.670959]   GBROWSE*******************

 CG6414 in-situ | CG6414 | - | -48036 | -50386 | UPSTREAM | CG6414-RA | "-"
 CG32790 in-situ | CG32790 | + | 58532 | 59830 | UPSTREAM | CG32790-RA | "-"

*********************** Rank 324 [Score 17.666809]   GBROWSE*******************

 CG5097 in-situ | CG5097 | + | -17115 | -16925 | DOWNSTREAM | CG5097-RA | "-"
 CG4342 in-situ | CG4342 | - | 8335 | 3853 | DOWNSTREAM | CG4342-RA | "-"

*********************** Rank 325 [Score 17.665283]   GBROWSE*******************

insitu highlight CG1378 in-situ | tll | + | -3694 | -1689 | DOWNSTREAM | CG1378-RA | "-"
 CG12045 in-situ | CG12045 | + | 11461 | 13124 | UPSTREAM | CG12045-RA | "-"

note: overlaps known module tll_rescue by 312 bases (module coords: 26661256-26671461)

*********************** Rank 326 [Score 17.654297]   GBROWSE*******************

 CG5060 in-situ | CG5060 | + | -2778 | 35557 | INTRAGENIC | intron:CG5060-RA:1 | CG5060-RA | "-"
 CG10883 in-situ | CG10883 | - | 33252 | 32719 | DOWNSTREAM | CG10883-RA | "-"

*********************** Rank 327 [Score 17.649841]   GBROWSE*******************

 CG31769 in-situ | CG31769 | - | -4609 | -5757 | UPSTREAM | CG31769-RA | "-"
 CG15292 in-situ | CG15292 | - | 11817 | 11647 | DOWNSTREAM | CG15292-RA | "-"

*********************** Rank 328 [Score 17.647217]   GBROWSE*******************

 CG7527 in-situ | CadN2 | - | -75613 | -101612 | UPSTREAM | CG7527-RA | "-"
 CG5674 in-situ | CG5674 | + | 75121 | 86653 | UPSTREAM | CG5674-RA | "-" | CG5674-RB | "-" | CG5674-RC | "-"

*********************** Rank 329 [Score 17.632202]   GBROWSE*******************

 CG31495 in-situ | CG31495 | - | -9729 | -11421 | UPSTREAM | CG31495-RA | "-"
 CG14362 in-situ | CG14362 | - | 5790 | 5002 | DOWNSTREAM | CG14362-RA | "-"

*********************** Rank 330 [Score 17.623352]   GBROWSE*******************

 CG6112 in-situ | CG6112 | - | -2731 | -7122 | UPSTREAM | CG6112-RA | "-"
 CG32151 in-situ | CG32151 | + | 28911 | 29525 | UPSTREAM | CG32151-RA | "-"


*********************** Rank 331 [Score 17.610840]   GBROWSE*******************

insitu CG31632 in-situ | CG31632 | + | -25417 | -8729 | DOWNSTREAM | CG31632-RA | "-"
 CG10800 in-situ | Rca1 | + | 4590 | 6142 | UPSTREAM | CG10800-RA | "-"

*********************** Rank 332 [Score 17.604004]   GBROWSE*******************

 CG31923 in-situ | CG31923 | + | -33756 | -33068 | DOWNSTREAM | CG31923-RA | "-"
 CG4375 in-situ | CG4375 | - | 62463 | 61673 | DOWNSTREAM | CG4375-RA | "-"

*********************** Rank 333 [Score 17.598328]   GBROWSE*******************

 CG4328 in-situ | CG4328 | - | -12491 | -14417 | UPSTREAM | CG4328-RA | "-"
insitu highlight CG32105 in-situ | CG32105 | + | 23783 | 30453 | UPSTREAM | CG32105-RB | "-"


*********************** Rank 334 [Score 17.597412]   GBROWSE*******************

 CG12027 in-situ | CG12027 | - | -23895 | -24501 | UPSTREAM | CG12027-RA | "-"
 CG4597 in-situ | CG4597 | + | 75033 | 75599 | UPSTREAM | CG4597-RA | "-"


*********************** Rank 335 [Score 17.597412]   GBROWSE*******************

 CG31695 in-situ | scw | - | -4332 | -5743 | UPSTREAM | CG31695-RA | "-"
insitu CG10462 in-situ | CG10462 | - | 30581 | 26801 | DOWNSTREAM | CG10462-RA | "-"


*********************** Rank 336 [Score 17.597412]   GBROWSE*******************

 CG7968 in-situ | BG:DS00941.15 | + | -5486 | -4571 | DOWNSTREAM | CG7968-RA | "-"
insitu CG8954 in-situ | CG8954 | - | 49761 | 44455 | DOWNSTREAM | CG8954-RA | "-" | CG8954-RB | "-"


*********************** Rank 337 [Score 17.597412]   GBROWSE*******************

insitu CG12214 in-situ | CG12214 | - | -19837 | -22347 | UPSTREAM | CG12214-RB | "-" | CG12214-RA | "-"
insitu CG33183 in-situ | Hr46 | - | 54674 | 26719 | DOWNSTREAM | CG33183-RC | "-" | CG33183-RB | "-" | CG33183-RA | "-"


*********************** Rank 338 [Score 17.597412]   GBROWSE*******************

 CG31644 in-situ | CG31644 | - | -11042 | -11543 | UPSTREAM | CG31644-RA | "-"
 CG14004 in-situ | CG14004 | + | 15917 | 16525 | UPSTREAM | CG14004-RA | "-"


*********************** Rank 339 [Score 17.597412]   GBROWSE*******************

 CG31923 in-situ | CG31923 | + | -67906 | -67218 | DOWNSTREAM | CG31923-RA | "-"
 CG4375 in-situ | CG4375 | - | 28313 | 27523 | DOWNSTREAM | CG4375-RA | "-"


*********************** Rank 340 [Score 17.597412]   GBROWSE*******************

 CG12643 in-situ | CG12643 | - | -3885 | -4918 | UPSTREAM | CG12643-RA | "-"
 CG32682 in-situ | CG32682 | - | 39419 | 38691 | DOWNSTREAM | CG32682-RA | "-"


*********************** Rank 341 [Score 17.597412]   GBROWSE*******************

insitu highlight CG17228 in-situ | pros | + | -8272 | 12544 | INTRAGENIC | intron:CG17228-RA:2 | intron:CG17228-RD:3 | intron:CG17228-RC:3 | CG17228-RA | "-" | CG17228-RD | "-" | CG17228-RC | "-"
insitu CG5242 in-situ | mRpL22-24 | + | 26863 | 27700 | UPSTREAM | CG5242-RA | "-"


*********************** Rank 342 [Score 17.582764]   GBROWSE*******************

 CG33111 in-situ | CG33111 | - | -23089 | -40564 | UPSTREAM | CG33111-RA | "-"
 CG12492 in-situ | CG12492 | + | 3085 | 7028 | UPSTREAM | CG12492-RA | "-"

*********************** Rank 343 [Score 17.565491]   GBROWSE*******************

 CG15321 in-situ | CG15321 | - | -7244 | -7951 | UPSTREAM | CG15321-RA | "-"
insitu highlight CG12653 in-situ | btd | + | 3272 | 6657 | UPSTREAM | CG12653-RA | "-"

note: overlaps known module btd_head by 500 bases (module coords: 9429057-9430856)

*********************** Rank 344 [Score 17.552490]   GBROWSE*******************

 CG13958 in-situ | CG13958 | + | -32949 | -31333 | DOWNSTREAM | CG13958-RA | "-"
 CG13959 in-situ | CG13959 | + | 26013 | 27101 | UPSTREAM | CG13959-RA | "-"

*********************** Rank 345 [Score 17.547607]   GBROWSE*******************

 CG17278 in-situ | BcDNA:SD04019 | - | -9498 | -12627 | UPSTREAM | CG17278-RA | "-"
 CG3723 in-situ | Dhc93AB | - | 8758 | -8642 | INTRAGENIC | intron:CG3723-RA:12 | CG3723-RA | "-"

*********************** Rank 346 [Score 17.506104]   GBROWSE*******************

 CG10633 in-situ | CG10633 | - | -46371 | -50406 | UPSTREAM | CG10633-RA | "-"
 CG4814 in-situ | CG4814 | + | 3815 | 5085 | UPSTREAM | CG4814-RA | "-"


*********************** Rank 347 [Score 17.497559]   GBROWSE*******************

 CG1964 in-situ | CG1964 | + | -3534 | 2104 | INTRAGENIC | intron:CG1964-RA:7 | CG1964-RA | "-"
 CG7601 in-situ | BcDNA:GH06026 | - | 6258 | 4706 | DOWNSTREAM | CG7601-RA | "-"

*********************** Rank 348 [Score 17.493164]   GBROWSE*******************

 CG15179 in-situ | CG15179 | + | -6927 | -5892 | DOWNSTREAM | CG15179-RA | "-"
 CG31286 in-situ | CG31286 | + | 4155 | 6271 | UPSTREAM | CG31286-RB | "-" | CG31286-RC | "-"

*********************** Rank 349 [Score 17.492188]   GBROWSE*******************

 CG13964 in-situ | CG13964 | + | -8158 | -6752 | DOWNSTREAM | CG13964-RA | "-"
 CG10662 in-situ | CG10662 | + | 872 | 3929 | UPSTREAM | CG10662-RA | "-"

*********************** Rank 350 [Score 17.462585]   GBROWSE*******************

insitu highlight CG6634 in-situ | CG6634 | + | -27444 | -21476 | DOWNSTREAM | CG6634-RA | "-"
 CG14020 in-situ | CG14020 | + | 28757 | 29737 | UPSTREAM | CG14020-RA | "-"

*********************** Rank 351 [Score 17.454346]   GBROWSE*******************

 CG10039 in-situ | CG10039 | + | -13278 | -11982 | DOWNSTREAM | CG10039-RA | "-"
 CG12676 in-situ | ed | + | 10398 | 94078 | UPSTREAM | CG12676-RA | "-"

*********************** Rank 352 [Score 17.442261]   GBROWSE*******************

insitu highlight CG1897 in-situ | Dr | + | -11825 | -2927 | DOWNSTREAM | CG1897-RA | "-"
insitu CG7567 in-situ | CG7567 | - | 19888 | 19024 | DOWNSTREAM | CG7567-RA | "-"

*********************** Rank 353 [Score 17.432739]   GBROWSE*******************

 CG16918 in-situ | CG16918 | + | -8159 | -3425 | DOWNSTREAM | CG16918-RA | "-"
 CG11719 in-situ | Mst98Ca | + | 1471 | 2801 | UPSTREAM | CG11719-RA | "-"

*********************** Rank 354 [Score 17.427002]   GBROWSE*******************

insitu CG8440 in-situ | Lis1 | + | -571 | 5732 | INTRAGENIC | intron:CG8440-RA:1 | intron:CG8440-RC:1 | intron:CG8440-RD:1 | intron:CG8440-RE:1 | intron:CG8440-RB:1 | CG8440-RA | "-" | CG8440-RC | "-" | CG8440-RD | "-" | CG8440-RE | "-" | CG8440-RB | "-"
 CG8441 in-situ | CG8441 | - | 6626 | 5739 | DOWNSTREAM | CG8441-RA | "-"


*********************** Rank 355 [Score 17.422363]   GBROWSE*******************

 CG31275 in-situ | CG31275 | - | -29521 | -30178 | UPSTREAM | CG31275-RB | "-" | CG31275-RA | "-"
 CG3853 in-situ | Glut3 | + | 7401 | 9113 | UPSTREAM | CG3853-RA | "-"

*********************** Rank 356 [Score 17.406982]   GBROWSE*******************

 CG6599 in-situ | CG6599 | + | -6273 | -1333 | DOWNSTREAM | CG6599-RA | "-"
 CG13980 in-situ | CG13980 | + | 3482 | 5317 | UPSTREAM | CG13980-RA | "-"

*********************** Rank 357 [Score 17.378418]   GBROWSE*******************

insitu highlight CG12653 in-situ | btd | + | -11578 | -8193 | DOWNSTREAM | CG12653-RA | "-"
 CG1343 in-situ | Sp1 | + | 47193 | 49503 | UPSTREAM | CG1343-RA | "-" | CG1343-RB | "-"

*********************** Rank 358 [Score 17.375610]   GBROWSE*******************

 CG15480 in-situ | CG15480 | - | -174 | -674 | UPSTREAM | CG15480-RA | "-"
 CG16813 in-situ | CG16813 | + | 2473 | 3027 | UPSTREAM | CG16813-RA | "-"

*********************** Rank 359 [Score 17.374329]   GBROWSE*******************

 CG13980 in-situ | CG13980 | + | -17568 | -15733 | DOWNSTREAM | CG13980-RA | "-"
 CG12509 in-situ | CG12509 | - | 21445 | 21152 | DOWNSTREAM | CG12509-RA | "-"


*********************** Rank 360 [Score 17.371704]   GBROWSE*******************

 CG13826 in-situ | CG13826 | - | -8113 | -12657 | UPSTREAM | CG13826-RA | "-"
 CG4467 in-situ | CG4467 | - | 5228 | -4760 | INTRAGENIC | intron:CG4467-RA:3 | CG4467-RA | "-"

*********************** Rank 361 [Score 17.369873]   GBROWSE*******************

 CG11913 in-situ | CG11913 | + | -15513 | -13892 | DOWNSTREAM | CG11913-RA | "-"
insitu CG11910 in-situ | CG11910 | - | 8979 | 7242 | DOWNSTREAM | CG11910-RA | "-"

*********************** Rank 362 [Score 17.349854]   GBROWSE*******************

 CG11522 in-situ | CG11522 | - | -11367 | -12825 | UPSTREAM | CG11522-RA | "-" | CG11522-RB | "-"
 CG1775 in-situ | Med | + | 3436 | 8144 | UPSTREAM | CG1775-RA | "-" | CG1775-RB | "-" | CG1775-RC | "-" | CG1775-RD | "-"


*********************** Rank 363 [Score 17.330933]   GBROWSE*******************

 CG7843 in-situ | CG7843 | - | -7512 | -12304 | UPSTREAM | CG7843-RA | "-" | CG7843-RB | "-" | CG7843-RD | "-" | CG7843-RC | "-"
 CG14589 in-situ | CG14589 | + | 44258 | 45319 | UPSTREAM | CG14589-RA | "-"


*********************** Rank 364 [Score 17.323975]   GBROWSE*******************

 CG7060 in-situ | CG7060 | + | -4779 | 617 | INTRAGENIC | intron:CG7060-RA:9 | CG7060-RA | "-"
 CG7207 in-situ | BcDNA:GH07688 | - | 9541 | 2231 | DOWNSTREAM | CG7207-RA | "-"

*********************** Rank 365 [Score 17.320068]   GBROWSE*******************

insitu CG17932 in-situ | Ugt36Bc | + | -4887 | -2331 | DOWNSTREAM | CG17932-RA | "-" | CG17932-RB | "-"
 CG13272 in-situ | CG13272 | + | 7749 | 11877 | UPSTREAM | CG13272-RA | "-"


*********************** Rank 366 [Score 17.316101]   GBROWSE*******************

 CG32655 in-situ | CG32655 | - | -62161 | -63219 | UPSTREAM | CG32655-RA | "-"
 CG2577 in-situ | CG2577 | + | 75321 | 76561 | UPSTREAM | CG2577-RA | "-"


*********************** Rank 367 [Score 17.302979]   GBROWSE*******************

insitu highlight CG1849 in-situ | run | + | -38394 | -35509 | DOWNSTREAM | CG1849-RA | "-"
 CG1324 in-situ | CG1324 | - | 3125 | 1909 | DOWNSTREAM | CG1324-RA | "-"

*********************** Rank 368 [Score 17.294312]   GBROWSE*******************

 CG12066 in-situ | Pka-C2 | - | -1074 | -2684 | UPSTREAM | CG12066-RA | "-" | CG12066-RB | "-"
 CG31010 in-situ | CG31010 | - | 7881 | 6547 | DOWNSTREAM | CG31010-RA | "-"

*********************** Rank 369 [Score 17.281128]   GBROWSE*******************

 CG2224 in-situ | CG2224 | - | -274 | -2708 | UPSTREAM | CG2224-RA | "-"
 CG1471 in-situ | CG1471 | + | 127 | 6074 | UPSTREAM | CG1471-RB | "-" | CG1471-RA | "-" | CG1471-RC | "-" | CG1471-RD | "-" | CG1471-RE | "-"


*********************** Rank 370 [Score 17.275879]   GBROWSE*******************

insitu CG12701 in-situ | CG12701 | - | -23612 | -30553 | UPSTREAM | CG12701-RB | "-" | CG12701-RA | "-"
 CG12700 in-situ | skpD | + | 6189 | 6848 | UPSTREAM | CG12700-RA | "-"

*********************** Rank 371 [Score 17.271729]   GBROWSE*******************

 CG10847 in-situ | enc | + | -24684 | -2163 | DOWNSTREAM | CG10847-RB | "-"
 CG1072 in-situ | Awh | + | 2782 | 18326 | UPSTREAM | CG1072-RA | "-" | CG1072-RB | "-"


*********************** Rank 372 [Score 17.267822]   GBROWSE*******************

 CG9074 in-situ | Mst57Da | - | -7737 | -8041 | UPSTREAM | CG9074-RA | "-"
 CG5878 in-situ | CG5878 | + | 2530 | 2952 | UPSTREAM | CG5878-RA | "-"

*********************** Rank 373 [Score 17.267822]   GBROWSE*******************

insitu highlight CG17390 in-situ | CG17390 | + | -15488 | -8767 | DOWNSTREAM | CG17390-RA | "-"
 CG10109 in-situ | L | + | 6979 | 23088 | UPSTREAM | CG10109-RA | "-"

*********************** Rank 374 [Score 17.267822]   GBROWSE*******************

insitu CG31264 in-situ | CG31264 | - | -11927 | -30745 | UPSTREAM | CG31264-RA | "-"
 CG11732 in-situ | Obp85a | - | 14872 | 9909 | DOWNSTREAM | CG11732-RA | "-"

*********************** Rank 375 [Score 17.267822]   GBROWSE*******************

 CG17952 in-situ | CG17952 | + | -24702 | -21140 | DOWNSTREAM | CG17952-RB | "-" | CG17952-RA | "-" | CG17952-RC | "-"
 CG7975 in-situ | Grx-1 | + | 12228 | 12779 | UPSTREAM | CG7975-RA | "-"

*********************** Rank 376 [Score 17.266907]   GBROWSE*******************

 CG5151 in-situ | CG5151 | - | -13487 | -22738 | UPSTREAM | CG5151-RA | "-"
 CG13073 in-situ | CG13073 | - | 51885 | 50830 | DOWNSTREAM | CG13073-RB | "-" | CG13073-RA | "-"

*********************** Rank 377 [Score 17.260559]   GBROWSE*******************

 CG14390 in-situ | beat-Vc | - | -29558 | -37644 | UPSTREAM | CG14390-RA | "-"
 CG31345 in-situ | CG31345 | - | 32770 | 27086 | DOWNSTREAM | CG31345-RA | "-"

*********************** Rank 378 [Score 17.257446]   GBROWSE*******************

 CG12425 in-situ | CG12425 | + | -63538 | -61983 | DOWNSTREAM | CG12425-RA | "-"
 CG4787 in-situ | CG4787 | + | 24737 | 27023 | UPSTREAM | CG4787-RA | "-"


*********************** Rank 379 [Score 17.255615]   GBROWSE*******************

 CG31856 in-situ | CG31856 | - | -32085 | -34390 | UPSTREAM | CG31856-RA | "-"
 CG5142 in-situ | CG5142 | + | 955 | 3479 | UPSTREAM | CG5142-RA | "-"

*********************** Rank 380 [Score 17.251770]   GBROWSE*******************

 CG4374 in-situ | CG4374 | - | -59164 | -63516 | UPSTREAM | CG4374-RA | "-"
 CG31225 in-situ | CG31225 | + | 10196 | 11770 | UPSTREAM | CG31225-RA | "-"

*********************** Rank 381 [Score 17.235657]   GBROWSE*******************

 CG7320 in-situ | CG7320 | + | -10882 | -9155 | DOWNSTREAM | CG7320-RA | "-"
 CG7313 in-situ | CG7313 | + | 39376 | 40125 | UPSTREAM | CG7313-RA | "-"

*********************** Rank 382 [Score 17.234253]   GBROWSE*******************

 CG12165 in-situ | Incenp | + | -13082 | -9988 | DOWNSTREAM | CG12165-RA | "-"
 CG11101 in-situ | pwn | + | 3759 | 8853 | UPSTREAM | CG11101-RA | "-"

*********************** Rank 383 [Score 17.231201]   GBROWSE*******************

 CG31257 in-situ | CG31257 | + | -4448 | -2401 | DOWNSTREAM | CG31257-RA | "-"
 CG31418 in-situ | CG31418 | + | 25282 | 26002 | UPSTREAM | CG31418-RA | "-"

*********************** Rank 384 [Score 17.229492]   GBROWSE*******************

 CG17178 in-situ | ACXE | + | -5611 | -1475 | DOWNSTREAM | CG17178-RA | "-"
 CG16800 in-situ | CG16800 | + | 35600 | 36558 | UPSTREAM | CG16800-RA | "-"

*********************** Rank 385 [Score 17.221436]   GBROWSE*******************

insitu CG10710 in-situ | CG10710 | - | -84431 | -87759 | UPSTREAM | CG10710-RA | "-"
 CG12478 in-situ | bru-3 | - | 62717 | -66114 | INTRAGENIC | intron:CG12478-RA:4 | intron:CG12478-RB:3 | CG12478-RA | "-" | CG12478-RB | "-"

*********************** Rank 386 [Score 17.197449]   GBROWSE*******************

 CG14680 in-situ | Cyp12e1 | + | -22510 | -20647 | DOWNSTREAM | CG14680-RA | "-" | CG14680-RB | "-"
 CG31395 in-situ | CG31395 | + | 15806 | 16501 | UPSTREAM | CG31395-RA | "-"

*********************** Rank 387 [Score 17.180176]   GBROWSE*******************

 CG31640 in-situ | CG31640 | + | -16142 | -3701 | DOWNSTREAM | CG31640-RA | "-"
insitu highlight CG9481 in-situ | Ugt37b1 | + | 12465 | 14257 | UPSTREAM | CG9481-RA | "-"

*********************** Rank 388 [Score 17.174255]   GBROWSE*******************

 CG13291 in-situ | CG13291 | - | -17115 | -18077 | UPSTREAM | CG13291-RA | "-"
insitu highlight CG10479 in-situ | CG10479 | - | 4463 | -4001 | INTRAGENIC | intron:CG10479-RA:1 | CG10479-RA | "-"

*********************** Rank 389 [Score 17.173096]   GBROWSE*******************

 CG4683 in-situ | CG4683 | + | -40241 | -38886 | DOWNSTREAM | CG4683-RA | "-"
 CG14698 in-situ | CG14698 | + | 43935 | 44461 | UPSTREAM | CG14698-RA | "-"

*********************** Rank 390 [Score 17.161133]   GBROWSE*******************

 CG10236 in-situ | LanA | - | -9837 | -23990 | UPSTREAM | CG10236-RA | "-"
 CG13299 in-situ | CG13299 | - | 4503 | 4102 | DOWNSTREAM | CG13299-RA | "-"

*********************** Rank 391 [Score 17.161133]   GBROWSE*******************

 CG32192 in-situ | CG32192 | + | -14204 | -13469 | DOWNSTREAM | CG32192-RA | "-" | CG32192-RB | "-"
 CG8127 in-situ | Eip75B | - | 5077 | -102558 | INTRAGENIC | intron:CG8127-RB:1 | CG8127-RB | "-" | CG8127-RC | "-" | CG8127-RA | "-" | CG8127-RD | "-"

*********************** Rank 392 [Score 17.161133]   GBROWSE*******************

 CG3199 in-situ | CG3199 | + | -38640 | -37734 | DOWNSTREAM | CG3199-RA | "-"
 CG9652 in-situ | DopR | - | 13328 | -24076 | INTRAGENIC | intron:CG9652-RA:3 | CG9652-RA | "-"

*********************** Rank 393 [Score 17.161133]   GBROWSE*******************

 CG9665 in-situ | CG9665 | - | -11031 | -13568 | UPSTREAM | CG9665-RA | "-"
 CG11661 in-situ | CG11661 | + | 2835 | 15415 | UPSTREAM | CG11661-RC | "-" | CG11661-RF | "-" | CG11661-RG | "-" | CG11661-RB | "-" | CG11661-RE | "-" | CG11661-RA | "-" | CG11661-RH | "-"

*********************** Rank 394 [Score 17.161133]   GBROWSE*******************

 CG12454 in-situ | CG12454 | + | -42399 | -42211 | DOWNSTREAM | CG12454-RA | "-"
 CG32614 in-situ | CG32614 | + | 20117 | 20827 | UPSTREAM | CG32614-RA | "-"

*********************** Rank 395 [Score 17.158203]   GBROWSE*******************

 CG11634 in-situ | CG11634 | + | -17063 | -15976 | DOWNSTREAM | CG11634-RA | "-"
 CG2528 in-situ | CG2528 | - | 59509 | 57407 | DOWNSTREAM | CG2528-RA | "-"

*********************** Rank 396 [Score 17.152954]   GBROWSE*******************

 CG5423 in-situ | robo3 | + | -45658 | -8649 | DOWNSTREAM | CG5423-RA | "-"
 CG5430 in-situ | a5 | + | 26677 | 27420 | UPSTREAM | CG5430-RA | "-"


*********************** Rank 397 [Score 17.149780]   GBROWSE*******************

 CG8476 in-situ | CG8476 | - | -6331 | -7269 | UPSTREAM | CG8476-RA | "-"
insitu CG17907 in-situ | Ace | - | 31959 | 1331 | DOWNSTREAM | CG17907-RA | "-"


*********************** Rank 398 [Score 17.149658]   GBROWSE*******************

 CG10803 in-situ | CG10803 | + | -9260 | -6005 | DOWNSTREAM | CG10803-RA | "-"
 CG14271 in-situ | Gas8 | - | 9938 | -3904 | INTRAGENIC | intron:CG14271-RB:2 | CG14271-RB | "-"


*********************** Rank 399 [Score 17.149109]   GBROWSE*******************

 CG5097 in-situ | CG5097 | + | -2515 | -2325 | DOWNSTREAM | CG5097-RA | "-"
 CG4342 in-situ | CG4342 | - | 22935 | 18453 | DOWNSTREAM | CG4342-RA | "-"

*********************** Rank 400 [Score 17.144775]   GBROWSE*******************

 CG13109 in-situ | tai | + | -81867 | -2279 | DOWNSTREAM | CG13109-RA | "-"
 CG17009 in-situ | CG17009 | - | 2343 | 1627 | DOWNSTREAM | CG17009-RA | "-"

*********************** Rank 401 [Score 17.140015]   GBROWSE*******************

 CG8404 in-situ | Sox15 | - | -65 | -11091 | UPSTREAM | CG8404-RA | "-"
 CG8415 in-situ | CG8415 | - | 4153 | 2761 | DOWNSTREAM | CG8415-RA | "-"

*********************** Rank 402 [Score 17.137939]   GBROWSE*******************

insitu highlight CG16738 in-situ | slp1 | + | -3351 | -1894 | DOWNSTREAM | CG16738-RA | "-"
insitu highlight CG2939 in-situ | slp2 | + | 7811 | 10170 | UPSTREAM | CG2939-RA | "-"

*********************** Rank 403 [Score 17.117554]   GBROWSE*******************

 CG5432 in-situ | CG5432 | + | -6875 | -5574 | DOWNSTREAM | CG5432-RA | "-"
 CG6503 in-situ | CG6503 | - | 4142 | 3734 | DOWNSTREAM | CG6503-RA | "-"


*********************** Rank 404 [Score 17.097839]   GBROWSE*******************

 CG31395 in-situ | CG31395 | + | -12744 | -12049 | DOWNSTREAM | CG31395-RA | "-"
 CG31397 in-situ | CG31397 | + | 17879 | 18547 | UPSTREAM | CG31397-RA | "-"


*********************** Rank 405 [Score 17.082764]   GBROWSE*******************

 CG4568 in-situ | fzo | - | -1937 | -4335 | UPSTREAM | CG4568-RA | "-"
insitu highlight CG17894 in-situ | cnc | - | 15990 | -20393 | INTRAGENIC | intron:CG17894-RC:5 | CG17894-RC | "-" | CG17894-RB | "-" | CG17894-RG | "-" | CG17894-RF | "-" | CG17894-RE | "-" | CG17894-RD | "-" | CG17894-RA | "-"


*********************** Rank 406 [Score 17.074463]   GBROWSE*******************

 CG31275 in-situ | CG31275 | - | -35471 | -36128 | UPSTREAM | CG31275-RB | "-" | CG31275-RA | "-"
 CG3853 in-situ | Glut3 | + | 1451 | 3163 | UPSTREAM | CG3853-RA | "-"

*********************** Rank 407 [Score 17.072998]   GBROWSE*******************

insitu CG11395 in-situ | CG11395 | + | -15834 | -14243 | DOWNSTREAM | CG11395-RA | "-"
 CG17290 in-situ | CG17290 | + | 1624 | 2235 | UPSTREAM | CG17290-RA | "-" | CG17290-RB | "-"


*********************** Rank 408 [Score 17.052734]   GBROWSE*******************

 CG7727 in-situ | Appl | + | -26443 | 20076 | INTRAGENIC | intron:CG7727-RA:1 | CG7727-RA | "-"
insitu highlight CG6172 in-situ | vnd | + | 32979 | 39759 | UPSTREAM | CG6172-RA | "-"

*********************** Rank 409 [Score 17.052734]   GBROWSE*******************

 CG31481 in-situ | CG31481 | - | -21801 | -26619 | UPSTREAM | CG31481-RA | "-"
 CG1048 in-situ | zen2 | - | 9313 | 8305 | DOWNSTREAM | CG1048-RA | "-"

*********************** Rank 410 [Score 17.052734]   GBROWSE*******************

 CG31031 in-situ | CG31031 | + | -52234 | -51770 | DOWNSTREAM | CG31031-RA | "-"
 CG18682 in-situ | CG18682 | - | 9423 | 6742 | DOWNSTREAM | CG18682-RA | "-"

*********************** Rank 411 [Score 17.044067]   GBROWSE*******************

 CG32725 in-situ | CG32725 | - | -16412 | -17139 | UPSTREAM | CG32725-RA | "-"
 CG1958 in-situ | CG1958 | + | 12331 | 13308 | UPSTREAM | CG1958-RA | "-"

*********************** Rank 412 [Score 17.043762]   GBROWSE*******************

insitu highlight CG10798 in-situ | dm | + | -14929 | -2096 | DOWNSTREAM | CG10798-RA | "-"
 CG12535 in-situ | CG12535 | - | 5302 | 4517 | DOWNSTREAM | CG12535-RB | "-" | CG12535-RA | "-"

*********************** Rank 413 [Score 17.042175]   GBROWSE*******************

insitu CG17932 in-situ | Ugt36Bc | + | -9387 | -6831 | DOWNSTREAM | CG17932-RA | "-" | CG17932-RB | "-"
 CG13272 in-situ | CG13272 | + | 3249 | 7377 | UPSTREAM | CG13272-RA | "-"

*********************** Rank 414 [Score 17.039062]   GBROWSE*******************

 CG1088 in-situ | Vha26 | + | -5900 | -2924 | DOWNSTREAM | CG1088-RB | "-" | CG1088-RA | "-"
 CG2922 in-situ | eIF-5C | - | 3538 | -2267 | INTRAGENIC | intron:CG2922-RA:5 | intron:CG2922-RG:5 | intron:CG2922-RC:6 | intron:CG2922-RF:5 | intron:CG2922-RD:5 | intron:CG2922-RB:6 | intron:CG2922-RE:4 | CG2922-RA | "-" | CG2922-RG | "-" | CG2922-RC | "-" | CG2922-RF | "-" | CG2922-RD | "-" | CG2922-RB | "-" | CG2922-RE | "-"

*********************** Rank 415 [Score 17.031982]   GBROWSE*******************

insitu highlight CG6634 in-situ | CG6634 | + | -19494 | -13526 | DOWNSTREAM | CG6634-RA | "-"
 CG14020 in-situ | CG14020 | + | 36707 | 37687 | UPSTREAM | CG14020-RA | "-"

*********************** Rank 416 [Score 17.014160]   GBROWSE*******************

insitu CG31337 in-situ | CG31337 | - | -8775 | -10187 | UPSTREAM | CG31337-RA | "-"
 CG14370 in-situ | CG14370 | + | 34028 | 34426 | UPSTREAM | CG14370-RA | "-"

*********************** Rank 417 [Score 17.008423]   GBROWSE*******************

 CG10131 in-situ | CG10131 | + | -4511 | -3403 | DOWNSTREAM | CG10131-RA | "-"
 CG12862 in-situ | CG12862 | - | 6467 | 5790 | DOWNSTREAM | CG12862-RB | "-" | CG12862-RA | "-"

*********************** Rank 418 [Score 17.004272]   GBROWSE*******************

 CG6559 in-situ | CG6559 | - | -52408 | -68646 | UPSTREAM | CG6559-RA | "-"
 CG12362 in-situ | CG12362 | + | 47695 | 49656 | UPSTREAM | CG12362-RB | "-" | CG12362-RA | "-"

*********************** Rank 419 [Score 16.999756]   GBROWSE*******************

 CG17681 in-situ | CG17681 | + | -1788 | 10093 | INTRAGENIC | intron:CG17681-RA:1 | CG17681-RA | "-" | CG17681-RB | "-"
 CG15154 in-situ | Socs36E | - | 8093 | -5649 | INTRAGENIC | intron:CG15154-RA:1 | intron:CG15154-RB:1 | CG15154-RA | "-" | CG15154-RB | "-"

*********************** Rank 420 [Score 16.991333]   GBROWSE*******************

insitu CG12075 in-situ | CG12075 | + | -8888 | -866 | DOWNSTREAM | CG12075-RA | "-"
insitu highlight CG10701 in-situ | Moe | - | 18800 | 602 | DOWNSTREAM | CG10701-RD | "-" | CG10701-RB | "-" | CG10701-RA | "-" | CG10701-RC | "-"

*********************** Rank 421 [Score 16.985718]   GBROWSE*******************

insitu highlight CG5461 in-situ | bun | - | -38659 | -122652 | UPSTREAM | CG5461-RA | "-" | CG5461-RB | "-" | CG5461-RC | "-"
 CG15489 in-situ | CG15489 | + | 14558 | 15460 | UPSTREAM | CG15489-RA | "-"

*********************** Rank 422 [Score 16.984863]   GBROWSE*******************

 CG12283 in-situ | kek1 | - | -80143 | -84000 | UPSTREAM | CG12283-RA | "-"
 CG5983 in-situ | ACXC | + | 9600 | 13844 | UPSTREAM | CG5983-RA | "-"

*********************** Rank 423 [Score 16.981262]   GBROWSE*******************

 CG1789 in-situ | CG1789 | + | -38794 | -37850 | DOWNSTREAM | CG1789-RA | "-"
 CG11354 in-situ | Lim1 | - | 46424 | -2116 | INTRAGENIC | intron:CG11354-RA:5 | CG11354-RA | "-"

*********************** Rank 424 [Score 16.977173]   GBROWSE*******************

 CG2617 in-situ | CG2617 | + | -11338 | -10076 | DOWNSTREAM | CG2617-RA | "-"
 CG1864 in-situ | Hr38 | - | 20881 | -10248 | INTRAGENIC | intron:CG1864-RB:1 | CG1864-RB | "-" | CG1864-RC | "-"

*********************** Rank 425 [Score 16.974243]   GBROWSE*******************

 CG4437 in-situ | PGRP-LF | + | -3179 | -1301 | DOWNSTREAM | CG4437-RA | "-"
 CG32040 in-situ | CG32040 | + | 1546 | 2548 | UPSTREAM | CG32040-RA | "-"


*********************** Rank 426 [Score 16.965698]   GBROWSE*******************

 CG7446 in-situ | Grd | + | -1463 | 8472 | INTRAGENIC | intron:CG7446-RA:1 | CG7446-RA | "-"
 CG32188 in-situ | CG32188 | - | 3173 | 2856 | DOWNSTREAM | CG32188-RA | "-"

*********************** Rank 427 [Score 16.963257]   GBROWSE*******************

 CG14358 in-situ | CG14358 | - | -2513 | -3073 | UPSTREAM | CG14358-RA | "-"
 CG9920 in-situ | CG9920 | - | 2371 | 1577 | DOWNSTREAM | CG9920-RA | "-"

*********************** Rank 428 [Score 16.943726]   GBROWSE*******************

insitu highlight CG1030 in-situ | Scr | - | -8608 | -34107 | UPSTREAM | CG1030-RA | "-"
insitu highlight CG2047 in-situ | ftz | + | 7097 | 9001 | UPSTREAM | CG2047-RA | "-"

*********************** Rank 429 [Score 16.942139]   GBROWSE*******************

 CG9380 in-situ | CG9380 | - | -36393 | -41601 | UPSTREAM | CG9380-RA | "-" | CG9380-RB | "-"
insitu highlight CG3340 in-situ | Kr | + | 1385 | 4304 | UPSTREAM | CG3340-RA | "-"


note: overlaps known module Kr_CD2_AD1 by 500 bases (module coords: 20267622-20269328)
note: overlaps known module Kr_CDHK by 500 bases (module coords: 20268583-20269949)

*********************** Rank 430 [Score 16.940552]   GBROWSE*******************

 CG31172 in-situ | CG31172 | - | -3620 | -4488 | UPSTREAM | CG31172-RA | "-"
 CG13627 in-situ | CG13627 | + | 37963 | 42732 | UPSTREAM | CG13627-RA | "-" | CG13627-RB | "-"


*********************** Rank 431 [Score 16.932983]   GBROWSE*******************

 CG12676 in-situ | ed | + | -38802 | 44878 | INTRAGENIC | intron:CG12676-RA:2 | CG12676-RA | "-"
 CG31962 in-situ | Sr-CIII | + | 50182 | 51261 | UPSTREAM | CG31962-RA | "-"


*********************** Rank 432 [Score 16.916626]   GBROWSE*******************

 CG30358 in-situ | CG30358 | + | -20006 | -19313 | DOWNSTREAM | CG30358-RA | "-"
 CG14755 in-situ | CG14755 | + | 7686 | 8540 | UPSTREAM | CG14755-RA | "-"


*********************** Rank 433 [Score 16.905151]   GBROWSE*******************

 CG14974 in-situ | CG14974 | - | -12972 | -13495 | UPSTREAM | CG14974-RA | "-"
 CG12029 in-situ | CG12029 | + | 13195 | 18931 | UPSTREAM | CG12029-RA | "-"

*********************** Rank 434 [Score 16.904053]   GBROWSE*******************

 CG16898 in-situ | CG16898 | - | -40451 | -41819 | UPSTREAM | CG16898-RA | "-"
 CG8896 in-situ | 18w | + | 66073 | 71494 | UPSTREAM | CG8896-RA | "-"

*********************** Rank 435 [Score 16.902283]   GBROWSE*******************

 CG31772 in-situ | CG31772 | + | -8813 | -690 | DOWNSTREAM | CG31772-RA | "-"
insitu highlight CG10021 in-situ | bowl | + | 2825 | 15248 | UPSTREAM | CG10021-RB | "-" | CG10021-RC | "-" | CG10021-RD | "-" | CG10021-RA | "-"


*********************** Rank 436 [Score 16.901733]   GBROWSE*******************

 CG10844 in-situ | Rya-r44F | + | -10162 | 17544 | INTRAGENIC | intron:CG10844-RC:9 | intron:CG10844-RB:9 | intron:CG10844-RD:9 | intron:CG10844-RA:9 | CG10844-RC | "-" | CG10844-RB | "-" | CG10844-RD | "-" | CG10844-RA | "-"
 CG8272 in-situ | CG8272 | - | 20846 | 17853 | DOWNSTREAM | CG8272-RA | "-"

*********************** Rank 437 [Score 16.901001]   GBROWSE*******************

insitu CG32954 in-situ | CG32954 | + | -17793 | -14446 | DOWNSTREAM | CG32954-RA | "-" | CG32954-RB | "-" | CG32954-RC | "-" | CG32954-RG | "-" | CG32954-RH | "-" | CG32954-RF | "-" | CG32954-RD | "-" | CG32954-RE | "-"
insitu CG3479 in-situ | osp | - | 55966 | -33572 | INTRAGENIC | intron:CG3479-RA:2 | CG3479-RA | "-"

*********************** Rank 438 [Score 16.888550]   GBROWSE*******************

 CG30085 in-situ | CG30085 | - | -16630 | -21471 | UPSTREAM | CG30085-RA | "-"
 CG8355 in-situ | sli | - | 5079 | -13104 | INTRAGENIC | intron:CG8355-RA:3 | intron:CG8355-RC:3 | intron:CG8355-RB:3 | CG8355-RA | "-" | CG8355-RC | "-" | CG8355-RB | "-"

*********************** Rank 439 [Score 16.887695]   GBROWSE*******************

 CG4215 in-situ | spel1 | + | -18526 | -14749 | DOWNSTREAM | CG4215-RB | "-" | CG4215-RA | "-"
insitu CG3478 in-situ | ppk | - | 722 | -2230 | INTRAGENIC | intron:CG3478-RA:2 | CG3478-RA | "-"

Polluted


*********************** Rank 440 [Score 16.883118]   GBROWSE*******************

insitu highlight CG4345 in-situ | grim | - | -31907 | -33602 | UPSTREAM | CG4345-RA | "-"
 CG4319 in-situ | rpr | - | 62082 | 61232 | DOWNSTREAM | CG4319-RA | "-"

*********************** Rank 441 [Score 16.869141]   GBROWSE*******************

 CG11282 in-situ | caps | + | -1064 | 48007 | INTRAGENIC | intron:CG11282-RA:1 | intron:CG11282-RB:1 | CG11282-RA | "-" | CG11282-RB | "-"
 CG32119 in-situ | CG32119 | - | 18949 | 17110 | DOWNSTREAM | CG32119-RA | "-"

*********************** Rank 442 [Score 16.860718]   GBROWSE*******************

 CG1976 in-situ | RhoGAP100F | + | -117685 | -105303 | DOWNSTREAM | CG1976-RA | "-"
insitu CG2003 in-situ | CG2003 | + | 27568 | 40354 | UPSTREAM | CG2003-RA | "-" | CG2003-RB | "-"

*********************** Rank 443 [Score 16.836914]   GBROWSE*******************

 CG33152 in-situ | hbn | - | -5947 | -12192 | UPSTREAM | CG33152-RA | "-"
 CG15649 in-situ | CG15649 | - | 7075 | 6363 | DOWNSTREAM | CG15649-RA | "-"


*********************** Rank 444 [Score 16.831299]   GBROWSE*******************

insitu highlight CG2102 in-situ | cas | - | -11931 | -16289 | UPSTREAM | CG2102-RA | "-" | CG2102-RB | "-"
 CG1239 in-situ | CG1239 | + | 4310 | 5471 | UPSTREAM | CG1239-RA | "-"

*********************** Rank 445 [Score 16.828369]   GBROWSE*******************

 CG10349 in-situ | CG10349 | + | -31835 | -26453 | DOWNSTREAM | CG10349-RA | "-" | CG10349-RB | "-"
 CG31270 in-situ | CG31270 | - | 12222 | 11106 | DOWNSTREAM | CG31270-RA | "-"


*********************** Rank 446 [Score 16.815430]   GBROWSE*******************

insitu CG32954 in-situ | CG32954 | + | -28843 | -25496 | DOWNSTREAM | CG32954-RA | "-" | CG32954-RB | "-" | CG32954-RC | "-" | CG32954-RG | "-" | CG32954-RH | "-" | CG32954-RF | "-" | CG32954-RD | "-" | CG32954-RE | "-"
insitu CG3479 in-situ | osp | - | 44916 | -44622 | INTRAGENIC | intron:CG3479-RA:1 | CG3479-RA | "-"


*********************** Rank 447 [Score 16.815430]   GBROWSE*******************

 CG2839 in-situ | CG2839 | + | -63122 | -60642 | DOWNSTREAM | CG2839-RA | "-"
 CG17941 in-situ | ds | - | 26183 | -48764 | INTRAGENIC | intron:CG17941-RA:2 | CG17941-RA | "-"


*********************** Rank 448 [Score 16.815430]   GBROWSE*******************

 CG11634 in-situ | CG11634 | + | -67163 | -66076 | DOWNSTREAM | CG11634-RA | "-"
 CG2528 in-situ | CG2528 | - | 9409 | 7307 | DOWNSTREAM | CG2528-RA | "-"


*********************** Rank 449 [Score 16.815430]   GBROWSE*******************

 CG14503 in-situ | CG14503 | + | -52088 | -51912 | DOWNSTREAM | CG14503-RA | "-"
 CG15066 in-situ | CG15066 | - | 19302 | 18766 | DOWNSTREAM | CG15066-RA | "-"


*********************** Rank 450 [Score 16.815430]   GBROWSE*******************

 CG17326 in-situ | CG17326 | - | -20091 | -22266 | UPSTREAM | CG17326-RA | "-"
 CG13235 in-situ | CG13235 | + | 69378 | 69545 | UPSTREAM | CG13235-RA | "-"


*********************** Rank 451 [Score 16.815430]   GBROWSE*******************

 CG12223 in-situ | Dsp1 | + | -8919 | -3638 | DOWNSTREAM | CG12223-RB | "-" | CG12223-RA | "-" | CG12223-RC | "-"
 CG4200 in-situ | sl | + | 7779 | 13957 | UPSTREAM | CG4200-RA | "-"


*********************** Rank 452 [Score 16.813477]   GBROWSE*******************

 CG8119 in-situ | CG8119 | + | -1151 | -417 | DOWNSTREAM | CG8119-RA | "-"
 CG15646 in-situ | CG15646 | - | 5558 | 4326 | DOWNSTREAM | CG15646-RA | "-"


*********************** Rank 453 [Score 16.809937]   GBROWSE*******************

 CG10677 in-situ | CG10677 | + | -47318 | -45390 | DOWNSTREAM | CG10677-RA | "-"
 CG4669 in-situ | CG4669 | + | 4237 | 6392 | UPSTREAM | CG4669-RA | "-"


*********************** Rank 454 [Score 16.809448]   GBROWSE*******************

 CG11219 in-situ | PIP82 | - | -24129 | -28137 | UPSTREAM | CG11219-RA | "-"
insitu CG12113 in-situ | CG12113 | - | 5107 | 1589 | DOWNSTREAM | CG12113-RA | "-"

*********************** Rank 455 [Score 16.802734]   GBROWSE*******************

 CG14846 in-situ | CG14846 | - | -6803 | -9063 | UPSTREAM | CG14846-RA | "-"
 CG14847 in-situ | CG14847 | - | 259 | -808 | INTRAGENIC | intron:CG14847-RA:1 | CG14847-RA | "-"

*********************** Rank 456 [Score 16.790527]   GBROWSE*******************

 CG1338 in-situ | CG1338 | - | -4594 | -9006 | UPSTREAM | CG1338-RA | "-" | CG1338-RB | "-"
insitu highlight CG1849 in-situ | run | + | 14606 | 17491 | UPSTREAM | CG1849-RA | "-"

note: overlaps known module run_stripe1 by 323 bases (module coords: 20352427-20354043)
note: overlaps known module run_stripe7 by 323 bases (module coords: 20352427-20358012)

*********************** Rank 457 [Score 16.772705]   GBROWSE*******************

 CG1210 in-situ | Pk61C | + | -10618 | 5235 | INTRAGENIC | intron:CG1210-RD:3 | intron:CG1210-RA:3 | intron:CG1210-RH:3 | intron:CG1210-RF:3 | intron:CG1210-RC:3 | CG1210-RD | "-" | CG1210-RA | "-" | CG1210-RH | "-" | CG1210-RF | "-" | CG1210-RC | "-" | CG1210-RG | "-" | CG1210-RB | "-" | CG1210-RE | "-"
 CG6845 in-situ | CG6845 | + | 6383 | 8989 | UPSTREAM | CG6845-RA | "-"

*********************** Rank 458 [Score 16.764221]   GBROWSE*******************

 CG12478 in-situ | bru-3 | - | -67633 | -196464 | UPSTREAM | CG12478-RA | "-" | CG12478-RB | "-"
 CG8757 in-situ | CG8757 | - | 99170 | 98204 | DOWNSTREAM | CG8757-RA | "-"

*********************** Rank 459 [Score 16.749634]   GBROWSE*******************

 CG9582 in-situ | CG9582 | - | -6815 | -7769 | UPSTREAM | CG9582-RA | "-"
 CG31708 in-situ | CG31708 | - | 38943 | 19405 | DOWNSTREAM | CG31708-RB | "-" | CG31708-RA | "-"

*********************** Rank 460 [Score 16.746460]   GBROWSE*******************

 CG31759 in-situ | CG31759 | + | -42502 | -40010 | DOWNSTREAM | CG31759-RB | "-" | CG31759-RA | "-"
 CG31862 in-situ | CG31862 | + | 88741 | 89289 | UPSTREAM | CG31862-RA | "-"


*********************** Rank 461 [Score 16.744385]   GBROWSE*******************

 CG4760 in-situ | bol | - | -738 | -29464 | UPSTREAM | CG4760-RB | "-" | CG4760-RC | "-" | CG4760-RD | "-" | CG4760-RA | "-"
insitu CG4665 in-situ | Dhpr | + | 3471 | 4823 | UPSTREAM | CG4665-RA | "-"


*********************** Rank 462 [Score 16.721741]   GBROWSE*******************

 CG12526 in-situ | Or67a | + | -10500 | -9085 | DOWNSTREAM | CG12526-RA | "-"
 CG12525 in-situ | CG12525 | - | 3578 | 1819 | DOWNSTREAM | CG12525-RA | "-"

*********************** Rank 463 [Score 16.716064]   GBROWSE*******************

insitu highlight CG10021 in-situ | bowl | + | -3625 | 8798 | INTRAGENIC | intron:CG10021-RB:2 | intron:CG10021-RC:2 | intron:CG10021-RD:2 | CG10021-RB | "-" | CG10021-RC | "-" | CG10021-RD | "-" | CG10021-RA | "-"
 CG31960 in-situ | CG31960 | + | 9615 | 10184 | UPSTREAM | CG31960-RA | "-"

*********************** Rank 464 [Score 16.702515]   GBROWSE*******************

 CG10001 in-situ | AR-2 | - | -2879 | -9903 | UPSTREAM | CG10001-RA | "-"
 CG10000 in-situ | CG10000 | - | 4579 | 1371 | DOWNSTREAM | CG10000-RA | "-"

*********************** Rank 465 [Score 16.698486]   GBROWSE*******************

insitu highlight CG4345 in-situ | grim | - | -61407 | -63102 | UPSTREAM | CG4345-RA | "-"
 CG4319 in-situ | rpr | - | 32582 | 31732 | DOWNSTREAM | CG4319-RA | "-"


*********************** Rank 466 [Score 16.697998]   GBROWSE*******************

insitu CG11387 in-situ | ct | + | -14325 | 52550 | INTRAGENIC | intron:CG11387-RA:1 | CG11387-RA | "-" | CG11387-RB | "-"
 CG12690 in-situ | CHES-1-like | - | 73221 | 61414 | DOWNSTREAM | CG12690-RA | "-"

*********************** Rank 467 [Score 16.696167]   GBROWSE*******************

 CG5156 in-situ | CG5156 | + | -6157 | -4663 | DOWNSTREAM | CG5156-RA | "-"
 CG5397 in-situ | CG5397 | + | 5904 | 8071 | UPSTREAM | CG5397-RA | "-"

*********************** Rank 468 [Score 16.671692]   GBROWSE*******************

 CG10543 in-situ | CG10543 | + | -10515 | -513 | DOWNSTREAM | CG10543-RB | "-" | CG10543-RA | "-" | CG10543-RC | "-"
insitu CG30291 in-situ | CG30291 | - | 2417 | 599 | DOWNSTREAM | CG30291-RA | "-"

*********************** Rank 469 [Score 16.666626]   GBROWSE*******************

 CG12014 in-situ | CG12014 | + | -36836 | -35037 | DOWNSTREAM | CG12014-RA | "-"
 CG1921 in-situ | sty | - | 9814 | -13984 | INTRAGENIC | intron:CG1921-RC:1 | intron:CG1921-RB:1 | CG1921-RC | "-" | CG1921-RB | "-"

*********************** Rank 470 [Score 16.662415]   GBROWSE*******************

 CG13934 in-situ | CG13934 | - | -8120 | -8901 | UPSTREAM | CG13934-RA | "-"
 CG13935 in-situ | CG13935 | - | 1840 | -685 | INTRAGENIC | intron:CG13935-RA:4 | CG13935-RA | "-"

*********************** Rank 471 [Score 16.661499]   GBROWSE*******************

 CG3690 in-situ | EG:BACR7A4.13 | - | -36215 | -38773 | UPSTREAM | CG3690-RA | "-"
 CG18823 in-situ | CG18823 | + | 2519 | 2839 | UPSTREAM | CG18823-RA | "-"

*********************** Rank 472 [Score 16.650757]   GBROWSE*******************

 CG1304 in-situ | CG1304 | - | -11868 | -12763 | UPSTREAM | CG1304-RA | "-"
 CG11666 in-situ | CG11666 | + | 7437 | 7727 | UPSTREAM | CG11666-RA | "-"

*********************** Rank 473 [Score 16.648682]   GBROWSE*******************

 CG15296 in-situ | CG15296 | - | -1991 | -2655 | UPSTREAM | CG15296-RA | "-"
 CG32674 in-situ | CG32674 | - | 7687 | 6896 | DOWNSTREAM | CG32674-RA | "-"


*********************** Rank 474 [Score 16.638550]   GBROWSE*******************

 CG1324 in-situ | CG1324 | - | -25 | -1241 | UPSTREAM | CG1324-RA | "-"
 CG15452 in-situ | CG15452 | - | 9964 | 9086 | DOWNSTREAM | CG15452-RA | "-"


*********************** Rank 475 [Score 16.634155]   GBROWSE*******************

 CG9695 in-situ | Dab | + | -4128 | 6719 | INTRAGENIC | intron:CG9695-RA:4 | CG9695-RA | "-"
 CG13031 in-situ | CG13031 | + | 7621 | 8044 | UPSTREAM | CG13031-RA | "-"


*********************** Rank 476 [Score 16.628174]   GBROWSE*******************

 CG11281 in-situ | CG11281 | + | -14971 | -11956 | DOWNSTREAM | CG11281-RA | "-"
 CG11282 in-situ | caps | + | 40036 | 89107 | UPSTREAM | CG11282-RA | "-" | CG11282-RB | "-"

*********************** Rank 477 [Score 16.626038]   GBROWSE*******************

insitu highlight CG7250 in-situ | Toll-6 | + | -20721 | -15210 | DOWNSTREAM | CG7250-RA | "-"
 CG7804 in-situ | CG7804 | - | 116689 | 114776 | DOWNSTREAM | CG7804-RA | "-"

*********************** Rank 478 [Score 16.622314]   GBROWSE*******************

 CG31085 in-situ | CG31085 | + | -23608 | -10524 | DOWNSTREAM | CG31085-RB | "-" | CG31085-RA | "-"
 CG14239 in-situ | CG14239 | - | 30275 | 29396 | DOWNSTREAM | CG14239-RA | "-"

*********************** Rank 479 [Score 16.620422]   GBROWSE*******************

 CG8715 in-situ | CG8715 | + | -1822 | 9022 | INTRAGENIC | intron:CG8715-RA:1 | intron:CG8715-RB:1 | CG8715-RA | "-" | CG8715-RB | "-"
 CG12770 in-situ | l(2)k16503 | - | 10232 | 9415 | DOWNSTREAM | CG12770-RA | "-"

*********************** Rank 480 [Score 16.619507]   GBROWSE*******************

 CG17681 in-situ | CG17681 | + | -9188 | 2693 | INTRAGENIC | intron:CG17681-RA:1 | CG17681-RA | "-" | CG17681-RB | "-"
 CG15154 in-situ | Socs36E | - | 693 | -13049 | INTRAGENIC | intron:CG15154-RA:1 | intron:CG15154-RB:1 | CG15154-RA | "-" | CG15154-RB | "-"

*********************** Rank 481 [Score 16.614136]   GBROWSE*******************

 CG7554 in-situ | comm2 | - | -14372 | -16630 | UPSTREAM | CG7554-RA | "-"
insitu highlight CG17943 in-situ | comm | - | 14202 | 8103 | DOWNSTREAM | CG17943-RA | "-"


*********************** Rank 482 [Score 16.612305]   GBROWSE*******************

 CG5308 in-situ | CG5308 | + | -8676 | -2705 | DOWNSTREAM | CG5308-RA | "-" | CG5308-RB | "-"
 CG12593 in-situ | CG12593 | - | 28235 | 25840 | DOWNSTREAM | CG12593-RA | "-"

*********************** Rank 483 [Score 16.604004]   GBROWSE*******************

 CG3837 in-situ | CG3837 | + | -16230 | -12605 | DOWNSTREAM | CG3837-RA | "-"
 CG14861 in-situ | CG14861 | + | 79860 | 81127 | UPSTREAM | CG14861-RA | "-"

*********************** Rank 484 [Score 16.598511]   GBROWSE*******************

 CG6269 in-situ | unc-4 | + | -3808 | 7692 | INTRAGENIC | intron:CG6269-RA:1 | CG6269-RA | "-"
 CG6352 in-situ | OdsH | + | 17859 | 40797 | UPSTREAM | CG6352-RA | "-"

*********************** Rank 485 [Score 16.591003]   GBROWSE*******************

 CG8594 in-situ | CG8594 | + | -6275 | -3079 | DOWNSTREAM | CG8594-RA | "-"
 CG30055 in-situ | CG30055 | + | 10916 | 12324 | UPSTREAM | CG30055-RA | "-"

*********************** Rank 486 [Score 16.587524]   GBROWSE*******************

 CG9885 in-situ | dpp | + | -50629 | -19474 | DOWNSTREAM | CG9885-RA | "-" | CG9885-RB | "-" | CG9885-RC | "-" | CG9885-RD | "-" | CG9885-RE | "-"
 CG15393 in-situ | CG15393 | - | 11036 | 10671 | DOWNSTREAM | CG15393-RA | "-"

*********************** Rank 487 [Score 16.575562]   GBROWSE*******************

 CG31647 in-situ | CG31647 | - | -3133 | -16756 | UPSTREAM | CG31647-RA | "-" | CG31647-RB | "-"
insitu highlight CG6634 in-situ | CG6634 | + | 9306 | 15274 | UPSTREAM | CG6634-RA | "-"

*********************** Rank 488 [Score 16.573730]   GBROWSE*******************

insitu CG31264 in-situ | CG31264 | - | -20627 | -39445 | UPSTREAM | CG31264-RA | "-"
 CG11732 in-situ | Obp85a | - | 6172 | 1209 | DOWNSTREAM | CG11732-RA | "-"

*********************** Rank 489 [Score 16.571777]   GBROWSE*******************

 CG17278 in-situ | BcDNA:SD04019 | - | -498 | -3627 | UPSTREAM | CG17278-RA | "-"
 CG3723 in-situ | Dhc93AB | - | 17758 | 358 | DOWNSTREAM | CG3723-RA | "-"

*********************** Rank 490 [Score 16.552490]   GBROWSE*******************

 CG31394 in-situ | CG31394 | - | -8804 | -9542 | UPSTREAM | CG31394-RA | "-"
insitu highlight CG17117 in-situ | hth | - | 100704 | -28120 | INTRAGENIC | intron:CG17117-RB:7 | intron:CG17117-RC:8 | intron:CG17117-RA:6 | CG17117-RD | "-" | CG17117-RB | "-" | CG17117-RC | "-" | CG17117-RA | "-"

*********************** Rank 491 [Score 16.551392]   GBROWSE*******************

 CG4427 in-situ | EP2237 | - | -9653 | -12898 | UPSTREAM | CG4427-RA | "-" | CG4427-RB | "-"
insitu highlight CG2762 in-situ | ush | + | 34132 | 51206 | UPSTREAM | CG2762-RA | "-"

*********************** Rank 492 [Score 16.540039]   GBROWSE*******************

 CG15150 in-situ | CG15150 | - | -19008 | -20307 | UPSTREAM | CG15150-RA | "-"
insitu CG15151 in-situ | PFE | + | 21244 | 40029 | UPSTREAM | CG15151-RA | "-"

*********************** Rank 493 [Score 16.534790]   GBROWSE*******************

 CG1999 in-situ | CG1999 | + | -15727 | -14455 | DOWNSTREAM | CG1999-RA | "-"
 CG15033 in-situ | CG15033 | + | 3740 | 4276 | UPSTREAM | CG15033-RA | "-"


*********************** Rank 494 [Score 16.531982]   GBROWSE*******************

 CG7398 in-situ | Trn | + | -5637 | -116 | DOWNSTREAM | CG7398-RA | "-" | CG7398-RB | "-" | CG7398-RC | "-"
 CG8219 in-situ | CG8219 | + | 1352 | 4655 | UPSTREAM | CG8219-RA | "-"


*********************** Rank 495 [Score 16.531494]   GBROWSE*******************

 CG32387 in-situ | CG32387 | - | -24419 | -45034 | UPSTREAM | CG32387-RA | "-" | CG32387-RB | "-"
 CG14826 in-situ | CG14826 | + | 26463 | 27251 | UPSTREAM | CG14826-RA | "-"

*********************** Rank 496 [Score 16.530640]   GBROWSE*******************

 CG32450 in-situ | CG32450 | + | -71994 | -71095 | DOWNSTREAM | CG32450-RA | "-"
insitu highlight CG5723 in-situ | Ten-m | - | 6115 | -108740 | INTRAGENIC | intron:CG5723-RB:1 | CG5723-RB | "-"

*********************** Rank 497 [Score 16.528320]   GBROWSE*******************

 CG6026 in-situ | CG6026 | + | -48860 | -44168 | DOWNSTREAM | CG6026-RA | "-"
 CG6027 in-situ | cdi | - | 5943 | -43529 | INTRAGENIC | intron:CG6027-RA:1 | CG6027-RA | "-"

*********************** Rank 498 [Score 16.523560]   GBROWSE*******************

 CG32655 in-situ | CG32655 | - | -69011 | -70069 | UPSTREAM | CG32655-RA | "-"
 CG2577 in-situ | CG2577 | + | 68471 | 69711 | UPSTREAM | CG2577-RA | "-"

*********************** Rank 499 [Score 16.516724]   GBROWSE*******************

 CG7527 in-situ | CadN2 | - | -95813 | -121812 | UPSTREAM | CG7527-RA | "-"
 CG5674 in-situ | CG5674 | + | 54921 | 66453 | UPSTREAM | CG5674-RA | "-" | CG5674-RB | "-" | CG5674-RC | "-"

*********************** Rank 500 [Score 16.513672]   GBROWSE*******************

 CG9885 in-situ | dpp | + | -28779 | 2376 | INTRAGENIC | intron:CG9885-RA:2 | intron:CG9885-RB:2 | intron:CG9885-RC:2 | intron:CG9885-RD:2 | intron:CG9885-RE:2 | CG9885-RA | "-" | CG9885-RB | "-" | CG9885-RC | "-" | CG9885-RD | "-" | CG9885-RE | "-"
 CG15393 in-situ | CG15393 | - | 32886 | 32521 | DOWNSTREAM | CG15393-RA | "-"

*********************** Rank 501 [Score 16.512146]   GBROWSE*******************

 CG4659 in-situ | Srp54k | + | -7222 | -5012 | DOWNSTREAM | CG4659-RA | "-"
 CG10677 in-situ | CG10677 | + | 23132 | 25060 | UPSTREAM | CG10677-RA | "-"

*********************** Rank 502 [Score 16.489380]   GBROWSE*******************

 CG14678 in-situ | CG14678 | - | -32473 | -35274 | UPSTREAM | CG14678-RA | "-"
 CG11373 in-situ | CG11373 | - | 41054 | 40392 | DOWNSTREAM | CG11373-RA | "-"

*********************** Rank 503 [Score 16.485840]   GBROWSE*******************

 CG3333 in-situ | Nop60B | + | -2907 | -502 | DOWNSTREAM | CG3333-RA | "-"
 CG3363 in-situ | CG3363 | + | 10725 | 17660 | UPSTREAM | CG3363-RA | "-"

*********************** Rank 504 [Score 16.483948]   GBROWSE*******************

 CG7573 in-situ | CG7573 | + | -10114 | -5271 | DOWNSTREAM | CG7573-RA | "-" | CG7573-RB | "-"
 CG6168 in-situ | CG6168 | - | 3051 | 2023 | DOWNSTREAM | CG6168-RB | "-"

*********************** Rank 505 [Score 16.457031]   GBROWSE*******************

 CG12732 in-situ | CG12732 | + | -38858 | -38478 | DOWNSTREAM | CG12732-RA | "-"
insitu highlight CG3252 in-situ | CG3252 | - | 13659 | 8328 | DOWNSTREAM | CG3252-RA | "-"


*********************** Rank 506 [Score 16.454834]   GBROWSE*******************

insitu highlight CG9908 in-situ | disco | - | -4447 | -10583 | UPSTREAM | CG9908-RA | "-"
 CG12507 in-situ | CG12507 | - | 45283 | 44199 | DOWNSTREAM | CG12507-RA | "-"

*********************** Rank 507 [Score 16.449463]   GBROWSE*******************

insitu CG10710 in-situ | CG10710 | - | -128981 | -132309 | UPSTREAM | CG10710-RA | "-"
 CG12478 in-situ | bru-3 | - | 18167 | -110664 | INTRAGENIC | intron:CG12478-RA:3 | intron:CG12478-RB:2 | CG12478-RA | "-" | CG12478-RB | "-"

*********************** Rank 508 [Score 16.446594]   GBROWSE*******************

 CG6349 in-situ | DNApol-alpha180 | - | -12914 | -18111 | UPSTREAM | CG6349-RA | "-"
 CG31176 in-situ | CG31176 | - | 34994 | -12368 | INTRAGENIC | intron:CG31176-RA:1 | CG31176-RA | "-"

*********************** Rank 509 [Score 16.440796]   GBROWSE*******************

 CG3647 in-situ | stc | + | -35283 | -30695 | DOWNSTREAM | CG3647-RB | "-" | CG3647-RA | "-"
 CG4168 in-situ | BG:DS03192.2 | - | 29555 | 13342 | DOWNSTREAM | CG4168-RA | "-"

*********************** Rank 510 [Score 16.440369]   GBROWSE*******************

 CG11279 in-situ | CG11279 | + | -9829 | -9330 | DOWNSTREAM | CG11279-RA | "-"
 CG14115 in-situ | CG14115 | - | 4541 | 3669 | DOWNSTREAM | CG14115-RA | "-"

*********************** Rank 511 [Score 16.424561]   GBROWSE*******************

 CG1151 in-situ | BcDNA:LD21503 | + | -5919 | -3747 | DOWNSTREAM | CG1151-RA | "-"
insitu CG1153 in-situ | CG1153 | + | 8364 | 9445 | UPSTREAM | CG1153-RA | "-"

*********************** Rank 512 [Score 16.423584]   GBROWSE*******************

 CG8086 in-situ | CG8086 | - | -16767 | -29724 | UPSTREAM | CG8086-RA | "-" | CG8086-RB | "-"
insitu highlight CG8049 in-situ | Btk29A | - | 26803 | -14765 | INTRAGENIC | intron:CG8049-RB:5 | intron:CG8049-RD:5 | intron:CG8049-RC:1 | intron:CG8049-RA:1 | CG8049-RB | "-" | CG8049-RD | "-" | CG8049-RC | "-" | CG8049-RA | "-"

*********************** Rank 513 [Score 16.420654]   GBROWSE*******************

insitu highlight CG2047 in-situ | ftz | + | -25353 | -23449 | DOWNSTREAM | CG2047-RA | "-"
 CG31488 in-situ | CG31488 | + | 68057 | 68686 | UPSTREAM | CG31488-RA | "-"

*********************** Rank 514 [Score 16.406616]   GBROWSE*******************

insitu CG11453 in-situ | CG11453 | + | -30438 | -28431 | DOWNSTREAM | CG11453-RA | "-"
insitu CG4608 in-situ | bnl | - | 22856 | -19888 | INTRAGENIC | intron:CG4608-RA:2 | intron:CG4608-RB:2 | CG4608-RA | "-" | CG4608-RB | "-"

*********************** Rank 515 [Score 16.406494]   GBROWSE*******************

 CG13353 in-situ | CG13353 | + | -4910 | -3559 | DOWNSTREAM | CG13353-RA | "-"
 CG30483 in-situ | Prosap | - | 45669 | -33807 | INTRAGENIC | intron:CG30483-RA:2 | CG30483-RA | "-"


*********************** Rank 516 [Score 16.405762]   GBROWSE*******************

 CG3758 in-situ | esg | + | -5151 | -2865 | DOWNSTREAM | CG3758-RA | "-"
 CG15258 in-situ | CG15258 | - | 22573 | 21983 | DOWNSTREAM | CG15258-RA | "-"


*********************** Rank 517 [Score 16.399536]   GBROWSE*******************

 CG8853 in-situ | CG8853 | + | -7928 | -6305 | DOWNSTREAM | CG8853-RA | "-"
insitu highlight CG10016 in-situ | drm | + | 420 | 9255 | UPSTREAM | CG10016-RB | "-" | CG10016-RA | "-"

*********************** Rank 518 [Score 16.392456]   GBROWSE*******************

 CG33152 in-situ | hbn | - | -697 | -6942 | UPSTREAM | CG33152-RA | "-"
 CG15649 in-situ | CG15649 | - | 12325 | 11613 | DOWNSTREAM | CG15649-RA | "-"

*********************** Rank 519 [Score 16.388062]   GBROWSE*******************

 CG7847 in-situ | sr | + | -51810 | -8864 | DOWNSTREAM | CG7847-RA | "-" | CG7847-RB | "-"
 CG14316 in-situ | CG14316 | + | 9507 | 11029 | UPSTREAM | CG14316-RA | "-"

*********************** Rank 520 [Score 16.386353]   GBROWSE*******************

 CG6559 in-situ | CG6559 | - | -30308 | -46546 | UPSTREAM | CG6559-RA | "-"
 CG12362 in-situ | CG12362 | + | 69795 | 71756 | UPSTREAM | CG12362-RB | "-" | CG12362-RA | "-"

*********************** Rank 521 [Score 16.385986]   GBROWSE*******************

 CG12110 in-situ | Pld | + | -91061 | -77819 | DOWNSTREAM | CG12110-RB | "-" | CG12110-RC | "-" | CG12110-RA | "-" | CG12110-RE | "-" | CG12110-RD | "-"
 CG9397 in-situ | 1.28 | + | 92120 | 93070 | UPSTREAM | CG9397-RA | "-"

*********************** Rank 522 [Score 16.382324]   GBROWSE*******************

 CG12730 in-situ | CG12730 | + | -10375 | -8866 | DOWNSTREAM | CG12730-RA | "-"
 CG15783 in-situ | CG15783 | - | 2269 | 1266 | DOWNSTREAM | CG15783-RA | "-"

*********************** Rank 523 [Score 16.368652]   GBROWSE*******************

 CG18241 in-situ | Toll-4 | + | -7612 | -2279 | DOWNSTREAM | CG18241-RA | "-"
 CG31609 in-situ | CG31609 | + | 5908 | 6819 | UPSTREAM | CG31609-RA | "-"

*********************** Rank 524 [Score 16.357300]   GBROWSE*******************

 CG14886 in-situ | CG14886 | + | -789 | 2912 | INTRAGENIC | intron:CG14886-RA:1 | CG14886-RA | "-"
 CG14887 in-situ | Dhfr | + | 3074 | 3719 | UPSTREAM | CG14887-RA | "-"

*********************** Rank 525 [Score 16.356201]   GBROWSE*******************

 CG31662 in-situ | Gr22a | - | -3918 | -5167 | UPSTREAM | CG31662-RA | "-"
 CG31933 in-situ | CG31933 | - | 24496 | 22435 | DOWNSTREAM | CG31933-RA | "-"

*********************** Rank 526 [Score 16.352600]   GBROWSE*******************

 CG2839 in-situ | CG2839 | + | -53072 | -50592 | DOWNSTREAM | CG2839-RA | "-"
 CG17941 in-situ | ds | - | 36233 | -38714 | INTRAGENIC | intron:CG17941-RA:2 | CG17941-RA | "-"

*********************** Rank 527 [Score 16.340454]   GBROWSE*******************

 CG14061 in-situ | CG14061 | - | -8428 | -9528 | UPSTREAM | CG14061-RA | "-"
 CG12558 in-situ | CG12558 | + | 23860 | 24799 | UPSTREAM | CG12558-RA | "-"

*********************** Rank 528 [Score 16.340454]   GBROWSE*******************

insitu CG12701 in-situ | CG12701 | - | -17162 | -24103 | UPSTREAM | CG12701-RB | "-" | CG12701-RA | "-"
 CG12700 in-situ | skpD | + | 12639 | 13298 | UPSTREAM | CG12700-RA | "-"

*********************** Rank 529 [Score 16.339111]   GBROWSE*******************

insitu CG32096 in-situ | rols | - | -14694 | -71338 | UPSTREAM | CG32096-RB | "-" | CG32096-RD | "-" | CG32096-RE | "-" | CG32096-RA | "-" | CG32096-RC | "-"
insitu highlight CG5661 in-situ | Sema-5c | - | 2232 | -12042 | INTRAGENIC | intron:CG5661-RA:3 | CG5661-RA | "-"

*********************** Rank 530 [Score 16.335938]   GBROWSE*******************

insitu CG32194 in-situ | CG32194 | - | -16313 | -18067 | UPSTREAM | CG32194-RB | "-"
 CG13698 in-situ | CG13698 | - | 2759 | -14262 | INTRAGENIC | intron:CG13698-RB:1 | CG13698-RB | "-"

*********************** Rank 531 [Score 16.317261]   GBROWSE*******************

 CG18405 in-situ | Sema-1a | + | -77123 | 26510 | INTRAGENIC | intron:CG18405-RA:3 | CG18405-RA | "-"
 CG9280 in-situ | Glt | + | 31124 | 35892 | UPSTREAM | CG9280-RC | "-" | CG9280-RB | "-" | CG9280-RA | "-"

*********************** Rank 532 [Score 16.307617]   GBROWSE*******************

insitu highlight CG1030 in-situ | Scr | - | -9208 | -34707 | UPSTREAM | CG1030-RA | "-"
insitu highlight CG2047 in-situ | ftz | + | 6497 | 8401 | UPSTREAM | CG2047-RA | "-"


note: overlaps known module ftz_distal by 419 bases (module coords: 2683631-2685133)
note: overlaps known module ftz_distal_enhancer by 90 bases (module coords: 2683960-2685131)
note: overlaps known module ftz_ps4_activator by 413 bases (module coords: 2683630-2684042)
note: overlaps known module ftz_rescue_construct by 420 bases (module coords: 2683630-2694036)
note: overlaps known module ftz_upstream_1985 by 420 bases (module coords: 2683630-2686200)

*********************** Rank 533 [Score 16.307007]   GBROWSE*******************

 CG32577 in-situ | disco-r | - | -8487 | -13378 | UPSTREAM | CG32577-RA | "-"
insitu highlight CG9908 in-situ | disco | - | 85053 | 78917 | DOWNSTREAM | CG9908-RA | "-"

*********************** Rank 534 [Score 16.301636]   GBROWSE*******************

 CG12425 in-situ | CG12425 | + | -3288 | -1733 | DOWNSTREAM | CG12425-RA | "-"
 CG4787 in-situ | CG4787 | + | 84987 | 87273 | UPSTREAM | CG4787-RA | "-"


*********************** Rank 535 [Score 16.294312]   GBROWSE*******************

insitu CG31216 in-situ | CG31216 | + | -16550 | 10539 | INTRAGENIC | intron:CG31216-RA:2 | CG31216-RA | "-"
 CG7535 in-situ | GluClalpha | + | 42948 | 87434 | UPSTREAM | CG7535-RA | "-" | CG7535-RB | "-"

*********************** Rank 536 [Score 16.292969]   GBROWSE*******************

 CG32390 in-situ | CG32390 | - | -6059 | -6645 | UPSTREAM | CG32390-RA | "-"
 CG32386 in-situ | corn | - | 6652 | -5897 | INTRAGENIC | intron:CG32386-RA:1 | CG32386-RA | "-"

*********************** Rank 537 [Score 16.288818]   GBROWSE*******************

insitu highlight CG10325 in-situ | abd-A | - | -48462 | -70888 | UPSTREAM | CG10325-RA | "-" | CG10325-RB | "-"
 CG10349 in-situ | CG10349 | + | 1215 | 6597 | UPSTREAM | CG10349-RA | "-" | CG10349-RB | "-"

*********************** Rank 538 [Score 16.283936]   GBROWSE*******************

insitu highlight CG10052 in-situ | Rx | + | -8626 | 11626 | INTRAGENIC | intron:CG10052-RA:4 | CG10052-RA | "-"
insitu CG10067 in-situ | Act57B | + | 18622 | 21034 | UPSTREAM | CG10067-RA | "-"

*********************** Rank 539 [Score 16.276245]   GBROWSE*******************

insitu CG32139 in-situ | Sox21b | - | -26846 | -45787 | UPSTREAM | CG32139-RA | "-"
insitu highlight CG5893 in-situ | D | - | 20450 | 17431 | DOWNSTREAM | CG5893-RA | "-"

*********************** Rank 540 [Score 16.271484]   GBROWSE*******************

 CG6414 in-situ | CG6414 | - | -15936 | -18286 | UPSTREAM | CG6414-RA | "-"
 CG32790 in-situ | CG32790 | + | 90632 | 91930 | UPSTREAM | CG32790-RA | "-"

*********************** Rank 541 [Score 16.270508]   GBROWSE*******************

 CG17150 in-situ | CG17150 | + | -12049 | 21782 | INTRAGENIC | intron:CG17150-RA:20 | CG17150-RA | "-" | CG17150-RB | "-" | CG17150-RC | "-"
 CG13705 in-situ | CG13705 | + | 23349 | 24961 | UPSTREAM | CG13705-RA | "-"

*********************** Rank 542 [Score 16.258301]   GBROWSE*******************

 CG7886 in-situ | CG7886 | - | -8847 | -24737 | UPSTREAM | CG7886-RA | "-"
 CG7832 in-situ | BEST:LD14744 | - | 4291 | -6741 | INTRAGENIC | intron:CG7832-RA:1 | CG7832-RA | "-"

*********************** Rank 543 [Score 16.250977]   GBROWSE*******************

 CG31644 in-situ | CG31644 | - | -18792 | -19293 | UPSTREAM | CG31644-RA | "-"
 CG14004 in-situ | CG14004 | + | 8167 | 8775 | UPSTREAM | CG14004-RA | "-"

*********************** Rank 544 [Score 16.245850]   GBROWSE*******************

 CG7835 in-situ | CG7835 | - | -2401 | -3053 | UPSTREAM | CG7835-RA | "-"
 CG12985 in-situ | CG12985 | + | 6367 | 7643 | UPSTREAM | CG12985-RA | "-"


*********************** Rank 545 [Score 16.237427]   GBROWSE*******************

 CG14390 in-situ | beat-Vc | - | -32658 | -40744 | UPSTREAM | CG14390-RA | "-"
 CG31345 in-situ | CG31345 | - | 29670 | 23986 | DOWNSTREAM | CG31345-RA | "-"

*********************** Rank 546 [Score 16.234253]   GBROWSE*******************

 CG13785 in-situ | CG13785 | + | -18286 | -17389 | DOWNSTREAM | CG13785-RA | "-"
insitu highlight CG4889 in-situ | wg | + | 6342 | 15436 | UPSTREAM | CG4889-RA | "-" | CG4889-RB | "-"


*********************** Rank 547 [Score 16.234009]   GBROWSE*******************

 CG12682 in-situ | CG12682 | + | -4833 | -4120 | DOWNSTREAM | CG12682-RA | "-"
 CG12681 in-situ | CG12681 | + | 44845 | 46308 | UPSTREAM | CG12681-RA | "-"


*********************** Rank 548 [Score 16.231934]   GBROWSE*******************

insitu CG13701 in-situ | skl | - | -11134 | -12515 | UPSTREAM | CG13701-RA | "-"
 CG32196 in-situ | CG32196 | - | 9245 | 7890 | DOWNSTREAM | CG32196-RA | "-"


*********************** Rank 549 [Score 16.218201]   GBROWSE*******************

insitu CG3619 in-situ | Dl | - | -31748 | -55230 | UPSTREAM | CG3619-RA | "-" | CG3619-RB | "-"
 CG3581 in-situ | CG3581 | - | 16788 | 15796 | DOWNSTREAM | CG3581-RA | "-"


*********************** Rank 550 [Score 16.215088]   GBROWSE*******************

 CG6628 in-situ | CG6628 | - | -11246 | -12203 | UPSTREAM | CG6628-RA | "-"
 CG32057 in-situ | CG32057 | - | 7834 | -30818 | INTRAGENIC | intron:CG32057-RA:2 | intron:CG32057-RB:2 | intron:CG32057-RC:2 | CG32057-RA | "-" | CG32057-RB | "-" | CG32057-RC | "-"

*********************** Rank 551 [Score 16.213745]   GBROWSE*******************

 CG5290 in-situ | CG5290 | - | -27264 | -30342 | UPSTREAM | CG5290-RA | "-"
 CG32193 in-situ | CG32193 | + | 21839 | 25859 | UPSTREAM | CG32193-RA | "-"

*********************** Rank 552 [Score 16.203918]   GBROWSE*******************

 CG18405 in-situ | Sema-1a | + | -106373 | -2740 | DOWNSTREAM | CG18405-RA | "-"
 CG9280 in-situ | Glt | + | 1874 | 6642 | UPSTREAM | CG9280-RC | "-" | CG9280-RB | "-" | CG9280-RA | "-"

*********************** Rank 553 [Score 16.196899]   GBROWSE*******************

 CG5890 in-situ | CG5890 | + | -8059 | -5697 | DOWNSTREAM | CG5890-RA | "-"
 CG6120 in-situ | Tsp96F | - | 6077 | -1829 | INTRAGENIC | intron:CG6120-RA:4 | CG6120-RA | "-"


*********************** Rank 554 [Score 16.195068]   GBROWSE*******************

 CG15283 in-situ | BG:DS08340.1 | - | -38382 | -41821 | UPSTREAM | CG15283-RA | "-"
insitu highlight CG4491 in-situ | noc | + | 1664 | 4821 | UPSTREAM | CG4491-RA | "-"

*********************** Rank 555 [Score 16.186768]   GBROWSE*******************

 CG18404 in-situ | CG18404 | + | -14930 | -14014 | DOWNSTREAM | CG18404-RA | "-"
 CG15532 in-situ | hdc | + | 9822 | 94057 | UPSTREAM | CG15532-RA | "-" | CG15532-RC | "-" | CG15532-RB | "-"

*********************** Rank 556 [Score 16.179199]   GBROWSE*******************

 CG7449 in-situ | hbs | + | -6926 | 23071 | INTRAGENIC | intron:CG7449-RA:1 | intron:CG7449-RB:1 | CG7449-RA | "-" | CG7449-RB | "-"
 CG30473 in-situ | Obp51a | - | 6515 | 6095 | DOWNSTREAM | CG30473-RA | "-"


*********************** Rank 557 [Score 16.162231]   GBROWSE*******************

insitu highlight CG18024 in-situ | SoxN | + | -22194 | -18149 | DOWNSTREAM | CG18024-RA | "-"
 CG32986 in-situ | CG32986 | + | 14988 | 15860 | UPSTREAM | CG32986-RA | "-"

*********************** Rank 558 [Score 16.162109]   GBROWSE*******************

 CG31834 in-situ | BG:DS00929.16 | + | -1815 | 3510 | INTRAGENIC | intron:CG31834-RA:1 | CG31834-RA | "-"
 CG10846 in-situ | dynactin-subunit-p25 | + | 9078 | 9949 | UPSTREAM | CG10846-RA | "-"

*********************** Rank 559 [Score 16.145264]   GBROWSE*******************

 CG14458 in-situ | CG14458 | + | -17196 | -13784 | DOWNSTREAM | CG14458-RA | "-"
 CG14457 in-situ | CG14457 | + | 6184 | 16262 | UPSTREAM | CG14457-RA | "-"

*********************** Rank 560 [Score 16.139404]   GBROWSE*******************

 CG32975 in-situ | nAcRalpha-34E | + | -7250 | 20762 | INTRAGENIC | intron:CG32975-RA:2 | CG32975-RA | "-"
 CG32973 in-situ | CG32973 | - | 32391 | 31407 | DOWNSTREAM | CG32973-RA | "-"

*********************** Rank 561 [Score 16.131836]   GBROWSE*******************

 CG13479 in-situ | CG13479 | - | -13390 | -13739 | UPSTREAM | CG13479-RA | "-"
insitu highlight CG13475 in-situ | HGTX | - | 18048 | 1695 | DOWNSTREAM | CG13475-RA | "-"

*********************** Rank 562 [Score 16.130737]   GBROWSE*******************

 CG11368 in-situ | CG11368 | + | -43615 | -43160 | DOWNSTREAM | CG11368-RA | "-"
 CG32719 in-situ | CG32719 | - | 17020 | 13068 | DOWNSTREAM | CG32719-RA | "-"


*********************** Rank 563 [Score 16.124878]   GBROWSE*******************

 CG32119 in-situ | CG32119 | - | -33151 | -34990 | UPSTREAM | CG32119-RA | "-"
 CG17673 in-situ | Acp70A | + | 19803 | 20090 | UPSTREAM | CG17673-RA | "-"

*********************** Rank 564 [Score 16.121582]   GBROWSE*******************

 CG31685 in-situ | CG31685 | - | -109735 | -110614 | UPSTREAM | CG31685-RA | "-"
 CG12617 in-situ | CG12617 | + | 14598 | 15244 | UPSTREAM | CG12617-RA | "-"

*********************** Rank 565 [Score 16.114746]   GBROWSE*******************

insitu highlight CG4761 in-situ | knrl | - | -2376 | -25771 | UPSTREAM | CG4761-RA | "-"
 CG13251 in-situ | CG13251 | + | 14318 | 17883 | UPSTREAM | CG13251-RA | "-"

*********************** Rank 566 [Score 16.113281]   GBROWSE*******************

 CG1887 in-situ | CG1887 | - | -18216 | -21991 | UPSTREAM | CG1887-RA | "-"
 CG13925 in-situ | CG13925 | + | 5544 | 6230 | UPSTREAM | CG13925-RA | "-"

*********************** Rank 567 [Score 16.113037]   GBROWSE*******************

 CG31759 in-situ | CG31759 | + | -21902 | -19410 | DOWNSTREAM | CG31759-RB | "-" | CG31759-RA | "-"
 CG31862 in-situ | CG31862 | + | 109341 | 109889 | UPSTREAM | CG31862-RA | "-"


*********************** Rank 568 [Score 16.110474]   GBROWSE*******************

 CG5481 in-situ | lea | - | -5035 | -44596 | UPSTREAM | CG5481-RA | "-"
 CG31925 in-situ | CG31925 | - | 48559 | 47866 | DOWNSTREAM | CG31925-RA | "-"


*********************** Rank 569 [Score 16.110107]   GBROWSE*******************

 CG6043 in-situ | CG6043 | + | -2420 | 20556 | INTRAGENIC | intron:CG6043-RB:2 | intron:CG6043-RC:1 | intron:CG6043-RD:2 | intron:CG6043-RA:1 | CG6043-RB | "-" | CG6043-RC | "-" | CG6043-RD | "-" | CG6043-RA | "-"
 CG31848 in-situ | CG31848 | - | 2235 | 1651 | DOWNSTREAM | CG31848-RA | "-"

*********************** Rank 570 [Score 16.109619]   GBROWSE*******************

 CG31140 in-situ | CG31140 | + | -13287 | 6779 | INTRAGENIC | intron:CG31140-RA:10 | CG31140-RA | "-"
 CG31451 in-situ | CG31451 | + | 6875 | 8099 | UPSTREAM | CG31451-RA | "-"

*********************** Rank 571 [Score 16.109131]   GBROWSE*******************

insitu highlight CG17117 in-situ | hth | - | -20746 | -149570 | UPSTREAM | CG17117-RD | "-" | CG17117-RB | "-" | CG17117-RC | "-" | CG17117-RA | "-"
insitu CG6465 in-situ | CG6465 | - | 17760 | 16149 | DOWNSTREAM | CG6465-RA | "-"

*********************** Rank 572 [Score 16.106445]   GBROWSE*******************

insitu CG8581 in-situ | fra | + | -24281 | 10849 | INTRAGENIC | intron:CG8581-RB:1 | intron:CG8581-RA:1 | CG8581-RB | "-" | CG8581-RA | "-"
 CG30056 in-situ | CG30056 | + | 8748 | 9324 | UPSTREAM | CG30056-RA | "-"


*********************** Rank 573 [Score 16.103210]   GBROWSE*******************

 CG1800 in-situ | CG1800 | + | -31635 | -28137 | DOWNSTREAM | CG1800-RA | "-"
 CG1804 in-situ | CG1804 | + | 9306 | 11997 | UPSTREAM | CG1804-RA | "-"

*********************** Rank 574 [Score 16.093018]   GBROWSE*******************

insitu highlight CG10619 in-situ | tup | - | -7611 | -29367 | UPSTREAM | CG10619-RA | "-" | CG10619-RB | "-"
 CG18397 in-situ | CG18397 | - | 55081 | 27658 | DOWNSTREAM | CG18397-RA | "-"


*********************** Rank 575 [Score 16.082397]   GBROWSE*******************

 CG17941 in-situ | ds | - | -5867 | -80814 | UPSTREAM | CG17941-RA | "-"
 CG2830 in-situ | Hsp60B | + | 7577 | 9731 | UPSTREAM | CG2830-RA | "-"

*********************** Rank 576 [Score 16.081482]   GBROWSE*******************

 CG6414 in-situ | CG6414 | - | -85336 | -87686 | UPSTREAM | CG6414-RA | "-"
 CG32790 in-situ | CG32790 | + | 21232 | 22530 | UPSTREAM | CG32790-RA | "-"

*********************** Rank 577 [Score 16.079102]   GBROWSE*******************

insitu CG6883 in-situ | trh | - | -42104 | -52897 | UPSTREAM | CG6883-RA | "-"
 CG13891 in-situ | CG13891 | - | 14470 | 13809 | DOWNSTREAM | CG13891-RA | "-"

*********************** Rank 578 [Score 16.069458]   GBROWSE*******************

 CG17839 in-situ | CG17839 | + | -68568 | 7913 | INTRAGENIC | intron:CG17839-RA:9 | CG17839-RA | "-"
 CG13467 in-situ | CG13467 | + | 30293 | 31316 | UPSTREAM | CG13467-RA | "-"

*********************** Rank 579 [Score 16.060242]   GBROWSE*******************

 CG11248 in-situ | CG11248 | + | -21786 | -18641 | DOWNSTREAM | CG11248-RB | "-" | CG11248-RA | "-"
 CG32447 in-situ | CG32447 | - | 4475 | -9069 | INTRAGENIC | intron:CG32447-RA:1 | CG32447-RA | "-"

*********************** Rank 580 [Score 16.059692]   GBROWSE*******************

 CG7580 in-situ | CG7580 | + | -2560 | -1475 | DOWNSTREAM | CG7580-RA | "-"
 CG13733 in-situ | CG13733 | - | 1605 | 466 | DOWNSTREAM | CG13733-RA | "-"

*********************** Rank 581 [Score 16.056519]   GBROWSE*******************

insitu CG11567 in-situ | Cpr | + | -8863 | -1353 | DOWNSTREAM | CG11567-RA | "-" | CG11567-RB | "-"
 CG9497 in-situ | CG9497 | - | 516 | -1093 | INTRAGENIC | intron:CG9497-RA:1 | CG9497-RA | "-"


*********************** Rank 582 [Score 16.055054]   GBROWSE*******************

 CG31006 in-situ | CG31006 | - | -23809 | -34757 | UPSTREAM | CG31006-RB | "-" | CG31006-RA | "-"
 CG1480 in-situ | bnk | + | 312 | 1883 | UPSTREAM | CG1480-RA | "-"

*********************** Rank 583 [Score 16.053772]   GBROWSE*******************

 CG12063 in-situ | CG12063 | + | -42789 | -34664 | DOWNSTREAM | CG12063-RA | "-"
insitu CG1499 in-situ | CG1499 | + | 1370 | 24505 | UPSTREAM | CG1499-RA | "-" | CG1499-RB | "-"

*********************** Rank 584 [Score 16.050659]   GBROWSE*******************

 CG13872 in-situ | CG13872 | - | -36767 | -38859 | UPSTREAM | CG13872-RA | "-"
 CG30447 in-situ | CG30447 | + | 29629 | 30170 | UPSTREAM | CG30447-RA | "-"


*********************** Rank 585 [Score 16.047974]   GBROWSE*******************

 CG15499 in-situ | CG15499 | - | -1197 | -1866 | UPSTREAM | CG15499-RA | "-"
 CG6534 in-situ | slou | - | 23918 | 15289 | DOWNSTREAM | CG6534-RA | "-"

*********************** Rank 586 [Score 16.037720]   GBROWSE*******************

 CG31481 in-situ | CG31481 | - | -19751 | -24569 | UPSTREAM | CG31481-RA | "-"
 CG1048 in-situ | zen2 | - | 11363 | 10355 | DOWNSTREAM | CG1048-RA | "-"


*********************** Rank 587 [Score 16.034119]   GBROWSE*******************

 CG18023 in-situ | Eip78C | + | -23405 | 14297 | INTRAGENIC | intron:CG18023-RA:3 | intron:CG18023-RB:3 | CG18023-RA | "-" | CG18023-RB | "-"
insitu CG9391 in-situ | CG9391 | - | 18016 | 16454 | DOWNSTREAM | CG9391-RB | "-" | CG9391-RA | "-"


*********************** Rank 588 [Score 16.033386]   GBROWSE*******************

 CG10249 in-situ | BcDNA:GH03482 | - | -2357 | -13099 | UPSTREAM | CG10249-RC | "-" | CG10249-RA | "-" | CG10249-RB | "-"
insitu CG10253 in-situ | CG10253 | - | 18726 | 15507 | DOWNSTREAM | CG10253-RA | "-"

*********************** Rank 589 [Score 16.029053]   GBROWSE*******************

 CG3413 in-situ | wdp | - | -3479 | -17186 | UPSTREAM | CG3413-RB | "-" | CG3413-RD | "-" | CG3413-RC | "-" | CG3413-RA | "-"
insitu CG5820 in-situ | Gp150 | + | 2263 | 14029 | UPSTREAM | CG5820-RA | "-" | CG5820-RB | "-" | CG5820-RC | "-" | CG5820-RD | "-"

*********************** Rank 590 [Score 16.025146]   GBROWSE*******************

 CG9709 in-situ | Acox57D-d | + | -52009 | -48959 | DOWNSTREAM | CG9709-RA | "-"
 CG10497 in-situ | Sdc | - | 39769 | -47782 | INTRAGENIC | intron:CG10497-RC:2 | intron:CG10497-RA:2 | intron:CG10497-RB:2 | CG10497-RC | "-" | CG10497-RA | "-" | CG10497-RB | "-"

*********************** Rank 591 [Score 16.024719]   GBROWSE*******************

 CG11340 in-situ | CG11340 | - | -1880 | -4112 | UPSTREAM | CG11340-RA | "-"
 CG11339 in-situ | CG11339 | + | 44109 | 55435 | UPSTREAM | CG11339-RA | "-"

*********************** Rank 592 [Score 16.022827]   GBROWSE*******************

insitu CG3380 in-situ | CG3380 | - | -6410 | -10968 | UPSTREAM | CG3380-RA | "-"
insitu CG5799 in-situ | dve | + | 9314 | 51769 | UPSTREAM | CG5799-RA | "-" | CG5799-RD | "-" | CG5799-RB | "-" | CG5799-RC | "-"

*********************** Rank 593 [Score 16.021729]   GBROWSE*******************

 CG2857 in-situ | CG2857 | - | -14012 | -15295 | UPSTREAM | CG2857-RA | "-"
 CG18506 in-situ | CG18506 | + | 601 | 5600 | UPSTREAM | CG18506-RA | "-"


*********************** Rank 594 [Score 16.018311]   GBROWSE*******************

 CG12425 in-situ | CG12425 | + | -47738 | -46183 | DOWNSTREAM | CG12425-RA | "-"
 CG4787 in-situ | CG4787 | + | 40537 | 42823 | UPSTREAM | CG4787-RA | "-"


*********************** Rank 595 [Score 16.014893]   GBROWSE*******************

 CG1149 in-situ | MstProx | - | -7487 | -9952 | UPSTREAM | CG1149-RA | "-"
 CG31513 in-situ | CG31513 | + | 12777 | 13425 | UPSTREAM | CG31513-RA | "-"

*********************** Rank 596 [Score 16.002563]   GBROWSE*******************

 CG32450 in-situ | CG32450 | + | -63344 | -62445 | DOWNSTREAM | CG32450-RA | "-"
insitu highlight CG5723 in-situ | Ten-m | - | 14765 | -100090 | INTRAGENIC | intron:CG5723-RB:1 | CG5723-RB | "-"

*********************** Rank 597 [Score 16.001709]   GBROWSE*******************

insitu highlight CG17943 in-situ | comm | - | -11948 | -18047 | UPSTREAM | CG17943-RA | "-"
 CG13445 in-situ | CG13445 | + | 69127 | 69512 | UPSTREAM | CG13445-RA | "-"

*********************** Rank 598 [Score 15.995850]   GBROWSE*******************

 CG13442 in-situ | CG13442 | - | -19328 | -22114 | UPSTREAM | CG13442-RA | "-"
 CG13443 in-situ | CG13443 | + | 10004 | 11388 | UPSTREAM | CG13443-RA | "-"


*********************** Rank 599 [Score 15.990356]   GBROWSE*******************

 CG32718 in-situ | CG32718 | + | -48897 | -47923 | DOWNSTREAM | CG32718-RA | "-"
 CG1402 in-situ | CG1402 | + | 8226 | 8912 | UPSTREAM | CG1402-RA | "-"


*********************** Rank 600 [Score 15.971436]   GBROWSE*******************

 CG6414 in-situ | CG6414 | - | -95986 | -98336 | UPSTREAM | CG6414-RA | "-"
 CG32790 in-situ | CG32790 | + | 10582 | 11880 | UPSTREAM | CG32790-RA | "-"

*********************** Rank 601 [Score 15.967957]   GBROWSE*******************

insitu highlight CG4345 in-situ | grim | - | -64707 | -66402 | UPSTREAM | CG4345-RA | "-"
 CG4319 in-situ | rpr | - | 29282 | 28432 | DOWNSTREAM | CG4319-RA | "-"

*********************** Rank 602 [Score 15.963745]   GBROWSE*******************

 CG6265 in-situ | CG6265 | - | -4285 | -10168 | UPSTREAM | CG6265-RA | "-" | CG6265-RB | "-"
insitu CG6134 in-situ | spz | - | 2005 | -2993 | INTRAGENIC | intron:CG6134-RA:1 | intron:CG6134-RB:1 | intron:CG6134-RC:1 | intron:CG6134-RD:1 | intron:CG6134-RE:1 | intron:CG6134-RF:1 | intron:CG6134-RG:1 | intron:CG6134-RH:1 | intron:CG6134-RI:1 | intron:CG6134-RJ:1 | CG6134-RA | "-" | CG6134-RB | "-" | CG6134-RC | "-" | CG6134-RD | "-" | CG6134-RE | "-" | CG6134-RF | "-" | CG6134-RG | "-" | CG6134-RH | "-" | CG6134-RI | "-" | CG6134-RJ | "-"


*********************** Rank 603 [Score 15.962524]   GBROWSE*******************

insitu highlight CG1934 in-situ | ImpE2 | - | -894 | -2598 | UPSTREAM | CG1934-RA | "-"
 CG10579 in-situ | Eip63E | + | 2114 | 94980 | UPSTREAM | CG10579-RD | "-" | CG10579-RE | "-" | CG10579-RA | "-" | CG10579-RB | "-" | CG10579-RC | "-"

*********************** Rank 604 [Score 15.958008]   GBROWSE*******************

insitu highlight CG6494 in-situ | h | + | -41881 | -38601 | DOWNSTREAM | CG6494-RA | "-"
 CG33162 in-situ | SrpRbeta | - | 2377 | 1427 | DOWNSTREAM | CG33162-RA | "-"

*********************** Rank 605 [Score 15.957153]   GBROWSE*******************

 CG4220 in-situ | elB | - | -3340 | -23976 | UPSTREAM | CG4220-RA | "-" | CG4220-RB | "-"
 CG15284 in-situ | CG15284 | - | 6220 | 5737 | DOWNSTREAM | CG15284-RA | "-"

*********************** Rank 606 [Score 15.955566]   GBROWSE*******************

insitu highlight CG10479 in-situ | CG10479 | - | -6287 | -14751 | UPSTREAM | CG10479-RA | "-"
 CG32406 in-situ | CG32406 | + | 14922 | 43918 | UPSTREAM | CG32406-RA | "-"


*********************** Rank 607 [Score 15.954956]   GBROWSE*******************

insitu CG2679 in-situ | gol | - | -30110 | -41810 | UPSTREAM | CG2679-RB | "-" | CG2679-RA | "-"
 CG30430 in-situ | CG30430 | + | 15002 | 15482 | UPSTREAM | CG30430-RA | "-"

*********************** Rank 608 [Score 15.951416]   GBROWSE*******************

 CG11280 in-situ | trn | + | -480 | 3335 | INTRAGENIC | intron:CG11280-RA:1 | CG11280-RA | "-"
 CG11281 in-situ | CG11281 | + | 58929 | 61944 | UPSTREAM | CG11281-RA | "-"


*********************** Rank 609 [Score 15.947571]   GBROWSE*******************

 CG12454 in-situ | CG12454 | + | -28049 | -27861 | DOWNSTREAM | CG12454-RA | "-"
 CG32614 in-situ | CG32614 | + | 34467 | 35177 | UPSTREAM | CG32614-RA | "-"

*********************** Rank 610 [Score 15.944702]   GBROWSE*******************

insitu CG31666 in-situ | CG31666 | + | -14973 | 30394 | INTRAGENIC | intron:CG31666-RD:1 | intron:CG31666-RA:1 | intron:CG31666-RB:1 | intron:CG31666-RC:1 | CG31666-RD | "-" | CG31666-RA | "-" | CG31666-RB | "-" | CG31666-RC | "-"
 CG31934 in-situ | CG31934 | - | 3081 | 2397 | DOWNSTREAM | CG31934-RA | "-"


*********************** Rank 611 [Score 15.941284]   GBROWSE*******************

 CG1867 in-situ | Or98b | + | -34120 | -32742 | DOWNSTREAM | CG1867-RA | "-"
 CG14064 in-situ | beat-VI | + | 31842 | 86847 | UPSTREAM | CG14064-RA | "-"

*********************** Rank 612 [Score 15.940063]   GBROWSE*******************

 CG11755 in-situ | CG11755 | + | -3728 | -3264 | DOWNSTREAM | CG11755-RA | "-"
insitu highlight CG9786 in-situ | hb | - | 15805 | 9303 | DOWNSTREAM | CG9786-RB | "-" | CG9786-RA | "-"

*********************** Rank 613 [Score 15.936523]   GBROWSE*******************

 CG9452 in-situ | CG9452 | - | -5810 | -8526 | UPSTREAM | CG9452-RA | "-"
 CG9283 in-situ | CG9283 | - | 9682 | 9068 | DOWNSTREAM | CG9283-RA | "-"

*********************** Rank 614 [Score 15.933838]   GBROWSE*******************

 CG11368 in-situ | CG11368 | + | -7065 | -6610 | DOWNSTREAM | CG11368-RA | "-"
 CG32719 in-situ | CG32719 | - | 53570 | 49618 | DOWNSTREAM | CG32719-RA | "-"

*********************** Rank 615 [Score 15.931946]   GBROWSE*******************

 CG8929 in-situ | CG8929 | + | -7571 | -4114 | DOWNSTREAM | CG8929-RC | "-" | CG8929-RB | "-" | CG8929-RA | "-"
 CG16739 in-situ | CG16739 | + | 7190 | 7936 | UPSTREAM | CG16739-RA | "-"


*********************** Rank 616 [Score 15.927856]   GBROWSE*******************

insitu highlight CG12287 in-situ | pdm2 | + | -3451 | 25033 | INTRAGENIC | intron:CG12287-RB:2 | CG12287-RB | "-" | CG12287-RA | "-"
 CG15485 in-situ | CG15485 | - | 7889 | 5985 | DOWNSTREAM | CG15485-RA | "-"

*********************** Rank 617 [Score 15.899902]   GBROWSE*******************

 CG13208 in-situ | Obp47b | - | -2095 | -2824 | UPSTREAM | CG13208-RA | "-"
 CG12389 in-situ | Fpps | - | 2494 | 431 | DOWNSTREAM | CG12389-RA | "-"

*********************** Rank 618 [Score 15.896118]   GBROWSE*******************

 CG13712 in-situ | CG13712 | - | -4003 | -4356 | UPSTREAM | CG13712-RA | "-"
 CG12493 in-situ | CG12493 | - | 39859 | 38534 | DOWNSTREAM | CG12493-RA | "-"

*********************** Rank 619 [Score 15.893677]   GBROWSE*******************

insitu highlight CG2939 in-situ | slp2 | + | -23639 | -21280 | DOWNSTREAM | CG2939-RA | "-"
 CG3964 in-situ | CG3964 | + | 2200 | 7026 | UPSTREAM | CG3964-RB | "-" | CG3964-RA | "-"

*********************** Rank 620 [Score 15.893311]   GBROWSE*******************

 CG14506 in-situ | CG14506 | - | -7383 | -8793 | UPSTREAM | CG14506-RA | "-"
 CG11958 in-situ | Cnx99A | - | 15032 | 10639 | DOWNSTREAM | CG11958-RA | "-" | CG11958-RB | "-"


*********************** Rank 621 [Score 15.887634]   GBROWSE*******************

 CG14521 in-situ | CG14521 | - | -5958 | -51317 | UPSTREAM | CG14521-RA | "-"
 CG14520 in-situ | CG14520 | + | 1606 | 2203 | UPSTREAM | CG14520-RA | "-"

*********************** Rank 622 [Score 15.882324]   GBROWSE*******************

 CG11313 in-situ | CG11313 | - | -6816 | -8035 | UPSTREAM | CG11313-RA | "-"
 CG15543 in-situ | CG15543 | + | 4939 | 5952 | UPSTREAM | CG15543-RA | "-"

*********************** Rank 623 [Score 15.867859]   GBROWSE*******************

 CG6559 in-situ | CG6559 | - | -66658 | -82896 | UPSTREAM | CG6559-RA | "-"
 CG12362 in-situ | CG12362 | + | 33445 | 35406 | UPSTREAM | CG12362-RB | "-" | CG12362-RA | "-"

*********************** Rank 624 [Score 15.858398]   GBROWSE*******************

 CG5099 in-situ | msi | + | -2490 | 81608 | INTRAGENIC | intron:CG5099-RB:2 | CG5099-RB | "-" | CG5099-RA | "-"
 CG4582 in-situ | CG4582 | - | 62151 | 60654 | DOWNSTREAM | CG4582-RA | "-"

*********************** Rank 625 [Score 15.855652]   GBROWSE*******************

 CG5488 in-situ | B-H2 | + | -36210 | -26629 | DOWNSTREAM | CG5488-RA | "-"
insitu CG5529 in-situ | B-H1 | + | 46710 | 52488 | UPSTREAM | CG5529-RA | "-"

*********************** Rank 626 [Score 15.855347]   GBROWSE*******************

 CG15532 in-situ | hdc | + | -75128 | 9107 | INTRAGENIC | intron:CG15532-RA:2 | intron:CG15532-RC:2 | CG15532-RA | "-" | CG15532-RC | "-" | CG15532-RB | "-"
 CG1469 in-situ | Fer2LCH | + | 34770 | 37522 | UPSTREAM | CG1469-RA | "-" | CG1469-RB | "-" | CG1469-RC | "-"

*********************** Rank 627 [Score 15.836121]   GBROWSE*******************

 CG10478 in-situ | CG10478 | - | -3741 | -5136 | UPSTREAM | CG10478-RA | "-"
 CG10477 in-situ | CG10477 | - | 19919 | 19043 | DOWNSTREAM | CG10477-RA | "-"

*********************** Rank 628 [Score 15.832764]   GBROWSE*******************

 CG2714 in-situ | crm | - | -2501 | -7034 | UPSTREAM | CG2714-RB | "-" | CG2714-RA | "-"
 CG2715 in-situ | Syx4 | - | 2788 | -2191 | INTRAGENIC | intron:CG2715-RA:5 | CG2715-RA | "-"

*********************** Rank 629 [Score 15.832153]   GBROWSE*******************

insitu highlight CG9739 in-situ | fz2 | - | -6295 | -34023 | UPSTREAM | CG9739-RB | "-" | CG9739-RA | "-"
 CG9730 in-situ | mRpL21 | - | 78136 | 77313 | DOWNSTREAM | CG9730-RA | "-"

*********************** Rank 630 [Score 15.830688]   GBROWSE*******************

 CG8809 in-situ | Camta | + | -11640 | -5026 | DOWNSTREAM | CG8809-RA | "-"
 CG1916 in-situ | Wnt2 | + | 11051 | 20072 | UPSTREAM | CG1916-RA | "-"

Polluted


*********************** Rank 631 [Score 15.829590]   GBROWSE*******************

 CG31820 in-situ | CG31820 | - | -30987 | -31498 | UPSTREAM | CG31820-RA | "-"
 CG4824 in-situ | BicC | + | 4766 | 11360 | UPSTREAM | CG4824-RA | "-" | CG4824-RB | "-" | CG4824-RD | "-"

*********************** Rank 632 [Score 15.827026]   GBROWSE*******************

insitu CG8487 in-situ | garz | + | -8063 | -915 | DOWNSTREAM | CG8487-RB | "-" | CG8487-RA | "-"
 CG8490 in-situ | CG8490 | + | 612 | 1468 | UPSTREAM | CG8490-RA | "-"

*********************** Rank 633 [Score 15.826416]   GBROWSE*******************

 CG31394 in-situ | CG31394 | - | -104254 | -104992 | UPSTREAM | CG31394-RA | "-"
insitu highlight CG17117 in-situ | hth | - | 5254 | -123570 | INTRAGENIC | intron:CG17117-RD:1 | CG17117-RD | "-" | CG17117-RB | "-" | CG17117-RC | "-" | CG17117-RA | "-"

*********************** Rank 634 [Score 15.826355]   GBROWSE*******************

 CG12877 in-situ | CG12877 | + | -5461 | -1870 | DOWNSTREAM | CG12877-RB | "-" | CG12877-RA | "-"
 CG5583 in-situ | Ets98B | + | 23977 | 34512 | UPSTREAM | CG5583-RA | "-"


*********************** Rank 635 [Score 15.810852]   GBROWSE*******************

 CG12110 in-situ | Pld | + | -92911 | -79669 | DOWNSTREAM | CG12110-RB | "-" | CG12110-RC | "-" | CG12110-RA | "-" | CG12110-RE | "-" | CG12110-RD | "-"
 CG9397 in-situ | 1.28 | + | 90270 | 91220 | UPSTREAM | CG9397-RA | "-"


*********************** Rank 636 [Score 15.808838]   GBROWSE*******************

insitu CG9468 in-situ | CG9468 | - | -27518 | -31109 | UPSTREAM | CG9468-RA | "-"
insitu highlight CG18024 in-situ | SoxN | + | 16856 | 20901 | UPSTREAM | CG18024-RA | "-"

*********************** Rank 637 [Score 15.797363]   GBROWSE*******************

 CG10119 in-situ | LamC | - | -2922 | -7920 | UPSTREAM | CG10119-RA | "-"
insitu CG12869 in-situ | CG12869 | + | 12479 | 16897 | UPSTREAM | CG12869-RA | "-"

*********************** Rank 638 [Score 15.795166]   GBROWSE*******************

insitu highlight CG1212 in-situ | p130CAS | + | -5560 | 8158 | INTRAGENIC | intron:CG1212-RA:1 | CG1212-RA | "-" | CG1212-RB | "-"
 CG7049 in-situ | CG7049 | - | 9856 | 8738 | DOWNSTREAM | CG7049-RA | "-"

*********************** Rank 639 [Score 15.794800]   GBROWSE*******************

 CG7855 in-situ | timeout | + | -27258 | 47967 | INTRAGENIC | intron:CG7855-RA:11 | CG7855-RA | "-"
 CG17319 in-situ | CG17319 | - | 12640 | 10202 | DOWNSTREAM | CG17319-RA | "-"


*********************** Rank 640 [Score 15.789795]   GBROWSE*******************

 CG3653 in-situ | kirre | + | -16784 | 15941 | INTRAGENIC | intron:CG3653-RB:1 | CG3653-RB | "-" | CG3653-RA | "-"
 CG3936 in-situ | N | + | 18010 | 55400 | UPSTREAM | CG3936-RA | "-"

*********************** Rank 641 [Score 15.789429]   GBROWSE*******************

 CG7313 in-situ | CG7313 | + | -7374 | -6625 | DOWNSTREAM | CG7313-RA | "-"
 CG5103 in-situ | CG5103 | - | 34896 | 32646 | DOWNSTREAM | CG5103-RA | "-"


*********************** Rank 642 [Score 15.774414]   GBROWSE*******************

 CG14662 in-situ | CG14662 | - | -699 | -2932 | UPSTREAM | CG14662-RA | "-"
 CG2022 in-situ | CG2022 | - | 3821 | 1734 | DOWNSTREAM | CG2022-RA | "-"

*********************** Rank 643 [Score 15.773071]   GBROWSE*******************

 CG5194 in-situ | CG5194 | - | -6639 | -7866 | UPSTREAM | CG5194-RA | "-"
insitu highlight CG5187 in-situ | Doc2 | - | 2750 | -3805 | INTRAGENIC | intron:CG5187-RA:2 | CG5187-RA | "-"

*********************** Rank 644 [Score 15.768372]   GBROWSE*******************

 CG6890 in-situ | Tollo | + | -13680 | -6489 | DOWNSTREAM | CG6890-RA | "-"
 CG7259 in-situ | Best4 | + | 49980 | 51571 | UPSTREAM | CG7259-RA | "-"

*********************** Rank 645 [Score 15.765259]   GBROWSE*******************

 CG6824 in-situ | ovo | + | -23939 | -2532 | DOWNSTREAM | CG6824-RB | "-" | CG6824-RC | "-" | CG6824-RA | "-"
 CG32767 in-situ | CG32767 | - | 8837 | 2398 | DOWNSTREAM | CG32767-RA | "-"

*********************** Rank 646 [Score 15.764893]   GBROWSE*******************

 CG15550 in-situ | CG15550 | - | -7114 | -7545 | UPSTREAM | CG15550-RA | "-"
 CG15548 in-situ | CG15548 | + | 15595 | 18039 | UPSTREAM | CG15548-RA | "-"


*********************** Rank 647 [Score 15.762451]   GBROWSE*******************

 CG5361 in-situ | CG5361 | - | -22894 | -24246 | UPSTREAM | CG5361-RA | "-"
 CG6203 in-situ | Fmr1 | - | 11862 | 3154 | DOWNSTREAM | CG6203-RB | "-" | CG6203-RA | "-" | CG6203-RC | "-" | CG6203-RD | "-" | CG6203-RE | "-"


*********************** Rank 648 [Score 15.758179]   GBROWSE*******************

 CG31394 in-situ | CG31394 | - | -96454 | -97192 | UPSTREAM | CG31394-RA | "-"
insitu highlight CG17117 in-situ | hth | - | 13054 | -115770 | INTRAGENIC | intron:CG17117-RD:1 | CG17117-RD | "-" | CG17117-RB | "-" | CG17117-RC | "-" | CG17117-RA | "-"


*********************** Rank 649 [Score 15.755188]   GBROWSE*******************

insitu CG13922 in-situ | mRpL46 | + | -4980 | -4074 | DOWNSTREAM | CG13922-RA | "-"
 CG32315 in-situ | Vanaso | - | 12897 | 7204 | DOWNSTREAM | CG32315-RA | "-"


*********************** Rank 650 [Score 15.754517]   GBROWSE*******************

 CG31760 in-situ | CG31760 | + | -8945 | 18492 | INTRAGENIC | intron:CG31760-RA:2 | CG31760-RA | "-"
 CG31861 in-situ | CG31861 | + | 10159 | 10766 | UPSTREAM | CG31861-RA | "-"

*********************** Rank 651 [Score 15.750977]   GBROWSE*******************

 CG10202 in-situ | CG10202 | - | -8337 | -10464 | UPSTREAM | CG10202-RA | "-"
 CG10205 in-situ | CG10205 | + | 4509 | 5631 | UPSTREAM | CG10205-RB | "-" | CG10205-RA | "-"


*********************** Rank 652 [Score 15.737366]   GBROWSE*******************

 CG9397 in-situ | 1.28 | + | -73130 | -72180 | DOWNSTREAM | CG9397-RA | "-"
 CG15233 in-situ | CG15233 | - | 25080 | 24007 | DOWNSTREAM | CG15233-RA | "-"

*********************** Rank 653 [Score 15.736633]   GBROWSE*******************

insitu CG32139 in-situ | Sox21b | - | -28646 | -47587 | UPSTREAM | CG32139-RA | "-"
insitu highlight CG5893 in-situ | D | - | 18650 | 15631 | DOWNSTREAM | CG5893-RA | "-"


*********************** Rank 654 [Score 15.729858]   GBROWSE*******************

insitu highlight CG4125 in-situ | rst | - | -7147 | -28772 | UPSTREAM | CG4125-RA | "-"
 CG4116 in-situ | CG4116 | - | 86583 | 85789 | DOWNSTREAM | CG4116-RA | "-"


*********************** Rank 655 [Score 15.729858]   GBROWSE*******************

insitu CG3979 in-situ | Indy | - | -6161 | -23789 | UPSTREAM | CG3979-RB | "-" | CG3979-RC | "-" | CG3979-RA | "-"
 CG6865 in-situ | CG6865 | + | 11828 | 13000 | UPSTREAM | CG6865-RA | "-"

*********************** Rank 656 [Score 15.719238]   GBROWSE*******************

insitu highlight CG5249 in-situ | CG5249 | + | -7459 | 10507 | INTRAGENIC | intron:CG5249-RA:1 | CG5249-RA | "-"
insitu CG17334 in-situ | lin-28 | + | 16241 | 18942 | UPSTREAM | CG17334-RA | "-"

*********************** Rank 657 [Score 15.717041]   GBROWSE*******************

 CG31695 in-situ | scw | - | -732 | -2143 | UPSTREAM | CG31695-RA | "-"
insitu CG10462 in-situ | CG10462 | - | 34181 | 30401 | DOWNSTREAM | CG10462-RA | "-"

*********************** Rank 658 [Score 15.711670]   GBROWSE*******************

 CG15389 in-situ | CG15389 | + | -2275 | -1500 | DOWNSTREAM | CG15389-RA | "-"
insitu CG7245 in-situ | CG7245 | + | 1969 | 9498 | UPSTREAM | CG7245-RA | "-"

*********************** Rank 659 [Score 15.702393]   GBROWSE*******************

 CG3517 in-situ | CG3517 | - | -23162 | -24604 | UPSTREAM | CG3517-RA | "-"
 CG6255 in-situ | CG6255 | - | 14705 | 13518 | DOWNSTREAM | CG6255-RA | "-"

*********************** Rank 660 [Score 15.700806]   GBROWSE*******************

 CG31369 in-situ | CG31369 | - | -32738 | -78054 | UPSTREAM | CG31369-RA | "-"
 CG11735 in-situ | Or85b | - | 34795 | 33514 | DOWNSTREAM | CG11735-RA | "-"


*********************** Rank 661 [Score 15.692871]   GBROWSE*******************

 CG31386 in-situ | CG31386 | - | -13602 | -39457 | UPSTREAM | CG31386-RA | "-"
 CG17216 in-situ | KP78b | - | 39581 | 37498 | DOWNSTREAM | CG17216-RA | "-"

*********************** Rank 662 [Score 15.692139]   GBROWSE*******************

 CG4881 in-situ | salr | + | -47224 | -39132 | DOWNSTREAM | CG4881-RA | "-" | CG4881-RB | "-"
insitu highlight CG6464 in-situ | salm | - | 33074 | 21782 | DOWNSTREAM | CG6464-RA | "-"

*********************** Rank 663 [Score 15.689087]   GBROWSE*******************

 CG12444 in-situ | CG12444 | - | -14544 | -16229 | UPSTREAM | CG12444-RB | "-" | CG12444-RA | "-"
 CG12443 in-situ | CG12443 | + | 5771 | 7831 | UPSTREAM | CG12443-RA | "-"

*********************** Rank 664 [Score 15.686707]   GBROWSE*******************

 CG31128 in-situ | CG31128 | + | -14274 | -13352 | DOWNSTREAM | CG31128-RA | "-"
insitu CG6844 in-situ | nAcRalpha-96Ab | + | 1771 | 7482 | UPSTREAM | CG6844-RA | "-" | CG6844-RB | "-"


*********************** Rank 665 [Score 15.682251]   GBROWSE*******************

 CG18404 in-situ | CG18404 | + | -14080 | -13164 | DOWNSTREAM | CG18404-RA | "-"
 CG15532 in-situ | hdc | + | 10672 | 94907 | UPSTREAM | CG15532-RA | "-" | CG15532-RC | "-" | CG15532-RB | "-"


*********************** Rank 666 [Score 15.679932]   GBROWSE*******************

 CG15004 in-situ | CG15004 | - | -1073 | -3332 | UPSTREAM | CG15004-RA | "-"
 CG18676 in-situ | CG18676 | - | 1789 | 443 | DOWNSTREAM | CG18676-RA | "-"

*********************** Rank 667 [Score 15.677246]   GBROWSE*******************

 CG30473 in-situ | Obp51a | - | -5285 | -5705 | UPSTREAM | CG30473-RA | "-"
insitu highlight CG11798 in-situ | CG11798 | + | 98791 | 114032 | UPSTREAM | CG11798-RA | "-"

*********************** Rank 668 [Score 15.670959]   GBROWSE*******************

 CG13707 in-situ | CG13707 | - | -5717 | -6380 | UPSTREAM | CG13707-RA | "-"
 CG32237 in-situ | CG32237 | - | 7996 | 4976 | DOWNSTREAM | CG32237-RA | "-"

*********************** Rank 669 [Score 15.665527]   GBROWSE*******************

 CG17208 in-situ | CG17208 | - | -39837 | -40172 | UPSTREAM | CG17208-RA | "-"
 CG5115 in-situ | CG5115 | + | 5994 | 6584 | UPSTREAM | CG5115-RB | "-"

*********************** Rank 670 [Score 15.662415]   GBROWSE*******************

insitu CG31122 in-situ | CG31122 | - | -7195 | -13431 | UPSTREAM | CG31122-RA | "-"
 CG14307 in-situ | fru | - | 125460 | -3469 | INTRAGENIC | intron:CG14307-RB:7 | intron:CG14307-RF:7 | CG14307-RH | "-" | CG14307-RB | "-" | CG14307-RC | "-" | CG14307-RE | "-" | CG14307-RF | "-" | CG14307-RG | "-" | CG14307-RA | "-" | CG14307-RD | "-"

*********************** Rank 671 [Score 15.658203]   GBROWSE*******************

 CG15147 in-situ | CG15147 | - | -82072 | -82670 | UPSTREAM | CG15147-RA | "-"
 CG7100 in-situ | CadN | - | 31104 | -58551 | INTRAGENIC | intron:CG7100-RA:9 | intron:CG7100-RC:9 | intron:CG7100-RD:9 | intron:CG7100-RE:9 | intron:CG7100-RF:9 | intron:CG7100-RG:9 | intron:CG7100-RH:9 | intron:CG7100-RB:9 | CG7100-RA | "-" | CG7100-RC | "-" | CG7100-RD | "-" | CG7100-RE | "-" | CG7100-RF | "-" | CG7100-RG | "-" | CG7100-RH | "-" | CG7100-RB | "-"

*********************** Rank 672 [Score 15.654907]   GBROWSE*******************

insitu CG15251 in-situ | CG15251 | - | -6734 | -10170 | UPSTREAM | CG15251-RA | "-"
insitu CG15252 in-situ | CG15252 | - | 3135 | 713 | DOWNSTREAM | CG15252-RA | "-"


*********************** Rank 673 [Score 15.653809]   GBROWSE*******************

 CG15058 in-situ | CG15058 | - | -94 | -297 | UPSTREAM | CG15058-RA | "-"
 CG15057 in-situ | CG15057 | - | 5658 | 5398 | DOWNSTREAM | CG15057-RA | "-"

*********************** Rank 674 [Score 15.652283]   GBROWSE*******************

 CG17738 in-situ | CG17738 | + | -29589 | -29257 | DOWNSTREAM | CG17738-RA | "-"
 CG4066 in-situ | CG4066 | + | 10844 | 12610 | UPSTREAM | CG4066-RA | "-"

*********************** Rank 675 [Score 15.645630]   GBROWSE*******************

 CG16800 in-situ | CG16800 | + | -1300 | -342 | DOWNSTREAM | CG16800-RA | "-"
insitu CG3762 in-situ | Vha68-2 | + | 6192 | 10485 | UPSTREAM | CG3762-RA | "-" | CG3762-RB | "-" | CG3762-RC | "-"

*********************** Rank 676 [Score 15.643311]   GBROWSE*******************

 CG1958 in-situ | CG1958 | + | -6669 | -5692 | DOWNSTREAM | CG1958-RA | "-"
 CG2059 in-situ | CG2059 | + | 13280 | 14476 | UPSTREAM | CG2059-RA | "-"

*********************** Rank 677 [Score 15.639954]   GBROWSE*******************

 CG14503 in-situ | CG14503 | + | -26838 | -26662 | DOWNSTREAM | CG14503-RA | "-"
 CG15066 in-situ | CG15066 | - | 44552 | 44016 | DOWNSTREAM | CG15066-RA | "-"

*********************** Rank 678 [Score 15.639954]   GBROWSE*******************

 CG11767 in-situ | Or24a | - | -5980 | -7392 | UPSTREAM | CG11767-RA | "-"
 CG31961 in-situ | CG31961 | - | 15708 | 14164 | DOWNSTREAM | CG31961-RA | "-" | CG31961-RB | "-"

*********************** Rank 679 [Score 15.637390]   GBROWSE*******************

 CG15030 in-situ | CG15030 | + | -3597 | -3328 | DOWNSTREAM | CG15030-RA | "-"
 CG9101 in-situ | CG9101 | - | 10305 | 9617 | DOWNSTREAM | CG9101-RA | "-"


*********************** Rank 680 [Score 15.633667]   GBROWSE*******************

 CG7527 in-situ | CadN2 | - | -4913 | -30912 | UPSTREAM | CG7527-RA | "-"
 CG5674 in-situ | CG5674 | + | 145821 | 157353 | UPSTREAM | CG5674-RA | "-" | CG5674-RB | "-" | CG5674-RC | "-"

*********************** Rank 681 [Score 15.629761]   GBROWSE*******************

 CG6127 in-situ | Ser | - | -14819 | -36717 | UPSTREAM | CG6127-RA | "-"
 CG31063 in-situ | CG31063 | - | 20611 | 18625 | DOWNSTREAM | CG31063-RA | "-"

*********************** Rank 682 [Score 15.628662]   GBROWSE*******************

 CG11777 in-situ | CG11777 | + | -1498 | -467 | DOWNSTREAM | CG11777-RA | "-"
 CG30320 in-situ | CG30320 | - | 7153 | 6299 | DOWNSTREAM | CG30320-RA | "-"


*********************** Rank 683 [Score 15.627686]   GBROWSE*******************

insitu CG9184 in-situ | CG9184 | - | -4819 | -5839 | UPSTREAM | CG9184-RA | "-" | CG9184-RB | "-"
 CG9160 in-situ | mtacp1 | + | 2185 | 4012 | UPSTREAM | CG9160-RA | "-" | CG9160-RB | "-"

*********************** Rank 684 [Score 15.627075]   GBROWSE*******************

 CG15405 in-situ | CG15405 | - | -12745 | -17094 | UPSTREAM | CG15405-RA | "-"
 CG3347 in-situ | CG3347 | + | 54171 | 66065 | UPSTREAM | CG3347-RA | "-"

*********************** Rank 685 [Score 15.627075]   GBROWSE*******************

 CG15636 in-situ | CG15636 | - | -26478 | -26798 | UPSTREAM | CG15636-RA | "-"
 CG15635 in-situ | CG15635 | + | 38535 | 41891 | UPSTREAM | CG15635-RA | "-"

*********************** Rank 686 [Score 15.626953]   GBROWSE*******************

insitu CG32139 in-situ | Sox21b | - | -7746 | -26687 | UPSTREAM | CG32139-RA | "-"
insitu highlight CG5893 in-situ | D | - | 39550 | 36531 | DOWNSTREAM | CG5893-RA | "-"

*********************** Rank 687 [Score 15.626221]   GBROWSE*******************

 CG7229 in-situ | CG7229 | + | -13016 | -10691 | DOWNSTREAM | CG7229-RA | "-"
insitu highlight CG7230 in-situ | rib | + | 2796 | 8466 | UPSTREAM | CG7230-RA | "-"


*********************** Rank 688 [Score 15.623657]   GBROWSE*******************

insitu CG3683 in-situ | CG3683 | + | -1607 | -592 | DOWNSTREAM | CG3683-RA | "-" | CG3683-RB | "-" | CG3683-RC | "-"
 CG4806 in-situ | CG4806 | - | 2298 | 120 | DOWNSTREAM | CG4806-RA | "-"


*********************** Rank 689 [Score 15.621094]   GBROWSE*******************

 CG30384 in-situ | CG30384 | + | -21101 | -18795 | DOWNSTREAM | CG30384-RA | "-"
 CG1854 in-situ | Or43a | - | 30815 | 28741 | DOWNSTREAM | CG1854-RA | "-"


*********************** Rank 690 [Score 15.620361]   GBROWSE*******************

 CG4641 in-situ | CG4641 | + | -5859 | -2931 | DOWNSTREAM | CG4641-RA | "-"
 CG10537 in-situ | Rdl | - | 28964 | 1955 | DOWNSTREAM | CG10537-RA | "-" | CG10537-RB | "-" | CG10537-RC | "-"

*********************** Rank 691 [Score 15.618530]   GBROWSE*******************

 CG31386 in-situ | CG31386 | - | -48702 | -74557 | UPSTREAM | CG31386-RA | "-"
 CG17216 in-situ | KP78b | - | 4481 | 2398 | DOWNSTREAM | CG17216-RA | "-"

*********************** Rank 692 [Score 15.615540]   GBROWSE*******************

 CG31085 in-situ | CG31085 | + | -47158 | -34074 | DOWNSTREAM | CG31085-RB | "-" | CG31085-RA | "-"
 CG14239 in-situ | CG14239 | - | 6725 | 5846 | DOWNSTREAM | CG14239-RA | "-"

*********************** Rank 693 [Score 15.609253]   GBROWSE*******************

 CG31750 in-situ | Gr36d | + | -2279 | -563 | DOWNSTREAM | CG31750-RA | "-"
 CG31745 in-situ | CG31745 | + | 61333 | 62266 | UPSTREAM | CG31745-RA | "-"


*********************** Rank 694 [Score 15.608032]   GBROWSE*******************

 CG13616 in-situ | CG13616 | + | -4348 | -3529 | DOWNSTREAM | CG13616-RA | "-"
 CG5610 in-situ | nAcRalpha-96Aa | - | 59658 | 3955 | DOWNSTREAM | CG5610-RA | "-"

*********************** Rank 695 [Score 15.605835]   GBROWSE*******************

insitu CG10710 in-situ | CG10710 | - | -116331 | -119659 | UPSTREAM | CG10710-RA | "-"
 CG12478 in-situ | bru-3 | - | 30817 | -98014 | INTRAGENIC | intron:CG12478-RA:4 | intron:CG12478-RB:3 | CG12478-RA | "-" | CG12478-RB | "-"

*********************** Rank 696 [Score 15.605469]   GBROWSE*******************

 CG13789 in-situ | CG13789 | - | -6481 | -7118 | UPSTREAM | CG13789-RA | "-"
 CG13790 in-situ | CG13790 | - | 25749 | 25465 | DOWNSTREAM | CG13790-RA | "-"

*********************** Rank 697 [Score 15.600098]   GBROWSE*******************

insitu CG3258 in-situ | ase | + | -6462 | -3776 | DOWNSTREAM | CG3258-RA | "-"
insitu CG3972 in-situ | Cyp4g1 | - | 2371 | 87 | DOWNSTREAM | CG3972-RA | "-"

*********************** Rank 698 [Score 15.594360]   GBROWSE*******************

 CG31706 in-situ | CG31706 | - | -18580 | -20108 | UPSTREAM | CG31706-RA | "-"
 CG6541 in-situ | Mst33A | - | 37204 | 35801 | DOWNSTREAM | CG6541-RA | "-" | CG6541-RB | "-"

*********************** Rank 699 [Score 15.593506]   GBROWSE*******************

 CG18265 in-situ | CG18265 | + | -17410 | -1538 | DOWNSTREAM | CG18265-RA | "-"
 CG7603 in-situ | CG7603 | + | 9128 | 9950 | UPSTREAM | CG7603-RA | "-"

*********************** Rank 700 [Score 15.593384]   GBROWSE*******************

insitu CG2493 in-situ | CG2493 | - | -23309 | -25173 | UPSTREAM | CG2493-RA | "-"
 CG15476 in-situ | CG15476 | - | 15392 | 15126 | DOWNSTREAM | CG15476-RA | "-"

*********************** Rank 701 [Score 15.588745]   GBROWSE*******************

 CG17178 in-situ | ACXE | + | -33161 | -29025 | DOWNSTREAM | CG17178-RA | "-"
 CG16800 in-situ | CG16800 | + | 8050 | 9008 | UPSTREAM | CG16800-RA | "-"

*********************** Rank 702 [Score 15.588135]   GBROWSE*******************

 CG4786 in-situ | CG4786 | - | -72098 | -84544 | UPSTREAM | CG4786-RA | "-"
insitu highlight CG4761 in-situ | knrl | - | 8024 | -15371 | INTRAGENIC | intron:CG4761-RA:2 | CG4761-RA | "-"

*********************** Rank 703 [Score 15.584351]   GBROWSE*******************

 CG7234 in-situ | Glu-RIIB | + | -5893 | -1733 | DOWNSTREAM | CG7234-RI | "-"
 CG14011 in-situ | CG14011 | - | 15881 | 14345 | DOWNSTREAM | CG14011-RA | "-" | CG14011-RB | "-" | CG14011-RC | "-"

*********************** Rank 704 [Score 15.580994]   GBROWSE*******************

insitu highlight CG10917 in-situ | fj | + | -4153 | -602 | DOWNSTREAM | CG10917-RA | "-"
 CG5581 in-situ | Ote | - | 40277 | 38687 | DOWNSTREAM | CG5581-RA | "-"


*********************** Rank 705 [Score 15.576294]   GBROWSE*******************

 CG6890 in-situ | Tollo | + | -18580 | -11389 | DOWNSTREAM | CG6890-RA | "-"
 CG7259 in-situ | Best4 | + | 45080 | 46671 | UPSTREAM | CG7259-RA | "-"


*********************** Rank 706 [Score 15.568359]   GBROWSE*******************

 CG4069 in-situ | CG4069 | - | -16093 | -17979 | UPSTREAM | CG4069-RA | "-"
 CG10632 in-situ | CG10632 | - | 19119 | -15894 | INTRAGENIC | intron:CG10632-RA:2 | CG10632-RA | "-" | CG10632-RB | "-"

*********************** Rank 707 [Score 15.567505]   GBROWSE*******************

 CG18480 in-situ | BG:DS07108.4 | + | -20382 | -17841 | DOWNSTREAM | CG18480-RA | "-"
 CG4587 in-situ | BG:DS07108.2 | - | 2354 | -2631 | INTRAGENIC | intron:CG4587-RA:6 | CG4587-RA | "-"


*********************** Rank 708 [Score 15.567017]   GBROWSE*******************

 CG4356 in-situ | mAcR-60C | - | -6520 | -17596 | UPSTREAM | CG4356-RA | "-" | CG4356-RB | "-"
 CG4527 in-situ | CG4527 | - | 6456 | -4103 | INTRAGENIC | intron:CG4527-RA:10 | intron:CG4527-RB:10 | CG4527-RA | "-" | CG4527-RB | "-"


*********************** Rank 709 [Score 15.566406]   GBROWSE*******************

insitu highlight CG2411 in-situ | ptc | + | -4331 | 9583 | INTRAGENIC | intron:CG2411-RA:1 | CG2411-RA | "-" | CG2411-RB | "-"
 CG30353 in-situ | CG30353 | + | 3453 | 4121 | UPSTREAM | CG30353-RA | "-"


*********************** Rank 710 [Score 15.564575]   GBROWSE*******************

 CG6154 in-situ | CG6154 | + | -42651 | -35125 | DOWNSTREAM | CG6154-RA | "-" | CG6154-RB | "-"
 CG14559 in-situ | CG14559 | + | 13045 | 26334 | UPSTREAM | CG14559-RA | "-"

*********************** Rank 711 [Score 15.564209]   GBROWSE*******************

 CG13739 in-situ | CG13739 | - | -2319 | -36412 | UPSTREAM | CG13739-RA | "-"
 CG13954 in-situ | CG13954 | - | 20168 | 17391 | DOWNSTREAM | CG13954-RA | "-"

*********************** Rank 712 [Score 15.562256]   GBROWSE*******************

 CG10001 in-situ | AR-2 | - | -4029 | -11053 | UPSTREAM | CG10001-RA | "-"
 CG10000 in-situ | CG10000 | - | 3429 | 221 | DOWNSTREAM | CG10000-RA | "-"

*********************** Rank 713 [Score 15.558899]   GBROWSE*******************

insitu highlight CG3242 in-situ | sob | - | -11865 | -14621 | UPSTREAM | CG3242-RA | "-"
insitu highlight CG3851 in-situ | odd | - | 14117 | 11591 | DOWNSTREAM | CG3851-RA | "-"


*********************** Rank 714 [Score 15.552551]   GBROWSE*******************

 CG4021 in-situ | CG4021 | + | -7754 | -6274 | DOWNSTREAM | CG4021-RA | "-"
 CG4402 in-situ | lox2 | - | 10556 | 8517 | DOWNSTREAM | CG4402-RA | "-"


*********************** Rank 715 [Score 15.536987]   GBROWSE*******************

insitu highlight CG4345 in-situ | grim | - | -88157 | -89852 | UPSTREAM | CG4345-RA | "-"
 CG4319 in-situ | rpr | - | 5832 | 4982 | DOWNSTREAM | CG4319-RA | "-"

*********************** Rank 716 [Score 15.530640]   GBROWSE*******************

 CG18249 in-situ | CG18249 | + | -9644 | -7646 | DOWNSTREAM | CG18249-RA | "-"
 CG7602 in-situ | DNApol-iota | + | 3694 | 6993 | UPSTREAM | CG7602-RB | "-" | CG7602-RA | "-"

*********************** Rank 717 [Score 15.528931]   GBROWSE*******************

insitu CG3479 in-situ | osp | - | -21684 | -111222 | UPSTREAM | CG3479-RA | "-"
insitu CG15282 in-situ | BG:DS07721.3 | + | 1670 | 2272 | UPSTREAM | CG15282-RA | "-"

*********************** Rank 718 [Score 15.521729]   GBROWSE*******************

insitu CG3324 in-situ | Pkg21D | - | -16840 | -21482 | UPSTREAM | CG3324-RA | "-"
 CG31658 in-situ | CG31658 | + | 3011 | 3797 | UPSTREAM | CG31658-RA | "-"

*********************** Rank 719 [Score 15.510864]   GBROWSE*******************

 CG12438 in-situ | CG12438 | + | -8919 | -8194 | DOWNSTREAM | CG12438-RA | "-"
insitu CG9463 in-situ | CG9463 | - | 7389 | 4040 | DOWNSTREAM | CG9463-RA | "-"

*********************** Rank 720 [Score 15.490479]   GBROWSE*******************

 CG4746 in-situ | mab-2 | - | -9646 | -16964 | UPSTREAM | CG4746-RA | "-"
insitu CG4336 in-situ | rux | - | 14352 | 12921 | DOWNSTREAM | CG4336-RA | "-"

*********************** Rank 721 [Score 15.489380]   GBROWSE*******************

 CG1211 in-situ | CG1211 | + | -367 | 1962 | INTRAGENIC | intron:CG1211-RA:1 | CG1211-RA | "-"
insitu CG9194 in-situ | CG9194 | + | 2458 | 8754 | UPSTREAM | CG9194-RA | "-"

*********************** Rank 722 [Score 15.487915]   GBROWSE*******************

insitu highlight CG10197 in-situ | kn | - | -1270 | -27577 | UPSTREAM | CG10197-RA | "-" | CG10197-RB | "-"
 CG10200 in-situ | CG10200 | + | 8200 | 9709 | UPSTREAM | CG10200-RB | "-" | CG10200-RA | "-"

*********************** Rank 723 [Score 15.479004]   GBROWSE*******************

 CG16898 in-situ | CG16898 | - | -27301 | -28669 | UPSTREAM | CG16898-RA | "-"
 CG8896 in-situ | 18w | + | 79223 | 84644 | UPSTREAM | CG8896-RA | "-"

*********************** Rank 724 [Score 15.465942]   GBROWSE*******************

 CG32156 in-situ | Mbs | + | -21653 | 8409 | INTRAGENIC | intron:CG32156-RC:15 | intron:CG32156-RA:14 | intron:CG32156-RD:15 | intron:CG32156-RB:14 | intron:CG32156-RE:1 | CG32156-RC | "-" | CG32156-RA | "-" | CG32156-RD | "-" | CG32156-RB | "-" | CG32156-RE | "-"
 CG13075 in-situ | CG13075 | + | 10415 | 11557 | UPSTREAM | CG13075-RA | "-"

*********************** Rank 725 [Score 15.464111]   GBROWSE*******************

 CG10277 in-situ | CG10277 | + | -7067 | -1674 | DOWNSTREAM | CG10277-RA | "-" | CG10277-RB | "-" | CG10277-RC | "-"
 CG1021 in-situ | CG1021 | - | 15913 | -819 | INTRAGENIC | intron:CG1021-RA:10 | intron:CG1021-RB:10 | CG1021-RA | "-" | CG1021-RB | "-"

*********************** Rank 726 [Score 15.463379]   GBROWSE*******************

 CG6154 in-situ | CG6154 | + | -15401 | -7875 | DOWNSTREAM | CG6154-RA | "-" | CG6154-RB | "-"
 CG14559 in-situ | CG14559 | + | 40295 | 53584 | UPSTREAM | CG14559-RA | "-"

*********************** Rank 727 [Score 15.460205]   GBROWSE*******************

 CG10293 in-situ | how | + | -18122 | 19013 | INTRAGENIC | intron:CG10293-RA:2 | intron:CG10293-RB:2 | CG10293-RA | "-" | CG10293-RB | "-"
 CG13408 in-situ | CG13408 | + | 20148 | 22824 | UPSTREAM | CG13408-RA | "-"

*********************** Rank 728 [Score 15.458374]   GBROWSE*******************

insitu CG6586 in-situ | tan | + | -3364 | -1700 | DOWNSTREAM | CG6586-RA | "-"
 CG6592 in-situ | CG6592 | + | 1404 | 2836 | UPSTREAM | CG6592-RA | "-"

*********************** Rank 729 [Score 15.454102]   GBROWSE*******************

insitu highlight CG1849 in-situ | run | + | -30644 | -27759 | DOWNSTREAM | CG1849-RA | "-"
 CG1324 in-situ | CG1324 | - | 10875 | 9659 | DOWNSTREAM | CG1324-RA | "-"

*********************** Rank 730 [Score 15.445923]   GBROWSE*******************

insitu CG6703 in-situ | Caki | - | -5122 | -42019 | UPSTREAM | CG6703-RB | "-" | CG6703-RA | "-" | CG6703-RC | "-"
 CG13417 in-situ | Gr93a | + | 4739 | 6249 | UPSTREAM | CG13417-RA | "-"

*********************** Rank 731 [Score 15.444641]   GBROWSE*******************

insitu CG31122 in-situ | CG31122 | - | -59795 | -66031 | UPSTREAM | CG31122-RA | "-"
 CG14307 in-situ | fru | - | 72860 | -56069 | INTRAGENIC | intron:CG14307-RH:2 | intron:CG14307-RB:2 | intron:CG14307-RC:2 | intron:CG14307-RE:2 | intron:CG14307-RF:2 | intron:CG14307-RG:2 | CG14307-RH | "-" | CG14307-RB | "-" | CG14307-RC | "-" | CG14307-RE | "-" | CG14307-RF | "-" | CG14307-RG | "-" | CG14307-RA | "-" | CG14307-RD | "-"

*********************** Rank 732 [Score 15.443359]   GBROWSE*******************

 CG3942 in-situ | CG3942 | + | -2522 | -117 | DOWNSTREAM | CG3942-RA | "-"
 CG11502 in-situ | svp | + | 2040 | 46078 | UPSTREAM | CG11502-RC | "-" | CG11502-RA | "-" | CG11502-RB | "-"

*********************** Rank 733 [Score 15.442139]   GBROWSE*******************

 CG7320 in-situ | CG7320 | + | -14132 | -12405 | DOWNSTREAM | CG7320-RA | "-"
 CG7313 in-situ | CG7313 | + | 36126 | 36875 | UPSTREAM | CG7313-RA | "-"


*********************** Rank 734 [Score 15.440186]   GBROWSE*******************

 CG4559 in-situ | Idgf3 | + | -25009 | -22740 | DOWNSTREAM | CG4559-RC | "-" | CG4559-RB | "-" | CG4559-RA | "-"
insitu CG4952 in-situ | dac | - | 10068 | -9406 | INTRAGENIC | intron:CG4952-RA:2 | intron:CG4952-RB:3 | intron:CG4952-RC:2 | intron:CG4952-RD:3 | intron:CG4952-RE:2 | CG4952-RA | "-" | CG4952-RB | "-" | CG4952-RC | "-" | CG4952-RD | "-" | CG4952-RE | "-"

*********************** Rank 735 [Score 15.437744]   GBROWSE*******************

 CG15580 in-situ | CG15580 | - | -15993 | -18820 | UPSTREAM | CG15580-RA | "-" | CG15580-RB | "-"
 CG15581 in-situ | Or83c | - | 4753 | 3274 | DOWNSTREAM | CG15581-RA | "-"


*********************** Rank 736 [Score 15.428772]   GBROWSE*******************

 CG9895 in-situ | CG9895 | - | -3781 | -5663 | UPSTREAM | CG9895-RA | "-"
insitu CG2956 in-situ | twi | + | 28326 | 30544 | UPSTREAM | CG2956-RA | "-"


*********************** Rank 737 [Score 15.427185]   GBROWSE*******************

insitu highlight CG2189 in-situ | Dfd | + | -53941 | -43346 | DOWNSTREAM | CG2189-RA | "-"
insitu highlight CG1030 in-situ | Scr | - | 2842 | -22657 | INTRAGENIC | intron:CG1030-RA:1 | CG1030-RA | "-"

*********************** Rank 738 [Score 15.425049]   GBROWSE*******************

 CG6163 in-situ | CG6163 | - | -4483 | -7382 | UPSTREAM | CG6163-RA | "-"
 CG11726 in-situ | CG11726 | + | 25403 | 26278 | UPSTREAM | CG11726-RA | "-"

*********************** Rank 739 [Score 15.423584]   GBROWSE*******************

 CG14045 in-situ | EG:BACH7M4.1 | - | -7614 | -17160 | UPSTREAM | CG14045-RA | "-"
 CG12496 in-situ | EG:BACH7M4.4 | - | 15621 | 14079 | DOWNSTREAM | CG12496-RA | "-"

*********************** Rank 740 [Score 15.421448]   GBROWSE*******************

insitu highlight CG9908 in-situ | disco | - | -16347 | -22483 | UPSTREAM | CG9908-RA | "-"
 CG12507 in-situ | CG12507 | - | 33383 | 32299 | DOWNSTREAM | CG12507-RA | "-"

*********************** Rank 741 [Score 15.418823]   GBROWSE*******************

 CG5870 in-situ | beta-Spec | + | -1770 | 10046 | INTRAGENIC | intron:CG5870-RA:1 | CG5870-RA | "-"
 CG12990 in-situ | CG12990 | - | 12697 | 10017 | DOWNSTREAM | CG12990-RA | "-"

*********************** Rank 742 [Score 15.413269]   GBROWSE*******************

 CG31304 in-situ | CG31304 | - | -860 | -6006 | UPSTREAM | CG31304-RA | "-"
insitu CG6934 in-situ | CG6934 | - | 28863 | 8529 | DOWNSTREAM | CG6934-RA | "-"

*********************** Rank 743 [Score 15.411987]   GBROWSE*******************

 CG10363 in-situ | TepIV | + | -1424 | 5238 | INTRAGENIC | intron:CG10363-RA:1 | CG10363-RA | "-"
 CG13079 in-situ | CG13079 | + | 6830 | 11832 | UPSTREAM | CG13079-RA | "-"

*********************** Rank 744 [Score 15.409546]   GBROWSE*******************

 CG3347 in-situ | CG3347 | + | -15929 | -4035 | DOWNSTREAM | CG3347-RA | "-"
 CG3332 in-situ | CG3332 | - | 4802 | 63 | DOWNSTREAM | CG3332-RB | "-" | CG3332-RA | "-"


*********************** Rank 745 [Score 15.408691]   GBROWSE*******************

 CG7527 in-situ | CadN2 | - | -89263 | -115262 | UPSTREAM | CG7527-RA | "-"
 CG5674 in-situ | CG5674 | + | 61471 | 73003 | UPSTREAM | CG5674-RA | "-" | CG5674-RB | "-" | CG5674-RC | "-"

*********************** Rank 746 [Score 15.407349]   GBROWSE*******************

 CG1421 in-situ | CG1421 | + | -2462 | -1785 | DOWNSTREAM | CG1421-RA | "-"
 CG1428 in-situ | CG1428 | + | 765 | 2808 | UPSTREAM | CG1428-RA | "-"

*********************** Rank 747 [Score 15.405884]   GBROWSE*******************

 CG10037 in-situ | vvl | + | -96811 | -92435 | DOWNSTREAM | CG10037-RA | "-"
insitu CG10078 in-situ | Prat2 | - | 27887 | 25022 | DOWNSTREAM | CG10078-RB | "-" | CG10078-RA | "-"

*********************** Rank 748 [Score 15.405640]   GBROWSE*******************

 CG9143 in-situ | CG9143 | - | -1642 | -4668 | UPSTREAM | CG9143-RA | "-"
 CG9090 in-situ | CG9090 | - | 486 | -1582 | INTRAGENIC | intron:CG9090-RA:1 | CG9090-RA | "-"

*********************** Rank 749 [Score 15.402222]   GBROWSE*******************

 CG5006 in-situ | Or33c | + | -4560 | -3327 | DOWNSTREAM | CG5006-RA | "-"
 CG16963 in-situ | Cry | + | 1612 | 4276 | UPSTREAM | CG16963-RA | "-"

*********************** Rank 750 [Score 15.400635]   GBROWSE*******************

 CG2616 in-situ | CG2616 | + | -12896 | -10895 | DOWNSTREAM | CG2616-RA | "-"
 CG3027 in-situ | pyd3 | - | 14763 | 12600 | DOWNSTREAM | CG3027-RA | "-"

*********************** Rank 751 [Score 15.383850]   GBROWSE*******************

 CG2616 in-situ | CG2616 | + | -11696 | -9695 | DOWNSTREAM | CG2616-RA | "-"
 CG3027 in-situ | pyd3 | - | 15963 | 13800 | DOWNSTREAM | CG3027-RA | "-"


*********************** Rank 752 [Score 15.381348]   GBROWSE*******************

 CG3837 in-situ | CG3837 | + | -23980 | -20355 | DOWNSTREAM | CG3837-RA | "-"
 CG14861 in-situ | CG14861 | + | 72110 | 73377 | UPSTREAM | CG14861-RA | "-"

*********************** Rank 753 [Score 15.377563]   GBROWSE*******************

 CG13291 in-situ | CG13291 | - | -20165 | -21127 | UPSTREAM | CG13291-RA | "-"
insitu highlight CG10479 in-situ | CG10479 | - | 1413 | -7051 | INTRAGENIC | intron:CG10479-RA:1 | CG10479-RA | "-"

*********************** Rank 754 [Score 15.375977]   GBROWSE*******************

 CG12605 in-situ | CG12605 | - | -1600 | -9531 | UPSTREAM | CG12605-RB | "-"
 CG1130 in-situ | scrt | + | 17545 | 22218 | UPSTREAM | CG1130-RA | "-"

*********************** Rank 755 [Score 15.373291]   GBROWSE*******************

insitu CG10710 in-situ | CG10710 | - | -114081 | -117409 | UPSTREAM | CG10710-RA | "-"
 CG12478 in-situ | bru-3 | - | 33067 | -95764 | INTRAGENIC | intron:CG12478-RA:4 | intron:CG12478-RB:3 | CG12478-RA | "-" | CG12478-RB | "-"

*********************** Rank 756 [Score 15.363525]   GBROWSE*******************

 CG9265 in-situ | CG9265 | - | -1115 | -13304 | UPSTREAM | CG9265-RA | "-"
 CG9264 in-situ | CG9264 | + | 3 | 10047 | UPSTREAM | CG9264-RB | "-" | CG9264-RA | "-"


*********************** Rank 757 [Score 15.362305]   GBROWSE*******************

 CG7554 in-situ | comm2 | - | -22072 | -24330 | UPSTREAM | CG7554-RA | "-"
insitu highlight CG17943 in-situ | comm | - | 6502 | 403 | DOWNSTREAM | CG17943-RA | "-"

*********************** Rank 758 [Score 15.356934]   GBROWSE*******************

 CG9266 in-situ | CG9266 | - | -61465 | -64889 | UPSTREAM | CG9266-RB | "-"
insitu CG1762 in-situ | betaInt-nu | + | 22426 | 27638 | UPSTREAM | CG1762-RA | "-"

*********************** Rank 759 [Score 15.355469]   GBROWSE*******************

 CG30111 in-situ | CG30111 | + | -16530 | -13151 | DOWNSTREAM | CG30111-RA | "-"
 CG11430 in-situ | olf186-F | + | 12570 | 29695 | UPSTREAM | CG11430-RB | "-" | CG11430-RC | "-" | CG11430-RA | "-"

*********************** Rank 760 [Score 15.353882]   GBROWSE*******************

insitu CG8112 in-situ | CG8112 | + | -9531 | 4755 | INTRAGENIC | intron:CG8112-RA:2 | CG8112-RA | "-" | CG8112-RB | "-"
 CG9790 in-situ | CG9790 | - | 5343 | 4687 | DOWNSTREAM | CG9790-RA | "-"

*********************** Rank 761 [Score 15.348511]   GBROWSE*******************

insitu highlight CG6464 in-situ | salm | - | -7726 | -19018 | UPSTREAM | CG6464-RA | "-"
insitu highlight CG4922 in-situ | sala | + | 32860 | 33626 | UPSTREAM | CG4922-RA | "-"

*********************** Rank 762 [Score 15.344971]   GBROWSE*******************

insitu highlight CG4761 in-situ | knrl | - | -10826 | -34221 | UPSTREAM | CG4761-RA | "-"
 CG13251 in-situ | CG13251 | + | 5868 | 9433 | UPSTREAM | CG13251-RA | "-"

*********************** Rank 763 [Score 15.344788]   GBROWSE*******************

insitu CG7891 in-situ | CG7891 | + | -35099 | -32867 | DOWNSTREAM | CG7891-RA | "-"
 CG9656 in-situ | grn | - | 19729 | -14363 | INTRAGENIC | intron:CG9656-RA:4 | CG9656-RA | "-"

*********************** Rank 764 [Score 15.341248]   GBROWSE*******************

insitu CG3619 in-situ | Dl | - | -8648 | -32130 | UPSTREAM | CG3619-RA | "-" | CG3619-RB | "-"
 CG3581 in-situ | CG3581 | - | 39888 | 38896 | DOWNSTREAM | CG3581-RA | "-"

*********************** Rank 765 [Score 15.340881]   GBROWSE*******************

 CG32169 in-situ | CG32169 | - | -84319 | -95788 | UPSTREAM | CG32169-RA | "-"
 CG32175 in-situ | CG32175 | + | 34190 | 34840 | UPSTREAM | CG32175-RA | "-"

*********************** Rank 766 [Score 15.339600]   GBROWSE*******************

 CG8117 in-situ | CG8117 | + | -1795 | -1307 | DOWNSTREAM | CG8117-RA | "-"
 CG8119 in-situ | CG8119 | + | 16499 | 17233 | UPSTREAM | CG8119-RA | "-"

*********************** Rank 767 [Score 15.338867]   GBROWSE*******************

 CG31702 in-situ | CG31702 | + | -3821 | -2299 | DOWNSTREAM | CG31702-RA | "-"
 CG6691 in-situ | CG6691 | + | 4698 | 5745 | UPSTREAM | CG6691-RA | "-"

*********************** Rank 768 [Score 15.336548]   GBROWSE*******************

 CG32320 in-situ | CG32320 | + | -8267 | -5843 | DOWNSTREAM | CG32320-RA | "-"
 CG9168 in-situ | CG9168 | + | 4355 | 6207 | UPSTREAM | CG9168-RA | "-"


*********************** Rank 769 [Score 15.335571]   GBROWSE*******************

 CG4328 in-situ | CG4328 | - | -29341 | -31267 | UPSTREAM | CG4328-RA | "-"
insitu highlight CG32105 in-situ | CG32105 | + | 6933 | 13603 | UPSTREAM | CG32105-RB | "-"

*********************** Rank 770 [Score 15.334229]   GBROWSE*******************

 CG4916 in-situ | me31B | + | -2465 | 384 | INTRAGENIC | intron:CG4916-RA:4 | intron:CG4916-RB:4 | CG4916-RA | "-" | CG4916-RB | "-"
 CG5686 in-situ | chico | - | 5273 | 719 | DOWNSTREAM | CG5686-RA | "-"

*********************** Rank 771 [Score 15.332764]   GBROWSE*******************

 CG9300 in-situ | CG9300 | + | -9121 | -6771 | DOWNSTREAM | CG9300-RA | "-"
insitu CG9262 in-situ | Shal | - | 23037 | 10037 | DOWNSTREAM | CG9262-RA | "-"

*********************** Rank 772 [Score 15.332520]   GBROWSE*******************

 CG15541 in-situ | CG15541 | + | -15095 | -8551 | DOWNSTREAM | CG15541-RA | "-"
 CG1342 in-situ | CG1342 | + | 26382 | 28506 | UPSTREAM | CG1342-RA | "-"


*********************** Rank 773 [Score 15.330688]   GBROWSE*******************

insitu highlight CG5461 in-situ | bun | - | -25409 | -109402 | UPSTREAM | CG5461-RA | "-" | CG5461-RB | "-" | CG5461-RC | "-"
 CG15489 in-situ | CG15489 | + | 27808 | 28710 | UPSTREAM | CG15489-RA | "-"

*********************** Rank 774 [Score 15.322998]   GBROWSE*******************

 CG12756 in-situ | CG12756 | - | -13102 | -14183 | UPSTREAM | CG12756-RA | "-"
insitu highlight CG5249 in-situ | CG5249 | + | 9641 | 27607 | UPSTREAM | CG5249-RA | "-"

*********************** Rank 775 [Score 15.317017]   GBROWSE*******************

 CG13527 in-situ | CG13527 | + | -12369 | -11236 | DOWNSTREAM | CG13527-RA | "-"
 CG13530 in-situ | CG13530 | - | 2893 | 457 | DOWNSTREAM | CG13530-RA | "-"

*********************** Rank 776 [Score 15.316650]   GBROWSE*******************

insitu CG10953 in-situ | CG10953 | - | -23824 | -24864 | UPSTREAM | CG10953-RA | "-"
 CG10950 in-situ | CG10950 | + | 4878 | 6839 | UPSTREAM | CG10950-RA | "-"

*********************** Rank 777 [Score 15.311951]   GBROWSE*******************

insitu CG31337 in-situ | CG31337 | - | -7025 | -8437 | UPSTREAM | CG31337-RA | "-"
 CG14370 in-situ | CG14370 | + | 35778 | 36176 | UPSTREAM | CG14370-RA | "-"

*********************** Rank 778 [Score 15.310059]   GBROWSE*******************

 CG31419 in-situ | CG31419 | + | -17953 | -17480 | DOWNSTREAM | CG31419-RA | "-"
insitu CG14334 in-situ | beat-IIa | - | 63254 | 17136 | DOWNSTREAM | CG14334-RA | "-"


*********************** Rank 779 [Score 15.309998]   GBROWSE*******************

 CG13785 in-situ | CG13785 | + | -3386 | -2489 | DOWNSTREAM | CG13785-RA | "-"
insitu highlight CG4889 in-situ | wg | + | 21242 | 30336 | UPSTREAM | CG4889-RA | "-" | CG4889-RB | "-"

*********************** Rank 780 [Score 15.307007]   GBROWSE*******************

insitu highlight CG10798 in-situ | dm | + | -5929 | 6904 | INTRAGENIC | intron:CG10798-RA:2 | CG10798-RA | "-"
 CG12535 in-situ | CG12535 | - | 14302 | 13517 | DOWNSTREAM | CG12535-RB | "-" | CG12535-RA | "-"

*********************** Rank 781 [Score 15.306396]   GBROWSE*******************

 CG32193 in-situ | CG32193 | + | -20861 | -16841 | DOWNSTREAM | CG32193-RA | "-"
 CG32192 in-situ | CG32192 | + | 57796 | 58531 | UPSTREAM | CG32192-RA | "-" | CG32192-RB | "-"


*********************** Rank 782 [Score 15.302490]   GBROWSE*******************

 CG13712 in-situ | CG13712 | - | -12703 | -13056 | UPSTREAM | CG13712-RA | "-"
 CG12493 in-situ | CG12493 | - | 31159 | 29834 | DOWNSTREAM | CG12493-RA | "-"

*********************** Rank 783 [Score 15.301392]   GBROWSE*******************

insitu highlight CG3340 in-situ | Kr | + | -9365 | -6446 | DOWNSTREAM | CG3340-RA | "-"
 CG30429 in-situ | CG30429 | + | 10549 | 11524 | UPSTREAM | CG30429-RA | "-"

*********************** Rank 784 [Score 15.295837]   GBROWSE*******************

 CG15280 in-situ | BG:DS06874.4 | - | -11333 | -12351 | UPSTREAM | CG15280-RA | "-"
 CG15279 in-situ | BG:DS03431.1 | - | 57683 | 50930 | DOWNSTREAM | CG15279-RB | "-" | CG15279-RA | "-"

*********************** Rank 785 [Score 15.292053]   GBROWSE*******************

 CG10037 in-situ | vvl | + | -31361 | -26985 | DOWNSTREAM | CG10037-RA | "-"
insitu CG10078 in-situ | Prat2 | - | 93337 | 90472 | DOWNSTREAM | CG10078-RB | "-" | CG10078-RA | "-"

*********************** Rank 786 [Score 15.287598]   GBROWSE*******************

 CG4774 in-situ | CG4774 | - | -4843 | -6979 | UPSTREAM | CG4774-RB | "-" | CG4774-RA | "-" | CG4774-RC | "-"
insitu CG31092 in-situ | CG31092 | - | 48252 | 6553 | DOWNSTREAM | CG31092-RA | "-" | CG31092-RB | "-"

*********************** Rank 787 [Score 15.281555]   GBROWSE*******************

 CG12540 in-situ | CG12540 | + | -14249 | -13144 | DOWNSTREAM | CG12540-RA | "-"
insitu CG14414 in-situ | CG14414 | + | 70222 | 71788 | UPSTREAM | CG14414-RA | "-" | CG14414-RC | "-" | CG14414-RB | "-"


*********************** Rank 788 [Score 15.278931]   GBROWSE*******************

insitu CG32498 in-situ | dnc | + | -43920 | 118984 | INTRAGENIC | intron:CG32498-RB:1 | intron:CG32498-RO:1 | CG32498-RB | "-" | CG32498-RO | "-" | CG32498-RI | "-" | CG32498-RC | "-" | CG32498-RJ | "-" | CG32498-RK | "-" | CG32498-RD | "-" | CG32498-RM | "-" | CG32498-RA | "-" | CG32498-RE | "-" | CG32498-RN | "-" | CG32498-RG | "-" | CG32498-RF | "-" | CG32498-RL | "-"
 CG14265 in-situ | EG:96G10.8 | - | 16320 | 15762 | DOWNSTREAM | CG14265-RB | "-"


*********************** Rank 789 [Score 15.276733]   GBROWSE*******************

insitu highlight CG2102 in-situ | cas | - | -5631 | -9989 | UPSTREAM | CG2102-RA | "-" | CG2102-RB | "-"
 CG1239 in-situ | CG1239 | + | 10610 | 11771 | UPSTREAM | CG1239-RA | "-"


*********************** Rank 790 [Score 15.263672]   GBROWSE*******************

insitu CG31243 in-situ | cpo | + | -20690 | 63231 | INTRAGENIC | intron:CG31243-RA:2 | intron:CG31243-RE:2 | intron:CG31243-RF:2 | intron:CG31243-RB:2 | CG31243-RA | "-" | CG31243-RE | "-" | CG31243-RF | "-" | CG31243-RB | "-"
 CG7780 in-situ | DNaseII | + | 66814 | 68598 | UPSTREAM | CG7780-RA | "-"

*********************** Rank 791 [Score 15.262451]   GBROWSE*******************

 CG10037 in-situ | vvl | + | -88611 | -84235 | DOWNSTREAM | CG10037-RA | "-"
insitu CG10078 in-situ | Prat2 | - | 36087 | 33222 | DOWNSTREAM | CG10078-RB | "-" | CG10078-RA | "-"

*********************** Rank 792 [Score 15.259094]   GBROWSE*******************

 CG33158 in-situ | CG33158 | + | -29777 | 46999 | INTRAGENIC | intron:CG33158-RB:3 | CG33158-RB | "-"
insitu CG32159 in-situ | CG32159 | - | 2383 | -26777 | INTRAGENIC | intron:CG32159-RB:1 | CG32159-RB | "-"

*********************** Rank 793 [Score 15.255127]   GBROWSE*******************

 CG30103 in-situ | CG30103 | + | -3813 | -1764 | DOWNSTREAM | CG30103-RA | "-"
insitu CG33006 in-situ | CG33006 | + | 5153 | 11072 | UPSTREAM | CG33006-RE | "-" | CG33006-RD | "-" | CG33006-RF | "-"

*********************** Rank 794 [Score 15.254944]   GBROWSE*******************

 CG30472 in-situ | CG30472 | - | -5460 | -6093 | UPSTREAM | CG30472-RA | "-"
 CG12959 in-situ | CG12959 | + | 27035 | 27632 | UPSTREAM | CG12959-RA | "-"

*********************** Rank 795 [Score 15.250488]   GBROWSE*******************

 CG7450 in-situ | CrebA | + | -7707 | 10093 | INTRAGENIC | intron:CG7450-RA:2 | CG7450-RA | "-"
 CG7439 in-situ | AGO2 | + | 10996 | 17925 | UPSTREAM | CG7439-RB | "-" | CG7439-RC | "-"

*********************** Rank 796 [Score 15.246094]   GBROWSE*******************

insitu CG6904 in-situ | CG6904 | - | -26153 | -31920 | UPSTREAM | CG6904-RC | "-" | CG6904-RB | "-" | CG6904-RA | "-"
insitu CG6803 in-situ | CG6803 | - | 6510 | -3135 | INTRAGENIC | intron:CG6803-RB:6 | intron:CG6803-RD:6 | intron:CG6803-RE:5 | CG6803-RA | "-" | CG6803-RB | "-" | CG6803-RC | "-" | CG6803-RD | "-" | CG6803-RE | "-"

*********************** Rank 797 [Score 15.237976]   GBROWSE*******************

 CG4399 in-situ | east | + | -17454 | -7120 | DOWNSTREAM | CG4399-RB | "-"
 CG4380 in-situ | usp | - | 11505 | 9018 | DOWNSTREAM | CG4380-RA | "-"


*********************** Rank 798 [Score 15.237366]   GBROWSE*******************

 CG32395 in-situ | Gr65a | - | -15744 | -17033 | UPSTREAM | CG32395-RA | "-"
insitu CG10121 in-situ | SP1173 | - | 19848 | 5654 | DOWNSTREAM | CG10121-RC | "-" | CG10121-RD | "-" | CG10121-RA | "-" | CG10121-RB | "-"

*********************** Rank 799 [Score 15.236694]   GBROWSE*******************

insitu highlight CG10021 in-situ | bowl | + | -5525 | 6898 | INTRAGENIC | intron:CG10021-RB:2 | intron:CG10021-RC:2 | intron:CG10021-RD:2 | CG10021-RB | "-" | CG10021-RC | "-" | CG10021-RD | "-" | CG10021-RA | "-"
 CG31960 in-situ | CG31960 | + | 7715 | 8284 | UPSTREAM | CG31960-RA | "-"

*********************** Rank 800 [Score 15.226929]   GBROWSE*******************

 CG10910 in-situ | CG10910 | + | -3267 | -1110 | DOWNSTREAM | CG10910-RA | "-" | CG10910-RB | "-"
insitu CG5773 in-situ | CG5773 | - | 20228 | 19388 | DOWNSTREAM | CG5773-RA | "-"


*********************** Rank 801 [Score 15.221375]   GBROWSE*******************

 CG17330 in-situ | BG:DS09218.5 | + | -12297 | -11239 | DOWNSTREAM | CG17330-RA | "-"
 CG4472 in-situ | Idgf1 | + | 67064 | 68631 | UPSTREAM | CG4472-RA | "-"

*********************** Rank 802 [Score 15.213379]   GBROWSE*******************

 CG32626 in-situ | CG32626 | - | -811 | -15895 | UPSTREAM | CG32626-RA | "-" | CG32626-RB | "-" | CG32626-RD | "-" | CG32626-RC | "-"
 CG11071 in-situ | CG11071 | - | 10483 | 7458 | DOWNSTREAM | CG11071-RA | "-"


*********************** Rank 803 [Score 15.209106]   GBROWSE*******************

 CG30084 in-situ | CG30084 | - | -8862 | -61210 | UPSTREAM | CG30084-RA | "-" | CG30084-RB | "-" | CG30084-RC | "-" | CG30084-RD | "-"
 CG8246 in-situ | Poxn | - | 11000 | 2918 | DOWNSTREAM | CG8246-RA | "-"


*********************** Rank 804 [Score 15.208984]   GBROWSE*******************

 CG16779 in-situ | CG16779 | - | -11529 | -20822 | UPSTREAM | CG16779-RA | "-"
 CG8147 in-situ | CG8147 | + | 7491 | 9418 | UPSTREAM | CG8147-RA | "-"


*********************** Rank 805 [Score 15.208557]   GBROWSE*******************

 CG8454 in-situ | CG8454 | + | -39730 | -36526 | DOWNSTREAM | CG8454-RA | "-"
 CG8120 in-situ | CG8120 | + | 15064 | 15588 | UPSTREAM | CG8120-RA | "-"

*********************** Rank 806 [Score 15.197876]   GBROWSE*******************

insitu CG31361 in-situ | CG31361 | + | -18431 | -1887 | DOWNSTREAM | CG31361-RA | "-" | CG31361-RB | "-"
insitu highlight CG4702 in-situ | CG4702 | - | 14888 | 8268 | DOWNSTREAM | CG4702-RA | "-"


*********************** Rank 807 [Score 15.193237]   GBROWSE*******************

insitu CG5820 in-situ | Gp150 | + | -4737 | 7029 | INTRAGENIC | intron:CG5820-RA:2 | intron:CG5820-RB:2 | intron:CG5820-RC:1 | CG5820-RA | "-" | CG5820-RB | "-" | CG5820-RC | "-" | CG5820-RD | "-"
 CG3425 in-situ | T3dh | - | 9658 | 7327 | DOWNSTREAM | CG3425-RA | "-"

*********************** Rank 808 [Score 15.188599]   GBROWSE*******************

 CG14351 in-situ | CG14351 | + | -25825 | 26633 | INTRAGENIC | intron:CG14351-RA:2 | CG14351-RA | "-"
 CG10869 in-situ | CG10869 | - | 6286 | 3874 | DOWNSTREAM | CG10869-RA | "-"

*********************** Rank 809 [Score 15.184570]   GBROWSE*******************

insitu highlight CG4345 in-situ | grim | - | -25357 | -27052 | UPSTREAM | CG4345-RA | "-"
 CG4319 in-situ | rpr | - | 68632 | 67782 | DOWNSTREAM | CG4319-RA | "-"

*********************** Rank 810 [Score 15.173340]   GBROWSE*******************

 CG12610 in-situ | CG12610 | - | -13564 | -14371 | UPSTREAM | CG12610-RA | "-"
insitu CG6361 in-situ | CG6361 | - | 7654 | 4011 | DOWNSTREAM | CG6361-RA | "-"

*********************** Rank 811 [Score 15.166992]   GBROWSE*******************

 CG18363 in-situ | CG18363 | - | -11450 | -12558 | UPSTREAM | CG18363-RA | "-"
 CG12477 in-situ | CG12477 | - | 13406 | 12609 | DOWNSTREAM | CG12477-RA | "-"

*********************** Rank 812 [Score 15.166626]   GBROWSE*******************

 CG13988 in-situ | CG13988 | + | -2064 | -1813 | DOWNSTREAM | CG13988-RA | "-"
 CG31640 in-situ | CG31640 | + | 3908 | 16349 | UPSTREAM | CG31640-RA | "-"

*********************** Rank 813 [Score 15.162598]   GBROWSE*******************

insitu CG9468 in-situ | CG9468 | - | -42518 | -46109 | UPSTREAM | CG9468-RA | "-"
insitu highlight CG18024 in-situ | SoxN | + | 1856 | 5901 | UPSTREAM | CG18024-RA | "-"

*********************** Rank 814 [Score 15.161743]   GBROWSE*******************

insitu CG8740 in-situ | BcDNA:GH05582 | + | -16084 | 7687 | INTRAGENIC | intron:CG8740-RB:1 | intron:CG8740-RC:2 | intron:CG8740-RA:1 | CG8740-RB | "-" | CG8740-RC | "-" | CG8740-RA | "-"
 CG8746 in-situ | CG8746 | + | 40730 | 41874 | UPSTREAM | CG8746-RA | "-"

*********************** Rank 815 [Score 15.159180]   GBROWSE*******************

 CG6629 in-situ | CG6629 | - | -9954 | -10989 | UPSTREAM | CG6629-RA | "-"
 CG6633 in-situ | Ugt86Dd | - | 2780 | 1040 | DOWNSTREAM | CG6633-RA | "-"

*********************** Rank 816 [Score 15.157227]   GBROWSE*******************

 CG15380 in-situ | CG15380 | - | -5564 | -6504 | UPSTREAM | CG15380-RA | "-"
 CG12674 in-situ | CG12674 | + | 7092 | 8185 | UPSTREAM | CG12674-RA | "-"

*********************** Rank 817 [Score 15.156250]   GBROWSE*******************

 CG13374 in-situ | pcl | - | -16170 | -17426 | UPSTREAM | CG13374-RA | "-"
insitu CG3258 in-situ | ase | + | 6888 | 9574 | UPSTREAM | CG3258-RA | "-"


*********************** Rank 818 [Score 15.151123]   GBROWSE*******************

 CG31345 in-situ | CG31345 | - | -22880 | -28564 | UPSTREAM | CG31345-RA | "-"
 CG10134 in-situ | beat-Va | - | 3661 | 2309 | DOWNSTREAM | CG10134-RA | "-"

*********************** Rank 819 [Score 15.149414]   GBROWSE*******************

 CG3819 in-situ | CG3819 | - | -23315 | -24900 | UPSTREAM | CG3819-RA | "-"
insitu CG32204 in-situ | CG32204 | - | 615 | -23002 | INTRAGENIC | intron:CG32204-RA:1 | CG32204-RA | "-"


*********************** Rank 820 [Score 15.145081]   GBROWSE*******************

insitu CG18507 in-situ | BG:DS01368.1 | + | -36570 | -31244 | DOWNSTREAM | CG18507-RA | "-" | CG18507-RB | "-"
 CG7311 in-situ | BG:DS08249.2 | + | 6117 | 8494 | UPSTREAM | CG7311-RA | "-" | CG7311-RC | "-"

*********************** Rank 821 [Score 15.144409]   GBROWSE*******************

 CG12426 in-situ | CG12426 | - | -26670 | -27346 | UPSTREAM | CG12426-RA | "-"
 CG5017 in-situ | CG5017 | + | 13434 | 14770 | UPSTREAM | CG5017-RA | "-"

*********************** Rank 822 [Score 15.140747]   GBROWSE*******************

 CG13334 in-situ | CG13334 | + | -3191 | -1631 | DOWNSTREAM | CG13334-RA | "-"
 CG13335 in-situ | CG13335 | - | 12493 | 9648 | DOWNSTREAM | CG13335-RA | "-" | CG13335-RB | "-"

*********************** Rank 823 [Score 15.138306]   GBROWSE*******************

 CG14342 in-situ | CG14342 | + | -768 | -3 | DOWNSTREAM | CG14342-RA | "-"
 CG14343 in-situ | CG14343 | + | 14279 | 14955 | UPSTREAM | CG14343-RA | "-"

*********************** Rank 824 [Score 15.128296]   GBROWSE*******************

 CG31171 in-situ | CG31171 | - | -12944 | -13599 | UPSTREAM | CG31171-RA | "-"
 CG18389 in-situ | Eip93F | + | 29862 | 66424 | UPSTREAM | CG18389-RA | "-"

*********************** Rank 825 [Score 15.119873]   GBROWSE*******************

 CG1907 in-situ | CG1907 | + | -15078 | -13354 | DOWNSTREAM | CG1907-RA | "-"
 CG18741 in-situ | DopR2 | - | 25872 | -3811 | INTRAGENIC | intron:CG18741-RA:4 | intron:CG18741-RB:3 | CG18741-RA | "-" | CG18741-RB | "-"

*********************** Rank 826 [Score 15.119202]   GBROWSE*******************

insitu CG6769 in-situ | CG6769 | + | -6363 | -4589 | DOWNSTREAM | CG6769-RA | "-"
 CG7835 in-situ | CG7835 | - | 7749 | 7097 | DOWNSTREAM | CG7835-RA | "-"

*********************** Rank 827 [Score 15.115051]   GBROWSE*******************

insitu CG32139 in-situ | Sox21b | - | -25746 | -44687 | UPSTREAM | CG32139-RA | "-"
insitu highlight CG5893 in-situ | D | - | 21550 | 18531 | DOWNSTREAM | CG5893-RA | "-"

*********************** Rank 828 [Score 15.111206]   GBROWSE*******************

insitu highlight CG3340 in-situ | Kr | + | -10115 | -7196 | DOWNSTREAM | CG3340-RA | "-"
 CG30429 in-situ | CG30429 | + | 9799 | 10774 | UPSTREAM | CG30429-RA | "-"

*********************** Rank 829 [Score 15.108887]   GBROWSE*******************

 CG13884 in-situ | CG13884 | - | -3633 | -4407 | UPSTREAM | CG13884-RA | "-"
insitu CG6883 in-situ | trh | - | 7246 | -3547 | INTRAGENIC | intron:CG6883-RA:6 | CG6883-RA | "-"


*********************** Rank 830 [Score 15.104004]   GBROWSE*******************

 CG31738 in-situ | CG31738 | + | -6472 | 33510 | INTRAGENIC | intron:CG31738-RB:1 | CG31738-RB | "-" | CG31738-RA | "-"
 CG5996 in-situ | trpgamma | - | 48150 | 36345 | DOWNSTREAM | CG5996-RA | "-" | CG5996-RB | "-"

*********************** Rank 831 [Score 15.098389]   GBROWSE*******************

 CG11312 in-situ | insc | - | -4242 | -19040 | UPSTREAM | CG11312-RA | "-"
 CG17999 in-situ | CG17999 | - | 9613 | 7674 | DOWNSTREAM | CG17999-RA | "-"

*********************** Rank 832 [Score 15.093506]   GBROWSE*******************

insitu CG2819 in-situ | Pph13 | + | -12197 | -10136 | DOWNSTREAM | CG2819-RA | "-"
insitu highlight CG2851 in-situ | Gsc | - | 5002 | -6145 | INTRAGENIC | intron:CG2851-RA:2 | CG2851-RA | "-"

*********************** Rank 833 [Score 15.092407]   GBROWSE*******************

insitu highlight CG32423 in-situ | CG32423 | - | -49898 | -167166 | UPSTREAM | CG32423-RA | "-" | CG32423-RD | "-" | CG32423-RB | "-" | CG32423-RC | "-"
 CG10645 in-situ | lama | - | 28081 | 15773 | DOWNSTREAM | CG10645-RC | "-" | CG10645-RB | "-" | CG10645-RA | "-"

*********************** Rank 834 [Score 15.086792]   GBROWSE*******************

 CG6559 in-situ | CG6559 | - | -858 | -17096 | UPSTREAM | CG6559-RA | "-"
 CG12362 in-situ | CG12362 | + | 99245 | 101206 | UPSTREAM | CG12362-RB | "-" | CG12362-RA | "-"

*********************** Rank 835 [Score 15.084106]   GBROWSE*******************

 CG14597 in-situ | CG14597 | - | -21868 | -22509 | UPSTREAM | CG14597-RA | "-"
 CG31146 in-situ | CG31146 | + | 32640 | 68709 | UPSTREAM | CG31146-RD | "-"


*********************** Rank 836 [Score 15.080200]   GBROWSE*******************

 CG32569 in-situ | CG32569 | + | -741 | -70 | DOWNSTREAM | CG32569-RA | "-"
 CG32568 in-situ | CG32568 | + | 2056 | 3296 | UPSTREAM | CG32568-RA | "-"

*********************** Rank 837 [Score 15.078857]   GBROWSE*******************

 CG5485 in-situ | CG5485 | - | -1018 | -3502 | UPSTREAM | CG5485-RA | "-"
 CG14353 in-situ | CG14353 | + | 5483 | 6666 | UPSTREAM | CG14353-RA | "-"

*********************** Rank 838 [Score 15.073425]   GBROWSE*******************

 CG30131 in-situ | CG30131 | + | -8382 | -5061 | DOWNSTREAM | CG30131-RA | "-" | CG30131-RB | "-" | CG30131-RC | "-" | CG30131-RD | "-"
 CG11242 in-situ | CG11242 | - | 16005 | 14661 | DOWNSTREAM | CG11242-RA | "-"

*********************** Rank 839 [Score 15.072083]   GBROWSE*******************

 CG5119 in-situ | pAbp | + | -3288 | 2872 | INTRAGENIC | intron:CG5119-RC:2 | intron:CG5119-RE:2 | intron:CG5119-RA:2 | intron:CG5119-RF:2 | intron:CG5119-RB:2 | intron:CG5119-RD:2 | intron:CG5119-RG:1 | CG5119-RC | "-" | CG5119-RE | "-" | CG5119-RA | "-" | CG5119-RF | "-" | CG5119-RB | "-" | CG5119-RD | "-" | CG5119-RG | "-"
 CG17680 in-situ | CG17680 | - | 4628 | 3931 | DOWNSTREAM | CG17680-RA | "-"

*********************** Rank 840 [Score 15.055908]   GBROWSE*******************

 CG6103 in-situ | CrebB-17A | + | -5108 | -1190 | DOWNSTREAM | CG6103-RA | "-" | CG6103-RB | "-"
insitu CG6179 in-situ | CG6179 | + | 2309 | 4248 | UPSTREAM | CG6179-RA | "-"


*********************** Rank 841 [Score 15.054871]   GBROWSE*******************

 CG11401 in-situ | Trxr-2 | + | -25646 | -23850 | DOWNSTREAM | CG11401-RA | "-"
 CG14459 in-situ | CG14459 | - | 7781 | 7042 | DOWNSTREAM | CG14459-RA | "-"

*********************** Rank 842 [Score 15.050171]   GBROWSE*******************

 CG31750 in-situ | Gr36d | + | -31129 | -29413 | DOWNSTREAM | CG31750-RA | "-"
 CG31745 in-situ | CG31745 | + | 32483 | 33416 | UPSTREAM | CG31745-RA | "-"


*********************** Rank 843 [Score 15.048706]   GBROWSE*******************

 CG14063 in-situ | CG14063 | + | -4749 | -4130 | DOWNSTREAM | CG14063-RA | "-"
insitu highlight CG10002 in-situ | fkh | - | 25971 | 22704 | DOWNSTREAM | CG10002-RA | "-"


*********************** Rank 844 [Score 15.044495]   GBROWSE*******************

 CG32336 in-situ | CG32336 | - | -24335 | -25038 | UPSTREAM | CG32336-RA | "-"
 CG9102 in-situ | bab2 | - | 33953 | -2872 | INTRAGENIC | intron:CG9102-RA:4 | CG9102-RA | "-"

*********************** Rank 845 [Score 15.044312]   GBROWSE*******************

insitu CG32139 in-situ | Sox21b | - | -18496 | -37437 | UPSTREAM | CG32139-RA | "-"
insitu highlight CG5893 in-situ | D | - | 28800 | 25781 | DOWNSTREAM | CG5893-RA | "-"


*********************** Rank 846 [Score 15.043884]   GBROWSE*******************

insitu CG4779 in-situ | hgo | + | -6346 | -4174 | DOWNSTREAM | CG4779-RA | "-"
 CG31867 in-situ | CG31867 | - | 2676 | 1838 | DOWNSTREAM | CG31867-RA | "-"

*********************** Rank 847 [Score 15.043579]   GBROWSE*******************

insitu highlight CG4531 in-situ | argos | - | -7420 | -20742 | UPSTREAM | CG4531-RA | "-"
insitu CG4314 in-situ | st | + | 6533 | 9346 | UPSTREAM | CG4314-RA | "-"


*********************** Rank 848 [Score 15.041748]   GBROWSE*******************

insitu highlight CG2988 in-situ | ems | + | -25400 | -22635 | DOWNSTREAM | CG2988-RA | "-"
 CG9929 in-situ | CG9929 | - | 16143 | 15089 | DOWNSTREAM | CG9929-RA | "-"

*********************** Rank 849 [Score 15.040283]   GBROWSE*******************

 CG3578 in-situ | bi | + | -37628 | 33980 | INTRAGENIC | intron:CG3578-RA:2 | CG3578-RA | "-"
 CG12685 in-situ | CG12685 | + | 65130 | 65698 | UPSTREAM | CG12685-RA | "-"

*********************** Rank 850 [Score 15.040283]   GBROWSE*******************

insitu CG31762 in-situ | aret | + | -1236 | 105722 | INTRAGENIC | intron:CG31762-RB:1 | CG31762-RB | "-" | CG31762-RA | "-" | CG31762-RC | "-" | CG31762-RD | "-"
 CG31759 in-situ | CG31759 | + | 548 | 3040 | UPSTREAM | CG31759-RB | "-" | CG31759-RA | "-"

*********************** Rank 851 [Score 15.036865]   GBROWSE*******************

 CG7720 in-situ | CG7720 | - | -44057 | -79977 | UPSTREAM | CG7720-RB | "-" | CG7720-RA | "-"
 CG18208 in-situ | CG18208 | - | 9180 | -33060 | INTRAGENIC | intron:CG18208-RA:1 | CG18208-RA | "-"

*********************** Rank 852 [Score 15.034912]   GBROWSE*******************

 CG8086 in-situ | CG8086 | - | -1917 | -14874 | UPSTREAM | CG8086-RA | "-" | CG8086-RB | "-"
insitu highlight CG8049 in-situ | Btk29A | - | 41653 | 85 | DOWNSTREAM | CG8049-RB | "-" | CG8049-RD | "-" | CG8049-RC | "-" | CG8049-RA | "-"

*********************** Rank 853 [Score 15.032104]   GBROWSE*******************

 CG6414 in-situ | CG6414 | - | -8436 | -10786 | UPSTREAM | CG6414-RA | "-"
 CG32790 in-situ | CG32790 | + | 98132 | 99430 | UPSTREAM | CG32790-RA | "-"

*********************** Rank 854 [Score 15.028564]   GBROWSE*******************

insitu CG7097 in-situ | CG7097 | + | -10176 | 38270 | INTRAGENIC | intron:CG7097-RB:2 | intron:CG7097-RA:2 | CG7097-RB | "-" | CG7097-RA | "-"
 CG7137 in-situ | CG7137 | + | 38400 | 39687 | UPSTREAM | CG7137-RA | "-"

*********************** Rank 855 [Score 15.024292]   GBROWSE*******************

 CG9328 in-situ | CG9328 | + | -14677 | 1642 | INTRAGENIC | intron:CG9328-RB:1 | intron:CG9328-RA:1 | CG9328-RB | "-" | CG9328-RA | "-"
 CG9329 in-situ | CG9329 | - | 6124 | 1923 | DOWNSTREAM | CG9329-RA | "-"

*********************** Rank 856 [Score 15.023193]   GBROWSE*******************

 CG13790 in-situ | CG13790 | - | -301 | -585 | UPSTREAM | CG13790-RA | "-"
 CG13791 in-situ | CG13791 | - | 9440 | 9114 | DOWNSTREAM | CG13791-RA | "-"


*********************** Rank 857 [Score 15.010742]   GBROWSE*******************

 CG13647 in-situ | CG13647 | + | -24359 | -22761 | DOWNSTREAM | CG13647-RA | "-"
 CG31422 in-situ | CG31422 | - | 3343 | -9862 | INTRAGENIC | intron:CG31422-RA:1 | CG31422-RA | "-"

*********************** Rank 858 [Score 15.006226]   GBROWSE*******************

 CG13788 in-situ | Gr28b | - | -1822 | -9061 | UPSTREAM | CG13788-RA | "-"
insitu CG6055 in-situ | CG6055 | + | 2481 | 8136 | UPSTREAM | CG6055-RA | "-"

*********************** Rank 859 [Score 14.993164]   GBROWSE*******************

 CG11286 in-situ | CG11286 | - | -10916 | -11656 | UPSTREAM | CG11286-RA | "-"
 CG18747 in-situ | CG18747 | - | 999 | -1673 | INTRAGENIC | intron:CG18747-RA:3 | CG18747-RA | "-"


*********************** Rank 860 [Score 14.992432]   GBROWSE*******************

 CG17025 in-situ | CG17025 | - | -1703 | -9474 | UPSTREAM | CG17025-RA | "-"
 CG12538 in-situ | CG12538 | - | 55726 | 55142 | DOWNSTREAM | CG12538-RA | "-"


*********************** Rank 861 [Score 14.992371]   GBROWSE*******************

 CG8180 in-situ | CG8180 | - | -592 | -10960 | UPSTREAM | CG8180-RA | "-"
 CG12964 in-situ | CG12964 | - | 6685 | 2836 | DOWNSTREAM | CG12964-RA | "-"

*********************** Rank 862 [Score 14.991394]   GBROWSE*******************

 CG12377 in-situ | CG12377 | - | -11289 | -13528 | UPSTREAM | CG12377-RA | "-"
 CG11404 in-situ | CG11404 | + | 31915 | 32785 | UPSTREAM | CG11404-RA | "-"


*********************** Rank 863 [Score 14.988403]   GBROWSE*******************

 CG14113 in-situ | CG14113 | + | -964 | -477 | DOWNSTREAM | CG14113-RA | "-"
 CG17687 in-situ | CG17687 | + | 19044 | 24667 | UPSTREAM | CG17687-RA | "-"


*********************** Rank 864 [Score 14.986389]   GBROWSE*******************

 CG9978 in-situ | CG9978 | + | -2154 | -787 | DOWNSTREAM | CG9978-RA | "-"
 CG13085 in-situ | CG13085 | + | 5855 | 9803 | UPSTREAM | CG13085-RA | "-"

*********************** Rank 865 [Score 14.983154]   GBROWSE*******************

 CG18341 in-situ | CG18341 | + | -4919 | -3868 | DOWNSTREAM | CG18341-RA | "-"
 CG15766 in-situ | CG15766 | - | 1841 | 1191 | DOWNSTREAM | CG15766-RA | "-"

*********************** Rank 866 [Score 14.981995]   GBROWSE*******************

insitu CG12701 in-situ | CG12701 | - | -25862 | -32803 | UPSTREAM | CG12701-RB | "-" | CG12701-RA | "-"
 CG12700 in-situ | skpD | + | 3939 | 4598 | UPSTREAM | CG12700-RA | "-"

*********************** Rank 867 [Score 14.970886]   GBROWSE*******************

insitu CG7962 in-situ | CdsA | + | -1743 | 1835 | INTRAGENIC | intron:CG7962-RA:4 | CG7962-RA | "-"
 CG7504 in-situ | CG7504 | - | 7373 | 1957 | DOWNSTREAM | CG7504-RA | "-"

Polluted


*********************** Rank 868 [Score 14.963013]   GBROWSE*******************

 CG31176 in-situ | CG31176 | - | -6406 | -53768 | UPSTREAM | CG31176-RA | "-"
insitu CG5849 in-situ | CG5849 | + | 14041 | 18367 | UPSTREAM | CG5849-RA | "-"

*********************** Rank 869 [Score 14.957397]   GBROWSE*******************

 CG31856 in-situ | CG31856 | - | -9385 | -11690 | UPSTREAM | CG31856-RA | "-"
 CG5142 in-situ | CG5142 | + | 23655 | 26179 | UPSTREAM | CG5142-RA | "-"


*********************** Rank 870 [Score 14.954834]   GBROWSE*******************

 CG4099 in-situ | Sr-CI | + | -20860 | -18579 | DOWNSTREAM | CG4099-RA | "-"
 CG2955 in-situ | CG2955 | + | 6698 | 8622 | UPSTREAM | CG2955-RA | "-"

*********************** Rank 871 [Score 14.954834]   GBROWSE*******************

 CG11641 in-situ | CG11641 | + | -4833 | 4095 | INTRAGENIC | intron:CG11641-RA:5 | CG11641-RA | "-"
 CG2121 in-situ | CG2121 | - | 19022 | 4190 | DOWNSTREAM | CG2121-RA | "-"

*********************** Rank 872 [Score 14.954590]   GBROWSE*******************

 CG2890 in-situ | PPP4R2r | + | -4457 | -1346 | DOWNSTREAM | CG2890-RA | "-" | CG2890-RB | "-"
insitu CG32687 in-situ | CG32687 | - | 3337 | -1262 | INTRAGENIC | intron:CG32687-RA:2 | CG32687-RA | "-"

*********************** Rank 873 [Score 14.952271]   GBROWSE*******************

 CG14323 in-situ | CG14323 | - | -1013 | -1645 | UPSTREAM | CG14323-RA | "-"
 CG7629 in-situ | AttD | + | 2155 | 2975 | UPSTREAM | CG7629-RA | "-"

*********************** Rank 874 [Score 14.950623]   GBROWSE*******************

 CG4841 in-situ | CG4841 | + | -30738 | -24364 | DOWNSTREAM | CG4841-RA | "-"
 CG33179 in-situ | beat-IIIb | + | 43239 | 51626 | UPSTREAM | CG33179-RA | "-"

*********************** Rank 875 [Score 14.950562]   GBROWSE*******************

insitu CG12218 in-situ | mei-P26 | + | -12149 | -3739 | DOWNSTREAM | CG12218-RA | "-"
 CG12115 in-situ | CG12115 | - | 15165 | 13907 | DOWNSTREAM | CG12115-RA | "-"

*********************** Rank 876 [Score 14.947998]   GBROWSE*******************

insitu CG14048 in-situ | mRpL14 | - | -291 | -933 | UPSTREAM | CG14048-RA | "-"
 CG2841 in-situ | ptr | - | 9337 | 205 | DOWNSTREAM | CG2841-RB | "-" | CG2841-RC | "-" | CG2841-RA | "-"


*********************** Rank 877 [Score 14.944397]   GBROWSE*******************

insitu CG11051 in-situ | CG11051 | + | -20699 | -20004 | DOWNSTREAM | CG11051-RA | "-"
insitu CG14112 in-situ | CG14112 | + | 1353 | 1967 | UPSTREAM | CG14112-RA | "-"

*********************** Rank 878 [Score 14.943970]   GBROWSE*******************

insitu CG5799 in-situ | dve | + | -32186 | 10269 | INTRAGENIC | intron:CG5799-RA:2 | intron:CG5799-RD:2 | intron:CG5799-RC:3 | CG5799-RA | "-" | CG5799-RD | "-" | CG5799-RB | "-" | CG5799-RC | "-"
insitu CG5819 in-situ | CG5819 | + | 17310 | 20809 | UPSTREAM | CG5819-RA | "-" | CG5819-RB | "-"

*********************** Rank 879 [Score 14.929810]   GBROWSE*******************

insitu highlight CG2988 in-situ | ems | + | -39350 | -36585 | DOWNSTREAM | CG2988-RA | "-"
 CG9929 in-situ | CG9929 | - | 2193 | 1139 | DOWNSTREAM | CG9929-RA | "-"

*********************** Rank 880 [Score 14.928833]   GBROWSE*******************

insitu highlight CG8254 in-situ | exex | + | -14879 | -11503 | DOWNSTREAM | CG8254-RA | "-"
 CG8194 in-situ | RNaseX25 | + | 7524 | 9380 | UPSTREAM | CG8194-RA | "-"

*********************** Rank 881 [Score 14.924561]   GBROWSE*******************

 CG10534 in-situ | Lcp65Ag2 | - | -1042 | -1653 | UPSTREAM | CG10534-RA | "-"
 CG10530 in-situ | Lcp65Ag1 | - | 628 | 55 | DOWNSTREAM | CG10530-RA | "-"

Polluted


*********************** Rank 882 [Score 14.923828]   GBROWSE*******************

insitu highlight CG7771 in-situ | sim | + | -13000 | 7469 | INTRAGENIC | intron:CG7771-RB:1 | CG7771-RB | "-" | CG7771-RA | "-"
 CG7855 in-situ | timeout | + | 17892 | 93117 | UPSTREAM | CG7855-RA | "-"

note: overlaps known module sim_promoter by 500 bases (module coords: 8895531-8898331)

*********************** Rank 883 [Score 14.923828]   GBROWSE*******************

insitu highlight CG5893 in-situ | D | - | -4100 | -7119 | UPSTREAM | CG5893-RA | "-"
 CG5842 in-situ | CG5842 | - | 6702 | 3648 | DOWNSTREAM | CG5842-RA | "-"

*********************** Rank 884 [Score 14.923462]   GBROWSE*******************

 CG7370 in-situ | CG7370 | + | -24707 | -23196 | DOWNSTREAM | CG7370-RA | "-"
 CG7383 in-situ | eg | + | 14181 | 23459 | UPSTREAM | CG7383-RB | "-" | CG7383-RA | "-"

*********************** Rank 885 [Score 14.920898]   GBROWSE*******************

 CG31941 in-situ | Obp22a | - | -4533 | -5104 | UPSTREAM | CG31941-RA | "-"
 CG15355 in-situ | CG15355 | - | 2249 | 1089 | DOWNSTREAM | CG15355-RA | "-"

*********************** Rank 886 [Score 14.917908]   GBROWSE*******************

 CG33171 in-situ | CG33171 | + | -3246 | 35202 | INTRAGENIC | intron:CG33171-RC:1 | intron:CG33171-RE:1 | CG33171-RC | "-" | CG33171-RE | "-"
 CG17744 in-situ | CG17744 | - | 9410 | 8430 | DOWNSTREAM | CG17744-RA | "-"

*********************** Rank 887 [Score 14.917725]   GBROWSE*******************

 CG2899 in-situ | ksr | - | -696 | -4432 | UPSTREAM | CG2899-RA | "-"
 CG31550 in-situ | CG31550 | + | 951 | 4913 | UPSTREAM | CG31550-RB | "-" | CG31550-RA | "-"


*********************** Rank 888 [Score 14.910522]   GBROWSE*******************

 CG15532 in-situ | hdc | + | -71228 | 13007 | INTRAGENIC | intron:CG15532-RA:2 | intron:CG15532-RC:2 | CG15532-RA | "-" | CG15532-RC | "-" | CG15532-RB | "-"
 CG1469 in-situ | Fer2LCH | + | 38670 | 41422 | UPSTREAM | CG1469-RA | "-" | CG1469-RB | "-" | CG1469-RC | "-"

*********************** Rank 889 [Score 14.909058]   GBROWSE*******************

 CG33203 in-situ | CG33203 | - | -1911 | -20304 | UPSTREAM | CG33203-RC | "-"
 CG31049 in-situ | CG31049 | + | 982 | 4154 | UPSTREAM | CG31049-RA | "-"

*********************** Rank 890 [Score 14.908081]   GBROWSE*******************

 CG7109 in-situ | mts | + | -6626 | -1624 | DOWNSTREAM | CG7109-RA | "-"
 CG14537 in-situ | CG14537 | + | 6112 | 6893 | UPSTREAM | CG14537-RA | "-"

*********************** Rank 891 [Score 14.905945]   GBROWSE*******************

 CG8624 in-situ | melt | + | -3303 | 4914 | INTRAGENIC | intron:CG8624-RA:1 | CG8624-RA | "-"
 CG32390 in-situ | CG32390 | - | 6541 | 5955 | DOWNSTREAM | CG32390-RA | "-"

*********************** Rank 892 [Score 14.905518]   GBROWSE*******************

 CG1158 in-situ | Tim17b1 | + | -31686 | -30642 | DOWNSTREAM | CG1158-RA | "-"
 CG32464 in-situ | l(3)82Fd | - | 4073 | -58889 | INTRAGENIC | intron:CG32464-RD:2 | CG32464-RD | "-" | CG32464-RL | "-" | CG32464-RB | "-" | CG32464-RJ | "-" | CG32464-RK | "-" | CG32464-RE | "-" | CG32464-RF | "-" | CG32464-RI | "-" | CG32464-RC | "-" | CG32464-RH | "-" | CG32464-RA | "-"

*********************** Rank 893 [Score 14.899414]   GBROWSE*******************

 CG15544 in-situ | CG15544 | + | -18612 | -4428 | DOWNSTREAM | CG15544-RA | "-"
insitu highlight CG1378 in-situ | tll | + | 5906 | 7911 | UPSTREAM | CG1378-RA | "-"


note: overlaps known module tll_rescue by 500 bases (module coords: 26661256-26671461)

*********************** Rank 894 [Score 14.899353]   GBROWSE*******************

insitu CG32954 in-situ | CG32954 | + | -51493 | -48146 | DOWNSTREAM | CG32954-RA | "-" | CG32954-RB | "-" | CG32954-RC | "-" | CG32954-RG | "-" | CG32954-RH | "-" | CG32954-RF | "-" | CG32954-RD | "-" | CG32954-RE | "-"
insitu CG3479 in-situ | osp | - | 22266 | -67272 | INTRAGENIC | intron:CG3479-RA:1 | CG3479-RA | "-"

*********************** Rank 895 [Score 14.890625]   GBROWSE*******************

 CG15631 in-situ | CG15631 | - | -14573 | -16622 | UPSTREAM | CG15631-RA | "-"
 CG15630 in-situ | CG15630 | - | 45011 | -13484 | INTRAGENIC | intron:CG15630-RA:1 | CG15630-RA | "-"

*********************** Rank 896 [Score 14.889893]   GBROWSE*******************

 CG17689 in-situ | CG17689 | + | -8492 | -1434 | DOWNSTREAM | CG17689-RA | "-"
 CG10711 in-situ | CG10711 | - | 1875 | 414 | DOWNSTREAM | CG10711-RA | "-"

*********************** Rank 897 [Score 14.887085]   GBROWSE*******************

insitu highlight CG4491 in-situ | noc | + | -24286 | -21129 | DOWNSTREAM | CG4491-RA | "-"
 CG4218 in-situ | BG:DS04641.8 | - | 33927 | 32487 | DOWNSTREAM | CG4218-RA | "-"

*********************** Rank 898 [Score 14.885986]   GBROWSE*******************

 CG14273 in-situ | CG14273 | + | -3255 | -1709 | DOWNSTREAM | CG14273-RA | "-"
 CG7778 in-situ | CG7778 | + | 602 | 4178 | UPSTREAM | CG7778-RA | "-"


*********************** Rank 899 [Score 14.881836]   GBROWSE*******************

 CG14500 in-situ | CG14500 | + | -2137 | -1462 | DOWNSTREAM | CG14500-RA | "-"
 CG14501 in-situ | CG14501 | - | 521 | -920 | INTRAGENIC | intron:CG14501-RA:1 | CG14501-RA | "-"

*********************** Rank 900 [Score 14.879272]   GBROWSE*******************

 CG30084 in-situ | CG30084 | - | -18912 | -71260 | UPSTREAM | CG30084-RA | "-" | CG30084-RB | "-" | CG30084-RC | "-" | CG30084-RD | "-"
 CG8246 in-situ | Poxn | - | 950 | -7132 | INTRAGENIC | intron:CG8246-RA:1 | CG8246-RA | "-"

*********************** Rank 901 [Score 14.877319]   GBROWSE*******************

 CG6371 in-situ | hug | + | -14071 | -11183 | DOWNSTREAM | CG6371-RA | "-"
 CG6989 in-situ | CG6989 | - | 14627 | 12385 | DOWNSTREAM | CG6989-RA | "-"

*********************** Rank 902 [Score 14.875854]   GBROWSE*******************

 CG12682 in-situ | CG12682 | + | -6833 | -6120 | DOWNSTREAM | CG12682-RA | "-"
 CG12681 in-situ | CG12681 | + | 42845 | 44308 | UPSTREAM | CG12681-RA | "-"


*********************** Rank 903 [Score 14.875488]   GBROWSE*******************

 CG7527 in-situ | CadN2 | - | -78213 | -104212 | UPSTREAM | CG7527-RA | "-"
 CG5674 in-situ | CG5674 | + | 72521 | 84053 | UPSTREAM | CG5674-RA | "-" | CG5674-RB | "-" | CG5674-RC | "-"

*********************** Rank 904 [Score 14.875000]   GBROWSE*******************

 CG12726 in-situ | CG12726 | - | -42509 | -43178 | UPSTREAM | CG12726-RA | "-"
 CG15753 in-situ | CG15753 | + | 20612 | 23924 | UPSTREAM | CG15753-RA | "-"

*********************** Rank 905 [Score 14.870605]   GBROWSE*******************

 CG4928 in-situ | BcDNA:GH10120 | + | -9381 | 14832 | INTRAGENIC | intron:CG4928-RA:1 | CG4928-RA | "-" | CG4928-RB | "-"
 CG9089 in-situ | wus | - | 18247 | 15918 | DOWNSTREAM | CG9089-RA | "-"

*********************** Rank 906 [Score 14.864746]   GBROWSE*******************

 CG17762 in-situ | tomosyn | + | -5370 | 16354 | INTRAGENIC | intron:CG17762-RB:5 | intron:CG17762-RA:4 | intron:CG17762-RC:4 | intron:CG17762-RD:4 | CG17762-RB | "-" | CG17762-RA | "-" | CG17762-RC | "-" | CG17762-RD | "-"
 CG2028 in-situ | CkIalpha | + | 16793 | 20295 | UPSTREAM | CG2028-RB | "-" | CG2028-RC | "-" | CG2028-RA | "-"


*********************** Rank 907 [Score 14.859863]   GBROWSE*******************

 CG7013 in-situ | ARP-like | + | -997 | 394 | INTRAGENIC | intron:CG7013-RA:3 | CG7013-RA | "-"
 CG10311 in-situ | CG10311 | - | 6601 | 495 | DOWNSTREAM | CG10311-RA | "-"

*********************** Rank 908 [Score 14.851685]   GBROWSE*******************

insitu CG7802 in-situ | CG7802 | + | -8950 | -2262 | DOWNSTREAM | CG7802-RA | "-"
 CG31033 in-situ | CG31033 | - | 6993 | -784 | INTRAGENIC | intron:CG31033-RC:8 | intron:CG31033-RA:6 | intron:CG31033-RB:5 | CG31033-RC | "-" | CG31033-RA | "-" | CG31033-RB | "-"


*********************** Rank 909 [Score 14.842041]   GBROWSE*******************

insitu CG32139 in-situ | Sox21b | - | -17496 | -36437 | UPSTREAM | CG32139-RA | "-"
insitu highlight CG5893 in-situ | D | - | 29800 | 26781 | DOWNSTREAM | CG5893-RA | "-"

*********************** Rank 910 [Score 14.839233]   GBROWSE*******************

 CG1657 in-situ | CG1657 | + | -12749 | -4137 | DOWNSTREAM | CG1657-RA | "-"
 CG11759 in-situ | Kap | - | 1344 | -4024 | INTRAGENIC | intron:CG11759-RA:2 | CG11759-RA | "-"

*********************** Rank 911 [Score 14.838379]   GBROWSE*******************

 CG2595 in-situ | RacGAP84C | + | -14576 | -11877 | DOWNSTREAM | CG2595-RA | "-" | CG2595-RB | "-"
 CG32467 in-situ | CG32467 | - | 2663 | 2208 | DOWNSTREAM | CG32467-RA | "-"

*********************** Rank 912 [Score 14.837769]   GBROWSE*******************

 CG11192 in-situ | CG11192 | - | -41615 | -42424 | UPSTREAM | CG11192-RA | "-"
 CG33041 in-situ | CG33041 | + | 27697 | 74326 | UPSTREAM | CG33041-RA | "-"

*********************** Rank 913 [Score 14.833740]   GBROWSE*******************

 CG32626 in-situ | CG32626 | - | -3061 | -18145 | UPSTREAM | CG32626-RA | "-" | CG32626-RB | "-" | CG32626-RD | "-" | CG32626-RC | "-"
 CG11071 in-situ | CG11071 | - | 8233 | 5208 | DOWNSTREAM | CG11071-RA | "-"

*********************** Rank 914 [Score 14.829224]   GBROWSE*******************

insitu highlight CG7771 in-situ | sim | + | -8750 | 11719 | INTRAGENIC | intron:CG7771-RB:1 | CG7771-RB | "-" | CG7771-RA | "-"
 CG7855 in-situ | timeout | + | 22142 | 97367 | UPSTREAM | CG7855-RA | "-"

*********************** Rank 915 [Score 14.827698]   GBROWSE*******************

 CG15476 in-situ | CG15476 | - | -34508 | -34774 | UPSTREAM | CG15476-RA | "-"
 CG15477 in-situ | CG15477 | + | 7983 | 8501 | UPSTREAM | CG15477-RA | "-"

*********************** Rank 916 [Score 14.823792]   GBROWSE*******************

 CG31394 in-situ | CG31394 | - | -11254 | -11992 | UPSTREAM | CG31394-RA | "-"
insitu highlight CG17117 in-situ | hth | - | 98254 | -30570 | INTRAGENIC | intron:CG17117-RB:7 | intron:CG17117-RC:8 | intron:CG17117-RA:6 | CG17117-RD | "-" | CG17117-RB | "-" | CG17117-RC | "-" | CG17117-RA | "-"


*********************** Rank 917 [Score 14.813477]   GBROWSE*******************

 CG32512 in-situ | CG32512 | - | -13862 | -19000 | UPSTREAM | CG32512-RA | "-"
 CG32514 in-situ | CG32514 | + | 12460 | 30033 | UPSTREAM | CG32514-RA | "-" | CG32514-RB | "-"

*********************** Rank 918 [Score 14.812012]   GBROWSE*******************

 CG31082 in-situ | CG31082 | + | -3553 | -1112 | DOWNSTREAM | CG31082-RA | "-"
insitu CG5467 in-situ | CG5467 | + | 9759 | 16149 | UPSTREAM | CG5467-RA | "-"

*********************** Rank 919 [Score 14.805725]   GBROWSE*******************

 CG30111 in-situ | CG30111 | + | -10830 | -7451 | DOWNSTREAM | CG30111-RA | "-"
 CG11430 in-situ | olf186-F | + | 18270 | 35395 | UPSTREAM | CG11430-RB | "-" | CG11430-RC | "-" | CG11430-RA | "-"

*********************** Rank 920 [Score 14.802612]   GBROWSE*******************

 CG11071 in-situ | CG11071 | - | -17467 | -20492 | UPSTREAM | CG11071-RA | "-"
 CG32611 in-situ | CG32611 | - | 10921 | -5209 | INTRAGENIC | intron:CG32611-RB:3 | CG32611-RB | "-"

*********************** Rank 921 [Score 14.799438]   GBROWSE*******************

 CG5988 in-situ | CG5988 | - | -12207 | -13993 | UPSTREAM | CG5988-RA | "-"
 CG15059 in-situ | CG15059 | - | 4115 | 3493 | DOWNSTREAM | CG15059-RB | "-" | CG15059-RA | "-"

*********************** Rank 922 [Score 14.797974]   GBROWSE*******************

 CG10491 in-situ | vn | - | -5378 | -37302 | UPSTREAM | CG10491-RA | "-"
 CG5568 in-situ | CG5568 | + | 20510 | 22527 | UPSTREAM | CG5568-RA | "-"


*********************** Rank 923 [Score 14.797241]   GBROWSE*******************

 CG10148 in-situ | CG10148 | - | -13524 | -14623 | UPSTREAM | CG10148-RA | "-"
 CG8138 in-situ | CG8138 | + | 34817 | 36244 | UPSTREAM | CG8138-RA | "-"


*********************** Rank 924 [Score 14.797241]   GBROWSE*******************

 CG2022 in-situ | CG2022 | - | -82079 | -84166 | UPSTREAM | CG2022-RA | "-"
insitu highlight CG2530 in-situ | corto | - | 15855 | 12790 | DOWNSTREAM | CG2530-RA | "-"


*********************** Rank 925 [Score 14.797241]   GBROWSE*******************

 CG4429 in-situ | Rbp2 | + | -46782 | -43305 | DOWNSTREAM | CG4429-RA | "-" | CG4429-RB | "-"
 CG9906 in-situ | CG9906 | - | 29741 | 27822 | DOWNSTREAM | CG9906-RA | "-"


*********************** Rank 926 [Score 14.797241]   GBROWSE*******************

 CG31662 in-situ | Gr22a | - | -9118 | -10367 | UPSTREAM | CG31662-RA | "-"
 CG31933 in-situ | CG31933 | - | 19296 | 17235 | DOWNSTREAM | CG31933-RA | "-"


*********************** Rank 927 [Score 14.797241]   GBROWSE*******************

 CG7017 in-situ | CG7017 | - | -3344 | -5027 | UPSTREAM | CG7017-RA | "-"
 CG6933 in-situ | CG6933 | - | 4935 | 3040 | DOWNSTREAM | CG6933-RA | "-" | CG6933-RC | "-" | CG6933-RB | "-"


*********************** Rank 928 [Score 14.797241]   GBROWSE*******************

 CG12816 in-situ | CG12816 | + | -9644 | -8428 | DOWNSTREAM | CG12816-RA | "-"
 CG3996 in-situ | CG3996 | + | 5008 | 15361 | UPSTREAM | CG3996-RA | "-"


*********************** Rank 929 [Score 14.787964]   GBROWSE*******************

 CG31749 in-situ | CG31749 | + | -36203 | -35413 | DOWNSTREAM | CG31749-RA | "-"
 CG10305 in-situ | RpS26 | - | 20720 | 19981 | DOWNSTREAM | CG10305-RA | "-" | CG10305-RB | "-" | CG10305-RC | "-"

*********************** Rank 930 [Score 14.780396]   GBROWSE*******************

 CG3856 in-situ | Oamb | - | -5720 | -31243 | UPSTREAM | CG3856-RA | "-" | CG3856-RB | "-" | CG3856-RC | "-"
 CG31205 in-situ | CG31205 | + | 10159 | 12281 | UPSTREAM | CG31205-RA | "-"

*********************** Rank 931 [Score 14.776123]   GBROWSE*******************

insitu highlight CG16738 in-situ | slp1 | + | -8701 | -7244 | DOWNSTREAM | CG16738-RA | "-"
insitu highlight CG2939 in-situ | slp2 | + | 2461 | 4820 | UPSTREAM | CG2939-RA | "-"

*********************** Rank 932 [Score 14.775879]   GBROWSE*******************

insitu CG10704 in-situ | toe | - | -13335 | -23495 | UPSTREAM | CG10704-RA | "-"
 CG10488 in-situ | eyg | - | 22018 | 17029 | DOWNSTREAM | CG10488-RA | "-"

*********************** Rank 933 [Score 14.773865]   GBROWSE*******************

 CG17100 in-situ | stich1 | + | -8847 | 3240 | INTRAGENIC | intron:CG17100-RA:2 | CG17100-RA | "-"
 CG31147 in-situ | Mth-like-11 | + | 3935 | 5719 | UPSTREAM | CG31147-RA | "-"

*********************** Rank 934 [Score 14.769409]   GBROWSE*******************

 CG6936 in-situ | mth | - | -7811 | -11344 | UPSTREAM | CG6936-RA | "-" | CG6936-RB | "-"
 CG1231 in-situ | CG1231 | + | 1702 | 2944 | UPSTREAM | CG1231-RA | "-"

*********************** Rank 935 [Score 14.768677]   GBROWSE*******************

insitu highlight CG4125 in-situ | rst | - | -9097 | -30722 | UPSTREAM | CG4125-RA | "-"
 CG4116 in-situ | CG4116 | - | 84633 | 83839 | DOWNSTREAM | CG4116-RA | "-"

*********************** Rank 936 [Score 14.765015]   GBROWSE*******************

 CG31386 in-situ | CG31386 | - | -11802 | -37657 | UPSTREAM | CG31386-RA | "-"
 CG17216 in-situ | KP78b | - | 41381 | 39298 | DOWNSTREAM | CG17216-RA | "-"

*********************** Rank 937 [Score 14.762329]   GBROWSE*******************

 CG31749 in-situ | CG31749 | + | -41803 | -41013 | DOWNSTREAM | CG31749-RA | "-"
 CG10305 in-situ | RpS26 | - | 15120 | 14381 | DOWNSTREAM | CG10305-RA | "-" | CG10305-RB | "-" | CG10305-RC | "-"

*********************** Rank 938 [Score 14.756958]   GBROWSE*******************

 CG32175 in-situ | CG32175 | + | -15910 | -15260 | DOWNSTREAM | CG32175-RA | "-"
 CG6512 in-situ | CG6512 | - | 29503 | 25997 | DOWNSTREAM | CG6512-RA | "-" | CG6512-RB | "-"

*********************** Rank 939 [Score 14.755859]   GBROWSE*******************

 CG8997 in-situ | BG:DS00941.12 | - | -893 | -1782 | UPSTREAM | CG8997-RA | "-"
 CG7916 in-situ | BG:DS00941.13 | + | 559 | 1602 | UPSTREAM | CG7916-RA | "-"

*********************** Rank 940 [Score 14.754761]   GBROWSE*******************

 CG14247 in-situ | CG14247 | + | -17171 | -16644 | DOWNSTREAM | CG14247-RA | "-"
 CG5490 in-situ | Tl | + | 44030 | 87390 | UPSTREAM | CG5490-RB | "-" | CG5490-RA | "-"

*********************** Rank 941 [Score 14.749939]   GBROWSE*******************

 CG12602 in-situ | CG12602 | + | -36877 | -31668 | DOWNSTREAM | CG12602-RA | "-"
 CG14931 in-situ | CG14931 | + | 12847 | 13502 | UPSTREAM | CG14931-RA | "-"

*********************** Rank 942 [Score 14.748901]   GBROWSE*******************

 CG32725 in-situ | CG32725 | - | -12912 | -13639 | UPSTREAM | CG32725-RA | "-"
 CG1958 in-situ | CG1958 | + | 15831 | 16808 | UPSTREAM | CG1958-RA | "-"

*********************** Rank 943 [Score 14.746826]   GBROWSE*******************

 CG12623 in-situ | CG12623 | - | -2436 | -3146 | UPSTREAM | CG12623-RA | "-"
 CG1641 in-situ | sisA | - | 8735 | 7968 | DOWNSTREAM | CG1641-RA | "-"

*********************** Rank 944 [Score 14.745117]   GBROWSE*******************

 CG3578 in-situ | bi | + | -65178 | 6430 | INTRAGENIC | intron:CG3578-RA:4 | CG3578-RA | "-"
 CG12685 in-situ | CG12685 | + | 37580 | 38148 | UPSTREAM | CG12685-RA | "-"

*********************** Rank 945 [Score 14.731995]   GBROWSE*******************

insitu CG31361 in-situ | CG31361 | + | -21231 | -4687 | DOWNSTREAM | CG31361-RA | "-" | CG31361-RB | "-"
insitu highlight CG4702 in-situ | CG4702 | - | 12088 | 5468 | DOWNSTREAM | CG4702-RA | "-"


*********************** Rank 946 [Score 14.731140]   GBROWSE*******************

 CG32350 in-situ | CG32350 | - | -22483 | -25319 | UPSTREAM | CG32350-RA | "-"
 CG32230 in-situ | CG32230 | - | 87882 | 85601 | DOWNSTREAM | CG32230-RA | "-" | CG32230-RB | "-"

*********************** Rank 947 [Score 14.730286]   GBROWSE*******************

 CG31085 in-situ | CG31085 | + | -9858 | 3226 | INTRAGENIC | intron:CG31085-RB:5 | intron:CG31085-RA:3 | CG31085-RB | "-" | CG31085-RA | "-"
 CG14239 in-situ | CG14239 | - | 44025 | 43146 | DOWNSTREAM | CG14239-RA | "-"


*********************** Rank 948 [Score 14.725464]   GBROWSE*******************

 CG4746 in-situ | mab-2 | - | -16846 | -24164 | UPSTREAM | CG4746-RA | "-"
insitu CG4336 in-situ | rux | - | 7152 | 5721 | DOWNSTREAM | CG4336-RA | "-"


*********************** Rank 949 [Score 14.725220]   GBROWSE*******************

 CG9380 in-situ | CG9380 | - | -9193 | -14401 | UPSTREAM | CG9380-RA | "-" | CG9380-RB | "-"
insitu highlight CG3340 in-situ | Kr | + | 28585 | 31504 | UPSTREAM | CG3340-RA | "-"

*********************** Rank 950 [Score 14.707214]   GBROWSE*******************

insitu CG32139 in-situ | Sox21b | - | -4596 | -23537 | UPSTREAM | CG32139-RA | "-"
insitu highlight CG5893 in-situ | D | - | 42700 | 39681 | DOWNSTREAM | CG5893-RA | "-"


*********************** Rank 951 [Score 14.703125]   GBROWSE*******************

 CG11883 in-situ | CG11883 | + | -9953 | 17690 | INTRAGENIC | intron:CG11883-RB:2 | intron:CG11883-RA:2 | CG11883-RB | "-" | CG11883-RA | "-"
 CG16728 in-situ | CG16728 | - | 21893 | 18291 | DOWNSTREAM | CG16728-RA | "-"

*********************** Rank 952 [Score 14.702576]   GBROWSE*******************

insitu CG7891 in-situ | CG7891 | + | -6649 | -4417 | DOWNSTREAM | CG7891-RA | "-"
 CG9656 in-situ | grn | - | 48179 | 14087 | DOWNSTREAM | CG9656-RA | "-"


*********************** Rank 953 [Score 14.701721]   GBROWSE*******************

 CG3474 in-situ | BG:DS06238.4 | - | -17517 | -18235 | UPSTREAM | CG3474-RA | "-"
 CG15283 in-situ | BG:DS08340.1 | - | 8168 | 4729 | DOWNSTREAM | CG15283-RA | "-"


*********************** Rank 954 [Score 14.700073]   GBROWSE*******************

insitu CG8635 in-situ | CG8635 | - | -8691 | -10459 | UPSTREAM | CG8635-RA | "-"
insitu highlight CG2411 in-situ | ptc | + | 9769 | 23683 | UPSTREAM | CG2411-RA | "-" | CG2411-RB | "-"

*********************** Rank 955 [Score 14.697754]   GBROWSE*******************

 CG15452 in-situ | CG15452 | - | -26336 | -27214 | UPSTREAM | CG15452-RA | "-"
 CG32507 in-situ | CG32507 | - | 8868 | 8287 | DOWNSTREAM | CG32507-RA | "-"

*********************** Rank 956 [Score 14.697144]   GBROWSE*******************

 CG4969 in-situ | Wnt6 | + | -11371 | -9989 | DOWNSTREAM | CG4969-RA | "-"
 CG4971 in-situ | Wnt10 | + | 12493 | 14990 | UPSTREAM | CG4971-RA | "-"

*********************** Rank 957 [Score 14.693237]   GBROWSE*******************

 CG5481 in-situ | lea | - | -42685 | -82246 | UPSTREAM | CG5481-RA | "-"
 CG31925 in-situ | CG31925 | - | 10909 | 10216 | DOWNSTREAM | CG31925-RA | "-"


*********************** Rank 958 [Score 14.692688]   GBROWSE*******************

 CG32268 in-situ | CG32268 | - | -959 | -1177 | UPSTREAM | CG32268-RA | "-"
 CG12008 in-situ | kst | + | 14206 | 30815 | UPSTREAM | CG12008-RA | "-"

*********************** Rank 959 [Score 14.680786]   GBROWSE*******************

insitu highlight CG7250 in-situ | Toll-6 | + | -117621 | -112110 | DOWNSTREAM | CG7250-RA | "-"
 CG7804 in-situ | CG7804 | - | 19789 | 17876 | DOWNSTREAM | CG7804-RA | "-"


*********************** Rank 960 [Score 14.673096]   GBROWSE*******************

insitu highlight CG1897 in-situ | Dr | + | -12475 | -3577 | DOWNSTREAM | CG1897-RA | "-"
insitu CG7567 in-situ | CG7567 | - | 19238 | 18374 | DOWNSTREAM | CG7567-RA | "-"

*********************** Rank 961 [Score 14.670532]   GBROWSE*******************

 CG15214 in-situ | CG15214 | + | -15596 | -11600 | DOWNSTREAM | CG15214-RA | "-"
 CG4835 in-situ | CG4835 | + | 28944 | 32471 | UPSTREAM | CG4835-RA | "-"

*********************** Rank 962 [Score 14.669922]   GBROWSE*******************

 CG12673 in-situ | olf413 | + | -22307 | 36735 | INTRAGENIC | intron:CG12673-RA:1 | CG12673-RA | "-"
 CG9063 in-situ | BcDNA:GH03694 | - | 56823 | 51675 | DOWNSTREAM | CG9063-RA | "-"

*********************** Rank 963 [Score 14.669067]   GBROWSE*******************

 CG13251 in-situ | CG13251 | + | -47582 | -44017 | DOWNSTREAM | CG13251-RA | "-"
 CG32431 in-situ | CG32431 | + | 2930 | 3637 | UPSTREAM | CG32431-RA | "-"

*********************** Rank 964 [Score 14.667969]   GBROWSE*******************

 CG4815 in-situ | CG4815 | - | -1249 | -2046 | UPSTREAM | CG4815-RA | "-"
 CG12872 in-situ | CG12872 | + | 38876 | 39750 | UPSTREAM | CG12872-RA | "-"

*********************** Rank 965 [Score 14.666077]   GBROWSE*******************

 CG32118 in-situ | CG32118 | + | -7602 | -7225 | DOWNSTREAM | CG32118-RA | "-"
 CG11280 in-situ | trn | + | 8420 | 12235 | UPSTREAM | CG11280-RA | "-"


*********************** Rank 966 [Score 14.662231]   GBROWSE*******************

 CG4478 in-situ | Mst35Bb | - | -1501 | -3115 | UPSTREAM | CG4478-RA | "-"
 CG15277 in-situ | CG15277 | - | 24551 | 23305 | DOWNSTREAM | CG15277-RA | "-"


*********************** Rank 967 [Score 14.658569]   GBROWSE*******************

 CG10205 in-situ | CG10205 | + | -2241 | -1119 | DOWNSTREAM | CG10205-RB | "-" | CG10205-RA | "-"
insitu CG10207 in-situ | NaPi-T | - | 10800 | 7637 | DOWNSTREAM | CG10207-RA | "-"

*********************** Rank 968 [Score 14.657288]   GBROWSE*******************

 CG7325 in-situ | Eig71Ek | + | -11073 | -10518 | DOWNSTREAM | CG7325-RA | "-"
 CG7304 in-situ | CG7304 | + | 5912 | 9149 | UPSTREAM | CG7304-RA | "-"

*********************** Rank 969 [Score 14.656006]   GBROWSE*******************

 CG6391 in-situ | CG6391 | - | -27574 | -31274 | UPSTREAM | CG6391-RA | "-" | CG6391-RB | "-" | CG6391-RC | "-"
 CG14148 in-situ | CG14148 | - | 13595 | 13305 | DOWNSTREAM | CG14148-RA | "-"

*********************** Rank 970 [Score 14.645386]   GBROWSE*******************

insitu CG2493 in-situ | CG2493 | - | -18109 | -19973 | UPSTREAM | CG2493-RA | "-"
 CG15476 in-situ | CG15476 | - | 20592 | 20326 | DOWNSTREAM | CG15476-RA | "-"

*********************** Rank 971 [Score 14.638855]   GBROWSE*******************

 CG33197 in-situ | CG33197 | + | -49972 | 60344 | INTRAGENIC | intron:CG33197-RA:2 | intron:CG33197-RB:2 | intron:CG33197-RD:2 | CG33197-RA | "-" | CG33197-RB | "-" | CG33197-RC | "-" | CG33197-RD | "-"
 CG14477 in-situ | mm | + | 35569 | 60344 | UPSTREAM | CG14477-RA | "-"

*********************** Rank 972 [Score 14.630615]   GBROWSE*******************

 CG13111 in-situ | CG13111 | - | -3174 | -3740 | UPSTREAM | CG13111-RA | "-"
 CG4450 in-situ | CG4450 | - | 8319 | 704 | DOWNSTREAM | CG4450-RA | "-"

*********************** Rank 973 [Score 14.621948]   GBROWSE*******************

 CG10152 in-situ | beat-IV | + | -18980 | 3143 | INTRAGENIC | intron:CG10152-RA:5 | CG10152-RA | "-"
 CG10182 in-situ | CG10182 | - | 13394 | 10786 | DOWNSTREAM | CG10182-RA | "-"

*********************** Rank 974 [Score 14.613953]   GBROWSE*******************

 CG14298 in-situ | CG14298 | + | -11376 | -10239 | DOWNSTREAM | CG14298-RA | "-"
 CG14297 in-situ | CG14297 | + | 10957 | 12074 | UPSTREAM | CG14297-RA | "-"

*********************** Rank 975 [Score 14.612915]   GBROWSE*******************

 CG6069 in-situ | CG6069 | - | -22661 | -24796 | UPSTREAM | CG6069-RA | "-"
 CG12290 in-situ | CG12290 | + | 16389 | 19485 | UPSTREAM | CG12290-RA | "-"

*********************** Rank 976 [Score 14.611450]   GBROWSE*******************

 CG12689 in-situ | CG12689 | - | -17063 | -17632 | UPSTREAM | CG12689-RA | "-"
insitu CG11387 in-situ | ct | + | 6075 | 72950 | UPSTREAM | CG11387-RA | "-" | CG11387-RB | "-"

*********************** Rank 977 [Score 14.611389]   GBROWSE*******************

insitu CG4364 in-situ | CG4364 | - | -1352 | -3701 | UPSTREAM | CG4364-RA | "-"
 CG13116 in-situ | CG13116 | + | 17319 | 18328 | UPSTREAM | CG13116-RA | "-"

*********************** Rank 978 [Score 14.610840]   GBROWSE*******************

 CG8100 in-situ | CG8100 | + | -6889 | -4786 | DOWNSTREAM | CG8100-RA | "-"
 CG17285 in-situ | Fbp1 | - | 7536 | 4066 | DOWNSTREAM | CG17285-RA | "-" | CG17285-RB | "-"

*********************** Rank 979 [Score 14.610107]   GBROWSE*******************

insitu CG5799 in-situ | dve | + | -46436 | -3981 | DOWNSTREAM | CG5799-RA | "-" | CG5799-RD | "-" | CG5799-RB | "-" | CG5799-RC | "-"
insitu CG5819 in-situ | CG5819 | + | 3060 | 6559 | UPSTREAM | CG5819-RA | "-" | CG5819-RB | "-"


*********************** Rank 980 [Score 14.606812]   GBROWSE*******************

 CG31550 in-situ | CG31550 | + | -8299 | -4337 | DOWNSTREAM | CG31550-RB | "-" | CG31550-RA | "-"
insitu CG31547 in-situ | CG31547 | - | 8871 | -4326 | INTRAGENIC | intron:CG31547-RB:2 | intron:CG31547-RA:2 | CG31547-RB | "-" | CG31547-RA | "-"

*********************** Rank 981 [Score 14.606323]   GBROWSE*******************

insitu highlight CG7250 in-situ | Toll-6 | + | -11521 | -6010 | DOWNSTREAM | CG7250-RA | "-"
 CG7804 in-situ | CG7804 | - | 125889 | 123976 | DOWNSTREAM | CG7804-RA | "-"

*********************** Rank 982 [Score 14.604309]   GBROWSE*******************

 CG17762 in-situ | tomosyn | + | -8770 | 12954 | INTRAGENIC | intron:CG17762-RB:13 | intron:CG17762-RA:12 | intron:CG17762-RC:12 | intron:CG17762-RD:12 | CG17762-RB | "-" | CG17762-RA | "-" | CG17762-RC | "-" | CG17762-RD | "-"
 CG2028 in-situ | CkIalpha | + | 13393 | 16895 | UPSTREAM | CG2028-RB | "-" | CG2028-RC | "-" | CG2028-RA | "-"

*********************** Rank 983 [Score 14.603027]   GBROWSE*******************

 CG9031 in-situ | BG:BACR48E02.4 | - | -21527 | -23271 | UPSTREAM | CG9031-RA | "-"
insitu CG18507 in-situ | BG:DS01368.1 | + | 2730 | 8056 | UPSTREAM | CG18507-RA | "-" | CG18507-RB | "-"

*********************** Rank 984 [Score 14.594727]   GBROWSE*******************

 CG5206 in-situ | bon | + | -4398 | 14981 | INTRAGENIC | intron:CG5206-RA:1 | CG5206-RA | "-"
 CG15923 in-situ | CG15923 | - | 19336 | 15048 | DOWNSTREAM | CG15923-RA | "-"


*********************** Rank 985 [Score 14.594727]   GBROWSE*******************

 CG31481 in-situ | CG31481 | - | -20801 | -25619 | UPSTREAM | CG31481-RA | "-"
 CG1048 in-situ | zen2 | - | 10313 | 9305 | DOWNSTREAM | CG1048-RA | "-"


*********************** Rank 986 [Score 14.593384]   GBROWSE*******************

 CG31923 in-situ | CG31923 | + | -1456 | -768 | DOWNSTREAM | CG31923-RA | "-"
 CG4375 in-situ | CG4375 | - | 94763 | 93973 | DOWNSTREAM | CG4375-RA | "-"

*********************** Rank 987 [Score 14.587891]   GBROWSE*******************

 CG31353 in-situ | CG31353 | - | -22015 | -22320 | UPSTREAM | CG31353-RA | "-"
 CG6332 in-situ | CG6332 | + | 13583 | 15107 | UPSTREAM | CG6332-RA | "-"

*********************** Rank 988 [Score 14.586975]   GBROWSE*******************

insitu highlight CG1374 in-situ | tsh | + | -47634 | -39216 | DOWNSTREAM | CG1374-RA | "-"
 CG11629 in-situ | CG11629 | - | 22032 | 20641 | DOWNSTREAM | CG11629-RA | "-"

*********************** Rank 989 [Score 14.578613]   GBROWSE*******************

 CG14560 in-situ | msopa | + | -66414 | -65919 | DOWNSTREAM | CG14560-RA | "-"
 CG15374 in-situ | CG15374 | + | 16205 | 16699 | UPSTREAM | CG15374-RA | "-"

*********************** Rank 990 [Score 14.577026]   GBROWSE*******************

insitu highlight CG9598 in-situ | CG9598 | - | -57188 | -61697 | UPSTREAM | CG9598-RA | "-"
 CG9587 in-situ | CG9587 | - | 38728 | 33086 | DOWNSTREAM | CG9587-RA | "-"

*********************** Rank 991 [Score 14.572998]   GBROWSE*******************

insitu CG10078 in-situ | Prat2 | - | -13913 | -16778 | UPSTREAM | CG10078-RB | "-" | CG10078-RA | "-"
 CG14820 in-situ | CG14820 | + | 4159 | 6068 | UPSTREAM | CG14820-RA | "-"

*********************** Rank 992 [Score 14.567627]   GBROWSE*******************

 CG31069 in-situ | CG31069 | + | -10195 | -8792 | DOWNSTREAM | CG31069-RA | "-"
 CG31066 in-situ | CG31066 | + | 9854 | 10475 | UPSTREAM | CG31066-RA | "-"

*********************** Rank 993 [Score 14.565796]   GBROWSE*******************

 CG31759 in-situ | CG31759 | + | -105252 | -102760 | DOWNSTREAM | CG31759-RB | "-" | CG31759-RA | "-"
 CG31862 in-situ | CG31862 | + | 25991 | 26539 | UPSTREAM | CG31862-RA | "-"

*********************** Rank 994 [Score 14.563599]   GBROWSE*******************

 CG14247 in-situ | CG14247 | + | -19921 | -19394 | DOWNSTREAM | CG14247-RA | "-"
 CG5490 in-situ | Tl | + | 41280 | 84640 | UPSTREAM | CG5490-RB | "-" | CG5490-RA | "-"

*********************** Rank 995 [Score 14.562073]   GBROWSE*******************

 CG10881 in-situ | CG10881 | + | -8646 | -7763 | DOWNSTREAM | CG10881-RA | "-"
 CG17208 in-situ | CG17208 | - | 21013 | 20678 | DOWNSTREAM | CG17208-RA | "-"


*********************** Rank 996 [Score 14.561768]   GBROWSE*******************

 CG14280 in-situ | CG14280 | + | -29495 | -26879 | DOWNSTREAM | CG14280-RA | "-"
insitu CG3619 in-situ | Dl | - | 39452 | 15970 | DOWNSTREAM | CG3619-RA | "-" | CG3619-RB | "-"

*********************** Rank 997 [Score 14.558228]   GBROWSE*******************

insitu highlight CG2189 in-situ | Dfd | + | -28141 | -17546 | DOWNSTREAM | CG2189-RA | "-"
insitu highlight CG1030 in-situ | Scr | - | 28642 | 3143 | DOWNSTREAM | CG1030-RA | "-"


*********************** Rank 998 [Score 14.556396]   GBROWSE*******************

insitu CG1702 in-situ | CG1702 | + | -7415 | -6006 | DOWNSTREAM | CG1702-RA | "-"
 CG15461 in-situ | CG15461 | + | 10378 | 10805 | UPSTREAM | CG15461-RA | "-"


*********************** Rank 999 [Score 14.555054]   GBROWSE*******************

 CG10029 in-situ | CG10029 | - | -5340 | -6676 | UPSTREAM | CG10029-RA | "-"
 CG31496 in-situ | CG31496 | + | 22704 | 23769 | UPSTREAM | CG31496-RA | "-"

*********************** Rank 1000 [Score 14.554504]   GBROWSE*******************

 CG10579 in-situ | Eip63E | + | -62536 | 30330 | INTRAGENIC | intron:CG10579-RD:2 | intron:CG10579-RE:3 | intron:CG10579-RA:4 | intron:CG10579-RB:3 | intron:CG10579-RC:3 | CG10579-RD | "-" | CG10579-RE | "-" | CG10579-RA | "-" | CG10579-RB | "-" | CG10579-RC | "-"
 CG10359 in-situ | CG10359 | + | 33973 | 37024 | UPSTREAM | CG10359-RA | "-"


*********************** Rank 1001 [Score 14.552246]   GBROWSE*******************

 CG11401 in-situ | Trxr-2 | + | -15296 | -13500 | DOWNSTREAM | CG11401-RA | "-"
 CG14459 in-situ | CG14459 | - | 18131 | 17392 | DOWNSTREAM | CG14459-RA | "-"

*********************** Rank 1002 [Score 14.547729]   GBROWSE*******************

 CG14325 in-situ | CG14325 | + | -11986 | -10101 | DOWNSTREAM | CG14325-RA | "-"
 CG14322 in-situ | CG14322 | + | 21511 | 30104 | UPSTREAM | CG14322-RA | "-"

*********************** Rank 1003 [Score 14.545166]   GBROWSE*******************

 CG9820 in-situ | Or59a | - | -9267 | -10464 | UPSTREAM | CG9820-RA | "-"
 CG5357 in-situ | CG5357 | + | 5643 | 8278 | UPSTREAM | CG5357-RA | "-"

*********************** Rank 1004 [Score 14.540649]   GBROWSE*******************

 CG7313 in-situ | CG7313 | + | -33824 | -33075 | DOWNSTREAM | CG7313-RA | "-"
 CG5103 in-situ | CG5103 | - | 8446 | 6196 | DOWNSTREAM | CG5103-RA | "-"

*********************** Rank 1005 [Score 14.537598]   GBROWSE*******************

 CG31759 in-situ | CG31759 | + | -26152 | -23660 | DOWNSTREAM | CG31759-RB | "-" | CG31759-RA | "-"
 CG31862 in-situ | CG31862 | + | 105091 | 105639 | UPSTREAM | CG31862-RA | "-"

*********************** Rank 1006 [Score 14.536865]   GBROWSE*******************

 CG3587 in-situ | EG:39E1.2 | + | -15142 | -13837 | DOWNSTREAM | CG3587-RA | "-"
 CG32802 in-situ | CG32802 | - | 34751 | 33374 | DOWNSTREAM | CG32802-RA | "-"

*********************** Rank 1007 [Score 14.536377]   GBROWSE*******************

insitu CG18507 in-situ | BG:DS01368.1 | + | -24320 | -18994 | DOWNSTREAM | CG18507-RA | "-" | CG18507-RB | "-"
 CG7311 in-situ | BG:DS08249.2 | + | 18367 | 20744 | UPSTREAM | CG7311-RA | "-" | CG7311-RC | "-"


*********************** Rank 1008 [Score 14.536133]   GBROWSE*******************

insitu CG31337 in-situ | CG31337 | - | -33075 | -34487 | UPSTREAM | CG31337-RA | "-"
 CG14370 in-situ | CG14370 | + | 9728 | 10126 | UPSTREAM | CG14370-RA | "-"

*********************** Rank 1009 [Score 14.529663]   GBROWSE*******************

 CG11498 in-situ | CG11498 | - | -4653 | -8573 | UPSTREAM | CG11498-RA | "-"
 CG31030 in-situ | CG31030 | - | 4768 | 3027 | DOWNSTREAM | CG31030-RA | "-"


*********************** Rank 1010 [Score 14.529358]   GBROWSE*******************

 CG31050 in-situ | CG31050 | - | -9532 | -9931 | UPSTREAM | CG31050-RA | "-"
insitu CG9989 in-situ | CG9989 | + | 10724 | 12009 | UPSTREAM | CG9989-RA | "-"

*********************** Rank 1011 [Score 14.528198]   GBROWSE*******************

 CG12676 in-situ | ed | + | -79602 | 4078 | INTRAGENIC | intron:CG12676-RA:8 | CG12676-RA | "-"
 CG31962 in-situ | Sr-CIII | + | 9382 | 10461 | UPSTREAM | CG31962-RA | "-"

*********************** Rank 1012 [Score 14.515015]   GBROWSE*******************

 CG12478 in-situ | bru-3 | - | -114883 | -243714 | UPSTREAM | CG12478-RA | "-" | CG12478-RB | "-"
 CG8757 in-situ | CG8757 | - | 51920 | 50954 | DOWNSTREAM | CG8757-RA | "-"

*********************** Rank 1013 [Score 14.514282]   GBROWSE*******************

 CG31226 in-situ | CG31226 | - | -6957 | -7458 | UPSTREAM | CG31226-RA | "-" | CG31226-RB | "-"
 CG14298 in-situ | CG14298 | + | 17024 | 18161 | UPSTREAM | CG14298-RA | "-"

*********************** Rank 1014 [Score 14.513672]   GBROWSE*******************

insitu CG3352 in-situ | ft | - | -5379 | -24829 | UPSTREAM | CG3352-RA | "-"
 CG3702 in-situ | CG3702 | - | 3866 | 1419 | DOWNSTREAM | CG3702-RA | "-"

*********************** Rank 1015 [Score 14.512939]   GBROWSE*******************

 CG4695 in-situ | CG4695 | + | -44688 | -40460 | DOWNSTREAM | CG4695-RA | "-"
 CG6629 in-situ | CG6629 | - | 27046 | 26011 | DOWNSTREAM | CG6629-RA | "-"

*********************** Rank 1016 [Score 14.511230]   GBROWSE*******************

 CG11368 in-situ | CG11368 | + | -39565 | -39110 | DOWNSTREAM | CG11368-RA | "-"
 CG32719 in-situ | CG32719 | - | 21070 | 17118 | DOWNSTREAM | CG32719-RA | "-"

*********************** Rank 1017 [Score 14.509521]   GBROWSE*******************

 CG4683 in-situ | CG4683 | + | -29491 | -28136 | DOWNSTREAM | CG4683-RA | "-"
 CG14698 in-situ | CG14698 | + | 54685 | 55211 | UPSTREAM | CG14698-RA | "-"

*********************** Rank 1018 [Score 14.502563]   GBROWSE*******************

 CG14030 in-situ | CG14030 | + | -8928 | -4961 | DOWNSTREAM | CG14030-RA | "-"
 CG14029 in-situ | vri | + | 11668 | 23018 | UPSTREAM | CG14029-RC | "-" | CG14029-RA | "-" | CG14029-RB | "-"


*********************** Rank 1019 [Score 14.500854]   GBROWSE*******************

insitu CG32473 in-situ | CG32473 | + | -24806 | -14662 | DOWNSTREAM | CG32473-RA | "-" | CG32473-RC | "-" | CG32473-RB | "-"
 CG8795 in-situ | CG8795 | - | 6704 | 1606 | DOWNSTREAM | CG8795-RA | "-" | CG8795-RB | "-"

*********************** Rank 1020 [Score 14.493164]   GBROWSE*******************

 CG14532 in-situ | CG14532 | - | -58234 | -58827 | UPSTREAM | CG14532-RA | "-"
 CG7233 in-situ | CG7233 | - | 17921 | 16905 | DOWNSTREAM | CG7233-RA | "-"

*********************** Rank 1021 [Score 14.490845]   GBROWSE*******************

insitu CG33110 in-situ | CG33110 | + | -533 | 9676 | INTRAGENIC | intron:CG33110-RA:1 | CG33110-RA | "-"
 CG6932 in-situ | CSN6 | + | 10053 | 11316 | UPSTREAM | CG6932-RA | "-"

*********************** Rank 1022 [Score 14.488586]   GBROWSE*******************

insitu highlight CG6464 in-situ | salm | - | -20676 | -31968 | UPSTREAM | CG6464-RA | "-"
insitu highlight CG4922 in-situ | sala | + | 19910 | 20676 | UPSTREAM | CG4922-RA | "-"


*********************** Rank 1023 [Score 14.481323]   GBROWSE*******************

 CG17572 in-situ | CG17572 | - | -421 | -3375 | UPSTREAM | CG17572-RA | "-"
 CG10700 in-situ | CG10700 | - | 4428 | 2809 | DOWNSTREAM | CG10700-RA | "-"


*********************** Rank 1024 [Score 14.479980]   GBROWSE*******************

 CG32971 in-situ | CG32971 | + | -1018 | -581 | DOWNSTREAM | CG32971-RA | "-"
 CG32970 in-situ | CG32970 | + | 29659 | 32500 | UPSTREAM | CG32970-RA | "-"


*********************** Rank 1025 [Score 14.477295]   GBROWSE*******************

 CG7527 in-situ | CadN2 | - | -50763 | -76762 | UPSTREAM | CG7527-RA | "-"
 CG5674 in-situ | CG5674 | + | 99971 | 111503 | UPSTREAM | CG5674-RA | "-" | CG5674-RB | "-" | CG5674-RC | "-"

*********************** Rank 1026 [Score 14.476501]   GBROWSE*******************

insitu highlight CG7771 in-situ | sim | + | -13650 | 6819 | INTRAGENIC | intron:CG7771-RB:1 | CG7771-RB | "-" | CG7771-RA | "-"
 CG7855 in-situ | timeout | + | 17242 | 92467 | UPSTREAM | CG7855-RA | "-"


note: overlaps known module sim_promoter by 500 bases (module coords: 8895531-8898331)

*********************** Rank 1027 [Score 14.471191]   GBROWSE*******************

insitu CG8595 in-situ | Toll-7 | + | -29932 | -25592 | DOWNSTREAM | CG8595-RA | "-"
 CG30448 in-situ | Obp56i | + | 11279 | 11759 | UPSTREAM | CG30448-RA | "-"


*********************** Rank 1028 [Score 14.469360]   GBROWSE*******************

 CG31397 in-situ | CG31397 | + | -23921 | -23253 | DOWNSTREAM | CG31397-RA | "-"
 CG31394 in-situ | CG31394 | - | 1496 | 758 | DOWNSTREAM | CG31394-RA | "-"


*********************** Rank 1029 [Score 14.467529]   GBROWSE*******************

 CG17024 in-situ | CG17024 | + | -17147 | -15878 | DOWNSTREAM | CG17024-RA | "-"
 CG31858 in-situ | CG31858 | - | 26466 | 25816 | DOWNSTREAM | CG31858-RA | "-"

*********************** Rank 1030 [Score 14.467041]   GBROWSE*******************

 CG13117 in-situ | CG13117 | + | -8276 | -7588 | DOWNSTREAM | CG13117-RA | "-"
 CG18660 in-situ | Nckx30C | - | 1562 | -27398 | INTRAGENIC | intron:CG18660-RB:1 | intron:CG18660-RC:1 | intron:CG18660-RA:1 | CG18660-RB | "-" | CG18660-RC | "-" | CG18660-RA | "-"

*********************** Rank 1031 [Score 14.464478]   GBROWSE*******************

 CG32080 in-situ | CG32080 | + | -2916 | -2155 | DOWNSTREAM | CG32080-RA | "-"
 CG12296 in-situ | klu | - | 30306 | 3208 | DOWNSTREAM | CG12296-RA | "-"

*********************** Rank 1032 [Score 14.457031]   GBROWSE*******************

insitu CG31243 in-situ | cpo | + | -55740 | 28181 | INTRAGENIC | intron:CG31243-RA:3 | intron:CG31243-RE:3 | intron:CG31243-RF:3 | intron:CG31243-RB:3 | CG31243-RA | "-" | CG31243-RE | "-" | CG31243-RF | "-" | CG31243-RB | "-"
 CG7780 in-situ | DNaseII | + | 31764 | 33548 | UPSTREAM | CG7780-RA | "-"

*********************** Rank 1033 [Score 14.450012]   GBROWSE*******************

 CG10887 in-situ | CG10887 | + | -4622 | -2478 | DOWNSTREAM | CG10887-RA | "-"
 CG31208 in-situ | Gr92a | + | 1499 | 2539 | UPSTREAM | CG31208-RA | "-"

*********************** Rank 1034 [Score 14.449036]   GBROWSE*******************

 CG13789 in-situ | CG13789 | - | -3281 | -3918 | UPSTREAM | CG13789-RA | "-"
 CG13790 in-situ | CG13790 | - | 28949 | 28665 | DOWNSTREAM | CG13790-RA | "-"


*********************** Rank 1035 [Score 14.447266]   GBROWSE*******************

 CG1636 in-situ | CG1636 | + | -23304 | -21337 | DOWNSTREAM | CG1636-RA | "-"
 CG15344 in-situ | CG15344 | + | 27964 | 28371 | UPSTREAM | CG15344-RA | "-"

*********************** Rank 1036 [Score 14.445923]   GBROWSE*******************

 CG14176 in-situ | Or67b | - | -4428 | -6209 | UPSTREAM | CG14176-RB | "-"
 CG8329 in-situ | CG8329 | + | 4491 | 5405 | UPSTREAM | CG8329-RA | "-"


*********************** Rank 1037 [Score 14.443115]   GBROWSE*******************

 CG12681 in-situ | CG12681 | + | -14705 | -13242 | DOWNSTREAM | CG12681-RA | "-"
 CG15470 in-situ | CG15470 | + | 4303 | 5463 | UPSTREAM | CG15470-RA | "-"

*********************** Rank 1038 [Score 14.441650]   GBROWSE*******************

 CG31807 in-situ | CG31807 | - | -7050 | -7517 | UPSTREAM | CG31807-RA | "-"
 CG13263 in-situ | Cyt-c-d | + | 26442 | 30226 | UPSTREAM | CG13263-RA | "-"


*********************** Rank 1039 [Score 14.440613]   GBROWSE*******************

 CG12478 in-situ | bru-3 | - | -71683 | -200514 | UPSTREAM | CG12478-RA | "-" | CG12478-RB | "-"
 CG8757 in-situ | CG8757 | - | 95120 | 94154 | DOWNSTREAM | CG8757-RA | "-"

*********************** Rank 1040 [Score 14.440308]   GBROWSE*******************

 CG10117 in-situ | ttv | + | -11403 | 47347 | INTRAGENIC | intron:CG10117-RA:1 | CG10117-RA | "-"
 CG30076 in-situ | CG30076 | - | 7785 | 6463 | DOWNSTREAM | CG30076-RA | "-"

*********************** Rank 1041 [Score 14.436096]   GBROWSE*******************

 CG31921 in-situ | CG31921 | - | -16015 | -18359 | UPSTREAM | CG31921-RA | "-"
 CG11907 in-situ | CG11907 | - | 9517 | 7208 | DOWNSTREAM | CG11907-RA | "-" | CG11907-RB | "-"

*********************** Rank 1042 [Score 14.432739]   GBROWSE*******************

 CG3200 in-situ | Reg-2 | - | -10813 | -12206 | UPSTREAM | CG3200-RA | "-"
 CG32345 in-situ | CG32345 | - | 4945 | -1748 | INTRAGENIC | intron:CG32345-RA:1 | CG32345-RA | "-"

*********************** Rank 1043 [Score 14.425537]   GBROWSE*******************

 CG31394 in-situ | CG31394 | - | -74654 | -75392 | UPSTREAM | CG31394-RA | "-"
insitu highlight CG17117 in-situ | hth | - | 34854 | -93970 | INTRAGENIC | intron:CG17117-RD:3 | intron:CG17117-RB:2 | intron:CG17117-RC:2 | intron:CG17117-RA:1 | CG17117-RD | "-" | CG17117-RB | "-" | CG17117-RC | "-" | CG17117-RA | "-"

*********************** Rank 1044 [Score 14.421875]   GBROWSE*******************

 CG12283 in-situ | kek1 | - | -22443 | -26300 | UPSTREAM | CG12283-RA | "-"
 CG5983 in-situ | ACXC | + | 67300 | 71544 | UPSTREAM | CG5983-RA | "-"

*********************** Rank 1045 [Score 14.418579]   GBROWSE*******************

 CG10113 in-situ | CG10113 | + | -14844 | -12597 | DOWNSTREAM | CG10113-RA | "-"
insitu CG14598 in-situ | CG14598 | + | 4157 | 6383 | UPSTREAM | CG14598-RA | "-"

*********************** Rank 1046 [Score 14.414673]   GBROWSE*******************

 CG13873 in-situ | Obp56g | - | -19717 | -20180 | UPSTREAM | CG13873-RA | "-"
 CG13874 in-situ | Obp56h | + | 11840 | 12302 | UPSTREAM | CG13874-RA | "-"

*********************** Rank 1047 [Score 14.414307]   GBROWSE*******************

 CG10839 in-situ | BG:DS07486.2 | + | -16456 | -15896 | DOWNSTREAM | CG10839-RA | "-"
 CG4838 in-situ | beat-Ic | + | 4521 | 45031 | UPSTREAM | CG4838-RA | "-"


*********************** Rank 1048 [Score 14.410400]   GBROWSE*******************

 CG30384 in-situ | CG30384 | + | -6851 | -4545 | DOWNSTREAM | CG30384-RA | "-"
 CG1854 in-situ | Or43a | - | 45065 | 42991 | DOWNSTREAM | CG1854-RA | "-"


*********************** Rank 1049 [Score 14.409119]   GBROWSE*******************

 CG6914 in-situ | CG6914 | + | -11253 | -10492 | DOWNSTREAM | CG6914-RA | "-"
 CG14458 in-situ | CG14458 | + | 28954 | 32366 | UPSTREAM | CG14458-RA | "-"

*********************** Rank 1050 [Score 14.408203]   GBROWSE*******************

 CG7100 in-situ | CadN | - | -70896 | -160551 | UPSTREAM | CG7100-RA | "-" | CG7100-RC | "-" | CG7100-RD | "-" | CG7100-RE | "-" | CG7100-RF | "-" | CG7100-RG | "-" | CG7100-RH | "-" | CG7100-RB | "-"
 CG7527 in-situ | CadN2 | - | 2187 | -23812 | INTRAGENIC | intron:CG7527-RA:2 | CG7527-RA | "-"

*********************** Rank 1051 [Score 14.406128]   GBROWSE*******************

 CG11769 in-situ | CG11769 | + | -29910 | -29164 | DOWNSTREAM | CG11769-RA | "-"
 CG31448 in-situ | CG31448 | + | 258 | 950 | UPSTREAM | CG31448-RA | "-"

*********************** Rank 1052 [Score 14.404785]   GBROWSE*******************

 CG15021 in-situ | CG15021 | + | -8186 | -6587 | DOWNSTREAM | CG15021-RA | "-"
 CG12607 in-situ | CG12607 | + | 1669 | 3158 | UPSTREAM | CG12607-RB | "-"


*********************** Rank 1053 [Score 14.401123]   GBROWSE*******************

insitu CG3979 in-situ | Indy | - | -3811 | -21439 | UPSTREAM | CG3979-RB | "-" | CG3979-RC | "-" | CG3979-RA | "-"
 CG6865 in-situ | CG6865 | + | 14178 | 15350 | UPSTREAM | CG6865-RA | "-"

*********************** Rank 1054 [Score 14.400879]   GBROWSE*******************

 CG31395 in-situ | CG31395 | + | -15594 | -14899 | DOWNSTREAM | CG31395-RA | "-"
 CG31397 in-situ | CG31397 | + | 15029 | 15697 | UPSTREAM | CG31397-RA | "-"

*********************** Rank 1055 [Score 14.396729]   GBROWSE*******************

 CG32398 in-situ | CG32398 | - | -42860 | -43690 | UPSTREAM | CG32398-RA | "-"
 CG14910 in-situ | CG14910 | + | 49145 | 49648 | UPSTREAM | CG14910-RA | "-"

*********************** Rank 1056 [Score 14.396729]   GBROWSE*******************

 CG33111 in-situ | CG33111 | - | -8939 | -26414 | UPSTREAM | CG33111-RA | "-"
 CG12492 in-situ | CG12492 | + | 17235 | 21178 | UPSTREAM | CG12492-RA | "-"

*********************** Rank 1057 [Score 14.377075]   GBROWSE*******************

 CG31395 in-situ | CG31395 | + | -10344 | -9649 | DOWNSTREAM | CG31395-RA | "-"
 CG31397 in-situ | CG31397 | + | 20279 | 20947 | UPSTREAM | CG31397-RA | "-"

*********************** Rank 1058 [Score 14.374023]   GBROWSE*******************

 CG32368 in-situ | CG32368 | + | -6085 | -5667 | DOWNSTREAM | CG32368-RA | "-"
 CG8110 in-situ | syd | - | 14442 | 5071 | DOWNSTREAM | CG8110-RA | "-" | CG8110-RB | "-"

*********************** Rank 1059 [Score 14.371216]   GBROWSE*******************

 CG5020 in-situ | CLIP-190 | + | -3227 | 21732 | INTRAGENIC | intron:CG5020-RB:3 | intron:CG5020-RA:2 | intron:CG5020-RD:1 | CG5020-RB | "-" | CG5020-RA | "-" | CG5020-RD | "-" | CG5020-RC | "-"
 CG6840 in-situ | Rpb11 | - | 22401 | 21729 | DOWNSTREAM | CG6840-RA | "-"

*********************** Rank 1060 [Score 14.371094]   GBROWSE*******************

 CG8204 in-situ | CG8204 | - | -8484 | -9339 | UPSTREAM | CG8204-RA | "-"
 CG30465 in-situ | CG30465 | + | 23792 | 24413 | UPSTREAM | CG30465-RA | "-"

*********************** Rank 1061 [Score 14.368408]   GBROWSE*******************

 CG8585 in-situ | Ih | + | -12039 | 1334 | INTRAGENIC | intron:CG8585-RA:9 | CG8585-RA | "-"
 CG8589 in-situ | CG8589 | - | 4779 | 2526 | DOWNSTREAM | CG8589-RA | "-"

*********************** Rank 1062 [Score 14.366577]   GBROWSE*******************

 CG17330 in-situ | BG:DS09218.5 | + | -27447 | -26389 | DOWNSTREAM | CG17330-RA | "-"
 CG4472 in-situ | Idgf1 | + | 51914 | 53481 | UPSTREAM | CG4472-RA | "-"

*********************** Rank 1063 [Score 14.365967]   GBROWSE*******************

 CG14459 in-situ | CG14459 | - | -14969 | -15708 | UPSTREAM | CG14459-RA | "-"
 CG6914 in-situ | CG6914 | + | 7747 | 8508 | UPSTREAM | CG6914-RA | "-"

*********************** Rank 1064 [Score 14.359497]   GBROWSE*******************

 CG8705 in-situ | pnut | + | -11184 | -8123 | DOWNSTREAM | CG8705-RB | "-" | CG8705-RA | "-"
 CG14760 in-situ | CG14760 | + | 5803 | 8283 | UPSTREAM | CG14760-RA | "-"


*********************** Rank 1065 [Score 14.355957]   GBROWSE*******************

 CG7763 in-situ | BEST:CK02422 | + | -6438 | -5728 | DOWNSTREAM | CG7763-RA | "-"
 CG9023 in-situ | Drip | - | 8454 | -8804 | INTRAGENIC | intron:CG9023-RA:2 | intron:CG9023-RB:3 | CG9023-RA | "-" | CG9023-RB | "-"

*********************** Rank 1066 [Score 14.352051]   GBROWSE*******************

 CG13984 in-situ | CG13984 | + | -5468 | -5166 | DOWNSTREAM | CG13984-RA | "-"
 CG9490 in-situ | CG9490 | + | 5893 | 7800 | UPSTREAM | CG9490-RA | "-"

*********************** Rank 1067 [Score 14.344482]   GBROWSE*******************

 CG31315 in-situ | CG31315 | + | -5069 | -4596 | DOWNSTREAM | CG31315-RA | "-"
 CG12784 in-situ | CG12784 | + | 3155 | 3604 | UPSTREAM | CG12784-RA | "-"

*********************** Rank 1068 [Score 14.339294]   GBROWSE*******************

insitu highlight CG15162 in-situ | MESR3 | + | -10310 | 34220 | INTRAGENIC | intron:CG15162-RA:1 | CG15162-RA | "-"
insitu CG10391 in-situ | Cyp310a1 | - | 25417 | 23490 | DOWNSTREAM | CG10391-RA | "-"

*********************** Rank 1069 [Score 14.339111]   GBROWSE*******************

 CG30413 in-situ | CG30413 | - | -12907 | -13275 | UPSTREAM | CG30413-RA | "-"
 CG9863 in-situ | CG9863 | + | 3703 | 4845 | UPSTREAM | CG9863-RA | "-"

*********************** Rank 1070 [Score 14.338135]   GBROWSE*******************

 CG14325 in-situ | CG14325 | + | -29886 | -28001 | DOWNSTREAM | CG14325-RA | "-"
 CG14322 in-situ | CG14322 | + | 3611 | 12204 | UPSTREAM | CG14322-RA | "-"

*********************** Rank 1071 [Score 14.337402]   GBROWSE*******************

insitu highlight CG1133 in-situ | opa | + | -6334 | 10808 | INTRAGENIC | intron:CG1133-RA:1 | CG1133-RA | "-"
 CG14660 in-situ | CG14660 | - | 19354 | 16826 | DOWNSTREAM | CG14660-RA | "-"

*********************** Rank 1072 [Score 14.336182]   GBROWSE*******************

 CG13783 in-situ | CG13783 | - | -1822 | -3062 | UPSTREAM | CG13783-RA | "-"
 CG4495 in-situ | CG4495 | + | 23717 | 27011 | UPSTREAM | CG4495-RA | "-"


*********************** Rank 1073 [Score 14.336182]   GBROWSE*******************

 CG6902 in-situ | CG6902 | - | -1129 | -4326 | UPSTREAM | CG6902-RA | "-"
 CG6694 in-situ | CG6694 | - | 14307 | 12029 | DOWNSTREAM | CG6694-RA | "-"

*********************** Rank 1074 [Score 14.334717]   GBROWSE*******************

 CG18516 in-situ | CG18516 | - | -17038 | -21182 | UPSTREAM | CG18516-RA | "-"
 CG5302 in-situ | CG5302 | + | 63073 | 69445 | UPSTREAM | CG5302-RA | "-"

*********************** Rank 1075 [Score 14.331909]   GBROWSE*******************

insitu highlight CG10325 in-situ | abd-A | - | -29762 | -52188 | UPSTREAM | CG10325-RA | "-" | CG10325-RB | "-"
 CG10349 in-situ | CG10349 | + | 19915 | 25297 | UPSTREAM | CG10349-RA | "-" | CG10349-RB | "-"

*********************** Rank 1076 [Score 14.322998]   GBROWSE*******************

insitu CG31666 in-situ | CG31666 | + | -17273 | 28094 | INTRAGENIC | intron:CG31666-RD:1 | intron:CG31666-RA:2 | intron:CG31666-RB:2 | intron:CG31666-RC:2 | CG31666-RD | "-" | CG31666-RA | "-" | CG31666-RB | "-" | CG31666-RC | "-"
 CG31934 in-situ | CG31934 | - | 781 | 97 | DOWNSTREAM | CG31934-RA | "-"

*********************** Rank 1077 [Score 14.322205]   GBROWSE*******************

insitu highlight CG10619 in-situ | tup | - | -13611 | -35367 | UPSTREAM | CG10619-RA | "-" | CG10619-RB | "-"
 CG18397 in-situ | CG18397 | - | 49081 | 21658 | DOWNSTREAM | CG18397-RA | "-"

*********************** Rank 1078 [Score 14.316040]   GBROWSE*******************

 CG32406 in-situ | CG32406 | + | -1778 | 27218 | INTRAGENIC | intron:CG32406-RA:1 | CG32406-RA | "-"
 CG10478 in-situ | CG10478 | - | 11959 | 10564 | DOWNSTREAM | CG10478-RA | "-"

*********************** Rank 1079 [Score 14.314514]   GBROWSE*******************

 CG1303 in-situ | agt | - | -6627 | -7321 | UPSTREAM | CG1303-RA | "-"
insitu highlight CG1264 in-situ | lab | - | 11863 | -5309 | INTRAGENIC | intron:CG1264-RA:1 | CG1264-RA | "-"

*********************** Rank 1080 [Score 14.312988]   GBROWSE*******************

insitu highlight CG10002 in-situ | fkh | - | -1229 | -4496 | UPSTREAM | CG10002-RA | "-"
insitu CG10009 in-situ | Noa36 | - | 12967 | 11746 | DOWNSTREAM | CG10009-RA | "-"

*********************** Rank 1081 [Score 14.304443]   GBROWSE*******************

 CG13325 in-situ | CG13325 | + | -3831 | 5644 | INTRAGENIC | intron:CG13325-RA:1 | CG13325-RA | "-"
 CG3955 in-situ | CG3955 | - | 8918 | 7776 | DOWNSTREAM | CG3955-RA | "-"

*********************** Rank 1082 [Score 14.303589]   GBROWSE*******************

 CG13616 in-situ | CG13616 | + | -27298 | -26479 | DOWNSTREAM | CG13616-RA | "-"
 CG5610 in-situ | nAcRalpha-96Aa | - | 36708 | -18995 | INTRAGENIC | intron:CG5610-RA:3 | CG5610-RA | "-"


*********************** Rank 1083 [Score 14.302612]   GBROWSE*******************

 CG12582 in-situ | CG12582 | + | -11122 | -3856 | DOWNSTREAM | CG12582-RA | "-"
insitu CG1107 in-situ | auxillin | + | 3354 | 19093 | UPSTREAM | CG1107-RA | "-" | CG1107-RB | "-"


*********************** Rank 1084 [Score 14.302612]   GBROWSE*******************

 CG12726 in-situ | CG12726 | - | -8609 | -9278 | UPSTREAM | CG12726-RA | "-"
 CG15753 in-situ | CG15753 | + | 54512 | 57824 | UPSTREAM | CG15753-RA | "-"


*********************** Rank 1085 [Score 14.301147]   GBROWSE*******************

 CG5103 in-situ | CG5103 | - | -2004 | -4254 | UPSTREAM | CG5103-RA | "-"
 CG13700 in-situ | CG13700 | - | 4889 | 2516 | DOWNSTREAM | CG13700-RA | "-"

*********************** Rank 1086 [Score 14.300476]   GBROWSE*******************

insitu CG5799 in-situ | dve | + | -19636 | 22819 | INTRAGENIC | intron:CG5799-RA:2 | intron:CG5799-RD:2 | intron:CG5799-RC:2 | CG5799-RA | "-" | CG5799-RD | "-" | CG5799-RB | "-" | CG5799-RC | "-"
insitu CG5819 in-situ | CG5819 | + | 29860 | 33359 | UPSTREAM | CG5819-RA | "-" | CG5819-RB | "-"

*********************** Rank 1087 [Score 14.299591]   GBROWSE*******************

 CG1631 in-situ | CG1631 | + | -12558 | -11371 | DOWNSTREAM | CG1631-RA | "-"
 CG15462 in-situ | CG15462 | - | 27898 | 26723 | DOWNSTREAM | CG15462-RA | "-"

*********************** Rank 1088 [Score 14.298950]   GBROWSE*******************

insitu CG9614 in-situ | pip | - | -8472 | -47541 | UPSTREAM | CG9614-RA | "-" | CG9614-RC | "-" | CG9614-RD | "-" | CG9614-RE | "-" | CG9614-RF | "-" | CG9614-RG | "-" | CG9614-RH | "-" | CG9614-RI | "-" | CG9614-RJ | "-" | CG9614-RK | "-" | CG9614-RL | "-"
 CG14087 in-situ | CG14087 | + | 41531 | 43962 | UPSTREAM | CG14087-RA | "-"

*********************** Rank 1089 [Score 14.298889]   GBROWSE*******************

 CG31796 in-situ | CG31796 | - | -6868 | -7441 | UPSTREAM | CG31796-RA | "-"
 CG17564 in-situ | CG17564 | - | 19684 | 18168 | DOWNSTREAM | CG17564-RB | "-"

*********************** Rank 1090 [Score 14.298828]   GBROWSE*******************

 CG13652 in-situ | CG13652 | + | -4466 | -3492 | DOWNSTREAM | CG13652-RA | "-"
 CG13661 in-situ | CG13661 | - | 7290 | 4237 | DOWNSTREAM | CG13661-RA | "-"

*********************** Rank 1091 [Score 14.298828]   GBROWSE*******************

 CG12498 in-situ | EG:BACR43E12.1 | + | -11311 | -10526 | DOWNSTREAM | CG12498-RA | "-"
 CG14416 in-situ | EG:BACR43E12.7 | - | 51542 | 50711 | DOWNSTREAM | CG14416-RA | "-"

*********************** Rank 1092 [Score 14.296753]   GBROWSE*******************

insitu CG9717 in-situ | CG9717 | - | -9563 | -12978 | UPSTREAM | CG9717-RA | "-"
 CG31019 in-situ | CG31019 | + | 142 | 2434 | UPSTREAM | CG31019-RA | "-"


*********************** Rank 1093 [Score 14.296631]   GBROWSE*******************

 CG15552 in-situ | Sox100B | + | -23255 | -5205 | DOWNSTREAM | CG15552-RA | "-"
insitu CG11317 in-situ | CG11317 | - | 8706 | 2095 | DOWNSTREAM | CG11317-RA | "-"

*********************** Rank 1094 [Score 14.295044]   GBROWSE*******************

 CG6640 in-situ | CG6640 | - | -40490 | -45624 | UPSTREAM | CG6640-RA | "-" | CG6640-RB | "-"
 CG8072 in-situ | CG8072 | + | 10593 | 11385 | UPSTREAM | CG8072-RA | "-"

*********************** Rank 1095 [Score 14.288330]   GBROWSE*******************

 CG30165 in-situ | CG30165 | - | -210 | -3311 | UPSTREAM | CG30165-RA | "-"
 CG30164 in-situ | CG30164 | - | 4941 | 79 | DOWNSTREAM | CG30164-RA | "-"

*********************** Rank 1096 [Score 14.287476]   GBROWSE*******************

 CG12493 in-situ | CG12493 | - | -141 | -1466 | UPSTREAM | CG12493-RA | "-"
 CG13711 in-situ | CG13711 | + | 4170 | 4553 | UPSTREAM | CG13711-RA | "-"

*********************** Rank 1097 [Score 14.271851]   GBROWSE*******************

 CG31826 in-situ | CG31826 | - | -9597 | -11457 | UPSTREAM | CG31826-RA | "-"
 CG18109 in-situ | BG:DS02252.3 | + | 1539 | 7334 | UPSTREAM | CG18109-RA | "-"


*********************** Rank 1098 [Score 14.271606]   GBROWSE*******************

insitu CG18783 in-situ | Kr-h1 | + | -10677 | 4019 | INTRAGENIC | intron:CG18783-RA:1 | intron:CG18783-RB:1 | CG18783-RA | "-" | CG18783-RB | "-"
insitu CG9175 in-situ | CG9175 | - | 6479 | 4210 | DOWNSTREAM | CG9175-RA | "-" | CG9175-RB | "-"

*********************** Rank 1099 [Score 14.269775]   GBROWSE*******************

insitu CG10059 in-situ | MAGE | - | -2383 | -3321 | UPSTREAM | CG10059-RA | "-"
 CG2507 in-situ | sas | + | 5270 | 26143 | UPSTREAM | CG2507-RB | "-" | CG2507-RA | "-"

*********************** Rank 1100 [Score 14.263550]   GBROWSE*******************

 CG13997 in-situ | CG13997 | - | -694 | -1239 | UPSTREAM | CG13997-RA | "-"
 CG9048 in-situ | Vm26Aa | - | 1055 | 414 | DOWNSTREAM | CG9048-RA | "-"

*********************** Rank 1101 [Score 14.260742]   GBROWSE*******************

 CG10039 in-situ | CG10039 | + | -9228 | -7932 | DOWNSTREAM | CG10039-RA | "-"
 CG12676 in-situ | ed | + | 14448 | 98128 | UPSTREAM | CG12676-RA | "-"

*********************** Rank 1102 [Score 14.260132]   GBROWSE*******************

 CG11692 in-situ | CG11692 | - | -872 | -1428 | UPSTREAM | CG11692-RA | "-"
 CG1829 in-situ | Cyp6v1 | + | 18284 | 21753 | UPSTREAM | CG1829-RA | "-"

*********************** Rank 1103 [Score 14.258423]   GBROWSE*******************

 CG6633 in-situ | Ugt86Dd | - | -570 | -2310 | UPSTREAM | CG6633-RA | "-"
 CG4706 in-situ | CG4706 | + | 8167 | 10740 | UPSTREAM | CG4706-RA | "-"

*********************** Rank 1104 [Score 14.257324]   GBROWSE*******************

insitu CG11387 in-situ | ct | + | -67475 | -600 | DOWNSTREAM | CG11387-RA | "-" | CG11387-RB | "-"
 CG12690 in-situ | CHES-1-like | - | 20071 | 8264 | DOWNSTREAM | CG12690-RA | "-"

*********************** Rank 1105 [Score 14.256958]   GBROWSE*******************

 CG31262 in-situ | CG31262 | - | -16981 | -19074 | UPSTREAM | CG31262-RA | "-"
 CG4135 in-situ | beat-IIb | - | 11463 | -3653 | INTRAGENIC | intron:CG4135-RA:4 | CG4135-RA | "-"

*********************** Rank 1106 [Score 14.251709]   GBROWSE*******************

 CG14247 in-situ | CG14247 | + | -6171 | -5644 | DOWNSTREAM | CG14247-RA | "-"
 CG5490 in-situ | Tl | + | 55030 | 98390 | UPSTREAM | CG5490-RB | "-" | CG5490-RA | "-"


*********************** Rank 1107 [Score 14.247925]   GBROWSE*******************

 CG13442 in-situ | CG13442 | - | -1128 | -3914 | UPSTREAM | CG13442-RA | "-"
 CG13443 in-situ | CG13443 | + | 28204 | 29588 | UPSTREAM | CG13443-RA | "-"

*********************** Rank 1108 [Score 14.246094]   GBROWSE*******************

 CG15631 in-situ | CG15631 | - | -31023 | -33072 | UPSTREAM | CG15631-RA | "-"
 CG15630 in-situ | CG15630 | - | 28561 | -29934 | INTRAGENIC | intron:CG15630-RA:1 | CG15630-RA | "-"

*********************** Rank 1109 [Score 14.244141]   GBROWSE*******************

insitu CG5799 in-situ | dve | + | -31636 | 10819 | INTRAGENIC | intron:CG5799-RA:2 | intron:CG5799-RD:2 | intron:CG5799-RC:3 | CG5799-RA | "-" | CG5799-RD | "-" | CG5799-RB | "-" | CG5799-RC | "-"
insitu CG5819 in-situ | CG5819 | + | 17860 | 21359 | UPSTREAM | CG5819-RA | "-" | CG5819-RB | "-"

*********************** Rank 1110 [Score 14.223633]   GBROWSE*******************

 CG9656 in-situ | grn | - | -20971 | -55063 | UPSTREAM | CG9656-RA | "-"
 CG7800 in-situ | CG7800 | + | 15565 | 17333 | UPSTREAM | CG7800-RA | "-"

*********************** Rank 1111 [Score 14.218933]   GBROWSE*******************

 CG6604 in-situ | H15 | + | -37643 | -26057 | DOWNSTREAM | CG6604-RA | "-"
 CG31647 in-situ | CG31647 | - | 7217 | -6406 | INTRAGENIC | intron:CG31647-RA:3 | CG31647-RA | "-" | CG31647-RB | "-"

*********************** Rank 1112 [Score 14.218872]   GBROWSE*******************

insitu highlight CG6464 in-situ | salm | - | -33776 | -45068 | UPSTREAM | CG6464-RA | "-"
insitu highlight CG4922 in-situ | sala | + | 6810 | 7576 | UPSTREAM | CG4922-RA | "-"

*********************** Rank 1113 [Score 14.218750]   GBROWSE*******************

 CG5526 in-situ | Dhc36C | + | -18650 | 5400 | INTRAGENIC | intron:CG5526-RA:21 | CG5526-RA | "-"
 CG15143 in-situ | CG15143 | - | 4432 | 772 | DOWNSTREAM | CG15143-RA | "-"

*********************** Rank 1114 [Score 14.218384]   GBROWSE*******************

 CG12444 in-situ | CG12444 | - | -18194 | -19879 | UPSTREAM | CG12444-RB | "-" | CG12444-RA | "-"
 CG12443 in-situ | CG12443 | + | 2121 | 4181 | UPSTREAM | CG12443-RA | "-"


*********************** Rank 1115 [Score 14.216003]   GBROWSE*******************

insitu highlight CG5461 in-situ | bun | - | -15209 | -99202 | UPSTREAM | CG5461-RA | "-" | CG5461-RB | "-" | CG5461-RC | "-"
 CG15489 in-situ | CG15489 | + | 38008 | 38910 | UPSTREAM | CG15489-RA | "-"

*********************** Rank 1116 [Score 14.211060]   GBROWSE*******************

 CG10037 in-situ | vvl | + | -72461 | -68085 | DOWNSTREAM | CG10037-RA | "-"
insitu CG10078 in-situ | Prat2 | - | 52237 | 49372 | DOWNSTREAM | CG10078-RB | "-" | CG10078-RA | "-"

*********************** Rank 1117 [Score 14.208984]   GBROWSE*******************

 CG2146 in-situ | didum | + | -744 | 7726 | INTRAGENIC | intron:CG2146-RA:1 | intron:CG2146-RB:1 | intron:CG2146-RC:1 | CG2146-RA | "-" | CG2146-RB | "-" | CG2146-RC | "-"
 CG12736 in-situ | CG12736 | - | 9555 | 7628 | DOWNSTREAM | CG12736-RA | "-"


*********************** Rank 1118 [Score 14.208374]   GBROWSE*******************

insitu CG10710 in-situ | CG10710 | - | -100881 | -104209 | UPSTREAM | CG10710-RA | "-"
 CG12478 in-situ | bru-3 | - | 46267 | -82564 | INTRAGENIC | intron:CG12478-RA:4 | intron:CG12478-RB:3 | CG12478-RA | "-" | CG12478-RB | "-"


*********************** Rank 1119 [Score 14.206909]   GBROWSE*******************

 CG4891 in-situ | BG:DS04095.1 | + | -19857 | -18922 | DOWNSTREAM | CG4891-RA | "-"
 CG4892 in-situ | BG:DS04095.2 | + | 18642 | 19546 | UPSTREAM | CG4892-RA | "-"

*********************** Rank 1120 [Score 14.206543]   GBROWSE*******************

 CG13387 in-situ | emb | + | -2244 | 3034 | INTRAGENIC | intron:CG13387-RA:2 | CG13387-RA | "-"
 CG13397 in-situ | ESTS:172F5T | - | 6049 | 3227 | DOWNSTREAM | CG13397-RA | "-"

*********************** Rank 1121 [Score 14.206055]   GBROWSE*******************

 CG7449 in-situ | hbs | + | -3276 | 26721 | INTRAGENIC | intron:CG7449-RA:1 | intron:CG7449-RB:1 | CG7449-RA | "-" | CG7449-RB | "-"
 CG30473 in-situ | Obp51a | - | 10165 | 9745 | DOWNSTREAM | CG30473-RA | "-"

*********************** Rank 1122 [Score 14.205444]   GBROWSE*******************

insitu CG11387 in-situ | ct | + | -31375 | 35500 | INTRAGENIC | intron:CG11387-RA:1 | intron:CG11387-RB:2 | CG11387-RA | "-" | CG11387-RB | "-"
 CG12690 in-situ | CHES-1-like | - | 56171 | 44364 | DOWNSTREAM | CG12690-RA | "-"

*********************** Rank 1123 [Score 14.203674]   GBROWSE*******************

 CG18363 in-situ | CG18363 | - | -13000 | -14108 | UPSTREAM | CG18363-RA | "-"
 CG12477 in-situ | CG12477 | - | 11856 | 11059 | DOWNSTREAM | CG12477-RA | "-"

*********************** Rank 1124 [Score 14.200806]   GBROWSE*******************

insitu CG1416 in-situ | CG1416 | + | -13777 | -10864 | DOWNSTREAM | CG1416-RA | "-" | CG1416-RB | "-" | CG1416-RC | "-"
 CG11630 in-situ | CG11630 | - | 12234 | 10339 | DOWNSTREAM | CG11630-RA | "-"

*********************** Rank 1125 [Score 14.194824]   GBROWSE*******************

 CG31283 in-situ | CG31283 | - | -17273 | -18487 | UPSTREAM | CG31283-RA | "-"
 CG6898 in-situ | Zip3 | + | 5853 | 8792 | UPSTREAM | CG6898-RA | "-"

*********************** Rank 1126 [Score 14.189819]   GBROWSE*******************

 CG31262 in-situ | CG31262 | - | -16081 | -18174 | UPSTREAM | CG31262-RA | "-"
 CG4135 in-situ | beat-IIb | - | 12363 | -2753 | INTRAGENIC | intron:CG4135-RA:4 | CG4135-RA | "-"

*********************** Rank 1127 [Score 14.187744]   GBROWSE*******************

 CG12063 in-situ | CG12063 | + | -39189 | -31064 | DOWNSTREAM | CG12063-RA | "-"
insitu CG1499 in-situ | CG1499 | + | 4970 | 28105 | UPSTREAM | CG1499-RA | "-" | CG1499-RB | "-"

*********************** Rank 1128 [Score 14.187561]   GBROWSE*******************

 CG6287 in-situ | CG6287 | - | -2875 | -7085 | UPSTREAM | CG6287-RA | "-"
 CG6320 in-situ | Ca-beta | - | 11880 | 1654 | DOWNSTREAM | CG6320-RA | "-" | CG6320-RB | "-"

*********************** Rank 1129 [Score 14.185364]   GBROWSE*******************

insitu CG4476 in-situ | CG4476 | + | -13106 | -9721 | DOWNSTREAM | CG4476-RB | "-"
 CG4483 in-situ | CG4483 | - | 11658 | 10160 | DOWNSTREAM | CG4483-RA | "-"

*********************** Rank 1130 [Score 14.174072]   GBROWSE*******************

 CG14162 in-situ | CG14162 | + | -109452 | -42763 | DOWNSTREAM | CG14162-RA | "-"
 CG14160 in-situ | CG14160 | - | 5058 | 3071 | DOWNSTREAM | CG14160-RA | "-"

*********************** Rank 1131 [Score 14.173035]   GBROWSE*******************

 CG9266 in-situ | CG9266 | - | -965 | -4389 | UPSTREAM | CG9266-RB | "-"
insitu CG1762 in-situ | betaInt-nu | + | 82926 | 88138 | UPSTREAM | CG1762-RA | "-"


*********************** Rank 1132 [Score 14.171631]   GBROWSE*******************

 CG1031 in-situ | alpha-Est1 | - | -15987 | -19382 | UPSTREAM | CG1031-RA | "-"
 CG32465 in-situ | CG32465 | - | 16156 | 6181 | DOWNSTREAM | CG32465-RB | "-"

*********************** Rank 1133 [Score 14.171265]   GBROWSE*******************

 CG5732 in-situ | CG5732 | + | -67390 | -62518 | DOWNSTREAM | CG5732-RA | "-"
 CG7084 in-situ | CG7084 | + | 19602 | 24617 | UPSTREAM | CG7084-RB | "-" | CG7084-RA | "-"

*********************** Rank 1134 [Score 14.171265]   GBROWSE*******************

 CG13978 in-situ | CG13978 | + | -5474 | -3481 | DOWNSTREAM | CG13978-RA | "-"
insitu CG13977 in-situ | Cyp6a18 | + | 9014 | 10805 | UPSTREAM | CG13977-RA | "-"

*********************** Rank 1135 [Score 14.170898]   GBROWSE*******************

 CG9887 in-situ | CG9887 | + | -2677 | 4715 | INTRAGENIC | intron:CG9887-RA:3 | CG9887-RA | "-"
 CG18641 in-situ | CG18641 | - | 9146 | 7901 | DOWNSTREAM | CG18641-RA | "-"

*********************** Rank 1136 [Score 14.170532]   GBROWSE*******************

insitu CG5799 in-situ | dve | + | -25536 | 16919 | INTRAGENIC | intron:CG5799-RA:2 | intron:CG5799-RD:2 | intron:CG5799-RC:2 | CG5799-RA | "-" | CG5799-RD | "-" | CG5799-RB | "-" | CG5799-RC | "-"
insitu CG5819 in-situ | CG5819 | + | 23960 | 27459 | UPSTREAM | CG5819-RA | "-" | CG5819-RB | "-"

*********************** Rank 1137 [Score 14.167847]   GBROWSE*******************

 CG11550 in-situ | CG11550 | - | -10482 | -12135 | UPSTREAM | CG11550-RA | "-"
insitu CG1856 in-situ | ttk | + | 23522 | 45030 | UPSTREAM | CG1856-RE | "-" | CG1856-RF | "-" | CG1856-RB | "-" | CG1856-RC | "-" | CG1856-RA | "-" | CG1856-RD | "-"

*********************** Rank 1138 [Score 14.167725]   GBROWSE*******************

 CG14363 in-situ | CG14363 | - | -18580 | -21557 | UPSTREAM | CG14363-RA | "-"
 CG17956 in-situ | Mst87F | - | 18151 | 17586 | DOWNSTREAM | CG17956-RA | "-"

*********************** Rank 1139 [Score 14.167358]   GBROWSE*******************

insitu CG32139 in-situ | Sox21b | - | -37896 | -56837 | UPSTREAM | CG32139-RA | "-"
insitu highlight CG5893 in-situ | D | - | 9400 | 6381 | DOWNSTREAM | CG5893-RA | "-"

*********************** Rank 1140 [Score 14.164429]   GBROWSE*******************

 CG32193 in-situ | CG32193 | + | -12061 | -8041 | DOWNSTREAM | CG32193-RA | "-"
 CG32192 in-situ | CG32192 | + | 66596 | 67331 | UPSTREAM | CG32192-RA | "-" | CG32192-RB | "-"

*********************** Rank 1141 [Score 14.158752]   GBROWSE*******************

 CG5890 in-situ | CG5890 | + | -2659 | -297 | DOWNSTREAM | CG5890-RA | "-"
 CG6120 in-situ | Tsp96F | - | 11477 | 3571 | DOWNSTREAM | CG6120-RA | "-"

*********************** Rank 1142 [Score 14.154663]   GBROWSE*******************

 CG4374 in-situ | CG4374 | - | -50964 | -55316 | UPSTREAM | CG4374-RA | "-"
 CG31225 in-situ | CG31225 | + | 18396 | 19970 | UPSTREAM | CG31225-RA | "-"

*********************** Rank 1143 [Score 14.148926]   GBROWSE*******************

 CG7574 in-situ | bip1 | - | -6300 | -10660 | UPSTREAM | CG7574-RA | "-"
 CG13681 in-situ | CG13681 | + | 6810 | 7322 | UPSTREAM | CG13681-RA | "-"

*********************** Rank 1144 [Score 14.147095]   GBROWSE*******************

 CG16991 in-situ | Tsp66A | + | -2601 | -269 | DOWNSTREAM | CG16991-RB | "-" | CG16991-RA | "-"
 CG8543 in-situ | CG8543 | - | 1164 | 505 | DOWNSTREAM | CG8543-RA | "-"


*********************** Rank 1145 [Score 14.146240]   GBROWSE*******************

 CG4162 in-situ | lace | + | -31356 | -25868 | DOWNSTREAM | CG4162-RA | "-"
 CG15256 in-situ | BG:DS04862.2 | + | 20006 | 32784 | UPSTREAM | CG15256-RA | "-"

*********************** Rank 1146 [Score 14.145874]   GBROWSE*******************

 CG12990 in-situ | CG12990 | - | -17053 | -19733 | UPSTREAM | CG12990-RA | "-"
insitu CG32556 in-situ | CG32556 | - | 9585 | -16487 | INTRAGENIC | intron:CG32556-RA:3 | CG32556-RA | "-"


*********************** Rank 1147 [Score 14.145264]   GBROWSE*******************

 CG15275 in-situ | BG:DS01219.3 | - | -46620 | -47343 | UPSTREAM | CG15275-RA | "-"
 CG4482 in-situ | BG:DS01219.1 | - | 10825 | -7463 | INTRAGENIC | intron:CG4482-RA:4 | intron:CG4482-RB:3 | CG4482-RA | "-" | CG4482-RB | "-"

*********************** Rank 1148 [Score 14.140503]   GBROWSE*******************

 CG4849 in-situ | CG4849 | + | -13646 | -10096 | DOWNSTREAM | CG4849-RA | "-"
 CG4869 in-situ | CG4869 | - | 11305 | -10054 | INTRAGENIC | intron:CG4869-RA:1 | CG4869-RA | "-"

*********************** Rank 1149 [Score 14.139160]   GBROWSE*******************

 CG13896 in-situ | CG13896 | - | -14497 | -15129 | UPSTREAM | CG13896-RA | "-"
 CG13897 in-situ | CG13897 | - | 13049 | 11606 | DOWNSTREAM | CG13897-RA | "-"

*********************** Rank 1150 [Score 14.138428]   GBROWSE*******************

 CG31769 in-situ | CG31769 | - | -15709 | -16857 | UPSTREAM | CG31769-RA | "-"
 CG15292 in-situ | CG15292 | - | 717 | 547 | DOWNSTREAM | CG15292-RA | "-"

*********************** Rank 1151 [Score 14.135376]   GBROWSE*******************

 CG5267 in-situ | CG5267 | + | -6162 | -5626 | DOWNSTREAM | CG5267-RA | "-"
 CG8566 in-situ | unc-104 | - | 4657 | -9829 | INTRAGENIC | intron:CG8566-RD:9 | CG8566-RD | "-"

*********************** Rank 1152 [Score 14.133728]   GBROWSE*******************

 CG8541 in-situ | CG8541 | + | -12211 | -11384 | DOWNSTREAM | CG8541-RA | "-"
 CG16992 in-situ | CG16992 | + | 2249 | 4022 | UPSTREAM | CG16992-RA | "-"

*********************** Rank 1153 [Score 14.132446]   GBROWSE*******************

insitu CG8770 in-situ | Gbeta76C | + | -2715 | -351 | DOWNSTREAM | CG8770-RA | "-"
 CG8756 in-situ | CG8756 | - | 4888 | 163 | DOWNSTREAM | CG8756-RB | "-" | CG8756-RA | "-" | CG8756-RC | "-" | CG8756-RD | "-"

*********************** Rank 1154 [Score 14.130920]   GBROWSE*******************

 CG17839 in-situ | CG17839 | + | -26868 | 49613 | INTRAGENIC | intron:CG17839-RA:2 | CG17839-RA | "-"
 CG13467 in-situ | CG13467 | + | 71993 | 73016 | UPSTREAM | CG13467-RA | "-"

*********************** Rank 1155 [Score 14.129272]   GBROWSE*******************

 CG31630 in-situ | CG31630 | + | -2152 | 2648 | INTRAGENIC | intron:CG31630-RA:1 | CG31630-RA | "-"
 CG13778 in-situ | Mnn1 | + | 11687 | 17102 | UPSTREAM | CG13778-RA | "-" | CG13778-RB | "-"

*********************** Rank 1156 [Score 14.124268]   GBROWSE*******************

 CG31093 in-situ | CG31093 | - | -3492 | -4144 | UPSTREAM | CG31093-RA | "-"
 CG5024 in-situ | CG5024 | + | 17133 | 17793 | UPSTREAM | CG5024-RA | "-"


*********************** Rank 1157 [Score 14.123901]   GBROWSE*******************

 CG30474 in-situ | CG30474 | - | -1472 | -2073 | UPSTREAM | CG30474-RA | "-"
 CG7449 in-situ | hbs | + | 14724 | 44721 | UPSTREAM | CG7449-RA | "-" | CG7449-RB | "-"

*********************** Rank 1158 [Score 14.123230]   GBROWSE*******************

 CG14460 in-situ | CG14460 | + | -28603 | -28316 | DOWNSTREAM | CG14460-RA | "-"
 CG11449 in-situ | CG11449 | + | 1851 | 3467 | UPSTREAM | CG11449-RA | "-"

*********************** Rank 1159 [Score 14.120728]   GBROWSE*******************

 CG10417 in-situ | CG10417 | - | -25127 | -27904 | UPSTREAM | CG10417-RA | "-" | CG10417-RB | "-"
 CG30437 in-situ | CG30437 | + | 25507 | 65922 | UPSTREAM | CG30437-RA | "-" | CG30437-RC | "-" | CG30437-RB | "-"

*********************** Rank 1160 [Score 14.120300]   GBROWSE*******************

 CG9895 in-situ | CG9895 | - | -2381 | -4263 | UPSTREAM | CG9895-RA | "-"
insitu CG2956 in-situ | twi | + | 29726 | 31944 | UPSTREAM | CG2956-RA | "-"

*********************** Rank 1161 [Score 14.116089]   GBROWSE*******************

 CG15414 in-situ | CG15414 | - | -2315 | -3277 | UPSTREAM | CG15414-RA | "-"
insitu CG3234 in-situ | tim | - | 22510 | 9745 | DOWNSTREAM | CG3234-RA | "-" | CG3234-RC | "-" | CG3234-RB | "-"


*********************** Rank 1162 [Score 14.108032]   GBROWSE*******************

 CG10858 in-situ | CG10858 | - | -3470 | -10146 | UPSTREAM | CG10858-RA | "-"
 CG32266 in-situ | CG32266 | + | 32133 | 32891 | UPSTREAM | CG32266-RA | "-"

*********************** Rank 1163 [Score 14.095459]   GBROWSE*******************

 CG15259 in-situ | nht | - | -6159 | -7956 | UPSTREAM | CG15259-RA | "-"
 CG3758 in-situ | esg | + | 7799 | 10085 | UPSTREAM | CG3758-RA | "-"

*********************** Rank 1164 [Score 14.093506]   GBROWSE*******************

insitu highlight CG5461 in-situ | bun | - | -32209 | -116202 | UPSTREAM | CG5461-RA | "-" | CG5461-RB | "-" | CG5461-RC | "-"
 CG15489 in-situ | CG15489 | + | 21008 | 21910 | UPSTREAM | CG15489-RA | "-"

*********************** Rank 1165 [Score 14.091553]   GBROWSE*******************

insitu CG13321 in-situ | CG13321 | + | -12030 | -10411 | DOWNSTREAM | CG13321-RA | "-"
 CG3886 in-situ | Psc | - | 15403 | 724 | DOWNSTREAM | CG3886-RA | "-"


*********************** Rank 1166 [Score 14.090820]   GBROWSE*******************

insitu highlight CG6464 in-situ | salm | - | -8476 | -19768 | UPSTREAM | CG6464-RA | "-"
insitu highlight CG4922 in-situ | sala | + | 32110 | 32876 | UPSTREAM | CG4922-RA | "-"

*********************** Rank 1167 [Score 14.089722]   GBROWSE*******************

 CG14768 in-situ | CG14768 | + | -6029 | -5694 | DOWNSTREAM | CG14768-RA | "-"
 CG2905 in-situ | CG2905 | - | 29298 | -5129 | INTRAGENIC | intron:CG2905-RA:13 | CG2905-RA | "-"


*********************** Rank 1168 [Score 14.087585]   GBROWSE*******************

 CG31437 in-situ | CG31437 | + | -6505 | -5636 | DOWNSTREAM | CG31437-RA | "-"
 CG31439 in-situ | CG31439 | - | 9105 | 8018 | DOWNSTREAM | CG31439-RA | "-"

*********************** Rank 1169 [Score 14.085815]   GBROWSE*******************

insitu CG15281 in-situ | BG:DS00810.3 | + | -5463 | -4856 | DOWNSTREAM | CG15281-RA | "-" | CG15281-RB | "-"
 CG4691 in-situ | BG:DS06874.1 | + | 12051 | 13217 | UPSTREAM | CG4691-RA | "-"


*********************** Rank 1170 [Score 14.081726]   GBROWSE*******************

 CG5142 in-situ | CG5142 | + | -15145 | -12621 | DOWNSTREAM | CG5142-RA | "-"
 CG5122 in-situ | CG5122 | + | 8583 | 12775 | UPSTREAM | CG5122-RA | "-"


*********************** Rank 1171 [Score 14.081543]   GBROWSE*******************

 CG13032 in-situ | CG13032 | + | -29506 | -27204 | DOWNSTREAM | CG13032-RA | "-"
 CG9692 in-situ | CG9692 | + | 1547 | 2964 | UPSTREAM | CG9692-RB | "-" | CG9692-RA | "-"

*********************** Rank 1172 [Score 14.076294]   GBROWSE*******************

 CG12673 in-situ | olf413 | + | -31407 | 27635 | INTRAGENIC | intron:CG12673-RA:2 | CG12673-RA | "-"
 CG9063 in-situ | BcDNA:GH03694 | - | 47723 | 42575 | DOWNSTREAM | CG9063-RA | "-"


*********************** Rank 1173 [Score 14.073120]   GBROWSE*******************

 CG6660 in-situ | CG6660 | + | -18301 | -17421 | DOWNSTREAM | CG6660-RA | "-"
 CG31281 in-situ | CG31281 | + | 24529 | 25689 | UPSTREAM | CG31281-RA | "-"

*********************** Rank 1174 [Score 14.071228]   GBROWSE*******************

 CG15214 in-situ | CG15214 | + | -2796 | 1200 | INTRAGENIC | intron:CG15214-RA:5 | CG15214-RA | "-"
 CG4835 in-situ | CG4835 | + | 41744 | 45271 | UPSTREAM | CG4835-RA | "-"

*********************** Rank 1175 [Score 14.071167]   GBROWSE*******************

insitu CG32369 in-situ | CG32369 | - | -7565 | -33562 | UPSTREAM | CG32369-RA | "-" | CG32369-RB | "-"
 CG17888 in-situ | Pdp1 | - | 55401 | 2364 | DOWNSTREAM | CG17888-RF | "-" | CG17888-RB | "-" | CG17888-RG | "-" | CG17888-RA | "-" | CG17888-RE | "-" | CG17888-RD | "-" | CG17888-RH | "-" | CG17888-RC | "-"

*********************** Rank 1176 [Score 14.057922]   GBROWSE*******************

 CG12190 in-situ | CG12190 | + | -14398 | -11938 | DOWNSTREAM | CG12190-RA | "-"
insitu highlight CG9952 in-situ | ppa | - | 3823 | 375 | DOWNSTREAM | CG9952-RA | "-"

*********************** Rank 1177 [Score 14.055298]   GBROWSE*******************

 CG8472 in-situ | Cam | + | -5279 | 9938 | INTRAGENIC | intron:CG8472-RA:2 | intron:CG8472-RB:1 | CG8472-RA | "-" | CG8472-RB | "-"
 CG13165 in-situ | CG13165 | + | 28730 | 33026 | UPSTREAM | CG13165-RA | "-"

*********************** Rank 1178 [Score 14.053833]   GBROWSE*******************

 CG8510 in-situ | CG8510 | + | -682 | -244 | DOWNSTREAM | CG8510-RA | "-"
 CG8511 in-situ | CG8511 | + | 1149 | 1846 | UPSTREAM | CG8511-RA | "-"

*********************** Rank 1179 [Score 14.051636]   GBROWSE*******************

 CG11958 in-situ | Cnx99A | - | -15168 | -19561 | UPSTREAM | CG11958-RA | "-" | CG11958-RB | "-"
 CG11516 in-situ | CG11516 | + | 91890 | 93943 | UPSTREAM | CG11516-RA | "-"


*********************** Rank 1180 [Score 14.050354]   GBROWSE*******************

 CG6111 in-situ | CG6111 | - | -2821 | -4508 | UPSTREAM | CG6111-RA | "-"
 CG14547 in-situ | CG14547 | - | 12862 | 12467 | DOWNSTREAM | CG14547-RA | "-"

*********************** Rank 1181 [Score 14.047668]   GBROWSE*******************

 CG17382 in-situ | CG17382 | + | -21086 | -19641 | DOWNSTREAM | CG17382-RA | "-"
 CG4370 in-situ | Irk2 | - | 10624 | 5147 | DOWNSTREAM | CG4370-RA | "-" | CG4370-RB | "-"


*********************** Rank 1182 [Score 14.046814]   GBROWSE*******************

 CG3647 in-situ | stc | + | -43083 | -38495 | DOWNSTREAM | CG3647-RB | "-" | CG3647-RA | "-"
 CG4168 in-situ | BG:DS03192.2 | - | 21755 | 5542 | DOWNSTREAM | CG4168-RA | "-"

*********************** Rank 1183 [Score 14.045105]   GBROWSE*******************

insitu CG12911 in-situ | CG12911 | + | -4928 | -341 | DOWNSTREAM | CG12911-RA | "-"
 CG12910 in-situ | CG12910 | + | 5252 | 7590 | UPSTREAM | CG12910-RA | "-"

*********************** Rank 1184 [Score 14.043945]   GBROWSE*******************

 CG9820 in-situ | Or59a | - | -11017 | -12214 | UPSTREAM | CG9820-RA | "-"
 CG5357 in-situ | CG5357 | + | 3893 | 6528 | UPSTREAM | CG5357-RA | "-"

*********************** Rank 1185 [Score 14.042725]   GBROWSE*******************

 CG3151 in-situ | Rbp9 | + | -13469 | -3378 | DOWNSTREAM | CG3151-RA | "-" | CG3151-RD | "-" | CG3151-RB | "-" | CG3151-RE | "-" | CG3151-RC | "-" | CG3151-RF | "-"
insitu CG3181 in-situ | Ts | - | 1389 | 62 | DOWNSTREAM | CG3181-RA | "-"

*********************** Rank 1186 [Score 14.037842]   GBROWSE*******************

 CG13289 in-situ | CG13289 | - | -4464 | -5372 | UPSTREAM | CG13289-RA | "-"
insitu highlight CG13290 in-situ | CG13290 | - | 11513 | -1863 | INTRAGENIC | intron:CG13290-RA:1 | CG13290-RA | "-"

*********************** Rank 1187 [Score 14.036621]   GBROWSE*******************

 CG7906 in-situ | CG7906 | + | -23996 | -22448 | DOWNSTREAM | CG7906-RA | "-"
 CG17697 in-situ | fz | + | 13627 | 107928 | UPSTREAM | CG17697-RB | "-" | CG17697-RA | "-"

*********************** Rank 1188 [Score 14.033142]   GBROWSE*******************

 CG12478 in-situ | bru-3 | - | -52033 | -180864 | UPSTREAM | CG12478-RA | "-" | CG12478-RB | "-"
 CG8757 in-situ | CG8757 | - | 114770 | 113804 | DOWNSTREAM | CG8757-RA | "-"

*********************** Rank 1189 [Score 14.031494]   GBROWSE*******************

 CG9483 in-situ | CG9483 | + | -43652 | -42732 | DOWNSTREAM | CG9483-RA | "-"
 CG15867 in-situ | CG15867 | + | 5416 | 5646 | UPSTREAM | CG15867-RA | "-"

*********************** Rank 1190 [Score 14.026123]   GBROWSE*******************

 CG14565 in-situ | CG14565 | - | -1158 | -2178 | UPSTREAM | CG14565-RA | "-"
 CG14564 in-situ | CG14564 | + | 11735 | 12559 | UPSTREAM | CG14564-RA | "-"

*********************** Rank 1191 [Score 14.025269]   GBROWSE*******************

insitu highlight CG15162 in-situ | MESR3 | + | -27660 | 16870 | INTRAGENIC | intron:CG15162-RA:2 | CG15162-RA | "-"
insitu CG10391 in-situ | Cyp310a1 | - | 8067 | 6140 | DOWNSTREAM | CG10391-RA | "-"

*********************** Rank 1192 [Score 14.018066]   GBROWSE*******************

 CG31637 in-situ | CG31637 | + | -4242 | 24112 | INTRAGENIC | intron:CG31637-RA:1 | CG31637-RA | "-"
insitu highlight CG9554 in-situ | eya | - | 44088 | 24565 | DOWNSTREAM | CG9554-RB | "-" | CG9554-RA | "-"


*********************** Rank 1193 [Score 14.017578]   GBROWSE*******************

 CG17330 in-situ | BG:DS09218.5 | + | -75147 | -74089 | DOWNSTREAM | CG17330-RA | "-"
 CG4472 in-situ | Idgf1 | + | 4214 | 5781 | UPSTREAM | CG4472-RA | "-"

*********************** Rank 1194 [Score 14.015808]   GBROWSE*******************

 CG4429 in-situ | Rbp2 | + | -33882 | -30405 | DOWNSTREAM | CG4429-RA | "-" | CG4429-RB | "-"
 CG9906 in-situ | CG9906 | - | 42641 | 40722 | DOWNSTREAM | CG9906-RA | "-"


*********************** Rank 1195 [Score 14.013733]   GBROWSE*******************

 CG12756 in-situ | CG12756 | - | -652 | -1733 | UPSTREAM | CG12756-RA | "-"
insitu highlight CG5249 in-situ | CG5249 | + | 22091 | 40057 | UPSTREAM | CG5249-RA | "-"

*********************** Rank 1196 [Score 14.011108]   GBROWSE*******************

 CG7573 in-situ | CG7573 | + | -1914 | 2929 | INTRAGENIC | intron:CG7573-RA:1 | CG7573-RA | "-" | CG7573-RB | "-"
 CG6168 in-situ | CG6168 | - | 11251 | 10223 | DOWNSTREAM | CG6168-RB | "-"

*********************** Rank 1197 [Score 14.010620]   GBROWSE*******************

 CG10037 in-situ | vvl | + | -64061 | -59685 | DOWNSTREAM | CG10037-RA | "-"
insitu CG10078 in-situ | Prat2 | - | 60637 | 57772 | DOWNSTREAM | CG10078-RB | "-" | CG10078-RA | "-"

*********************** Rank 1198 [Score 14.010193]   GBROWSE*******************

insitu CG31337 in-situ | CG31337 | - | -31325 | -32737 | UPSTREAM | CG31337-RA | "-"
 CG14370 in-situ | CG14370 | + | 11478 | 11876 | UPSTREAM | CG14370-RA | "-"

*********************** Rank 1199 [Score 14.010193]   GBROWSE*******************

 CG1088 in-situ | Vha26 | + | -4750 | -1774 | DOWNSTREAM | CG1088-RB | "-" | CG1088-RA | "-"
 CG2922 in-situ | eIF-5C | - | 4688 | -1117 | INTRAGENIC | intron:CG2922-RA:7 | intron:CG2922-RG:7 | intron:CG2922-RC:8 | intron:CG2922-RF:7 | intron:CG2922-RD:7 | intron:CG2922-RB:8 | intron:CG2922-RE:6 | CG2922-RA | "-" | CG2922-RG | "-" | CG2922-RC | "-" | CG2922-RF | "-" | CG2922-RD | "-" | CG2922-RB | "-" | CG2922-RE | "-"

*********************** Rank 1200 [Score 14.010010]   GBROWSE*******************

insitu CG10710 in-situ | CG10710 | - | -67031 | -70359 | UPSTREAM | CG10710-RA | "-"
 CG12478 in-situ | bru-3 | - | 80117 | -48714 | INTRAGENIC | intron:CG12478-RA:5 | intron:CG12478-RB:3 | CG12478-RA | "-" | CG12478-RB | "-"

*********************** Rank 1201 [Score 14.008545]   GBROWSE*******************

insitu CG18507 in-situ | BG:DS01368.1 | + | -27320 | -21994 | DOWNSTREAM | CG18507-RA | "-" | CG18507-RB | "-"
 CG7311 in-situ | BG:DS08249.2 | + | 15367 | 17744 | UPSTREAM | CG7311-RA | "-" | CG7311-RC | "-"

*********************** Rank 1202 [Score 14.006226]   GBROWSE*******************

 CG18478 in-situ | BG:DS07108.5 | + | -2127 | -1143 | DOWNSTREAM | CG18478-RA | "-"
 CG31780 in-situ | CG31780 | + | 23677 | 25563 | UPSTREAM | CG31780-RB | "-" | CG31780-RA | "-"

*********************** Rank 1203 [Score 14.003906]   GBROWSE*******************

 CG3759 in-situ | CG3759 | + | -1422 | 8329 | INTRAGENIC | intron:CG3759-RA:1 | CG3759-RA | "-"
 CG3763 in-situ | Fbp2 | + | 8857 | 9821 | UPSTREAM | CG3763-RA | "-"


*********************** Rank 1204 [Score 14.002075]   GBROWSE*******************

 CG8756 in-situ | CG8756 | - | -3062 | -7787 | UPSTREAM | CG8756-RB | "-" | CG8756-RA | "-" | CG8756-RC | "-" | CG8756-RD | "-"
insitu CG32209 in-situ | CG32209 | - | 9716 | 2684 | DOWNSTREAM | CG32209-RB | "-"


*********************** Rank 1205 [Score 14.001831]   GBROWSE*******************

 CG31820 in-situ | CG31820 | - | -34887 | -35398 | UPSTREAM | CG31820-RA | "-"
 CG4824 in-situ | BicC | + | 866 | 7460 | UPSTREAM | CG4824-RA | "-" | CG4824-RB | "-" | CG4824-RD | "-"

*********************** Rank 1206 [Score 14.000732]   GBROWSE*******************

 CG12756 in-situ | CG12756 | - | -15552 | -16633 | UPSTREAM | CG12756-RA | "-"
insitu highlight CG5249 in-situ | CG5249 | + | 7191 | 25157 | UPSTREAM | CG5249-RA | "-"

*********************** Rank 1207 [Score 13.999573]   GBROWSE*******************

 CG7370 in-situ | CG7370 | + | -26557 | -25046 | DOWNSTREAM | CG7370-RA | "-"
 CG7383 in-situ | eg | + | 12331 | 21609 | UPSTREAM | CG7383-RB | "-" | CG7383-RA | "-"

*********************** Rank 1208 [Score 13.996155]   GBROWSE*******************

 CG14494 in-situ | CG14494 | - | -23191 | -23406 | UPSTREAM | CG14494-RA | "-"
 CG5084 in-situ | CG5084 | + | 3476 | 4596 | UPSTREAM | CG5084-RA | "-"

*********************** Rank 1209 [Score 13.994751]   GBROWSE*******************

 CG32115 in-situ | CG32115 | - | -19107 | -20713 | UPSTREAM | CG32115-RA | "-"
 CG10752 in-situ | CG10752 | - | 21809 | 19930 | DOWNSTREAM | CG10752-RA | "-"


*********************** Rank 1210 [Score 13.993164]   GBROWSE*******************

insitu CG14039 in-situ | qtc | + | -8097 | 1826 | INTRAGENIC | intron:CG14039-RA:5 | intron:CG14039-RB:4 | intron:CG14039-RC:4 | intron:CG14039-RD:4 | CG14039-RA | "-" | CG14039-RB | "-" | CG14039-RC | "-" | CG14039-RD | "-"
 CG5827 in-situ | RpL37a | + | 2074 | 3126 | UPSTREAM | CG5827-RA | "-" | CG5827-RB | "-"

*********************** Rank 1211 [Score 13.991455]   GBROWSE*******************

 CG3915 in-situ | Drl-2 | - | -22048 | -45024 | UPSTREAM | CG3915-RB | "-" | CG3915-RA | "-"
 CG13325 in-situ | CG13325 | + | 14719 | 24194 | UPSTREAM | CG13325-RA | "-"


*********************** Rank 1212 [Score 13.989502]   GBROWSE*******************

insitu CG15105 in-situ | CG15105 | + | -19339 | 2264 | INTRAGENIC | intron:CG15105-RB:9 | intron:CG15105-RA:8 | CG15105-RB | "-" | CG15105-RA | "-"
 CG15113 in-situ | 5-HT1B | - | 22297 | 16234 | DOWNSTREAM | CG15113-RA | "-"

*********************** Rank 1213 [Score 13.989014]   GBROWSE*******************

insitu CG9614 in-situ | pip | - | -4472 | -43541 | UPSTREAM | CG9614-RA | "-" | CG9614-RC | "-" | CG9614-RD | "-" | CG9614-RE | "-" | CG9614-RF | "-" | CG9614-RG | "-" | CG9614-RH | "-" | CG9614-RI | "-" | CG9614-RJ | "-" | CG9614-RK | "-" | CG9614-RL | "-"
 CG14087 in-situ | CG14087 | + | 45531 | 47962 | UPSTREAM | CG14087-RA | "-"

*********************** Rank 1214 [Score 13.987183]   GBROWSE*******************

insitu highlight CG9598 in-situ | CG9598 | - | -36038 | -40547 | UPSTREAM | CG9598-RA | "-"
 CG9587 in-situ | CG9587 | - | 59878 | 54236 | DOWNSTREAM | CG9587-RA | "-"

*********************** Rank 1215 [Score 13.986816]   GBROWSE*******************

 CG32121 in-situ | CG32121 | - | -1874 | -3929 | UPSTREAM | CG32121-RA | "-"
 CG14106 in-situ | CG14106 | - | 4336 | 3238 | DOWNSTREAM | CG14106-RA | "-"


*********************** Rank 1216 [Score 13.985718]   GBROWSE*******************

 CG13872 in-situ | CG13872 | - | -47817 | -49909 | UPSTREAM | CG13872-RA | "-"
 CG30447 in-situ | CG30447 | + | 18579 | 19120 | UPSTREAM | CG30447-RA | "-"

*********************** Rank 1217 [Score 13.985046]   GBROWSE*******************

 CG17025 in-situ | CG17025 | - | -9503 | -17274 | UPSTREAM | CG17025-RA | "-"
 CG12538 in-situ | CG12538 | - | 47926 | 47342 | DOWNSTREAM | CG12538-RA | "-"


*********************** Rank 1218 [Score 13.984863]   GBROWSE*******************

 CG4956 in-situ | CG4956 | - | -7135 | -7992 | UPSTREAM | CG4956-RA | "-"
 CG4960 in-situ | CG4960 | - | 1909 | 1202 | DOWNSTREAM | CG4960-RA | "-"

*********************** Rank 1219 [Score 13.982788]   GBROWSE*******************

 CG17326 in-situ | CG17326 | - | -5941 | -8116 | UPSTREAM | CG17326-RA | "-"
 CG13235 in-situ | CG13235 | + | 83528 | 83695 | UPSTREAM | CG13235-RA | "-"

*********************** Rank 1220 [Score 13.981934]   GBROWSE*******************

 CG17769 in-situ | And | + | -2524 | -1861 | DOWNSTREAM | CG17769-RA | "-"
 CG14354 in-situ | CG14354 | + | 416 | 1511 | UPSTREAM | CG14354-RA | "-"


*********************** Rank 1221 [Score 13.977051]   GBROWSE*******************

 CG9967 in-situ | CG9967 | - | -851 | -49422 | UPSTREAM | CG9967-RA | "-"
 CG4272 in-situ | BcDNA:GH09817 | + | 7152 | 11931 | UPSTREAM | CG4272-RA | "-" | CG4272-RB | "-"

*********************** Rank 1222 [Score 13.976562]   GBROWSE*******************

 CG31759 in-situ | CG31759 | + | -102952 | -100460 | DOWNSTREAM | CG31759-RB | "-" | CG31759-RA | "-"
 CG31862 in-situ | CG31862 | + | 28291 | 28839 | UPSTREAM | CG31862-RA | "-"

*********************** Rank 1223 [Score 13.971741]   GBROWSE*******************

 CG6989 in-situ | CG6989 | - | -3423 | -5665 | UPSTREAM | CG6989-RA | "-"
 CG18553 in-situ | CG18553 | - | 2645 | 1743 | DOWNSTREAM | CG18553-RA | "-"

*********************** Rank 1224 [Score 13.971313]   GBROWSE*******************

 CG15623 in-situ | CG15623 | - | -1978 | -2743 | UPSTREAM | CG15623-RA | "-"
 CG31665 in-situ | CG31665 | + | 8464 | 24016 | UPSTREAM | CG31665-RA | "-"

*********************** Rank 1225 [Score 13.969482]   GBROWSE*******************

insitu CG4623 in-situ | CG4623 | + | -786 | 1578 | INTRAGENIC | intron:CG4623-RA:1 | CG4623-RA | "-"
insitu CG4633 in-situ | Aats-ala-m | + | 3364 | 7113 | UPSTREAM | CG4633-RA | "-"

*********************** Rank 1226 [Score 13.967834]   GBROWSE*******************

 CG13315 in-situ | CG13315 | + | -22464 | -21844 | DOWNSTREAM | CG13315-RA | "-"
 CG4760 in-situ | bol | - | 11012 | -17714 | INTRAGENIC | intron:CG4760-RB:1 | intron:CG4760-RC:1 | intron:CG4760-RD:2 | intron:CG4760-RA:2 | CG4760-RB | "-" | CG4760-RC | "-" | CG4760-RD | "-" | CG4760-RA | "-"

*********************** Rank 1227 [Score 13.966431]   GBROWSE*******************

 CG30405 in-situ | CG30405 | - | -15635 | -18243 | UPSTREAM | CG30405-RA | "-"
 CG9308 in-situ | CG9308 | + | 23672 | 24700 | UPSTREAM | CG9308-RA | "-"

*********************** Rank 1228 [Score 13.958496]   GBROWSE*******************

 CG11994 in-situ | Ada | + | -79784 | -78711 | DOWNSTREAM | CG11994-RA | "-"
 CG11997 in-situ | CG11997 | + | 32600 | 33910 | UPSTREAM | CG11997-RA | "-"

*********************** Rank 1229 [Score 13.956787]   GBROWSE*******************

 CG16995 in-situ | CG16995 | + | -5206 | -4562 | DOWNSTREAM | CG16995-RA | "-"
 CG15388 in-situ | CG15388 | + | 24731 | 27876 | UPSTREAM | CG15388-RA | "-"

*********************** Rank 1230 [Score 13.956543]   GBROWSE*******************

 CG14915 in-situ | CG14915 | + | -4330 | -3968 | DOWNSTREAM | CG14915-RA | "-"
insitu CG14919 in-situ | Ast2 | - | 6006 | 761 | DOWNSTREAM | CG14919-RA | "-"

*********************** Rank 1231 [Score 13.956543]   GBROWSE*******************

 CG6545 in-situ | lbe | - | -3244 | -7972 | UPSTREAM | CG6545-RA | "-"
 CG7922 in-situ | CG7922 | + | 32761 | 37318 | UPSTREAM | CG7922-RA | "-"


*********************** Rank 1232 [Score 13.952942]   GBROWSE*******************

 CG17047 in-situ | CG17047 | + | -18531 | -16714 | DOWNSTREAM | CG17047-RA | "-"
 CG17048 in-situ | CG17048 | - | 36147 | 35623 | DOWNSTREAM | CG17048-RA | "-"

*********************** Rank 1233 [Score 13.944824]   GBROWSE*******************

 CG8585 in-situ | Ih | + | -5889 | 7484 | INTRAGENIC | intron:CG8585-RA:2 | CG8585-RA | "-"
 CG8589 in-situ | CG8589 | - | 10929 | 8676 | DOWNSTREAM | CG8589-RA | "-"


*********************** Rank 1234 [Score 13.944092]   GBROWSE*******************

 CG1072 in-situ | Awh | + | -24418 | -8874 | DOWNSTREAM | CG1072-RA | "-" | CG1072-RB | "-"
 CG1079 in-situ | BEST:HL04053 | + | 22003 | 24262 | UPSTREAM | CG1079-RA | "-"

*********************** Rank 1235 [Score 13.943604]   GBROWSE*******************

 CG14045 in-situ | EG:BACH7M4.1 | - | -464 | -10010 | UPSTREAM | CG14045-RA | "-"
 CG12496 in-situ | EG:BACH7M4.4 | - | 22771 | 21229 | DOWNSTREAM | CG12496-RA | "-"

*********************** Rank 1236 [Score 13.943237]   GBROWSE*******************

 CG31082 in-situ | CG31082 | + | -9853 | -7412 | DOWNSTREAM | CG31082-RA | "-"
insitu CG5467 in-situ | CG5467 | + | 3459 | 9849 | UPSTREAM | CG5467-RA | "-"

*********************** Rank 1237 [Score 13.942749]   GBROWSE*******************

 CG4645 in-situ | CG4645 | + | -19337 | -17375 | DOWNSTREAM | CG4645-RA | "-"
 CG4396 in-situ | fne | + | 13310 | 16513 | UPSTREAM | CG4396-RA | "-"

*********************** Rank 1238 [Score 13.942139]   GBROWSE*******************

 CG4069 in-situ | CG4069 | - | -25293 | -27179 | UPSTREAM | CG4069-RA | "-"
 CG10632 in-situ | CG10632 | - | 9919 | -25094 | INTRAGENIC | intron:CG10632-RA:2 | CG10632-RA | "-" | CG10632-RB | "-"

*********************** Rank 1239 [Score 13.937378]   GBROWSE*******************

 CG6739 in-situ | CG6739 | + | -32379 | -24241 | DOWNSTREAM | CG6739-RA | "-"
 CG13792 in-situ | CG13792 | - | 5228 | 2758 | DOWNSTREAM | CG13792-RA | "-"


*********************** Rank 1240 [Score 13.932678]   GBROWSE*******************

 CG31172 in-situ | CG31172 | - | -12070 | -12938 | UPSTREAM | CG31172-RA | "-"
 CG13627 in-situ | CG13627 | + | 29513 | 34282 | UPSTREAM | CG13627-RA | "-" | CG13627-RB | "-"

*********************** Rank 1241 [Score 13.931885]   GBROWSE*******************

 CG30405 in-situ | CG30405 | - | -585 | -3193 | UPSTREAM | CG30405-RA | "-"
 CG9308 in-situ | CG9308 | + | 38722 | 39750 | UPSTREAM | CG9308-RA | "-"

*********************** Rank 1242 [Score 13.931152]   GBROWSE*******************

 CG31802 in-situ | CG31802 | - | -3530 | -4353 | UPSTREAM | CG31802-RA | "-"
 CG31786 in-situ | CG31786 | - | 3086 | -3028 | INTRAGENIC | intron:CG31786-RA:2 | CG31786-RA | "-"

*********************** Rank 1243 [Score 13.930847]   GBROWSE*******************

insitu CG7891 in-situ | CG7891 | + | -30899 | -28667 | DOWNSTREAM | CG7891-RA | "-"
 CG9656 in-situ | grn | - | 23929 | -10163 | INTRAGENIC | intron:CG9656-RA:5 | CG9656-RA | "-"

*********************** Rank 1244 [Score 13.928955]   GBROWSE*******************

 CG3474 in-situ | BG:DS06238.4 | - | -24617 | -25335 | UPSTREAM | CG3474-RA | "-"
 CG15283 in-situ | BG:DS08340.1 | - | 1068 | -2371 | INTRAGENIC | intron:CG15283-RA:1 | CG15283-RA | "-"

*********************** Rank 1245 [Score 13.927185]   GBROWSE*******************

 CG10152 in-situ | beat-IV | + | -29180 | -7057 | DOWNSTREAM | CG10152-RA | "-"
 CG10182 in-situ | CG10182 | - | 3194 | 586 | DOWNSTREAM | CG10182-RA | "-"

*********************** Rank 1246 [Score 13.926880]   GBROWSE*******************

insitu CG32159 in-situ | CG32159 | - | -15217 | -44377 | UPSTREAM | CG32159-RB | "-"
insitu highlight CG4531 in-situ | argos | - | 16530 | 3208 | DOWNSTREAM | CG4531-RA | "-"

*********************** Rank 1247 [Score 13.924805]   GBROWSE*******************

 CG10491 in-situ | vn | - | -24678 | -56602 | UPSTREAM | CG10491-RA | "-"
 CG5568 in-situ | CG5568 | + | 1210 | 3227 | UPSTREAM | CG5568-RA | "-"

*********************** Rank 1248 [Score 13.913940]   GBROWSE*******************

 CG3058 in-situ | CG3058 | + | -1776 | -1076 | DOWNSTREAM | CG3058-RA | "-"
 CG15431 in-situ | CG15431 | - | 4322 | 1098 | DOWNSTREAM | CG15431-RA | "-"


*********************** Rank 1249 [Score 13.913330]   GBROWSE*******************

 CG16758 in-situ | CG16758 | - | -9137 | -14017 | UPSTREAM | CG16758-RB | "-" | CG16758-RD | "-" | CG16758-RC | "-"
 CG32295 in-situ | CG32295 | - | 14251 | 13096 | DOWNSTREAM | CG32295-RA | "-"


*********************** Rank 1250 [Score 13.911865]   GBROWSE*******************

 CG1877 in-situ | lin19 | + | -3143 | 978 | INTRAGENIC | intron:CG1877-RC:4 | intron:CG1877-RB:3 | intron:CG1877-RA:3 | intron:CG1877-RD:3 | CG1877-RC | "-" | CG1877-RB | "-" | CG1877-RA | "-" | CG1877-RD | "-"
 CG17853 in-situ | Or43b | - | 2544 | 975 | DOWNSTREAM | CG17853-RA | "-"


*********************** Rank 1251 [Score 13.910522]   GBROWSE*******************

insitu highlight CG4702 in-situ | CG4702 | - | -6162 | -12782 | UPSTREAM | CG4702-RA | "-"
 CG10095 in-situ | CG10095 | - | 24236 | 4474 | DOWNSTREAM | CG10095-RA | "-"

*********************** Rank 1252 [Score 13.908813]   GBROWSE*******************

 CG7313 in-situ | CG7313 | + | -13174 | -12425 | DOWNSTREAM | CG7313-RA | "-"
 CG5103 in-situ | CG5103 | - | 29096 | 26846 | DOWNSTREAM | CG5103-RA | "-"


*********************** Rank 1253 [Score 13.901611]   GBROWSE*******************

insitu CG11337 in-situ | CG11337 | - | -48610 | -52227 | UPSTREAM | CG11337-RB | "-" | CG11337-RA | "-"
 CG11335 in-situ | lox | - | 5665 | 4522 | DOWNSTREAM | CG11335-RA | "-"

*********************** Rank 1254 [Score 13.901123]   GBROWSE*******************

 CG2014 in-situ | CG2014 | + | -30517 | -29879 | DOWNSTREAM | CG2014-RA | "-"
insitu highlight CG1897 in-situ | Dr | + | 10025 | 18923 | UPSTREAM | CG1897-RA | "-"

*********************** Rank 1255 [Score 13.898071]   GBROWSE*******************

 CG15532 in-situ | hdc | + | -54228 | 30007 | INTRAGENIC | intron:CG15532-RA:2 | intron:CG15532-RC:2 | CG15532-RA | "-" | CG15532-RC | "-" | CG15532-RB | "-"
 CG1469 in-situ | Fer2LCH | + | 55670 | 58422 | UPSTREAM | CG1469-RA | "-" | CG1469-RB | "-" | CG1469-RC | "-"

*********************** Rank 1256 [Score 13.897583]   GBROWSE*******************

 CG32140 in-situ | CG32140 | + | -13575 | -6839 | DOWNSTREAM | CG32140-RA | "-" | CG32140-RB | "-"
 CG7924 in-situ | CG7924 | + | 149 | 1290 | UPSTREAM | CG7924-RA | "-"

*********************** Rank 1257 [Score 13.895874]   GBROWSE*******************

 CG13482 in-situ | CG13482 | + | -24833 | -24525 | DOWNSTREAM | CG13482-RA | "-"
 CG13481 in-situ | CG13481 | - | 15373 | 14851 | DOWNSTREAM | CG13481-RA | "-"

*********************** Rank 1258 [Score 13.895264]   GBROWSE*******************

 CG11192 in-situ | CG11192 | - | -43665 | -44474 | UPSTREAM | CG11192-RA | "-"
 CG33041 in-situ | CG33041 | + | 25647 | 72276 | UPSTREAM | CG33041-RA | "-"

*********************** Rank 1259 [Score 13.895142]   GBROWSE*******************

insitu highlight CG4345 in-situ | grim | - | -83457 | -85152 | UPSTREAM | CG4345-RA | "-"
 CG4319 in-situ | rpr | - | 10532 | 9682 | DOWNSTREAM | CG4319-RA | "-"


*********************** Rank 1260 [Score 13.894897]   GBROWSE*******************

 CG30125 in-situ | CG30125 | - | -16506 | -18413 | UPSTREAM | CG30125-RA | "-"
insitu CG15117 in-situ | CG15117 | - | 16561 | 9550 | DOWNSTREAM | CG15117-RA | "-" | CG15117-RB | "-"

*********************** Rank 1261 [Score 13.892700]   GBROWSE*******************

insitu highlight CG2189 in-situ | Dfd | + | -55441 | -44846 | DOWNSTREAM | CG2189-RA | "-"
insitu highlight CG1030 in-situ | Scr | - | 1342 | -24157 | INTRAGENIC | intron:CG1030-RA:1 | CG1030-RA | "-"

*********************** Rank 1262 [Score 13.890259]   GBROWSE*******************

 CG15816 in-situ | CG15816 | + | -8767 | -6944 | DOWNSTREAM | CG15816-RA | "-"
 CG6269 in-situ | unc-4 | + | 21342 | 32842 | UPSTREAM | CG6269-RA | "-"

*********************** Rank 1263 [Score 13.890198]   GBROWSE*******************

 CG3717 in-situ | bcn92 | - | -478 | -1631 | UPSTREAM | CG3717-RA | "-"
 CG3707 in-situ | wapl | - | 8905 | 24 | DOWNSTREAM | CG3707-RA | "-" | CG3707-RB | "-"


*********************** Rank 1264 [Score 13.885498]   GBROWSE*******************

 CG5610 in-situ | nAcRalpha-96Aa | - | -9542 | -65245 | UPSTREAM | CG5610-RA | "-"
 CG31128 in-situ | CG31128 | + | 2926 | 3848 | UPSTREAM | CG31128-RA | "-"

*********************** Rank 1265 [Score 13.879822]   GBROWSE*******************

insitu CG5467 in-situ | CG5467 | + | -15141 | -8751 | DOWNSTREAM | CG5467-RA | "-"
 CG5468 in-situ | CG5468 | + | 6310 | 7418 | UPSTREAM | CG5468-RA | "-"

*********************** Rank 1266 [Score 13.879028]   GBROWSE*******************

 CG32030 in-situ | CG32030 | + | -8196 | 24085 | INTRAGENIC | intron:CG32030-RA:1 | intron:CG32030-RB:1 | CG32030-RA | "-" | CG32030-RB | "-"
insitu CG5804 in-situ | CG5804 | - | 13900 | 13502 | DOWNSTREAM | CG5804-RA | "-"

*********************** Rank 1267 [Score 13.878357]   GBROWSE*******************

 CG7147 in-situ | kuz | + | -18958 | 69165 | INTRAGENIC | intron:CG7147-RA:3 | intron:CG7147-RB:3 | CG7147-RA | "-" | CG7147-RB | "-"
 CG9254 in-situ | BG:DS07660.1 | - | 28087 | 26403 | DOWNSTREAM | CG9254-RA | "-"

*********************** Rank 1268 [Score 13.875122]   GBROWSE*******************

insitu highlight CG15162 in-situ | MESR3 | + | -2210 | 42320 | INTRAGENIC | intron:CG15162-RA:1 | CG15162-RA | "-"
insitu CG10391 in-situ | Cyp310a1 | - | 33517 | 31590 | DOWNSTREAM | CG10391-RA | "-"

*********************** Rank 1269 [Score 13.871460]   GBROWSE*******************

insitu CG1273 in-situ | CG1273 | + | -2203 | 11858 | INTRAGENIC | intron:CG1273-RB:1 | CG1273-RB | "-"
 CG1311 in-situ | CG1311 | + | 12040 | 15694 | UPSTREAM | CG1311-RA | "-"

*********************** Rank 1270 [Score 13.871277]   GBROWSE*******************

insitu CG7437 in-situ | mub | + | -44643 | 1954 | INTRAGENIC | intron:CG7437-RA:8 | intron:CG7437-RB:8 | CG7437-RA | "-" | CG7437-RB | "-"
 CG7442 in-situ | CG7442 | + | 10691 | 14245 | UPSTREAM | CG7442-RA | "-"


*********************** Rank 1271 [Score 13.869629]   GBROWSE*******************

 CG1379 in-situ | CG1379 | - | -25020 | -31959 | UPSTREAM | CG1379-RA | "-"
 CG11692 in-situ | CG11692 | - | 8128 | 7572 | DOWNSTREAM | CG11692-RA | "-"


*********************** Rank 1272 [Score 13.868958]   GBROWSE*******************

 CG15405 in-situ | CG15405 | - | -50245 | -54594 | UPSTREAM | CG15405-RA | "-"
 CG3347 in-situ | CG3347 | + | 16671 | 28565 | UPSTREAM | CG3347-RA | "-"


*********************** Rank 1273 [Score 13.867065]   GBROWSE*******************

insitu CG12926 in-situ | CG12926 | - | -49384 | -51541 | UPSTREAM | CG12926-RA | "-"
 CG1794 in-situ | Mmp2 | - | 26997 | -47576 | INTRAGENIC | intron:CG1794-RA:6 | CG1794-RA | "-"

*********************** Rank 1274 [Score 13.866455]   GBROWSE*******************

 CG31537 in-situ | CG31537 | - | -10493 | -12457 | UPSTREAM | CG31537-RA | "-"
 CG2534 in-situ | cno | - | 26244 | -17583 | INTRAGENIC | intron:CG2534-RA:6 | intron:CG2534-RB:6 | CG2534-RA | "-" | CG2534-RB | "-"

*********************** Rank 1275 [Score 13.864563]   GBROWSE*******************

 CG31665 in-situ | CG31665 | + | -25236 | -9684 | DOWNSTREAM | CG31665-RA | "-"
 CG7295 in-situ | CG7295 | + | 1632 | 2721 | UPSTREAM | CG7295-RA | "-"

*********************** Rank 1276 [Score 13.864136]   GBROWSE*******************

 CG15476 in-situ | CG15476 | - | -17458 | -17724 | UPSTREAM | CG15476-RA | "-"
 CG15477 in-situ | CG15477 | + | 25033 | 25551 | UPSTREAM | CG15477-RA | "-"


*********************** Rank 1277 [Score 13.862061]   GBROWSE*******************

 CG14184 in-situ | CG14184 | - | -48110 | -49095 | UPSTREAM | CG14184-RA | "-"
 CG7395 in-situ | NPFR76F | - | 11920 | -17066 | INTRAGENIC | intron:CG7395-RA:1 | CG7395-RA | "-"

*********************** Rank 1278 [Score 13.860962]   GBROWSE*******************

insitu highlight CG2102 in-situ | cas | - | -3181 | -7539 | UPSTREAM | CG2102-RA | "-" | CG2102-RB | "-"
 CG1239 in-situ | CG1239 | + | 13060 | 14221 | UPSTREAM | CG1239-RA | "-"

*********************** Rank 1279 [Score 13.854858]   GBROWSE*******************

insitu highlight CG33207 in-situ | CG33207 | + | -12224 | 9526 | INTRAGENIC | intron:CG33207-RB:2 | CG33207-RA | "-" | CG33207-RB | "-"
 CG31446 in-situ | CG31446 | + | 14679 | 15788 | UPSTREAM | CG31446-RA | "-"

*********************** Rank 1280 [Score 13.852661]   GBROWSE*******************

 CG31729 in-situ | CG31729 | + | -2574 | 9080 | INTRAGENIC | intron:CG31729-RB:1 | CG31729-RB | "-" | CG31729-RA | "-"
 CG16825 in-situ | CG16825 | + | 14008 | 15227 | UPSTREAM | CG16825-RA | "-"

*********************** Rank 1281 [Score 13.852539]   GBROWSE*******************

 CG14426 in-situ | nullo | + | -13853 | -12890 | DOWNSTREAM | CG14426-RA | "-"
 CG32734 in-situ | CG32734 | + | 143 | 782 | UPSTREAM | CG32734-RA | "-"

*********************** Rank 1282 [Score 13.849731]   GBROWSE*******************

 CG4750 in-situ | CG4750 | + | -3546 | -1439 | DOWNSTREAM | CG4750-RA | "-"
 CG4905 in-situ | Syn2 | + | 13735 | 19958 | UPSTREAM | CG4905-RA | "-" | CG4905-RB | "-" | CG4905-RC | "-" | CG4905-RD | "-" | CG4905-RE | "-"


*********************** Rank 1283 [Score 13.848267]   GBROWSE*******************

 CG4162 in-situ | lace | + | -44456 | -38968 | DOWNSTREAM | CG4162-RA | "-"
 CG15256 in-situ | BG:DS04862.2 | + | 6906 | 19684 | UPSTREAM | CG15256-RA | "-"

*********************** Rank 1284 [Score 13.847168]   GBROWSE*******************

 CG13109 in-situ | tai | + | -19217 | 60371 | INTRAGENIC | intron:CG13109-RA:1 | CG13109-RA | "-"
 CG17009 in-situ | CG17009 | - | 64993 | 64277 | DOWNSTREAM | CG17009-RA | "-"


*********************** Rank 1285 [Score 13.846924]   GBROWSE*******************

insitu CG18136 in-situ | CG18136 | - | -22228 | -24439 | UPSTREAM | CG18136-RA | "-"
 CG11614 in-situ | nkd | - | 19810 | -21789 | INTRAGENIC | intron:CG11614-RA:1 | CG11614-RA | "-"

*********************** Rank 1286 [Score 13.844788]   GBROWSE*******************

 CG18563 in-situ | CG18563 | - | -4636 | -5662 | UPSTREAM | CG18563-RA | "-"
insitu CG6605 in-situ | BicD | - | 10838 | -1583 | INTRAGENIC | intron:CG6605-RA:6 | CG6605-RA | "-"

*********************** Rank 1287 [Score 13.844177]   GBROWSE*******************

 CG4659 in-situ | Srp54k | + | -4222 | -2012 | DOWNSTREAM | CG4659-RA | "-"
 CG10677 in-situ | CG10677 | + | 26132 | 28060 | UPSTREAM | CG10677-RA | "-"

*********************** Rank 1288 [Score 13.839722]   GBROWSE*******************

 CG9425 in-situ | CG9425 | + | -7639 | -420 | DOWNSTREAM | CG9425-RA | "-"
insitu CG5258 in-situ | NHP2 | - | 2419 | 1620 | DOWNSTREAM | CG5258-RA | "-"

*********************** Rank 1289 [Score 13.837891]   GBROWSE*******************

 CG17697 in-situ | fz | + | -8823 | 85478 | INTRAGENIC | intron:CG17697-RB:1 | intron:CG17697-RA:1 | CG17697-RB | "-" | CG17697-RA | "-"
 CG13482 in-situ | CG13482 | + | 46267 | 46575 | UPSTREAM | CG13482-RA | "-"

*********************** Rank 1290 [Score 13.836365]   GBROWSE*******************

insitu CG4863 in-situ | RpL3 | + | -1864 | 1415 | INTRAGENIC | intron:CG4863-RA:4 | intron:CG4863-RE:3 | intron:CG4863-RB:3 | CG4863-RA | "-" | CG4863-RD | "-" | CG4863-RE | "-" | CG4863-RB | "-" | CG4863-RC | "-"
 CG5106 in-situ | CG5106 | + | 1760 | 2830 | UPSTREAM | CG5106-RA | "-"


*********************** Rank 1291 [Score 13.833984]   GBROWSE*******************

 CG5685 in-situ | Calx | + | -12935 | 23249 | INTRAGENIC | intron:CG5685-RA:1 | intron:CG5685-RB:2 | intron:CG5685-RC:1 | CG5685-RA | "-" | CG5685-RB | "-" | CG5685-RC | "-"
 CG10827 in-situ | CG10827 | - | 15776 | 13948 | DOWNSTREAM | CG10827-RA | "-"

*********************** Rank 1292 [Score 13.831055]   GBROWSE*******************

insitu CG3478 in-situ | ppk | - | -2128 | -5080 | UPSTREAM | CG3478-RA | "-"
 CG4220 in-situ | elB | - | 24460 | 3824 | DOWNSTREAM | CG4220-RA | "-" | CG4220-RB | "-"

*********************** Rank 1293 [Score 13.823120]   GBROWSE*******************

 CG10032 in-situ | CG10032 | + | -10867 | -9950 | DOWNSTREAM | CG10032-RA | "-"
 CG2595 in-situ | RacGAP84C | + | 2474 | 5173 | UPSTREAM | CG2595-RA | "-" | CG2595-RB | "-"

*********************** Rank 1294 [Score 13.823059]   GBROWSE*******************

 CG4774 in-situ | CG4774 | - | -35343 | -37479 | UPSTREAM | CG4774-RB | "-" | CG4774-RA | "-" | CG4774-RC | "-"
insitu CG31092 in-situ | CG31092 | - | 17752 | -23947 | INTRAGENIC | intron:CG31092-RA:2 | CG31092-RA | "-" | CG31092-RB | "-"

*********************** Rank 1295 [Score 13.817261]   GBROWSE*******************

 CG4154 in-situ | CG4154 | + | -3124 | 2273 | INTRAGENIC | intron:CG4154-RA:6 | intron:CG4154-RC:6 | CG4154-RA | "-" | CG4154-RC | "-"
insitu CG6752 in-situ | CG6752 | - | 8900 | 3240 | DOWNSTREAM | CG6752-RA | "-"

*********************** Rank 1296 [Score 13.812134]   GBROWSE*******************

insitu CG7891 in-situ | CG7891 | + | -11199 | -8967 | DOWNSTREAM | CG7891-RA | "-"
 CG9656 in-situ | grn | - | 43629 | 9537 | DOWNSTREAM | CG9656-RA | "-"

*********************** Rank 1297 [Score 13.808167]   GBROWSE*******************

 CG14985 in-situ | CG14985 | + | -2049 | 1105 | INTRAGENIC | intron:CG14985-RA:3 | CG14985-RA | "-"
insitu highlight CG1132 in-situ | fd64A | + | 13573 | 15658 | UPSTREAM | CG1132-RA | "-"

*********************** Rank 1298 [Score 13.805298]   GBROWSE*******************

 CG4396 in-situ | fne | + | -10590 | -7387 | DOWNSTREAM | CG4396-RA | "-"
 CG4395 in-situ | CG4395 | - | 1349 | -1886 | INTRAGENIC | intron:CG4395-RA:2 | CG4395-RA | "-"


*********************** Rank 1299 [Score 13.803955]   GBROWSE*******************

 CG10601 in-situ | mirr | + | -21455 | -5060 | DOWNSTREAM | CG10601-RA | "-" | CG10601-RB | "-"
 CG10753 in-situ | snRNP69D | - | 19405 | 18156 | DOWNSTREAM | CG10753-RA | "-"

*********************** Rank 1300 [Score 13.803955]   GBROWSE*******************

 CG15696 in-situ | CG15696 | + | -2350 | -1811 | DOWNSTREAM | CG15696-RA | "-"
 CG15697 in-situ | CG15697 | + | 1088 | 1973 | UPSTREAM | CG15697-RA | "-" | CG15697-RB | "-"

*********************** Rank 1301 [Score 13.801025]   GBROWSE*******************

 CG14913 in-situ | CG14913 | + | -2819 | -1632 | DOWNSTREAM | CG14913-RA | "-"
 CG18666 in-situ | CG18666 | + | 14861 | 15244 | UPSTREAM | CG18666-RA | "-"


*********************** Rank 1302 [Score 13.800293]   GBROWSE*******************

 CG6380 in-situ | CG6380 | - | -53810 | -54847 | UPSTREAM | CG6380-RA | "-"
 CG31804 in-situ | CG31804 | + | 17478 | 18218 | UPSTREAM | CG31804-RA | "-"

*********************** Rank 1303 [Score 13.799683]   GBROWSE*******************

 CG14740 in-situ | CG14740 | - | -4665 | -6540 | UPSTREAM | CG14740-RA | "-"
 CG14741 in-situ | CG14741 | - | 9867 | 1974 | DOWNSTREAM | CG14741-RA | "-"

*********************** Rank 1304 [Score 13.797058]   GBROWSE*******************

 CG12617 in-situ | CG12617 | + | -8902 | -8256 | DOWNSTREAM | CG12617-RA | "-"
insitu CG10076 in-situ | spir | + | 7611 | 44787 | UPSTREAM | CG10076-RA | "-" | CG10076-RB | "-" | CG10076-RD | "-" | CG10076-RC | "-"

*********************** Rank 1305 [Score 13.795532]   GBROWSE*******************

 CG14346 in-situ | CG14346 | + | -16032 | -15087 | DOWNSTREAM | CG14346-RA | "-" | CG14346-RB | "-"
 CG5481 in-situ | lea | - | 36315 | -3246 | INTRAGENIC | intron:CG5481-RA:13 | CG5481-RA | "-"

*********************** Rank 1306 [Score 13.793213]   GBROWSE*******************

 CG6902 in-situ | CG6902 | - | -4429 | -7626 | UPSTREAM | CG6902-RA | "-"
 CG6694 in-situ | CG6694 | - | 11007 | 8729 | DOWNSTREAM | CG6694-RA | "-"

*********************** Rank 1307 [Score 13.793213]   GBROWSE*******************

 CG32822 in-situ | CG32822 | + | -16842 | -16180 | DOWNSTREAM | CG32822-RA | "-"
 CG14476 in-situ | BcDNA:GH04962 | - | 68240 | 63573 | DOWNSTREAM | CG14476-RB | "-" | CG14476-RC | "-" | CG14476-RA | "-" | CG14476-RE | "-" | CG14476-RD | "-"

*********************** Rank 1308 [Score 13.793213]   GBROWSE*******************

 CG17178 in-situ | ACXE | + | -8661 | -4525 | DOWNSTREAM | CG17178-RA | "-"
 CG16800 in-situ | CG16800 | + | 32550 | 33508 | UPSTREAM | CG16800-RA | "-"

*********************** Rank 1309 [Score 13.792847]   GBROWSE*******************

 CG16716 in-situ | CG16716 | + | -7060 | -4137 | DOWNSTREAM | CG16716-RB | "-" | CG16716-RA | "-"
insitu CG9218 in-situ | sm | - | 30622 | -71007 | INTRAGENIC | intron:CG9218-RA:3 | intron:CG9218-RC:3 | CG9218-RA | "-" | CG9218-RC | "-" | CG9218-RD | "-" | CG9218-RB | "-"

*********************** Rank 1310 [Score 13.790894]   GBROWSE*******************

 CG15414 in-situ | CG15414 | - | -6565 | -7527 | UPSTREAM | CG15414-RA | "-"
insitu CG3234 in-situ | tim | - | 18260 | 5495 | DOWNSTREAM | CG3234-RA | "-" | CG3234-RC | "-" | CG3234-RB | "-"

*********************** Rank 1311 [Score 13.790649]   GBROWSE*******************

insitu CG31632 in-situ | CG31632 | + | -19917 | -3229 | DOWNSTREAM | CG31632-RA | "-"
 CG10800 in-situ | Rca1 | + | 10090 | 11642 | UPSTREAM | CG10800-RA | "-"


*********************** Rank 1312 [Score 13.787231]   GBROWSE*******************

 CG31386 in-situ | CG31386 | - | -10102 | -35957 | UPSTREAM | CG31386-RA | "-"
 CG17216 in-situ | KP78b | - | 43081 | 40998 | DOWNSTREAM | CG17216-RA | "-"

*********************** Rank 1313 [Score 13.784790]   GBROWSE*******************

 CG10593 in-situ | Acer | + | -4520 | -1428 | DOWNSTREAM | CG10593-RA | "-"
 CG18042 in-situ | lmg | - | 2199 | 119 | DOWNSTREAM | CG18042-RA | "-"

*********************** Rank 1314 [Score 13.782471]   GBROWSE*******************

 CG10579 in-situ | Eip63E | + | -80486 | 12380 | INTRAGENIC | intron:CG10579-RD:6 | intron:CG10579-RE:7 | intron:CG10579-RA:7 | intron:CG10579-RB:6 | intron:CG10579-RC:6 | CG10579-RD | "-" | CG10579-RE | "-" | CG10579-RA | "-" | CG10579-RB | "-" | CG10579-RC | "-"
 CG10359 in-situ | CG10359 | + | 16023 | 19074 | UPSTREAM | CG10359-RA | "-"

*********************** Rank 1315 [Score 13.781128]   GBROWSE*******************

insitu CG3619 in-situ | Dl | - | -28798 | -52280 | UPSTREAM | CG3619-RA | "-" | CG3619-RB | "-"
 CG3581 in-situ | CG3581 | - | 19738 | 18746 | DOWNSTREAM | CG3581-RA | "-"

*********************** Rank 1316 [Score 13.774048]   GBROWSE*******************

 CG15925 in-situ | CG15925 | + | -14889 | -13361 | DOWNSTREAM | CG15925-RA | "-"
 CG15712 in-situ | CG15712 | - | 24665 | 23966 | DOWNSTREAM | CG15712-RA | "-"


*********************** Rank 1317 [Score 13.773438]   GBROWSE*******************

 CG18769 in-situ | CG18769 | + | -4182 | 39019 | INTRAGENIC | intron:CG18769-RA:2 | intron:CG18769-RC:2 | intron:CG18769-RB:1 | CG18769-RA | "-" | CG18769-RC | "-" | CG18769-RB | "-"
 CG32397 in-situ | CG32397 | - | 18142 | 8522 | DOWNSTREAM | CG32397-RA | "-"

*********************** Rank 1318 [Score 13.771851]   GBROWSE*******************

 CG7530 in-situ | CG7530 | - | -4751 | -11510 | UPSTREAM | CG7530-RA | "-" | CG7530-RB | "-" | CG7530-RC | "-"
 CG7425 in-situ | eff | - | 4951 | -3570 | INTRAGENIC | intron:CG7425-RA:3 | CG7425-RA | "-"

*********************** Rank 1319 [Score 13.767944]   GBROWSE*******************

 CG10862 in-situ | CG10862 | - | -12600 | -13906 | UPSTREAM | CG10862-RA | "-"
 CG10858 in-situ | CG10858 | - | 87830 | 81154 | DOWNSTREAM | CG10858-RA | "-"

*********************** Rank 1320 [Score 13.766296]   GBROWSE*******************

insitu CG11453 in-situ | CG11453 | + | -37738 | -35731 | DOWNSTREAM | CG11453-RA | "-"
insitu CG4608 in-situ | bnl | - | 15556 | -27188 | INTRAGENIC | intron:CG4608-RA:2 | intron:CG4608-RB:2 | CG4608-RA | "-" | CG4608-RB | "-"

*********************** Rank 1321 [Score 13.759155]   GBROWSE*******************

 CG1921 in-situ | sty | - | -8086 | -31884 | UPSTREAM | CG1921-RC | "-" | CG1921-RB | "-"
 CG10840 in-situ | cIF2 | - | 27581 | -4367 | INTRAGENIC | intron:CG10840-RB:7 | CG10840-RB | "-"

*********************** Rank 1322 [Score 13.758423]   GBROWSE*******************

 CG3590 in-situ | CG3590 | - | -6675 | -8989 | UPSTREAM | CG3590-RA | "-"
 CG5225 in-situ | CG5225 | + | 943 | 2901 | UPSTREAM | CG5225-RA | "-"

*********************** Rank 1323 [Score 13.757935]   GBROWSE*******************

insitu CG8646 in-situ | CG8646 | + | -4681 | -1289 | DOWNSTREAM | CG8646-RA | "-"
 CG8772 in-situ | nemy | - | 18952 | 9968 | DOWNSTREAM | CG8772-RD | "-" | CG8772-RA | "-" | CG8772-RE | "-" | CG8772-RC | "-" | CG8772-RB | "-"

*********************** Rank 1324 [Score 13.756348]   GBROWSE*******************

 CG9973 in-situ | CG9973 | - | -611 | -3055 | UPSTREAM | CG9973-RA | "-"
 CG9972 in-situ | CG9972 | - | 19843 | 11317 | DOWNSTREAM | CG9972-RA | "-"

*********************** Rank 1325 [Score 13.755737]   GBROWSE*******************

insitu CG3376 in-situ | CG3376 | + | -5453 | 1741 | INTRAGENIC | intron:CG3376-RB:3 | intron:CG3376-RA:3 | CG3376-RB | "-" | CG3376-RA | "-"
 CG13577 in-situ | CG13577 | - | 5476 | 4198 | DOWNSTREAM | CG13577-RA | "-"

*********************** Rank 1326 [Score 13.753906]   GBROWSE*******************

 CG15225 in-situ | CG15225 | + | -14089 | -13370 | DOWNSTREAM | CG15225-RA | "-"
 CG9985 in-situ | sktl | - | 25358 | 20355 | DOWNSTREAM | CG9985-RA | "-"

*********************** Rank 1327 [Score 13.750732]   GBROWSE*******************

 CG6227 in-situ | CG6227 | + | -7917 | -3015 | DOWNSTREAM | CG6227-RA | "-"
 CG9156 in-situ | Pp1-13C | - | 8025 | 6063 | DOWNSTREAM | CG9156-RA | "-"


*********************** Rank 1328 [Score 13.750244]   GBROWSE*******************

 CG3779 in-situ | numb | + | -45102 | -19322 | DOWNSTREAM | CG3779-RB | "-" | CG3779-RA | "-" | CG3779-RC | "-"
insitu CG4422 in-situ | Gdi | - | 12306 | 9297 | DOWNSTREAM | CG4422-RA | "-"

*********************** Rank 1329 [Score 13.745972]   GBROWSE*******************

 CG4090 in-situ | CG4090 | - | -54315 | -60743 | UPSTREAM | CG4090-RA | "-"
 CG31262 in-situ | CG31262 | - | 5169 | 3076 | DOWNSTREAM | CG31262-RA | "-"

*********************** Rank 1330 [Score 13.743408]   GBROWSE*******************

 CG8742 in-situ | Gyc76C | - | -1310 | -8667 | UPSTREAM | CG8742-RA | "-"
 CG14101 in-situ | CG14101 | + | 730 | 2028 | UPSTREAM | CG14101-RA | "-"

*********************** Rank 1331 [Score 13.739319]   GBROWSE*******************

insitu CG17765 in-situ | CG17765 | + | -31607 | -29874 | DOWNSTREAM | CG17765-RA | "-"
 CG12052 in-situ | lola | - | 31522 | -28827 | INTRAGENIC | intron:CG12052-RI:5 | intron:CG12052-RC:5 | intron:CG12052-RH:5 | intron:CG12052-RG:5 | intron:CG12052-RT:5 | intron:CG12052-RU:5 | intron:CG12052-RN:5 | intron:CG12052-RP:5 | intron:CG12052-RR:5 | intron:CG12052-RJ:5 | intron:CG12052-RO:5 | CG12052-RI | "-" | CG12052-RE | "-" | CG12052-RB | "-" | CG12052-RA | "-" | CG12052-RC | "-" | CG12052-RH | "-" | CG12052-RG | "-" | CG12052-RF | "-" | CG12052-RT | "-" | CG12052-RU | "-" | CG12052-RL | "-" | CG12052-RK | "-" | CG12052-RN | "-" | CG12052-RP | "-" | CG12052-RQ | "-" | CG12052-RR | "-" | CG12052-RS | "-" | CG12052-RJ | "-" | CG12052-RO | "-" | CG12052-RM | "-" | CG12052-RD | "-"

*********************** Rank 1332 [Score 13.736206]   GBROWSE*******************

 CG6352 in-situ | OdsH | + | -15591 | 7347 | INTRAGENIC | intron:CG6352-RA:1 | CG6352-RA | "-"
 CG12986 in-situ | CG12986 | - | 12974 | 12597 | DOWNSTREAM | CG12986-RA | "-"

*********************** Rank 1333 [Score 13.728638]   GBROWSE*******************

 CG2715 in-situ | Syx4 | - | -43562 | -48541 | UPSTREAM | CG2715-RA | "-"
 CG32795 in-situ | EG:BACN33B1.2 | - | 2314 | -4722 | INTRAGENIC | intron:CG32795-RA:3 | intron:CG32795-RB:2 | CG32795-RA | "-" | CG32795-RB | "-"


*********************** Rank 1334 [Score 13.727051]   GBROWSE*******************

 CG31146 in-situ | CG31146 | + | -15660 | 20409 | INTRAGENIC | intron:CG31146-RD:8 | CG31146-RD | "-"
 CG2616 in-situ | CG2616 | + | 48504 | 50505 | UPSTREAM | CG2616-RA | "-"

*********************** Rank 1335 [Score 13.725952]   GBROWSE*******************

 CG31340 in-situ | CG31340 | - | -30975 | -32832 | UPSTREAM | CG31340-RA | "-"
 CG17077 in-situ | pnt | - | 18091 | -36967 | INTRAGENIC | intron:CG17077-RB:3 | CG17077-RB | "-" | CG17077-RD | "-" | CG17077-RC | "-"

*********************** Rank 1336 [Score 13.720947]   GBROWSE*******************

 CG3473 in-situ | BG:DS01486.1 | - | -6322 | -6976 | UPSTREAM | CG3473-RA | "-"
insitu CG32954 in-situ | CG32954 | + | 45507 | 48854 | UPSTREAM | CG32954-RA | "-" | CG32954-RB | "-" | CG32954-RC | "-" | CG32954-RG | "-" | CG32954-RH | "-" | CG32954-RF | "-" | CG32954-RD | "-" | CG32954-RE | "-"

*********************** Rank 1337 [Score 13.720459]   GBROWSE*******************

 CG14910 in-situ | CG14910 | + | -17205 | -16702 | DOWNSTREAM | CG14910-RA | "-"
 CG14911 in-situ | CG14911 | + | 5182 | 6185 | UPSTREAM | CG14911-RA | "-"

*********************** Rank 1338 [Score 13.719727]   GBROWSE*******************

 CG4988 in-situ | CG4988 | + | -35873 | -34657 | DOWNSTREAM | CG4988-RA | "-"
 CG12602 in-situ | CG12602 | + | 8223 | 13432 | UPSTREAM | CG12602-RA | "-"

*********************** Rank 1339 [Score 13.717957]   GBROWSE*******************

 CG32725 in-situ | CG32725 | - | -11062 | -11789 | UPSTREAM | CG32725-RA | "-"
 CG1958 in-situ | CG1958 | + | 17681 | 18658 | UPSTREAM | CG1958-RA | "-"

*********************** Rank 1340 [Score 13.713257]   GBROWSE*******************

 CG17453 in-situ | Cyp317a1 | + | -6829 | -5273 | DOWNSTREAM | CG17453-RA | "-"
 CG10249 in-situ | BcDNA:GH03482 | - | 5893 | -4849 | INTRAGENIC | intron:CG10249-RC:3 | intron:CG10249-RA:1 | CG10249-RC | "-" | CG10249-RA | "-" | CG10249-RB | "-"

*********************** Rank 1341 [Score 13.712524]   GBROWSE*******************

 CG9733 in-situ | CG9733 | - | -909 | -3636 | UPSTREAM | CG9733-RA | "-"
 CG9682 in-situ | CG9682 | + | 3310 | 5223 | UPSTREAM | CG9682-RA | "-"


*********************** Rank 1342 [Score 13.711060]   GBROWSE*******************

 CG15544 in-situ | CG15544 | + | -18012 | -3828 | DOWNSTREAM | CG15544-RA | "-"
insitu highlight CG1378 in-situ | tll | + | 6506 | 8511 | UPSTREAM | CG1378-RA | "-"

note: overlaps known module tll_rescue by 194 bases (module coords: 26661256-26671461)

*********************** Rank 1343 [Score 13.710449]   GBROWSE*******************

 CG31085 in-situ | CG31085 | + | -19408 | -6324 | DOWNSTREAM | CG31085-RB | "-" | CG31085-RA | "-"
 CG14239 in-situ | CG14239 | - | 34475 | 33596 | DOWNSTREAM | CG14239-RA | "-"

*********************** Rank 1344 [Score 13.704468]   GBROWSE*******************

 CG3611 in-situ | CG3611 | + | -1982 | -1218 | DOWNSTREAM | CG3611-RA | "-"
 CG3629 in-situ | Dll | + | 14050 | 34383 | UPSTREAM | CG3629-RB | "-" | CG3629-RA | "-"

*********************** Rank 1345 [Score 13.703613]   GBROWSE*******************

insitu CG9986 in-situ | CG9986 | + | -10175 | -7938 | DOWNSTREAM | CG9986-RA | "-"
 CG10011 in-situ | CG10011 | - | 18751 | -8381 | INTRAGENIC | intron:CG10011-RA:1 | CG10011-RA | "-"


*********************** Rank 1346 [Score 13.702148]   GBROWSE*******************

 CG15061 in-situ | CG15061 | + | -11712 | -10713 | DOWNSTREAM | CG15061-RA | "-"
 CG5993 in-situ | os | - | 3534 | 1053 | DOWNSTREAM | CG5993-RA | "-"

*********************** Rank 1347 [Score 13.701294]   GBROWSE*******************

 CG31419 in-situ | CG31419 | + | -76203 | -75730 | DOWNSTREAM | CG31419-RA | "-"
insitu CG14334 in-situ | beat-IIa | - | 5004 | -41114 | INTRAGENIC | intron:CG14334-RA:1 | CG14334-RA | "-"

*********************** Rank 1348 [Score 13.701050]   GBROWSE*******************

 CG30128 in-situ | Obp56c | - | -1283 | -2157 | UPSTREAM | CG30128-RA | "-"
 CG11218 in-situ | Obp56d | - | 1465 | 800 | DOWNSTREAM | CG11218-RA | "-"

*********************** Rank 1349 [Score 13.699097]   GBROWSE*******************

 CG6793 in-situ | CG6793 | + | -15293 | -13538 | DOWNSTREAM | CG6793-RA | "-"
insitu CG32096 in-situ | rols | - | 7156 | -49488 | INTRAGENIC | intron:CG32096-RB:2 | CG32096-RB | "-" | CG32096-RD | "-" | CG32096-RE | "-" | CG32096-RA | "-" | CG32096-RC | "-"


*********************** Rank 1350 [Score 13.693604]   GBROWSE*******************

 CG11994 in-situ | Ada | + | -68684 | -67611 | DOWNSTREAM | CG11994-RA | "-"
 CG11997 in-situ | CG11997 | + | 43700 | 45010 | UPSTREAM | CG11997-RA | "-"

*********************** Rank 1351 [Score 13.693298]   GBROWSE*******************

 CG1867 in-situ | Or98b | + | -50720 | -49342 | DOWNSTREAM | CG1867-RA | "-"
 CG14064 in-situ | beat-VI | + | 15242 | 70247 | UPSTREAM | CG14064-RA | "-"

*********************** Rank 1352 [Score 13.692200]   GBROWSE*******************

 CG7527 in-situ | CadN2 | - | -38163 | -64162 | UPSTREAM | CG7527-RA | "-"
 CG5674 in-situ | CG5674 | + | 112571 | 124103 | UPSTREAM | CG5674-RA | "-" | CG5674-RB | "-" | CG5674-RC | "-"

*********************** Rank 1353 [Score 13.691895]   GBROWSE*******************

 CG12732 in-situ | CG12732 | + | -45408 | -45028 | DOWNSTREAM | CG12732-RA | "-"
insitu highlight CG3252 in-situ | CG3252 | - | 7109 | 1778 | DOWNSTREAM | CG3252-RA | "-"

*********************** Rank 1354 [Score 13.689941]   GBROWSE*******************

 CG7370 in-situ | CG7370 | + | -19557 | -18046 | DOWNSTREAM | CG7370-RA | "-"
 CG7383 in-situ | eg | + | 19331 | 28609 | UPSTREAM | CG7383-RB | "-" | CG7383-RA | "-"

*********************** Rank 1355 [Score 13.689697]   GBROWSE*******************

 CG31605 in-situ | CG31605 | + | -20847 | 6276 | INTRAGENIC | intron:CG31605-RB:2 | intron:CG31605-RA:2 | intron:CG31605-RD:2 | intron:CG31605-RC:2 | intron:CG31605-RE:2 | intron:CG31605-RF:3 | intron:CG31605-RH:2 | intron:CG31605-RI:2 | intron:CG31605-RG:1 | CG31605-RB | "-" | CG31605-RA | "-" | CG31605-RD | "-" | CG31605-RC | "-" | CG31605-RE | "-" | CG31605-RF | "-" | CG31605-RH | "-" | CG31605-RI | "-" | CG31605-RG | "-"
 CG31756 in-situ | CG31756 | - | 19732 | 12694 | DOWNSTREAM | CG31756-RA | "-"

*********************** Rank 1356 [Score 13.686279]   GBROWSE*******************

 CG31226 in-situ | CG31226 | - | -10607 | -11108 | UPSTREAM | CG31226-RA | "-" | CG31226-RB | "-"
 CG14298 in-situ | CG14298 | + | 13374 | 14511 | UPSTREAM | CG14298-RA | "-"

*********************** Rank 1357 [Score 13.683350]   GBROWSE*******************

 CG1960 in-situ | mu2 | - | -1958 | -7287 | UPSTREAM | CG1960-RA | "-" | CG1960-RB | "-"
 CG11814 in-situ | CG11814 | - | 8762 | 558 | DOWNSTREAM | CG11814-RA | "-"

*********************** Rank 1358 [Score 13.681641]   GBROWSE*******************

 CG11769 in-situ | CG11769 | + | -12910 | -12164 | DOWNSTREAM | CG11769-RA | "-"
 CG31448 in-situ | CG31448 | + | 17258 | 17950 | UPSTREAM | CG31448-RA | "-"

*********************** Rank 1359 [Score 13.677124]   GBROWSE*******************

 CG12899 in-situ | CG12899 | - | -4703 | -4987 | UPSTREAM | CG12899-RA | "-"
 CG12898 in-situ | CG12898 | - | 14328 | 13858 | DOWNSTREAM | CG12898-RA | "-"

*********************** Rank 1360 [Score 13.676575]   GBROWSE*******************

 CG31172 in-situ | CG31172 | - | -39570 | -40438 | UPSTREAM | CG31172-RA | "-"
 CG13627 in-situ | CG13627 | + | 2013 | 6782 | UPSTREAM | CG13627-RA | "-" | CG13627-RB | "-"

*********************** Rank 1361 [Score 13.676147]   GBROWSE*******************

 CG14935 in-situ | CG14935 | + | -6467 | -3860 | DOWNSTREAM | CG14935-RA | "-" | CG14935-RB | "-"
 CG14943 in-situ | CG14943 | - | 2921 | 2397 | DOWNSTREAM | CG14943-RA | "-"

*********************** Rank 1362 [Score 13.675049]   GBROWSE*******************

 CG15685 in-situ | CG15685 | + | -8307 | -4559 | DOWNSTREAM | CG15685-RA | "-"
 CG5097 in-situ | CG5097 | + | 11885 | 12075 | UPSTREAM | CG5097-RA | "-"

*********************** Rank 1363 [Score 13.673706]   GBROWSE*******************

insitu highlight CG18455 in-situ | Optix | + | -21970 | -11067 | DOWNSTREAM | CG18455-RA | "-" | CG18455-RB | "-"
 CG12769 in-situ | CG12769 | - | 11948 | 223 | DOWNSTREAM | CG12769-RB | "-" | CG12769-RA | "-"

*********************** Rank 1364 [Score 13.671509]   GBROWSE*******************

insitu CG2083 in-situ | CG2083 | - | -6791 | -18799 | UPSTREAM | CG2083-RA | "-"
 CG14952 in-situ | CG14952 | - | 40974 | 40591 | DOWNSTREAM | CG14952-RA | "-"

*********************** Rank 1365 [Score 13.670410]   GBROWSE*******************

 CG31263 in-situ | CG31263 | - | -7560 | -8042 | UPSTREAM | CG31263-RA | "-"
 CG5184 in-situ | mRpS11 | + | 2918 | 3996 | UPSTREAM | CG5184-RA | "-" | CG5184-RB | "-"

*********************** Rank 1366 [Score 13.670288]   GBROWSE*******************

 CG30473 in-situ | Obp51a | - | -75685 | -76105 | UPSTREAM | CG30473-RA | "-"
insitu highlight CG11798 in-situ | CG11798 | + | 28391 | 43632 | UPSTREAM | CG11798-RA | "-"

*********************** Rank 1367 [Score 13.669373]   GBROWSE*******************

 CG16898 in-situ | CG16898 | - | -77651 | -79019 | UPSTREAM | CG16898-RA | "-"
 CG8896 in-situ | 18w | + | 28873 | 34294 | UPSTREAM | CG8896-RA | "-"


*********************** Rank 1368 [Score 13.667969]   GBROWSE*******************

 CG3245 in-situ | PpN58A | - | -24966 | -28570 | UPSTREAM | CG3245-RB | "-" | CG3245-RA | "-"
 CG4054 in-situ | CG4054 | + | 21671 | 22812 | UPSTREAM | CG4054-RA | "-"

*********************** Rank 1369 [Score 13.662964]   GBROWSE*******************

insitu CG8782 in-situ | CG8782 | - | -5544 | -8117 | UPSTREAM | CG8782-RA | "-"
insitu CG8780 in-situ | CG8780 | - | 8270 | -4591 | INTRAGENIC | intron:CG8780-RA:3 | CG8780-RA | "-"

*********************** Rank 1370 [Score 13.661255]   GBROWSE*******************

 CG9166 in-situ | 312 | + | -9482 | -7768 | DOWNSTREAM | CG9166-RA | "-"
 CG1214 in-situ | ru | + | 9999 | 26814 | UPSTREAM | CG1214-RA | "-"

*********************** Rank 1371 [Score 13.659119]   GBROWSE*******************

 CG17127 in-situ | CG17127 | + | -12306 | -12010 | DOWNSTREAM | CG17127-RA | "-"
 CG31870 in-situ | CG31870 | + | 6951 | 7485 | UPSTREAM | CG31870-RA | "-" | CG31870-RC | "-"

*********************** Rank 1372 [Score 13.658936]   GBROWSE*******************

 CG1472 in-situ | CG1472 | - | -1414 | -6868 | UPSTREAM | CG1472-RA | "-"
 CG12923 in-situ | CG12923 | + | 3881 | 5143 | UPSTREAM | CG12923-RA | "-"

*********************** Rank 1373 [Score 13.658569]   GBROWSE*******************

 CG9380 in-situ | CG9380 | - | -16793 | -22001 | UPSTREAM | CG9380-RA | "-" | CG9380-RB | "-"
insitu highlight CG3340 in-situ | Kr | + | 20985 | 23904 | UPSTREAM | CG3340-RA | "-"

*********************** Rank 1374 [Score 13.656982]   GBROWSE*******************

 CG32414 in-situ | CG32414 | + | -6367 | -4944 | DOWNSTREAM | CG32414-RA | "-"
insitu CG10578 in-situ | DnaJ-1 | - | 5238 | 3030 | DOWNSTREAM | CG10578-RA | "-" | CG10578-RB | "-"

*********************** Rank 1375 [Score 13.656860]   GBROWSE*******************

 CG4704 in-situ | CG4704 | - | -43560 | -44680 | UPSTREAM | CG4704-RA | "-"
 CG6669 in-situ | klg | + | 10430 | 72862 | UPSTREAM | CG6669-RA | "-"

*********************** Rank 1376 [Score 13.655640]   GBROWSE*******************

 CG14064 in-situ | beat-VI | + | -22608 | 32397 | INTRAGENIC | intron:CG14064-RA:1 | CG14064-RA | "-"
 CG1894 in-situ | CG1894 | + | 43840 | 45186 | UPSTREAM | CG1894-RA | "-"

*********************** Rank 1377 [Score 13.655334]   GBROWSE*******************

 CG14247 in-situ | CG14247 | + | -21571 | -21044 | DOWNSTREAM | CG14247-RA | "-"
 CG5490 in-situ | Tl | + | 39630 | 82990 | UPSTREAM | CG5490-RB | "-" | CG5490-RA | "-"

*********************** Rank 1378 [Score 13.655212]   GBROWSE*******************

 CG11251 in-situ | CG11251 | - | -8175 | -9191 | UPSTREAM | CG11251-RA | "-"
 CG32118 in-situ | CG32118 | + | 248 | 625 | UPSTREAM | CG32118-RA | "-"

*********************** Rank 1379 [Score 13.654907]   GBROWSE*******************

 CG8517 in-situ | CG8517 | + | -8404 | -7607 | DOWNSTREAM | CG8517-RA | "-"
 CG12501 in-situ | Or56a | - | 48746 | 46974 | DOWNSTREAM | CG12501-RA | "-"

*********************** Rank 1380 [Score 13.653687]   GBROWSE*******************

 CG11828 in-situ | CG11828 | + | -12442 | -10579 | DOWNSTREAM | CG11828-RA | "-"
 CG14521 in-situ | CG14521 | - | 34792 | -10567 | INTRAGENIC | intron:CG14521-RA:1 | CG14521-RA | "-"


*********************** Rank 1381 [Score 13.653259]   GBROWSE*******************

 CG7050 in-situ | Nrx-1 | + | -9287 | 4203 | INTRAGENIC | intron:CG7050-RA:11 | CG7050-RA | "-"
insitu CG7048 in-situ | CG7048 | + | 7433 | 8267 | UPSTREAM | CG7048-RA | "-"

*********************** Rank 1382 [Score 13.651978]   GBROWSE*******************

 CG14680 in-situ | Cyp12e1 | + | -9810 | -7947 | DOWNSTREAM | CG14680-RA | "-" | CG14680-RB | "-"
 CG31395 in-situ | CG31395 | + | 28506 | 29201 | UPSTREAM | CG31395-RA | "-"

*********************** Rank 1383 [Score 13.650391]   GBROWSE*******************

 CG4319 in-situ | rpr | - | -27668 | -28518 | UPSTREAM | CG4319-RA | "-"
insitu CG13701 in-situ | skl | - | 13866 | 12485 | DOWNSTREAM | CG13701-RA | "-"

*********************** Rank 1384 [Score 13.644165]   GBROWSE*******************

 CG14619 in-situ | CG14619 | + | -10632 | 2056 | INTRAGENIC | intron:CG14619-RE:5 | intron:CG14619-RD:5 | intron:CG14619-RA:5 | intron:CG14619-RC:2 | CG14619-RE | "-" | CG14619-RD | "-" | CG14619-RA | "-" | CG14619-RC | "-" | CG14619-RB | "-"
 CG14613 in-situ | CG14613 | - | 5540 | 3823 | DOWNSTREAM | CG14613-RA | "-"

*********************** Rank 1385 [Score 13.643311]   GBROWSE*******************

 CG31172 in-situ | CG31172 | - | -4420 | -5288 | UPSTREAM | CG31172-RA | "-"
 CG13627 in-situ | CG13627 | + | 37163 | 41932 | UPSTREAM | CG13627-RA | "-" | CG13627-RB | "-"

*********************** Rank 1386 [Score 13.643188]   GBROWSE*******************

insitu CG12911 in-situ | CG12911 | + | -5878 | -1291 | DOWNSTREAM | CG12911-RA | "-"
 CG12910 in-situ | CG12910 | + | 4302 | 6640 | UPSTREAM | CG12910-RA | "-"

*********************** Rank 1387 [Score 13.638489]   GBROWSE*******************

 CG30125 in-situ | CG30125 | - | -18956 | -20863 | UPSTREAM | CG30125-RA | "-"
insitu CG15117 in-situ | CG15117 | - | 14111 | 7100 | DOWNSTREAM | CG15117-RA | "-" | CG15117-RB | "-"

*********************** Rank 1388 [Score 13.637146]   GBROWSE*******************

 CG3407 in-situ | CG3407 | - | -5524 | -8132 | UPSTREAM | CG3407-RA | "-"
insitu highlight CG16738 in-situ | slp1 | + | 6749 | 8206 | UPSTREAM | CG16738-RA | "-"

*********************** Rank 1389 [Score 13.636963]   GBROWSE*******************

 CG3523 in-situ | BcDNA:GH07626 | + | -48366 | -36797 | DOWNSTREAM | CG3523-RA | "-"
 CG8822 in-situ | PpD6 | + | 4691 | 5749 | UPSTREAM | CG8822-RA | "-"

*********************** Rank 1390 [Score 13.633240]   GBROWSE*******************

 CG13109 in-situ | tai | + | -11717 | 67871 | INTRAGENIC | intron:CG13109-RA:1 | CG13109-RA | "-"
 CG17009 in-situ | CG17009 | - | 72493 | 71777 | DOWNSTREAM | CG17009-RA | "-"

*********************** Rank 1391 [Score 13.632080]   GBROWSE*******************

 CG15380 in-situ | CG15380 | - | -11014 | -11954 | UPSTREAM | CG15380-RA | "-"
 CG12674 in-situ | CG12674 | + | 1642 | 2735 | UPSTREAM | CG12674-RA | "-"

*********************** Rank 1392 [Score 13.630005]   GBROWSE*******************

 CG10481 in-situ | CG10481 | - | -9344 | -11626 | UPSTREAM | CG10481-RA | "-"
insitu CG13960 in-situ | CG13960 | + | 6867 | 8563 | UPSTREAM | CG13960-RA | "-"

*********************** Rank 1393 [Score 13.627563]   GBROWSE*******************

insitu highlight CG32423 in-situ | CG32423 | - | -4698 | -121966 | UPSTREAM | CG32423-RA | "-" | CG32423-RD | "-" | CG32423-RB | "-" | CG32423-RC | "-"
 CG10645 in-situ | lama | - | 73281 | 60973 | DOWNSTREAM | CG10645-RC | "-" | CG10645-RB | "-" | CG10645-RA | "-"


*********************** Rank 1394 [Score 13.626648]   GBROWSE*******************

 CG31827 in-situ | CG31827 | + | -36630 | -35646 | DOWNSTREAM | CG31827-RA | "-" | CG31827-RB | "-"
insitu highlight CG3938 in-situ | CycE | - | 6289 | -14296 | INTRAGENIC | intron:CG3938-RE:1 | intron:CG3938-RA:1 | intron:CG3938-RC:1 | intron:CG3938-RD:2 | CG3938-RE | "-" | CG3938-RA | "-" | CG3938-RC | "-" | CG3938-RD | "-" | CG3938-RB | "-"

*********************** Rank 1395 [Score 13.623962]   GBROWSE*******************

 CG4563 in-situ | CG4563 | - | -10595 | -12550 | UPSTREAM | CG4563-RA | "-"
 CG3492 in-situ | CG3492 | + | 9080 | 10601 | UPSTREAM | CG3492-RA | "-"


*********************** Rank 1396 [Score 13.618164]   GBROWSE*******************

 CG17843 in-situ | CG17843 | + | -8665 | -6866 | DOWNSTREAM | CG17843-RA | "-"
 CG31171 in-situ | CG31171 | - | 10406 | 9751 | DOWNSTREAM | CG31171-RA | "-"

*********************** Rank 1397 [Score 13.616333]   GBROWSE*******************

 CG5999 in-situ | CG5999 | - | -6937 | -8531 | UPSTREAM | CG5999-RA | "-"
 CG14390 in-situ | beat-Vc | - | 4142 | -3944 | INTRAGENIC | intron:CG14390-RA:2 | CG14390-RA | "-"

*********************** Rank 1398 [Score 13.613403]   GBROWSE*******************

insitu highlight CG6246 in-situ | nub | + | -25882 | -16096 | DOWNSTREAM | CG6246-RA | "-"
insitu highlight CG12287 in-situ | pdm2 | + | 13599 | 42083 | UPSTREAM | CG12287-RB | "-" | CG12287-RA | "-"

*********************** Rank 1399 [Score 13.601074]   GBROWSE*******************

 CG30089 in-situ | CG30089 | + | -36327 | -1162 | DOWNSTREAM | CG30089-RA | "-"
 CG33153 in-situ | CG33153 | - | 27710 | 26441 | DOWNSTREAM | CG33153-RA | "-"

*********************** Rank 1400 [Score 13.600098]   GBROWSE*******************

insitu CG15509 in-situ | kay | + | -3283 | 17854 | INTRAGENIC | intron:CG15509-RA:1 | CG15509-RA | "-" | CG15509-RB | "-"
 CG7615 in-situ | CG7615 | - | 4190 | 3150 | DOWNSTREAM | CG7615-RA | "-"


*********************** Rank 1401 [Score 13.596802]   GBROWSE*******************

 CG6559 in-situ | CG6559 | - | -17108 | -33346 | UPSTREAM | CG6559-RA | "-"
 CG12362 in-situ | CG12362 | + | 82995 | 84956 | UPSTREAM | CG12362-RB | "-" | CG12362-RA | "-"

*********************** Rank 1402 [Score 13.596436]   GBROWSE*******************

 CG6824 in-situ | ovo | + | -21489 | -82 | DOWNSTREAM | CG6824-RB | "-" | CG6824-RC | "-" | CG6824-RA | "-"
 CG32767 in-situ | CG32767 | - | 11287 | 4848 | DOWNSTREAM | CG32767-RA | "-"

*********************** Rank 1403 [Score 13.591919]   GBROWSE*******************

 CG5065 in-situ | CG5065 | + | -6055 | 9637 | INTRAGENIC | intron:CG5065-RA:1 | CG5065-RA | "-"
 CG8250 in-situ | Alk | - | 24049 | 12360 | DOWNSTREAM | CG8250-RA | "-"


*********************** Rank 1404 [Score 13.590454]   GBROWSE*******************

 CG12027 in-situ | CG12027 | - | -16795 | -17401 | UPSTREAM | CG12027-RA | "-"
 CG4597 in-situ | CG4597 | + | 82133 | 82699 | UPSTREAM | CG4597-RA | "-"

*********************** Rank 1405 [Score 13.590454]   GBROWSE*******************

 CG11401 in-situ | Trxr-2 | + | -12146 | -10350 | DOWNSTREAM | CG11401-RA | "-"
 CG14459 in-situ | CG14459 | - | 21281 | 20542 | DOWNSTREAM | CG14459-RA | "-"

*********************** Rank 1406 [Score 13.584351]   GBROWSE*******************

 CG14597 in-situ | CG14597 | - | -4218 | -4859 | UPSTREAM | CG14597-RA | "-"
 CG31146 in-situ | CG31146 | + | 50290 | 86359 | UPSTREAM | CG31146-RD | "-"

*********************** Rank 1407 [Score 13.583374]   GBROWSE*******************

 CG5559 in-situ | CG5559 | + | -23303 | -12879 | DOWNSTREAM | CG5559-RA | "-"
 CG15147 in-situ | CG15147 | - | 10178 | 9580 | DOWNSTREAM | CG15147-RA | "-"


*********************** Rank 1408 [Score 13.582397]   GBROWSE*******************

insitu CG31235 in-situ | CG31235 | - | -10673 | -22606 | UPSTREAM | CG31235-RA | "-"
 CG15803 in-situ | CG15803 | - | 15915 | 13222 | DOWNSTREAM | CG15803-RA | "-"

*********************** Rank 1409 [Score 13.579224]   GBROWSE*******************

insitu CG11387 in-situ | ct | + | -30525 | 36350 | INTRAGENIC | intron:CG11387-RA:1 | intron:CG11387-RB:2 | CG11387-RA | "-" | CG11387-RB | "-"
 CG12690 in-situ | CHES-1-like | - | 57021 | 45214 | DOWNSTREAM | CG12690-RA | "-"

*********************** Rank 1410 [Score 13.578491]   GBROWSE*******************

 CG11405 in-situ | A3-3 | - | -8657 | -17573 | UPSTREAM | CG11405-RA | "-"
 CG32812 in-situ | EG:114D9.1 | + | 27733 | 28410 | UPSTREAM | CG32812-RA | "-"

*********************** Rank 1411 [Score 13.577881]   GBROWSE*******************

 CG4983 in-situ | CG4983 | + | -20681 | -19282 | DOWNSTREAM | CG4983-RA | "-"
 CG4988 in-situ | CG4988 | + | 21727 | 22943 | UPSTREAM | CG4988-RA | "-"

*********************** Rank 1412 [Score 13.577820]   GBROWSE*******************

insitu CG8440 in-situ | Lis1 | + | -2021 | 4282 | INTRAGENIC | intron:CG8440-RA:1 | intron:CG8440-RC:1 | intron:CG8440-RD:1 | intron:CG8440-RE:1 | intron:CG8440-RB:1 | CG8440-RA | "-" | CG8440-RC | "-" | CG8440-RD | "-" | CG8440-RE | "-" | CG8440-RB | "-"
 CG8441 in-situ | CG8441 | - | 5176 | 4289 | DOWNSTREAM | CG8441-RA | "-"

*********************** Rank 1413 [Score 13.574463]   GBROWSE*******************

insitu highlight CG8254 in-situ | exex | + | -14229 | -10853 | DOWNSTREAM | CG8254-RA | "-"
 CG8194 in-situ | RNaseX25 | + | 8174 | 10030 | UPSTREAM | CG8194-RA | "-"

*********************** Rank 1414 [Score 13.570496]   GBROWSE*******************

 CG31438 in-situ | CG31438 | + | -8937 | -8204 | DOWNSTREAM | CG31438-RA | "-"
 CG6570 in-situ | lbl | - | 16059 | -8087 | INTRAGENIC | intron:CG6570-RA:1 | CG6570-RA | "-"

*********************** Rank 1415 [Score 13.570190]   GBROWSE*******************

 CG13106 in-situ | Or30a | + | -7755 | -6284 | DOWNSTREAM | CG13106-RA | "-"
insitu highlight CG32982 in-situ | CG32982 | + | 6182 | 44158 | UPSTREAM | CG32982-RA | "-" | CG32982-RB | "-"

*********************** Rank 1416 [Score 13.568848]   GBROWSE*******************

insitu CG5467 in-situ | CG5467 | + | -15891 | -9501 | DOWNSTREAM | CG5467-RA | "-"
 CG5468 in-situ | CG5468 | + | 5560 | 6668 | UPSTREAM | CG5468-RA | "-"

*********************** Rank 1417 [Score 13.567566]   GBROWSE*******************

 CG6154 in-situ | CG6154 | + | -32801 | -25275 | DOWNSTREAM | CG6154-RA | "-" | CG6154-RB | "-"
 CG14559 in-situ | CG14559 | + | 22895 | 36184 | UPSTREAM | CG14559-RA | "-"


*********************** Rank 1418 [Score 13.565125]   GBROWSE*******************

 CG6888 in-situ | CG6888 | + | -18927 | -18179 | DOWNSTREAM | CG6888-RA | "-"
 CG6890 in-situ | Tollo | + | 8870 | 16061 | UPSTREAM | CG6890-RA | "-"

*********************** Rank 1419 [Score 13.564209]   GBROWSE*******************

 CG1789 in-situ | CG1789 | + | -56094 | -55150 | DOWNSTREAM | CG1789-RA | "-"
 CG11354 in-situ | Lim1 | - | 29124 | -19416 | INTRAGENIC | intron:CG11354-RA:3 | CG11354-RA | "-"

*********************** Rank 1420 [Score 13.563477]   GBROWSE*******************

 CG14027 in-situ | TotM | + | -1372 | -763 | DOWNSTREAM | CG14027-RA | "-"
 CG14021 in-situ | CG14021 | - | 6824 | -761 | INTRAGENIC | intron:CG14021-RB:4 | intron:CG14021-RA:4 | intron:CG14021-RC:3 | CG14021-RB | "-" | CG14021-RA | "-" | CG14021-RC | "-"

*********************** Rank 1421 [Score 13.560669]   GBROWSE*******************

insitu highlight CG2939 in-situ | slp2 | + | -20839 | -18480 | DOWNSTREAM | CG2939-RA | "-"
 CG3964 in-situ | CG3964 | + | 5000 | 9826 | UPSTREAM | CG3964-RB | "-" | CG3964-RA | "-"

*********************** Rank 1422 [Score 13.552856]   GBROWSE*******************

insitu CG12345 in-situ | Cha | + | -19488 | 7335 | INTRAGENIC | intron:CG12345-RA:2 | CG12345-RA | "-"
insitu CG7714 in-situ | CG7714 | - | 1423 | 383 | DOWNSTREAM | CG7714-RA | "-"

*********************** Rank 1423 [Score 13.549927]   GBROWSE*******************

insitu highlight CG4889 in-situ | wg | + | -17908 | -8814 | DOWNSTREAM | CG4889-RA | "-" | CG4889-RB | "-"
 CG4969 in-situ | Wnt6 | + | 25829 | 27211 | UPSTREAM | CG4969-RA | "-"

*********************** Rank 1424 [Score 13.548645]   GBROWSE*******************

insitu CG31243 in-situ | cpo | + | -77790 | 6131 | INTRAGENIC | intron:CG31243-RA:7 | intron:CG31243-RE:7 | intron:CG31243-RF:7 | intron:CG31243-RB:7 | CG31243-RA | "-" | CG31243-RE | "-" | CG31243-RF | "-" | CG31243-RB | "-"
 CG7780 in-situ | DNaseII | + | 9714 | 11498 | UPSTREAM | CG7780-RA | "-"


*********************** Rank 1425 [Score 13.547974]   GBROWSE*******************

 CG12063 in-situ | CG12063 | + | -43389 | -35264 | DOWNSTREAM | CG12063-RA | "-"
insitu CG1499 in-situ | CG1499 | + | 770 | 23905 | UPSTREAM | CG1499-RA | "-" | CG1499-RB | "-"

*********************** Rank 1426 [Score 13.546631]   GBROWSE*******************

insitu highlight CG4889 in-situ | wg | + | -22408 | -13314 | DOWNSTREAM | CG4889-RA | "-" | CG4889-RB | "-"
 CG4969 in-situ | Wnt6 | + | 21329 | 22711 | UPSTREAM | CG4969-RA | "-"

*********************** Rank 1427 [Score 13.545288]   GBROWSE*******************

 CG13700 in-situ | CG13700 | - | -711 | -3084 | UPSTREAM | CG13700-RA | "-"
insitu highlight CG4345 in-situ | grim | - | 2443 | 748 | DOWNSTREAM | CG4345-RA | "-"

*********************** Rank 1428 [Score 13.544800]   GBROWSE*******************

insitu highlight CG4889 in-situ | wg | + | -3558 | 5536 | INTRAGENIC | intron:CG4889-RA:2 | intron:CG4889-RB:1 | CG4889-RA | "-" | CG4889-RB | "-"
 CG4969 in-situ | Wnt6 | + | 40179 | 41561 | UPSTREAM | CG4969-RA | "-"

*********************** Rank 1429 [Score 13.544556]   GBROWSE*******************

 CG3694 in-situ | Ggamma30A | + | -28504 | 6735 | INTRAGENIC | intron:CG3694-RA:3 | intron:CG3694-RB:2 | intron:CG3694-RC:2 | CG3694-RA | "-" | CG3694-RB | "-" | CG3694-RC | "-"
 CG17005 in-situ | CG17005 | + | 42679 | 44968 | UPSTREAM | CG17005-RA | "-"

*********************** Rank 1430 [Score 13.542114]   GBROWSE*******************

 CG12425 in-situ | CG12425 | + | -80938 | -79383 | DOWNSTREAM | CG12425-RA | "-"
 CG4787 in-situ | CG4787 | + | 7337 | 9623 | UPSTREAM | CG4787-RA | "-"

*********************** Rank 1431 [Score 13.541382]   GBROWSE*******************

 CG15431 in-situ | CG15431 | - | -22478 | -25702 | UPSTREAM | CG15431-RA | "-"
 CG12677 in-situ | CG12677 | + | 2077 | 3229 | UPSTREAM | CG12677-RA | "-" | CG12677-RB | "-"


*********************** Rank 1432 [Score 13.540771]   GBROWSE*******************

 CG4383 in-situ | CG4383 | + | -241 | 5590 | INTRAGENIC | intron:CG4383-RA:1 | CG4383-RA | "-"
 CG4398 in-situ | CG4398 | + | 8948 | 9907 | UPSTREAM | CG4398-RA | "-"

*********************** Rank 1433 [Score 13.537659]   GBROWSE*******************

 CG14157 in-situ | Or67d | + | -7967 | -6547 | DOWNSTREAM | CG14157-RA | "-"
 CG6559 in-situ | CG6559 | - | 31542 | 15304 | DOWNSTREAM | CG6559-RA | "-"

*********************** Rank 1434 [Score 13.534424]   GBROWSE*******************

 CG31784 in-situ | CG31784 | - | -7941 | -31384 | UPSTREAM | CG31784-RA | "-" | CG31784-RB | "-"
 CG12621 in-situ | beat-IIIa | + | 24745 | 36298 | UPSTREAM | CG12621-RA | "-"

*********************** Rank 1435 [Score 13.528931]   GBROWSE*******************

 CG7345 in-situ | Sox21a | - | -23846 | -26666 | UPSTREAM | CG7345-RA | "-"
insitu CG32139 in-situ | Sox21b | - | 404 | -18537 | INTRAGENIC | intron:CG32139-RA:1 | CG32139-RA | "-"

*********************** Rank 1436 [Score 13.528198]   GBROWSE*******************

 CG5576 in-situ | imd | - | -7058 | -10839 | UPSTREAM | CG5576-RA | "-"
 CG5174 in-situ | CG5174 | + | 780 | 5380 | UPSTREAM | CG5174-RJ | "-" | CG5174-RI | "-" | CG5174-RA | "-" | CG5174-RH | "-" | CG5174-RK | "-" | CG5174-RG | "-" | CG5174-RB | "-"

*********************** Rank 1437 [Score 13.527466]   GBROWSE*******************

 CG3775 in-situ | CG3775 | + | -10280 | -7754 | DOWNSTREAM | CG3775-RA | "-"
 CG32648 in-situ | CG32648 | - | 547 | -20526 | INTRAGENIC | intron:CG32648-RA:1 | CG32648-RA | "-"

*********************** Rank 1438 [Score 13.526794]   GBROWSE*******************

 CG31672 in-situ | BEST:LD15963 | + | -7460 | -4931 | DOWNSTREAM | CG31672-RA | "-"
 CG15377 in-situ | Or22c | + | 1604 | 4982 | UPSTREAM | CG15377-RA | "-"

*********************** Rank 1439 [Score 13.526184]   GBROWSE*******************

 CG8808 in-situ | Pdk | + | -9876 | -2924 | DOWNSTREAM | CG8808-RA | "-"
insitu CG11804 in-situ | ced-6 | - | 5242 | -1649 | INTRAGENIC | intron:CG11804-RB:3 | intron:CG11804-RC:3 | intron:CG11804-RA:3 | CG11804-RB | "-" | CG11804-RC | "-" | CG11804-RA | "-"

*********************** Rank 1440 [Score 13.525330]   GBROWSE*******************

 CG31257 in-situ | CG31257 | + | -11498 | -9451 | DOWNSTREAM | CG31257-RA | "-"
 CG31418 in-situ | CG31418 | + | 18232 | 18952 | UPSTREAM | CG31418-RA | "-"

*********************** Rank 1441 [Score 13.524780]   GBROWSE*******************

 CG10202 in-situ | CG10202 | - | -10837 | -12964 | UPSTREAM | CG10202-RA | "-"
 CG10205 in-situ | CG10205 | + | 2009 | 3131 | UPSTREAM | CG10205-RB | "-" | CG10205-RA | "-"


*********************** Rank 1442 [Score 13.524414]   GBROWSE*******************

 CG10032 in-situ | CG10032 | + | -7917 | -7000 | DOWNSTREAM | CG10032-RA | "-"
 CG2595 in-situ | RacGAP84C | + | 5424 | 8123 | UPSTREAM | CG2595-RA | "-" | CG2595-RB | "-"


*********************** Rank 1443 [Score 13.520874]   GBROWSE*******************

 CG12147 in-situ | CG12147 | - | -4774 | -6662 | UPSTREAM | CG12147-RA | "-"
 CG14669 in-situ | CG14669 | + | 22572 | 23585 | UPSTREAM | CG14669-RA | "-"

*********************** Rank 1444 [Score 13.520325]   GBROWSE*******************

 CG15485 in-situ | CG15485 | - | -3111 | -5015 | UPSTREAM | CG15485-RA | "-"
insitu CG5525 in-situ | CG5525 | - | 20657 | 18045 | DOWNSTREAM | CG5525-RA | "-"

*********************** Rank 1445 [Score 13.518677]   GBROWSE*******************

 CG14390 in-situ | beat-Vc | - | -61508 | -69594 | UPSTREAM | CG14390-RA | "-"
 CG31345 in-situ | CG31345 | - | 820 | -4864 | INTRAGENIC | intron:CG31345-RA:1 | CG31345-RA | "-"

*********************** Rank 1446 [Score 13.517761]   GBROWSE*******************

 CG6414 in-situ | CG6414 | - | -78386 | -80736 | UPSTREAM | CG6414-RA | "-"
 CG32790 in-situ | CG32790 | + | 28182 | 29480 | UPSTREAM | CG32790-RA | "-"


*********************** Rank 1447 [Score 13.516113]   GBROWSE*******************

 CG30371 in-situ | CG30371 | - | -36495 | -38128 | UPSTREAM | CG30371-RA | "-"
 CG30358 in-situ | CG30358 | + | 14044 | 14737 | UPSTREAM | CG30358-RA | "-"

*********************** Rank 1448 [Score 13.512146]   GBROWSE*******************

 CG15532 in-situ | hdc | + | -88928 | -4693 | DOWNSTREAM | CG15532-RA | "-" | CG15532-RC | "-" | CG15532-RB | "-"
 CG1469 in-situ | Fer2LCH | + | 20970 | 23722 | UPSTREAM | CG1469-RA | "-" | CG1469-RB | "-" | CG1469-RC | "-"

*********************** Rank 1449 [Score 13.506165]   GBROWSE*******************

insitu CG32499 in-situ | CG32499 | - | -32446 | -81513 | UPSTREAM | CG32499-RA | "-"
 CG12446 in-situ | CG12446 | - | 146780 | 144132 | DOWNSTREAM | CG12446-RA | "-"


*********************** Rank 1450 [Score 13.498230]   GBROWSE*******************

 CG30131 in-situ | CG30131 | + | -14982 | -11661 | DOWNSTREAM | CG30131-RA | "-" | CG30131-RB | "-" | CG30131-RC | "-" | CG30131-RD | "-"
 CG11242 in-situ | CG11242 | - | 9405 | 8061 | DOWNSTREAM | CG11242-RA | "-"


*********************** Rank 1451 [Score 13.496460]   GBROWSE*******************

 CG11942 in-situ | CG11942 | - | -541 | -1044 | UPSTREAM | CG11942-RA | "-"
 CG11940 in-situ | CG11940 | - | 27511 | 8799 | DOWNSTREAM | CG11940-RA | "-" | CG11940-RB | "-"

*********************** Rank 1452 [Score 13.495422]   GBROWSE*******************

 CG31031 in-situ | CG31031 | + | -26934 | -26470 | DOWNSTREAM | CG31031-RA | "-"
 CG18682 in-situ | CG18682 | - | 34723 | 32042 | DOWNSTREAM | CG18682-RA | "-"

*********************** Rank 1453 [Score 13.490601]   GBROWSE*******************

 CG9380 in-situ | CG9380 | - | -26093 | -31301 | UPSTREAM | CG9380-RA | "-" | CG9380-RB | "-"
insitu highlight CG3340 in-situ | Kr | + | 11685 | 14604 | UPSTREAM | CG3340-RA | "-"

note: overlaps known module Kr_PD_AP_MT by 500 bases (module coords: 20252899-20260988)

*********************** Rank 1454 [Score 13.489258]   GBROWSE*******************

 CG12541 in-situ | CG12541 | - | -6095 | -8049 | UPSTREAM | CG12541-RA | "-"
insitu highlight CG14427 in-situ | CG14427 | + | 934 | 2095 | UPSTREAM | CG14427-RA | "-"

*********************** Rank 1455 [Score 13.487427]   GBROWSE*******************

 CG15475 in-situ | CG15475 | + | -8840 | -7899 | DOWNSTREAM | CG15475-RA | "-"
 CG17472 in-situ | CG17472 | + | 11099 | 11705 | UPSTREAM | CG17472-RA | "-"

*********************** Rank 1456 [Score 13.484863]   GBROWSE*******************

 CG12437 in-situ | raw | - | -9586 | -40349 | UPSTREAM | CG12437-RB | "-" | CG12437-RA | "-"
 CG12438 in-situ | CG12438 | + | 2181 | 2906 | UPSTREAM | CG12438-RA | "-"


*********************** Rank 1457 [Score 13.484619]   GBROWSE*******************

insitu highlight CG10325 in-situ | abd-A | - | -5962 | -28388 | UPSTREAM | CG10325-RA | "-" | CG10325-RB | "-"
 CG10349 in-situ | CG10349 | + | 43715 | 49097 | UPSTREAM | CG10349-RA | "-" | CG10349-RB | "-"

*********************** Rank 1458 [Score 13.482056]   GBROWSE*******************

 CG1448 in-situ | inx3 | - | -7969 | -13047 | UPSTREAM | CG1448-RA | "-"
 CG14529 in-situ | CG14529 | + | 5753 | 7798 | UPSTREAM | CG14529-RA | "-"


*********************** Rank 1459 [Score 13.480835]   GBROWSE*******************

insitu highlight CG4345 in-situ | grim | - | -35307 | -37002 | UPSTREAM | CG4345-RA | "-"
 CG4319 in-situ | rpr | - | 58682 | 57832 | DOWNSTREAM | CG4319-RA | "-"

*********************** Rank 1460 [Score 13.478394]   GBROWSE*******************

 CG3301 in-situ | CG3301 | - | -3860 | -5868 | UPSTREAM | CG3301-RA | "-" | CG3301-RB | "-"
 CG17298 in-situ | CG17298 | + | 3086 | 3944 | UPSTREAM | CG17298-RA | "-"


*********************** Rank 1461 [Score 13.477661]   GBROWSE*******************

 CG32081 in-situ | CG32081 | + | -9689 | -7835 | DOWNSTREAM | CG32081-RA | "-"
 CG6327 in-situ | CG6327 | - | 9370 | -911 | INTRAGENIC | intron:CG6327-RA:5 | intron:CG6327-RB:6 | intron:CG6327-RC:3 | CG6327-RA | "-" | CG6327-RB | "-" | CG6327-RC | "-"

*********************** Rank 1462 [Score 13.477173]   GBROWSE*******************

 CG18023 in-situ | Eip78C | + | -13805 | 23897 | INTRAGENIC | intron:CG18023-RA:3 | intron:CG18023-RB:3 | CG18023-RA | "-" | CG18023-RB | "-"
insitu CG9391 in-situ | CG9391 | - | 27616 | 26054 | DOWNSTREAM | CG9391-RB | "-" | CG9391-RA | "-"

*********************** Rank 1463 [Score 13.476440]   GBROWSE*******************

 CG31660 in-situ | CG31660 | + | -15666 | 6702 | INTRAGENIC | intron:CG31660-RB:3 | CG31660-RB | "-"
 CG11924 in-situ | Cf2 | - | 14582 | 8758 | DOWNSTREAM | CG11924-RA | "-" | CG11924-RB | "-" | CG11924-RC | "-" | CG11924-RD | "-"

*********************** Rank 1464 [Score 13.467896]   GBROWSE*******************

insitu CG5799 in-situ | dve | + | -27336 | 15119 | INTRAGENIC | intron:CG5799-RA:2 | intron:CG5799-RD:2 | intron:CG5799-RC:3 | CG5799-RA | "-" | CG5799-RD | "-" | CG5799-RB | "-" | CG5799-RC | "-"
insitu CG5819 in-situ | CG5819 | + | 22160 | 25659 | UPSTREAM | CG5819-RA | "-" | CG5819-RB | "-"

*********************** Rank 1465 [Score 13.465942]   GBROWSE*******************

 CG4319 in-situ | rpr | - | -21368 | -22218 | UPSTREAM | CG4319-RA | "-"
insitu CG13701 in-situ | skl | - | 20166 | 18785 | DOWNSTREAM | CG13701-RA | "-"

*********************** Rank 1466 [Score 13.462280]   GBROWSE*******************

 CG12063 in-situ | CG12063 | + | -39689 | -31564 | DOWNSTREAM | CG12063-RA | "-"
insitu CG1499 in-situ | CG1499 | + | 4470 | 27605 | UPSTREAM | CG1499-RA | "-" | CG1499-RB | "-"


*********************** Rank 1467 [Score 13.460083]   GBROWSE*******************

 CG9930 in-situ | E5 | - | -4130 | -10811 | UPSTREAM | CG9930-RA | "-"
insitu highlight CG2988 in-situ | ems | + | 23550 | 26315 | UPSTREAM | CG2988-RA | "-"

*********************** Rank 1468 [Score 13.459900]   GBROWSE*******************

 CG7391 in-situ | Clk | - | -14718 | -26350 | UPSTREAM | CG7391-RA | "-" | CG7391-RB | "-"
insitu CG32369 in-situ | CG32369 | - | 14085 | -11912 | INTRAGENIC | intron:CG32369-RA:1 | CG32369-RA | "-" | CG32369-RB | "-"

*********************** Rank 1469 [Score 13.459351]   GBROWSE*******************

 CG5462 in-situ | scrib | + | -27017 | 32827 | INTRAGENIC | intron:CG5462-RA:5 | intron:CG5462-RB:5 | intron:CG5462-RC:5 | intron:CG5462-RD:5 | CG5462-RA | "-" | CG5462-RB | "-" | CG5462-RC | "-" | CG5462-RD | "-"
 CG31082 in-situ | CG31082 | + | 20997 | 23438 | UPSTREAM | CG31082-RA | "-"

*********************** Rank 1470 [Score 13.457825]   GBROWSE*******************

insitu highlight CG10917 in-situ | fj | + | -39603 | -36052 | DOWNSTREAM | CG10917-RA | "-"
 CG5581 in-situ | Ote | - | 4827 | 3237 | DOWNSTREAM | CG5581-RA | "-"


*********************** Rank 1471 [Score 13.454102]   GBROWSE*******************

 CG14064 in-situ | beat-VI | + | -59558 | -4553 | DOWNSTREAM | CG14064-RA | "-"
 CG1894 in-situ | CG1894 | + | 6890 | 8236 | UPSTREAM | CG1894-RA | "-"

*********************** Rank 1472 [Score 13.453003]   GBROWSE*******************

insitu highlight CG2189 in-situ | Dfd | + | -34641 | -24046 | DOWNSTREAM | CG2189-RA | "-"
insitu highlight CG1030 in-situ | Scr | - | 22142 | -3357 | INTRAGENIC | intron:CG1030-RA:2 | CG1030-RA | "-"

*********************** Rank 1473 [Score 13.452698]   GBROWSE*******************

 CG15734 in-situ | CG15734 | - | -10146 | -10537 | UPSTREAM | CG15734-RA | "-"
 CG11356 in-situ | CG11356 | - | 6639 | 5998 | DOWNSTREAM | CG11356-RA | "-"

*********************** Rank 1474 [Score 13.448242]   GBROWSE*******************

 CG17673 in-situ | Acp70A | + | -23647 | -23360 | DOWNSTREAM | CG17673-RA | "-"
 CG14113 in-situ | CG14113 | + | 3936 | 4423 | UPSTREAM | CG14113-RA | "-"

*********************** Rank 1475 [Score 13.447021]   GBROWSE*******************

 CG17025 in-situ | CG17025 | - | -15853 | -23624 | UPSTREAM | CG17025-RA | "-"
 CG12538 in-situ | CG12538 | - | 41576 | 40992 | DOWNSTREAM | CG12538-RA | "-"


*********************** Rank 1476 [Score 13.446960]   GBROWSE*******************

 CG5290 in-situ | CG5290 | - | -21164 | -24242 | UPSTREAM | CG5290-RA | "-"
 CG32193 in-situ | CG32193 | + | 27939 | 31959 | UPSTREAM | CG32193-RA | "-"

*********************** Rank 1477 [Score 13.445801]   GBROWSE*******************

insitu highlight CG2988 in-situ | ems | + | -7950 | -5185 | DOWNSTREAM | CG2988-RA | "-"
 CG9929 in-situ | CG9929 | - | 33593 | 32539 | DOWNSTREAM | CG9929-RA | "-"

*********************** Rank 1478 [Score 13.445557]   GBROWSE*******************

 CG31007 in-situ | CG31007 | - | -21487 | -21975 | UPSTREAM | CG31007-RA | "-"
insitu CG12073 in-situ | 5-HT7 | - | 2985 | -44720 | INTRAGENIC | intron:CG12073-RA:1 | CG12073-RA | "-"

*********************** Rank 1479 [Score 13.444824]   GBROWSE*******************

 CG11898 in-situ | CG11898 | + | -6469 | -1113 | DOWNSTREAM | CG11898-RA | "-"
insitu CG14509 in-situ | CG14509 | - | 22732 | 1002 | DOWNSTREAM | CG14509-RA | "-"

*********************** Rank 1480 [Score 13.444336]   GBROWSE*******************

insitu CG11387 in-situ | ct | + | -32375 | 34500 | INTRAGENIC | intron:CG11387-RA:1 | intron:CG11387-RB:2 | CG11387-RA | "-" | CG11387-RB | "-"
 CG12690 in-situ | CHES-1-like | - | 55171 | 43364 | DOWNSTREAM | CG12690-RA | "-"

*********************** Rank 1481 [Score 13.442139]   GBROWSE*******************

 CG9995 in-situ | huntingtin | + | -6820 | 31289 | INTRAGENIC | intron:CG9995-RA:6 | CG9995-RA | "-"
 CG10001 in-situ | AR-2 | - | 41071 | 34047 | DOWNSTREAM | CG10001-RA | "-"


*********************** Rank 1482 [Score 13.439819]   GBROWSE*******************

 CG30500 in-situ | CG30500 | + | -1608 | -831 | DOWNSTREAM | CG30500-RA | "-"
 CG30501 in-situ | CG30501 | + | 8984 | 9870 | UPSTREAM | CG30501-RA | "-"

*********************** Rank 1483 [Score 13.439209]   GBROWSE*******************
[truncated: 1,046,872 more chars]
