# Supplementary material for: Cross-species comparison significantly improves genome-wide prediction of cis-regulatory modules in Drosophila
Source: BMC Bioinformatics. 2004 Sep 9;5:129. doi: 10.1186/1471-2105-5-129 (PMC521067; doi:10.1186/1471-2105-5-129)
Supplement: Additional File 5 — Predicted CRM's – single species List of predicted CRM's using single-species Stubb with a local, 1st order background, sorted in decreasing order of Stubb score. This list of predictions, as well as that in Additional File 4 (above), is meant to be as inclusive as possible; therefore, the specificity of the lowest ranked predictions may be poor. [file 1471-2105-5-129-S5.html]

```

```

```

```

```

```

```

```

```
*********************** EXPLANATIONS ************************
** List of predicted CRM's using STUBBSS, local 1st order background.
** "Score" refers to StubbSS score.
** List is sorted in decreasing order of score.
** "GBROWSE" link displays the CRM on genome browser.
** For each predicted CRM, two nearest genes are reported.
** "insitu" at the beginning of a gene line means the gene has insitu information.
** "highlight" at the beginning of a gene line means the gene is blastoderm patterned.
** Coordinates of a module are with respect to D.melanogaster Release 3.
** For each CRM, its coordinates are followed by its binding site contents.
** For example, "knirps  0.0375	7.0267" means there are 7.0267
** predicted occurrences of hunchback. (Ignore the number in second column.)
**
** To use Gbrowse : Click on "GBROWSE" link; Under "Dump, Searches and other Operations",
** choose "Annotate Fast Function Plot" and click "Configure"; Click "on" and the tracks
** you want to see, and click "Configure"; 
*************************************************************

*********************** Rank 1 [Score 22.748000] GBROWSE*******************

 CG6486 in-situ | CG6486 | + | -17316 | -16124 | DOWNSTREAM | CG6486-RA | "-"
insitu highlight CG6494 in-situ | h | + | 9119 | 12399 | UPSTREAM | CG6494-RA | "-"


note: overlaps known module h_rescue by 500 bases (module coords: 8620516-8642090)
note: overlaps known module h_stripe2_6 by 500 bases (module coords: 8625021-8627680)
note: overlaps known module h_stripe2_1990 by 500 bases (module coords: 8625021-8626101)
note: overlaps known module h_stripe2_1991 by 500 bases (module coords: 8625021-8630510)
note: overlaps known module h_stripe6_1991 by 500 bases (module coords: 8625021-8627680)
note: overlaps known module h_stripe6_1990 by 484 bases (module coords: 8625566-8626101)

*********************** Rank 2 [Score 19.628100] GBROWSE*******************

 CG12134 in-situ | CG12134 | + | -4368 | -2734 | DOWNSTREAM | CG12134-RB | "-" | CG12134-RA | "-"
insitu highlight CG2328 in-situ | eve | + | 2074 | 5262 | UPSTREAM | CG2328-RA | "-"


note: overlaps known module eve_stripe3_7 by 495 bases (module coords: 5036134-5036644)

*********************** Rank 3 [Score 19.105000] GBROWSE*******************

 CG15544 in-situ | CG15544 | + | -21812 | -7628 | DOWNSTREAM | CG15544-RA | "-"
insitu highlight CG1378 in-situ | tll | + | 2706 | 4711 | UPSTREAM | CG1378-RA | "-"


note: overlaps known module tll_K11_post by 361 bases (module coords: 26664631-26665110)
note: overlaps known module tll_rescue by 500 bases (module coords: 26661256-26671461)

*********************** Rank 4 [Score 18.223300] GBROWSE*******************

 CG1338 in-situ | CG1338 | - | -2094 | -6506 | UPSTREAM | CG1338-RA | "-" | CG1338-RB | "-"
insitu highlight CG1849 in-situ | run | + | 17106 | 19991 | UPSTREAM | CG1849-RA | "-"

*********************** Rank 5 [Score 15.327300] GBROWSE*******************

 CG1338 in-situ | CG1338 | - | -15744 | -20156 | UPSTREAM | CG1338-RA | "-" | CG1338-RB | "-"
insitu highlight CG1849 in-situ | run | + | 3456 | 6341 | UPSTREAM | CG1849-RA | "-"

note: overlaps known module run_7stripes by 500 bases (module coords: 20361765-20366856)

*********************** Rank 6 [Score 15.153600] GBROWSE*******************

 CG6486 in-situ | CG6486 | + | -21666 | -20474 | DOWNSTREAM | CG6486-RA | "-"
insitu highlight CG6494 in-situ | h | + | 4769 | 8049 | UPSTREAM | CG6494-RA | "-"

note: overlaps known module h_rescue by 500 bases (module coords: 8620516-8642090)
note: overlaps known module h_stripe1_1990 by 500 bases (module coords: 8629353-8630638)
note: overlaps known module h_stripe1_1991 by 500 bases (module coords: 8629635-8630510)
note: overlaps known module h_stripe2_1991 by 500 bases (module coords: 8625021-8630510)
note: overlaps known module h_stripe5_1990a by 500 bases (module coords: 8627668-8630638)
note: overlaps known module h_stripe1_5_1991 by 500 bases (module coords: 8627680-8630510)

*********************** Rank 7 [Score 15.093000] GBROWSE*******************

insitu CG32139 in-situ | Sox21b | - | -41946 | -60887 | UPSTREAM | CG32139-RA | "-"
insitu highlight CG5893 in-situ | D | - | 5350 | 2331 | DOWNSTREAM | CG5893-RA | "-"

*********************** Rank 8 [Score 14.794200] GBROWSE*******************

 CG7229 in-situ | CG7229 | + | -13416 | -11091 | DOWNSTREAM | CG7229-RA | "-"
insitu highlight CG7230 in-situ | rib | + | 2396 | 8066 | UPSTREAM | CG7230-RA | "-"

*********************** Rank 9 [Score 14.533400] GBROWSE*******************

 CG15544 in-situ | CG15544 | + | -24112 | -9928 | DOWNSTREAM | CG15544-RA | "-"
insitu highlight CG1378 in-situ | tll | + | 406 | 2411 | UPSTREAM | CG1378-RA | "-"

note: overlaps known module tll_D3_anter_post by 347 bases (module coords: 26667029-26667396)
note: overlaps known module tll_rescue by 500 bases (module coords: 26661256-26671461)

*********************** Rank 10 [Score 14.410000] GBROWSE*******************

insitu highlight CG4717 in-situ | kni | - | -1599 | -4632 | UPSTREAM | CG4717-RA | "-"
insitu CG13253 in-situ | CG13253 | - | 22236 | 19833 | DOWNSTREAM | CG13253-RA | "-"


note: overlaps known module kni_223_1995 by 47 bases (module coords: 20615953-20616109)
note: overlaps known module kni_64_1995 by 67 bases (module coords: 20615887-20615953)
note: overlaps known module kni_kd by 455 bases (module coords: 20615078-20615954)

*********************** Rank 11 [Score 14.239000] GBROWSE*******************

 CG15494 in-situ | CG15494 | + | -1746 | -1531 | DOWNSTREAM | CG15494-RA | "-"
 CG5442 in-situ | SC35 | - | 916 | -1261 | INTRAGENIC | intron:CG5442-RB:2 | CG5442-RB | "-"


*********************** Rank 12 [Score 13.905900] GBROWSE*******************

 CG1338 in-situ | CG1338 | - | -9694 | -14106 | UPSTREAM | CG1338-RA | "-" | CG1338-RB | "-"
insitu highlight CG1849 in-situ | run | + | 9506 | 12391 | UPSTREAM | CG1849-RA | "-"

note: overlaps known module run_stripe3 by 500 bases (module coords: 20355609-20357923)
note: overlaps known module run_stripe7 by 500 bases (module coords: 20352427-20358012)

*********************** Rank 13 [Score 13.851300] GBROWSE*******************

 CG4774 in-situ | CG4774 | - | -29643 | -31779 | UPSTREAM | CG4774-RB | "-" | CG4774-RA | "-" | CG4774-RC | "-"
insitu CG31092 in-situ | CG31092 | - | 23452 | -18247 | INTRAGENIC | intron:CG31092-RA:3 | intron:CG31092-RB:2 | CG31092-RA | "-" | CG31092-RB | "-"


*********************** Rank 14 [Score 13.845500] GBROWSE*******************

insitu CG10191 in-situ | CG10191 | - | -2544 | -4239 | UPSTREAM | CG10191-RA | "-"
 CG32120 in-situ | Ly | - | 8593 | 3697 | DOWNSTREAM | CG32120-RA | "-"

*********************** Rank 15 [Score 13.501900] GBROWSE*******************

 CG30040 in-situ | jeb | - | -11815 | -40065 | UPSTREAM | CG30040-RA | "-"
 CG18343 in-situ | CG18343 | + | 8115 | 8589 | UPSTREAM | CG18343-RA | "-"

*********************** Rank 16 [Score 13.207300] GBROWSE*******************

 CG6486 in-situ | CG6486 | + | -20316 | -19124 | DOWNSTREAM | CG6486-RA | "-"
insitu highlight CG6494 in-situ | h | + | 6119 | 9399 | UPSTREAM | CG6494-RA | "-"


note: overlaps known module h_rescue by 500 bases (module coords: 8620516-8642090)
note: overlaps known module h_stripe2_1991 by 500 bases (module coords: 8625021-8630510)
note: overlaps known module h_stripe5_1990a by 500 bases (module coords: 8627668-8630638)
note: overlaps known module h_stripe5_1990b by 500 bases (module coords: 8627680-8629353)
note: overlaps known module h_stripe1_5_1991 by 500 bases (module coords: 8627680-8630510)
note: overlaps known module h_stripe5_1993 by 260 bases (module coords: 8628790-8629353)

*********************** Rank 17 [Score 12.582000] GBROWSE*******************

 CG12425 in-situ | CG12425 | + | -3288 | -1733 | DOWNSTREAM | CG12425-RA | "-"
 CG4787 in-situ | CG4787 | + | 84987 | 87273 | UPSTREAM | CG4787-RA | "-"


*********************** Rank 18 [Score 12.522400] GBROWSE*******************

 CG6486 in-situ | CG6486 | + | -16066 | -14874 | DOWNSTREAM | CG6486-RA | "-"
insitu highlight CG6494 in-situ | h | + | 10369 | 13649 | UPSTREAM | CG6494-RA | "-"

note: overlaps known module h_rescue by 500 bases (module coords: 8620516-8642090)
note: overlaps known module h_stripe7_1990a by 500 bases (module coords: 8623548-8625021)
note: overlaps known module h_stripe7_1997 by 420 bases (module coords: 8623787-8624719)
note: overlaps known module h_stripe7_1990b by 500 bases (module coords: 8623984-8625021)

*********************** Rank 19 [Score 12.427600] GBROWSE*******************

 CG13293 in-situ | CG13293 | + | -1173 | 1592 | INTRAGENIC | intron:CG13293-RA:4 | CG13293-RA | "-"
 CG10469 in-situ | CG10469 | - | 4643 | 3781 | DOWNSTREAM | CG10469-RA | "-"

*********************** Rank 20 [Score 12.409700] GBROWSE*******************

 CG8351 in-situ | CG8351 | + | -260 | 4423 | INTRAGENIC | intron:CG8351-RA:1 | CG8351-RA | "-"
 CG9839 in-situ | CG9839 | - | 3817 | 1658 | DOWNSTREAM | CG9839-RA | "-"

*********************** Rank 21 [Score 12.294500] GBROWSE*******************

 CG7892 in-situ | nmo | + | -40420 | 30060 | INTRAGENIC | intron:CG7892-RA:3 | intron:CG7892-RB:2 | intron:CG7892-RE:3 | intron:CG7892-RC:2 | intron:CG7892-RF:2 | intron:CG7892-RG:2 | CG7892-RA | "-" | CG7892-RB | "-" | CG7892-RE | "-" | CG7892-RC | "-" | CG7892-RF | "-" | CG7892-RG | "-" | CG7892-RD | "-"
 CG7574 in-situ | bip1 | - | 38400 | 34040 | DOWNSTREAM | CG7574-RA | "-"


*********************** Rank 22 [Score 12.216400] GBROWSE*******************

 CG13723 in-situ | CG13723 | + | -19420 | -19088 | DOWNSTREAM | CG13723-RA | "-"
 CG6485 in-situ | CG6485 | - | 36890 | 36041 | DOWNSTREAM | CG6485-RA | "-"


*********************** Rank 23 [Score 12.166100] GBROWSE*******************

 CG31184 in-situ | CG31184 | + | -6202 | -5592 | DOWNSTREAM | CG31184-RA | "-"
insitu CG5405 in-situ | KrT95D | - | 29189 | -4184 | INTRAGENIC | intron:CG5405-RB:6 | intron:CG5405-RA:5 | CG5405-RB | "-" | CG5405-RA | "-"


*********************** Rank 24 [Score 12.121800] GBROWSE*******************

 CG14033 in-situ | CG14033 | + | -10322 | -8790 | DOWNSTREAM | CG14033-RA | "-"
 CG14032 in-situ | Cyp4ac1 | + | 10972 | 12870 | UPSTREAM | CG14032-RA | "-"

*********************** Rank 25 [Score 12.104700] GBROWSE*******************

 CG5737 in-situ | dmrt93B | + | -5004 | -2213 | DOWNSTREAM | CG5737-RA | "-"
 CG7056 in-situ | CG7056 | - | 1519 | -2097 | INTRAGENIC | intron:CG7056-RA:1 | CG7056-RA | "-"

*********************** Rank 26 [Score 12.058600] GBROWSE*******************

 CG17681 in-situ | CG17681 | + | -9138 | 2743 | INTRAGENIC | intron:CG17681-RA:1 | CG17681-RA | "-" | CG17681-RB | "-"
 CG15154 in-situ | Socs36E | - | 743 | -12999 | INTRAGENIC | intron:CG15154-RA:1 | intron:CG15154-RB:1 | CG15154-RA | "-" | CG15154-RB | "-"

*********************** Rank 27 [Score 11.983800] GBROWSE*******************

 CG1530 in-situ | CG1530 | + | -2285 | 2397 | INTRAGENIC | intron:CG1530-RA:1 | CG1530-RA | "-"
 CG2206 in-situ | CG2206 | - | 7752 | 2223 | DOWNSTREAM | CG2206-RA | "-"

*********************** Rank 28 [Score 11.979600] GBROWSE*******************

 CG12496 in-situ | EG:BACH7M4.4 | - | -1229 | -2771 | UPSTREAM | CG12496-RA | "-"
 CG32797 in-situ | CG32797 | - | 13577 | 12969 | DOWNSTREAM | CG32797-RA | "-"

*********************** Rank 29 [Score 11.774100] GBROWSE*******************

 CG5988 in-situ | CG5988 | - | -12207 | -13993 | UPSTREAM | CG5988-RA | "-"
 CG15059 in-situ | CG15059 | - | 4115 | 3493 | DOWNSTREAM | CG15059-RB | "-" | CG15059-RA | "-"

*********************** Rank 30 [Score 11.701800] GBROWSE*******************

 CG18468 in-situ | CG18468 | + | -1174 | -2 | DOWNSTREAM | CG18468-RA | "-"
 CG18467 in-situ | CG18467 | + | 8379 | 10264 | UPSTREAM | CG18467-RA | "-"

*********************** Rank 31 [Score 11.574500] GBROWSE*******************

 CG7580 in-situ | CG7580 | + | -2610 | -1525 | DOWNSTREAM | CG7580-RA | "-"
 CG13733 in-situ | CG13733 | - | 1555 | 416 | DOWNSTREAM | CG13733-RA | "-"

*********************** Rank 32 [Score 11.555700] GBROWSE*******************

 CG13194 in-situ | CG13194 | + | -41114 | -38737 | DOWNSTREAM | CG13194-RA | "-"
 CG13193 in-situ | CG13193 | + | 3416 | 4524 | UPSTREAM | CG13193-RA | "-"

*********************** Rank 33 [Score 11.409900] GBROWSE*******************

insitu CG11387 in-situ | ct | + | -32325 | 34550 | INTRAGENIC | intron:CG11387-RA:1 | intron:CG11387-RB:2 | CG11387-RA | "-" | CG11387-RB | "-"
 CG12690 in-situ | CHES-1-like | - | 55221 | 43414 | DOWNSTREAM | CG12690-RA | "-"

*********************** Rank 34 [Score 11.386300] GBROWSE*******************

 CG14280 in-situ | CG14280 | + | -29745 | -27129 | DOWNSTREAM | CG14280-RA | "-"
insitu CG3619 in-situ | Dl | - | 39202 | 15720 | DOWNSTREAM | CG3619-RA | "-" | CG3619-RB | "-"

*********************** Rank 35 [Score 11.377700] GBROWSE*******************

 CG13841 in-situ | CG13841 | - | -740 | -1548 | UPSTREAM | CG13841-RA | "-"
 CG7029 in-situ | CG7029 | + | 16062 | 24347 | UPSTREAM | CG7029-RA | "-"

*********************** Rank 36 [Score 11.364600] GBROWSE*******************

insitu CG7018 in-situ | Ets65A | + | -18070 | 7738 | INTRAGENIC | intron:CG7018-RA:7 | intron:CG7018-RB:4 | CG7018-RA | "-" | CG7018-RB | "-"
 CG12755 in-situ | l(3)mbn | - | 13241 | 7694 | DOWNSTREAM | CG12755-RA | "-" | CG12755-RB | "-"

*********************** Rank 37 [Score 11.358100] GBROWSE*******************

 CG8853 in-situ | CG8853 | + | -7378 | -5755 | DOWNSTREAM | CG8853-RA | "-"
insitu highlight CG10016 in-situ | drm | + | 970 | 9805 | UPSTREAM | CG10016-RB | "-" | CG10016-RA | "-"

*********************** Rank 38 [Score 11.316200] GBROWSE*******************

 CG13193 in-situ | CG13193 | + | -12684 | -11576 | DOWNSTREAM | CG13193-RA | "-"
 CG12444 in-situ | CG12444 | - | 8356 | 6671 | DOWNSTREAM | CG12444-RB | "-" | CG12444-RA | "-"

*********************** Rank 39 [Score 11.303500] GBROWSE*******************

 CG30111 in-situ | CG30111 | + | -9680 | -6301 | DOWNSTREAM | CG30111-RA | "-"
 CG11430 in-situ | olf186-F | + | 19420 | 36545 | UPSTREAM | CG11430-RB | "-" | CG11430-RC | "-" | CG11430-RA | "-"

*********************** Rank 40 [Score 11.215300] GBROWSE*******************

 CG6171 in-situ | CG6171 | - | -1829 | -2792 | UPSTREAM | CG6171-RA | "-"
 CG6156 in-situ | CG6156 | - | 1020 | -1981 | INTRAGENIC | intron:CG6156-RA:4 | intron:CG6156-RB:3 | CG6156-RA | "-" | CG6156-RB | "-"

*********************** Rank 41 [Score 11.195400] GBROWSE*******************

 CG14233 in-situ | meso18E | - | -7949 | -14592 | UPSTREAM | CG14233-RA | "-"
 CG12531 in-situ | CG12531 | + | 1050 | 5696 | UPSTREAM | CG12531-RA | "-"

*********************** Rank 42 [Score 11.184300] GBROWSE*******************

 CG9380 in-situ | CG9380 | - | -33693 | -38901 | UPSTREAM | CG9380-RA | "-" | CG9380-RB | "-"
insitu highlight CG3340 in-situ | Kr | + | 4085 | 7004 | UPSTREAM | CG3340-RA | "-"

note: overlaps known module Kr_730 by 411 bases (module coords: 20266189-20266919)
note: overlaps known module Kr_CD1 by 411 bases (module coords: 20266189-20267347)

*********************** Rank 43 [Score 11.177900] GBROWSE*******************

insitu highlight CG4717 in-situ | kni | - | -4649 | -7682 | UPSTREAM | CG4717-RA | "-"
insitu CG13253 in-situ | CG13253 | - | 19186 | 16783 | DOWNSTREAM | CG13253-RA | "-"

*********************** Rank 44 [Score 11.175800] GBROWSE*******************

 CG17650 in-situ | CG17650 | - | -19257 | -20049 | UPSTREAM | CG17650-RA | "-"
 CG31936 in-situ | Gr22e | + | 5727 | 6951 | UPSTREAM | CG31936-RA | "-"

*********************** Rank 45 [Score 11.145600] GBROWSE*******************

insitu highlight CG14427 in-situ | CG14427 | + | -4166 | -3005 | DOWNSTREAM | CG14427-RA | "-"
 CG14426 in-situ | nullo | + | 4647 | 5610 | UPSTREAM | CG14426-RA | "-"

*********************** Rank 46 [Score 11.112500] GBROWSE*******************

 CG14532 in-situ | CG14532 | - | -13384 | -13977 | UPSTREAM | CG14532-RA | "-"
 CG7233 in-situ | CG7233 | - | 62771 | 61755 | DOWNSTREAM | CG7233-RA | "-"

*********************** Rank 47 [Score 11.090500] GBROWSE*******************

 CG31439 in-situ | CG31439 | - | -18745 | -19832 | UPSTREAM | CG31439-RA | "-"
insitu CG10772 in-situ | Fur1 | - | 53693 | -70703 | INTRAGENIC | intron:CG10772-RA:2 | intron:CG10772-RB:2 | intron:CG10772-RC:2 | intron:CG10772-RD:1 | CG10772-RA | "-" | CG10772-RB | "-" | CG10772-RC | "-" | CG10772-RD | "-"

*********************** Rank 48 [Score 10.973300] GBROWSE*******************

insitu CG15598 in-situ | CG15598 | + | -1630 | 10383 | INTRAGENIC | intron:CG15598-RA:1 | CG15598-RA | "-"
insitu CG1169 in-situ | CG1169 | + | 13322 | 14886 | UPSTREAM | CG1169-RA | "-"

*********************** Rank 49 [Score 10.956500] GBROWSE*******************

 CG17453 in-situ | Cyp317a1 | + | -6979 | -5423 | DOWNSTREAM | CG17453-RA | "-"
 CG10249 in-situ | BcDNA:GH03482 | - | 5743 | -4999 | INTRAGENIC | intron:CG10249-RC:3 | intron:CG10249-RA:1 | CG10249-RC | "-" | CG10249-RA | "-" | CG10249-RB | "-"

*********************** Rank 50 [Score 10.935700] GBROWSE*******************

insitu CG6586 in-situ | tan | + | -3164 | -1500 | DOWNSTREAM | CG6586-RA | "-"
 CG6592 in-situ | CG6592 | + | 1604 | 3036 | UPSTREAM | CG6592-RA | "-"

*********************** Rank 51 [Score 10.932900] GBROWSE*******************

insitu highlight CG10002 in-situ | fkh | - | -1229 | -4496 | UPSTREAM | CG10002-RA | "-"
insitu CG10009 in-situ | Noa36 | - | 12967 | 11746 | DOWNSTREAM | CG10009-RA | "-"

*********************** Rank 52 [Score 10.906500] GBROWSE*******************

 CG13388 in-situ | Akap200 | + | -2179 | 13115 | INTRAGENIC | intron:CG13388-RA:1 | intron:CG13388-RD:1 | CG13388-RA | "-" | CG13388-RD | "-" | CG13388-RC | "-" | CG13388-RB | "-"
 CG31894 in-situ | CG31894 | - | 8249 | 7681 | DOWNSTREAM | CG31894-RA | "-"

*********************** Rank 53 [Score 10.906100] GBROWSE*******************

insitu highlight CG2047 in-situ | ftz | + | -3653 | -1749 | DOWNSTREAM | CG2047-RA | "-"
 CG31488 in-situ | CG31488 | + | 89757 | 90386 | UPSTREAM | CG31488-RA | "-"

note: overlaps known module ftz_3prime_element by 337 bases (module coords: 2692342-2694036)
note: overlaps known module ftz_rescue_construct by 337 bases (module coords: 2683630-2694036)

*********************** Rank 54 [Score 10.897100] GBROWSE*******************

 CG17097 in-situ | CG17097 | + | -4706 | -1097 | DOWNSTREAM | CG17097-RB | "-" | CG17097-RA | "-"
 CG17098 in-situ | CG17098 | + | 837 | 3369 | UPSTREAM | CG17098-RA | "-"

*********************** Rank 55 [Score 10.889200] GBROWSE*******************

 CG14238 in-situ | CG14238 | + | -1910 | 484 | INTRAGENIC | intron:CG14238-RA:5 | CG14238-RA | "-"
 CG5455 in-situ | CG5455 | + | 1266 | 9747 | UPSTREAM | CG5455-RA | "-" | CG5455-RB | "-" | CG5455-RC | "-"

*********************** Rank 56 [Score 10.851600] GBROWSE*******************

insitu highlight CG2047 in-situ | ftz | + | -65203 | -63299 | DOWNSTREAM | CG2047-RA | "-"
 CG31488 in-situ | CG31488 | + | 28207 | 28836 | UPSTREAM | CG31488-RA | "-"

*********************** Rank 57 [Score 10.843400] GBROWSE*******************

 CG2204 in-situ | G-oalpha47A | + | -12206 | 12497 | INTRAGENIC | intron:CG2204-RB:4 | intron:CG2204-RC:5 | intron:CG2204-RD:4 | intron:CG2204-RE:4 | intron:CG2204-RF:4 | intron:CG2204-RG:4 | intron:CG2204-RA:4 | CG2204-RB | "-" | CG2204-RC | "-" | CG2204-RD | "-" | CG2204-RE | "-" | CG2204-RF | "-" | CG2204-RG | "-" | CG2204-RA | "-"
insitu CG18377 in-situ | Cyp49a1 | - | 8717 | -825 | INTRAGENIC | intron:CG18377-RA:12 | intron:CG18377-RC:8 | intron:CG18377-RB:7 | CG18377-RA | "-" | CG18377-RC | "-" | CG18377-RB | "-"

*********************** Rank 58 [Score 10.841000] GBROWSE*******************

 CG12478 in-situ | bru-3 | - | -11583 | -140414 | UPSTREAM | CG12478-RA | "-" | CG12478-RB | "-"
 CG8757 in-situ | CG8757 | - | 155220 | 154254 | DOWNSTREAM | CG8757-RA | "-"

*********************** Rank 59 [Score 10.838400] GBROWSE*******************

 CG6414 in-situ | CG6414 | - | -47986 | -50336 | UPSTREAM | CG6414-RA | "-"
 CG32790 in-situ | CG32790 | + | 58582 | 59880 | UPSTREAM | CG32790-RA | "-"

*********************** Rank 60 [Score 10.765700] GBROWSE*******************

insitu CG7011 in-situ | CG7011 | - | -36297 | -39052 | UPSTREAM | CG7011-RA | "-"
 CG6888 in-situ | CG6888 | + | 7923 | 8671 | UPSTREAM | CG6888-RA | "-"

*********************** Rank 61 [Score 10.760100] GBROWSE*******************

 CG5790 in-situ | CG5790 | + | -10813 | -8440 | DOWNSTREAM | CG5790-RA | "-"
 CG5803 in-situ | Fas3 | + | 14384 | 86873 | UPSTREAM | CG5803-RA | "-" | CG5803-RB | "-"

*********************** Rank 62 [Score 10.754400] GBROWSE*******************

 CG4881 in-situ | salr | + | -75374 | -67282 | DOWNSTREAM | CG4881-RA | "-" | CG4881-RB | "-"
insitu highlight CG6464 in-situ | salm | - | 4924 | -6368 | INTRAGENIC | intron:CG6464-RA:1 | CG6464-RA | "-"

*********************** Rank 63 [Score 10.749200] GBROWSE*******************

 CG31738 in-situ | CG31738 | + | -11872 | 28110 | INTRAGENIC | intron:CG31738-RB:1 | CG31738-RB | "-" | CG31738-RA | "-"
 CG5996 in-situ | trpgamma | - | 42750 | 30945 | DOWNSTREAM | CG5996-RA | "-" | CG5996-RB | "-"


*********************** Rank 64 [Score 10.702500] GBROWSE*******************

insitu highlight CG17390 in-situ | CG17390 | + | -7138 | -417 | DOWNSTREAM | CG17390-RA | "-"
 CG10109 in-situ | L | + | 15329 | 31438 | UPSTREAM | CG10109-RA | "-"

*********************** Rank 65 [Score 10.697500] GBROWSE*******************

 CG4162 in-situ | lace | + | -44406 | -38918 | DOWNSTREAM | CG4162-RA | "-"
 CG15256 in-situ | BG:DS04862.2 | + | 6956 | 19734 | UPSTREAM | CG15256-RA | "-"

*********************** Rank 66 [Score 10.639100] GBROWSE*******************

insitu CG6847 in-situ | CG6847 | + | -2328 | 10375 | INTRAGENIC | intron:CG6847-RA:1 | CG6847-RA | "-"
 CG32494 in-situ | CG32494 | + | 86200 | 87437 | UPSTREAM | CG32494-RA | "-"


*********************** Rank 67 [Score 10.614300] GBROWSE*******************

 CG13958 in-situ | CG13958 | + | -56749 | -55133 | DOWNSTREAM | CG13958-RA | "-"
 CG13959 in-situ | CG13959 | + | 2213 | 3301 | UPSTREAM | CG13959-RA | "-"

*********************** Rank 68 [Score 10.604800] GBROWSE*******************

 CG8786 in-situ | CG8786 | - | -529 | -5578 | UPSTREAM | CG8786-RB | "-"
insitu CG8782 in-situ | CG8782 | - | 6506 | 3933 | DOWNSTREAM | CG8782-RA | "-"

*********************** Rank 69 [Score 10.575500] GBROWSE*******************

 CG4439 in-situ | CG4439 | + | -5733 | -3639 | DOWNSTREAM | CG4439-RA | "-"
 CG7813 in-situ | CG7813 | - | 2554 | 14 | DOWNSTREAM | CG7813-RA | "-"

*********************** Rank 70 [Score 10.562000] GBROWSE*******************

 CG30437 in-situ | CG30437 | + | -8993 | 31422 | INTRAGENIC | intron:CG30437-RA:1 | intron:CG30437-RC:1 | intron:CG30437-RB:1 | CG30437-RA | "-" | CG30437-RC | "-" | CG30437-RB | "-"
 CG32838 in-situ | CG32838 | + | 31709 | 32284 | UPSTREAM | CG32838-RA | "-"

*********************** Rank 71 [Score 10.544400] GBROWSE*******************

 CG12540 in-situ | CG12540 | + | -14349 | -13244 | DOWNSTREAM | CG12540-RA | "-"
insitu CG14414 in-situ | CG14414 | + | 70122 | 71688 | UPSTREAM | CG14414-RA | "-" | CG14414-RC | "-" | CG14414-RB | "-"


*********************** Rank 72 [Score 10.530800] GBROWSE*******************

 CG15544 in-situ | CG15544 | + | -23162 | -8978 | DOWNSTREAM | CG15544-RA | "-"
insitu highlight CG1378 in-situ | tll | + | 1356 | 3361 | UPSTREAM | CG1378-RA | "-"

note: overlaps known module tll_CD1_anter by 457 bases (module coords: 26666143-26666638)
note: overlaps known module tll_rescue by 500 bases (module coords: 26661256-26671461)

*********************** Rank 73 [Score 10.498400] GBROWSE*******************

 CG9792 in-situ | yellow-e | - | -4575 | -10317 | UPSTREAM | CG9792-RA | "-"
 CG14376 in-situ | CG14376 | + | 414 | 3281 | UPSTREAM | CG14376-RA | "-"

*********************** Rank 74 [Score 10.495800] GBROWSE*******************

 CG14947 in-situ | CG14947 | + | -4206 | -3394 | DOWNSTREAM | CG14947-RA | "-"
insitu highlight CG6716 in-situ | prd | - | 5427 | 1968 | DOWNSTREAM | CG6716-RB | "-" | CG6716-RA | "-"

note: overlaps known module prd_rescue by 500 bases (module coords: 12066868-12085327)

*********************** Rank 75 [Score 10.490800] GBROWSE*******************

 CG3006 in-situ | Fmo-1 | + | -757 | 920 | INTRAGENIC | intron:CG3006-RA:3 | CG3006-RA | "-"
 CG3017 in-situ | Alas | + | 969 | 3016 | UPSTREAM | CG3017-RA | "-"

*********************** Rank 76 [Score 10.487800] GBROWSE*******************

 CG31912 in-situ | CG31912 | + | -12483 | -11296 | DOWNSTREAM | CG31912-RA | "-"
 CG31913 in-situ | CG31913 | + | 1796 | 2658 | UPSTREAM | CG31913-RA | "-"

*********************** Rank 77 [Score 10.468700] GBROWSE*******************

 CG9284 in-situ | BEST:GH11908 | + | -2275 | -1358 | DOWNSTREAM | CG9284-RA | "-"
 CG13492 in-situ | CG13492 | - | 24511 | 15145 | DOWNSTREAM | CG13492-RA | "-" | CG13492-RB | "-"

*********************** Rank 78 [Score 10.461500] GBROWSE*******************

 CG31439 in-situ | CG31439 | - | -41395 | -42482 | UPSTREAM | CG31439-RA | "-"
insitu CG10772 in-situ | Fur1 | - | 31043 | -93353 | INTRAGENIC | intron:CG10772-RA:1 | intron:CG10772-RB:1 | intron:CG10772-RC:1 | intron:CG10772-RD:1 | CG10772-RA | "-" | CG10772-RB | "-" | CG10772-RC | "-" | CG10772-RD | "-"


*********************** Rank 79 [Score 10.433200] GBROWSE*******************

insitu CG6134 in-situ | spz | - | -1345 | -6343 | UPSTREAM | CG6134-RA | "-" | CG6134-RB | "-" | CG6134-RC | "-" | CG6134-RD | "-" | CG6134-RE | "-" | CG6134-RF | "-" | CG6134-RG | "-" | CG6134-RH | "-" | CG6134-RI | "-" | CG6134-RJ | "-"
 CG14257 in-situ | CG14257 | + | 6856 | 10804 | UPSTREAM | CG14257-RA | "-"

*********************** Rank 80 [Score 10.421600] GBROWSE*******************

 CG15001 in-situ | CG15001 | - | -325 | -651 | UPSTREAM | CG15001-RA | "-"
 CG15002 in-situ | mas | - | 6487 | 680 | DOWNSTREAM | CG15002-RB | "-"

*********************** Rank 81 [Score 10.406900] GBROWSE*******************

 CG15899 in-situ | Ca-alpha1T | - | -49136 | -87471 | UPSTREAM | CG15899-RB | "-"
 CG32750 in-situ | CG32750 | - | 1893 | 264 | DOWNSTREAM | CG32750-RA | "-"

*********************** Rank 82 [Score 10.373200] GBROWSE*******************

 CG30048 in-situ | CG30048 | + | -4437 | -1381 | DOWNSTREAM | CG30048-RA | "-"
 CG8505 in-situ | CG8505 | + | 1728 | 4628 | UPSTREAM | CG8505-RA | "-"

*********************** Rank 83 [Score 10.354400] GBROWSE*******************

 CG15747 in-situ | CG15747 | + | -16308 | -13622 | DOWNSTREAM | CG15747-RB | "-" | CG15747-RA | "-"
 CG10617 in-situ | CG10617 | + | 4609 | 13540 | UPSTREAM | CG10617-RA | "-"

*********************** Rank 84 [Score 10.349700] GBROWSE*******************

insitu highlight CG4889 in-situ | wg | + | -25658 | -16564 | DOWNSTREAM | CG4889-RA | "-" | CG4889-RB | "-"
 CG4969 in-situ | Wnt6 | + | 18079 | 19461 | UPSTREAM | CG4969-RA | "-"

*********************** Rank 85 [Score 10.346100] GBROWSE*******************

 CG7313 in-situ | CG7313 | + | -13124 | -12375 | DOWNSTREAM | CG7313-RA | "-"
 CG5103 in-situ | CG5103 | - | 29146 | 26896 | DOWNSTREAM | CG5103-RA | "-"

*********************** Rank 86 [Score 10.313600] GBROWSE*******************

 CG12454 in-situ | CG12454 | + | -8599 | -8411 | DOWNSTREAM | CG12454-RA | "-"
 CG32614 in-situ | CG32614 | + | 53917 | 54627 | UPSTREAM | CG32614-RA | "-"

*********************** Rank 87 [Score 10.306300] GBROWSE*******************

 CG17601 in-situ | CG17601 | + | -69163 | -68501 | DOWNSTREAM | CG17601-RA | "-"
insitu CG32499 in-situ | CG32499 | - | 16404 | -32663 | INTRAGENIC | intron:CG32499-RA:3 | CG32499-RA | "-"


*********************** Rank 88 [Score 10.296300] GBROWSE*******************

 CG13577 in-situ | CG13577 | - | -1824 | -3102 | UPSTREAM | CG13577-RA | "-"
insitu CG3385 in-situ | nvy | + | 4955 | 20306 | UPSTREAM | CG3385-RA | "-"

*********************** Rank 89 [Score 10.288800] GBROWSE*******************

insitu CG32369 in-situ | CG32369 | - | -13915 | -39912 | UPSTREAM | CG32369-RA | "-" | CG32369-RB | "-"
 CG17888 in-situ | Pdp1 | - | 49051 | -3986 | INTRAGENIC | intron:CG17888-RF:1 | intron:CG17888-RB:2 | intron:CG17888-RG:3 | intron:CG17888-RA:1 | intron:CG17888-RE:2 | intron:CG17888-RD:3 | intron:CG17888-RH:1 | intron:CG17888-RC:1 | CG17888-RF | "-" | CG17888-RB | "-" | CG17888-RG | "-" | CG17888-RA | "-" | CG17888-RE | "-" | CG17888-RD | "-" | CG17888-RH | "-" | CG17888-RC | "-"

*********************** Rank 90 [Score 10.225300] GBROWSE*******************

insitu CG9986 in-situ | CG9986 | + | -11125 | -8888 | DOWNSTREAM | CG9986-RA | "-"
 CG10011 in-situ | CG10011 | - | 17801 | -9331 | INTRAGENIC | intron:CG10011-RA:1 | CG10011-RA | "-"

*********************** Rank 91 [Score 10.214500] GBROWSE*******************

 CG6486 in-situ | CG6486 | + | -4116 | -2924 | DOWNSTREAM | CG6486-RA | "-"
insitu highlight CG6494 in-situ | h | + | 22319 | 25599 | UPSTREAM | CG6494-RA | "-"


*********************** Rank 92 [Score 10.209400] GBROWSE*******************

 CG4928 in-situ | BcDNA:GH10120 | + | -4581 | 19632 | INTRAGENIC | intron:CG4928-RA:1 | CG4928-RA | "-" | CG4928-RB | "-"
 CG9089 in-situ | wus | - | 23047 | 20718 | DOWNSTREAM | CG9089-RA | "-"

*********************** Rank 93 [Score 10.206900] GBROWSE*******************

 CG12014 in-situ | CG12014 | + | -22486 | -20687 | DOWNSTREAM | CG12014-RA | "-"
 CG1921 in-situ | sty | - | 24164 | 366 | DOWNSTREAM | CG1921-RC | "-" | CG1921-RB | "-"

*********************** Rank 94 [Score 10.199000] GBROWSE*******************

 CG10231 in-situ | CG10231 | - | -7845 | -38289 | UPSTREAM | CG10231-RA | "-"
 CG15160 in-situ | CG15160 | + | 19880 | 24414 | UPSTREAM | CG15160-RA | "-"

*********************** Rank 95 [Score 10.182400] GBROWSE*******************

 CG12170 in-situ | CG12170 | + | -7544 | -6124 | DOWNSTREAM | CG12170-RA | "-"
 CG31545 in-situ | CG31545 | - | 3129 | 2496 | DOWNSTREAM | CG31545-RA | "-"

*********************** Rank 96 [Score 10.175200] GBROWSE*******************

 CG14362 in-situ | CG14362 | - | -4310 | -5098 | UPSTREAM | CG14362-RA | "-"
 CG9930 in-situ | E5 | - | 14420 | 7739 | DOWNSTREAM | CG9930-RA | "-"

*********************** Rank 97 [Score 10.167600] GBROWSE*******************

 CG1451 in-situ | Apc | - | -5862 | -18212 | UPSTREAM | CG1451-RA | "-"
 CG31048 in-situ | CG31048 | + | 2195 | 19106 | UPSTREAM | CG31048-RA | "-"

*********************** Rank 98 [Score 10.166300] GBROWSE*******************

 CG8355 in-situ | sli | - | -12771 | -30954 | UPSTREAM | CG8355-RA | "-" | CG8355-RC | "-" | CG8355-RB | "-"
 CG8291 in-situ | CG8291 | - | 28304 | 19415 | DOWNSTREAM | CG8291-RA | "-" | CG8291-RC | "-" | CG8291-RB | "-"

*********************** Rank 99 [Score 10.159100] GBROWSE*******************

 CG32970 in-situ | CG32970 | + | -12741 | -9900 | DOWNSTREAM | CG32970-RA | "-"
 CG15287 in-situ | BG:DS01068.1 | + | 7531 | 12969 | UPSTREAM | CG15287-RA | "-"

*********************** Rank 100 [Score 10.157300] GBROWSE*******************

 CG10954 in-situ | Arc-p34 | + | -1697 | 4276 | INTRAGENIC | intron:CG10954-RA:1 | CG10954-RA | "-"
 CG15130 in-situ | CG15130 | - | 5841 | 4427 | DOWNSTREAM | CG15130-RA | "-"

*********************** Rank 101 [Score 10.156700] GBROWSE*******************

 CG33158 in-situ | CG33158 | + | -29727 | 47049 | INTRAGENIC | intron:CG33158-RB:3 | CG33158-RB | "-"
insitu CG32159 in-situ | CG32159 | - | 2433 | -26727 | INTRAGENIC | intron:CG32159-RB:1 | CG32159-RB | "-"

*********************** Rank 102 [Score 10.156400] GBROWSE*******************

 CG31957 in-situ | CG31957 | - | -16745 | -17618 | UPSTREAM | CG31957-RA | "-"
insitu CG3399 in-situ | capu | - | 13729 | -16473 | INTRAGENIC | intron:CG3399-RC:2 | intron:CG3399-RD:3 | intron:CG3399-RB:2 | CG3399-RC | "-" | CG3399-RD | "-" | CG3399-RB | "-" | CG3399-RA | "-"

*********************** Rank 103 [Score 10.141500] GBROWSE*******************

 CG3827 in-situ | sc | + | -5352 | -3931 | DOWNSTREAM | CG3827-RA | "-"
insitu highlight CG3839 in-situ | l(1)sc | + | 8311 | 9407 | UPSTREAM | CG3839-RA | "-"

*********************** Rank 104 [Score 10.141300] GBROWSE*******************

insitu CG3619 in-situ | Dl | - | -13848 | -37330 | UPSTREAM | CG3619-RA | "-" | CG3619-RB | "-"
 CG3581 in-situ | CG3581 | - | 34688 | 33696 | DOWNSTREAM | CG3581-RA | "-"


*********************** Rank 105 [Score 10.139100] GBROWSE*******************

 CG11328 in-situ | Nhe3 | - | -27 | -6541 | UPSTREAM | CG11328-RA | "-" | CG11328-RB | "-"
 CG11327 in-situ | CG11327 | - | 2611 | 461 | DOWNSTREAM | CG11327-RB | "-" | CG11327-RA | "-"

*********************** Rank 106 [Score 10.118300] GBROWSE*******************

insitu CG32687 in-situ | CG32687 | - | -2213 | -6812 | UPSTREAM | CG32687-RA | "-"
 CG32680 in-situ | CG32680 | - | 4897 | 3622 | DOWNSTREAM | CG32680-RB | "-"


*********************** Rank 107 [Score 10.111500] GBROWSE*******************

 CG2750 in-situ | CG2750 | + | -15490 | -9436 | DOWNSTREAM | CG2750-RA | "-"
 CG1924 in-situ | CG1924 | - | 55917 | 54205 | DOWNSTREAM | CG1924-RA | "-"

*********************** Rank 108 [Score 10.091900] GBROWSE*******************

 CG15541 in-situ | CG15541 | + | -22945 | -16401 | DOWNSTREAM | CG15541-RA | "-"
 CG1342 in-situ | CG1342 | + | 18532 | 20656 | UPSTREAM | CG1342-RA | "-"

*********************** Rank 109 [Score 10.068800] GBROWSE*******************

 CG3811 in-situ | CG3811 | + | -6556 | 12274 | INTRAGENIC | intron:CG3811-RA:2 | intron:CG3811-RB:2 | CG3811-RA | "-" | CG3811-RB | "-"
 CG31883 in-situ | CG31883 | + | 752 | 1877 | UPSTREAM | CG31883-RA | "-"


*********************** Rank 110 [Score 10.063800] GBROWSE*******************

 CG3347 in-situ | CG3347 | + | -3129 | 8765 | INTRAGENIC | intron:CG3347-RA:1 | CG3347-RA | "-"
 CG3332 in-situ | CG3332 | - | 17602 | 12863 | DOWNSTREAM | CG3332-RB | "-" | CG3332-RA | "-"

*********************** Rank 111 [Score 10.009300] GBROWSE*******************

 CG14280 in-situ | CG14280 | + | -24095 | -21479 | DOWNSTREAM | CG14280-RA | "-"
insitu CG3619 in-situ | Dl | - | 44852 | 21370 | DOWNSTREAM | CG3619-RA | "-" | CG3619-RB | "-"

*********************** Rank 112 [Score  9.994700] GBROWSE*******************

 CG31934 in-situ | CG31934 | - | -33519 | -34203 | UPSTREAM | CG31934-RA | "-"
 CG17158 in-situ | cpb | + | 69 | 1944 | UPSTREAM | CG17158-RA | "-"

*********************** Rank 113 [Score  9.985100] GBROWSE*******************

insitu CG16786 in-situ | CG16786 | + | -4800 | -372 | DOWNSTREAM | CG16786-RA | "-" | CG16786-RB | "-"
 CG3231 in-situ | BcDNA:LD21643 | + | 74 | 5487 | UPSTREAM | CG3231-RA | "-"

*********************** Rank 114 [Score  9.976900] GBROWSE*******************

 CG18106 in-situ | IM2 | + | -1133 | -695 | DOWNSTREAM | CG18106-RA | "-"
 CG16844 in-situ | CG16844 | + | 390 | 777 | UPSTREAM | CG16844-RA | "-"

*********************** Rank 115 [Score  9.957900] GBROWSE*******************

insitu CG3619 in-situ | Dl | - | -8598 | -32080 | UPSTREAM | CG3619-RA | "-" | CG3619-RB | "-"
 CG3581 in-situ | CG3581 | - | 39938 | 38946 | DOWNSTREAM | CG3581-RA | "-"

*********************** Rank 116 [Score  9.957900] GBROWSE*******************

 CG13826 in-situ | CG13826 | - | -9313 | -13857 | UPSTREAM | CG13826-RA | "-"
 CG4467 in-situ | CG4467 | - | 4028 | -5960 | INTRAGENIC | intron:CG4467-RA:3 | CG4467-RA | "-"

*********************** Rank 117 [Score  9.945500] GBROWSE*******************

insitu highlight CG3851 in-situ | odd | - | -4233 | -6759 | UPSTREAM | CG3851-RA | "-"
 CG2788 in-situ | Dot | + | 8311 | 10190 | UPSTREAM | CG2788-RA | "-"

*********************** Rank 118 [Score  9.944500] GBROWSE*******************

insitu CG5799 in-situ | dve | + | -19586 | 22869 | INTRAGENIC | intron:CG5799-RA:2 | intron:CG5799-RD:2 | intron:CG5799-RC:2 | CG5799-RA | "-" | CG5799-RD | "-" | CG5799-RB | "-" | CG5799-RC | "-"
insitu CG5819 in-situ | CG5819 | + | 29910 | 33409 | UPSTREAM | CG5819-RA | "-" | CG5819-RB | "-"

*********************** Rank 119 [Score  9.939600] GBROWSE*******************

 CG3953 in-situ | l(3)IX-14 | + | -4911 | 8699 | INTRAGENIC | intron:CG3953-RA:1 | CG3953-RA | "-"
 CG6254 in-situ | CG6254 | - | 11718 | 9526 | DOWNSTREAM | CG6254-RA | "-"


*********************** Rank 120 [Score  9.914400] GBROWSE*******************

 CG10093 in-situ | Cyp313a3 | - | -8500 | -10330 | UPSTREAM | CG10093-RA | "-"
 CG3942 in-situ | CG3942 | + | 6578 | 8983 | UPSTREAM | CG3942-RA | "-"

*********************** Rank 121 [Score  9.904900] GBROWSE*******************

 CG12501 in-situ | Or56a | - | -10204 | -11976 | UPSTREAM | CG12501-RA | "-"
 CG13873 in-situ | Obp56g | - | 2583 | 2120 | DOWNSTREAM | CG13873-RA | "-"

*********************** Rank 122 [Score  9.902200] GBROWSE*******************

 CG14351 in-situ | CG14351 | + | -20875 | 31583 | INTRAGENIC | intron:CG14351-RA:2 | CG14351-RA | "-"
 CG10869 in-situ | CG10869 | - | 11236 | 8824 | DOWNSTREAM | CG10869-RA | "-"


*********************** Rank 123 [Score  9.895800] GBROWSE*******************

 CG6789 in-situ | CG6789 | + | -22588 | -21497 | DOWNSTREAM | CG6789-RA | "-"
 CG15465 in-situ | CG15465 | + | 20754 | 22022 | UPSTREAM | CG15465-RA | "-"

*********************** Rank 124 [Score  9.890600] GBROWSE*******************

 CG7906 in-situ | CG7906 | + | -23846 | -22298 | DOWNSTREAM | CG7906-RA | "-"
 CG17697 in-situ | fz | + | 13777 | 108078 | UPSTREAM | CG17697-RB | "-" | CG17697-RA | "-"

*********************** Rank 125 [Score  9.889200] GBROWSE*******************

 CG6747 in-situ | Ir | + | -12701 | -10062 | DOWNSTREAM | CG6747-RA | "-"
 CG4568 in-situ | fzo | - | 8413 | 6015 | DOWNSTREAM | CG4568-RA | "-"

*********************** Rank 126 [Score  9.885600] GBROWSE*******************

 CG32030 in-situ | CG32030 | + | -11846 | 20435 | INTRAGENIC | intron:CG32030-RA:2 | intron:CG32030-RB:2 | CG32030-RA | "-" | CG32030-RB | "-"
insitu CG5804 in-situ | CG5804 | - | 10250 | 9852 | DOWNSTREAM | CG5804-RA | "-"

*********************** Rank 127 [Score  9.859800] GBROWSE*******************

 CG32115 in-situ | CG32115 | - | -33457 | -35063 | UPSTREAM | CG32115-RA | "-"
 CG10752 in-situ | CG10752 | - | 7459 | 5580 | DOWNSTREAM | CG10752-RA | "-"

*********************** Rank 128 [Score  9.843000] GBROWSE*******************

 CG14477 in-situ | mm | + | -17031 | 7744 | INTRAGENIC | intron:CG14477-RA:4 | CG14477-RA | "-"
 CG10939 in-situ | Sip1 | + | 26471 | 37048 | UPSTREAM | CG10939-RA | "-"

*********************** Rank 129 [Score  9.839500] GBROWSE*******************

insitu CG31632 in-situ | CG31632 | + | -25367 | -8679 | DOWNSTREAM | CG31632-RA | "-"
 CG10800 in-situ | Rca1 | + | 4640 | 6192 | UPSTREAM | CG10800-RA | "-"

*********************** Rank 130 [Score  9.837400] GBROWSE*******************

 CG8817 in-situ | lilli | + | -12179 | 54946 | INTRAGENIC | intron:CG8817-RA:2 | intron:CG8817-RB:2 | intron:CG8817-RC:2 | CG8817-RA | "-" | CG8817-RB | "-" | CG8817-RC | "-"
 CG3151 in-situ | Rbp9 | + | 56631 | 66722 | UPSTREAM | CG3151-RA | "-" | CG3151-RD | "-" | CG3151-RB | "-" | CG3151-RE | "-" | CG3151-RC | "-" | CG3151-RF | "-"


*********************** Rank 131 [Score  9.814100] GBROWSE*******************

insitu highlight CG4345 in-situ | grim | - | -32957 | -34652 | UPSTREAM | CG4345-RA | "-"
 CG4319 in-situ | rpr | - | 61032 | 60182 | DOWNSTREAM | CG4319-RA | "-"

*********************** Rank 132 [Score  9.804100] GBROWSE*******************

insitu highlight CG1225 in-situ | RhoGEF3 | + | -15098 | -7626 | DOWNSTREAM | CG1225-RA | "-" | CG1225-RB | "-" | CG1225-RC | "-" | CG1225-RE | "-"
 CG7004 in-situ | fwd | - | 6960 | -6713 | INTRAGENIC | intron:CG7004-RB:2 | intron:CG7004-RA:2 | CG7004-RB | "-" | CG7004-RA | "-" | CG7004-RC | "-"

*********************** Rank 133 [Score  9.772600] GBROWSE*******************

 CG2595 in-situ | RacGAP84C | + | -14526 | -11827 | DOWNSTREAM | CG2595-RA | "-" | CG2595-RB | "-"
 CG32467 in-situ | CG32467 | - | 2713 | 2258 | DOWNSTREAM | CG32467-RA | "-"


*********************** Rank 134 [Score  9.755400] GBROWSE*******************

 CG16898 in-situ | CG16898 | - | -40151 | -41519 | UPSTREAM | CG16898-RA | "-"
 CG8896 in-situ | 18w | + | 66373 | 71794 | UPSTREAM | CG8896-RA | "-"

*********************** Rank 135 [Score  9.743800] GBROWSE*******************

 CG15147 in-situ | CG15147 | - | -34072 | -34670 | UPSTREAM | CG15147-RA | "-"
 CG7100 in-situ | CadN | - | 79104 | -10551 | INTRAGENIC | intron:CG7100-RA:15 | intron:CG7100-RC:15 | intron:CG7100-RD:15 | intron:CG7100-RE:15 | intron:CG7100-RF:15 | intron:CG7100-RG:15 | intron:CG7100-RH:15 | intron:CG7100-RB:15 | CG7100-RA | "-" | CG7100-RC | "-" | CG7100-RD | "-" | CG7100-RE | "-" | CG7100-RF | "-" | CG7100-RG | "-" | CG7100-RH | "-" | CG7100-RB | "-"

*********************** Rank 136 [Score  9.731900] GBROWSE*******************

insitu CG32604 in-situ | CG32604 | + | -7169 | 16187 | INTRAGENIC | intron:CG32604-RA:2 | CG32604-RA | "-" | CG32604-RB | "-"
 CG11674 in-situ | CG11674 | - | 17343 | 16102 | DOWNSTREAM | CG11674-RA | "-"

*********************** Rank 137 [Score  9.725500] GBROWSE*******************

 CG12754 in-situ | Or42b | - | -20017 | -21334 | UPSTREAM | CG12754-RA | "-"
 CG7796 in-situ | CG7796 | + | 28432 | 34812 | UPSTREAM | CG7796-RA | "-"

*********************** Rank 138 [Score  9.714500] GBROWSE*******************

 CG2961 in-situ | CG2961 | - | -15497 | -16898 | UPSTREAM | CG2961-RA | "-"
 CG32688 in-situ | Hk | - | 6412 | -9648 | INTRAGENIC | intron:CG32688-RB:1 | CG32688-RB | "-" | CG32688-RA | "-"

*********************** Rank 139 [Score  9.702100] GBROWSE*******************

 CG13480 in-situ | Leucokinin | - | -2656 | -3286 | UPSTREAM | CG13480-RA | "-"
 CG13479 in-situ | CG13479 | - | 4710 | 4361 | DOWNSTREAM | CG13479-RA | "-"

*********************** Rank 140 [Score  9.671000] GBROWSE*******************

 CG4641 in-situ | CG4641 | + | -19909 | -16981 | DOWNSTREAM | CG4641-RA | "-"
 CG10537 in-situ | Rdl | - | 14914 | -12095 | INTRAGENIC | intron:CG10537-RA:4 | intron:CG10537-RB:4 | intron:CG10537-RC:4 | CG10537-RA | "-" | CG10537-RB | "-" | CG10537-RC | "-"

*********************** Rank 141 [Score  9.669300] GBROWSE*******************

 CG7131 in-situ | CG7131 | - | -4135 | -6297 | UPSTREAM | CG7131-RA | "-"
 CG7129 in-situ | l(3)05822 | - | 5972 | -4055 | INTRAGENIC | intron:CG7129-RA:1 | CG7129-RA | "-" | CG7129-RB | "-"

*********************** Rank 142 [Score  9.655700] GBROWSE*******************

insitu highlight CG1759 in-situ | cad | + | -14122 | -1716 | DOWNSTREAM | CG1759-RA | "-" | CG1759-RB | "-"
 CG9324 in-situ | CG9324 | + | 2791 | 4242 | UPSTREAM | CG9324-RA | "-"

*********************** Rank 143 [Score  9.654000] GBROWSE*******************

 CG9147 in-situ | CG9147 | + | -1649 | -207 | DOWNSTREAM | CG9147-RB | "-" | CG9147-RA | "-"
 CG9150 in-situ | CG9150 | - | 744 | 4 | DOWNSTREAM | CG9150-RA | "-"

*********************** Rank 144 [Score  9.648600] GBROWSE*******************

insitu highlight CG6889 in-situ | tara | + | -4361 | 30292 | INTRAGENIC | intron:CG6889-RA:1 | CG6889-RA | "-" | CG6889-RB | "-"
 CG6815 in-situ | bor | - | 34078 | 31340 | DOWNSTREAM | CG6815-RA | "-"

*********************** Rank 145 [Score  9.642300] GBROWSE*******************

 CG5481 in-situ | lea | - | -42685 | -82246 | UPSTREAM | CG5481-RA | "-"
 CG31925 in-situ | CG31925 | - | 10909 | 10216 | DOWNSTREAM | CG31925-RA | "-"

*********************** Rank 146 [Score  9.642000] GBROWSE*******************

 CG5549 in-situ | CG5549 | + | -3186 | 4158 | INTRAGENIC | intron:CG5549-RA:2 | CG5549-RA | "-"
insitu CG4735 in-situ | shu | - | 10097 | 8275 | DOWNSTREAM | CG4735-RA | "-"

*********************** Rank 147 [Score  9.615700] GBROWSE*******************

 CG14063 in-situ | CG14063 | + | -13849 | -13230 | DOWNSTREAM | CG14063-RA | "-"
insitu highlight CG10002 in-situ | fkh | - | 16871 | 13604 | DOWNSTREAM | CG10002-RA | "-"

*********************** Rank 148 [Score  9.598100] GBROWSE*******************

 CG32150 in-situ | CG32150 | - | -763 | -7057 | UPSTREAM | CG32150-RB | "-" | CG32150-RA | "-"
 CG12486 in-situ | CG12486 | - | 11556 | 10861 | DOWNSTREAM | CG12486-RA | "-"

*********************** Rank 149 [Score  9.593900] GBROWSE*******************

 CG12632 in-situ | fd3F | + | -9732 | -8625 | DOWNSTREAM | CG12632-RB | "-"
 CG2901 in-situ | CG2901 | + | 9168 | 11746 | UPSTREAM | CG2901-RA | "-"

*********************** Rank 150 [Score  9.579500] GBROWSE*******************

 CG15596 in-situ | CG15596 | - | -8790 | -9698 | UPSTREAM | CG15596-RA | "-"
 CG1154 in-situ | CG1154 | + | 1053 | 5017 | UPSTREAM | CG1154-RA | "-"

*********************** Rank 151 [Score  9.579300] GBROWSE*******************

 CG8301 in-situ | CG8301 | + | -3850 | 1489 | INTRAGENIC | intron:CG8301-RA:3 | CG8301-RA | "-"
 CG8286 in-situ | CG8286 | + | 1777 | 4418 | UPSTREAM | CG8286-RA | "-"

*********************** Rank 152 [Score  9.574800] GBROWSE*******************

 CG15631 in-situ | CG15631 | - | -21973 | -24022 | UPSTREAM | CG15631-RA | "-"
 CG15630 in-situ | CG15630 | - | 37611 | -20884 | INTRAGENIC | intron:CG15630-RA:1 | CG15630-RA | "-"

*********************** Rank 153 [Score  9.565700] GBROWSE*******************

insitu CG9468 in-situ | CG9468 | - | -27268 | -30859 | UPSTREAM | CG9468-RA | "-"
insitu highlight CG18024 in-situ | SoxN | + | 17106 | 21151 | UPSTREAM | CG18024-RA | "-"

*********************** Rank 154 [Score  9.563200] GBROWSE*******************

 CG2865 in-situ | EG:25E8.4 | - | -15432 | -26666 | UPSTREAM | CG2865-RA | "-"
 CG14050 in-situ | EG:BACH48C10.1 | - | 17512 | 16648 | DOWNSTREAM | CG14050-RA | "-"


*********************** Rank 155 [Score  9.561500] GBROWSE*******************

 CG9266 in-situ | CG9266 | - | -57165 | -60589 | UPSTREAM | CG9266-RB | "-"
insitu CG1762 in-situ | betaInt-nu | + | 26726 | 31938 | UPSTREAM | CG1762-RA | "-"


*********************** Rank 156 [Score  9.560100] GBROWSE*******************

 CG5870 in-situ | beta-Spec | + | -1720 | 10096 | INTRAGENIC | intron:CG5870-RA:1 | CG5870-RA | "-"
 CG12990 in-situ | CG12990 | - | 12747 | 10067 | DOWNSTREAM | CG12990-RA | "-"

*********************** Rank 157 [Score  9.535000] GBROWSE*******************

 CG32827 in-situ | CG32827 | + | -55255 | -51256 | DOWNSTREAM | CG32827-RA | "-"
 CG12200 in-situ | CG12200 | + | 7287 | 8214 | UPSTREAM | CG12200-RA | "-"

*********************** Rank 158 [Score  9.522900] GBROWSE*******************

 CG15214 in-situ | CG15214 | + | -38846 | -34850 | DOWNSTREAM | CG15214-RA | "-"
 CG4835 in-situ | CG4835 | + | 5694 | 9221 | UPSTREAM | CG4835-RA | "-"


*********************** Rank 159 [Score  9.521900] GBROWSE*******************

 CG5423 in-situ | robo3 | + | -27008 | 10001 | INTRAGENIC | intron:CG5423-RA:3 | CG5423-RA | "-"
 CG5430 in-situ | a5 | + | 45327 | 46070 | UPSTREAM | CG5430-RA | "-"


*********************** Rank 160 [Score  9.506600] GBROWSE*******************

 CG1088 in-situ | Vha26 | + | -500 | 2476 | INTRAGENIC | intron:CG1088-RB:2 | intron:CG1088-RA:1 | CG1088-RB | "-" | CG1088-RA | "-"
 CG2922 in-situ | eIF-5C | - | 8938 | 3133 | DOWNSTREAM | CG2922-RA | "-" | CG2922-RG | "-" | CG2922-RC | "-" | CG2922-RF | "-" | CG2922-RD | "-" | CG2922-RB | "-" | CG2922-RE | "-"

*********************** Rank 161 [Score  9.470300] GBROWSE*******************

 CG3355 in-situ | CG3355 | + | -19228 | -17754 | DOWNSTREAM | CG3355-RA | "-"
 CG11929 in-situ | CG11929 | + | 12046 | 16604 | UPSTREAM | CG11929-RA | "-"

*********************** Rank 162 [Score  9.461900] GBROWSE*******************

 CG13539 in-situ | CG13539 | + | -24274 | -23394 | DOWNSTREAM | CG13539-RA | "-"
 CG3162 in-situ | CG3162 | - | 2857 | 1443 | DOWNSTREAM | CG3162-RA | "-"


*********************** Rank 163 [Score  9.454500] GBROWSE*******************

 CG32431 in-situ | CG32431 | + | -6870 | -6163 | DOWNSTREAM | CG32431-RA | "-"
insitu highlight CG4717 in-situ | kni | - | 751 | -2282 | INTRAGENIC | intron:CG4717-RA:1 | CG4717-RA | "-"


*********************** Rank 164 [Score  9.449200] GBROWSE*******************

insitu CG32194 in-situ | CG32194 | - | -16563 | -18317 | UPSTREAM | CG32194-RB | "-"
 CG13698 in-situ | CG13698 | - | 2509 | -14512 | INTRAGENIC | intron:CG13698-RB:1 | CG13698-RB | "-"

*********************** Rank 165 [Score  9.433300] GBROWSE*******************

insitu highlight CG18455 in-situ | Optix | + | -22070 | -11167 | DOWNSTREAM | CG18455-RA | "-" | CG18455-RB | "-"
 CG12769 in-situ | CG12769 | - | 11848 | 123 | DOWNSTREAM | CG12769-RB | "-" | CG12769-RA | "-"


*********************** Rank 166 [Score  9.431600] GBROWSE*******************

 CG6976 in-situ | Myo28B1 | + | -2822 | 20592 | INTRAGENIC | intron:CG6976-RB:1 | intron:CG6976-RC:1 | CG6976-RB | "-" | CG6976-RC | "-" | CG6976-RA | "-" | CG6976-RD | "-"
 CG6772 in-situ | Slob | - | 6209 | -21834 | INTRAGENIC | intron:CG6772-RB:2 | CG6772-RB | "-" | CG6772-RA | "-" | CG6772-RC | "-"

*********************** Rank 167 [Score  9.421000] GBROWSE*******************

 CG16757 in-situ | Spn | - | -29894 | -76051 | UPSTREAM | CG16757-RA | "-"
 CG16973 in-situ | msn | - | 2369 | -28397 | INTRAGENIC | intron:CG16973-RA:2 | CG16973-RA | "-"

*********************** Rank 168 [Score  9.414200] GBROWSE*******************

 CG32108 in-situ | CG32108 | + | -5607 | -3603 | DOWNSTREAM | CG32108-RA | "-"
insitu CG10960 in-situ | CG10960 | - | 14852 | -3371 | INTRAGENIC | intron:CG10960-RC:2 | intron:CG10960-RB:1 | CG10960-RC | "-" | CG10960-RB | "-" | CG10960-RA | "-"


*********************** Rank 169 [Score  9.410800] GBROWSE*******************

 CG32042 in-situ | PGRP-LA | + | -5707 | -2102 | DOWNSTREAM | CG32042-RB | "-" | CG32042-RA | "-"
 CG4432 in-situ | PGRP-LC | + | 414 | 8296 | UPSTREAM | CG4432-RA | "-" | CG4432-RB | "-"

*********************** Rank 170 [Score  9.406800] GBROWSE*******************

 CG6717 in-situ | CG6717 | + | -4134 | -2836 | DOWNSTREAM | CG6717-RA | "-"
 CG6730 in-situ | Cyp4d21 | + | 19531 | 21634 | UPSTREAM | CG6730-RA | "-"

*********************** Rank 171 [Score  9.399100] GBROWSE*******************

 CG9400 in-situ | CG9400 | - | -8013 | -9302 | UPSTREAM | CG9400-RA | "-"
 CG18157 in-situ | CG18157 | - | 18026 | 17433 | DOWNSTREAM | CG18157-RA | "-"

*********************** Rank 172 [Score  9.396800] GBROWSE*******************

 CG8051 in-situ | CG8051 | - | -9866 | -16469 | UPSTREAM | CG8051-RA | "-"
 CG8062 in-situ | BcDNA:LD28120 | - | 4275 | -6762 | INTRAGENIC | intron:CG8062-RA:1 | CG8062-RA | "-"

*********************** Rank 173 [Score  9.386000] GBROWSE*******************

 CG16726 in-situ | CG16726 | + | -1035 | 113 | INTRAGENIC | intron:CG16726-RA:1 | CG16726-RA | "-"
 CG32047 in-situ | CG32047 | + | 3919 | 8167 | UPSTREAM | CG32047-RA | "-"


*********************** Rank 174 [Score  9.385400] GBROWSE*******************

 CG10862 in-situ | CG10862 | - | -6800 | -8106 | UPSTREAM | CG10862-RA | "-"
 CG10858 in-situ | CG10858 | - | 93630 | 86954 | DOWNSTREAM | CG10858-RA | "-"

*********************** Rank 175 [Score  9.384300] GBROWSE*******************

 CG4262 in-situ | elav | - | -1642 | -7638 | UPSTREAM | CG4262-RA | "-"
 CG4293 in-situ | EG:65F1.1 | - | 4990 | 3075 | DOWNSTREAM | CG4293-RA | "-" | CG4293-RB | "-"

*********************** Rank 176 [Score  9.377200] GBROWSE*******************

 CG13055 in-situ | CG13055 | - | -2322 | -3251 | UPSTREAM | CG13055-RA | "-"
 CG13054 in-situ | CG13054 | - | 2664 | 1720 | DOWNSTREAM | CG13054-RA | "-"

*********************** Rank 177 [Score  9.373700] GBROWSE*******************

 CG10184 in-situ | CG10184 | - | -10534 | -13519 | UPSTREAM | CG10184-RA | "-"
 CG31145 in-situ | CG31145 | - | 53635 | -9285 | INTRAGENIC | intron:CG31145-RA:7 | intron:CG31145-RB:7 | CG31145-RA | "-" | CG31145-RB | "-"

*********************** Rank 178 [Score  9.372100] GBROWSE*******************

 CG1470 in-situ | Gycbeta100B | + | -26043 | 11008 | INTRAGENIC | intron:CG1470-RA:16 | CG1470-RA | "-"
 CG31006 in-situ | CG31006 | - | 8591 | -2357 | INTRAGENIC | intron:CG31006-RB:8 | intron:CG31006-RA:7 | CG31006-RB | "-" | CG31006-RA | "-"

*********************** Rank 179 [Score  9.369000] GBROWSE*******************

insitu highlight CG6494 in-situ | h | + | -11881 | -8601 | DOWNSTREAM | CG6494-RA | "-"
 CG33162 in-situ | SrpRbeta | - | 32377 | 31427 | DOWNSTREAM | CG33162-RA | "-"

*********************** Rank 180 [Score  9.356700] GBROWSE*******************

 CG14538 in-situ | CG14538 | + | -7602 | -6985 | DOWNSTREAM | CG14538-RA | "-"
 CG7191 in-situ | CG7191 | + | 3730 | 11114 | UPSTREAM | CG7191-RA | "-"

*********************** Rank 181 [Score  9.354900] GBROWSE*******************

 CG31330 in-situ | CG31330 | - | -55280 | -61494 | UPSTREAM | CG31330-RA | "-"
 CG8464 in-situ | CG8464 | - | 23232 | 21434 | DOWNSTREAM | CG8464-RA | "-"

*********************** Rank 182 [Score  9.353500] GBROWSE*******************

 CG8680 in-situ | CG8680 | - | -701 | -1385 | UPSTREAM | CG8680-RA | "-"
 CG14035 in-situ | CG14035 | + | 1919 | 3195 | UPSTREAM | CG14035-RA | "-"

*********************** Rank 183 [Score  9.343900] GBROWSE*******************

 CG9570 in-situ | CG9570 | + | -24597 | -23369 | DOWNSTREAM | CG9570-RA | "-"
 CG9571 in-situ | CG9571 | - | 16494 | 15712 | DOWNSTREAM | CG9571-RA | "-"

*********************** Rank 184 [Score  9.342400] GBROWSE*******************

 CG32629 in-situ | CG32629 | + | -2467 | 9810 | INTRAGENIC | intron:CG32629-RA:1 | CG32629-RA | "-"
 CG15758 in-situ | CG15758 | + | 9917 | 10764 | UPSTREAM | CG15758-RB | "-"

*********************** Rank 185 [Score  9.341500] GBROWSE*******************

insitu CG12214 in-situ | CG12214 | - | -33937 | -36447 | UPSTREAM | CG12214-RB | "-" | CG12214-RA | "-"
insitu CG33183 in-situ | Hr46 | - | 40574 | 12619 | DOWNSTREAM | CG33183-RC | "-" | CG33183-RB | "-" | CG33183-RA | "-"

*********************** Rank 186 [Score  9.338900] GBROWSE*******************

 CG11192 in-situ | CG11192 | - | -13215 | -14024 | UPSTREAM | CG11192-RA | "-"
 CG33041 in-situ | CG33041 | + | 56097 | 102726 | UPSTREAM | CG33041-RA | "-"

*********************** Rank 187 [Score  9.336000] GBROWSE*******************

 CG30431 in-situ | CG30431 | + | -2842 | -382 | DOWNSTREAM | CG30431-RA | "-"
 CG17994 in-situ | CG17994 | - | 3723 | 1633 | DOWNSTREAM | CG17994-RA | "-"

*********************** Rank 188 [Score  9.330200] GBROWSE*******************

 CG32465 in-situ | CG32465 | - | -8594 | -18569 | UPSTREAM | CG32465-RB | "-"
 CG14597 in-situ | CG14597 | - | 13882 | 13241 | DOWNSTREAM | CG14597-RA | "-"

*********************** Rank 189 [Score  9.326400] GBROWSE*******************

 CG7574 in-situ | bip1 | - | -6150 | -10510 | UPSTREAM | CG7574-RA | "-"
 CG13681 in-situ | CG13681 | + | 6960 | 7472 | UPSTREAM | CG13681-RA | "-"

*********************** Rank 190 [Score  9.325400] GBROWSE*******************

 CG8317 in-situ | CG8317 | - | -1443 | -3447 | UPSTREAM | CG8317-RA | "-"
 CG5072 in-situ | Cdk4 | + | 1451 | 7423 | UPSTREAM | CG5072-RC | "-" | CG5072-RA | "-" | CG5072-RB | "-"

*********************** Rank 191 [Score  9.319400] GBROWSE*******************

 CG11007 in-situ | CG11007 | + | -4551 | -3362 | DOWNSTREAM | CG11007-RA | "-"
 CG15121 in-situ | CG15121 | + | 537 | 1814 | UPSTREAM | CG15121-RA | "-"

*********************** Rank 192 [Score  9.308000] GBROWSE*******************

 CG16983 in-situ | skpA | + | -6991 | -5159 | DOWNSTREAM | CG16983-RA | "-" | CG16983-RD | "-" | CG16983-RG | "-" | CG16983-RB | "-" | CG16983-RC | "-" | CG16983-RF | "-" | CG16983-RE | "-"
 CG5227 in-situ | sdk | + | 23777 | 86435 | UPSTREAM | CG5227-RD | "-" | CG5227-RC | "-" | CG5227-RB | "-" | CG5227-RA | "-"

*********************** Rank 193 [Score  9.304800] GBROWSE*******************

 CG17280 in-situ | CG17280 | + | -2442 | -1700 | DOWNSTREAM | CG17280-RA | "-"
insitu CG5370 in-situ | Dcp-1 | + | 633 | 2874 | UPSTREAM | CG5370-RA | "-"

*********************** Rank 194 [Score  9.302300] GBROWSE*******************

 CG6486 in-situ | CG6486 | + | -21066 | -19874 | DOWNSTREAM | CG6486-RA | "-"
insitu highlight CG6494 in-situ | h | + | 5369 | 8649 | UPSTREAM | CG6494-RA | "-"

note: overlaps known module h_rescue by 500 bases (module coords: 8620516-8642090)
note: overlaps known module h_stripe1_1990 by 447 bases (module coords: 8629353-8630638)
note: overlaps known module h_stripe1_1991 by 165 bases (module coords: 8629635-8630510)
note: overlaps known module h_stripe2_1991 by 500 bases (module coords: 8625021-8630510)
note: overlaps known module h_stripe5_1990a by 500 bases (module coords: 8627668-8630638)
note: overlaps known module h_stripe5_1990b by 54 bases (module coords: 8627680-8629353)
note: overlaps known module h_stripe1_5_1991 by 500 bases (module coords: 8627680-8630510)
note: overlaps known module h_stripe5_1993 by 54 bases (module coords: 8628790-8629353)

*********************** Rank 195 [Score  9.290900] GBROWSE*******************

 CG30128 in-situ | Obp56c | - | -1483 | -2357 | UPSTREAM | CG30128-RA | "-"
 CG11218 in-situ | Obp56d | - | 1265 | 600 | DOWNSTREAM | CG11218-RA | "-"


*********************** Rank 196 [Score  9.281200] GBROWSE*******************

 CG5206 in-situ | bon | + | -4348 | 15031 | INTRAGENIC | intron:CG5206-RA:1 | CG5206-RA | "-"
 CG15923 in-situ | CG15923 | - | 19386 | 15098 | DOWNSTREAM | CG15923-RA | "-"

*********************** Rank 197 [Score  9.276600] GBROWSE*******************

 CG9686 in-situ | CG9686 | + | -2073 | -1407 | DOWNSTREAM | CG9686-RA | "-"
 CG9689 in-situ | CG9689 | + | 41 | 1017 | UPSTREAM | CG9689-RA | "-"

*********************** Rank 198 [Score  9.257600] GBROWSE*******************

insitu CG6821 in-situ | Lsp1gamma | + | -3286 | -716 | DOWNSTREAM | CG6821-RA | "-"
 CG13405 in-situ | CG13405 | - | 5545 | 4749 | DOWNSTREAM | CG13405-RA | "-"

*********************** Rank 199 [Score  9.251600] GBROWSE*******************

 CG3407 in-situ | CG3407 | - | -11124 | -13732 | UPSTREAM | CG3407-RA | "-"
insitu highlight CG16738 in-situ | slp1 | + | 1149 | 2606 | UPSTREAM | CG16738-RA | "-"

note: overlaps known module slp_meso_stripes by 166 bases (module coords: 3815712-3817265)

*********************** Rank 200 [Score  9.246800] GBROWSE*******************

 CG30473 in-situ | Obp51a | - | -37185 | -37605 | UPSTREAM | CG30473-RA | "-"
insitu highlight CG11798 in-situ | CG11798 | + | 66891 | 82132 | UPSTREAM | CG11798-RA | "-"

*********************** Rank 201 [Score  9.243200] GBROWSE*******************

 CG4231 in-situ | Or22b | + | -19243 | -17675 | DOWNSTREAM | CG4231-RA | "-"
 CG14351 in-situ | CG14351 | + | 13225 | 65683 | UPSTREAM | CG14351-RA | "-"

*********************** Rank 202 [Score  9.236900] GBROWSE*******************

 CG32859 in-situ | EG:BACR42I17.1 | - | -4073 | -7836 | UPSTREAM | CG32859-RA | "-"
 CG11378 in-situ | EG:BACR42I17.2 | + | 4966 | 6279 | UPSTREAM | CG11378-RA | "-"

*********************** Rank 203 [Score  9.235400] GBROWSE*******************

 CG30322 in-situ | CG30322 | + | -7448 | -6865 | DOWNSTREAM | CG30322-RA | "-"
 CG30097 in-situ | CG30097 | - | 13023 | 8431 | DOWNSTREAM | CG30097-RB | "-" | CG30097-RA | "-" | CG30097-RD | "-" | CG30097-RE | "-" | CG30097-RC | "-" | CG30097-RF | "-"


*********************** Rank 204 [Score  9.227600] GBROWSE*******************

insitu CG8376 in-situ | ap | - | -15349 | -35977 | UPSTREAM | CG8376-RA | "-" | CG8376-RB | "-"
 CG12792 in-situ | l(2)09851 | + | 12349 | 13874 | UPSTREAM | CG12792-RA | "-"

*********************** Rank 205 [Score  9.222600] GBROWSE*******************

 CG12187 in-situ | CG12187 | - | -45934 | -65018 | UPSTREAM | CG12187-RA | "-"
 CG1262 in-situ | Acp62F | + | 4129 | 11282 | UPSTREAM | CG1262-RA | "-"

*********************** Rank 206 [Score  9.216500] GBROWSE*******************

 CG8517 in-situ | CG8517 | + | -54254 | -53457 | DOWNSTREAM | CG8517-RA | "-"
 CG12501 in-situ | Or56a | - | 2896 | 1124 | DOWNSTREAM | CG12501-RA | "-"

*********************** Rank 207 [Score  9.215100] GBROWSE*******************

 CG32725 in-situ | CG32725 | - | -12912 | -13639 | UPSTREAM | CG32725-RA | "-"
 CG1958 in-situ | CG1958 | + | 15831 | 16808 | UPSTREAM | CG1958-RA | "-"

*********************** Rank 208 [Score  9.209100] GBROWSE*******************

 CG7345 in-situ | Sox21a | - | -16896 | -19716 | UPSTREAM | CG7345-RA | "-"
insitu CG32139 in-situ | Sox21b | - | 7354 | -11587 | INTRAGENIC | intron:CG32139-RA:3 | CG32139-RA | "-"


*********************** Rank 209 [Score  9.203800] GBROWSE*******************

insitu highlight CG10619 in-situ | tup | - | -28461 | -50217 | UPSTREAM | CG10619-RA | "-" | CG10619-RB | "-"
 CG18397 in-situ | CG18397 | - | 34231 | 6808 | DOWNSTREAM | CG18397-RA | "-"

*********************** Rank 210 [Score  9.197700] GBROWSE*******************

insitu CG9233 in-situ | fu2 | + | -5979 | -3773 | DOWNSTREAM | CG9233-RA | "-"
 CG13087 in-situ | CG13087 | + | 5822 | 6598 | UPSTREAM | CG13087-RA | "-"

*********************** Rank 211 [Score  9.193000] GBROWSE*******************

 CG17738 in-situ | CG17738 | + | -29539 | -29207 | DOWNSTREAM | CG17738-RA | "-"
 CG4066 in-situ | CG4066 | + | 10894 | 12660 | UPSTREAM | CG4066-RA | "-"

*********************** Rank 212 [Score  9.188000] GBROWSE*******************

insitu highlight CG31043 in-situ | gukh | + | -7336 | 31077 | INTRAGENIC | intron:CG31043-RA:1 | intron:CG31043-RB:1 | CG31043-RA | "-" | CG31043-RB | "-"
 CG6005 in-situ | CG6005 | - | 32661 | 31240 | DOWNSTREAM | CG6005-RA | "-"

*********************** Rank 213 [Score  9.177900] GBROWSE*******************

 CG10349 in-situ | CG10349 | + | -12185 | -6803 | DOWNSTREAM | CG10349-RA | "-" | CG10349-RB | "-"
 CG31270 in-situ | CG31270 | - | 31872 | 30756 | DOWNSTREAM | CG31270-RA | "-"

*********************** Rank 214 [Score  9.172700] GBROWSE*******************

 CG31275 in-situ | CG31275 | - | -29471 | -30128 | UPSTREAM | CG31275-RB | "-" | CG31275-RA | "-"
 CG3853 in-situ | Glut3 | + | 7451 | 9163 | UPSTREAM | CG3853-RA | "-"

*********************** Rank 215 [Score  9.168800] GBROWSE*******************

 CG15550 in-situ | CG15550 | - | -7264 | -7695 | UPSTREAM | CG15550-RA | "-"
 CG15548 in-situ | CG15548 | + | 15445 | 17889 | UPSTREAM | CG15548-RA | "-"

*********************** Rank 216 [Score  9.165800] GBROWSE*******************

 CG17974 in-situ | CG17974 | - | -8205 | -9994 | UPSTREAM | CG17974-RA | "-"
 CG15671 in-situ | cv-2 | - | 7892 | -3187 | INTRAGENIC | intron:CG15671-RA:3 | CG15671-RA | "-"

*********************** Rank 217 [Score  9.154600] GBROWSE*******************

 CG4969 in-situ | Wnt6 | + | -20221 | -18839 | DOWNSTREAM | CG4969-RA | "-"
 CG4971 in-situ | Wnt10 | + | 3643 | 6140 | UPSTREAM | CG4971-RA | "-"


*********************** Rank 218 [Score  9.148300] GBROWSE*******************

 CG8517 in-situ | CG8517 | + | -53454 | -52657 | DOWNSTREAM | CG8517-RA | "-"
 CG12501 in-situ | Or56a | - | 3696 | 1924 | DOWNSTREAM | CG12501-RA | "-"

*********************** Rank 219 [Score  9.146700] GBROWSE*******************

 CG1668 in-situ | Pbprp2 | + | -3789 | -2420 | DOWNSTREAM | CG1668-RA | "-" | CG1668-RB | "-"
 CG1676 in-situ | cactin | + | 578 | 3010 | UPSTREAM | CG1676-RA | "-"


*********************** Rank 220 [Score  9.143600] GBROWSE*******************

 CG14915 in-situ | CG14915 | + | -1430 | -1068 | DOWNSTREAM | CG14915-RA | "-"
insitu CG14919 in-situ | Ast2 | - | 8906 | 3661 | DOWNSTREAM | CG14919-RA | "-"

*********************** Rank 221 [Score  9.141200] GBROWSE*******************

 CG11913 in-situ | CG11913 | + | -15363 | -13742 | DOWNSTREAM | CG11913-RA | "-"
insitu CG11910 in-situ | CG11910 | - | 9129 | 7392 | DOWNSTREAM | CG11910-RA | "-"


*********************** Rank 222 [Score  9.138500] GBROWSE*******************

 CG31605 in-situ | CG31605 | + | -10397 | 16726 | INTRAGENIC | intron:CG31605-RB:2 | intron:CG31605-RA:2 | intron:CG31605-RD:2 | intron:CG31605-RC:2 | intron:CG31605-RE:2 | intron:CG31605-RF:3 | intron:CG31605-RH:2 | intron:CG31605-RI:2 | CG31605-RB | "-" | CG31605-RA | "-" | CG31605-RD | "-" | CG31605-RC | "-" | CG31605-RE | "-" | CG31605-RF | "-" | CG31605-RH | "-" | CG31605-RI | "-" | CG31605-RG | "-"
 CG31756 in-situ | CG31756 | - | 30182 | 23144 | DOWNSTREAM | CG31756-RA | "-"

*********************** Rank 223 [Score  9.134900] GBROWSE*******************

 CG13123 in-situ | CG13123 | - | -21224 | -22359 | UPSTREAM | CG13123-RA | "-"
 CG31880 in-situ | CG31880 | + | 20199 | 20974 | UPSTREAM | CG31880-RA | "-"

*********************** Rank 224 [Score  9.121000] GBROWSE*******************

 CG30111 in-situ | CG30111 | + | -3980 | -601 | DOWNSTREAM | CG30111-RA | "-"
 CG11430 in-situ | olf186-F | + | 25120 | 42245 | UPSTREAM | CG11430-RB | "-" | CG11430-RC | "-" | CG11430-RA | "-"

*********************** Rank 225 [Score  9.120600] GBROWSE*******************

insitu highlight CG32026 in-situ | CG32026 | + | -44028 | -41647 | DOWNSTREAM | CG32026-RA | "-"
 CG4978 in-situ | Mcm7 | + | 11120 | 13845 | UPSTREAM | CG4978-RA | "-"


*********************** Rank 226 [Score  9.106100] GBROWSE*******************

 CG12644 in-situ | CG12644 | + | -4982 | -4701 | DOWNSTREAM | CG12644-RA | "-"
 CG32691 in-situ | CG32691 | + | 745 | 1334 | UPSTREAM | CG32691-RA | "-"

*********************** Rank 227 [Score  9.105500] GBROWSE*******************

 CG31531 in-situ | CG31531 | + | -7946 | 37728 | INTRAGENIC | intron:CG31531-RA:2 | intron:CG31531-RC:2 | intron:CG31531-RB:1 | CG31531-RA | "-" | CG31531-RC | "-" | CG31531-RB | "-"
insitu CG31534 in-situ | CG31534 | + | 38617 | 44664 | UPSTREAM | CG31534-RA | "-" | CG31534-RB | "-"

*********************** Rank 228 [Score  9.097800] GBROWSE*******************

 CG8285 in-situ | boss | + | -2419 | 2535 | INTRAGENIC | intron:CG8285-RA:3 | CG8285-RA | "-"
 CG14541 in-situ | CG14541 | + | 8075 | 9406 | UPSTREAM | CG14541-RA | "-"

*********************** Rank 229 [Score  9.089500] GBROWSE*******************

 CG14061 in-situ | CG14061 | - | -31778 | -32878 | UPSTREAM | CG14061-RA | "-"
 CG12558 in-situ | CG12558 | + | 510 | 1449 | UPSTREAM | CG12558-RA | "-"

*********************** Rank 230 [Score  9.087200] GBROWSE*******************

insitu CG5799 in-situ | dve | + | -44336 | -1881 | DOWNSTREAM | CG5799-RA | "-" | CG5799-RD | "-" | CG5799-RB | "-" | CG5799-RC | "-"
insitu CG5819 in-situ | CG5819 | + | 5160 | 8659 | UPSTREAM | CG5819-RA | "-" | CG5819-RB | "-"

*********************** Rank 231 [Score  9.087200] GBROWSE*******************

 CG13789 in-situ | CG13789 | - | -27231 | -27868 | UPSTREAM | CG13789-RA | "-"
 CG13790 in-situ | CG13790 | - | 4999 | 4715 | DOWNSTREAM | CG13790-RA | "-"

*********************** Rank 232 [Score  9.083100] GBROWSE*******************

insitu CG5248 in-situ | loco | - | -10762 | -30154 | UPSTREAM | CG5248-RD | "-" | CG5248-RA | "-" | CG5248-RB | "-" | CG5248-RC | "-"
 CG17622 in-situ | CG17622 | + | 6492 | 9122 | UPSTREAM | CG17622-RA | "-"


*********************** Rank 233 [Score  9.080600] GBROWSE*******************

 CG6486 in-situ | CG6486 | + | -8516 | -7324 | DOWNSTREAM | CG6486-RA | "-"
insitu highlight CG6494 in-situ | h | + | 17919 | 21199 | UPSTREAM | CG6494-RA | "-"

*********************** Rank 234 [Score  9.080100] GBROWSE*******************

 CG14184 in-situ | CG14184 | - | -48110 | -49095 | UPSTREAM | CG14184-RA | "-"
 CG7395 in-situ | NPFR76F | - | 11920 | -17066 | INTRAGENIC | intron:CG7395-RA:1 | CG7395-RA | "-"

*********************** Rank 235 [Score  9.078000] GBROWSE*******************

 CG15925 in-situ | CG15925 | + | -11339 | -9811 | DOWNSTREAM | CG15925-RA | "-"
 CG15712 in-situ | CG15712 | - | 28215 | 27516 | DOWNSTREAM | CG15712-RA | "-"


*********************** Rank 236 [Score  9.077900] GBROWSE*******************

 CG32774 in-situ | CG32774 | - | -5665 | -7213 | UPSTREAM | CG32774-RA | "-"
 CG15576 in-situ | CG15576 | - | 1263 | 441 | DOWNSTREAM | CG15576-RB | "-"

*********************** Rank 237 [Score  9.055500] GBROWSE*******************

 CG1421 in-situ | CG1421 | + | -2462 | -1785 | DOWNSTREAM | CG1421-RA | "-"
 CG1428 in-situ | CG1428 | + | 765 | 2808 | UPSTREAM | CG1428-RA | "-"

*********************** Rank 238 [Score  9.054300] GBROWSE*******************

 CG10037 in-situ | vvl | + | -65261 | -60885 | DOWNSTREAM | CG10037-RA | "-"
insitu CG10078 in-situ | Prat2 | - | 59437 | 56572 | DOWNSTREAM | CG10078-RB | "-" | CG10078-RA | "-"

*********************** Rank 239 [Score  9.054300] GBROWSE*******************

insitu CG32159 in-situ | CG32159 | - | -26767 | -55927 | UPSTREAM | CG32159-RB | "-"
insitu highlight CG4531 in-situ | argos | - | 4980 | -8342 | INTRAGENIC | intron:CG4531-RA:1 | CG4531-RA | "-"

*********************** Rank 240 [Score  9.049900] GBROWSE*******************

 CG18281 in-situ | CG18281 | + | -1726 | -62 | DOWNSTREAM | CG18281-RA | "-"
 CG17637 in-situ | CG17637 | + | 2624 | 4138 | UPSTREAM | CG17637-RA | "-"


*********************** Rank 241 [Score  9.047900] GBROWSE*******************

 CG13493 in-situ | CG13493 | - | -1890 | -3882 | UPSTREAM | CG13493-RA | "-"
 CG3245 in-situ | PpN58A | - | 14134 | 10530 | DOWNSTREAM | CG3245-RB | "-" | CG3245-RA | "-"

*********************** Rank 242 [Score  9.047600] GBROWSE*******************

 CG12681 in-situ | CG12681 | + | -14505 | -13042 | DOWNSTREAM | CG12681-RA | "-"
 CG15470 in-situ | CG15470 | + | 4503 | 5663 | UPSTREAM | CG15470-RA | "-"

*********************** Rank 243 [Score  9.044700] GBROWSE*******************

 CG17744 in-situ | CG17744 | - | -190 | -1170 | UPSTREAM | CG17744-RA | "-"
 CG8641 in-situ | CG8641 | - | 35903 | 33361 | DOWNSTREAM | CG8641-RA | "-"

*********************** Rank 244 [Score  9.038900] GBROWSE*******************

 CG14273 in-situ | CG14273 | + | -2905 | -1359 | DOWNSTREAM | CG14273-RA | "-"
 CG7778 in-situ | CG7778 | + | 952 | 4528 | UPSTREAM | CG7778-RA | "-"


*********************** Rank 245 [Score  9.036000] GBROWSE*******************

insitu CG2199 in-situ | CG2199 | - | -7018 | -9964 | UPSTREAM | CG2199-RB | "-" | CG2199-RA | "-"
 CG9166 in-situ | 312 | + | 1718 | 3432 | UPSTREAM | CG9166-RA | "-"

*********************** Rank 246 [Score  9.029500] GBROWSE*******************

 CG15172 in-situ | CG15172 | + | -2503 | -2017 | DOWNSTREAM | CG15172-RA | "-"
insitu CG10446 in-situ | Side | + | 2045 | 5120 | UPSTREAM | CG10446-RA | "-"

*********************** Rank 247 [Score  9.023300] GBROWSE*******************

 CG6391 in-situ | CG6391 | - | -27574 | -31274 | UPSTREAM | CG6391-RA | "-" | CG6391-RB | "-" | CG6391-RC | "-"
 CG14148 in-situ | CG14148 | - | 13595 | 13305 | DOWNSTREAM | CG14148-RA | "-"

*********************** Rank 248 [Score  9.017800] GBROWSE*******************

 CG12857 in-situ | CG12857 | - | -5380 | -6922 | UPSTREAM | CG12857-RA | "-"
insitu CG10155 in-situ | CG10155 | - | 4448 | -3824 | INTRAGENIC | intron:CG10155-RA:2 | CG10155-RA | "-"

*********************** Rank 249 [Score  9.016800] GBROWSE*******************

 CG11692 in-situ | CG11692 | - | -13272 | -13828 | UPSTREAM | CG11692-RA | "-"
 CG1829 in-situ | Cyp6v1 | + | 5884 | 9353 | UPSTREAM | CG1829-RA | "-"


*********************** Rank 250 [Score  9.016000] GBROWSE*******************

 CG13908 in-situ | CG13908 | + | -43310 | -38057 | DOWNSTREAM | CG13908-RA | "-"
 CG9205 in-situ | CG9205 | + | 4176 | 5777 | UPSTREAM | CG9205-RA | "-" | CG9205-RB | "-"

*********************** Rank 251 [Score  9.014600] GBROWSE*******************

insitu highlight CG4922 in-situ | sala | + | -5340 | -4574 | DOWNSTREAM | CG4922-RA | "-"
 CG6488 in-situ | CG6488 | - | 7590 | 5265 | DOWNSTREAM | CG6488-RA | "-"

*********************** Rank 252 [Score  9.011400] GBROWSE*******************

insitu CG31163 in-situ | CG31163 | + | -9156 | 155057 | INTRAGENIC | intron:CG31163-RB:2 | intron:CG31163-RC:1 | CG31163-RB | "-" | CG31163-RC | "-" | CG31163-RA | "-"
 CG5732 in-situ | CG5732 | + | 68810 | 73682 | UPSTREAM | CG5732-RA | "-"


*********************** Rank 253 [Score  9.010700] GBROWSE*******************

insitu CG6030 in-situ | ATPsyn-d | + | -15013 | -13941 | DOWNSTREAM | CG6030-RB | "-" | CG6030-RA | "-"
insitu CG6040 in-situ | CG6040 | + | 13989 | 21928 | UPSTREAM | CG6040-RA | "-"


*********************** Rank 254 [Score  9.008400] GBROWSE*******************

 CG18023 in-situ | Eip78C | + | -34305 | 3397 | INTRAGENIC | intron:CG18023-RA:3 | intron:CG18023-RB:3 | CG18023-RA | "-" | CG18023-RB | "-"
insitu CG9391 in-situ | CG9391 | - | 7116 | 5554 | DOWNSTREAM | CG9391-RB | "-" | CG9391-RA | "-"

*********************** Rank 255 [Score  8.998000] GBROWSE*******************

 CG15399 in-situ | CG15399 | - | -19896 | -20693 | UPSTREAM | CG15399-RA | "-"
insitu CG3139 in-situ | syt | - | 8339 | -10890 | INTRAGENIC | intron:CG3139-RA:7 | intron:CG3139-RB:7 | intron:CG3139-RC:6 | CG3139-RA | "-" | CG3139-RB | "-" | CG3139-RC | "-"

*********************** Rank 256 [Score  8.978800] GBROWSE*******************

 CG3655 in-situ | EG:103E12.3 | - | -1824 | -10365 | UPSTREAM | CG3655-RA | "-"
 CG14628 in-situ | EG:BACR42I17.12 | + | 61415 | 61840 | UPSTREAM | CG14628-RA | "-"

*********************** Rank 257 [Score  8.975800] GBROWSE*******************

 CG3759 in-situ | CG3759 | + | -1822 | 7929 | INTRAGENIC | intron:CG3759-RA:1 | CG3759-RA | "-"
 CG3763 in-situ | Fbp2 | + | 8457 | 9421 | UPSTREAM | CG3763-RA | "-"

*********************** Rank 258 [Score  8.972500] GBROWSE*******************

insitu CG13321 in-situ | CG13321 | + | -6580 | -4961 | DOWNSTREAM | CG13321-RA | "-"
 CG3886 in-situ | Psc | - | 20853 | 6174 | DOWNSTREAM | CG3886-RA | "-"

*********************** Rank 259 [Score  8.969200] GBROWSE*******************

insitu highlight CG7952 in-situ | gt | - | -2015 | -3871 | UPSTREAM | CG7952-RB | "-"
 CG7925 in-situ | tko | - | 12998 | 11329 | DOWNSTREAM | CG7925-RB | "-"

note: overlaps known module gt_posterior by 500 bases (module coords: 2187439-2188383)

*********************** Rank 260 [Score  8.962200] GBROWSE*******************

 CG6660 in-situ | CG6660 | + | -3101 | -2221 | DOWNSTREAM | CG6660-RA | "-"
 CG31281 in-situ | CG31281 | + | 39729 | 40889 | UPSTREAM | CG31281-RA | "-"

*********************** Rank 261 [Score  8.958500] GBROWSE*******************

 CG14678 in-situ | CG14678 | - | -70923 | -73724 | UPSTREAM | CG14678-RA | "-"
 CG11373 in-situ | CG11373 | - | 2604 | 1942 | DOWNSTREAM | CG11373-RA | "-"

*********************** Rank 262 [Score  8.951500] GBROWSE*******************

 CG6604 in-situ | H15 | + | -12443 | -857 | DOWNSTREAM | CG6604-RA | "-"
 CG31647 in-situ | CG31647 | - | 32417 | 18794 | DOWNSTREAM | CG31647-RA | "-" | CG31647-RB | "-"

*********************** Rank 263 [Score  8.948500] GBROWSE*******************

 CG17657 in-situ | CG17657 | - | -2674 | -7236 | UPSTREAM | CG17657-RA | "-"
 CG18317 in-situ | CG18317 | - | 5856 | -1647 | INTRAGENIC | intron:CG18317-RA:2 | CG18317-RA | "-"

*********************** Rank 264 [Score  8.946300] GBROWSE*******************

 CG14067 in-situ | CG14067 | - | -8721 | -9026 | UPSTREAM | CG14067-RA | "-"
 CG14072 in-situ | CG14072 | + | 3144 | 3567 | UPSTREAM | CG14072-RA | "-"

*********************** Rank 265 [Score  8.944900] GBROWSE*******************

 CG32310 in-situ | CG32310 | - | -1799 | -3496 | UPSTREAM | CG32310-RA | "-"
 CG32311 in-situ | CG32311 | - | 5636 | -1620 | INTRAGENIC | intron:CG32311-RB:4 | CG32311-RB | "-"

*********************** Rank 266 [Score  8.941700] GBROWSE*******************

 CG4546 in-situ | CG4546 | + | -6652 | -4724 | DOWNSTREAM | CG4546-RA | "-"
insitu CG31150 in-situ | CG31150 | - | 419 | -8694 | INTRAGENIC | intron:CG31150-RA:1 | CG31150-RA | "-"

*********************** Rank 267 [Score  8.941600] GBROWSE*******************

 CG32613 in-situ | CG32613 | - | -24360 | -41369 | UPSTREAM | CG32613-RA | "-"
 CG18319 in-situ | ben | + | 4663 | 8117 | UPSTREAM | CG18319-RA | "-"

*********************** Rank 268 [Score  8.940800] GBROWSE*******************

 CG6697 in-situ | BcDNA:LD21504 | + | -4477 | -3220 | DOWNSTREAM | CG6697-RA | "-"
 CG17111 in-situ | CG17111 | - | 281 | -3197 | INTRAGENIC | intron:CG17111-RA:1 | CG17111-RA | "-"

*********************** Rank 269 [Score  8.939900] GBROWSE*******************

 CG13057 in-situ | retinin | + | -952 | -74 | DOWNSTREAM | CG13057-RA | "-"
 CG13056 in-situ | CG13056 | + | 619 | 831 | UPSTREAM | CG13056-RA | "-"


*********************** Rank 270 [Score  8.939300] GBROWSE*******************

insitu CG10610 in-situ | ECSIT | + | -4946 | -3617 | DOWNSTREAM | CG10610-RA | "-"
 CG2017 in-situ | CG2017 | - | 880 | -3454 | INTRAGENIC | intron:CG2017-RA:1 | intron:CG2017-RD:1 | intron:CG2017-RC:1 | CG2017-RA | "-" | CG2017-RD | "-" | CG2017-RC | "-" | CG2017-RB | "-"

*********************** Rank 271 [Score  8.936300] GBROWSE*******************

 CG31213 in-situ | CG31213 | + | -30598 | -25099 | DOWNSTREAM | CG31213-RA | "-"
 CG4451 in-situ | Hs6st | - | 4411 | -74397 | INTRAGENIC | intron:CG4451-RA:2 | CG4451-RA | "-"

*********************** Rank 272 [Score  8.928300] GBROWSE*******************

 CG10862 in-situ | CG10862 | - | -80350 | -81656 | UPSTREAM | CG10862-RA | "-"
 CG10858 in-situ | CG10858 | - | 20080 | 13404 | DOWNSTREAM | CG10858-RA | "-"

*********************** Rank 273 [Score  8.924500] GBROWSE*******************

 CG14628 in-situ | EG:BACR42I17.12 | + | -1035 | -610 | DOWNSTREAM | CG14628-RA | "-"
 CG32859 in-situ | EG:BACR42I17.1 | - | 15677 | 11914 | DOWNSTREAM | CG32859-RA | "-"

*********************** Rank 274 [Score  8.924300] GBROWSE*******************

 CG6127 in-situ | Ser | - | -18419 | -40317 | UPSTREAM | CG6127-RA | "-"
 CG31063 in-situ | CG31063 | - | 17011 | 15025 | DOWNSTREAM | CG31063-RA | "-"

*********************** Rank 275 [Score  8.914900] GBROWSE*******************

 CG9565 in-situ | Nep3 | + | -35852 | -29046 | DOWNSTREAM | CG9565-RA | "-"
 CG17003 in-situ | CG17003 | - | 4555 | 3426 | DOWNSTREAM | CG17003-RA | "-"

*********************** Rank 276 [Score  8.913900] GBROWSE*******************

 CG30442 in-situ | CG30442 | - | -22277 | -23586 | UPSTREAM | CG30442-RB | "-"
 CG2944 in-situ | CG2944 | - | 9282 | -1820 | INTRAGENIC | intron:CG2944-RB:2 | intron:CG2944-RA:2 | intron:CG2944-RC:3 | intron:CG2944-RD:3 | intron:CG2944-RE:3 | intron:CG2944-RF:2 | CG2944-RB | "-" | CG2944-RA | "-" | CG2944-RC | "-" | CG2944-RD | "-" | CG2944-RE | "-" | CG2944-RF | "-"

*********************** Rank 277 [Score  8.910000] GBROWSE*******************

insitu CG4145 in-situ | Cg25C | + | -2814 | 4685 | INTRAGENIC | intron:CG4145-RC:5 | intron:CG4145-RB:5 | intron:CG4145-RA:5 | CG4145-RC | "-" | CG4145-RB | "-" | CG4145-RA | "-"
insitu CG14041 in-situ | SP555 | + | 5624 | 8389 | UPSTREAM | CG14041-RA | "-" | CG14041-RB | "-"

*********************** Rank 278 [Score  8.908500] GBROWSE*******************

 CG17330 in-situ | BG:DS09218.5 | + | -49447 | -48389 | DOWNSTREAM | CG17330-RA | "-"
 CG4472 in-situ | Idgf1 | + | 29914 | 31481 | UPSTREAM | CG4472-RA | "-"

*********************** Rank 279 [Score  8.907700] GBROWSE*******************

 CG9380 in-situ | CG9380 | - | -35193 | -40401 | UPSTREAM | CG9380-RA | "-" | CG9380-RB | "-"
insitu highlight CG3340 in-situ | Kr | + | 2585 | 5504 | UPSTREAM | CG3340-RA | "-"


note: overlaps known module Kr_AS3 by 154 bases (module coords: 20267163-20267753)
note: overlaps known module Kr_CD2_AD1 by 478 bases (module coords: 20267622-20269328)
note: overlaps known module Kr_M by 132 bases (module coords: 20267622-20267753)

*********************** Rank 280 [Score  8.898100] GBROWSE*******************

 CG3578 in-situ | bi | + | -73728 | -2120 | DOWNSTREAM | CG3578-RA | "-"
 CG12685 in-situ | CG12685 | + | 29030 | 29598 | UPSTREAM | CG12685-RA | "-"

*********************** Rank 281 [Score  8.890400] GBROWSE*******************

 CG32206 in-situ | CG32206 | - | -19779 | -92350 | UPSTREAM | CG32206-RB | "-" | CG32206-RC | "-"
 CG33062 in-situ | CG33062 | - | 13712 | 9427 | DOWNSTREAM | CG33062-RA | "-"


*********************** Rank 282 [Score  8.884300] GBROWSE*******************

 CG12109 in-situ | Caf1-180 | - | -37128 | -40993 | UPSTREAM | CG12109-RB | "-" | CG12109-RA | "-"
insitu highlight CG12154 in-situ | oc | - | 7296 | -12014 | INTRAGENIC | intron:CG12154-RA:1 | CG12154-RA | "-"

*********************** Rank 283 [Score  8.882300] GBROWSE*******************

 CG12479 in-situ | CG12479 | - | -2727 | -2951 | UPSTREAM | CG12479-RA | "-"
 CG12480 in-situ | CG12480 | + | 14972 | 16914 | UPSTREAM | CG12480-RA | "-"

*********************** Rank 284 [Score  8.879000] GBROWSE*******************

 CG5557 in-situ | sqz | + | -10340 | -3987 | DOWNSTREAM | CG5557-RA | "-"
 CG14282 in-situ | CG14282 | + | 2439 | 3372 | UPSTREAM | CG14282-RA | "-"

*********************** Rank 285 [Score  8.876600] GBROWSE*******************

 CG32080 in-situ | CG32080 | + | -16066 | -15305 | DOWNSTREAM | CG32080-RA | "-"
 CG12296 in-situ | klu | - | 17156 | -9942 | INTRAGENIC | intron:CG12296-RA:2 | CG12296-RA | "-"

*********************** Rank 286 [Score  8.873300] GBROWSE*******************

 CG4161 in-situ | BG:DS03023.2 | + | -6453 | -4565 | DOWNSTREAM | CG4161-RA | "-"
insitu CG3956 in-situ | sna | - | 38453 | 36777 | DOWNSTREAM | CG3956-RA | "-"

*********************** Rank 287 [Score  8.871900] GBROWSE*******************

 CG7850 in-situ | puc | + | -2578 | 14388 | INTRAGENIC | intron:CG7850-RA:1 | CG7850-RA | "-"
 CG7878 in-situ | CG7878 | + | 14786 | 17362 | UPSTREAM | CG7878-RA | "-"


*********************** Rank 288 [Score  8.870400] GBROWSE*******************

 CG9519 in-situ | CG9519 | - | -5925 | -7961 | UPSTREAM | CG9519-RA | "-"
insitu CG9518 in-situ | CG9518 | - | 8888 | 703 | DOWNSTREAM | CG9518-RA | "-"

*********************** Rank 289 [Score  8.870200] GBROWSE*******************

 CG4122 in-situ | svr | + | -11584 | -185 | DOWNSTREAM | CG4122-RA | "-" | CG4122-RB | "-" | CG4122-RC | "-"
 CG18104 in-situ | arg | + | 1356 | 19975 | UPSTREAM | CG18104-RA | "-"

*********************** Rank 290 [Score  8.867600] GBROWSE*******************

 CG15147 in-situ | CG15147 | - | -85672 | -86270 | UPSTREAM | CG15147-RA | "-"
 CG7100 in-situ | CadN | - | 27504 | -62151 | INTRAGENIC | intron:CG7100-RA:7 | intron:CG7100-RC:7 | intron:CG7100-RD:7 | intron:CG7100-RE:7 | intron:CG7100-RF:7 | intron:CG7100-RG:7 | intron:CG7100-RH:7 | intron:CG7100-RB:7 | CG7100-RA | "-" | CG7100-RC | "-" | CG7100-RD | "-" | CG7100-RE | "-" | CG7100-RF | "-" | CG7100-RG | "-" | CG7100-RH | "-" | CG7100-RB | "-"

*********************** Rank 291 [Score  8.862100] GBROWSE*******************

 CG13758 in-situ | EG:BACR25B3.3 | + | -20227 | 2125 | INTRAGENIC | intron:CG13758-RA:3 | CG13758-RA | "-"
 CG8310 in-situ | EG:BACR25B3.4 | - | 5886 | 4955 | DOWNSTREAM | CG8310-RA | "-"

*********************** Rank 292 [Score  8.861100] GBROWSE*******************

 CG11769 in-situ | CG11769 | + | -13060 | -12314 | DOWNSTREAM | CG11769-RA | "-"
 CG31448 in-situ | CG31448 | + | 17108 | 17800 | UPSTREAM | CG31448-RA | "-"

*********************** Rank 293 [Score  8.861000] GBROWSE*******************

 CG18247 in-situ | shark | - | -5392 | -8852 | UPSTREAM | CG18247-RA | "-"
 CG8448 in-situ | CG8448 | - | 13521 | -4953 | INTRAGENIC | intron:CG8448-RD:1 | intron:CG8448-RB:1 | intron:CG8448-RC:1 | CG8448-RD | "-" | CG8448-RB | "-" | CG8448-RC | "-" | CG8448-RA | "-"

*********************** Rank 294 [Score  8.859300] GBROWSE*******************

 CG13432 in-situ | l(2)05510 | - | -4360 | -23453 | UPSTREAM | CG13432-RA | "-"
insitu CG13434 in-situ | CG13434 | - | 719 | -269 | INTRAGENIC | intron:CG13434-RA:3 | CG13434-RA | "-"

*********************** Rank 295 [Score  8.857800] GBROWSE*******************

 CG15614 in-situ | CG15614 | + | -726 | 836 | INTRAGENIC | intron:CG15614-RA:1 | CG15614-RA | "-"
 CG9013 in-situ | CG9013 | - | 1853 | 1386 | DOWNSTREAM | CG9013-RA | "-"

*********************** Rank 296 [Score  8.855200] GBROWSE*******************

 CG11920 in-situ | CG11920 | + | -3789 | -2289 | DOWNSTREAM | CG11920-RA | "-"
 CG11921 in-situ | fd96Ca | + | 4881 | 5999 | UPSTREAM | CG11921-RA | "-"

*********************** Rank 297 [Score  8.854000] GBROWSE*******************

 CG2107 in-situ | CG2107 | + | -11390 | -8870 | DOWNSTREAM | CG2107-RA | "-"
 CG2113 in-situ | CG2113 | + | 2759 | 3737 | UPSTREAM | CG2113-RA | "-"

*********************** Rank 298 [Score  8.849400] GBROWSE*******************

insitu CG6921 in-situ | CG6921 | + | -6989 | -1172 | DOWNSTREAM | CG6921-RB | "-" | CG6921-RA | "-" | CG6921-RC | "-"
insitu CG5278 in-situ | CG5278 | - | 4899 | 1189 | DOWNSTREAM | CG5278-RA | "-"

*********************** Rank 299 [Score  8.843600] GBROWSE*******************

 CG4563 in-situ | CG4563 | - | -10445 | -12400 | UPSTREAM | CG4563-RA | "-"
 CG3492 in-situ | CG3492 | + | 9230 | 10751 | UPSTREAM | CG3492-RA | "-"


*********************** Rank 300 [Score  8.837400] GBROWSE*******************

 CG31672 in-situ | BEST:LD15963 | + | -7660 | -5131 | DOWNSTREAM | CG31672-RA | "-"
 CG15377 in-situ | Or22c | + | 1404 | 4782 | UPSTREAM | CG15377-RA | "-"

*********************** Rank 301 [Score  8.835300] GBROWSE*******************

 CG15532 in-situ | hdc | + | -54328 | 29907 | INTRAGENIC | intron:CG15532-RA:2 | intron:CG15532-RC:2 | CG15532-RA | "-" | CG15532-RC | "-" | CG15532-RB | "-"
 CG1469 in-situ | Fer2LCH | + | 55570 | 58322 | UPSTREAM | CG1469-RA | "-" | CG1469-RB | "-" | CG1469-RC | "-"


*********************** Rank 302 [Score  8.828400] GBROWSE*******************

 CG31749 in-situ | CG31749 | + | -52803 | -52013 | DOWNSTREAM | CG31749-RA | "-"
 CG10305 in-situ | RpS26 | - | 4120 | 3381 | DOWNSTREAM | CG10305-RA | "-" | CG10305-RB | "-" | CG10305-RC | "-"

*********************** Rank 303 [Score  8.817700] GBROWSE*******************

 CG31923 in-situ | CG31923 | + | -64256 | -63568 | DOWNSTREAM | CG31923-RA | "-"
 CG4375 in-situ | CG4375 | - | 31963 | 31173 | DOWNSTREAM | CG4375-RA | "-"

*********************** Rank 304 [Score  8.817100] GBROWSE*******************

 CG12650 in-situ | CG12650 | + | -65224 | -57929 | DOWNSTREAM | CG12650-RB | "-"
 CG15316 in-situ | CG15316 | - | 23205 | -55183 | INTRAGENIC | intron:CG15316-RB:1 | intron:CG15316-RA:1 | CG15316-RB | "-" | CG15316-RA | "-"

*********************** Rank 305 [Score  8.813400] GBROWSE*******************

 CG30330 in-situ | Gr59d | - | -5763 | -6991 | UPSTREAM | CG30330-RA | "-"
 CG3219 in-situ | Klp59C | - | 15110 | 13095 | DOWNSTREAM | CG3219-RA | "-"

*********************** Rank 306 [Score  8.811200] GBROWSE*******************

 CG15136 in-situ | CG15136 | - | -10985 | -11718 | UPSTREAM | CG15136-RA | "-"
 CG12620 in-situ | CG12620 | + | 28761 | 29622 | UPSTREAM | CG12620-RA | "-"

*********************** Rank 307 [Score  8.811000] GBROWSE*******************

 CG11320 in-situ | CG11320 | - | -3508 | -4567 | UPSTREAM | CG11320-RA | "-"
 CG11053 in-situ | CG11053 | + | 1908 | 3017 | UPSTREAM | CG11053-RA | "-"

*********************** Rank 308 [Score  8.807100] GBROWSE*******************

 CG15150 in-situ | CG15150 | - | -19258 | -20557 | UPSTREAM | CG15150-RA | "-"
insitu CG15151 in-situ | PFE | + | 20994 | 39779 | UPSTREAM | CG15151-RA | "-"

*********************** Rank 309 [Score  8.806800] GBROWSE*******************

 CG15125 in-situ | CG15125 | + | -4097 | -2293 | DOWNSTREAM | CG15125-RA | "-"
 CG11018 in-situ | CG11018 | + | 556 | 1858 | UPSTREAM | CG11018-RA | "-"

*********************** Rank 310 [Score  8.801200] GBROWSE*******************

 CG31386 in-situ | CG31386 | - | -31502 | -57357 | UPSTREAM | CG31386-RA | "-"
 CG17216 in-situ | KP78b | - | 21681 | 19598 | DOWNSTREAM | CG17216-RA | "-"

*********************** Rank 311 [Score  8.797000] GBROWSE*******************

 CG31921 in-situ | CG31921 | - | -4865 | -7209 | UPSTREAM | CG31921-RA | "-"
 CG11907 in-situ | CG11907 | - | 20667 | 18358 | DOWNSTREAM | CG11907-RA | "-" | CG11907-RB | "-"

*********************** Rank 312 [Score  8.795000] GBROWSE*******************

 CG31085 in-situ | CG31085 | + | -19858 | -6774 | DOWNSTREAM | CG31085-RB | "-" | CG31085-RA | "-"
 CG14239 in-situ | CG14239 | - | 34025 | 33146 | DOWNSTREAM | CG14239-RA | "-"

*********************** Rank 313 [Score  8.793000] GBROWSE*******************

 CG30437 in-situ | CG30437 | + | -39493 | 922 | INTRAGENIC | intron:CG30437-RC:6 | CG30437-RA | "-" | CG30437-RC | "-" | CG30437-RB | "-"
 CG32838 in-situ | CG32838 | + | 1209 | 1784 | UPSTREAM | CG32838-RA | "-"

*********************** Rank 314 [Score  8.792100] GBROWSE*******************

 CG15729 in-situ | CG15729 | - | -14689 | -17097 | UPSTREAM | CG15729-RA | "-"
 CG11138 in-situ | CG11138 | - | 3042 | -8930 | INTRAGENIC | intron:CG11138-RC:1 | CG11138-RC | "-" | CG11138-RB | "-"


*********************** Rank 315 [Score  8.788600] GBROWSE*******************

 CG3881 in-situ | CG3881 | + | -1702 | 4944 | INTRAGENIC | intron:CG3881-RA:1 | intron:CG3881-RB:1 | CG3881-RA | "-" | CG3881-RB | "-"
 CG31882 in-situ | CG31882 | - | 7114 | 6412 | DOWNSTREAM | CG31882-RA | "-"


*********************** Rank 316 [Score  8.787300] GBROWSE*******************

 CG31230 in-situ | CG31230 | + | -432 | -64 | DOWNSTREAM | CG31230-RA | "-"
 CG7705 in-situ | CG7705 | - | 5445 | 2565 | DOWNSTREAM | CG7705-RA | "-" | CG7705-RB | "-"

*********************** Rank 317 [Score  8.787000] GBROWSE*******************

 CG6604 in-situ | H15 | + | -40243 | -28657 | DOWNSTREAM | CG6604-RA | "-"
 CG31647 in-situ | CG31647 | - | 4617 | -9006 | INTRAGENIC | intron:CG31647-RA:3 | CG31647-RA | "-" | CG31647-RB | "-"

*********************** Rank 318 [Score  8.781200] GBROWSE*******************

insitu CG12214 in-situ | CG12214 | - | -29887 | -32397 | UPSTREAM | CG12214-RB | "-" | CG12214-RA | "-"
insitu CG33183 in-situ | Hr46 | - | 44624 | 16669 | DOWNSTREAM | CG33183-RC | "-" | CG33183-RB | "-" | CG33183-RA | "-"

*********************** Rank 319 [Score  8.778600] GBROWSE*******************

 CG14586 in-situ | CG14586 | - | -4302 | -8898 | UPSTREAM | CG14586-RA | "-"
 CG7446 in-situ | Grd | + | 1987 | 11922 | UPSTREAM | CG7446-RA | "-"

*********************** Rank 320 [Score  8.764600] GBROWSE*******************

 CG32450 in-situ | CG32450 | + | -72844 | -71945 | DOWNSTREAM | CG32450-RA | "-"
insitu highlight CG5723 in-situ | Ten-m | - | 5265 | -109590 | INTRAGENIC | intron:CG5723-RB:1 | CG5723-RB | "-"


*********************** Rank 321 [Score  8.763300] GBROWSE*******************

insitu highlight CG3136 in-situ | CG3136 | + | -11933 | -1869 | DOWNSTREAM | CG3136-RA | "-" | CG3136-RB | "-"
 CG14768 in-situ | CG14768 | + | 20471 | 20806 | UPSTREAM | CG14768-RA | "-"

*********************** Rank 322 [Score  8.762900] GBROWSE*******************

 CG5151 in-situ | CG5151 | - | -8637 | -17888 | UPSTREAM | CG5151-RA | "-"
 CG13073 in-situ | CG13073 | - | 56735 | 55680 | DOWNSTREAM | CG13073-RB | "-" | CG13073-RA | "-"

*********************** Rank 323 [Score  8.761200] GBROWSE*******************

 CG5290 in-situ | CG5290 | - | -21264 | -24342 | UPSTREAM | CG5290-RA | "-"
 CG32193 in-situ | CG32193 | + | 27839 | 31859 | UPSTREAM | CG32193-RA | "-"


*********************** Rank 324 [Score  8.757900] GBROWSE*******************

 CG12681 in-situ | CG12681 | + | -6655 | -5192 | DOWNSTREAM | CG12681-RA | "-"
 CG15470 in-situ | CG15470 | + | 12353 | 13513 | UPSTREAM | CG15470-RA | "-"

*********************** Rank 325 [Score  8.756800] GBROWSE*******************

 CG15884 in-situ | CG15884 | - | -690 | -2118 | UPSTREAM | CG15884-RA | "-"
 CG14258 in-situ | CG14258 | + | 1366 | 2323 | UPSTREAM | CG14258-RA | "-"

*********************** Rank 326 [Score  8.749400] GBROWSE*******************

 CG4786 in-situ | CG4786 | - | -78798 | -91244 | UPSTREAM | CG4786-RA | "-"
insitu highlight CG4761 in-situ | knrl | - | 1324 | -22071 | INTRAGENIC | intron:CG4761-RA:1 | CG4761-RA | "-"


*********************** Rank 327 [Score  8.740700] GBROWSE*******************

 CG14560 in-situ | msopa | + | -78414 | -77919 | DOWNSTREAM | CG14560-RA | "-"
 CG15374 in-situ | CG15374 | + | 4205 | 4699 | UPSTREAM | CG15374-RA | "-"

*********************** Rank 328 [Score  8.738900] GBROWSE*******************

 CG10366 in-situ | CG10366 | + | -24708 | -22527 | DOWNSTREAM | CG10366-RA | "-"
 CG10443 in-situ | Lar | + | 2443 | 122595 | UPSTREAM | CG10443-RA | "-"

*********************** Rank 329 [Score  8.735700] GBROWSE*******************

 CG2096 in-situ | flw | + | -19309 | 3193 | INTRAGENIC | intron:CG2096-RA:2 | intron:CG2096-RB:1 | CG2096-RA | "-" | CG2096-RB | "-"
 CG2885 in-situ | CG2885 | + | 12603 | 13190 | UPSTREAM | CG2885-RA | "-"

*********************** Rank 330 [Score  8.734900] GBROWSE*******************

 CG12924 in-situ | CG12924 | - | -267 | -1124 | UPSTREAM | CG12924-RA | "-"
 CG1665 in-situ | CG1665 | + | 24 | 1395 | UPSTREAM | CG1665-RA | "-"

*********************** Rank 331 [Score  8.733200] GBROWSE*******************

 CG7527 in-situ | CadN2 | - | -34913 | -60912 | UPSTREAM | CG7527-RA | "-"
 CG5674 in-situ | CG5674 | + | 115821 | 127353 | UPSTREAM | CG5674-RA | "-" | CG5674-RB | "-" | CG5674-RC | "-"

*********************** Rank 332 [Score  8.731300] GBROWSE*******************

 CG10249 in-situ | BcDNA:GH03482 | - | -2557 | -13299 | UPSTREAM | CG10249-RC | "-" | CG10249-RA | "-" | CG10249-RB | "-"
insitu CG10253 in-situ | CG10253 | - | 18526 | 15307 | DOWNSTREAM | CG10253-RA | "-"

*********************** Rank 333 [Score  8.726000] GBROWSE*******************

 CG31209 in-situ | CG31209 | + | -11196 | 8268 | INTRAGENIC | intron:CG31209-RA:3 | CG31209-RA | "-"
 CG5060 in-situ | CG5060 | + | 16372 | 54707 | UPSTREAM | CG5060-RA | "-"

*********************** Rank 334 [Score  8.721300] GBROWSE*******************

 CG31685 in-situ | CG31685 | - | -80535 | -81414 | UPSTREAM | CG31685-RA | "-"
 CG12617 in-situ | CG12617 | + | 43798 | 44444 | UPSTREAM | CG12617-RA | "-"

*********************** Rank 335 [Score  8.718600] GBROWSE*******************

 CG8756 in-situ | CG8756 | - | -3112 | -7837 | UPSTREAM | CG8756-RB | "-" | CG8756-RA | "-" | CG8756-RC | "-" | CG8756-RD | "-"
insitu CG32209 in-situ | CG32209 | - | 9666 | 2634 | DOWNSTREAM | CG32209-RB | "-"

*********************** Rank 336 [Score  8.714700] GBROWSE*******************

 CG32062 in-situ | CG32062 | + | -11666 | 65750 | INTRAGENIC | intron:CG32062-RB:2 | intron:CG32062-RD:2 | CG32062-RB | "-" | CG32062-RD | "-"
 CG6527 in-situ | CG6527 | - | 31378 | 30494 | DOWNSTREAM | CG6527-RA | "-"

*********************** Rank 337 [Score  8.714400] GBROWSE*******************

 CG7855 in-situ | timeout | + | -27158 | 48067 | INTRAGENIC | intron:CG7855-RA:11 | CG7855-RA | "-"
 CG17319 in-situ | CG17319 | - | 12740 | 10302 | DOWNSTREAM | CG17319-RA | "-"

*********************** Rank 338 [Score  8.712300] GBROWSE*******************

 CG5897 in-situ | CG5897 | - | -11580 | -14367 | UPSTREAM | CG5897-RA | "-"
 CG11588 in-situ | CG11588 | + | 19314 | 20199 | UPSTREAM | CG11588-RA | "-"

*********************** Rank 339 [Score  8.703900] GBROWSE*******************

 CG12541 in-situ | CG12541 | - | -6545 | -8499 | UPSTREAM | CG12541-RA | "-"
insitu highlight CG14427 in-situ | CG14427 | + | 484 | 1645 | UPSTREAM | CG14427-RA | "-"

*********************** Rank 340 [Score  8.701000] GBROWSE*******************

 CG12589 in-situ | CG12589 | - | -1954 | -2244 | UPSTREAM | CG12589-RA | "-"
 CG12591 in-situ | CG12591 | + | 11477 | 30696 | UPSTREAM | CG12591-RA | "-"

*********************** Rank 341 [Score  8.700100] GBROWSE*******************

 CG7727 in-situ | Appl | + | -12043 | 34476 | INTRAGENIC | intron:CG7727-RA:1 | CG7727-RA | "-"
insitu highlight CG6172 in-situ | vnd | + | 47379 | 54159 | UPSTREAM | CG6172-RA | "-"

*********************** Rank 342 [Score  8.696400] GBROWSE*******************

 CG12433 in-situ | CG12433 | + | -22920 | -21446 | DOWNSTREAM | CG12433-RA | "-"
 CG8949 in-situ | CG8949 | - | 29607 | 24401 | DOWNSTREAM | CG8949-RA | "-"

*********************** Rank 343 [Score  8.691800] GBROWSE*******************

 CG5194 in-situ | CG5194 | - | -6639 | -7866 | UPSTREAM | CG5194-RA | "-"
insitu highlight CG5187 in-situ | Doc2 | - | 2750 | -3805 | INTRAGENIC | intron:CG5187-RA:2 | CG5187-RA | "-"

*********************** Rank 344 [Score  8.689500] GBROWSE*******************

 CG32175 in-situ | CG32175 | + | -9410 | -8760 | DOWNSTREAM | CG32175-RA | "-"
 CG6512 in-situ | CG6512 | - | 36003 | 32497 | DOWNSTREAM | CG6512-RA | "-" | CG6512-RB | "-"

*********************** Rank 345 [Score  8.689500] GBROWSE*******************

insitu CG1945 in-situ | faf | + | -12128 | 869 | INTRAGENIC | intron:CG1945-RA:17 | CG1945-RA | "-" | CG1945-RC | "-"
 CG2118 in-situ | CG2118 | - | 4744 | 1784 | DOWNSTREAM | CG2118-RB | "-" | CG2118-RA | "-"


*********************** Rank 346 [Score  8.688000] GBROWSE*******************

 CG1867 in-situ | Or98b | + | -65170 | -63792 | DOWNSTREAM | CG1867-RA | "-"
 CG14064 in-situ | beat-VI | + | 792 | 55797 | UPSTREAM | CG14064-RA | "-"

*********************** Rank 347 [Score  8.682300] GBROWSE*******************

 CG13646 in-situ | CG13646 | + | -15789 | -13882 | DOWNSTREAM | CG13646-RA | "-"
 CG13647 in-situ | CG13647 | + | 7041 | 8639 | UPSTREAM | CG13647-RA | "-"


*********************** Rank 348 [Score  8.680600] GBROWSE*******************

 CG6140 in-situ | CG6140 | - | -5611 | -7353 | UPSTREAM | CG6140-RA | "-"
 CG32090 in-situ | CG32090 | + | 2490 | 2966 | UPSTREAM | CG32090-RA | "-"

*********************** Rank 349 [Score  8.677600] GBROWSE*******************

 CG14363 in-situ | CG14363 | - | -20280 | -23257 | UPSTREAM | CG14363-RA | "-"
 CG17956 in-situ | Mst87F | - | 16451 | 15886 | DOWNSTREAM | CG17956-RA | "-"


*********************** Rank 350 [Score  8.677200] GBROWSE*******************

 CG11517 in-situ | CG11517 | + | -25587 | -25061 | DOWNSTREAM | CG11517-RA | "-"
 CG2005 in-situ | Ptp99A | + | 1257 | 30819 | UPSTREAM | CG2005-RA | "-" | CG2005-RB | "-"

*********************** Rank 351 [Score  8.669900] GBROWSE*******************

 CG3653 in-situ | kirre | + | -16734 | 15991 | INTRAGENIC | intron:CG3653-RB:1 | CG3653-RB | "-" | CG3653-RA | "-"
 CG3936 in-situ | N | + | 18060 | 55450 | UPSTREAM | CG3936-RA | "-"

*********************** Rank 352 [Score  8.669000] GBROWSE*******************

 CG12754 in-situ | Or42b | - | -26267 | -27584 | UPSTREAM | CG12754-RA | "-"
 CG7796 in-situ | CG7796 | + | 22182 | 28562 | UPSTREAM | CG7796-RA | "-"

*********************** Rank 353 [Score  8.666100] GBROWSE*******************

 CG15541 in-situ | CG15541 | + | -29095 | -22551 | DOWNSTREAM | CG15541-RA | "-"
 CG1342 in-situ | CG1342 | + | 12382 | 14506 | UPSTREAM | CG1342-RA | "-"


*********************** Rank 354 [Score  8.663300] GBROWSE*******************

 CG13921 in-situ | CG13921 | + | -8211 | -2726 | DOWNSTREAM | CG13921-RA | "-"
 CG2054 in-situ | Cht2 | - | 542 | -2575 | INTRAGENIC | intron:CG2054-RA:1 | CG2054-RA | "-"

*********************** Rank 355 [Score  8.649900] GBROWSE*******************

 CG13716 in-situ | CG13716 | - | -220 | -573 | UPSTREAM | CG13716-RA | "-"
 CG13715 in-situ | CG13715 | - | 2736 | 2425 | DOWNSTREAM | CG13715-RA | "-"


*********************** Rank 356 [Score  8.641000] GBROWSE*******************

 CG4626 in-situ | fz4 | - | -1782 | -5802 | UPSTREAM | CG4626-RB | "-" | CG4626-RA | "-"
 CG32729 in-situ | CG32729 | + | 77655 | 78262 | UPSTREAM | CG32729-RA | "-"

*********************** Rank 357 [Score  8.640400] GBROWSE*******************

 CG5423 in-situ | robo3 | + | -5708 | 31301 | INTRAGENIC | intron:CG5423-RA:1 | CG5423-RA | "-"
 CG5430 in-situ | a5 | + | 66627 | 67370 | UPSTREAM | CG5430-RA | "-"

*********************** Rank 358 [Score  8.630100] GBROWSE*******************

 CG14886 in-situ | CG14886 | + | -439 | 3262 | INTRAGENIC | intron:CG14886-RA:1 | CG14886-RA | "-"
 CG14887 in-situ | Dhfr | + | 3424 | 4069 | UPSTREAM | CG14887-RA | "-"

*********************** Rank 359 [Score  8.628700] GBROWSE*******************

 CG32461 in-situ | CG32461 | - | -9587 | -10345 | UPSTREAM | CG32461-RA | "-"
 CG32457 in-situ | CG32457 | + | 22638 | 23288 | UPSTREAM | CG32457-RA | "-"

*********************** Rank 360 [Score  8.621800] GBROWSE*******************

 CG30158 in-situ | CG30158 | + | -5635 | 25215 | INTRAGENIC | intron:CG30158-RA:2 | CG30158-RA | "-"
 CG3358 in-situ | CG3358 | + | 25973 | 27283 | UPSTREAM | CG3358-RA | "-" | CG3358-RB | "-"

*********************** Rank 361 [Score  8.615900] GBROWSE*******************

 CG2841 in-situ | ptr | - | -20363 | -29495 | UPSTREAM | CG2841-RB | "-" | CG2841-RC | "-" | CG2841-RA | "-"
 CG14047 in-situ | EG:BACH48C10.4 | - | 1144 | -6070 | INTRAGENIC | intron:CG14047-RA:2 | CG14047-RA | "-"

*********************** Rank 362 [Score  8.614400] GBROWSE*******************

 CG8590 in-situ | Klp3A | - | -2126 | -7021 | UPSTREAM | CG8590-RA | "-"
 CG13761 in-situ | Bzd | - | 2489 | 501 | DOWNSTREAM | CG13761-RB | "-"

*********************** Rank 363 [Score  8.606100] GBROWSE*******************

insitu CG12214 in-situ | CG12214 | - | -53537 | -56047 | UPSTREAM | CG12214-RB | "-" | CG12214-RA | "-"
insitu CG33183 in-situ | Hr46 | - | 20974 | -6981 | INTRAGENIC | intron:CG33183-RC:1 | intron:CG33183-RB:1 | intron:CG33183-RA:1 | CG33183-RC | "-" | CG33183-RB | "-" | CG33183-RA | "-"

*********************** Rank 364 [Score  8.605300] GBROWSE*******************

insitu CG31795 in-situ | ia2 | - | -5581 | -42213 | UPSTREAM | CG31795-RB | "-" | CG31795-RA | "-"
 CG4385 in-situ | S | - | 24177 | -2757 | INTRAGENIC | intron:CG4385-RB:2 | intron:CG4385-RA:2 | CG4385-RB | "-" | CG4385-RA | "-"

*********************** Rank 365 [Score  8.604800] GBROWSE*******************

 CG8929 in-situ | CG8929 | + | -6621 | -3164 | DOWNSTREAM | CG8929-RC | "-" | CG8929-RB | "-" | CG8929-RA | "-"
 CG16739 in-situ | CG16739 | + | 8140 | 8886 | UPSTREAM | CG16739-RA | "-"

*********************** Rank 366 [Score  8.599700] GBROWSE*******************

insitu CG15281 in-situ | BG:DS00810.3 | + | -5313 | -4706 | DOWNSTREAM | CG15281-RA | "-" | CG15281-RB | "-"
 CG4691 in-situ | BG:DS06874.1 | + | 12201 | 13367 | UPSTREAM | CG4691-RA | "-"

*********************** Rank 367 [Score  8.598400] GBROWSE*******************

 CG7798 in-situ | CG7798 | - | -3648 | -4094 | UPSTREAM | CG7798-RA | "-"
 CG3666 in-situ | Tsf3 | + | 251 | 3599 | UPSTREAM | CG3666-RA | "-"


*********************** Rank 368 [Score  8.597600] GBROWSE*******************

 CG7968 in-situ | BG:DS00941.15 | + | -22686 | -21771 | DOWNSTREAM | CG7968-RA | "-"
insitu CG8954 in-situ | CG8954 | - | 32561 | 27255 | DOWNSTREAM | CG8954-RA | "-" | CG8954-RB | "-"

*********************** Rank 369 [Score  8.591700] GBROWSE*******************

 CG30428 in-situ | CG30428 | + | 20265784 | 20266947 | UPSTREAM | CG30428-RA | "-"
 CG2981 in-situ | TpnC41C | - | 177884 | 174061 | DOWNSTREAM | CG2981-RA | "-"

*********************** Rank 370 [Score  8.588500] GBROWSE*******************

 CG15296 in-situ | CG15296 | - | -2191 | -2855 | UPSTREAM | CG15296-RA | "-"
 CG32674 in-situ | CG32674 | - | 7487 | 6696 | DOWNSTREAM | CG32674-RA | "-"

*********************** Rank 371 [Score  8.585300] GBROWSE*******************

insitu CG7920 in-situ | CG7920 | - | -5148 | -10045 | UPSTREAM | CG7920-RA | "-" | CG7920-RB | "-"
insitu CG7921 in-situ | Mgat2 | - | 1746 | -5007 | INTRAGENIC | intron:CG7921-RA:1 | CG7921-RA | "-"

*********************** Rank 372 [Score  8.585000] GBROWSE*******************

insitu highlight CG2368 in-situ | psq | + | -24063 | 34288 | INTRAGENIC | intron:CG2368-RB:3 | intron:CG2368-RA:3 | intron:CG2368-RE:2 | intron:CG2368-RF:2 | intron:CG2368-RG:2 | CG2368-RB | "-" | CG2368-RA | "-" | CG2368-RE | "-" | CG2368-RF | "-" | CG2368-RG | "-" | CG2368-RD | "-" | CG2368-RH | "-"
 CG11883 in-situ | CG11883 | + | 35697 | 63340 | UPSTREAM | CG11883-RB | "-" | CG11883-RA | "-"

*********************** Rank 373 [Score  8.581800] GBROWSE*******************

 CG6746 in-situ | CG6746 | - | -5953 | -6995 | UPSTREAM | CG6746-RA | "-"
 CG5279 in-situ | Rh5 | + | 163 | 1617 | UPSTREAM | CG5279-RA | "-"

*********************** Rank 374 [Score  8.581700] GBROWSE*******************

 CG9709 in-situ | Acox57D-d | + | -84559 | -81509 | DOWNSTREAM | CG9709-RA | "-"
 CG10497 in-situ | Sdc | - | 7219 | -80332 | INTRAGENIC | intron:CG10497-RC:2 | intron:CG10497-RA:2 | intron:CG10497-RB:2 | CG10497-RC | "-" | CG10497-RA | "-" | CG10497-RB | "-"

*********************** Rank 375 [Score  8.579500] GBROWSE*******************

 CG31311 in-situ | CG31311 | - | -5024 | -6833 | UPSTREAM | CG31311-RA | "-" | CG31311-RB | "-" | CG31311-RC | "-" | CG31311-RE | "-" | CG31311-RD | "-"
 CG9602 in-situ | CG9602 | - | 1307 | 591 | DOWNSTREAM | CG9602-RA | "-"

*********************** Rank 376 [Score  8.578400] GBROWSE*******************

 CG6335 in-situ | CG6335 | + | -5142 | -94 | DOWNSTREAM | CG6335-RA | "-" | CG6335-RB | "-"
 CG15048 in-situ | CG15048 | + | 118 | 696 | UPSTREAM | CG15048-RA | "-"

*********************** Rank 377 [Score  8.575600] GBROWSE*******************

 CG9413 in-situ | CG9413 | - | -40906 | -58747 | UPSTREAM | CG9413-RA | "-" | CG9413-RB | "-"
insitu CG18657 in-situ | NetA | - | 10346 | -39483 | INTRAGENIC | intron:CG18657-RA:1 | CG18657-RA | "-"

*********************** Rank 378 [Score  8.569500] GBROWSE*******************

insitu highlight CG2988 in-situ | ems | + | -25350 | -22585 | DOWNSTREAM | CG2988-RA | "-"
 CG9929 in-situ | CG9929 | - | 16193 | 15139 | DOWNSTREAM | CG9929-RA | "-"

*********************** Rank 379 [Score  8.569200] GBROWSE*******************

 CG11312 in-situ | insc | - | -4242 | -19040 | UPSTREAM | CG11312-RA | "-"
 CG17999 in-situ | CG17999 | - | 9613 | 7674 | DOWNSTREAM | CG17999-RA | "-"

*********************** Rank 380 [Score  8.566500] GBROWSE*******************

 CG10277 in-situ | CG10277 | + | -17817 | -12424 | DOWNSTREAM | CG10277-RA | "-" | CG10277-RB | "-" | CG10277-RC | "-"
 CG1021 in-situ | CG1021 | - | 5163 | -11569 | INTRAGENIC | intron:CG1021-RA:1 | intron:CG1021-RB:1 | CG1021-RA | "-" | CG1021-RB | "-"

*********************** Rank 381 [Score  8.565600] GBROWSE*******************

insitu CG10191 in-situ | CG10191 | - | -4994 | -6689 | UPSTREAM | CG10191-RA | "-"
 CG32120 in-situ | Ly | - | 6143 | 1247 | DOWNSTREAM | CG32120-RA | "-"

*********************** Rank 382 [Score  8.564600] GBROWSE*******************

 CG1367 in-situ | CecA2 | + | -474 | -208 | DOWNSTREAM | CG1367-RA | "-"
 CG32924 in-situ | Cec2 | + | 191 | 430 | UPSTREAM | CG32924-RA | "-"

*********************** Rank 383 [Score  8.560700] GBROWSE*******************

 CG13109 in-situ | tai | + | -71467 | 8121 | INTRAGENIC | intron:CG13109-RA:4 | CG13109-RA | "-"
 CG17009 in-situ | CG17009 | - | 12743 | 12027 | DOWNSTREAM | CG17009-RA | "-"

*********************** Rank 384 [Score  8.540500] GBROWSE*******************

insitu highlight CG7952 in-situ | gt | - | -165 | -2021 | UPSTREAM | CG7952-RB | "-"
 CG7925 in-situ | tko | - | 14848 | 13179 | DOWNSTREAM | CG7925-RB | "-"


*********************** Rank 385 [Score  8.540200] GBROWSE*******************

 CG18210 in-situ | CG18210 | - | -30328 | -33397 | UPSTREAM | CG18210-RA | "-"
 CG9195 in-situ | Scamp | - | 8453 | 4742 | DOWNSTREAM | CG9195-RA | "-" | CG9195-RB | "-"

*********************** Rank 386 [Score  8.521800] GBROWSE*******************

 CG2708 in-situ | Tom34 | + | -22308 | -18954 | DOWNSTREAM | CG2708-RA | "-"
 CG7918 in-situ | CG7918 | + | 3671 | 31432 | UPSTREAM | CG7918-RA | "-"

*********************** Rank 387 [Score  8.517600] GBROWSE*******************

insitu highlight CG1849 in-situ | run | + | -23894 | -21009 | DOWNSTREAM | CG1849-RA | "-"
 CG1324 in-situ | CG1324 | - | 17625 | 16409 | DOWNSTREAM | CG1324-RA | "-"

*********************** Rank 388 [Score  8.515700] GBROWSE*******************

 CG7423 in-situ | CG7423 | - | -15512 | -15886 | UPSTREAM | CG7423-RA | "-"
 CG15882 in-situ | CG15882 | - | 25586 | 25110 | DOWNSTREAM | CG15882-RA | "-"

*********************** Rank 389 [Score  8.509200] GBROWSE*******************

 CG18210 in-situ | CG18210 | - | -7678 | -10747 | UPSTREAM | CG18210-RA | "-"
 CG9195 in-situ | Scamp | - | 31103 | 27392 | DOWNSTREAM | CG9195-RA | "-" | CG9195-RB | "-"

*********************** Rank 390 [Score  8.505300] GBROWSE*******************

insitu CG11337 in-situ | CG11337 | - | -4710 | -8327 | UPSTREAM | CG11337-RB | "-" | CG11337-RA | "-"
 CG11335 in-situ | lox | - | 49565 | 48422 | DOWNSTREAM | CG11335-RA | "-"


*********************** Rank 391 [Score  8.503200] GBROWSE*******************

insitu highlight CG3136 in-situ | CG3136 | + | -2133 | 7931 | INTRAGENIC | intron:CG3136-RA:1 | CG3136-RA | "-" | CG3136-RB | "-"
 CG14768 in-situ | CG14768 | + | 30271 | 30606 | UPSTREAM | CG14768-RA | "-"

*********************** Rank 392 [Score  8.501200] GBROWSE*******************

insitu CG10067 in-situ | Act57B | + | -13078 | -10666 | DOWNSTREAM | CG10067-RA | "-"
 CG33152 in-situ | hbn | - | 3803 | -2442 | INTRAGENIC | intron:CG33152-RA:3 | CG33152-RA | "-"

*********************** Rank 393 [Score  8.498400] GBROWSE*******************

 CG3647 in-situ | stc | + | -48133 | -43545 | DOWNSTREAM | CG3647-RB | "-" | CG3647-RA | "-"
 CG4168 in-situ | BG:DS03192.2 | - | 16705 | 492 | DOWNSTREAM | CG4168-RA | "-"

*********************** Rank 394 [Score  8.497600] GBROWSE*******************

 CG31686 in-situ | CG31686 | + | -4010 | -3426 | DOWNSTREAM | CG31686-RA | "-"
 CG17240 in-situ | Ser12 | - | 2587 | 1850 | DOWNSTREAM | CG17240-RA | "-"

*********************** Rank 395 [Score  8.496800] GBROWSE*******************

 CG31786 in-situ | CG31786 | - | -24114 | -30228 | UPSTREAM | CG31786-RA | "-"
 CG31788 in-situ | CG31788 | - | 17925 | 17526 | DOWNSTREAM | CG31788-RA | "-"

*********************** Rank 396 [Score  8.496800] GBROWSE*******************

 CG6391 in-situ | CG6391 | - | -12974 | -16674 | UPSTREAM | CG6391-RA | "-" | CG6391-RB | "-" | CG6391-RC | "-"
 CG14148 in-situ | CG14148 | - | 28195 | 27905 | DOWNSTREAM | CG14148-RA | "-"

*********************** Rank 397 [Score  8.486300] GBROWSE*******************

 CG14390 in-situ | beat-Vc | - | -25008 | -33094 | UPSTREAM | CG14390-RA | "-"
 CG31345 in-situ | CG31345 | - | 37320 | 31636 | DOWNSTREAM | CG31345-RA | "-"


*********************** Rank 398 [Score  8.485100] GBROWSE*******************

 CG30153 in-situ | CG30153 | + | -15139 | -14483 | DOWNSTREAM | CG30153-RA | "-"
 CG30147 in-situ | CG30147 | - | 6226 | -5107 | INTRAGENIC | intron:CG30147-RB:4 | intron:CG30147-RA:4 | CG30147-RB | "-" | CG30147-RA | "-"

*********************** Rank 399 [Score  8.484300] GBROWSE*******************

 CG10363 in-situ | TepIV | + | -1424 | 5238 | INTRAGENIC | intron:CG10363-RA:1 | CG10363-RA | "-"
 CG13079 in-situ | CG13079 | + | 6830 | 11832 | UPSTREAM | CG13079-RA | "-"

*********************** Rank 400 [Score  8.484300] GBROWSE*******************

 CG31183 in-situ | CG31183 | - | -17208 | -29181 | UPSTREAM | CG31183-RA | "-"
 CG14877 in-situ | CG14877 | - | 2855 | 1934 | DOWNSTREAM | CG14877-RA | "-"


*********************** Rank 401 [Score  8.483200] GBROWSE*******************

 CG3757 in-situ | y | + | -6403 | -1667 | DOWNSTREAM | CG3757-RA | "-"
 CG3796 in-situ | ac | + | 7119 | 8079 | UPSTREAM | CG3796-RA | "-"


*********************** Rank 402 [Score  8.478300] GBROWSE*******************

 CG17681 in-situ | CG17681 | + | -1738 | 10143 | INTRAGENIC | intron:CG17681-RA:1 | CG17681-RA | "-" | CG17681-RB | "-"
 CG15154 in-situ | Socs36E | - | 8143 | -5599 | INTRAGENIC | intron:CG15154-RA:1 | intron:CG15154-RB:1 | CG15154-RA | "-" | CG15154-RB | "-"

*********************** Rank 403 [Score  8.475600] GBROWSE*******************

insitu CG7097 in-situ | CG7097 | + | -26476 | 21970 | INTRAGENIC | intron:CG7097-RB:2 | intron:CG7097-RA:2 | CG7097-RB | "-" | CG7097-RA | "-"
 CG7137 in-situ | CG7137 | + | 22100 | 23387 | UPSTREAM | CG7137-RA | "-"

*********************** Rank 404 [Score  8.459700] GBROWSE*******************

 CG14707 in-situ | CG14707 | - | -2013 | -2198 | UPSTREAM | CG14707-RA | "-"
 CG14708 in-situ | CG14708 | + | 12929 | 13546 | UPSTREAM | CG14708-RA | "-"

*********************** Rank 405 [Score  8.457200] GBROWSE*******************

insitu CG12214 in-situ | CG12214 | - | -35387 | -37897 | UPSTREAM | CG12214-RB | "-" | CG12214-RA | "-"
insitu CG33183 in-situ | Hr46 | - | 39124 | 11169 | DOWNSTREAM | CG33183-RC | "-" | CG33183-RB | "-" | CG33183-RA | "-"

*********************** Rank 406 [Score  8.453800] GBROWSE*******************

 CG32600 in-situ | CG32600 | + | -16260 | 123885 | INTRAGENIC | intron:CG32600-RA:2 | CG32600-RA | "-"
 CG32601 in-situ | CG32601 | + | 40390 | 41339 | UPSTREAM | CG32601-RA | "-"

*********************** Rank 407 [Score  8.449900] GBROWSE*******************

insitu CG32137 in-situ | CG32137 | + | -3918 | 19479 | INTRAGENIC | intron:CG32137-RA:2 | intron:CG32137-RB:2 | CG32137-RA | "-" | CG32137-RB | "-"
 CG8474 in-situ | Meics | - | 21523 | 19484 | DOWNSTREAM | CG8474-RA | "-"


*********************** Rank 408 [Score  8.447900] GBROWSE*******************

insitu CG17508 in-situ | CG17508 | + | -4105 | -1140 | DOWNSTREAM | CG17508-RA | "-"
 CG11665 in-situ | CG11665 | - | 40054 | 28926 | DOWNSTREAM | CG11665-RA | "-"


*********************** Rank 409 [Score  8.444500] GBROWSE*******************

 CG31353 in-situ | CG31353 | - | -29415 | -29720 | UPSTREAM | CG31353-RA | "-"
 CG6332 in-situ | CG6332 | + | 6183 | 7707 | UPSTREAM | CG6332-RA | "-"

*********************** Rank 410 [Score  8.443400] GBROWSE*******************

 CG4871 in-situ | ST6Gal | - | -1234 | -3367 | UPSTREAM | CG4871-RB | "-" | CG4871-RA | "-"
 CG13595 in-situ | CG13595 | - | 6794 | 4562 | DOWNSTREAM | CG13595-RA | "-"

*********************** Rank 411 [Score  8.437200] GBROWSE*******************

 CG1631 in-situ | CG1631 | + | -29208 | -28021 | DOWNSTREAM | CG1631-RA | "-"
 CG15462 in-situ | CG15462 | - | 11248 | 10073 | DOWNSTREAM | CG15462-RA | "-"


*********************** Rank 412 [Score  8.436400] GBROWSE*******************

 CG3849 in-situ | CG3849 | - | -418 | -34445 | UPSTREAM | CG3849-RA | "-"
 CG9695 in-situ | Dab | + | 1072 | 11919 | UPSTREAM | CG9695-RA | "-"

*********************** Rank 413 [Score  8.418400] GBROWSE*******************

 CG17330 in-situ | BG:DS09218.5 | + | -5647 | -4589 | DOWNSTREAM | CG17330-RA | "-"
 CG4472 in-situ | Idgf1 | + | 73714 | 75281 | UPSTREAM | CG4472-RA | "-"

*********************** Rank 414 [Score  8.414600] GBROWSE*******************

 CG31270 in-situ | CG31270 | - | -17628 | -18744 | UPSTREAM | CG31270-RA | "-"
 CG11648 in-situ | Abd-B | - | 22532 | -13740 | INTRAGENIC | intron:CG11648-RC:2 | intron:CG11648-RA:2 | intron:CG11648-RD:2 | CG11648-RC | "-" | CG11648-RA | "-" | CG11648-RD | "-" | CG11648-RB | "-"

*********************** Rank 415 [Score  8.413200] GBROWSE*******************

 CG31531 in-situ | CG31531 | + | -18746 | 26928 | INTRAGENIC | intron:CG31531-RA:3 | intron:CG31531-RC:3 | intron:CG31531-RB:2 | CG31531-RA | "-" | CG31531-RC | "-" | CG31531-RB | "-"
insitu CG31534 in-situ | CG31534 | + | 27817 | 33864 | UPSTREAM | CG31534-RA | "-" | CG31534-RB | "-"

*********************** Rank 416 [Score  8.408800] GBROWSE*******************

 CG5075 in-situ | CG5075 | + | -9144 | -6629 | DOWNSTREAM | CG5075-RA | "-"
 CG12404 in-situ | CG12404 | - | 12701 | 5818 | DOWNSTREAM | CG12404-RA | "-"

*********************** Rank 417 [Score  8.408300] GBROWSE*******************

 CG15352 in-situ | CG15352 | + | -1386 | -776 | DOWNSTREAM | CG15352-RA | "-"
 CG12660 in-situ | CG12660 | + | 3267 | 3847 | UPSTREAM | CG12660-RA | "-"

*********************** Rank 418 [Score  8.407500] GBROWSE*******************

 CG13070 in-situ | CG13070 | + | -733 | -350 | DOWNSTREAM | CG13070-RA | "-"
 CG13069 in-situ | CG13069 | + | 1009 | 1302 | UPSTREAM | CG13069-RA | "-"

*********************** Rank 419 [Score  8.406400] GBROWSE*******************

 CG4641 in-situ | CG4641 | + | -21709 | -18781 | DOWNSTREAM | CG4641-RA | "-"
 CG10537 in-situ | Rdl | - | 13114 | -13895 | INTRAGENIC | intron:CG10537-RA:3 | intron:CG10537-RB:3 | intron:CG10537-RC:3 | CG10537-RA | "-" | CG10537-RB | "-" | CG10537-RC | "-"

*********************** Rank 420 [Score  8.402500] GBROWSE*******************

 CG13596 in-situ | CG13596 | - | -3866 | -8356 | UPSTREAM | CG13596-RA | "-"
 CG18510 in-situ | CG18510 | - | 14673 | 3215 | DOWNSTREAM | CG18510-RA | "-"

*********************** Rank 421 [Score  8.401400] GBROWSE*******************

 CG17835 in-situ | inv | + | -34072 | -1354 | DOWNSTREAM | CG17835-RA | "-" | CG17835-RD | "-" | CG17835-RB | "-" | CG17835-RC | "-"
 CG30034 in-situ | CG30034 | - | 13511 | 7642 | DOWNSTREAM | CG30034-RA | "-"

*********************** Rank 422 [Score  8.397700] GBROWSE*******************

 CG18128 in-situ | CG18128 | - | -22296 | -23435 | UPSTREAM | CG18128-RA | "-"
insitu CG4051 in-situ | egl | - | 4593 | -7327 | INTRAGENIC | intron:CG4051-RA:2 | CG4051-RA | "-"

*********************** Rank 423 [Score  8.390300] GBROWSE*******************

 CG32119 in-situ | CG32119 | - | -2401 | -4240 | UPSTREAM | CG32119-RA | "-"
 CG17673 in-situ | Acp70A | + | 50553 | 50840 | UPSTREAM | CG17673-RA | "-"

*********************** Rank 424 [Score  8.384900] GBROWSE*******************

 CG7554 in-situ | comm2 | - | -21972 | -24230 | UPSTREAM | CG7554-RA | "-"
insitu highlight CG17943 in-situ | comm | - | 6602 | 503 | DOWNSTREAM | CG17943-RA | "-"

*********************** Rank 425 [Score  8.384100] GBROWSE*******************

 CG32266 in-situ | CG32266 | + | -1267 | -509 | DOWNSTREAM | CG32266-RA | "-"
insitu CG32264 in-situ | CG32264 | - | 9217 | -74874 | INTRAGENIC | intron:CG32264-RA:1 | CG32264-RA | "-" | CG32264-RD | "-" | CG32264-RB | "-"

*********************** Rank 426 [Score  8.378300] GBROWSE*******************

 CG1745 in-situ | CG1745 | + | -1720 | 1801 | INTRAGENIC | intron:CG1745-RB:2 | CG1745-RB | "-"
 CG15194 in-situ | CG15194 | + | 3371 | 4288 | UPSTREAM | CG15194-RA | "-"

*********************** Rank 427 [Score  8.378200] GBROWSE*******************

 CG1262 in-situ | Acp62F | + | -21221 | -14068 | DOWNSTREAM | CG1262-RA | "-"
 CG32296 in-situ | CG32296 | - | 5509 | -41286 | INTRAGENIC | intron:CG32296-RA:1 | CG32296-RA | "-"

*********************** Rank 428 [Score  8.377000] GBROWSE*******************

insitu CG15509 in-situ | kay | + | -3283 | 17854 | INTRAGENIC | intron:CG15509-RA:1 | CG15509-RA | "-" | CG15509-RB | "-"
 CG7615 in-situ | CG7615 | - | 4190 | 3150 | DOWNSTREAM | CG7615-RA | "-"

*********************** Rank 429 [Score  8.376600] GBROWSE*******************

insitu highlight CG4702 in-situ | CG4702 | - | -20362 | -26982 | UPSTREAM | CG4702-RA | "-"
 CG10095 in-situ | CG10095 | - | 10036 | -9726 | INTRAGENIC | intron:CG10095-RA:3 | CG10095-RA | "-"

*********************** Rank 430 [Score  8.369800] GBROWSE*******************

 CG3389 in-situ | Cad88C | + | -7257 | -398 | DOWNSTREAM | CG3389-RA | "-"
 CG7886 in-situ | CG7886 | - | 553 | -15337 | INTRAGENIC | intron:CG7886-RA:2 | CG7886-RA | "-"

*********************** Rank 431 [Score  8.366200] GBROWSE*******************

 CG16983 in-situ | skpA | + | -11941 | -10109 | DOWNSTREAM | CG16983-RA | "-" | CG16983-RD | "-" | CG16983-RG | "-" | CG16983-RB | "-" | CG16983-RC | "-" | CG16983-RF | "-" | CG16983-RE | "-"
 CG5227 in-situ | sdk | + | 18827 | 81485 | UPSTREAM | CG5227-RD | "-" | CG5227-RC | "-" | CG5227-RB | "-" | CG5227-RA | "-"

*********************** Rank 432 [Score  8.363800] GBROWSE*******************

insitu CG3726 in-situ | CG3726 | + | -7897 | 16596 | INTRAGENIC | intron:CG3726-RA:1 | CG3726-RA | "-"
 CG12728 in-situ | CG12728 | + | 17534 | 19313 | UPSTREAM | CG12728-RA | "-"

*********************** Rank 433 [Score  8.362600] GBROWSE*******************

insitu CG15203 in-situ | CG15203 | + | -13481 | -11980 | DOWNSTREAM | CG15203-RA | "-"
 CG18085 in-situ | sev | - | 6238 | -8620 | INTRAGENIC | intron:CG18085-RA:2 | CG18085-RA | "-"

*********************** Rank 434 [Score  8.360800] GBROWSE*******************

 CG31738 in-situ | CG31738 | + | -6472 | 33510 | INTRAGENIC | intron:CG31738-RB:1 | CG31738-RB | "-" | CG31738-RA | "-"
 CG5996 in-situ | trpgamma | - | 48150 | 36345 | DOWNSTREAM | CG5996-RA | "-" | CG5996-RB | "-"

*********************** Rank 435 [Score  8.350600] GBROWSE*******************

 CG1031 in-situ | alpha-Est1 | - | -20237 | -23632 | UPSTREAM | CG1031-RA | "-"
 CG32465 in-situ | CG32465 | - | 11906 | 1931 | DOWNSTREAM | CG32465-RB | "-"

*********************** Rank 436 [Score  8.348700] GBROWSE*******************

insitu highlight CG10325 in-situ | abd-A | - | -34912 | -57338 | UPSTREAM | CG10325-RA | "-" | CG10325-RB | "-"
 CG10349 in-situ | CG10349 | + | 14765 | 20147 | UPSTREAM | CG10349-RA | "-" | CG10349-RB | "-"

*********************** Rank 437 [Score  8.346200] GBROWSE*******************

 CG8141 in-situ | CG8141 | + | -13308 | -12494 | DOWNSTREAM | CG8141-RA | "-"
 CG8483 in-situ | CG8483 | - | 9456 | 3739 | DOWNSTREAM | CG8483-RA | "-"

*********************** Rank 438 [Score  8.345200] GBROWSE*******************

 CG8705 in-situ | pnut | + | -11134 | -8073 | DOWNSTREAM | CG8705-RB | "-" | CG8705-RA | "-"
 CG14760 in-situ | CG14760 | + | 5853 | 8333 | UPSTREAM | CG14760-RA | "-"

*********************** Rank 439 [Score  8.344500] GBROWSE*******************

insitu highlight CG9598 in-situ | CG9598 | - | -69138 | -73647 | UPSTREAM | CG9598-RA | "-"
 CG9587 in-situ | CG9587 | - | 26778 | 21136 | DOWNSTREAM | CG9587-RA | "-"

*********************** Rank 440 [Score  8.342800] GBROWSE*******************

 CG32750 in-situ | CG32750 | - | -107 | -1736 | UPSTREAM | CG32750-RA | "-"
 CG32751 in-situ | CG32751 | - | 2105 | 200 | DOWNSTREAM | CG32751-RA | "-"

*********************** Rank 441 [Score  8.342700] GBROWSE*******************

 CG31184 in-situ | CG31184 | + | -27002 | -26392 | DOWNSTREAM | CG31184-RA | "-"
insitu CG5405 in-situ | KrT95D | - | 8389 | -24984 | INTRAGENIC | intron:CG5405-RB:2 | intron:CG5405-RA:1 | CG5405-RB | "-" | CG5405-RA | "-"

*********************** Rank 442 [Score  8.341500] GBROWSE*******************

 CG9310 in-situ | Hnf4 | - | -5931 | -27688 | UPSTREAM | CG9310-RA | "-" | CG9310-RB | "-" | CG9310-RC | "-"
 CG12437 in-situ | raw | - | 25664 | -5099 | INTRAGENIC | intron:CG12437-RB:1 | intron:CG12437-RA:2 | CG12437-RB | "-" | CG12437-RA | "-"

*********************** Rank 443 [Score  8.340900] GBROWSE*******************

 CG5060 in-situ | CG5060 | + | -4178 | 34157 | INTRAGENIC | intron:CG5060-RA:1 | CG5060-RA | "-"
 CG10883 in-situ | CG10883 | - | 31852 | 31319 | DOWNSTREAM | CG10883-RA | "-"

*********************** Rank 444 [Score  8.336500] GBROWSE*******************

insitu CG10698 in-situ | GRHRII | - | -31167 | -43591 | UPSTREAM | CG10698-RA | "-"
 CG4357 in-situ | CG4357 | - | 8712 | -14057 | INTRAGENIC | intron:CG4357-RA:1 | CG4357-RA | "-"

*********************** Rank 445 [Score  8.332500] GBROWSE*******************

 CG13964 in-situ | CG13964 | + | -8108 | -6702 | DOWNSTREAM | CG13964-RA | "-"
 CG10662 in-situ | CG10662 | + | 922 | 3979 | UPSTREAM | CG10662-RA | "-"

*********************** Rank 446 [Score  8.330500] GBROWSE*******************

 CG13598 in-situ | sba | - | -348 | -29154 | UPSTREAM | CG13598-RA | "-" | CG13598-RB | "-"
 CG31141 in-situ | CG31141 | + | 183 | 877 | UPSTREAM | CG31141-RA | "-"

*********************** Rank 447 [Score  8.327100] GBROWSE*******************

insitu CG7891 in-situ | CG7891 | + | -33999 | -31767 | DOWNSTREAM | CG7891-RA | "-"
 CG9656 in-situ | grn | - | 20829 | -13263 | INTRAGENIC | intron:CG9656-RA:5 | CG9656-RA | "-"

*********************** Rank 448 [Score  8.325400] GBROWSE*******************

 CG32396 in-situ | CG32396 | + | -8442 | -6742 | DOWNSTREAM | CG32396-RA | "-"
 CG18769 in-situ | CG18769 | + | 11568 | 54769 | UPSTREAM | CG18769-RA | "-" | CG18769-RC | "-" | CG18769-RB | "-"

*********************** Rank 449 [Score  8.320300] GBROWSE*******************

 CG32119 in-situ | CG32119 | - | -52351 | -54190 | UPSTREAM | CG32119-RA | "-"
 CG17673 in-situ | Acp70A | + | 603 | 890 | UPSTREAM | CG17673-RA | "-"

*********************** Rank 450 [Score  8.318400] GBROWSE*******************

 CG18405 in-situ | Sema-1a | + | -24973 | 78660 | INTRAGENIC | intron:CG18405-RA:1 | CG18405-RA | "-"
 CG9280 in-situ | Glt | + | 83274 | 88042 | UPSTREAM | CG9280-RC | "-" | CG9280-RB | "-" | CG9280-RA | "-"

*********************** Rank 451 [Score  8.317600] GBROWSE*******************

 CG7361 in-situ | RFeSP | - | -18102 | -19598 | UPSTREAM | CG7361-RA | "-" | CG7361-RB | "-"
insitu CG31666 in-situ | CG31666 | + | 20977 | 66344 | UPSTREAM | CG31666-RD | "-" | CG31666-RA | "-" | CG31666-RB | "-" | CG31666-RC | "-"

*********************** Rank 452 [Score  8.308900] GBROWSE*******************

 CG1303 in-situ | agt | - | -6377 | -7071 | UPSTREAM | CG1303-RA | "-"
insitu highlight CG1264 in-situ | lab | - | 12113 | -5059 | INTRAGENIC | intron:CG1264-RA:1 | CG1264-RA | "-"

*********************** Rank 453 [Score  8.308000] GBROWSE*******************

 CG9922 in-situ | CG9922 | - | -9044 | -10193 | UPSTREAM | CG9922-RA | "-"
 CG3143 in-situ | foxo | + | 11763 | 42191 | UPSTREAM | CG3143-RA | "-"

*********************** Rank 454 [Score  8.300200] GBROWSE*******************

 CG15485 in-situ | CG15485 | - | -12661 | -14565 | UPSTREAM | CG15485-RA | "-"
insitu CG5525 in-situ | CG5525 | - | 11107 | 8495 | DOWNSTREAM | CG5525-RA | "-"

*********************** Rank 455 [Score  8.298000] GBROWSE*******************

 CG31774 in-situ | fred | - | -9271 | -50918 | UPSTREAM | CG31774-RA | "-"
 CG15422 in-situ | CG15422 | + | 48625 | 48939 | UPSTREAM | CG15422-RA | "-"

*********************** Rank 456 [Score  8.296900] GBROWSE*******************

 CG31085 in-situ | CG31085 | + | -2558 | 10526 | INTRAGENIC | intron:CG31085-RB:3 | CG31085-RB | "-" | CG31085-RA | "-"
 CG14239 in-situ | CG14239 | - | 51325 | 50446 | DOWNSTREAM | CG14239-RA | "-"

*********************** Rank 457 [Score  8.292700] GBROWSE*******************

 CG14597 in-situ | CG14597 | - | -51168 | -51809 | UPSTREAM | CG14597-RA | "-"
 CG31146 in-situ | CG31146 | + | 3340 | 39409 | UPSTREAM | CG31146-RD | "-"

*********************** Rank 458 [Score  8.291300] GBROWSE*******************

insitu highlight CG15085 in-situ | mae | - | -7498 | -13509 | UPSTREAM | CG15085-RA | "-"
insitu CG15086 in-situ | CG15086 | - | 5586 | -2350 | INTRAGENIC | intron:CG15086-RA:8 | intron:CG15086-RB:6 | intron:CG15086-RD:8 | intron:CG15086-RC:3 | CG15086-RA | "-" | CG15086-RB | "-" | CG15086-RD | "-" | CG15086-RC | "-"

*********************** Rank 459 [Score  8.291000] GBROWSE*******************

 CG12835 in-situ | CG12835 | + | -8556 | -7570 | DOWNSTREAM | CG12835-RA | "-"
 CG4485 in-situ | Cyp9b1 | - | 14309 | 12339 | DOWNSTREAM | CG4485-RA | "-"

*********************** Rank 460 [Score  8.287400] GBROWSE*******************

insitu CG10138 in-situ | PpD5 | - | -417 | -1632 | UPSTREAM | CG10138-RA | "-"
 CG13500 in-situ | CG13500 | - | 14382 | 13168 | DOWNSTREAM | CG13500-RA | "-"

*********************** Rank 461 [Score  8.284100] GBROWSE*******************

 CG1447 in-situ | Ptx1 | + | -9824 | 7970 | INTRAGENIC | intron:CG1447-RA:2 | CG1447-RA | "-" | CG1447-RB | "-"
 CG15549 in-situ | CG15549 | - | 13795 | 12684 | DOWNSTREAM | CG15549-RA | "-"

*********************** Rank 462 [Score  8.279500] GBROWSE*******************

insitu CG3831 in-situ | CG3831 | - | -1888 | -3847 | UPSTREAM | CG3831-RA | "-"
 CG13534 in-situ | CG13534 | + | 3906 | 4871 | UPSTREAM | CG13534-RA | "-"

*********************** Rank 463 [Score  8.279200] GBROWSE*******************

insitu CG1150 in-situ | CG1150 | + | -3592 | -1475 | DOWNSTREAM | CG1150-RA | "-"
 CG10303 in-situ | CG10303 | - | 2353 | -1110 | INTRAGENIC | intron:CG10303-RA:3 | CG10303-RA | "-"

*********************** Rank 464 [Score  8.271400] GBROWSE*******************

 CG31708 in-situ | CG31708 | - | -2007 | -21545 | UPSTREAM | CG31708-RB | "-" | CG31708-RA | "-"
 CG12439 in-situ | CG12439 | + | 3952 | 4819 | UPSTREAM | CG12439-RA | "-"

*********************** Rank 465 [Score  8.271400] GBROWSE*******************

 CG13353 in-situ | CG13353 | + | -27210 | -25859 | DOWNSTREAM | CG13353-RA | "-"
 CG30483 in-situ | Prosap | - | 23369 | -56107 | INTRAGENIC | intron:CG30483-RA:1 | CG30483-RA | "-"

*********************** Rank 466 [Score  8.270100] GBROWSE*******************

 CG14340 in-situ | CG14340 | + | -11596 | -10677 | DOWNSTREAM | CG14340-RA | "-"
insitu highlight CG4710 in-situ | CG4710 | - | 4369 | -9932 | INTRAGENIC | intron:CG4710-RB:2 | CG4710-RB | "-" | CG4710-RA | "-"

*********************** Rank 467 [Score  8.268200] GBROWSE*******************

 CG3690 in-situ | EG:BACR7A4.13 | - | -715 | -3273 | UPSTREAM | CG3690-RA | "-"
 CG18823 in-situ | CG18823 | + | 38019 | 38339 | UPSTREAM | CG18823-RA | "-"

*********************** Rank 468 [Score  8.267800] GBROWSE*******************

 CG4374 in-situ | CG4374 | - | -10814 | -15166 | UPSTREAM | CG4374-RA | "-"
 CG31225 in-situ | CG31225 | + | 58546 | 60120 | UPSTREAM | CG31225-RA | "-"

*********************** Rank 469 [Score  8.267200] GBROWSE*******************

 CG31612 in-situ | CG31612 | - | -20384 | -53540 | UPSTREAM | CG31612-RA | "-"
insitu highlight CG1374 in-situ | tsh | + | 12766 | 21184 | UPSTREAM | CG1374-RA | "-"

*********************** Rank 470 [Score  8.265400] GBROWSE*******************

 CG30080 in-situ | CG30080 | - | -5181 | -8156 | UPSTREAM | CG30080-RA | "-"
 CG30083 in-situ | CG30083 | - | 5719 | 4748 | DOWNSTREAM | CG30083-RA | "-"

*********************** Rank 471 [Score  8.264700] GBROWSE*******************

 CG15711 in-situ | CG15711 | - | -4868 | -5531 | UPSTREAM | CG15711-RA | "-"
 CG30322 in-situ | CG30322 | + | 29752 | 30335 | UPSTREAM | CG30322-RA | "-"

*********************** Rank 472 [Score  8.261700] GBROWSE*******************

insitu highlight CG2328 in-situ | eve | + | -8226 | -5038 | DOWNSTREAM | CG2328-RA | "-"
 CG2331 in-situ | TER94 | + | 3344 | 7750 | UPSTREAM | CG2331-RA | "-" | CG2331-RB | "-"

note: overlaps known module eve_stripe1 by 382 bases (module coords: 5046568-5047368)

*********************** Rank 473 [Score  8.261400] GBROWSE*******************

 CG7554 in-situ | comm2 | - | -14572 | -16830 | UPSTREAM | CG7554-RA | "-"
insitu highlight CG17943 in-situ | comm | - | 14002 | 7903 | DOWNSTREAM | CG17943-RA | "-"


*********************** Rank 474 [Score  8.248800] GBROWSE*******************

 CG32193 in-situ | CG32193 | + | -73611 | -69591 | DOWNSTREAM | CG32193-RA | "-"
 CG32192 in-situ | CG32192 | + | 5046 | 5781 | UPSTREAM | CG32192-RA | "-" | CG32192-RB | "-"

*********************** Rank 475 [Score  8.244100] GBROWSE*******************

 CG4395 in-situ | CG4395 | - | -4351 | -7586 | UPSTREAM | CG4395-RA | "-"
 CG32642 in-situ | CG32642 | + | 392 | 1620 | UPSTREAM | CG32642-RC | "-"

*********************** Rank 476 [Score  8.241900] GBROWSE*******************

 CG30076 in-situ | CG30076 | - | -9965 | -11287 | UPSTREAM | CG30076-RA | "-"
 CG10119 in-situ | LamC | - | 20078 | 15080 | DOWNSTREAM | CG10119-RA | "-"

*********************** Rank 477 [Score  8.241800] GBROWSE*******************

 CG32450 in-situ | CG32450 | + | -71944 | -71045 | DOWNSTREAM | CG32450-RA | "-"
insitu highlight CG5723 in-situ | Ten-m | - | 6165 | -108690 | INTRAGENIC | intron:CG5723-RB:1 | CG5723-RB | "-"

*********************** Rank 478 [Score  8.240300] GBROWSE*******************

 CG16997 in-situ | CG16997 | + | -12305 | -11484 | DOWNSTREAM | CG16997-RA | "-"
 CG17211 in-situ | CG17211 | + | 1669 | 6200 | UPSTREAM | CG17211-RA | "-"

*********************** Rank 479 [Score  8.235700] GBROWSE*******************

 CG16785 in-situ | fz3 | + | -1085 | 12198 | INTRAGENIC | intron:CG16785-RA:1 | CG16785-RA | "-"
 CG12311 in-situ | EG:34F3.7 | + | 13678 | 16931 | UPSTREAM | CG12311-RA | "-"


*********************** Rank 480 [Score  8.232700] GBROWSE*******************

 CG32376 in-situ | CG32376 | - | -3264 | -4139 | UPSTREAM | CG32376-RA | "-"
 CG14837 in-situ | CG14837 | - | 14492 | 10147 | DOWNSTREAM | CG14837-RB | "-" | CG14837-RA | "-"

*********************** Rank 481 [Score  8.231200] GBROWSE*******************

insitu CG11267 in-situ | CG11267 | + | -496 | 354 | INTRAGENIC | intron:CG11267-RA:2 | CG11267-RA | "-"
 CG11258 in-situ | mRpL20 | - | 1280 | 592 | DOWNSTREAM | CG11258-RA | "-"


*********************** Rank 482 [Score  8.229200] GBROWSE*******************

 CG4676 in-situ | CG4676 | + | -1324 | -265 | DOWNSTREAM | CG4676-RA | "-"
insitu CG4679 in-situ | CG4679 | - | 2281 | 69 | DOWNSTREAM | CG4679-RA | "-"

*********************** Rank 483 [Score  8.228800] GBROWSE*******************

 CG14597 in-situ | CG14597 | - | -25918 | -26559 | UPSTREAM | CG14597-RA | "-"
 CG31146 in-situ | CG31146 | + | 28590 | 64659 | UPSTREAM | CG31146-RD | "-"

*********************** Rank 484 [Score  8.227000] GBROWSE*******************

 CG4786 in-situ | CG4786 | - | -72148 | -84594 | UPSTREAM | CG4786-RA | "-"
insitu highlight CG4761 in-situ | knrl | - | 7974 | -15421 | INTRAGENIC | intron:CG4761-RA:2 | CG4761-RA | "-"

*********************** Rank 485 [Score  8.226800] GBROWSE*******************

 CG5411 in-situ | CG5411 | + | -4756 | 17696 | INTRAGENIC | intron:CG5411-RA:1 | intron:CG5411-RE:2 | CG5411-RA | "-" | CG5411-RE | "-" | CG5411-RB | "-" | CG5411-RD | "-" | CG5411-RC | "-"
 CG5428 in-situ | CG5428 | + | 19292 | 20620 | UPSTREAM | CG5428-RA | "-"

*********************** Rank 486 [Score  8.226000] GBROWSE*******************

insitu CG1560 in-situ | mys | + | -407 | 8183 | INTRAGENIC | intron:CG1560-RA:1 | CG1560-RA | "-"
 CG2253 in-situ | CG2253 | - | 13421 | 9231 | DOWNSTREAM | CG2253-RA | "-"

*********************** Rank 487 [Score  8.219400] GBROWSE*******************

 CG15025 in-situ | CG15025 | - | -29246 | -30791 | UPSTREAM | CG15025-RA | "-"
 CG7344 in-situ | CG7344 | + | 48851 | 66204 | UPSTREAM | CG7344-RA | "-"

*********************** Rank 488 [Score  8.218900] GBROWSE*******************

insitu CG31666 in-situ | CG31666 | + | -17273 | 28094 | INTRAGENIC | intron:CG31666-RD:1 | intron:CG31666-RA:2 | intron:CG31666-RB:2 | intron:CG31666-RC:2 | CG31666-RD | "-" | CG31666-RA | "-" | CG31666-RB | "-" | CG31666-RC | "-"
 CG31934 in-situ | CG31934 | - | 781 | 97 | DOWNSTREAM | CG31934-RA | "-"

*********************** Rank 489 [Score  8.215600] GBROWSE*******************

 CG32119 in-situ | CG32119 | - | -5451 | -7290 | UPSTREAM | CG32119-RA | "-"
 CG17673 in-situ | Acp70A | + | 47503 | 47790 | UPSTREAM | CG17673-RA | "-"

*********************** Rank 490 [Score  8.214200] GBROWSE*******************

insitu CG11347 in-situ | CG11347 | + | -14562 | -1441 | DOWNSTREAM | CG11347-RD | "-" | CG11347-RB | "-" | CG11347-RA | "-" | CG11347-RC | "-"
 CG15020 in-situ | CG15020 | + | 2082 | 4657 | UPSTREAM | CG15020-RA | "-"

*********************** Rank 491 [Score  8.212200] GBROWSE*******************

 CG31262 in-situ | CG31262 | - | -14381 | -16474 | UPSTREAM | CG31262-RA | "-"
 CG4135 in-situ | beat-IIb | - | 14063 | -1053 | INTRAGENIC | intron:CG4135-RA:4 | CG4135-RA | "-"

*********************** Rank 492 [Score  8.208100] GBROWSE*******************

insitu CG9285 in-situ | Dip-B | + | -1319 | 2070 | INTRAGENIC | intron:CG9285-RA:2 | intron:CG9285-RC:2 | intron:CG9285-RB:1 | CG9285-RA | "-" | CG9285-RC | "-" | CG9285-RB | "-"
 CG14363 in-situ | CG14363 | - | 7370 | 4393 | DOWNSTREAM | CG14363-RA | "-"

*********************** Rank 493 [Score  8.207500] GBROWSE*******************

 CG32170 in-situ | CG32170 | + | -14311 | -12689 | DOWNSTREAM | CG32170-RA | "-"
 CG11915 in-situ | CG11915 | + | 7697 | 15394 | UPSTREAM | CG11915-RA | "-"

*********************** Rank 494 [Score  8.206400] GBROWSE*******************

 CG14925 in-situ | CG14925 | - | -9976 | -10993 | UPSTREAM | CG14925-RA | "-"
 CG14926 in-situ | CG14926 | - | 47684 | 46734 | DOWNSTREAM | CG14926-RA | "-"

*********************** Rank 495 [Score  8.204200] GBROWSE*******************

 CG17738 in-situ | CG17738 | + | -22039 | -21707 | DOWNSTREAM | CG17738-RA | "-"
 CG4066 in-situ | CG4066 | + | 18394 | 20160 | UPSTREAM | CG4066-RA | "-"

*********************** Rank 496 [Score  8.203500] GBROWSE*******************

insitu CG32145 in-situ | ome | + | -18116 | 22273 | INTRAGENIC | intron:CG32145-RA:1 | CG32145-RA | "-"
 CG13473 in-situ | CG13473 | + | 3970 | 4389 | UPSTREAM | CG13473-RA | "-"

*********************** Rank 497 [Score  8.203400] GBROWSE*******************

 CG8989 in-situ | His3.3B | - | -182 | -3628 | UPSTREAM | CG8989-RA | "-" | CG8989-RC | "-" | CG8989-RB | "-"
insitu CG9022 in-situ | Ost48 | - | 1645 | 56 | DOWNSTREAM | CG9022-RA | "-"

*********************** Rank 498 [Score  8.203100] GBROWSE*******************

 CG31085 in-situ | CG31085 | + | -24308 | -11224 | DOWNSTREAM | CG31085-RB | "-" | CG31085-RA | "-"
 CG14239 in-situ | CG14239 | - | 29575 | 28696 | DOWNSTREAM | CG14239-RA | "-"

*********************** Rank 499 [Score  8.201000] GBROWSE*******************

 CG15544 in-situ | CG15544 | + | -17662 | -3478 | DOWNSTREAM | CG15544-RA | "-"
insitu highlight CG1378 in-situ | tll | + | 6856 | 8861 | UPSTREAM | CG1378-RA | "-"

*********************** Rank 500 [Score  8.200000] GBROWSE*******************

 CG5630 in-situ | CG5630 | + | -660 | 12661 | INTRAGENIC | intron:CG5630-RA:1 | CG5630-RA | "-"
 CG31191 in-situ | CG31191 | - | 30569 | -5748 | INTRAGENIC | intron:CG31191-RA:2 | CG31191-RA | "-"

*********************** Rank 501 [Score  8.199000] GBROWSE*******************

 CG10579 in-situ | Eip63E | + | -41686 | 51180 | INTRAGENIC | intron:CG10579-RD:2 | intron:CG10579-RE:3 | intron:CG10579-RA:4 | intron:CG10579-RB:3 | intron:CG10579-RC:3 | CG10579-RD | "-" | CG10579-RE | "-" | CG10579-RA | "-" | CG10579-RB | "-" | CG10579-RC | "-"
 CG10359 in-situ | CG10359 | + | 54823 | 57874 | UPSTREAM | CG10359-RA | "-"

*********************** Rank 502 [Score  8.192400] GBROWSE*******************

 CG13111 in-situ | CG13111 | - | -11024 | -11590 | UPSTREAM | CG13111-RA | "-"
 CG4450 in-situ | CG4450 | - | 469 | -7146 | INTRAGENIC | intron:CG4450-RA:2 | CG4450-RA | "-"

*********************** Rank 503 [Score  8.189800] GBROWSE*******************

 CG9476 in-situ | alphaTub85E | - | -3264 | -5296 | UPSTREAM | CG9476-RA | "-"
insitu CG12950 in-situ | CG12950 | - | 3417 | -1674 | INTRAGENIC | intron:CG12950-RA:11 | CG12950-RA | "-"

*********************** Rank 504 [Score  8.189000] GBROWSE*******************

 CG2095 in-situ | CG2095 | - | -6904 | -10953 | UPSTREAM | CG2095-RA | "-"
 CG10978 in-situ | CG10978 | + | 13428 | 15427 | UPSTREAM | CG10978-RB | "-" | CG10978-RA | "-" | CG10978-RC | "-"

*********************** Rank 505 [Score  8.188800] GBROWSE*******************

 CG17047 in-situ | CG17047 | + | -15031 | -13214 | DOWNSTREAM | CG17047-RA | "-"
 CG17048 in-situ | CG17048 | - | 39647 | 39123 | DOWNSTREAM | CG17048-RA | "-"

*********************** Rank 506 [Score  8.182600] GBROWSE*******************

 CG9086 in-situ | CG9086 | - | -7492 | -19194 | UPSTREAM | CG9086-RA | "-"
 CG9059 in-situ | CG9059 | - | 10925 | -3445 | INTRAGENIC | intron:CG9059-RA:4 | intron:CG9059-RB:3 | CG9059-RA | "-" | CG9059-RB | "-"


*********************** Rank 507 [Score  8.180400] GBROWSE*******************

 CG14910 in-situ | CG14910 | + | -8705 | -8202 | DOWNSTREAM | CG14910-RA | "-"
 CG14911 in-situ | CG14911 | + | 13682 | 14685 | UPSTREAM | CG14911-RA | "-"

*********************** Rank 508 [Score  8.169800] GBROWSE*******************

 CG6857 in-situ | CCKLR-17D1 | - | -8719 | -26627 | UPSTREAM | CG6857-RA | "-"
 CG6873 in-situ | CG6873 | - | 4433 | 3987 | DOWNSTREAM | CG6873-RA | "-"

*********************** Rank 509 [Score  8.168200] GBROWSE*******************

 CG17004 in-situ | DD2R | - | -3197 | -12309 | UPSTREAM | CG17004-RB | "-"
insitu CG9569 in-situ | CG9569 | - | 11876 | 9408 | DOWNSTREAM | CG9569-RA | "-"

*********************** Rank 510 [Score  8.160100] GBROWSE*******************

insitu highlight CG6716 in-situ | prd | - | -2723 | -6182 | UPSTREAM | CG6716-RB | "-" | CG6716-RA | "-"
 CG5325 in-situ | CG5325 | + | 4592 | 6073 | UPSTREAM | CG5325-RA | "-" | CG5325-RB | "-"

note: overlaps known module prd_Pstripe by 314 bases (module coords: 12077663-12078363)
note: overlaps known module prd_rescue by 500 bases (module coords: 12066868-12085327)

*********************** Rank 511 [Score  8.156900] GBROWSE*******************

 CG12621 in-situ | beat-IIIa | + | -4655 | 6898 | INTRAGENIC | intron:CG12621-RA:2 | CG12621-RA | "-"
 CG31747 in-situ | Gr36a | + | 13401 | 14704 | UPSTREAM | CG31747-RA | "-"

*********************** Rank 512 [Score  8.156800] GBROWSE*******************

 CG14707 in-situ | CG14707 | - | -7363 | -7548 | UPSTREAM | CG14707-RA | "-"
 CG14708 in-situ | CG14708 | + | 7579 | 8196 | UPSTREAM | CG14708-RA | "-"

*********************** Rank 513 [Score  8.153400] GBROWSE*******************

 CG11550 in-situ | CG11550 | - | -33282 | -34935 | UPSTREAM | CG11550-RA | "-"
insitu CG1856 in-situ | ttk | + | 722 | 22230 | UPSTREAM | CG1856-RE | "-" | CG1856-RF | "-" | CG1856-RB | "-" | CG1856-RC | "-" | CG1856-RA | "-" | CG1856-RD | "-"

*********************** Rank 514 [Score  8.151500] GBROWSE*******************

insitu CG13321 in-situ | CG13321 | + | -2830 | -1211 | DOWNSTREAM | CG13321-RA | "-"
 CG3886 in-situ | Psc | - | 24603 | 9924 | DOWNSTREAM | CG3886-RA | "-"


*********************** Rank 515 [Score  8.151400] GBROWSE*******************

 CG12110 in-situ | Pld | + | -114911 | -101669 | DOWNSTREAM | CG12110-RB | "-" | CG12110-RC | "-" | CG12110-RA | "-" | CG12110-RE | "-" | CG12110-RD | "-"
 CG9397 in-situ | 1.28 | + | 68270 | 69220 | UPSTREAM | CG9397-RA | "-"

*********************** Rank 516 [Score  8.150100] GBROWSE*******************

 CG14579 in-situ | CG14579 | + | -8539 | -7550 | DOWNSTREAM | CG14579-RA | "-"
 CG1724 in-situ | CG1724 | - | 1504 | 657 | DOWNSTREAM | CG1724-RA | "-"

*********************** Rank 517 [Score  8.149400] GBROWSE*******************

 CG3358 in-situ | CG3358 | + | -6027 | -4717 | DOWNSTREAM | CG3358-RA | "-" | CG3358-RB | "-"
 CG15241 in-situ | CG15241 | - | 16000 | 13772 | DOWNSTREAM | CG15241-RA | "-"

*********************** Rank 518 [Score  8.144500] GBROWSE*******************

 CG15275 in-situ | BG:DS01219.3 | - | -26170 | -26893 | UPSTREAM | CG15275-RA | "-"
 CG4482 in-situ | BG:DS01219.1 | - | 31275 | 12987 | DOWNSTREAM | CG4482-RA | "-" | CG4482-RB | "-"

*********************** Rank 519 [Score  8.142000] GBROWSE*******************

 CG7855 in-situ | timeout | + | -35908 | 39317 | INTRAGENIC | intron:CG7855-RA:11 | CG7855-RA | "-"
 CG17319 in-situ | CG17319 | - | 3990 | 1552 | DOWNSTREAM | CG17319-RA | "-"

*********************** Rank 520 [Score  8.140700] GBROWSE*******************

 CG5490 in-situ | Tl | + | -2170 | 41190 | INTRAGENIC | intron:CG5490-RB:1 | intron:CG5490-RA:1 | CG5490-RB | "-" | CG5490-RA | "-"
 CG31072 in-situ | CG31072 | - | 61908 | 41249 | DOWNSTREAM | CG31072-RB | "-" | CG31072-RC | "-" | CG31072-RA | "-"


*********************** Rank 521 [Score  8.139300] GBROWSE*******************

 CG7211 in-situ | CG7211 | + | -3226 | -2695 | DOWNSTREAM | CG7211-RA | "-"
 CG7203 in-situ | CG7203 | - | 2591 | 1599 | DOWNSTREAM | CG7203-RA | "-"

*********************** Rank 522 [Score  8.138400] GBROWSE*******************

 CG5488 in-situ | B-H2 | + | -69760 | -60179 | DOWNSTREAM | CG5488-RA | "-"
insitu CG5529 in-situ | B-H1 | + | 13160 | 18938 | UPSTREAM | CG5529-RA | "-"


*********************** Rank 523 [Score  8.131400] GBROWSE*******************

 CG13315 in-situ | CG13315 | + | -32264 | -31644 | DOWNSTREAM | CG13315-RA | "-"
 CG4760 in-situ | bol | - | 1212 | -27514 | INTRAGENIC | intron:CG4760-RB:1 | intron:CG4760-RC:1 | CG4760-RB | "-" | CG4760-RC | "-" | CG4760-RD | "-" | CG4760-RA | "-"

*********************** Rank 524 [Score  8.128200] GBROWSE*******************

 CG11641 in-situ | CG11641 | + | -20433 | -11505 | DOWNSTREAM | CG11641-RA | "-"
 CG2121 in-situ | CG2121 | - | 3422 | -11410 | INTRAGENIC | intron:CG2121-RA:1 | CG2121-RA | "-"

*********************** Rank 525 [Score  8.121600] GBROWSE*******************

 CG31828 in-situ | CG31828 | - | -27283 | -33632 | UPSTREAM | CG31828-RA | "-"
 CG18482 in-situ | BG:DS03023.4 | - | 4412 | -15480 | INTRAGENIC | intron:CG18482-RA:4 | CG18482-RA | "-"

*********************** Rank 526 [Score  8.117100] GBROWSE*******************

 CG14678 in-situ | CG14678 | - | -45073 | -47874 | UPSTREAM | CG14678-RA | "-"
 CG11373 in-situ | CG11373 | - | 28454 | 27792 | DOWNSTREAM | CG11373-RA | "-"


*********************** Rank 527 [Score  8.116100] GBROWSE*******************

 CG2245 in-situ | CG2245 | - | -6233 | -7317 | UPSTREAM | CG2245-RA | "-"
 CG2196 in-situ | CG2196 | - | 929 | -1953 | INTRAGENIC | intron:CG2196-RA:2 | CG2196-RA | "-"


*********************** Rank 528 [Score  8.106400] GBROWSE*******************

 CG31662 in-situ | Gr22a | - | -4068 | -5317 | UPSTREAM | CG31662-RA | "-"
 CG31933 in-situ | CG31933 | - | 24346 | 22285 | DOWNSTREAM | CG31933-RA | "-"

*********************** Rank 529 [Score  8.106100] GBROWSE*******************

 CG10272 in-situ | CG10272 | + | -15052 | 24086 | INTRAGENIC | intron:CG10272-RA:2 | intron:CG10272-RB:1 | intron:CG10272-RC:2 | intron:CG10272-RD:2 | CG10272-RA | "-" | CG10272-RB | "-" | CG10272-RC | "-" | CG10272-RD | "-"
 CG9727 in-situ | CG9727 | - | 34516 | 29922 | DOWNSTREAM | CG9727-RA | "-"

*********************** Rank 530 [Score  8.105300] GBROWSE*******************

 CG18812 in-situ | CG18812 | - | -130 | -17303 | UPSTREAM | CG18812-RC | "-" | CG18812-RB | "-" | CG18812-RA | "-"
insitu CG30497 in-situ | CG30497 | - | 46865 | 2001 | DOWNSTREAM | CG30497-RA | "-" | CG30497-RB | "-" | CG30497-RC | "-"

*********************** Rank 531 [Score  8.105200] GBROWSE*******************

 CG30111 in-situ | CG30111 | + | -15680 | -12301 | DOWNSTREAM | CG30111-RA | "-"
 CG11430 in-situ | olf186-F | + | 13420 | 30545 | UPSTREAM | CG11430-RB | "-" | CG11430-RC | "-" | CG11430-RA | "-"

*********************** Rank 532 [Score  8.104300] GBROWSE*******************

insitu CG10698 in-situ | GRHRII | - | -32767 | -45191 | UPSTREAM | CG10698-RA | "-"
 CG4357 in-situ | CG4357 | - | 7112 | -15657 | INTRAGENIC | intron:CG4357-RA:1 | CG4357-RA | "-"

*********************** Rank 533 [Score  8.103000] GBROWSE*******************

 CG11423 in-situ | CG11423 | + | -28608 | -26483 | DOWNSTREAM | CG11423-RA | "-"
insitu CG10683 in-situ | rhi | - | 9637 | 4614 | DOWNSTREAM | CG10683-RA | "-"

*********************** Rank 534 [Score  8.102400] GBROWSE*******************

 CG7112 in-situ | CG7112 | + | -8049 | -3866 | DOWNSTREAM | CG7112-RA | "-"
 CG17352 in-situ | CG17352 | - | 706 | -3235 | INTRAGENIC | intron:CG17352-RA:2 | intron:CG17352-RB:2 | intron:CG17352-RC:2 | CG17352-RA | "-" | CG17352-RB | "-" | CG17352-RC | "-"

*********************** Rank 535 [Score  8.102000] GBROWSE*******************

insitu highlight CG31246 in-situ | CG31246 | + | -31669 | -5013 | DOWNSTREAM | CG31246-RA | "-"
 CG31247 in-situ | tinc | + | 9161 | 27558 | UPSTREAM | CG31247-RB | "-" | CG31247-RA | "-" | CG31247-RC | "-" | CG31247-RD | "-"

*********************** Rank 536 [Score  8.095100] GBROWSE*******************

 CG4815 in-situ | CG4815 | - | -28199 | -28996 | UPSTREAM | CG4815-RA | "-"
 CG12872 in-situ | CG12872 | + | 11926 | 12800 | UPSTREAM | CG12872-RA | "-"

*********************** Rank 537 [Score  8.092500] GBROWSE*******************

insitu CG18507 in-situ | BG:DS01368.1 | + | -24270 | -18944 | DOWNSTREAM | CG18507-RA | "-" | CG18507-RB | "-"
 CG7311 in-situ | BG:DS08249.2 | + | 18417 | 20794 | UPSTREAM | CG7311-RA | "-" | CG7311-RC | "-"

*********************** Rank 538 [Score  8.092500] GBROWSE*******************

 CG32810 in-situ | EG:196F3.2 | + | -29104 | -24543 | DOWNSTREAM | CG32810-RB | "-"
 CG14796 in-situ | EG:56G7.1 | - | 15344 | 9563 | DOWNSTREAM | CG14796-RA | "-"

*********************** Rank 539 [Score  8.090900] GBROWSE*******************

 CG16741 in-situ | CG16741 | + | -6352 | -5792 | DOWNSTREAM | CG16741-RA | "-"
 CG11192 in-situ | CG11192 | - | 1635 | 826 | DOWNSTREAM | CG11192-RA | "-"

*********************** Rank 540 [Score  8.082400] GBROWSE*******************

 CG7727 in-situ | Appl | + | -3993 | 42526 | INTRAGENIC | intron:CG7727-RA:1 | CG7727-RA | "-"
insitu highlight CG6172 in-situ | vnd | + | 55429 | 62209 | UPSTREAM | CG6172-RA | "-"


*********************** Rank 541 [Score  8.078700] GBROWSE*******************

 CG1347 in-situ | CG1347 | + | -15468 | -6380 | DOWNSTREAM | CG1347-RA | "-"
insitu CG2108 in-situ | CG2108 | - | 245 | -5127 | INTRAGENIC | intron:CG2108-RA:1 | CG2108-RA | "-"

*********************** Rank 542 [Score  8.074500] GBROWSE*******************

 CG30423 in-situ | CG30423 | - | -425 | -1310 | UPSTREAM | CG30423-RB | "-" | CG30423-RA | "-"
 CG9047 in-situ | CG9047 | + | 5289 | 10170 | UPSTREAM | CG9047-RB | "-" | CG9047-RA | "-" | CG9047-RC | "-"

*********************** Rank 543 [Score  8.070500] GBROWSE*******************

 CG18341 in-situ | CG18341 | + | -4869 | -3818 | DOWNSTREAM | CG18341-RA | "-"
 CG15766 in-situ | CG15766 | - | 1891 | 1241 | DOWNSTREAM | CG15766-RA | "-"

*********************** Rank 544 [Score  8.070400] GBROWSE*******************

 CG15398 in-situ | CG15398 | - | -33113 | -34055 | UPSTREAM | CG15398-RA | "-"
insitu CG31689 in-situ | CG31689 | + | 675 | 10402 | UPSTREAM | CG31689-RC | "-" | CG31689-RD | "-" | CG31689-RA | "-" | CG31689-RB | "-"

*********************** Rank 545 [Score  8.067300] GBROWSE*******************

insitu CG15444 in-situ | ine | - | -56507 | -65545 | UPSTREAM | CG15444-RB | "-" | CG15444-RC | "-" | CG15444-RD | "-" | CG15444-RA | "-"
insitu CG33196 in-situ | CG33196 | - | 40811 | -54066 | INTRAGENIC | intron:CG33196-RB:15 | CG33196-RB | "-"

*********************** Rank 546 [Score  8.066300] GBROWSE*******************

 CG14438 in-situ | CG14438 | + | -752 | 13223 | INTRAGENIC | intron:CG14438-RA:1 | CG14438-RA | "-" | CG14438-RB | "-"
 CG14437 in-situ | COQ7 | + | 14162 | 15965 | UPSTREAM | CG14437-RA | "-"

*********************** Rank 547 [Score  8.065000] GBROWSE*******************

 CG4866 in-situ | CG4866 | + | -10809 | -10055 | DOWNSTREAM | CG4866-RA | "-"
 CG33130 in-situ | CG33130 | - | 6574 | -9395 | INTRAGENIC | intron:CG33130-RA:8 | CG33130-RA | "-"

*********************** Rank 548 [Score  8.064400] GBROWSE*******************

insitu highlight CG6464 in-situ | salm | - | -30976 | -42268 | UPSTREAM | CG6464-RA | "-"
insitu highlight CG4922 in-situ | sala | + | 9610 | 10376 | UPSTREAM | CG4922-RA | "-"


*********************** Rank 549 [Score  8.061700] GBROWSE*******************

insitu CG10953 in-situ | CG10953 | - | -18124 | -19164 | UPSTREAM | CG10953-RA | "-"
 CG10950 in-situ | CG10950 | + | 10578 | 12539 | UPSTREAM | CG10950-RA | "-"

*********************** Rank 550 [Score  8.060400] GBROWSE*******************

insitu highlight CG4702 in-situ | CG4702 | - | -23312 | -29932 | UPSTREAM | CG4702-RA | "-"
 CG10095 in-situ | CG10095 | - | 7086 | -12676 | INTRAGENIC | intron:CG10095-RA:2 | CG10095-RA | "-"

*********************** Rank 551 [Score  8.052300] GBROWSE*******************

 CG8589 in-situ | CG8589 | - | -571 | -2824 | UPSTREAM | CG8589-RA | "-"
 CG13942 in-situ | CG13942 | + | 22902 | 26894 | UPSTREAM | CG13942-RA | "-"

*********************** Rank 552 [Score  8.051900] GBROWSE*******************

 CG7343 in-situ | CG7343 | - | -13238 | -16180 | UPSTREAM | CG7343-RA | "-"
 CG3610 in-situ | CG3610 | + | 21683 | 23377 | UPSTREAM | CG3610-RA | "-"

*********************** Rank 553 [Score  8.051300] GBROWSE*******************

insitu CG10710 in-situ | CG10710 | - | -41781 | -45109 | UPSTREAM | CG10710-RA | "-"
 CG12478 in-situ | bru-3 | - | 105367 | -23464 | INTRAGENIC | intron:CG12478-RA:6 | intron:CG12478-RB:4 | CG12478-RA | "-" | CG12478-RB | "-"

*********************** Rank 554 [Score  8.050500] GBROWSE*******************

 CG5974 in-situ | pll | - | -2033 | -4082 | UPSTREAM | CG5974-RA | "-"
 CG5965 in-situ | woc | - | 8823 | 1380 | DOWNSTREAM | CG5965-RA | "-"

*********************** Rank 555 [Score  8.049700] GBROWSE*******************

 CG9045 in-situ | Myb | - | -733 | -3679 | UPSTREAM | CG9045-RA | "-"
 CG10545 in-situ | Gbeta13F | + | 1598 | 4350 | UPSTREAM | CG10545-RB | "-" | CG10545-RA | "-"

*********************** Rank 556 [Score  8.041900] GBROWSE*******************

 CG32320 in-situ | CG32320 | + | -7967 | -5543 | DOWNSTREAM | CG32320-RA | "-"
 CG9168 in-situ | CG9168 | + | 4655 | 6507 | UPSTREAM | CG9168-RA | "-"

*********************** Rank 557 [Score  8.038500] GBROWSE*******************

 CG32062 in-situ | CG32062 | + | -23116 | 54300 | INTRAGENIC | intron:CG32062-RB:2 | intron:CG32062-RD:2 | CG32062-RB | "-" | CG32062-RD | "-"
 CG6527 in-situ | CG6527 | - | 19928 | 19044 | DOWNSTREAM | CG6527-RA | "-"

*********************** Rank 558 [Score  8.038200] GBROWSE*******************

 CG8072 in-situ | CG8072 | + | -11507 | -10715 | DOWNSTREAM | CG8072-RA | "-"
 CG6628 in-situ | CG6628 | - | 3554 | 2597 | DOWNSTREAM | CG6628-RA | "-"


*********************** Rank 559 [Score  8.037600] GBROWSE*******************

 CG10633 in-situ | CG10633 | - | -4271 | -8306 | UPSTREAM | CG10633-RA | "-"
 CG4814 in-situ | CG4814 | + | 45915 | 47185 | UPSTREAM | CG4814-RA | "-"

*********************** Rank 560 [Score  8.033800] GBROWSE*******************

 CG31257 in-situ | CG31257 | + | -4398 | -2351 | DOWNSTREAM | CG31257-RA | "-"
 CG31418 in-situ | CG31418 | + | 25332 | 26052 | UPSTREAM | CG31418-RA | "-"


*********************** Rank 561 [Score  8.032800] GBROWSE*******************

 CG4069 in-situ | CG4069 | - | -22693 | -24579 | UPSTREAM | CG4069-RA | "-"
 CG10632 in-situ | CG10632 | - | 12519 | -22494 | INTRAGENIC | intron:CG10632-RA:2 | CG10632-RA | "-" | CG10632-RB | "-"

*********************** Rank 562 [Score  8.025900] GBROWSE*******************

 CG9445 in-situ | CG9445 | - | -9821 | -10674 | UPSTREAM | CG9445-RA | "-"
 CG9446 in-situ | coro | - | 24021 | 13253 | DOWNSTREAM | CG9446-RA | "-" | CG9446-RB | "-"


*********************** Rank 563 [Score  8.024400] GBROWSE*******************

 CG4797 in-situ | CG4797 | - | -4006 | -10151 | UPSTREAM | CG4797-RB | "-" | CG4797-RA | "-"
 CG4763 in-situ | CG4763 | - | 2536 | 1025 | DOWNSTREAM | CG4763-RA | "-"

*********************** Rank 564 [Score  8.020800] GBROWSE*******************

 CG16898 in-situ | CG16898 | - | -35101 | -36469 | UPSTREAM | CG16898-RA | "-"
 CG8896 in-situ | 18w | + | 71423 | 76844 | UPSTREAM | CG8896-RA | "-"

*********************** Rank 565 [Score  8.016700] GBROWSE*******************

insitu CG6844 in-situ | nAcRalpha-96Ab | + | -5779 | -68 | DOWNSTREAM | CG6844-RA | "-" | CG6844-RB | "-"
 CG6798 in-situ | nAcRbeta-96A | - | 23880 | 17993 | DOWNSTREAM | CG6798-RA | "-" | CG6798-RB | "-"

*********************** Rank 566 [Score  8.015000] GBROWSE*******************

insitu highlight CG3851 in-situ | odd | - | -2683 | -5209 | UPSTREAM | CG3851-RA | "-"
 CG2788 in-situ | Dot | + | 9861 | 11740 | UPSTREAM | CG2788-RA | "-"


*********************** Rank 567 [Score  8.014300] GBROWSE*******************

 CG17285 in-situ | Fbp1 | - | -7364 | -10834 | UPSTREAM | CG17285-RA | "-" | CG17285-RB | "-"
 CG7345 in-situ | Sox21a | - | 1654 | -1166 | INTRAGENIC | intron:CG7345-RA:1 | CG7345-RA | "-"

*********************** Rank 568 [Score  8.012100] GBROWSE*******************

insitu highlight CG10021 in-situ | bowl | + | -12675 | -252 | DOWNSTREAM | CG10021-RB | "-" | CG10021-RC | "-" | CG10021-RD | "-" | CG10021-RA | "-"
 CG31960 in-situ | CG31960 | + | 565 | 1134 | UPSTREAM | CG31960-RA | "-"

*********************** Rank 569 [Score  8.011500] GBROWSE*******************

 CG17304 in-situ | CG17304 | - | -6072 | -6605 | UPSTREAM | CG17304-RA | "-"
 CG3631 in-situ | CG3631 | + | 13531 | 16716 | UPSTREAM | CG3631-RA | "-" | CG3631-RB | "-"

*********************** Rank 570 [Score  8.009600] GBROWSE*******************

 CG9380 in-situ | CG9380 | - | -37143 | -42351 | UPSTREAM | CG9380-RA | "-" | CG9380-RB | "-"
insitu highlight CG3340 in-situ | Kr | + | 635 | 3554 | UPSTREAM | CG3340-RA | "-"

note: overlaps known module Kr_AD2 by 500 bases (module coords: 20269328-20270558)
note: overlaps known module Kr_CDHK by 400 bases (module coords: 20268583-20269949)
note: overlaps known module Kr_neuI by 311 bases (module coords: 20269638-20269948)
note: overlaps known module Kr_NS2 by 500 bases (module coords: 20269328-20271069)

*********************** Rank 571 [Score  8.009500] GBROWSE*******************

insitu highlight CG10052 in-situ | Rx | + | -5126 | 15126 | INTRAGENIC | intron:CG10052-RA:2 | CG10052-RA | "-"
insitu CG10067 in-situ | Act57B | + | 22122 | 24534 | UPSTREAM | CG10067-RA | "-"


*********************** Rank 572 [Score  8.006200] GBROWSE*******************

 CG9650 in-situ | CG9650 | + | -31648 | 15697 | INTRAGENIC | intron:CG9650-RA:2 | CG9650-RA | "-" | CG9650-RB | "-" | CG9650-RC | "-"
 CG32725 in-situ | CG32725 | - | 13438 | 12711 | DOWNSTREAM | CG32725-RA | "-"

*********************** Rank 573 [Score  8.000200] GBROWSE*******************

 CG31076 in-situ | CG31076 | - | -2035 | -2878 | UPSTREAM | CG31076-RA | "-"
insitu CG14254 in-situ | CG14254 | + | 2923 | 4318 | UPSTREAM | CG14254-RA | "-"

*********************** Rank 574 [Score  7.993700] GBROWSE*******************

insitu CG32473 in-situ | CG32473 | + | -24756 | -14612 | DOWNSTREAM | CG32473-RA | "-" | CG32473-RC | "-" | CG32473-RB | "-"
 CG8795 in-situ | CG8795 | - | 6754 | 1656 | DOWNSTREAM | CG8795-RA | "-" | CG8795-RB | "-"

*********************** Rank 575 [Score  7.990200] GBROWSE*******************

insitu CG3385 in-situ | nvy | + | -9695 | 5656 | INTRAGENIC | intron:CG3385-RA:2 | CG3385-RA | "-"
insitu CG3394 in-situ | CG3394 | + | 8666 | 12691 | UPSTREAM | CG3394-RA | "-" | CG3394-RB | "-"

*********************** Rank 576 [Score  7.989900] GBROWSE*******************

 CG32193 in-situ | CG32193 | + | -12061 | -8041 | DOWNSTREAM | CG32193-RA | "-"
 CG32192 in-situ | CG32192 | + | 66596 | 67331 | UPSTREAM | CG32192-RA | "-" | CG32192-RB | "-"

*********************** Rank 577 [Score  7.983400] GBROWSE*******************

insitu CG3001 in-situ | Hex-A | + | -6831 | -4312 | DOWNSTREAM | CG3001-RA | "-" | CG3001-RB | "-"
 CG3002 in-situ | CG3002 | + | 8318 | 11566 | UPSTREAM | CG3002-RB | "-"

*********************** Rank 578 [Score  7.982700] GBROWSE*******************

 CG8102 in-situ | CG8102 | - | -4096 | -5661 | UPSTREAM | CG8102-RB | "-" | CG8102-RA | "-"
 CG8152 in-situ | CG8152 | - | 8704 | 7506 | DOWNSTREAM | CG8152-RA | "-"

*********************** Rank 579 [Score  7.982000] GBROWSE*******************

 CG31208 in-situ | Gr92a | + | -31551 | -30511 | DOWNSTREAM | CG31208-RA | "-"
insitu CG5023 in-situ | CG5023 | + | 16825 | 22264 | UPSTREAM | CG5023-RA | "-"

*********************** Rank 580 [Score  7.978700] GBROWSE*******************

 CG9411 in-situ | CG9411 | - | -907 | -8583 | UPSTREAM | CG9411-RA | "-"
 CG12540 in-situ | CG12540 | + | 15151 | 16256 | UPSTREAM | CG12540-RA | "-"

*********************** Rank 581 [Score  7.974900] GBROWSE*******************

 CG11106 in-situ | CG11106 | + | -7442 | -6904 | DOWNSTREAM | CG11106-RA | "-"
 CG11105 in-situ | CG11105 | - | 27109 | 14418 | DOWNSTREAM | CG11105-RB | "-" | CG11105-RA | "-"

*********************** Rank 582 [Score  7.971500] GBROWSE*******************

 CG1691 in-situ | Imp | - | -7229 | -34173 | UPSTREAM | CG1691-RF | "-" | CG1691-RH | "-" | CG1691-RG | "-" | CG1691-RD | "-" | CG1691-RE | "-" | CG1691-RC | "-" | CG1691-RB | "-" | CG1691-RA | "-"
 CG15210 in-situ | CG15210 | + | 9376 | 9642 | UPSTREAM | CG15210-RA | "-"

*********************** Rank 583 [Score  7.970400] GBROWSE*******************

insitu CG32654 in-situ | CG32654 | + | -9937 | 2635 | INTRAGENIC | intron:CG32654-RC:7 | CG32654-RC | "-"
insitu CG1463 in-situ | CG1463 | - | 4309 | 2267 | DOWNSTREAM | CG1463-RA | "-"


*********************** Rank 584 [Score  7.967900] GBROWSE*******************

 CG7843 in-situ | CG7843 | - | -6312 | -11104 | UPSTREAM | CG7843-RA | "-" | CG7843-RB | "-" | CG7843-RD | "-" | CG7843-RC | "-"
 CG14589 in-situ | CG14589 | + | 45458 | 46519 | UPSTREAM | CG14589-RA | "-"

*********************** Rank 585 [Score  7.965000] GBROWSE*******************

 CG7147 in-situ | kuz | + | -2808 | 85315 | INTRAGENIC | intron:CG7147-RA:2 | intron:CG7147-RB:2 | CG7147-RA | "-" | CG7147-RB | "-"
 CG9254 in-situ | BG:DS07660.1 | - | 44237 | 42553 | DOWNSTREAM | CG9254-RA | "-"

*********************** Rank 586 [Score  7.956500] GBROWSE*******************

 CG6879 in-situ | CG6879 | + | -7254 | -2409 | DOWNSTREAM | CG6879-RA | "-"
 CG5805 in-situ | CG5805 | - | 449 | -2235 | INTRAGENIC | intron:CG5805-RA:1 | CG5805-RA | "-"

*********************** Rank 587 [Score  7.953400] GBROWSE*******************

 CG15631 in-situ | CG15631 | - | -30573 | -32622 | UPSTREAM | CG15631-RA | "-"
 CG15630 in-situ | CG15630 | - | 29011 | -29484 | INTRAGENIC | intron:CG15630-RA:1 | CG15630-RA | "-"

*********************** Rank 588 [Score  7.951800] GBROWSE*******************

 CG31513 in-situ | CG31513 | + | -13623 | -12975 | DOWNSTREAM | CG31513-RA | "-"
 CG10101 in-situ | CG10101 | + | 4123 | 6725 | UPSTREAM | CG10101-RA | "-"

*********************** Rank 589 [Score  7.950900] GBROWSE*******************

 CG4161 in-situ | BG:DS03023.2 | + | -34703 | -32815 | DOWNSTREAM | CG4161-RA | "-"
insitu CG3956 in-situ | sna | - | 10203 | 8527 | DOWNSTREAM | CG3956-RA | "-"

*********************** Rank 590 [Score  7.950300] GBROWSE*******************

 CG32467 in-situ | CG32467 | - | -3987 | -4442 | UPSTREAM | CG32467-RA | "-"
 CG32466 in-situ | rn | - | 2168 | -32117 | INTRAGENIC | intron:CG32466-RA:1 | CG32466-RA | "-"

*********************** Rank 591 [Score  7.950100] GBROWSE*******************

 CG7100 in-situ | CadN | - | -60796 | -150451 | UPSTREAM | CG7100-RA | "-" | CG7100-RC | "-" | CG7100-RD | "-" | CG7100-RE | "-" | CG7100-RF | "-" | CG7100-RG | "-" | CG7100-RH | "-" | CG7100-RB | "-"
 CG7527 in-situ | CadN2 | - | 12287 | -13712 | INTRAGENIC | intron:CG7527-RA:7 | CG7527-RA | "-"

*********************** Rank 592 [Score  7.949400] GBROWSE*******************

 CG15504 in-situ | dmrt99B | + | -20805 | -17188 | DOWNSTREAM | CG15504-RA | "-"
 CG15505 in-situ | Obp99d | + | 927 | 1340 | UPSTREAM | CG15505-RA | "-"

*********************** Rank 593 [Score  7.948500] GBROWSE*******************

insitu highlight CG4125 in-situ | rst | - | -41447 | -63072 | UPSTREAM | CG4125-RA | "-"
 CG4116 in-situ | CG4116 | - | 52283 | 51489 | DOWNSTREAM | CG4116-RA | "-"

*********************** Rank 594 [Score  7.947600] GBROWSE*******************

insitu CG4476 in-situ | CG4476 | + | -13106 | -9721 | DOWNSTREAM | CG4476-RB | "-"
 CG4483 in-situ | CG4483 | - | 11658 | 10160 | DOWNSTREAM | CG4483-RA | "-"

*********************** Rank 595 [Score  7.947500] GBROWSE*******************

 CG31258 in-situ | CG31258 | + | -23187 | -18361 | DOWNSTREAM | CG31258-RA | "-"
 CG7994 in-situ | CG7994 | + | 4175 | 6346 | UPSTREAM | CG7994-RA | "-"

*********************** Rank 596 [Score  7.947300] GBROWSE*******************

 CG17368 in-situ | CG17368 | - | -26333 | -27975 | UPSTREAM | CG17368-RA | "-"
 CG8865 in-situ | Rgl | - | 6478 | -11879 | INTRAGENIC | intron:CG8865-RB:2 | intron:CG8865-RD:2 | intron:CG8865-RC:2 | intron:CG8865-RA:1 | CG8865-RB | "-" | CG8865-RD | "-" | CG8865-RC | "-" | CG8865-RA | "-"

*********************** Rank 597 [Score  7.945700] GBROWSE*******************

 CG9868 in-situ | CG9868 | - | -3266 | -4428 | UPSTREAM | CG9868-RA | "-"
 CG30412 in-situ | CG30412 | - | 6266 | 5105 | DOWNSTREAM | CG30412-RB | "-" | CG30412-RA | "-"

*********************** Rank 598 [Score  7.943200] GBROWSE*******************

insitu highlight CG10159 in-situ | BEAF-32 | + | -20604 | -18436 | DOWNSTREAM | CG10159-RA | "-" | CG10159-RB | "-"
 CG12856 in-situ | CG12856 | + | 7205 | 7588 | UPSTREAM | CG12856-RA | "-"

*********************** Rank 599 [Score  7.939100] GBROWSE*******************

 CG9975 in-situ | CG9975 | + | -13871 | -10118 | DOWNSTREAM | CG9975-RA | "-"
insitu CG11961 in-situ | CG11961 | + | 2051 | 6693 | UPSTREAM | CG11961-RB | "-" | CG11961-RA | "-"

*********************** Rank 600 [Score  7.930500] GBROWSE*******************

 CG31537 in-situ | CG31537 | - | -5693 | -7657 | UPSTREAM | CG31537-RA | "-"
 CG2534 in-situ | cno | - | 31044 | -12783 | INTRAGENIC | intron:CG2534-RA:7 | intron:CG2534-RB:7 | CG2534-RA | "-" | CG2534-RB | "-"

*********************** Rank 601 [Score  7.928400] GBROWSE*******************

 CG31715 in-situ | CG31715 | + | -970 | -64 | DOWNSTREAM | CG31715-RA | "-"
 CG5025 in-situ | Sps2 | + | 135 | 1637 | UPSTREAM | CG5025-RB | "-" | CG5025-RA | "-"

*********************** Rank 602 [Score  7.927900] GBROWSE*******************

 CG10862 in-situ | CG10862 | - | -40800 | -42106 | UPSTREAM | CG10862-RA | "-"
 CG10858 in-situ | CG10858 | - | 59630 | 52954 | DOWNSTREAM | CG10858-RA | "-"

*********************** Rank 603 [Score  7.926600] GBROWSE*******************

 CG31685 in-situ | CG31685 | - | -29985 | -30864 | UPSTREAM | CG31685-RA | "-"
 CG12617 in-situ | CG12617 | + | 94348 | 94994 | UPSTREAM | CG12617-RA | "-"


*********************** Rank 604 [Score  7.913300] GBROWSE*******************

 CG7339 in-situ | CG7339 | + | -15234 | -14151 | DOWNSTREAM | CG7339-RA | "-"
 CG32085 in-situ | CG32085 | - | 16338 | -13518 | INTRAGENIC | intron:CG32085-RA:4 | CG32085-RA | "-"

*********************** Rank 605 [Score  7.912300] GBROWSE*******************

 CG6014 in-situ | BcDNA:GH11973 | + | -21650 | 19904 | INTRAGENIC | intron:CG6014-RA:1 | CG6014-RA | "-"
 CG11308 in-situ | CG11308 | - | 2522 | 1337 | DOWNSTREAM | CG11308-RA | "-"

*********************** Rank 606 [Score  7.911400] GBROWSE*******************

 CG13106 in-situ | Or30a | + | -6105 | -4634 | DOWNSTREAM | CG13106-RA | "-"
insitu highlight CG32982 in-situ | CG32982 | + | 7832 | 45808 | UPSTREAM | CG32982-RA | "-" | CG32982-RB | "-"

*********************** Rank 607 [Score  7.910900] GBROWSE*******************

 CG32655 in-situ | CG32655 | - | -53761 | -54819 | UPSTREAM | CG32655-RA | "-"
 CG2577 in-situ | CG2577 | + | 83721 | 84961 | UPSTREAM | CG2577-RA | "-"

*********************** Rank 608 [Score  7.909600] GBROWSE*******************

 CG4983 in-situ | CG4983 | + | -23131 | -21732 | DOWNSTREAM | CG4983-RA | "-"
 CG4988 in-situ | CG4988 | + | 19277 | 20493 | UPSTREAM | CG4988-RA | "-"

*********************** Rank 609 [Score  7.909200] GBROWSE*******************

 CG8853 in-situ | CG8853 | + | -8328 | -6705 | DOWNSTREAM | CG8853-RA | "-"
insitu highlight CG10016 in-situ | drm | + | 20 | 8855 | UPSTREAM | CG10016-RB | "-" | CG10016-RA | "-"

*********************** Rank 610 [Score  7.905200] GBROWSE*******************

 CG12617 in-situ | CG12617 | + | -6302 | -5656 | DOWNSTREAM | CG12617-RA | "-"
insitu CG10076 in-situ | spir | + | 10211 | 47387 | UPSTREAM | CG10076-RA | "-" | CG10076-RB | "-" | CG10076-RD | "-" | CG10076-RC | "-"

*********************** Rank 611 [Score  7.898000] GBROWSE*******************

 CG6185 in-situ | CG6185 | - | -14830 | -17767 | UPSTREAM | CG6185-RA | "-"
 CG7590 in-situ | scylla | + | 1541 | 4753 | UPSTREAM | CG7590-RA | "-"

*********************** Rank 612 [Score  7.897000] GBROWSE*******************

 CG1671 in-situ | CG1671 | + | -5606 | -2849 | DOWNSTREAM | CG1671-RA | "-"
 CG1516 in-situ | BcDNA:GH06348 | - | 7876 | -2959 | INTRAGENIC | intron:CG1516-RI:4 | intron:CG1516-RE:3 | intron:CG1516-RD:4 | intron:CG1516-RA:3 | intron:CG1516-RJ:4 | intron:CG1516-RB:3 | intron:CG1516-RK:4 | intron:CG1516-RL:3 | intron:CG1516-RG:2 | intron:CG1516-RF:2 | intron:CG1516-RH:3 | CG1516-RI | "-" | CG1516-RE | "-" | CG1516-RD | "-" | CG1516-RA | "-" | CG1516-RJ | "-" | CG1516-RB | "-" | CG1516-RK | "-" | CG1516-RL | "-" | CG1516-RG | "-" | CG1516-RF | "-" | CG1516-RH | "-"

*********************** Rank 613 [Score  7.894800] GBROWSE*******************

 CG4582 in-situ | CG4582 | - | -4199 | -5696 | UPSTREAM | CG4582-RA | "-"
 CG12250 in-situ | CG12250 | - | 21965 | 4970 | DOWNSTREAM | CG12250-RA | "-" | CG12250-RB | "-"

*********************** Rank 614 [Score  7.887500] GBROWSE*******************

insitu highlight CG1028 in-situ | Antp | - | -6522 | -110824 | UPSTREAM | CG1028-RA | "-" | CG1028-RC | "-" | CG1028-RB | "-"
 CG1982 in-situ | Sodh-1 | + | 45258 | 47465 | UPSTREAM | CG1982-RA | "-"


*********************** Rank 615 [Score  7.883900] GBROWSE*******************

 CG6352 in-situ | OdsH | + | -15541 | 7397 | INTRAGENIC | intron:CG6352-RA:1 | CG6352-RA | "-"
 CG12986 in-situ | CG12986 | - | 13024 | 12647 | DOWNSTREAM | CG12986-RA | "-"

*********************** Rank 616 [Score  7.880400] GBROWSE*******************

 CG3425 in-situ | T3dh | - | -15192 | -17523 | UPSTREAM | CG3425-RA | "-"
 CG11298 in-situ | CG11298 | + | 1058 | 1633 | UPSTREAM | CG11298-RA | "-"


*********************** Rank 617 [Score  7.880200] GBROWSE*******************

 CG15145 in-situ | CG15145 | - | -13622 | -15550 | UPSTREAM | CG15145-RA | "-"
 CG7094 in-situ | CG7094 | - | 10748 | 9336 | DOWNSTREAM | CG7094-RA | "-"

*********************** Rank 618 [Score  7.879400] GBROWSE*******************

 CG32172 in-situ | noe | - | -13413 | -15324 | UPSTREAM | CG32172-RA | "-"
 CG3897 in-situ | blot | - | 6641 | -18847 | INTRAGENIC | intron:CG3897-RD:1 | intron:CG3897-RB:1 | CG3897-RD | "-" | CG3897-RB | "-" | CG3897-RA | "-" | CG3897-RC | "-"


*********************** Rank 619 [Score  7.877700] GBROWSE*******************

 CG31066 in-situ | CG31066 | + | -3746 | -3125 | DOWNSTREAM | CG31066-RA | "-"
 CG6127 in-situ | Ser | - | 4481 | -17417 | INTRAGENIC | intron:CG6127-RA:2 | CG6127-RA | "-"

*********************** Rank 620 [Score  7.877000] GBROWSE*******************

 CG31270 in-situ | CG31270 | - | -21178 | -22294 | UPSTREAM | CG31270-RA | "-"
 CG11648 in-situ | Abd-B | - | 18982 | -17290 | INTRAGENIC | intron:CG11648-RC:1 | intron:CG11648-RA:1 | CG11648-RC | "-" | CG11648-RA | "-" | CG11648-RD | "-" | CG11648-RB | "-"

*********************** Rank 621 [Score  7.874900] GBROWSE*******************

insitu CG10045 in-situ | GstD1 | - | -794 | -2249 | UPSTREAM | CG10045-RA | "-"
 CG4181 in-situ | GstD2 | + | 1939 | 2586 | UPSTREAM | CG4181-RA | "-"

*********************** Rank 622 [Score  7.870100] GBROWSE*******************

 CG6380 in-situ | CG6380 | - | -66710 | -67747 | UPSTREAM | CG6380-RA | "-"
 CG31804 in-situ | CG31804 | + | 4578 | 5318 | UPSTREAM | CG31804-RA | "-"

*********************** Rank 623 [Score  7.869100] GBROWSE*******************

 CG18285 in-situ | igl | + | -26236 | 8619 | INTRAGENIC | intron:CG18285-RA:2 | intron:CG18285-RB:2 | CG18285-RA | "-" | CG18285-RB | "-"
 CG8090 in-situ | CG8090 | - | 19502 | 17632 | DOWNSTREAM | CG8090-RA | "-"

*********************** Rank 624 [Score  7.868500] GBROWSE*******************

 CG12110 in-situ | Pld | + | -154861 | -141619 | DOWNSTREAM | CG12110-RB | "-" | CG12110-RC | "-" | CG12110-RA | "-" | CG12110-RE | "-" | CG12110-RD | "-"
 CG9397 in-situ | 1.28 | + | 28320 | 29270 | UPSTREAM | CG9397-RA | "-"

*********************** Rank 625 [Score  7.868300] GBROWSE*******************

insitu CG3979 in-situ | Indy | - | -2411 | -20039 | UPSTREAM | CG3979-RB | "-" | CG3979-RC | "-" | CG3979-RA | "-"
 CG6865 in-situ | CG6865 | + | 15578 | 16750 | UPSTREAM | CG6865-RA | "-"

*********************** Rank 626 [Score  7.866700] GBROWSE*******************

 CG2022 in-situ | CG2022 | - | -84729 | -86816 | UPSTREAM | CG2022-RA | "-"
insitu highlight CG2530 in-situ | corto | - | 13205 | 10140 | DOWNSTREAM | CG2530-RA | "-"

*********************** Rank 627 [Score  7.863700] GBROWSE*******************

 CG32616 in-situ | Ste12DOR | + | -10431 | -9859 | DOWNSTREAM | CG32616-RA | "-"
 CG32605 in-situ | CG32605 | - | 50755 | 36252 | DOWNSTREAM | CG32605-RA | "-"

*********************** Rank 628 [Score  7.862200] GBROWSE*******************

 CG14503 in-situ | CG14503 | + | -26538 | -26362 | DOWNSTREAM | CG14503-RA | "-"
 CG15066 in-situ | CG15066 | - | 44852 | 44316 | DOWNSTREAM | CG15066-RA | "-"

*********************** Rank 629 [Score  7.861800] GBROWSE*******************

insitu CG12177 in-situ | CG12177 | - | -595 | -3092 | UPSTREAM | CG12177-RA | "-"
insitu CG11158 in-situ | CG11158 | + | 1916 | 3633 | UPSTREAM | CG11158-RA | "-"

*********************** Rank 630 [Score  7.861300] GBROWSE*******************

 CG10043 in-situ | rtGEF | + | -4772 | 10053 | INTRAGENIC | intron:CG10043-RA:1 | intron:CG10043-RB:1 | CG10043-RA | "-" | CG10043-RB | "-"
 CG11012 in-situ | Ugt37a1 | - | 18759 | 17265 | DOWNSTREAM | CG11012-RA | "-"

*********************** Rank 631 [Score  7.860500] GBROWSE*******************

 CG4351 in-situ | CG4351 | - | -259 | -6098 | UPSTREAM | CG4351-RA | "-"
 CG4349 in-situ | CG4349 | - | 5906 | 5284 | DOWNSTREAM | CG4349-RA | "-"

*********************** Rank 632 [Score  7.860400] GBROWSE*******************

 CG6414 in-situ | CG6414 | - | -4236 | -6586 | UPSTREAM | CG6414-RA | "-"
 CG32790 in-situ | CG32790 | + | 102332 | 103630 | UPSTREAM | CG32790-RA | "-"

*********************** Rank 633 [Score  7.859600] GBROWSE*******************

insitu CG17334 in-situ | lin-28 | + | -3859 | -1158 | DOWNSTREAM | CG17334-RA | "-"
 CG10583 in-situ | Sse | - | 3699 | 1152 | DOWNSTREAM | CG10583-RA | "-"


*********************** Rank 634 [Score  7.856000] GBROWSE*******************

 CG15523 in-situ | CG15523 | + | -22238 | -10282 | DOWNSTREAM | CG15523-RA | "-"
 CG7887 in-situ | Takr99D | - | 11035 | -7621 | INTRAGENIC | intron:CG7887-RA:3 | CG7887-RA | "-"

*********************** Rank 635 [Score  7.854400] GBROWSE*******************

 CG12444 in-situ | CG12444 | - | -14744 | -16429 | UPSTREAM | CG12444-RB | "-" | CG12444-RA | "-"
 CG12443 in-situ | CG12443 | + | 5571 | 7631 | UPSTREAM | CG12443-RA | "-"

*********************** Rank 636 [Score  7.850400] GBROWSE*******************

 CG3819 in-situ | CG3819 | - | -14565 | -16150 | UPSTREAM | CG3819-RA | "-"
insitu CG32204 in-situ | CG32204 | - | 9365 | -14252 | INTRAGENIC | intron:CG32204-RA:1 | CG32204-RA | "-"

*********************** Rank 637 [Score  7.849900] GBROWSE*******************

 CG31499 in-situ | CG31499 | + | -910 | -590 | DOWNSTREAM | CG31499-RA | "-"
 CG31209 in-situ | CG31209 | + | 4454 | 23918 | UPSTREAM | CG31209-RA | "-"

*********************** Rank 638 [Score  7.848100] GBROWSE*******************

 CG15464 in-situ | CG15464 | + | -7103 | -6406 | DOWNSTREAM | CG15464-RA | "-"
insitu CG6775 in-situ | rg | + | 26995 | 56914 | UPSTREAM | CG6775-RA | "-" | CG6775-RB | "-"

*********************** Rank 639 [Score  7.848000] GBROWSE*******************

 CG4998 in-situ | CG4998 | + | -822 | 5427 | INTRAGENIC | intron:CG4998-RA:1 | CG4998-RA | "-"
insitu highlight CG13037 in-situ | mRpS34 | + | 8276 | 8955 | UPSTREAM | CG13037-RA | "-"

*********************** Rank 640 [Score  7.846300] GBROWSE*******************

 CG15883 in-situ | Obp18a | - | -11622 | -12410 | UPSTREAM | CG15883-RA | "-"
 CG7502 in-situ | CG7502 | - | 7463 | 3204 | DOWNSTREAM | CG7502-RA | "-"

*********************** Rank 641 [Score  7.842700] GBROWSE*******************

 CG32698 in-situ | CG32698 | + | -16434 | 45225 | INTRAGENIC | intron:CG32698-RA:1 | CG32698-RA | "-"
 CG2045 in-situ | Ser7 | + | 61213 | 63065 | UPSTREAM | CG2045-RA | "-"

*********************** Rank 642 [Score  7.842500] GBROWSE*******************

 CG6168 in-situ | CG6168 | - | -12249 | -13277 | UPSTREAM | CG6168-RB | "-"
 CG6163 in-situ | CG6163 | - | 30917 | 28018 | DOWNSTREAM | CG6163-RA | "-"

*********************** Rank 643 [Score  7.842400] GBROWSE*******************

 CG12290 in-situ | CG12290 | + | -7911 | -4815 | DOWNSTREAM | CG12290-RA | "-"
 CG6058 in-situ | Ald | - | 23428 | 16519 | DOWNSTREAM | CG6058-RB | "-" | CG6058-RC | "-" | CG6058-RA | "-" | CG6058-RF | "-" | CG6058-RG | "-" | CG6058-RD | "-" | CG6058-RE | "-"

*********************** Rank 644 [Score  7.842100] GBROWSE*******************

 CG13790 in-situ | CG13790 | - | -601 | -885 | UPSTREAM | CG13790-RA | "-"
 CG13791 in-situ | CG13791 | - | 9140 | 8814 | DOWNSTREAM | CG13791-RA | "-"

*********************** Rank 645 [Score  7.837000] GBROWSE*******************

 CG32082 in-situ | CG32082 | + | -569 | 26423 | INTRAGENIC | intron:CG32082-RA:1 | CG32082-RA | "-"
 CG6216 in-situ | CG6216 | - | 14167 | 7617 | DOWNSTREAM | CG6216-RA | "-"

*********************** Rank 646 [Score  7.835200] GBROWSE*******************

 CG12149 in-situ | c12.2 | + | -6124 | -1257 | DOWNSTREAM | CG12149-RA | "-"
 CG32705 in-situ | CG32705 | - | 13381 | 1042 | DOWNSTREAM | CG32705-RA | "-"

*********************** Rank 647 [Score  7.831700] GBROWSE*******************

insitu highlight CG6634 in-situ | CG6634 | + | -36544 | -30576 | DOWNSTREAM | CG6634-RA | "-"
 CG14020 in-situ | CG14020 | + | 19657 | 20637 | UPSTREAM | CG14020-RA | "-"

*********************** Rank 648 [Score  7.829400] GBROWSE*******************

 CG30166 in-situ | CG30166 | - | -1109 | -1459 | UPSTREAM | CG30166-RA | "-"
 CG15873 in-situ | CG15873 | - | 3403 | 2383 | DOWNSTREAM | CG15873-RA | "-"

*********************** Rank 649 [Score  7.829100] GBROWSE*******************

 CG11192 in-situ | CG11192 | - | -38465 | -39274 | UPSTREAM | CG11192-RA | "-"
 CG33041 in-situ | CG33041 | + | 30847 | 77476 | UPSTREAM | CG33041-RA | "-"

*********************** Rank 650 [Score  7.828200] GBROWSE*******************

 CG32345 in-situ | CG32345 | - | -10155 | -16848 | UPSTREAM | CG32345-RA | "-"
 CG12030 in-situ | CG12030 | + | 16046 | 18842 | UPSTREAM | CG12030-RA | "-"

*********************** Rank 651 [Score  7.822600] GBROWSE*******************

 CG13078 in-situ | CG13078 | + | -1079 | -411 | DOWNSTREAM | CG13078-RA | "-"
 CG13077 in-situ | CG13077 | + | 445 | 1942 | UPSTREAM | CG13077-RA | "-"


*********************** Rank 652 [Score  7.821800] GBROWSE*******************

insitu CG9894 in-situ | CG9894 | + | -2932 | 875 | INTRAGENIC | intron:CG9894-RB:2 | intron:CG9894-RA:1 | CG9894-RB | "-" | CG9894-RA | "-"
 CG32951 in-situ | CG32951 | - | 3979 | 2696 | DOWNSTREAM | CG32951-RA | "-" | CG32951-RB | "-"

*********************** Rank 653 [Score  7.821200] GBROWSE*******************

 CG12115 in-situ | CG12115 | - | -985 | -2243 | UPSTREAM | CG12115-RA | "-"
 CG12057 in-situ | CG12057 | + | 107 | 983 | UPSTREAM | CG12057-RA | "-"

*********************** Rank 654 [Score  7.817000] GBROWSE*******************

 CG10862 in-situ | CG10862 | - | -12500 | -13806 | UPSTREAM | CG10862-RA | "-"
 CG10858 in-situ | CG10858 | - | 87930 | 81254 | DOWNSTREAM | CG10858-RA | "-"

*********************** Rank 655 [Score  7.816900] GBROWSE*******************

 CG14437 in-situ | COQ7 | + | -6238 | -4435 | DOWNSTREAM | CG14437-RA | "-"
 CG3135 in-situ | CG3135 | - | 2185 | -6030 | INTRAGENIC | intron:CG3135-RA:1 | CG3135-RA | "-"

*********************** Rank 656 [Score  7.816700] GBROWSE*******************

insitu CG4364 in-situ | CG4364 | - | -4702 | -7051 | UPSTREAM | CG4364-RA | "-"
 CG13116 in-situ | CG13116 | + | 13969 | 14978 | UPSTREAM | CG13116-RA | "-"

*********************** Rank 657 [Score  7.814700] GBROWSE*******************

 CG6414 in-situ | CG6414 | - | -28636 | -30986 | UPSTREAM | CG6414-RA | "-"
 CG32790 in-situ | CG32790 | + | 77932 | 79230 | UPSTREAM | CG32790-RA | "-"

*********************** Rank 658 [Score  7.813600] GBROWSE*******************

insitu highlight CG6634 in-situ | CG6634 | + | -14644 | -8676 | DOWNSTREAM | CG6634-RA | "-"
 CG14020 in-situ | CG14020 | + | 41557 | 42537 | UPSTREAM | CG14020-RA | "-"

*********************** Rank 659 [Score  7.811500] GBROWSE*******************

 CG12199 in-situ | CG12199 | + | -55405 | 61451 | INTRAGENIC | intron:CG12199-RC:2 | intron:CG12199-RB:2 | CG12199-RC | "-" | CG12199-RB | "-" | CG12199-RA | "-"
 CG32827 in-situ | CG32827 | + | 3995 | 7994 | UPSTREAM | CG32827-RA | "-"

*********************** Rank 660 [Score  7.809900] GBROWSE*******************

 CG3090 in-situ | Sox14 | + | -7361 | -1405 | DOWNSTREAM | CG3090-RA | "-" | CG3090-RB | "-"
 CG30178 in-situ | CG30178 | + | 92 | 701 | UPSTREAM | CG30178-RA | "-"

*********************** Rank 661 [Score  7.807100] GBROWSE*******************

 CG31006 in-situ | CG31006 | - | -23759 | -34707 | UPSTREAM | CG31006-RB | "-" | CG31006-RA | "-"
 CG1480 in-situ | bnk | + | 362 | 1933 | UPSTREAM | CG1480-RA | "-"

*********************** Rank 662 [Score  7.806900] GBROWSE*******************

 CG32172 in-situ | noe | - | -11763 | -13674 | UPSTREAM | CG32172-RA | "-"
 CG3897 in-situ | blot | - | 8291 | -17197 | INTRAGENIC | intron:CG3897-RD:1 | intron:CG3897-RB:1 | CG3897-RD | "-" | CG3897-RB | "-" | CG3897-RA | "-" | CG3897-RC | "-"

*********************** Rank 663 [Score  7.806400] GBROWSE*******************

 CG31394 in-situ | CG31394 | - | -74554 | -75292 | UPSTREAM | CG31394-RA | "-"
insitu highlight CG17117 in-situ | hth | - | 34954 | -93870 | INTRAGENIC | intron:CG17117-RD:3 | intron:CG17117-RB:2 | intron:CG17117-RC:2 | intron:CG17117-RA:1 | CG17117-RD | "-" | CG17117-RB | "-" | CG17117-RC | "-" | CG17117-RA | "-"

*********************** Rank 664 [Score  7.806400] GBROWSE*******************

 CG31834 in-situ | BG:DS00929.16 | + | -6665 | -1340 | DOWNSTREAM | CG31834-RA | "-"
 CG10846 in-situ | dynactin-subunit-p25 | + | 4228 | 5099 | UPSTREAM | CG10846-RA | "-"

*********************** Rank 665 [Score  7.806100] GBROWSE*******************

 CG31813 in-situ | CG31813 | + | -31058 | -30405 | DOWNSTREAM | CG31813-RA | "-"
 CG7364 in-situ | BG:DS00797.1 | + | 3889 | 7112 | UPSTREAM | CG7364-RA | "-"

*********************** Rank 666 [Score  7.805100] GBROWSE*******************

 CG12901 in-situ | CG12901 | - | -3479 | -5048 | UPSTREAM | CG12901-RA | "-" | CG12901-RC | "-" | CG12901-RB | "-"
 CG12906 in-situ | Gr47a | + | 1088 | 2367 | UPSTREAM | CG12906-RA | "-"

*********************** Rank 667 [Score  7.803100] GBROWSE*******************

 CG4192 in-situ | kek3 | + | -15863 | -11790 | DOWNSTREAM | CG4192-RA | "-"
 CG15255 in-situ | BG:BACR44L22.1 | - | 17275 | 16084 | DOWNSTREAM | CG15255-RA | "-"

*********************** Rank 668 [Score  7.801900] GBROWSE*******************

 CG18812 in-situ | CG18812 | - | -35830 | -53003 | UPSTREAM | CG18812-RC | "-" | CG18812-RB | "-" | CG18812-RA | "-"
insitu CG30497 in-situ | CG30497 | - | 11165 | -33699 | INTRAGENIC | intron:CG30497-RA:2 | CG30497-RA | "-" | CG30497-RB | "-" | CG30497-RC | "-"

*********************** Rank 669 [Score  7.801700] GBROWSE*******************

 CG12492 in-situ | CG12492 | + | -33015 | -29072 | DOWNSTREAM | CG12492-RA | "-"
 CG5212 in-situ | Pli | - | 11654 | -18267 | INTRAGENIC | intron:CG5212-RA:2 | CG5212-RA | "-"

*********************** Rank 670 [Score  7.800400] GBROWSE*******************

 CG14877 in-situ | CG14877 | - | -3995 | -4916 | UPSTREAM | CG14877-RA | "-"
 CG9734 in-situ | glob1 | - | 8394 | 2816 | DOWNSTREAM | CG9734-RC | "-" | CG9734-RD | "-" | CG9734-RB | "-" | CG9734-RA | "-"


*********************** Rank 671 [Score  7.793000] GBROWSE*******************

 CG9781 in-situ | CG9781 | - | -459 | -1353 | UPSTREAM | CG9781-RA | "-"
 CG7248 in-situ | CG7248 | + | 334 | 3032 | UPSTREAM | CG7248-RA | "-"

*********************** Rank 672 [Score  7.791900] GBROWSE*******************

 CG17697 in-situ | fz | + | -38323 | 55978 | INTRAGENIC | intron:CG17697-RB:3 | intron:CG17697-RA:3 | CG17697-RB | "-" | CG17697-RA | "-"
 CG13482 in-situ | CG13482 | + | 16767 | 17075 | UPSTREAM | CG13482-RA | "-"

*********************** Rank 673 [Score  7.791100] GBROWSE*******************

 CG14162 in-situ | CG14162 | + | -68402 | -1713 | DOWNSTREAM | CG14162-RA | "-"
 CG14160 in-situ | CG14160 | - | 46108 | 44121 | DOWNSTREAM | CG14160-RA | "-"

*********************** Rank 674 [Score  7.789100] GBROWSE*******************

insitu CG4951 in-situ | CG4951 | - | -450 | -1860 | UPSTREAM | CG4951-RA | "-"
 CG5527 in-situ | CG5527 | + | 459 | 2676 | UPSTREAM | CG5527-RA | "-"

*********************** Rank 675 [Score  7.785700] GBROWSE*******************

 CG31171 in-situ | CG31171 | - | -35594 | -36249 | UPSTREAM | CG31171-RA | "-"
 CG18389 in-situ | Eip93F | + | 7212 | 43774 | UPSTREAM | CG18389-RA | "-"

*********************** Rank 676 [Score  7.785600] GBROWSE*******************

 CG31769 in-situ | CG31769 | - | -14059 | -15207 | UPSTREAM | CG31769-RA | "-"
 CG15292 in-situ | CG15292 | - | 2367 | 2197 | DOWNSTREAM | CG15292-RA | "-"

*********************** Rank 677 [Score  7.782100] GBROWSE*******************

 CG13712 in-situ | CG13712 | - | -12653 | -13006 | UPSTREAM | CG13712-RA | "-"
 CG12493 in-situ | CG12493 | - | 31209 | 29884 | DOWNSTREAM | CG12493-RA | "-"


*********************** Rank 678 [Score  7.781900] GBROWSE*******************

 CG17686 in-situ | DIP1 | - | -31082 | -36444 | UPSTREAM | CG17686-RA | "-" | CG17686-RB | "-" | CG17686-RC | "-" | CG17686-RD | "-"
 CG14621 in-situ | CG14621 | + | 44469 | 46896 | UPSTREAM | CG14621-RA | "-"

*********************** Rank 679 [Score  7.780700] GBROWSE*******************

 CG7515 in-situ | CG7515 | + | -520 | 1063 | INTRAGENIC | intron:CG7515-RA:1 | CG7515-RA | "-"
insitu CG33167 in-situ | CG33167 | + | 3694 | 23010 | UPSTREAM | CG33167-RB | "-"

*********************** Rank 680 [Score  7.779700] GBROWSE*******************

 CG14661 in-situ | CG14661 | - | -2276 | -3073 | UPSTREAM | CG14661-RA | "-"
insitu CG2016 in-situ | CG2016 | - | 4667 | 314 | DOWNSTREAM | CG2016-RB | "-"

*********************** Rank 681 [Score  7.777800] GBROWSE*******************

 CG32600 in-situ | CG32600 | + | -33010 | 107135 | INTRAGENIC | intron:CG32600-RA:2 | CG32600-RA | "-"
 CG32601 in-situ | CG32601 | + | 23640 | 24589 | UPSTREAM | CG32601-RA | "-"


*********************** Rank 682 [Score  7.776900] GBROWSE*******************

 CG7815 in-situ | ran-like | - | -319 | -1328 | UPSTREAM | CG7815-RA | "-"
 CG13455 in-situ | CG13455 | + | 8021 | 12335 | UPSTREAM | CG13455-RA | "-"

*********************** Rank 683 [Score  7.776800] GBROWSE*******************

 CG31481 in-situ | CG31481 | - | -21801 | -26619 | UPSTREAM | CG31481-RA | "-"
 CG1048 in-situ | zen2 | - | 9313 | 8305 | DOWNSTREAM | CG1048-RA | "-"

*********************** Rank 684 [Score  7.776500] GBROWSE*******************

insitu CG5226 in-situ | CG5226 | + | -454 | 10404 | INTRAGENIC | intron:CG5226-RA:1 | CG5226-RA | "-"
insitu CG10924 in-situ | CG10924 | + | 12402 | 19620 | UPSTREAM | CG10924-RA | "-"

*********************** Rank 685 [Score  7.775500] GBROWSE*******************

 CG5481 in-situ | lea | - | -19535 | -59096 | UPSTREAM | CG5481-RA | "-"
 CG31925 in-situ | CG31925 | - | 34059 | 33366 | DOWNSTREAM | CG31925-RA | "-"

*********************** Rank 686 [Score  7.775100] GBROWSE*******************

 CG11769 in-situ | CG11769 | + | -1460 | -714 | DOWNSTREAM | CG11769-RA | "-"
 CG31448 in-situ | CG31448 | + | 28708 | 29400 | UPSTREAM | CG31448-RA | "-"

*********************** Rank 687 [Score  7.773900] GBROWSE*******************

 CG14351 in-situ | CG14351 | + | -23625 | 28833 | INTRAGENIC | intron:CG14351-RA:2 | CG14351-RA | "-"
 CG10869 in-situ | CG10869 | - | 8486 | 6074 | DOWNSTREAM | CG10869-RA | "-"

*********************** Rank 688 [Score  7.769800] GBROWSE*******************

 CG13728 in-situ | CG13728 | - | -7036 | -9697 | UPSTREAM | CG13728-RA | "-"
 CG13731 in-situ | CG13731 | - | 177 | -5700 | INTRAGENIC | intron:CG13731-RA:1 | CG13731-RA | "-"

*********************** Rank 689 [Score  7.769300] GBROWSE*******************

 CG15696 in-situ | CG15696 | + | -1400 | -861 | DOWNSTREAM | CG15696-RA | "-"
 CG15697 in-situ | CG15697 | + | 2038 | 2923 | UPSTREAM | CG15697-RA | "-" | CG15697-RB | "-"

*********************** Rank 690 [Score  7.766200] GBROWSE*******************

 CG32725 in-situ | CG32725 | - | -27912 | -28639 | UPSTREAM | CG32725-RA | "-"
 CG1958 in-situ | CG1958 | + | 831 | 1808 | UPSTREAM | CG1958-RA | "-"

*********************** Rank 691 [Score  7.765300] GBROWSE*******************

 CG12217 in-situ | PpV | - | -67083 | -68865 | UPSTREAM | CG12217-RA | "-"
 CG3367 in-situ | CG3367 | - | 28904 | 27110 | DOWNSTREAM | CG3367-RA | "-"

*********************** Rank 692 [Score  7.761400] GBROWSE*******************

 CG17686 in-situ | DIP1 | - | -49182 | -54544 | UPSTREAM | CG17686-RA | "-" | CG17686-RB | "-" | CG17686-RC | "-" | CG17686-RD | "-"
 CG14621 in-situ | CG14621 | + | 26369 | 28796 | UPSTREAM | CG14621-RA | "-"

*********************** Rank 693 [Score  7.754200] GBROWSE*******************

 CG32252 in-situ | CG32252 | - | -10190 | -11235 | UPSTREAM | CG32252-RA | "-"
insitu highlight CG15009 in-situ | ImpL2 | - | 3223 | -8225 | INTRAGENIC | intron:CG15009-RA:1 | CG15009-RA | "-" | CG15009-RB | "-" | CG15009-RC | "-"


*********************** Rank 694 [Score  7.754000] GBROWSE*******************

insitu highlight CG3242 in-situ | sob | - | -17515 | -20271 | UPSTREAM | CG3242-RA | "-"
insitu highlight CG3851 in-situ | odd | - | 8467 | 5941 | DOWNSTREAM | CG3851-RA | "-"

*********************** Rank 695 [Score  7.751800] GBROWSE*******************

 CG10174 in-situ | CG10174 | + | -15895 | -15285 | DOWNSTREAM | CG10174-RA | "-"
insitu highlight CG10283 in-situ | CG10283 | - | 13894 | 2333 | DOWNSTREAM | CG10283-RA | "-" | CG10283-RB | "-"


*********************** Rank 696 [Score  7.749900] GBROWSE*******************

insitu highlight CG1897 in-situ | Dr | + | -11725 | -2827 | DOWNSTREAM | CG1897-RA | "-"
insitu CG7567 in-situ | CG7567 | - | 19988 | 19124 | DOWNSTREAM | CG7567-RA | "-"

*********************** Rank 697 [Score  7.748500] GBROWSE*******************

insitu CG1049 in-situ | Cct1 | + | -690 | 6464 | INTRAGENIC | intron:CG1049-RA:1 | CG1049-RA | "-" | CG1049-RD | "-" | CG1049-RB | "-" | CG1049-RC | "-"
 CG18330 in-situ | Cct2 | + | 6722 | 8554 | UPSTREAM | CG18330-RA | "-"

*********************** Rank 698 [Score  7.746600] GBROWSE*******************

 CG30375 in-situ | CG30375 | - | -1221 | -2621 | UPSTREAM | CG30375-RA | "-"
 CG30371 in-situ | CG30371 | - | 805 | -828 | INTRAGENIC | intron:CG30371-RA:2 | CG30371-RA | "-"

*********************** Rank 699 [Score  7.745500] GBROWSE*******************

 CG12605 in-situ | CG12605 | - | -1550 | -9481 | UPSTREAM | CG12605-RB | "-"
 CG1130 in-situ | scrt | + | 17595 | 22268 | UPSTREAM | CG1130-RA | "-"

*********************** Rank 700 [Score  7.745100] GBROWSE*******************

 CG32698 in-situ | CG32698 | + | -77434 | -15775 | DOWNSTREAM | CG32698-RA | "-"
 CG2045 in-situ | Ser7 | + | 213 | 2065 | UPSTREAM | CG2045-RA | "-"

*********************** Rank 701 [Score  7.744900] GBROWSE*******************

 CG7571 in-situ | CG7571 | + | -1682 | 22872 | INTRAGENIC | intron:CG7571-RA:2 | CG7571-RA | "-"
 CG32185 in-situ | CG32185 | + | 6068 | 6415 | UPSTREAM | CG32185-RA | "-"

*********************** Rank 702 [Score  7.738200] GBROWSE*******************

 CG7527 in-situ | CadN2 | - | -75563 | -101562 | UPSTREAM | CG7527-RA | "-"
 CG5674 in-situ | CG5674 | + | 75171 | 86703 | UPSTREAM | CG5674-RA | "-" | CG5674-RB | "-" | CG5674-RC | "-"

*********************** Rank 703 [Score  7.737300] GBROWSE*******************

 CG13130 in-situ | CG13130 | - | -10784 | -11780 | UPSTREAM | CG13130-RA | "-"
 CG31875 in-situ | CG31875 | - | 1131 | 120 | DOWNSTREAM | CG31875-RA | "-"

*********************** Rank 704 [Score  7.734200] GBROWSE*******************

 CG7344 in-situ | CG7344 | + | -4899 | 12454 | INTRAGENIC | intron:CG7344-RA:1 | CG7344-RA | "-"
 CG6240 in-situ | CG6240 | - | 24362 | 23652 | DOWNSTREAM | CG6240-RA | "-"

*********************** Rank 705 [Score  7.731900] GBROWSE*******************

 CG5151 in-situ | CG5151 | - | -36137 | -45388 | UPSTREAM | CG5151-RA | "-"
 CG13073 in-situ | CG13073 | - | 29235 | 28180 | DOWNSTREAM | CG13073-RB | "-" | CG13073-RA | "-"

*********************** Rank 706 [Score  7.731400] GBROWSE*******************

 CG5977 in-situ | CG5977 | + | -6653 | -2217 | DOWNSTREAM | CG5977-RA | "-" | CG5977-RB | "-"
 CG5986 in-situ | BEST:LP03871 | + | 2919 | 4298 | UPSTREAM | CG5986-RA | "-"

*********************** Rank 707 [Score  7.730100] GBROWSE*******************

 CG15013 in-situ | CG15013 | - | -3213 | -16929 | UPSTREAM | CG15013-RB | "-" | CG15013-RA | "-" | CG15013-RC | "-"
 CG32251 in-situ | CG32251 | + | 2711 | 7549 | UPSTREAM | CG32251-RA | "-"

*********************** Rank 708 [Score  7.729400] GBROWSE*******************

 CG32336 in-situ | CG32336 | - | -13635 | -14338 | UPSTREAM | CG32336-RA | "-"
 CG9102 in-situ | bab2 | - | 44653 | 7828 | DOWNSTREAM | CG9102-RA | "-"

*********************** Rank 709 [Score  7.727100] GBROWSE*******************

 CG16741 in-situ | CG16741 | + | -852 | -292 | DOWNSTREAM | CG16741-RA | "-"
 CG11192 in-situ | CG11192 | - | 7135 | 6326 | DOWNSTREAM | CG11192-RA | "-"

*********************** Rank 710 [Score  7.726900] GBROWSE*******************

insitu CG3619 in-situ | Dl | - | -40598 | -64080 | UPSTREAM | CG3619-RA | "-" | CG3619-RB | "-"
 CG3581 in-situ | CG3581 | - | 7938 | 6946 | DOWNSTREAM | CG3581-RA | "-"


*********************** Rank 711 [Score  7.726700] GBROWSE*******************

 CG12870 in-situ | CG12870 | + | -59938 | -21736 | DOWNSTREAM | CG12870-RA | "-"
 CG12425 in-situ | CG12425 | + | 39312 | 40867 | UPSTREAM | CG12425-RA | "-"

*********************** Rank 712 [Score  7.726600] GBROWSE*******************

 CG13003 in-situ | CG13003 | - | -28094 | -36525 | UPSTREAM | CG13003-RA | "-"
 CG4872 in-situ | CG4872 | + | 40 | 1468 | UPSTREAM | CG4872-RA | "-"

*********************** Rank 713 [Score  7.726400] GBROWSE*******************

 CG7806 in-situ | CG7806 | + | -6000 | -402 | DOWNSTREAM | CG7806-RA | "-"
 CG7795 in-situ | CG7795 | - | 2501 | -712 | INTRAGENIC | intron:CG7795-RA:4 | intron:CG7795-RB:5 | CG7795-RA | "-" | CG7795-RB | "-"

*********************** Rank 714 [Score  7.726400] GBROWSE*******************

 CG30222 in-situ | CG30222 | + | -18699 | -16908 | DOWNSTREAM | CG30222-RB | "-" | CG30222-RD | "-"
 CG10433 in-situ | CG10433 | - | 5054 | 3769 | DOWNSTREAM | CG10433-RA | "-" | CG10433-RB | "-"


*********************** Rank 715 [Score  7.723000] GBROWSE*******************

insitu CG1520 in-situ | WASp | - | -10627 | -18156 | UPSTREAM | CG1520-RA | "-" | CG1520-RB | "-" | CG1520-RC | "-"
 CG1451 in-situ | Apc | - | 1988 | -10362 | INTRAGENIC | intron:CG1451-RA:1 | CG1451-RA | "-"

*********************** Rank 716 [Score  7.720300] GBROWSE*******************

 CG15283 in-situ | BG:DS08340.1 | - | -34832 | -38271 | UPSTREAM | CG15283-RA | "-"
insitu highlight CG4491 in-situ | noc | + | 5214 | 8371 | UPSTREAM | CG4491-RA | "-"

*********************** Rank 717 [Score  7.719600] GBROWSE*******************

insitu CG12214 in-situ | CG12214 | - | -4987 | -7497 | UPSTREAM | CG12214-RB | "-" | CG12214-RA | "-"
insitu CG33183 in-situ | Hr46 | - | 69524 | 41569 | DOWNSTREAM | CG33183-RC | "-" | CG33183-RB | "-" | CG33183-RA | "-"

*********************** Rank 718 [Score  7.719500] GBROWSE*******************

insitu CG13699 in-situ | CG13699 | - | -19506 | -27128 | UPSTREAM | CG13699-RA | "-"
 CG5123 in-situ | W | - | 3616 | -14279 | INTRAGENIC | intron:CG5123-RA:1 | CG5123-RA | "-"

*********************** Rank 719 [Score  7.716700] GBROWSE*******************

 CG12919 in-situ | eiger | + | -2302 | -756 | DOWNSTREAM | CG12919-RA | "-"
 CG1380 in-situ | sut4 | - | 2210 | 523 | DOWNSTREAM | CG1380-RB | "-" | CG1380-RA | "-"

*********************** Rank 720 [Score  7.714000] GBROWSE*******************

 CG32655 in-situ | CG32655 | - | -66861 | -67919 | UPSTREAM | CG32655-RA | "-"
 CG2577 in-situ | CG2577 | + | 70621 | 71861 | UPSTREAM | CG2577-RA | "-"

*********************** Rank 721 [Score  7.712400] GBROWSE*******************

insitu CG14902 in-situ | decay | - | -5438 | -8052 | UPSTREAM | CG14902-RA | "-"
 CG11769 in-situ | CG11769 | + | 6340 | 7086 | UPSTREAM | CG11769-RA | "-"

*********************** Rank 722 [Score  7.710900] GBROWSE*******************

insitu highlight CG9598 in-situ | CG9598 | - | -82838 | -87347 | UPSTREAM | CG9598-RA | "-"
 CG9587 in-situ | CG9587 | - | 13078 | 7436 | DOWNSTREAM | CG9587-RA | "-"

*********************** Rank 723 [Score  7.710300] GBROWSE*******************

 CG31760 in-situ | CG31760 | + | -8795 | 18642 | INTRAGENIC | intron:CG31760-RA:2 | CG31760-RA | "-"
 CG31861 in-situ | CG31861 | + | 10309 | 10916 | UPSTREAM | CG31861-RA | "-"

*********************** Rank 724 [Score  7.709700] GBROWSE*******************

 CG2022 in-situ | CG2022 | - | -36679 | -38766 | UPSTREAM | CG2022-RA | "-"
insitu highlight CG2530 in-situ | corto | - | 61255 | 58190 | DOWNSTREAM | CG2530-RA | "-"

*********************** Rank 725 [Score  7.707100] GBROWSE*******************

 CG12610 in-situ | CG12610 | - | -10464 | -11271 | UPSTREAM | CG12610-RA | "-"
insitu CG6361 in-situ | CG6361 | - | 10754 | 7111 | DOWNSTREAM | CG6361-RA | "-"

*********************** Rank 726 [Score  7.703700] GBROWSE*******************

 CG1262 in-situ | Acp62F | + | -24421 | -17268 | DOWNSTREAM | CG1262-RA | "-"
 CG32296 in-situ | CG32296 | - | 2309 | -44486 | INTRAGENIC | intron:CG32296-RA:1 | CG32296-RA | "-"

*********************** Rank 727 [Score  7.703300] GBROWSE*******************

 CG13966 in-situ | CG13966 | - | -21057 | -22836 | UPSTREAM | CG13966-RA | "-"
 CG10659 in-situ | CG10659 | - | 32908 | 32240 | DOWNSTREAM | CG10659-RA | "-"

*********************** Rank 728 [Score  7.703200] GBROWSE*******************

 CG9570 in-situ | CG9570 | + | -37997 | -36769 | DOWNSTREAM | CG9570-RA | "-"
 CG9571 in-situ | CG9571 | - | 3094 | 2312 | DOWNSTREAM | CG9571-RA | "-"

*********************** Rank 729 [Score  7.701800] GBROWSE*******************

 CG9965 in-situ | CG9965 | + | -5558 | -2788 | DOWNSTREAM | CG9965-RA | "-"
 CG9969 in-situ | Or63a | + | 1423 | 3295 | UPSTREAM | CG9969-RA | "-"

*********************** Rank 730 [Score  7.698400] GBROWSE*******************

 CG1976 in-situ | RhoGAP100F | + | -119385 | -107003 | DOWNSTREAM | CG1976-RA | "-"
insitu CG2003 in-situ | CG2003 | + | 25868 | 38654 | UPSTREAM | CG2003-RA | "-" | CG2003-RB | "-"


*********************** Rank 731 [Score  7.697300] GBROWSE*******************

 CG14754 in-situ | CG14754 | + | -4884 | -4540 | DOWNSTREAM | CG14754-RA | "-"
 CG12780 in-situ | CG12780 | - | 13250 | 12948 | DOWNSTREAM | CG12780-RA | "-"

*********************** Rank 732 [Score  7.694500] GBROWSE*******************

 CG33047 in-situ | CG33047 | - | -35005 | -38921 | UPSTREAM | CG33047-RA | "-" | CG33047-RB | "-" | CG33047-RC | "-"
 CG11711 in-situ | CG11711 | - | 4515 | -34961 | INTRAGENIC | intron:CG11711-RC:1 | intron:CG11711-RD:1 | CG11711-RC | "-" | CG11711-RD | "-" | CG11711-RA | "-" | CG11711-RB | "-"

*********************** Rank 733 [Score  7.691900] GBROWSE*******************

 CG30084 in-situ | CG30084 | - | -17762 | -70110 | UPSTREAM | CG30084-RA | "-" | CG30084-RB | "-" | CG30084-RC | "-" | CG30084-RD | "-"
 CG8246 in-situ | Poxn | - | 2100 | -5982 | INTRAGENIC | intron:CG8246-RA:1 | CG8246-RA | "-"


*********************** Rank 734 [Score  7.691600] GBROWSE*******************

 CG6007 in-situ | gatA | - | -25 | -2693 | UPSTREAM | CG6007-RA | "-"
insitu CG6009 in-situ | P5cr | + | 76 | 1319 | UPSTREAM | CG6009-RA | "-"

*********************** Rank 735 [Score  7.689900] GBROWSE*******************

 CG17686 in-situ | DIP1 | - | -6782 | -12144 | UPSTREAM | CG17686-RA | "-" | CG17686-RB | "-" | CG17686-RC | "-" | CG17686-RD | "-"
 CG14621 in-situ | CG14621 | + | 68769 | 71196 | UPSTREAM | CG14621-RA | "-"

*********************** Rank 736 [Score  7.689800] GBROWSE*******************

 CG1031 in-situ | alpha-Est1 | - | -19537 | -22932 | UPSTREAM | CG1031-RA | "-"
 CG32465 in-situ | CG32465 | - | 12606 | 2631 | DOWNSTREAM | CG32465-RB | "-"

*********************** Rank 737 [Score  7.688400] GBROWSE*******************

 CG12432 in-situ | CG12432 | - | -20123 | -21913 | UPSTREAM | CG12432-RA | "-"
 CG8527 in-situ | CG8527 | - | 14394 | 12109 | DOWNSTREAM | CG8527-RA | "-"


*********************** Rank 738 [Score  7.686800] GBROWSE*******************

 CG10349 in-situ | CG10349 | + | -7085 | -1703 | DOWNSTREAM | CG10349-RA | "-" | CG10349-RB | "-"
 CG31270 in-situ | CG31270 | - | 36972 | 35856 | DOWNSTREAM | CG31270-RA | "-"

*********************** Rank 739 [Score  7.685300] GBROWSE*******************

 CG31759 in-situ | CG31759 | + | -24352 | -21860 | DOWNSTREAM | CG31759-RB | "-" | CG31759-RA | "-"
 CG31862 in-situ | CG31862 | + | 106891 | 107439 | UPSTREAM | CG31862-RA | "-"


*********************** Rank 740 [Score  7.684600] GBROWSE*******************

 CG18023 in-situ | Eip78C | + | -27655 | 10047 | INTRAGENIC | intron:CG18023-RA:3 | intron:CG18023-RB:3 | CG18023-RA | "-" | CG18023-RB | "-"
insitu CG9391 in-situ | CG9391 | - | 13766 | 12204 | DOWNSTREAM | CG9391-RB | "-" | CG9391-RA | "-"

*********************** Rank 741 [Score  7.683800] GBROWSE*******************

 CG31069 in-situ | CG31069 | + | -17195 | -15792 | DOWNSTREAM | CG31069-RA | "-"
 CG31066 in-situ | CG31066 | + | 2854 | 3475 | UPSTREAM | CG31066-RA | "-"

*********************** Rank 742 [Score  7.679500] GBROWSE*******************

 CG5811 in-situ | NepYr | + | -2945 | 27076 | INTRAGENIC | intron:CG5811-RA:3 | CG5811-RA | "-"
 CG5812 in-situ | GCR(ich) | + | 18362 | 20157 | UPSTREAM | CG5812-RA | "-"

*********************** Rank 743 [Score  7.677400] GBROWSE*******************

 CG1817 in-situ | Ptp10D | + | -27649 | 26595 | INTRAGENIC | intron:CG1817-RB:2 | intron:CG1817-RC:1 | CG1817-RB | "-" | CG1817-RC | "-"
 CG2371 in-situ | CG2371 | - | 28893 | 27685 | DOWNSTREAM | CG2371-RA | "-" | CG2371-RB | "-" | CG2371-RC | "-"


*********************** Rank 744 [Score  7.674300] GBROWSE*******************

 CG11342 in-situ | CG11342 | - | -11043 | -11759 | UPSTREAM | CG11342-RA | "-"
insitu CG18314 in-situ | CG18314 | - | 1919 | -9343 | INTRAGENIC | intron:CG18314-RA:2 | CG18314-RA | "-"

*********************** Rank 745 [Score  7.674200] GBROWSE*******************

 CG5770 in-situ | CG5770 | - | -1356 | -2370 | UPSTREAM | CG5770-RA | "-"
 CG5767 in-situ | CG5767 | - | 1046 | 20 | DOWNSTREAM | CG5767-RA | "-"

*********************** Rank 746 [Score  7.673900] GBROWSE*******************

insitu CG11387 in-situ | ct | + | -45725 | 21150 | INTRAGENIC | intron:CG11387-RA:2 | CG11387-RA | "-" | CG11387-RB | "-"
 CG12690 in-situ | CHES-1-like | - | 41821 | 30014 | DOWNSTREAM | CG12690-RA | "-"

*********************** Rank 747 [Score  7.670800] GBROWSE*******************

 CG15734 in-situ | CG15734 | - | -6396 | -6787 | UPSTREAM | CG15734-RA | "-"
 CG11356 in-situ | CG11356 | - | 10389 | 9748 | DOWNSTREAM | CG11356-RA | "-"

*********************** Rank 748 [Score  7.669900] GBROWSE*******************

 CG7720 in-situ | CG7720 | - | -44707 | -80627 | UPSTREAM | CG7720-RB | "-" | CG7720-RA | "-"
 CG18208 in-situ | CG18208 | - | 8530 | -33710 | INTRAGENIC | intron:CG18208-RA:1 | CG18208-RA | "-"

*********************** Rank 749 [Score  7.668500] GBROWSE*******************

 CG13334 in-situ | CG13334 | + | -3341 | -1781 | DOWNSTREAM | CG13334-RA | "-"
 CG13335 in-situ | CG13335 | - | 12343 | 9498 | DOWNSTREAM | CG13335-RA | "-" | CG13335-RB | "-"

*********************** Rank 750 [Score  7.667900] GBROWSE*******************

 CG31146 in-situ | CG31146 | + | -53560 | -17491 | DOWNSTREAM | CG31146-RD | "-"
 CG2616 in-situ | CG2616 | + | 10604 | 12605 | UPSTREAM | CG2616-RA | "-"

*********************** Rank 751 [Score  7.666700] GBROWSE*******************

 CG32198 in-situ | CG32198 | + | -14930 | -14520 | DOWNSTREAM | CG32198-RB | "-"
 CG7285 in-situ | Drostar1 | + | 31967 | 33490 | UPSTREAM | CG7285-RA | "-"

*********************** Rank 752 [Score  7.666100] GBROWSE*******************

insitu CG12701 in-situ | CG12701 | - | -23862 | -30803 | UPSTREAM | CG12701-RB | "-" | CG12701-RA | "-"
 CG12700 in-situ | skpD | + | 5939 | 6598 | UPSTREAM | CG12700-RA | "-"

*********************** Rank 753 [Score  7.663300] GBROWSE*******************

 CG13789 in-situ | CG13789 | - | -6431 | -7068 | UPSTREAM | CG13789-RA | "-"
 CG13790 in-situ | CG13790 | - | 25799 | 25515 | DOWNSTREAM | CG13790-RA | "-"


*********************** Rank 754 [Score  7.663100] GBROWSE*******************

 CG6142 in-situ | CG6142 | + | -3468 | -1505 | DOWNSTREAM | CG6142-RA | "-"
insitu CG31323 in-situ | CG31323 | + | 32386 | 46884 | UPSTREAM | CG31323-RA | "-"

*********************** Rank 755 [Score  7.660800] GBROWSE*******************

 CG3017 in-situ | Alas | + | -2481 | -434 | DOWNSTREAM | CG3017-RA | "-"
 CG3029 in-situ | or | + | 762 | 1762 | UPSTREAM | CG3029-RA | "-"

*********************** Rank 756 [Score  7.660200] GBROWSE*******************

 CG3629 in-situ | Dll | + | -26600 | -6267 | DOWNSTREAM | CG3629-RB | "-" | CG3629-RA | "-"
 CG3650 in-situ | CG3650 | + | 7546 | 8331 | UPSTREAM | CG3650-RA | "-"


*********************** Rank 757 [Score  7.654400] GBROWSE*******************

insitu CG6883 in-situ | trh | - | -42154 | -52947 | UPSTREAM | CG6883-RA | "-"
 CG13891 in-situ | CG13891 | - | 14420 | 13759 | DOWNSTREAM | CG13891-RA | "-"

*********************** Rank 758 [Score  7.645700] GBROWSE*******************

 CG8394 in-situ | CG8394 | - | -1853 | -3574 | UPSTREAM | CG8394-RA | "-"
 CG30071 in-situ | CG30071 | + | 2083 | 2733 | UPSTREAM | CG30071-RA | "-"

*********************** Rank 759 [Score  7.643700] GBROWSE*******************

 CG2684 in-situ | lds | + | -11029 | -6974 | DOWNSTREAM | CG2684-RA | "-"
insitu CG11094 in-situ | dsx | - | 35945 | -7140 | INTRAGENIC | intron:CG11094-RA:3 | CG11094-RA | "-" | CG11094-RB | "-" | CG11094-RC | "-"

*********************** Rank 760 [Score  7.643200] GBROWSE*******************

 CG14457 in-situ | CG14457 | + | -7366 | 2712 | INTRAGENIC | intron:CG14457-RA:1 | CG14457-RA | "-"
 CG14455 in-situ | CG14455 | - | 9779 | 9112 | DOWNSTREAM | CG14455-RA | "-"

*********************** Rank 761 [Score  7.639300] GBROWSE*******************

 CG12052 in-situ | lola | - | -2278 | -62627 | UPSTREAM | CG12052-RI | "-" | CG12052-RE | "-" | CG12052-RB | "-" | CG12052-RA | "-" | CG12052-RC | "-" | CG12052-RH | "-" | CG12052-RG | "-" | CG12052-RF | "-" | CG12052-RT | "-" | CG12052-RU | "-" | CG12052-RL | "-" | CG12052-RK | "-" | CG12052-RN | "-" | CG12052-RP | "-" | CG12052-RQ | "-" | CG12052-RR | "-" | CG12052-RS | "-" | CG12052-RJ | "-" | CG12052-RO | "-" | CG12052-RM | "-" | CG12052-RD | "-"
insitu highlight CG2368 in-situ | psq | + | 12337 | 70688 | UPSTREAM | CG2368-RB | "-" | CG2368-RA | "-" | CG2368-RE | "-" | CG2368-RF | "-" | CG2368-RG | "-" | CG2368-RD | "-" | CG2368-RH | "-"


*********************** Rank 762 [Score  7.637500] GBROWSE*******************

 CG31936 in-situ | Gr22e | + | -2523 | -1299 | DOWNSTREAM | CG31936-RA | "-"
 CG31929 in-situ | Gr22c | - | 5238 | 4032 | DOWNSTREAM | CG31929-RA | "-"

*********************** Rank 763 [Score  7.636500] GBROWSE*******************

 CG14678 in-situ | CG14678 | - | -49473 | -52274 | UPSTREAM | CG14678-RA | "-"
 CG11373 in-situ | CG11373 | - | 24054 | 23392 | DOWNSTREAM | CG11373-RA | "-"

*********************** Rank 764 [Score  7.635700] GBROWSE*******************

 CG11692 in-situ | CG11692 | - | -13972 | -14528 | UPSTREAM | CG11692-RA | "-"
 CG1829 in-situ | Cyp6v1 | + | 5184 | 8653 | UPSTREAM | CG1829-RA | "-"


*********************** Rank 765 [Score  7.635600] GBROWSE*******************

 CG15570 in-situ | CG15570 | + | -21508 | -16551 | DOWNSTREAM | CG15570-RA | "-"
 CG15573 in-situ | Femcoat | - | 3073 | 2232 | DOWNSTREAM | CG15573-RB | "-"

*********************** Rank 766 [Score  7.635600] GBROWSE*******************

insitu CG10440 in-situ | CG10440 | - | -9697 | -20903 | UPSTREAM | CG10440-RA | "-"
 CG30222 in-situ | CG30222 | + | 2051 | 3842 | UPSTREAM | CG30222-RB | "-" | CG30222-RD | "-"

*********************** Rank 767 [Score  7.634400] GBROWSE*******************

 CG32231 in-situ | CG32231 | + | -6924 | -6271 | DOWNSTREAM | CG32231-RA | "-"
 CG12414 in-situ | nAcRalpha-80B | - | 45678 | 10165 | DOWNSTREAM | CG12414-RA | "-"

*********************** Rank 768 [Score  7.634300] GBROWSE*******************

 CG13952 in-situ | CG13952 | + | -3169 | -1673 | DOWNSTREAM | CG13952-RA | "-"
 CG13953 in-situ | CG13953 | + | 5972 | 6736 | UPSTREAM | CG13953-RA | "-"

*********************** Rank 769 [Score  7.633500] GBROWSE*******************

 CG7855 in-situ | timeout | + | -22658 | 52567 | INTRAGENIC | intron:CG7855-RA:10 | CG7855-RA | "-"
 CG17319 in-situ | CG17319 | - | 17240 | 14802 | DOWNSTREAM | CG17319-RA | "-"


*********************** Rank 770 [Score  7.633100] GBROWSE*******************

 CG11284 in-situ | CG11284 | + | -2421 | 4759 | INTRAGENIC | intron:CG11284-RA:1 | CG11284-RA | "-"
 CG12108 in-situ | Ppt1 | - | 6011 | 4626 | DOWNSTREAM | CG12108-RA | "-"

*********************** Rank 771 [Score  7.633000] GBROWSE*******************

 CG7527 in-situ | CadN2 | - | -4763 | -30762 | UPSTREAM | CG7527-RA | "-"
 CG5674 in-situ | CG5674 | + | 145971 | 157503 | UPSTREAM | CG5674-RA | "-" | CG5674-RB | "-" | CG5674-RC | "-"

*********************** Rank 772 [Score  7.632300] GBROWSE*******************

 CG32268 in-situ | CG32268 | - | -1059 | -1277 | UPSTREAM | CG32268-RA | "-"
 CG12008 in-situ | kst | + | 14106 | 30715 | UPSTREAM | CG12008-RA | "-"

*********************** Rank 773 [Score  7.631300] GBROWSE*******************

 CG16720 in-situ | 5-HT1A | + | -9121 | 45678 | INTRAGENIC | intron:CG16720-RB:1 | CG16720-RB | "-" | CG16720-RA | "-"
 CG30125 in-situ | CG30125 | - | 19894 | 17987 | DOWNSTREAM | CG30125-RA | "-"

*********************** Rank 774 [Score  7.628500] GBROWSE*******************

 CG32056 in-situ | CG32056 | - | -3281 | -8036 | UPSTREAM | CG32056-RA | "-" | CG32056-RB | "-" | CG32056-RC | "-"
 CG14156 in-situ | Or67c | - | 16185 | 14799 | DOWNSTREAM | CG14156-RA | "-"

*********************** Rank 775 [Score  7.618700] GBROWSE*******************

 CG7311 in-situ | BG:DS08249.2 | + | -2683 | -306 | DOWNSTREAM | CG7311-RA | "-" | CG7311-RC | "-"
 CG31814 in-situ | CG31814 | - | 6412 | -34038 | INTRAGENIC | intron:CG31814-RA:2 | CG31814-RA | "-"

*********************** Rank 776 [Score  7.618100] GBROWSE*******************

 CG14985 in-situ | CG14985 | + | -13649 | -10495 | DOWNSTREAM | CG14985-RA | "-"
insitu highlight CG1132 in-situ | fd64A | + | 1973 | 4058 | UPSTREAM | CG1132-RA | "-"

*********************** Rank 777 [Score  7.617000] GBROWSE*******************

 CG31160 in-situ | CG31160 | + | -7524 | 3418 | INTRAGENIC | intron:CG31160-RA:1 | CG31160-RA | "-"
 CG13845 in-situ | CG13845 | + | 3733 | 4628 | UPSTREAM | CG13845-RA | "-"

*********************** Rank 778 [Score  7.616900] GBROWSE*******************

 CG4680 in-situ | CG4680 | + | -3592 | -1879 | DOWNSTREAM | CG4680-RA | "-"
 CG13035 in-situ | CG13035 | - | 1836 | -1659 | INTRAGENIC | intron:CG13035-RA:5 | intron:CG13035-RB:5 | CG13035-RA | "-" | CG13035-RB | "-"

*********************** Rank 779 [Score  7.615800] GBROWSE*******************

 CG17025 in-situ | CG17025 | - | -153 | -7924 | UPSTREAM | CG17025-RA | "-"
 CG12538 in-situ | CG12538 | - | 57276 | 56692 | DOWNSTREAM | CG12538-RA | "-"

*********************** Rank 780 [Score  7.614400] GBROWSE*******************

 CG7313 in-situ | CG7313 | + | -17674 | -16925 | DOWNSTREAM | CG7313-RA | "-"
 CG5103 in-situ | CG5103 | - | 24596 | 22346 | DOWNSTREAM | CG5103-RA | "-"

*********************** Rank 781 [Score  7.613000] GBROWSE*******************

 CG12283 in-situ | kek1 | - | -20293 | -24150 | UPSTREAM | CG12283-RA | "-"
 CG5983 in-situ | ACXC | + | 69450 | 73694 | UPSTREAM | CG5983-RA | "-"

*********************** Rank 782 [Score  7.613000] GBROWSE*******************

 CG14459 in-situ | CG14459 | - | -10719 | -11458 | UPSTREAM | CG14459-RA | "-"
 CG6914 in-situ | CG6914 | + | 11997 | 12758 | UPSTREAM | CG6914-RA | "-"

*********************** Rank 783 [Score  7.610200] GBROWSE*******************

 CG30473 in-situ | Obp51a | - | -30085 | -30505 | UPSTREAM | CG30473-RA | "-"
insitu highlight CG11798 in-situ | CG11798 | + | 73991 | 89232 | UPSTREAM | CG11798-RA | "-"

*********************** Rank 784 [Score  7.609900] GBROWSE*******************

insitu CG6134 in-situ | spz | - | -2895 | -7893 | UPSTREAM | CG6134-RA | "-" | CG6134-RB | "-" | CG6134-RC | "-" | CG6134-RD | "-" | CG6134-RE | "-" | CG6134-RF | "-" | CG6134-RG | "-" | CG6134-RH | "-" | CG6134-RI | "-" | CG6134-RJ | "-"
 CG14257 in-situ | CG14257 | + | 5306 | 9254 | UPSTREAM | CG14257-RA | "-"

*********************** Rank 785 [Score  7.609200] GBROWSE*******************

 CG8339 in-situ | sfl | + | -3741 | 49775 | INTRAGENIC | intron:CG8339-RA:1 | CG8339-RA | "-"
 CG32396 in-situ | CG32396 | + | 31258 | 32958 | UPSTREAM | CG32396-RA | "-"

*********************** Rank 786 [Score  7.608900] GBROWSE*******************

insitu highlight CG2189 in-situ | Dfd | + | -53691 | -43096 | DOWNSTREAM | CG2189-RA | "-"
insitu highlight CG1030 in-situ | Scr | - | 3092 | -22407 | INTRAGENIC | intron:CG1030-RA:1 | CG1030-RA | "-"

*********************** Rank 787 [Score  7.607400] GBROWSE*******************

 CG5224 in-situ | CG5224 | + | -270 | 964 | INTRAGENIC | intron:CG5224-RA:2 | CG5224-RA | "-"
insitu CG5226 in-situ | CG5226 | + | 10696 | 21554 | UPSTREAM | CG5226-RA | "-"

*********************** Rank 788 [Score  7.605200] GBROWSE*******************

 CG12066 in-situ | Pka-C2 | - | -1474 | -3084 | UPSTREAM | CG12066-RA | "-" | CG12066-RB | "-"
 CG31010 in-situ | CG31010 | - | 7481 | 6147 | DOWNSTREAM | CG31010-RA | "-"

*********************** Rank 789 [Score  7.600600] GBROWSE*******************

 CG3151 in-situ | Rbp9 | + | -7269 | 2822 | INTRAGENIC | intron:CG3151-RA:4 | intron:CG3151-RD:4 | intron:CG3151-RB:5 | intron:CG3151-RE:5 | intron:CG3151-RC:5 | intron:CG3151-RF:5 | CG3151-RA | "-" | CG3151-RD | "-" | CG3151-RB | "-" | CG3151-RE | "-" | CG3151-RC | "-" | CG3151-RF | "-"
insitu CG3181 in-situ | Ts | - | 7589 | 6262 | DOWNSTREAM | CG3181-RA | "-"

*********************** Rank 790 [Score  7.596600] GBROWSE*******************

insitu CG10036 in-situ | otp | - | -873 | -13964 | UPSTREAM | CG10036-RA | "-"
 CG9235 in-situ | CG9235 | + | 11248 | 12352 | UPSTREAM | CG9235-RA | "-"

*********************** Rank 791 [Score  7.594900] GBROWSE*******************

 CG12295 in-situ | CG12295 | + | -7112 | 6989 | INTRAGENIC | intron:CG12295-RB:8 | CG12295-RB | "-"
 CG6315 in-situ | fl(2)d | - | 11968 | 7038 | DOWNSTREAM | CG6315-RA | "-" | CG6315-RB | "-"

*********************** Rank 792 [Score  7.592700] GBROWSE*******************

 CG12370 in-situ | CG12370 | + | -5580 | -3850 | DOWNSTREAM | CG12370-RA | "-" | CG12370-RB | "-"
 CG30044 in-situ | CG30044 | + | 5588 | 11530 | UPSTREAM | CG30044-RA | "-"

*********************** Rank 793 [Score  7.591800] GBROWSE*******************

 CG10950 in-situ | CG10950 | + | -47372 | -45411 | DOWNSTREAM | CG10950-RA | "-"
 CG18469 in-situ | CG18469 | - | 3702 | 3190 | DOWNSTREAM | CG18469-RA | "-"

*********************** Rank 794 [Score  7.591200] GBROWSE*******************

 CG1957 in-situ | BcDNA:LD14168 | + | -3759 | -1295 | DOWNSTREAM | CG1957-RA | "-" | CG1957-RB | "-"
 CG1420 in-situ | CG1420 | - | 704 | -1313 | INTRAGENIC | intron:CG1420-RA:1 | CG1420-RA | "-"

*********************** Rank 795 [Score  7.588900] GBROWSE*******************

 CG18214 in-situ | trio | - | -59046 | -97943 | UPSTREAM | CG18214-RA | "-" | CG18214-RC | "-" | CG18214-RD | "-" | CG18214-RB | "-" | CG18214-RE | "-"
 CG9097 in-situ | bab1 | - | 7168 | -57552 | INTRAGENIC | intron:CG9097-RB:1 | CG9097-RB | "-"

*********************** Rank 796 [Score  7.587500] GBROWSE*******************

insitu CG6930 in-situ | CG6930 | - | -1610 | -4547 | UPSTREAM | CG6930-RA | "-"
 CG31364 in-situ | CG31364 | - | 5944 | 4543 | DOWNSTREAM | CG31364-RA | "-"

*********************** Rank 797 [Score  7.587000] GBROWSE*******************

 CG9700 in-situ | Or85e | - | -1779 | -3193 | UPSTREAM | CG9700-RA | "-"
insitu CG9716 in-situ | Cyp313b1 | - | 7794 | 5397 | DOWNSTREAM | CG9716-RA | "-"

*********************** Rank 798 [Score  7.586900] GBROWSE*******************

 CG15415 in-situ | CG15415 | - | -295 | -3028 | UPSTREAM | CG15415-RA | "-"
 CG8853 in-situ | CG8853 | + | 322 | 1945 | UPSTREAM | CG8853-RA | "-"

*********************** Rank 799 [Score  7.584800] GBROWSE*******************

insitu CG10698 in-situ | GRHRII | - | -38717 | -51141 | UPSTREAM | CG10698-RA | "-"
 CG4357 in-situ | CG4357 | - | 1162 | -21607 | INTRAGENIC | intron:CG4357-RA:1 | CG4357-RA | "-"

*********************** Rank 800 [Score  7.582100] GBROWSE*******************

 CG9452 in-situ | CG9452 | - | -3060 | -5776 | UPSTREAM | CG9452-RA | "-"
 CG9283 in-situ | CG9283 | - | 12432 | 11818 | DOWNSTREAM | CG9283-RA | "-"


*********************** Rank 801 [Score  7.579000] GBROWSE*******************

 CG10952 in-situ | eag | + | -11430 | 22640 | INTRAGENIC | intron:CG10952-RA:1 | CG10952-RA | "-"
 CG32594 in-situ | CG32594 | + | 41748 | 69154 | UPSTREAM | CG32594-RA | "-" | CG32594-RB | "-" | CG32594-RC | "-" | CG32594-RD | "-" | CG32594-RE | "-"


*********************** Rank 802 [Score  7.575000] GBROWSE*******************

 CG8472 in-situ | Cam | + | -29579 | -14362 | DOWNSTREAM | CG8472-RA | "-" | CG8472-RB | "-"
 CG13165 in-situ | CG13165 | + | 4430 | 8726 | UPSTREAM | CG13165-RA | "-"

*********************** Rank 803 [Score  7.574900] GBROWSE*******************

 CG6928 in-situ | CG6928 | + | -16677 | -11797 | DOWNSTREAM | CG6928-RB | "-" | CG6928-RA | "-"
 CG6793 in-situ | CG6793 | + | 22557 | 24312 | UPSTREAM | CG6793-RA | "-"

*********************** Rank 804 [Score  7.573200] GBROWSE*******************

 CG11192 in-situ | CG11192 | - | -41665 | -42474 | UPSTREAM | CG11192-RA | "-"
 CG33041 in-situ | CG33041 | + | 27647 | 74276 | UPSTREAM | CG33041-RA | "-"

*********************** Rank 805 [Score  7.571700] GBROWSE*******************

 CG9397 in-situ | 1.28 | + | -73030 | -72080 | DOWNSTREAM | CG9397-RA | "-"
 CG15233 in-situ | CG15233 | - | 25180 | 24107 | DOWNSTREAM | CG15233-RA | "-"

*********************** Rank 806 [Score  7.567000] GBROWSE*******************

insitu highlight CG2328 in-situ | eve | + | -6376 | -3188 | DOWNSTREAM | CG2328-RA | "-"
 CG2331 in-situ | TER94 | + | 5194 | 9600 | UPSTREAM | CG2331-RA | "-" | CG2331-RB | "-"

note: overlaps known module eve_stripe4_6 by 500 bases (module coords: 5044532-5045133)

*********************** Rank 807 [Score  7.566500] GBROWSE*******************

 CG13858 in-situ | CG13858 | - | -1210 | -1419 | UPSTREAM | CG13858-RA | "-"
 CG7080 in-situ | CG7080 | + | 9153 | 10388 | UPSTREAM | CG7080-RA | "-"

*********************** Rank 808 [Score  7.563800] GBROWSE*******************

insitu CG10026 in-situ | CG10026 | + | -8111 | -4641 | DOWNSTREAM | CG10026-RA | "-" | CG10026-RB | "-" | CG10026-RC | "-"
 CG10034 in-situ | CG10034 | + | 2395 | 3924 | UPSTREAM | CG10034-RA | "-"

*********************** Rank 809 [Score  7.562900] GBROWSE*******************

 CG5112 in-situ | CG5112 | + | -4373 | -2600 | DOWNSTREAM | CG5112-RA | "-"
 CG5107 in-situ | CG5107 | + | 9350 | 10055 | UPSTREAM | CG5107-RA | "-"

*********************** Rank 810 [Score  7.562600] GBROWSE*******************

insitu CG15270 in-situ | BG:DS04929.1 | + | -28520 | -7250 | DOWNSTREAM | CG15270-RA | "-"
 CG15269 in-situ | BG:DS04929.3 | + | 5420 | 8749 | UPSTREAM | CG15269-RA | "-"

*********************** Rank 811 [Score  7.562600] GBROWSE*******************

 CG5734 in-situ | CG5734 | - | -2379 | -4584 | UPSTREAM | CG5734-RA | "-"
 CG5731 in-situ | CG5731 | - | 380 | -2259 | INTRAGENIC | intron:CG5731-RA:1 | CG5731-RA | "-"

*********************** Rank 812 [Score  7.562000] GBROWSE*******************

 CG32067 in-situ | CG32067 | + | -4028 | 20609 | INTRAGENIC | intron:CG32067-RB:2 | intron:CG32067-RA:1 | intron:CG32067-RC:1 | CG32067-RB | "-" | CG32067-RA | "-" | CG32067-RC | "-"
insitu CG11811 in-situ | CG11811 | + | 22246 | 23830 | UPSTREAM | CG11811-RA | "-"

*********************** Rank 813 [Score  7.561500] GBROWSE*******************

 CG11994 in-situ | Ada | + | -51334 | -50261 | DOWNSTREAM | CG11994-RA | "-"
 CG11997 in-situ | CG11997 | + | 61050 | 62360 | UPSTREAM | CG11997-RA | "-"

*********************** Rank 814 [Score  7.560500] GBROWSE*******************

 CG13726 in-situ | Or74a | - | -2952 | -4388 | UPSTREAM | CG13726-RA | "-"
 CG6479 in-situ | CG6479 | - | 7137 | 5049 | DOWNSTREAM | CG6479-RA | "-" | CG6479-RB | "-"

*********************** Rank 815 [Score  7.559600] GBROWSE*******************

 CG32682 in-situ | CG32682 | - | -8081 | -8809 | UPSTREAM | CG32682-RA | "-"
 CG32684 in-situ | alpha-Man-I | - | 9315 | -40193 | INTRAGENIC | intron:CG32684-RA:2 | CG32684-RA | "-" | CG32684-RB | "-"

*********************** Rank 816 [Score  7.556400] GBROWSE*******************

 CG10131 in-situ | CG10131 | + | -4461 | -3353 | DOWNSTREAM | CG10131-RA | "-"
 CG12862 in-situ | CG12862 | - | 6517 | 5840 | DOWNSTREAM | CG12862-RB | "-" | CG12862-RA | "-"

*********************** Rank 817 [Score  7.555500] GBROWSE*******************

 CG4841 in-situ | CG4841 | + | -57688 | -51314 | DOWNSTREAM | CG4841-RA | "-"
 CG33179 in-situ | beat-IIIb | + | 16289 | 24676 | UPSTREAM | CG33179-RA | "-"

*********************** Rank 818 [Score  7.554400] GBROWSE*******************

insitu highlight CG2102 in-situ | cas | - | -8031 | -12389 | UPSTREAM | CG2102-RA | "-" | CG2102-RB | "-"
 CG1239 in-situ | CG1239 | + | 8210 | 9371 | UPSTREAM | CG1239-RA | "-"

*********************** Rank 819 [Score  7.554100] GBROWSE*******************

 CG7075 in-situ | CG7075 | + | -6799 | -3796 | DOWNSTREAM | CG7075-RA | "-" | CG7075-RB | "-"
 CG13793 in-situ | CG13793 | - | 1237 | -767 | INTRAGENIC | intron:CG13793-RA:5 | CG13793-RA | "-"

*********************** Rank 820 [Score  7.553300] GBROWSE*******************

 CG10750 in-situ | CG10750 | + | -16811 | -15517 | DOWNSTREAM | CG10750-RA | "-"
 CG17559 in-situ | dnt | - | 46107 | 21559 | DOWNSTREAM | CG17559-RA | "-"

*********************** Rank 821 [Score  7.552700] GBROWSE*******************

 CG12523 in-situ | CG12523 | + | -44329 | -43404 | DOWNSTREAM | CG12523-RA | "-"
 CG7958 in-situ | CG7958 | + | 12371 | 29141 | UPSTREAM | CG7958-RA | "-" | CG7958-RB | "-"

*********************** Rank 822 [Score  7.551600] GBROWSE*******************

insitu highlight CG9015 in-situ | en | - | -1327 | -5533 | UPSTREAM | CG9015-RB | "-" | CG9015-RA | "-"
 CG10897 in-situ | tou | - | 86294 | 49345 | DOWNSTREAM | CG10897-RA | "-" | CG10897-RC | "-" | CG10897-RD | "-" | CG10897-RB | "-"

note: overlaps known module en_early_even+late_2 by 500 bases (module coords: 6591490-6596436)

*********************** Rank 823 [Score  7.548200] GBROWSE*******************

 CG14920 in-situ | CG14920 | - | -2521 | -3950 | UPSTREAM | CG14920-RA | "-"
 CG16854 in-situ | CG16854 | + | 640 | 3091 | UPSTREAM | CG16854-RA | "-"

*********************** Rank 824 [Score  7.546000] GBROWSE*******************

 CG13958 in-situ | CG13958 | + | -17499 | -15883 | DOWNSTREAM | CG13958-RA | "-"
 CG13959 in-situ | CG13959 | + | 41463 | 42551 | UPSTREAM | CG13959-RA | "-"

*********************** Rank 825 [Score  7.538100] GBROWSE*******************

 CG31245 in-situ | CG31245 | - | -8836 | -9866 | UPSTREAM | CG31245-RA | "-"
insitu CG3734 in-situ | CG3734 | + | 1798 | 3805 | UPSTREAM | CG3734-RA | "-"

*********************** Rank 826 [Score  7.537400] GBROWSE*******************

insitu highlight CG33207 in-situ | CG33207 | + | -10874 | 10876 | INTRAGENIC | intron:CG33207-RB:2 | CG33207-RA | "-" | CG33207-RB | "-"
 CG31446 in-situ | CG31446 | + | 16029 | 17138 | UPSTREAM | CG31446-RA | "-"

*********************** Rank 827 [Score  7.537300] GBROWSE*******************

 CG4772 in-situ | Ugt86Dh | + | -5138 | -2753 | DOWNSTREAM | CG4772-RA | "-"
 CG18577 in-situ | CG18577 | + | 15396 | 16124 | UPSTREAM | CG18577-RA | "-"

*********************** Rank 828 [Score  7.536800] GBROWSE*******************

 CG10912 in-situ | CG10912 | - | -17557 | -18553 | UPSTREAM | CG10912-RA | "-"
 CG14496 in-situ | CG14496 | + | 15412 | 16053 | UPSTREAM | CG14496-RA | "-"

*********************** Rank 829 [Score  7.536400] GBROWSE*******************

 CG15766 in-situ | CG15766 | - | -1659 | -2309 | UPSTREAM | CG15766-RA | "-"
insitu CG15765 in-situ | CG15765 | + | 1139 | 22148 | UPSTREAM | CG15765-RA | "-"

*********************** Rank 830 [Score  7.536000] GBROWSE*******************

 CG1636 in-situ | CG1636 | + | -35404 | -33437 | DOWNSTREAM | CG1636-RA | "-"
 CG15344 in-situ | CG15344 | + | 15864 | 16271 | UPSTREAM | CG15344-RA | "-"

*********************** Rank 831 [Score  7.534900] GBROWSE*******************

 CG17348 in-situ | drl | + | -13621 | 4648 | INTRAGENIC | intron:CG17348-RA:1 | CG17348-RA | "-"
 CG31797 in-situ | CG31797 | - | 58166 | 55560 | DOWNSTREAM | CG31797-RA | "-"

*********************** Rank 832 [Score  7.534700] GBROWSE*******************

 CG10174 in-situ | CG10174 | + | -24645 | -24035 | DOWNSTREAM | CG10174-RA | "-"
insitu highlight CG10283 in-situ | CG10283 | - | 5144 | -6417 | INTRAGENIC | intron:CG10283-RA:1 | intron:CG10283-RB:2 | CG10283-RA | "-" | CG10283-RB | "-"

*********************** Rank 833 [Score  7.533600] GBROWSE*******************

 CG5630 in-situ | CG5630 | + | -24810 | -11489 | DOWNSTREAM | CG5630-RA | "-"
 CG31191 in-situ | CG31191 | - | 6419 | -29898 | INTRAGENIC | intron:CG31191-RA:1 | CG31191-RA | "-"

*********************** Rank 834 [Score  7.532800] GBROWSE*******************

insitu CG32137 in-situ | CG32137 | + | -13968 | 9429 | INTRAGENIC | intron:CG32137-RA:2 | intron:CG32137-RB:2 | CG32137-RA | "-" | CG32137-RB | "-"
 CG8474 in-situ | Meics | - | 11473 | 9434 | DOWNSTREAM | CG8474-RA | "-"

*********************** Rank 835 [Score  7.532100] GBROWSE*******************

 CG14070 in-situ | CG14070 | - | -496 | -1307 | UPSTREAM | CG14070-RA | "-"
 CG7309 in-situ | CG7309 | - | 6552 | 4079 | DOWNSTREAM | CG7309-RA | "-"

*********************** Rank 836 [Score  7.530000] GBROWSE*******************

 CG17834 in-situ | CG17834 | + | -28176 | -15857 | DOWNSTREAM | CG17834-RA | "-" | CG17834-RC | "-" | CG17834-RB | "-"
 CG18405 in-situ | Sema-1a | + | 6477 | 110110 | UPSTREAM | CG18405-RA | "-"

*********************** Rank 837 [Score  7.529700] GBROWSE*******************

insitu highlight CG4345 in-situ | grim | - | -62957 | -64652 | UPSTREAM | CG4345-RA | "-"
 CG4319 in-situ | rpr | - | 31032 | 30182 | DOWNSTREAM | CG4319-RA | "-"

*********************** Rank 838 [Score  7.529400] GBROWSE*******************

insitu CG3039 in-situ | ogre | - | -2676 | -10358 | UPSTREAM | CG3039-RA | "-" | CG3039-RB | "-"
insitu highlight CG14430 in-situ | CG14430 | + | 2116 | 2829 | UPSTREAM | CG14430-RA | "-"

*********************** Rank 839 [Score  7.524100] GBROWSE*******************

 CG33047 in-situ | CG33047 | - | -15255 | -19171 | UPSTREAM | CG33047-RA | "-" | CG33047-RB | "-" | CG33047-RC | "-"
 CG11711 in-situ | CG11711 | - | 24265 | -15211 | INTRAGENIC | intron:CG11711-RC:1 | intron:CG11711-RD:1 | intron:CG11711-RA:1 | CG11711-RC | "-" | CG11711-RD | "-" | CG11711-RA | "-" | CG11711-RB | "-"

*********************** Rank 840 [Score  7.520800] GBROWSE*******************

insitu CG4843 in-situ | Tm2 | + | -5767 | -848 | DOWNSTREAM | CG4843-RA | "-" | CG4843-RB | "-"
insitu CG14866 in-situ | CG14866 | + | 513 | 2330 | UPSTREAM | CG14866-RA | "-"

*********************** Rank 841 [Score  7.519800] GBROWSE*******************

 CG32071 in-situ | CG32071 | - | -2045 | -2497 | UPSTREAM | CG32071-RA | "-"
 CG32073 in-situ | CG32073 | - | 1439 | 1110 | DOWNSTREAM | CG32073-RA | "-"

*********************** Rank 842 [Score  7.519300] GBROWSE*******************

 CG13109 in-situ | tai | + | -41417 | 38171 | INTRAGENIC | intron:CG13109-RA:2 | CG13109-RA | "-"
 CG17009 in-situ | CG17009 | - | 42793 | 42077 | DOWNSTREAM | CG17009-RA | "-"

*********************** Rank 843 [Score  7.519200] GBROWSE*******************

 CG11106 in-situ | CG11106 | + | -28542 | -28004 | DOWNSTREAM | CG11106-RA | "-"
 CG11105 in-situ | CG11105 | - | 6009 | -6682 | INTRAGENIC | intron:CG11105-RB:1 | CG11105-RB | "-" | CG11105-RA | "-"

*********************** Rank 844 [Score  7.519200] GBROWSE*******************

 CG5620 in-situ | CG5620 | - | -36799 | -45372 | UPSTREAM | CG5620-RA | "-"
 CG32098 in-situ | CG32098 | - | 6617 | 4138 | DOWNSTREAM | CG32098-RA | "-"

*********************** Rank 845 [Score  7.517600] GBROWSE*******************

 CG5227 in-situ | sdk | + | -13823 | 48835 | INTRAGENIC | intron:CG5227-RD:2 | intron:CG5227-RC:2 | intron:CG5227-RB:2 | intron:CG5227-RA:2 | CG5227-RD | "-" | CG5227-RC | "-" | CG5227-RB | "-" | CG5227-RA | "-"
insitu CG13362 in-situ | CG13362 | - | 50634 | 48572 | DOWNSTREAM | CG13362-RA | "-"

*********************** Rank 846 [Score  7.517000] GBROWSE*******************

 CG11471 in-situ | Aats-ile | + | -7860 | -3704 | DOWNSTREAM | CG11471-RA | "-" | CG11471-RC | "-" | CG11471-RD | "-"
 CG32450 in-situ | CG32450 | + | 33256 | 34155 | UPSTREAM | CG32450-RA | "-"

*********************** Rank 847 [Score  7.516600] GBROWSE*******************

 CG3022 in-situ | GABA-B-R3 | + | -13566 | -4543 | DOWNSTREAM | CG3022-RA | "-" | CG3022-RB | "-"
 CG12506 in-situ | CG12506 | + | 6890 | 7335 | UPSTREAM | CG12506-RA | "-"

*********************** Rank 848 [Score  7.515800] GBROWSE*******************

 CG4374 in-situ | CG4374 | - | -37964 | -42316 | UPSTREAM | CG4374-RA | "-"
 CG31225 in-situ | CG31225 | + | 31396 | 32970 | UPSTREAM | CG31225-RA | "-"

*********************** Rank 849 [Score  7.515100] GBROWSE*******************

 CG8808 in-situ | Pdk | + | -1426 | 5526 | INTRAGENIC | intron:CG8808-RA:1 | CG8808-RA | "-"
insitu CG11804 in-situ | ced-6 | - | 13692 | 6801 | DOWNSTREAM | CG11804-RB | "-" | CG11804-RC | "-" | CG11804-RA | "-"

*********************** Rank 850 [Score  7.513300] GBROWSE*******************

 CG31822 in-situ | CG31822 | - | -11994 | -12771 | UPSTREAM | CG31822-RA | "-"
 CG13243 in-situ | BG:DS02252.1 | + | 3307 | 4698 | UPSTREAM | CG13243-RA | "-"

*********************** Rank 851 [Score  7.510900] GBROWSE*******************

insitu CG14801 in-situ | EG:131F2.2 | + | -7756 | 13859 | INTRAGENIC | intron:CG14801-RB:1 | intron:CG14801-RD:1 | CG14801-RB | "-" | CG14801-RD | "-" | CG14801-RC | "-" | CG14801-RA | "-"
 CG14812 in-situ | EG:131F2.3 | - | 19873 | 18892 | DOWNSTREAM | CG14812-RA | "-"

*********************** Rank 852 [Score  7.509000] GBROWSE*******************

 CG31184 in-situ | CG31184 | + | -16552 | -15942 | DOWNSTREAM | CG31184-RA | "-"
insitu CG5405 in-situ | KrT95D | - | 18839 | -14534 | INTRAGENIC | intron:CG5405-RB:4 | intron:CG5405-RA:3 | CG5405-RB | "-" | CG5405-RA | "-"

*********************** Rank 853 [Score  7.508500] GBROWSE*******************

 CG15550 in-situ | CG15550 | - | -10564 | -10995 | UPSTREAM | CG15550-RA | "-"
 CG15548 in-situ | CG15548 | + | 12145 | 14589 | UPSTREAM | CG15548-RA | "-"

*********************** Rank 854 [Score  7.507600] GBROWSE*******************

insitu CG8112 in-situ | CG8112 | + | -181 | 14105 | INTRAGENIC | intron:CG8112-RA:1 | CG8112-RA | "-" | CG8112-RB | "-"
 CG9790 in-situ | CG9790 | - | 14693 | 14037 | DOWNSTREAM | CG9790-RA | "-"

note: overlaps known module hb_central_posterior_stripes by 500 bases (module coords: 4526535-4527557)

*********************** Rank 855 [Score  7.505400] GBROWSE*******************

 CG6214 in-situ | CG6214 | + | -7353 | 15186 | INTRAGENIC | intron:CG6214-RA:11 | intron:CG6214-RB:7 | CG6214-RA | "-" | CG6214-RB | "-"
 CG15483 in-situ | CG15483 | - | 32210 | 30826 | DOWNSTREAM | CG15483-RA | "-"

*********************** Rank 856 [Score  7.503800] GBROWSE*******************

 CG12693 in-situ | CG12693 | - | -10081 | -12304 | UPSTREAM | CG12693-RB | "-"
 CG3626 in-situ | CG3626 | + | 27035 | 31752 | UPSTREAM | CG3626-RA | "-"

*********************** Rank 857 [Score  7.502900] GBROWSE*******************

 CG31637 in-situ | CG31637 | + | -42692 | -14338 | DOWNSTREAM | CG31637-RA | "-"
insitu highlight CG9554 in-situ | eya | - | 5638 | -13885 | INTRAGENIC | intron:CG9554-RB:1 | CG9554-RB | "-" | CG9554-RA | "-"

*********************** Rank 858 [Score  7.500100] GBROWSE*******************

 CG32577 in-situ | disco-r | - | -78687 | -83578 | UPSTREAM | CG32577-RA | "-"
insitu highlight CG9908 in-situ | disco | - | 14853 | 8717 | DOWNSTREAM | CG9908-RA | "-"

*********************** Rank 859 [Score  7.500000] GBROWSE*******************

 CG13980 in-situ | CG13980 | + | -868 | 967 | INTRAGENIC | intron:CG13980-RA:2 | CG13980-RA | "-"
 CG12509 in-situ | CG12509 | - | 38145 | 37852 | DOWNSTREAM | CG12509-RA | "-"

*********************** Rank 860 [Score  7.490500] GBROWSE*******************

 CG3748 in-situ | CG3748 | + | -1420 | -92 | DOWNSTREAM | CG3748-RB | "-"
 CG13110 in-situ | CG13110 | + | 6062 | 6585 | UPSTREAM | CG13110-RA | "-"

*********************** Rank 861 [Score  7.489500] GBROWSE*******************

 CG32852 in-situ | CG32852 | - | -1836 | -2639 | UPSTREAM | CG32852-RA | "-"
 CG17309 in-situ | CG17309 | - | 5786 | -11632 | INTRAGENIC | intron:CG17309-RB:1 | intron:CG17309-RA:1 | CG17309-RB | "-" | CG17309-RA | "-" | CG17309-RC | "-" | CG17309-RD | "-" | CG17309-RE | "-"

*********************** Rank 862 [Score  7.487700] GBROWSE*******************

insitu highlight CG7952 in-situ | gt | - | -5215 | -7071 | UPSTREAM | CG7952-RB | "-"
 CG7925 in-situ | tko | - | 9798 | 8129 | DOWNSTREAM | CG7925-RB | "-"

*********************** Rank 863 [Score  7.485500] GBROWSE*******************

 CG7234 in-situ | Glu-RIIB | + | -15543 | -11383 | DOWNSTREAM | CG7234-RI | "-"
 CG14011 in-situ | CG14011 | - | 6231 | 4695 | DOWNSTREAM | CG14011-RA | "-" | CG14011-RB | "-" | CG14011-RC | "-"


*********************** Rank 864 [Score  7.485400] GBROWSE*******************

 CG1921 in-situ | sty | - | -27686 | -51484 | UPSTREAM | CG1921-RC | "-" | CG1921-RB | "-"
 CG10840 in-situ | cIF2 | - | 7981 | -23967 | INTRAGENIC | intron:CG10840-RB:4 | CG10840-RB | "-"

*********************** Rank 865 [Score  7.485300] GBROWSE*******************

 CG8197 in-situ | CG8197 | - | -4079 | -5380 | UPSTREAM | CG8197-RA | "-"
 CG8084 in-situ | ana | + | 3098 | 11551 | UPSTREAM | CG8084-RA | "-"

*********************** Rank 866 [Score  7.484500] GBROWSE*******************

 CG11282 in-situ | caps | + | -9314 | 39757 | INTRAGENIC | intron:CG11282-RA:1 | intron:CG11282-RB:1 | CG11282-RA | "-" | CG11282-RB | "-"
 CG32119 in-situ | CG32119 | - | 10699 | 8860 | DOWNSTREAM | CG32119-RA | "-"

*********************** Rank 867 [Score  7.484400] GBROWSE*******************

insitu highlight CG5393 in-situ | apt | + | -18435 | 16368 | INTRAGENIC | intron:CG5393-RB:1 | intron:CG5393-RD:2 | intron:CG5393-RE:1 | intron:CG5393-RC:1 | intron:CG5393-RA:1 | CG5393-RB | "-" | CG5393-RD | "-" | CG5393-RE | "-" | CG5393-RC | "-" | CG5393-RA | "-"
 CG30182 in-situ | CG30182 | - | 17422 | 16191 | DOWNSTREAM | CG30182-RA | "-"

*********************** Rank 868 [Score  7.480300] GBROWSE*******************

 CG31006 in-situ | CG31006 | - | -159 | -11107 | UPSTREAM | CG31006-RB | "-" | CG31006-RA | "-"
 CG1480 in-situ | bnk | + | 23962 | 25533 | UPSTREAM | CG1480-RA | "-"

*********************** Rank 869 [Score  7.478100] GBROWSE*******************

 CG10823 in-situ | CG10823 | - | -17763 | -34926 | UPSTREAM | CG10823-RA | "-" | CG10823-RB | "-"
 CG31182 in-situ | CG31182 | - | 7103 | 5616 | DOWNSTREAM | CG31182-RA | "-"

*********************** Rank 870 [Score  7.470800] GBROWSE*******************

 CG31773 in-situ | CG31773 | - | -18643 | -21141 | UPSTREAM | CG31773-RA | "-"
 CG31774 in-situ | fred | - | 14229 | -27418 | INTRAGENIC | intron:CG31774-RA:2 | CG31774-RA | "-"

*********************** Rank 871 [Score  7.468300] GBROWSE*******************

 CG13114 in-situ | CG13114 | + | -3977 | -2724 | DOWNSTREAM | CG13114-RA | "-"
insitu CG4405 in-situ | jp | - | 16729 | -2304 | INTRAGENIC | intron:CG4405-RB:8 | intron:CG4405-RA:8 | CG4405-RB | "-" | CG4405-RA | "-"

*********************** Rank 872 [Score  7.466100] GBROWSE*******************

 CG6669 in-situ | klg | + | -12570 | 49862 | INTRAGENIC | intron:CG6669-RA:1 | CG6669-RA | "-"
 CG6660 in-situ | CG6660 | + | 11599 | 12479 | UPSTREAM | CG6660-RA | "-"

*********************** Rank 873 [Score  7.465700] GBROWSE*******************

 CG15455 in-situ | CG15455 | + | -18021 | -5019 | DOWNSTREAM | CG15455-RA | "-"
 CG15454 in-situ | CG15454 | + | 5869 | 7111 | UPSTREAM | CG15454-RA | "-"

*********************** Rank 874 [Score  7.462300] GBROWSE*******************

 CG32494 in-situ | CG32494 | + | -2850 | -1613 | DOWNSTREAM | CG32494-RA | "-"
 CG12672 in-situ | CG12672 | - | 4651 | 3056 | DOWNSTREAM | CG12672-RA | "-"

*********************** Rank 875 [Score  7.457600] GBROWSE*******************

insitu CG17932 in-situ | Ugt36Bc | + | -4837 | -2281 | DOWNSTREAM | CG17932-RA | "-" | CG17932-RB | "-"
 CG13272 in-situ | CG13272 | + | 7799 | 11927 | UPSTREAM | CG13272-RA | "-"

*********************** Rank 876 [Score  7.456100] GBROWSE*******************

 CG5488 in-situ | B-H2 | + | -10610 | -1029 | DOWNSTREAM | CG5488-RA | "-"
insitu CG5529 in-situ | B-H1 | + | 72310 | 78088 | UPSTREAM | CG5529-RA | "-"


*********************** Rank 877 [Score  7.453400] GBROWSE*******************

 CG5079 in-situ | CG5079 | + | -3958 | -3445 | DOWNSTREAM | CG5079-RA | "-"
 CG5071 in-situ | CG5071 | + | 651 | 2901 | UPSTREAM | CG5071-RB | "-" | CG5071-RA | "-"

*********************** Rank 878 [Score  7.453200] GBROWSE*******************

 CG5361 in-situ | CG5361 | - | -23994 | -25346 | UPSTREAM | CG5361-RA | "-"
 CG6203 in-situ | Fmr1 | - | 10762 | 2054 | DOWNSTREAM | CG6203-RB | "-" | CG6203-RA | "-" | CG6203-RC | "-" | CG6203-RD | "-" | CG6203-RE | "-"

*********************** Rank 879 [Score  7.452800] GBROWSE*******************

 CG16813 in-situ | CG16813 | + | -1427 | -873 | DOWNSTREAM | CG16813-RA | "-"
 CG16815 in-situ | CG16815 | + | 437 | 1204 | UPSTREAM | CG16815-RA | "-"

*********************** Rank 880 [Score  7.452600] GBROWSE*******************

 CG31745 in-situ | CG31745 | + | -2217 | -1284 | DOWNSTREAM | CG31745-RA | "-"
insitu CG15138 in-situ | beat-IIIc | - | 12965 | -57957 | INTRAGENIC | intron:CG15138-RA:2 | intron:CG15138-RB:2 | CG15138-RA | "-" | CG15138-RB | "-"


*********************** Rank 881 [Score  7.452000] GBROWSE*******************

 CG3655 in-situ | EG:103E12.3 | - | -20424 | -28965 | UPSTREAM | CG3655-RA | "-"
 CG14628 in-situ | EG:BACR42I17.12 | + | 42815 | 43240 | UPSTREAM | CG14628-RA | "-"


*********************** Rank 882 [Score  7.448700] GBROWSE*******************

 CG14846 in-situ | CG14846 | - | -6753 | -9013 | UPSTREAM | CG14846-RA | "-"
 CG14847 in-situ | CG14847 | - | 309 | -758 | INTRAGENIC | intron:CG14847-RA:1 | CG14847-RA | "-"

*********************** Rank 883 [Score  7.448200] GBROWSE*******************

 CG13482 in-situ | CG13482 | + | -20883 | -20575 | DOWNSTREAM | CG13482-RA | "-"
 CG13481 in-situ | CG13481 | - | 19323 | 18801 | DOWNSTREAM | CG13481-RA | "-"

*********************** Rank 884 [Score  7.445000] GBROWSE*******************

 CG13802 in-situ | CG13802 | + | -20150 | -19698 | DOWNSTREAM | CG13802-RA | "-"
 CG8985 in-situ | CG8985 | + | 18567 | 24130 | UPSTREAM | CG8985-RA | "-"

*********************** Rank 885 [Score  7.443900] GBROWSE*******************

insitu CG5799 in-situ | dve | + | -29136 | 13319 | INTRAGENIC | intron:CG5799-RA:2 | intron:CG5799-RD:2 | intron:CG5799-RC:3 | CG5799-RA | "-" | CG5799-RD | "-" | CG5799-RB | "-" | CG5799-RC | "-"
insitu CG5819 in-situ | CG5819 | + | 20360 | 23859 | UPSTREAM | CG5819-RA | "-" | CG5819-RB | "-"

*********************** Rank 886 [Score  7.443200] GBROWSE*******************

 CG10043 in-situ | rtGEF | + | -18472 | -3647 | DOWNSTREAM | CG10043-RA | "-" | CG10043-RB | "-"
 CG11012 in-situ | Ugt37a1 | - | 5059 | 3565 | DOWNSTREAM | CG11012-RA | "-"

*********************** Rank 887 [Score  7.442500] GBROWSE*******************

insitu highlight CG12287 in-situ | pdm2 | + | -3451 | 25033 | INTRAGENIC | intron:CG12287-RB:2 | CG12287-RB | "-" | CG12287-RA | "-"
 CG15485 in-situ | CG15485 | - | 7889 | 5985 | DOWNSTREAM | CG15485-RA | "-"

*********************** Rank 888 [Score  7.442400] GBROWSE*******************

insitu highlight CG12708 in-situ | CG12708 | + | -8835 | -6599 | DOWNSTREAM | CG12708-RA | "-"
 CG15599 in-situ | CG15599 | - | 3479 | 739 | DOWNSTREAM | CG15599-RA | "-"

*********************** Rank 889 [Score  7.441200] GBROWSE*******************

 CG3953 in-situ | l(3)IX-14 | + | -3261 | 10349 | INTRAGENIC | intron:CG3953-RA:1 | CG3953-RA | "-"
 CG6254 in-situ | CG6254 | - | 13368 | 11176 | DOWNSTREAM | CG6254-RA | "-"

*********************** Rank 890 [Score  7.438800] GBROWSE*******************

 CG5526 in-situ | Dhc36C | + | -4700 | 19350 | INTRAGENIC | intron:CG5526-RA:6 | CG5526-RA | "-"
 CG15143 in-situ | CG15143 | - | 18382 | 14722 | DOWNSTREAM | CG15143-RA | "-"

*********************** Rank 891 [Score  7.437400] GBROWSE*******************

 CG3528 in-situ | CG3528 | - | -8196 | -9037 | UPSTREAM | CG3528-RA | "-"
 CG3515 in-situ | CG3515 | - | 26611 | 24911 | DOWNSTREAM | CG3515-RA | "-"

*********************** Rank 892 [Score  7.436600] GBROWSE*******************

 CG9456 in-situ | Spn1 | + | -2845 | -975 | DOWNSTREAM | CG9456-RA | "-"
insitu CG9460 in-situ | CG9460 | + | 557 | 2418 | UPSTREAM | CG9460-RA | "-"

*********************** Rank 893 [Score  7.436300] GBROWSE*******************

insitu highlight CG14427 in-situ | CG14427 | + | -6366 | -5205 | DOWNSTREAM | CG14427-RA | "-"
 CG14426 in-situ | nullo | + | 2447 | 3410 | UPSTREAM | CG14426-RA | "-"

*********************** Rank 894 [Score  7.434700] GBROWSE*******************

insitu CG6305 in-situ | CG6305 | + | -1133 | 9300 | INTRAGENIC | intron:CG6305-RA:2 | CG6305-RA | "-"
 CG12295 in-situ | CG12295 | + | 9538 | 23639 | UPSTREAM | CG12295-RB | "-"


*********************** Rank 895 [Score  7.432000] GBROWSE*******************

 CG1086 in-situ | Glut1 | + | -26447 | 43919 | INTRAGENIC | intron:CG1086-RA:2 | CG1086-RA | "-" | CG1086-RB | "-" | CG1086-RC | "-"
 CG13908 in-situ | CG13908 | + | 46690 | 51943 | UPSTREAM | CG13908-RA | "-"

*********************** Rank 896 [Score  7.431900] GBROWSE*******************

 CG4328 in-situ | CG4328 | - | -13341 | -15267 | UPSTREAM | CG4328-RA | "-"
insitu highlight CG32105 in-situ | CG32105 | + | 22933 | 29603 | UPSTREAM | CG32105-RB | "-"

*********************** Rank 897 [Score  7.427700] GBROWSE*******************

 CG11219 in-situ | PIP82 | - | -11079 | -15087 | UPSTREAM | CG11219-RA | "-"
insitu CG12113 in-situ | CG12113 | - | 18157 | 14639 | DOWNSTREAM | CG12113-RA | "-"

*********************** Rank 898 [Score  7.426800] GBROWSE*******************

 CG2212 in-situ | sws | - | -5174 | -16385 | UPSTREAM | CG2212-RA | "-" | CG2212-RB | "-"
 CG1543 in-situ | Tbh | + | 22493 | 53330 | UPSTREAM | CG1543-RA | "-" | CG1543-RB | "-"

*********************** Rank 899 [Score  7.426300] GBROWSE*******************

 CG5210 in-situ | Chit | + | -11677 | -9620 | DOWNSTREAM | CG5210-RA | "-"
 CG30463 in-situ | CG30463 | - | 28009 | -29914 | INTRAGENIC | intron:CG30463-RA:3 | intron:CG30463-RB:2 | CG30463-RA | "-" | CG30463-RB | "-"

*********************** Rank 900 [Score  7.424900] GBROWSE*******************

 CG12700 in-situ | skpD | + | -2711 | -2052 | DOWNSTREAM | CG12700-RA | "-"
 CG11941 in-situ | skpC | + | 698 | 1174 | UPSTREAM | CG11941-RA | "-"

*********************** Rank 901 [Score  7.424100] GBROWSE*******************

 CG11145 in-situ | CG11145 | - | -1306 | -1995 | UPSTREAM | CG11145-RA | "-"
insitu CG11123 in-situ | CG11123 | + | 10794 | 12986 | UPSTREAM | CG11123-RA | "-"

*********************** Rank 902 [Score  7.421900] GBROWSE*******************

 CG14935 in-situ | CG14935 | + | -6417 | -3810 | DOWNSTREAM | CG14935-RA | "-" | CG14935-RB | "-"
 CG14943 in-situ | CG14943 | - | 2971 | 2447 | DOWNSTREAM | CG14943-RA | "-"

*********************** Rank 903 [Score  7.420400] GBROWSE*******************

 CG13800 in-situ | CG13800 | + | -14203 | -7250 | DOWNSTREAM | CG13800-RA | "-"
insitu highlight CG32306 in-situ | CG32306 | + | 12982 | 48528 | UPSTREAM | CG32306-RB | "-" | CG32306-RA | "-" | CG32306-RC | "-"

*********************** Rank 904 [Score  7.417400] GBROWSE*******************

 CG30188 in-situ | CG30188 | - | -2226 | -11020 | UPSTREAM | CG30188-RA | "-"
 CG13548 in-situ | CG13548 | - | 1233 | 715 | DOWNSTREAM | CG13548-RA | "-"

*********************** Rank 905 [Score  7.417000] GBROWSE*******************

 CG15876 in-situ | CG15876 | - | -1520 | -2010 | UPSTREAM | CG15876-RA | "-"
 CG13713 in-situ | CG13713 | + | 1420 | 1719 | UPSTREAM | CG13713-RA | "-"

*********************** Rank 906 [Score  7.415500] GBROWSE*******************

insitu CG32096 in-situ | rols | - | -194 | -56838 | UPSTREAM | CG32096-RB | "-" | CG32096-RD | "-" | CG32096-RE | "-" | CG32096-RA | "-" | CG32096-RC | "-"
insitu highlight CG5661 in-situ | Sema-5c | - | 16732 | 2458 | DOWNSTREAM | CG5661-RA | "-"

*********************** Rank 907 [Score  7.412800] GBROWSE*******************

 CG5123 in-situ | W | - | -6284 | -24179 | UPSTREAM | CG5123-RA | "-"
 CG7320 in-situ | CG7320 | + | 9918 | 11645 | UPSTREAM | CG7320-RA | "-"


*********************** Rank 908 [Score  7.412100] GBROWSE*******************

 CG12674 in-situ | CG12674 | + | -19958 | -18865 | DOWNSTREAM | CG12674-RA | "-"
 CG15381 in-situ | CG15381 | - | 703 | -5741 | INTRAGENIC | intron:CG15381-RA:1 | CG15381-RA | "-"

*********************** Rank 909 [Score  7.410800] GBROWSE*******************

insitu highlight CG2939 in-situ | slp2 | + | -15789 | -13430 | DOWNSTREAM | CG2939-RA | "-"
 CG3964 in-situ | CG3964 | + | 10050 | 14876 | UPSTREAM | CG3964-RB | "-" | CG3964-RA | "-"

*********************** Rank 910 [Score  7.409500] GBROWSE*******************

 CG1424 in-situ | mst | + | -2130 | -287 | DOWNSTREAM | CG1424-RA | "-"
 CG12794 in-situ | lcs | - | 6162 | 5590 | DOWNSTREAM | CG12794-RA | "-"

*********************** Rank 911 [Score  7.409500] GBROWSE*******************

 CG1895 in-situ | Cyp28c1 | + | -2982 | -1234 | DOWNSTREAM | CG1895-RA | "-"
 CG15740 in-situ | CG15740 | - | 3573 | 70 | DOWNSTREAM | CG15740-RA | "-"

*********************** Rank 912 [Score  7.409100] GBROWSE*******************

 CG17686 in-situ | DIP1 | - | -31682 | -37044 | UPSTREAM | CG17686-RA | "-" | CG17686-RB | "-" | CG17686-RC | "-" | CG17686-RD | "-"
 CG14621 in-situ | CG14621 | + | 43869 | 46296 | UPSTREAM | CG14621-RA | "-"

*********************** Rank 913 [Score  7.407100] GBROWSE*******************

 CG4114 in-situ | ex | + | -10582 | 6866 | INTRAGENIC | intron:CG4114-RA:3 | CG4114-RA | "-"
insitu highlight CG4280 in-situ | crq | - | 11188 | 6419 | DOWNSTREAM | CG4280-RA | "-" | CG4280-RB | "-"

*********************** Rank 914 [Score  7.406700] GBROWSE*******************

 CG3822 in-situ | CG3822 | - | -1882 | -7924 | UPSTREAM | CG3822-RA | "-"
 CG31509 in-situ | TotA | + | 2925 | 3594 | UPSTREAM | CG31509-RA | "-"

*********************** Rank 915 [Score  7.406700] GBROWSE*******************

 CG11414 in-situ | CG11414 | + | -2654 | 784 | INTRAGENIC | intron:CG11414-RA:4 | CG11414-RA | "-"
 CG11416 in-situ | CG11416 | + | 1277 | 3779 | UPSTREAM | CG11416-RA | "-"

*********************** Rank 916 [Score  7.406200] GBROWSE*******************

 CG4559 in-situ | Idgf3 | + | -30909 | -28640 | DOWNSTREAM | CG4559-RC | "-" | CG4559-RB | "-" | CG4559-RA | "-"
insitu CG4952 in-situ | dac | - | 4168 | -15306 | INTRAGENIC | intron:CG4952-RA:2 | intron:CG4952-RB:2 | intron:CG4952-RC:2 | intron:CG4952-RD:2 | intron:CG4952-RE:1 | CG4952-RA | "-" | CG4952-RB | "-" | CG4952-RC | "-" | CG4952-RD | "-" | CG4952-RE | "-"

*********************** Rank 917 [Score  7.403000] GBROWSE*******************

 CG31522 in-situ | CG31522 | - | -20415 | -32314 | UPSTREAM | CG31522-RB | "-" | CG31522-RC | "-" | CG31522-RD | "-" | CG31522-RA | "-"
 CG31523 in-situ | CG31523 | - | 5377 | -8312 | INTRAGENIC | intron:CG31523-RC:1 | intron:CG31523-RB:1 | CG31523-RC | "-" | CG31523-RB | "-" | CG31523-RA | "-" | CG31523-RD | "-"

*********************** Rank 918 [Score  7.402300] GBROWSE*******************

 CG17348 in-situ | drl | + | -14871 | 3398 | INTRAGENIC | intron:CG17348-RA:1 | CG17348-RA | "-"
 CG31797 in-situ | CG31797 | - | 56916 | 54310 | DOWNSTREAM | CG31797-RA | "-"


*********************** Rank 919 [Score  7.402200] GBROWSE*******************

 CG31738 in-situ | CG31738 | + | -13722 | 26260 | INTRAGENIC | intron:CG31738-RB:1 | CG31738-RB | "-" | CG31738-RA | "-"
 CG5996 in-situ | trpgamma | - | 40900 | 29095 | DOWNSTREAM | CG5996-RA | "-" | CG5996-RB | "-"

*********************** Rank 920 [Score  7.398900] GBROWSE*******************

 CG5156 in-situ | CG5156 | + | -4107 | -2613 | DOWNSTREAM | CG5156-RA | "-"
 CG5397 in-situ | CG5397 | + | 7954 | 10121 | UPSTREAM | CG5397-RA | "-"

*********************** Rank 921 [Score  7.398500] GBROWSE*******************

 CG31759 in-situ | CG31759 | + | -127402 | -124910 | DOWNSTREAM | CG31759-RB | "-" | CG31759-RA | "-"
 CG31862 in-situ | CG31862 | + | 3841 | 4389 | UPSTREAM | CG31862-RA | "-"

*********************** Rank 922 [Score  7.397200] GBROWSE*******************

 CG12159 in-situ | CG12159 | - | -136 | -1266 | UPSTREAM | CG12159-RA | "-"
 CG1877 in-situ | lin19 | + | 257 | 4378 | UPSTREAM | CG1877-RC | "-" | CG1877-RB | "-" | CG1877-RA | "-" | CG1877-RD | "-"

*********************** Rank 923 [Score  7.397000] GBROWSE*******************

insitu CG31558 in-situ | Obp83g | - | -233 | -860 | UPSTREAM | CG31558-RA | "-"
 CG1076 in-situ | CG1076 | + | 10129 | 10891 | UPSTREAM | CG1076-RA | "-"

*********************** Rank 924 [Score  7.395100] GBROWSE*******************

 CG31257 in-situ | CG31257 | + | -9498 | -7451 | DOWNSTREAM | CG31257-RA | "-"
 CG31418 in-situ | CG31418 | + | 20232 | 20952 | UPSTREAM | CG31418-RA | "-"

*********************** Rank 925 [Score  7.394200] GBROWSE*******************

 CG15150 in-situ | CG15150 | - | -16708 | -18007 | UPSTREAM | CG15150-RA | "-"
insitu CG15151 in-situ | PFE | + | 23544 | 42329 | UPSTREAM | CG15151-RA | "-"

*********************** Rank 926 [Score  7.393400] GBROWSE*******************

 CG33047 in-situ | CG33047 | - | -9605 | -13521 | UPSTREAM | CG33047-RA | "-" | CG33047-RB | "-" | CG33047-RC | "-"
 CG11711 in-situ | CG11711 | - | 29915 | -9561 | INTRAGENIC | intron:CG11711-RC:1 | intron:CG11711-RD:1 | intron:CG11711-RA:1 | intron:CG11711-RB:1 | CG11711-RC | "-" | CG11711-RD | "-" | CG11711-RA | "-" | CG11711-RB | "-"

*********************** Rank 927 [Score  7.392400] GBROWSE*******************

insitu CG2679 in-situ | gol | - | -19710 | -31410 | UPSTREAM | CG2679-RB | "-" | CG2679-RA | "-"
 CG30430 in-situ | CG30430 | + | 25402 | 25882 | UPSTREAM | CG30430-RA | "-"


*********************** Rank 928 [Score  7.392000] GBROWSE*******************

 CG10393 in-situ | amos | - | -917 | -2070 | UPSTREAM | CG10393-RA | "-"
insitu CG10413 in-situ | CG10413 | - | 6904 | 1632 | DOWNSTREAM | CG10413-RA | "-"

*********************** Rank 929 [Score  7.390800] GBROWSE*******************

 CG12496 in-situ | EG:BACH7M4.4 | - | -5729 | -7271 | UPSTREAM | CG12496-RA | "-"
 CG32797 in-situ | CG32797 | - | 9077 | 8469 | DOWNSTREAM | CG32797-RA | "-"

*********************** Rank 930 [Score  7.389700] GBROWSE*******************

insitu highlight CG32434 in-situ | CG32434 | + | -75185 | -34282 | DOWNSTREAM | CG32434-RB | "-"
 CG10573 in-situ | ko | - | 21435 | -34157 | INTRAGENIC | intron:CG10573-RA:1 | CG10573-RA | "-"

*********************** Rank 931 [Score  7.388700] GBROWSE*******************

 CG17834 in-situ | CG17834 | + | -426 | 11893 | INTRAGENIC | intron:CG17834-RA:1 | intron:CG17834-RC:1 | CG17834-RA | "-" | CG17834-RC | "-" | CG17834-RB | "-"
 CG18405 in-situ | Sema-1a | + | 34227 | 137860 | UPSTREAM | CG18405-RA | "-"

*********************** Rank 932 [Score  7.388400] GBROWSE*******************

 CG6414 in-situ | CG6414 | - | -41536 | -43886 | UPSTREAM | CG6414-RA | "-"
 CG32790 in-situ | CG32790 | + | 65032 | 66330 | UPSTREAM | CG32790-RA | "-"

*********************** Rank 933 [Score  7.386500] GBROWSE*******************

 CG12116 in-situ | CG12116 | - | -1747 | -3311 | UPSTREAM | CG12116-RA | "-"
 CG15348 in-situ | CG15348 | + | 632 | 994 | UPSTREAM | CG15348-RA | "-"

*********************** Rank 934 [Score  7.385900] GBROWSE*******************

insitu CG13606 in-situ | CG13606 | + | -6140 | 2648 | INTRAGENIC | intron:CG13606-RA:2 | CG13606-RA | "-"
 CG6198 in-situ | CHORD | + | 7594 | 9022 | UPSTREAM | CG6198-RA | "-"

*********************** Rank 935 [Score  7.385700] GBROWSE*******************

 CG1725 in-situ | dlg1 | + | -11124 | 26986 | INTRAGENIC | intron:CG1725-RC:3 | intron:CG1725-RF:2 | CG1725-RC | "-" | CG1725-RF | "-" | CG1725-RD | "-" | CG1725-RA | "-"
 CG15196 in-situ | CG15196 | + | 28668 | 30904 | UPSTREAM | CG15196-RA | "-"

*********************** Rank 936 [Score  7.380900] GBROWSE*******************

 CG30001 in-situ | CG30001 | - | -1448 | -2435 | UPSTREAM | CG30001-RA | "-"
 CG1625 in-situ | CG1625 | - | 2758 | -1290 | INTRAGENIC | intron:CG1625-RA:6 | CG1625-RA | "-"

*********************** Rank 937 [Score  7.379900] GBROWSE*******************

 CG1373 in-situ | CecC | + | -6978 | -6525 | DOWNSTREAM | CG1373-RA | "-"
 CG9737 in-situ | CG9737 | - | 19848 | 17801 | DOWNSTREAM | CG9737-RA | "-"

*********************** Rank 938 [Score  7.379500] GBROWSE*******************

insitu CG6705 in-situ | tsl | - | -27130 | -31550 | UPSTREAM | CG6705-RA | "-" | CG6705-RB | "-"
insitu CG6703 in-situ | Caki | - | 12978 | -23919 | INTRAGENIC | intron:CG6703-RB:8 | CG6703-RB | "-" | CG6703-RA | "-" | CG6703-RC | "-"

*********************** Rank 939 [Score  7.375700] GBROWSE*******************

 CG15405 in-situ | CG15405 | - | -54145 | -58494 | UPSTREAM | CG15405-RA | "-"
 CG3347 in-situ | CG3347 | + | 12771 | 24665 | UPSTREAM | CG3347-RA | "-"

*********************** Rank 940 [Score  7.375100] GBROWSE*******************

 CG15147 in-situ | CG15147 | - | -40822 | -41420 | UPSTREAM | CG15147-RA | "-"
 CG7100 in-situ | CadN | - | 72354 | -17301 | INTRAGENIC | intron:CG7100-RA:12 | intron:CG7100-RC:12 | intron:CG7100-RD:12 | intron:CG7100-RE:12 | intron:CG7100-RF:12 | intron:CG7100-RG:12 | intron:CG7100-RH:12 | intron:CG7100-RB:12 | CG7100-RA | "-" | CG7100-RC | "-" | CG7100-RD | "-" | CG7100-RE | "-" | CG7100-RF | "-" | CG7100-RG | "-" | CG7100-RH | "-" | CG7100-RB | "-"

*********************** Rank 941 [Score  7.372800] GBROWSE*******************

 CG9582 in-situ | CG9582 | - | -29515 | -30469 | UPSTREAM | CG9582-RA | "-"
 CG31708 in-situ | CG31708 | - | 16243 | -3295 | INTRAGENIC | intron:CG31708-RB:8 | intron:CG31708-RA:8 | CG31708-RB | "-" | CG31708-RA | "-"


*********************** Rank 942 [Score  7.372000] GBROWSE*******************

 CG5099 in-situ | msi | + | -62090 | 22008 | INTRAGENIC | intron:CG5099-RB:4 | CG5099-RB | "-" | CG5099-RA | "-"
 CG4582 in-situ | CG4582 | - | 2551 | 1054 | DOWNSTREAM | CG4582-RA | "-"

*********************** Rank 943 [Score  7.370500] GBROWSE*******************

 CG31049 in-situ | CG31049 | + | -15668 | -12496 | DOWNSTREAM | CG31049-RA | "-"
 CG33204 in-situ | CG33204 | + | 5325 | 10714 | UPSTREAM | CG33204-RA | "-"

*********************** Rank 944 [Score  7.369700] GBROWSE*******************

insitu CG6883 in-situ | trh | - | -7454 | -18247 | UPSTREAM | CG6883-RA | "-"
 CG13891 in-situ | CG13891 | - | 49120 | 48459 | DOWNSTREAM | CG13891-RA | "-"

*********************** Rank 945 [Score  7.369000] GBROWSE*******************

 CG31920 in-situ | CG31920 | - | -139 | -4380 | UPSTREAM | CG31920-RB | "-"
 CG31921 in-situ | CG31921 | - | 2435 | 91 | DOWNSTREAM | CG31921-RA | "-"


*********************** Rank 946 [Score  7.368800] GBROWSE*******************

 CG7686 in-situ | BcDNA:LD21529 | - | -21495 | -23215 | UPSTREAM | CG7686-RA | "-"
 CG30017 in-situ | CG30017 | - | 12148 | 10343 | DOWNSTREAM | CG30017-RA | "-"

*********************** Rank 947 [Score  7.368700] GBROWSE*******************

 CG14340 in-situ | CG14340 | + | -10096 | -9177 | DOWNSTREAM | CG14340-RA | "-"
insitu highlight CG4710 in-situ | CG4710 | - | 5869 | -8432 | INTRAGENIC | intron:CG4710-RB:2 | CG4710-RB | "-" | CG4710-RA | "-"

*********************** Rank 948 [Score  7.368000] GBROWSE*******************

insitu CG7097 in-situ | CG7097 | + | -32826 | 15620 | INTRAGENIC | intron:CG7097-RB:2 | intron:CG7097-RA:2 | CG7097-RB | "-" | CG7097-RA | "-"
 CG7137 in-situ | CG7137 | + | 15750 | 17037 | UPSTREAM | CG7137-RA | "-"


*********************** Rank 949 [Score  7.368000] GBROWSE*******************

 CG31257 in-situ | CG31257 | + | -11398 | -9351 | DOWNSTREAM | CG31257-RA | "-"
 CG31418 in-situ | CG31418 | + | 18332 | 19052 | UPSTREAM | CG31418-RA | "-"

*********************** Rank 950 [Score  7.365300] GBROWSE*******************

 CG4073 in-situ | CG4073 | + | -11312 | -10211 | DOWNSTREAM | CG4073-RA | "-"
 CG14690 in-situ | CG14690 | - | 9847 | 9236 | DOWNSTREAM | CG14690-RA | "-"


*********************** Rank 951 [Score  7.365200] GBROWSE*******************

 CG15599 in-situ | CG15599 | - | -1671 | -4411 | UPSTREAM | CG15599-RA | "-"
 CG8128 in-situ | CG8128 | + | 8019 | 10554 | UPSTREAM | CG8128-RA | "-"

*********************** Rank 952 [Score  7.363500] GBROWSE*******************

insitu CG6794 in-situ | Dif | - | -4222 | -19473 | UPSTREAM | CG6794-RA | "-" | CG6794-RB | "-"
 CG5043 in-situ | CG5043 | + | 1445 | 3237 | UPSTREAM | CG5043-RA | "-"

*********************** Rank 953 [Score  7.363200] GBROWSE*******************

 CG14551 in-situ | CG14551 | - | -22384 | -23301 | UPSTREAM | CG14551-RA | "-"
 CG32474 in-situ | cranky | - | 11932 | -7191 | INTRAGENIC | intron:CG32474-RA:3 | CG32474-RA | "-"

*********************** Rank 954 [Score  7.361600] GBROWSE*******************

 CG15545 in-situ | CG15545 | - | -382 | -1604 | UPSTREAM | CG15545-RA | "-"
 CG15546 in-situ | CG15546 | - | 3472 | 2240 | DOWNSTREAM | CG15546-RA | "-"


*********************** Rank 955 [Score  7.361300] GBROWSE*******************

insitu CG17508 in-situ | CG17508 | + | -14855 | -11890 | DOWNSTREAM | CG17508-RA | "-"
 CG11665 in-situ | CG11665 | - | 29304 | 18176 | DOWNSTREAM | CG11665-RA | "-"

*********************** Rank 956 [Score  7.360000] GBROWSE*******************

insitu highlight CG9015 in-situ | en | - | -23677 | -27883 | UPSTREAM | CG9015-RB | "-" | CG9015-RA | "-"
 CG10897 in-situ | tou | - | 63944 | 26995 | DOWNSTREAM | CG10897-RA | "-" | CG10897-RC | "-" | CG10897-RD | "-" | CG10897-RB | "-"

*********************** Rank 957 [Score  7.359000] GBROWSE*******************

 CG3748 in-situ | CG3748 | + | -3420 | -2092 | DOWNSTREAM | CG3748-RB | "-"
 CG13110 in-situ | CG13110 | + | 4062 | 4585 | UPSTREAM | CG13110-RA | "-"

*********************** Rank 958 [Score  7.357400] GBROWSE*******************

 CG3973 in-situ | CG3973 | + | -559 | 51474 | INTRAGENIC | intron:CG3973-RA:1 | CG3973-RA | "-"
 CG14443 in-situ | CG14443 | + | 34229 | 35545 | UPSTREAM | CG14443-RA | "-"

*********************** Rank 959 [Score  7.356600] GBROWSE*******************

 CG32822 in-situ | CG32822 | + | -63842 | -63180 | DOWNSTREAM | CG32822-RA | "-"
 CG14476 in-situ | BcDNA:GH04962 | - | 21240 | 16573 | DOWNSTREAM | CG14476-RB | "-" | CG14476-RC | "-" | CG14476-RA | "-" | CG14476-RE | "-" | CG14476-RD | "-"

*********************** Rank 960 [Score  7.356400] GBROWSE*******************

 CG16766 in-situ | CG16766 | + | -7328 | -1343 | DOWNSTREAM | CG16766-RA | "-"
 CG7431 in-situ | CG7431 | - | 10298 | 4519 | DOWNSTREAM | CG7431-RA | "-"

*********************** Rank 961 [Score  7.352900] GBROWSE*******************

insitu highlight CG16765 in-situ | l(3)10615 | + | -7420 | 7177 | INTRAGENIC | intron:CG16765-RA:2 | intron:CG16765-RD:2 | intron:CG16765-RB:1 | CG16765-RA | "-" | CG16765-RD | "-" | CG16765-RB | "-" | CG16765-RC | "-"
insitu highlight CG8144 in-situ | ps | + | 7240 | 22513 | UPSTREAM | CG8144-RA | "-" | CG8144-RB | "-" | CG8144-RD | "-" | CG8144-RC | "-"

*********************** Rank 962 [Score  7.351000] GBROWSE*******************

 CG30428 in-situ | CG30428 | + | 20273134 | 20274297 | UPSTREAM | CG30428-RA | "-"
 CG2981 in-situ | TpnC41C | - | 185234 | 181411 | DOWNSTREAM | CG2981-RA | "-"

*********************** Rank 963 [Score  7.350800] GBROWSE*******************

 CG16757 in-situ | Spn | - | -27044 | -73201 | UPSTREAM | CG16757-RA | "-"
 CG16973 in-situ | msn | - | 5219 | -25547 | INTRAGENIC | intron:CG16973-RA:2 | CG16973-RA | "-"


*********************** Rank 964 [Score  7.348600] GBROWSE*******************

 CG31459 in-situ | CG31459 | + | -6466 | -5266 | DOWNSTREAM | CG31459-RA | "-"
 CG4662 in-situ | CG4662 | + | 12 | 7092 | UPSTREAM | CG4662-RA | "-" | CG4662-RB | "-"

*********************** Rank 965 [Score  7.348200] GBROWSE*******************

 CG31146 in-situ | CG31146 | + | -47760 | -11691 | DOWNSTREAM | CG31146-RD | "-"
 CG2616 in-situ | CG2616 | + | 16404 | 18405 | UPSTREAM | CG2616-RA | "-"

*********************** Rank 966 [Score  7.346200] GBROWSE*******************

 CG14346 in-situ | CG14346 | + | -39232 | -38287 | DOWNSTREAM | CG14346-RA | "-" | CG14346-RB | "-"
 CG5481 in-situ | lea | - | 13115 | -26446 | INTRAGENIC | intron:CG5481-RA:1 | CG5481-RA | "-"

*********************** Rank 967 [Score  7.345700] GBROWSE*******************

insitu CG31666 in-situ | CG31666 | + | -5873 | 39494 | INTRAGENIC | intron:CG31666-RD:1 | CG31666-RD | "-" | CG31666-RA | "-" | CG31666-RB | "-" | CG31666-RC | "-"
 CG31934 in-situ | CG31934 | - | 12181 | 11497 | DOWNSTREAM | CG31934-RA | "-"

*********************** Rank 968 [Score  7.345100] GBROWSE*******************

 CG12680 in-situ | CG12680 | + | -7802 | -7350 | DOWNSTREAM | CG12680-RA | "-"
 CG6824 in-situ | ovo | + | 42761 | 64168 | UPSTREAM | CG6824-RB | "-" | CG6824-RC | "-" | CG6824-RA | "-"

*********************** Rank 969 [Score  7.343200] GBROWSE*******************

 CG31537 in-situ | CG31537 | - | -34243 | -36207 | UPSTREAM | CG31537-RA | "-"
 CG2534 in-situ | cno | - | 2494 | -41333 | INTRAGENIC | intron:CG2534-RA:1 | intron:CG2534-RB:1 | CG2534-RA | "-" | CG2534-RB | "-"

*********************** Rank 970 [Score  7.342200] GBROWSE*******************

 CG5144 in-situ | CG5144 | + | -17850 | -16557 | DOWNSTREAM | CG5144-RA | "-"
insitu CG32031 in-situ | Argk | - | 715 | -16428 | INTRAGENIC | intron:CG32031-RA:1 | CG32031-RA | "-" | CG32031-RB | "-" | CG32031-RD | "-" | CG32031-RC | "-"

*********************** Rank 971 [Score  7.340400] GBROWSE*******************

 CG12563 in-situ | CG12563 | - | -29434 | -29745 | UPSTREAM | CG12563-RA | "-"

*********************** Rank 972 [Score  7.337800] GBROWSE*******************

 CG11490 in-situ | CG11490 | + | -16051 | -12944 | DOWNSTREAM | CG11490-RA | "-"
 CG13693 in-situ | CG13693 | + | 9064 | 10515 | UPSTREAM | CG13693-RA | "-"

*********************** Rank 973 [Score  7.335000] GBROWSE*******************

 CG31397 in-situ | CG31397 | + | -23971 | -23303 | DOWNSTREAM | CG31397-RA | "-"
 CG31394 in-situ | CG31394 | - | 1446 | 708 | DOWNSTREAM | CG31394-RA | "-"

*********************** Rank 974 [Score  7.334300] GBROWSE*******************

 CG3289 in-situ | Ptpa | + | -17523 | -15945 | DOWNSTREAM | CG3289-RA | "-"
 CG3254 in-situ | CG3254 | - | 13039 | 9929 | DOWNSTREAM | CG3254-RA | "-"

*********************** Rank 975 [Score  7.333100] GBROWSE*******************

 CG2945 in-situ | cin | + | -1760 | 1758 | INTRAGENIC | intron:CG2945-RB:3 | intron:CG2945-RA:2 | CG2945-RB | "-" | CG2945-RA | "-"
insitu CG13377 in-situ | EG:BACR37P7.9 | - | 7283 | 1621 | DOWNSTREAM | CG13377-RA | "-"

*********************** Rank 976 [Score  7.332900] GBROWSE*******************

 CG14811 in-situ | CG14811 | - | -1178 | -1828 | UPSTREAM | CG14811-RA | "-"
 CG14799 in-situ | CG14799 | + | 2332 | 3349 | UPSTREAM | CG14799-RA | "-"


*********************** Rank 977 [Score  7.332900] GBROWSE*******************

 CG5065 in-situ | CG5065 | + | -6255 | 9437 | INTRAGENIC | intron:CG5065-RA:1 | CG5065-RA | "-"
 CG8250 in-situ | Alk | - | 23849 | 12160 | DOWNSTREAM | CG8250-RA | "-"

*********************** Rank 978 [Score  7.331800] GBROWSE*******************

 CG15095 in-situ | l(2)08717 | - | -6953 | -13416 | UPSTREAM | CG15095-RB | "-" | CG15095-RA | "-"
 CG15096 in-situ | CG15096 | - | 4329 | -2344 | INTRAGENIC | intron:CG15096-RA:1 | intron:CG15096-RB:1 | CG15096-RA | "-" | CG15096-RB | "-"

*********************** Rank 979 [Score  7.331400] GBROWSE*******************

 CG5481 in-situ | lea | - | -25385 | -64946 | UPSTREAM | CG5481-RA | "-"
 CG31925 in-situ | CG31925 | - | 28209 | 27516 | DOWNSTREAM | CG31925-RA | "-"

*********************** Rank 980 [Score  7.330700] GBROWSE*******************

 CG32175 in-situ | CG32175 | + | -5110 | -4460 | DOWNSTREAM | CG32175-RA | "-"
 CG6512 in-situ | CG6512 | - | 40303 | 36797 | DOWNSTREAM | CG6512-RA | "-" | CG6512-RB | "-"

*********************** Rank 981 [Score  7.329000] GBROWSE*******************

 CG4356 in-situ | mAcR-60C | - | -6520 | -17596 | UPSTREAM | CG4356-RA | "-" | CG4356-RB | "-"
 CG4527 in-situ | CG4527 | - | 6456 | -4103 | INTRAGENIC | intron:CG4527-RA:10 | intron:CG4527-RB:10 | CG4527-RA | "-" | CG4527-RB | "-"

*********************** Rank 982 [Score  7.327400] GBROWSE*******************

 CG9871 in-situ | CG9871 | - | -5445 | -6690 | UPSTREAM | CG9871-RA | "-"
 CG12782 in-situ | CG12782 | - | 12252 | 10987 | DOWNSTREAM | CG12782-RA | "-"

*********************** Rank 983 [Score  7.326800] GBROWSE*******************

 CG13235 in-situ | CG13235 | + | -4772 | -4605 | DOWNSTREAM | CG13235-RA | "-"
 CG9087 in-situ | CG9087 | - | 14180 | 9809 | DOWNSTREAM | CG9087-RA | "-"


*********************** Rank 984 [Score  7.326600] GBROWSE*******************

insitu highlight CG30092 in-situ | jbug | - | -7309 | -26829 | UPSTREAM | CG30092-RD | "-" | CG30092-RB | "-" | CG30092-RC | "-"
 CG13526 in-situ | CG13526 | + | 11090 | 11746 | UPSTREAM | CG13526-RA | "-"

*********************** Rank 985 [Score  7.325100] GBROWSE*******************

insitu highlight CG6889 in-situ | tara | + | -25761 | 8892 | INTRAGENIC | intron:CG6889-RA:1 | intron:CG6889-RB:1 | CG6889-RA | "-" | CG6889-RB | "-"
 CG6815 in-situ | bor | - | 12678 | 9940 | DOWNSTREAM | CG6815-RA | "-"

*********************** Rank 986 [Score  7.323500] GBROWSE*******************

insitu CG6899 in-situ | Ptp4E | + | -1254 | 15039 | INTRAGENIC | intron:CG6899-RA:1 | intron:CG6899-RB:1 | CG6899-RA | "-" | CG6899-RB | "-"
 CG15468 in-situ | CG15468 | - | 20614 | 19540 | DOWNSTREAM | CG15468-RA | "-"

*********************** Rank 987 [Score  7.322300] GBROWSE*******************

insitu highlight CG1133 in-situ | opa | + | -6284 | 10858 | INTRAGENIC | intron:CG1133-RA:1 | CG1133-RA | "-"
 CG14660 in-situ | CG14660 | - | 19404 | 16876 | DOWNSTREAM | CG14660-RA | "-"

*********************** Rank 988 [Score  7.320600] GBROWSE*******************

 CG10160 in-situ | ImpL3 | - | -30878 | -34082 | UPSTREAM | CG10160-RA | "-"
 CG32401 in-situ | CG32401 | + | 27040 | 28527 | UPSTREAM | CG32401-RA | "-"

*********************** Rank 989 [Score  7.320300] GBROWSE*******************

 CG4069 in-situ | CG4069 | - | -25243 | -27129 | UPSTREAM | CG4069-RA | "-"
 CG10632 in-situ | CG10632 | - | 9969 | -25044 | INTRAGENIC | intron:CG10632-RA:2 | CG10632-RA | "-" | CG10632-RB | "-"

*********************** Rank 990 [Score  7.320200] GBROWSE*******************

 CG32596 in-situ | CG32596 | - | -4897 | -5648 | UPSTREAM | CG32596-RA | "-"
 CG9411 in-situ | CG9411 | - | 40193 | 32517 | DOWNSTREAM | CG9411-RA | "-"

*********************** Rank 991 [Score  7.319000] GBROWSE*******************

 CG16944 in-situ | sesB | - | -21526 | -27485 | UPSTREAM | CG16944-RA | "-" | CG16944-RC | "-" | CG16944-RB | "-" | CG16944-RD | "-"
 CG1691 in-situ | Imp | - | 14471 | -12473 | INTRAGENIC | intron:CG1691-RF:2 | intron:CG1691-RH:3 | intron:CG1691-RG:2 | intron:CG1691-RD:2 | intron:CG1691-RE:2 | CG1691-RF | "-" | CG1691-RH | "-" | CG1691-RG | "-" | CG1691-RD | "-" | CG1691-RE | "-" | CG1691-RC | "-" | CG1691-RB | "-" | CG1691-RA | "-"

*********************** Rank 992 [Score  7.318500] GBROWSE*******************

 CG7856 in-situ | CG7856 | - | -974 | -3175 | UPSTREAM | CG7856-RA | "-"
 CG14593 in-situ | CG14593 | + | 2169 | 7182 | UPSTREAM | CG14593-RA | "-"

*********************** Rank 993 [Score  7.318200] GBROWSE*******************

insitu CG11387 in-situ | ct | + | -14125 | 52750 | INTRAGENIC | intron:CG11387-RA:1 | CG11387-RA | "-" | CG11387-RB | "-"
 CG12690 in-situ | CHES-1-like | - | 73421 | 61614 | DOWNSTREAM | CG12690-RA | "-"

*********************** Rank 994 [Score  7.317400] GBROWSE*******************

insitu CG1743 in-situ | Gs2 | + | -12156 | -4816 | DOWNSTREAM | CG1743-RB | "-" | CG1743-RC | "-"
insitu CG1747 in-situ | Sphk1 | + | 7530 | 11001 | UPSTREAM | CG1747-RB | "-" | CG1747-RA | "-"

*********************** Rank 995 [Score  7.315300] GBROWSE*******************

 CG6621 in-situ | CG6621 | - | -56380 | -60142 | UPSTREAM | CG6621-RA | "-"
 CG4683 in-situ | CG4683 | + | 409 | 1764 | UPSTREAM | CG4683-RA | "-"

*********************** Rank 996 [Score  7.314800] GBROWSE*******************

 CG15031 in-situ | CG15031 | - | -5023 | -6084 | UPSTREAM | CG15031-RA | "-"
 CG9106 in-situ | CG9106 | - | 22469 | 21100 | DOWNSTREAM | CG9106-RA | "-" | CG9106-RB | "-"

*********************** Rank 997 [Score  7.314100] GBROWSE*******************

insitu highlight CG9739 in-situ | fz2 | - | -33195 | -60923 | UPSTREAM | CG9739-RB | "-" | CG9739-RA | "-"
 CG9730 in-situ | mRpL21 | - | 51236 | 50413 | DOWNSTREAM | CG9730-RA | "-"

*********************** Rank 998 [Score  7.312300] GBROWSE*******************

 CG4568 in-situ | fzo | - | -1837 | -4235 | UPSTREAM | CG4568-RA | "-"
insitu highlight CG17894 in-situ | cnc | - | 16090 | -20293 | INTRAGENIC | intron:CG17894-RC:5 | CG17894-RC | "-" | CG17894-RB | "-" | CG17894-RG | "-" | CG17894-RF | "-" | CG17894-RE | "-" | CG17894-RD | "-" | CG17894-RA | "-"

*********************** Rank 999 [Score  7.311600] GBROWSE*******************

 CG14944 in-situ | CG14944 | - | -2367 | -2945 | UPSTREAM | CG14944-RA | "-"
insitu CG31757 in-situ | CG31757 | - | 29641 | -68803 | INTRAGENIC | intron:CG31757-RA:3 | CG31757-RA | "-"

*********************** Rank 1000 [Score  7.309700] GBROWSE*******************

 CG10211 in-situ | CG10211 | + | -22374 | -8085 | DOWNSTREAM | CG10211-RA | "-"
 CG15159 in-situ | CG15159 | - | 4248 | 3379 | DOWNSTREAM | CG15159-RA | "-"

*********************** Rank 1001 [Score  7.304700] GBROWSE*******************

 CG31488 in-situ | CG31488 | + | -8093 | -7464 | DOWNSTREAM | CG31488-RA | "-"
insitu highlight CG1028 in-situ | Antp | - | 34728 | -69574 | INTRAGENIC | intron:CG1028-RA:3 | intron:CG1028-RC:4 | CG1028-RA | "-" | CG1028-RC | "-" | CG1028-RB | "-"

*********************** Rank 1002 [Score  7.304500] GBROWSE*******************

 CG1559 in-situ | Upf1 | - | -9252 | -14155 | UPSTREAM | CG1559-RA | "-"
insitu CG2467 in-situ | CG2467 | - | 2641 | -6689 | INTRAGENIC | intron:CG2467-RA:1 | intron:CG2467-RB:1 | CG2467-RA | "-" | CG2467-RB | "-"

*********************** Rank 1003 [Score  7.304500] GBROWSE*******************

 CG12290 in-situ | CG12290 | + | -23261 | -20165 | DOWNSTREAM | CG12290-RA | "-"
 CG6058 in-situ | Ald | - | 8078 | 1169 | DOWNSTREAM | CG6058-RB | "-" | CG6058-RC | "-" | CG6058-RA | "-" | CG6058-RF | "-" | CG6058-RG | "-" | CG6058-RD | "-" | CG6058-RE | "-"

*********************** Rank 1004 [Score  7.304300] GBROWSE*******************

 CG31637 in-situ | CG31637 | + | -43942 | -15588 | DOWNSTREAM | CG31637-RA | "-"
insitu highlight CG9554 in-situ | eya | - | 4388 | -15135 | INTRAGENIC | intron:CG9554-RB:1 | CG9554-RB | "-" | CG9554-RA | "-"

*********************** Rank 1005 [Score  7.303600] GBROWSE*******************

 CG32710 in-situ | CG32710 | + | -35479 | -34028 | DOWNSTREAM | CG32710-RA | "-"
insitu CG12075 in-situ | CG12075 | + | 9412 | 17434 | UPSTREAM | CG12075-RA | "-"

*********************** Rank 1006 [Score  7.302800] GBROWSE*******************

insitu CG18657 in-situ | NetA | - | -14504 | -64333 | UPSTREAM | CG18657-RA | "-"
 CG5321 in-situ | CG5321 | + | 11269 | 13494 | UPSTREAM | CG5321-RA | "-"

*********************** Rank 1007 [Score  7.301200] GBROWSE*******************

 CG31601 in-situ | CG31601 | - | -2290 | -4534 | UPSTREAM | CG31601-RA | "-"
 CG31700 in-situ | CG31700 | + | 21193 | 24028 | UPSTREAM | CG31700-RA | "-"

*********************** Rank 1008 [Score  7.300800] GBROWSE*******************

 CG12667 in-situ | CG12667 | - | -702 | -1773 | UPSTREAM | CG12667-RA | "-"
 CG12135 in-situ | c12.1 | - | 5628 | 4298 | DOWNSTREAM | CG12135-RA | "-"


*********************** Rank 1009 [Score  7.299800] GBROWSE*******************

 CG15485 in-situ | CG15485 | - | -13911 | -15815 | UPSTREAM | CG15485-RA | "-"
insitu CG5525 in-situ | CG5525 | - | 9857 | 7245 | DOWNSTREAM | CG5525-RA | "-"

*********************** Rank 1010 [Score  7.299600] GBROWSE*******************

 CG31275 in-situ | CG31275 | - | -21821 | -22478 | UPSTREAM | CG31275-RB | "-" | CG31275-RA | "-"
 CG3853 in-situ | Glut3 | + | 15101 | 16813 | UPSTREAM | CG3853-RA | "-"

note: overlaps known module ubx_PBX_1991 by 470 bases (module coords: 12598980-12600361)
note: overlaps known module ubx_PBX by 500 bases (module coords: 12598636-12600185)

*********************** Rank 1011 [Score  7.299100] GBROWSE*******************

 CG4021 in-situ | CG4021 | + | -6454 | -4974 | DOWNSTREAM | CG4021-RA | "-"
 CG4402 in-situ | lox2 | - | 11856 | 9817 | DOWNSTREAM | CG4402-RA | "-"

*********************** Rank 1012 [Score  7.297000] GBROWSE*******************

insitu CG2297 in-situ | Obp44a | - | -9862 | -10588 | UPSTREAM | CG2297-RA | "-"
insitu CG8709 in-situ | CG8709 | - | 1024 | -6965 | INTRAGENIC | intron:CG8709-RA:1 | CG8709-RA | "-"

*********************** Rank 1013 [Score  7.296900] GBROWSE*******************

 CG8497 in-situ | Rhp | + | -6543 | -1811 | DOWNSTREAM | CG8497-RA | "-"
 CG8974 in-situ | CG8974 | - | 540 | -1717 | INTRAGENIC | intron:CG8974-RC:2 | intron:CG8974-RA:1 | intron:CG8974-RB:1 | CG8974-RC | "-" | CG8974-RA | "-" | CG8974-RB | "-"

*********************** Rank 1014 [Score  7.290900] GBROWSE*******************

 CG18266 in-situ | CG18266 | + | -4302 | -2499 | DOWNSTREAM | CG18266-RA | "-"
 CG14010 in-situ | CG14010 | - | 11888 | 1695 | DOWNSTREAM | CG14010-RA | "-"

*********************** Rank 1015 [Score  7.284800] GBROWSE*******************

insitu CG16707 in-situ | BEST:GH15083 | + | -1251 | 2145 | INTRAGENIC | intron:CG16707-RA:1 | intron:CG16707-RD:1 | intron:CG16707-RB:2 | intron:CG16707-RC:2 | CG16707-RA | "-" | CG16707-RD | "-" | CG16707-RB | "-" | CG16707-RC | "-"
 CG18178 in-situ | CG18178 | - | 3570 | 2215 | DOWNSTREAM | CG18178-RA | "-"

*********************** Rank 1016 [Score  7.284700] GBROWSE*******************

 CG30143 in-situ | CG30143 | + | -30097 | -24722 | DOWNSTREAM | CG30143-RA | "-"
 CG13421 in-situ | Obp57c | + | 1918 | 2568 | UPSTREAM | CG13421-RA | "-"

*********************** Rank 1017 [Score  7.284500] GBROWSE*******************

 CG12408 in-situ | CG12408 | - | -60613 | -65043 | UPSTREAM | CG12408-RA | "-"
 CG17510 in-situ | CG17510 | - | 36349 | 35107 | DOWNSTREAM | CG17510-RA | "-" | CG17510-RB | "-"

*********************** Rank 1018 [Score  7.283400] GBROWSE*******************

 CG14915 in-situ | CG14915 | + | -2580 | -2218 | DOWNSTREAM | CG14915-RA | "-"
insitu CG14919 in-situ | Ast2 | - | 7756 | 2511 | DOWNSTREAM | CG14919-RA | "-"

*********************** Rank 1019 [Score  7.281000] GBROWSE*******************

 CG18369 in-situ | CG18369 | + | -3959 | -1886 | DOWNSTREAM | CG18369-RA | "-"
 CG12464 in-situ | CG12464 | - | 8542 | 8029 | DOWNSTREAM | CG12464-RA | "-"


*********************** Rank 1020 [Score  7.279800] GBROWSE*******************

 CG13758 in-situ | EG:BACR25B3.3 | + | -17977 | 4375 | INTRAGENIC | intron:CG13758-RA:1 | CG13758-RA | "-"
 CG8310 in-situ | EG:BACR25B3.4 | - | 8136 | 7205 | DOWNSTREAM | CG8310-RA | "-"

*********************** Rank 1021 [Score  7.278000] GBROWSE*******************

 CG4070 in-situ | Tis11 | + | -2728 | 17563 | INTRAGENIC | intron:CG4070-RA:2 | intron:CG4070-RB:2 | CG4070-RA | "-" | CG4070-RB | "-"
 CG15725 in-situ | CG15725 | + | 44058 | 46430 | UPSTREAM | CG15725-RA | "-"

*********************** Rank 1022 [Score  7.277800] GBROWSE*******************

 CG9266 in-situ | CG9266 | - | -11415 | -14839 | UPSTREAM | CG9266-RB | "-"
insitu CG1762 in-situ | betaInt-nu | + | 72476 | 77688 | UPSTREAM | CG1762-RA | "-"

*********************** Rank 1023 [Score  7.277700] GBROWSE*******************

 CG32350 in-situ | CG32350 | - | -80483 | -83319 | UPSTREAM | CG32350-RA | "-"
 CG32230 in-situ | CG32230 | - | 29882 | 27601 | DOWNSTREAM | CG32230-RA | "-" | CG32230-RB | "-"

*********************** Rank 1024 [Score  7.276600] GBROWSE*******************

 CG3528 in-situ | CG3528 | - | -16496 | -17337 | UPSTREAM | CG3528-RA | "-"
 CG3515 in-situ | CG3515 | - | 18311 | 16611 | DOWNSTREAM | CG3515-RA | "-"

*********************** Rank 1025 [Score  7.275900] GBROWSE*******************

 CG14011 in-situ | CG14011 | - | -369 | -1905 | UPSTREAM | CG14011-RA | "-" | CG14011-RB | "-" | CG14011-RC | "-"
 CG7251 in-situ | CG7251 | - | 6929 | 5658 | DOWNSTREAM | CG7251-RA | "-"


*********************** Rank 1026 [Score  7.274100] GBROWSE*******************

insitu CG6024 in-situ | CG6024 | - | -1354 | -33563 | UPSTREAM | CG6024-RA | "-"
 CG6004 in-situ | CG6004 | - | 4929 | 149 | DOWNSTREAM | CG6004-RB | "-"

*********************** Rank 1027 [Score  7.273700] GBROWSE*******************

 CG10633 in-situ | CG10633 | - | -2471 | -6506 | UPSTREAM | CG10633-RA | "-"
 CG4814 in-situ | CG4814 | + | 47715 | 48985 | UPSTREAM | CG4814-RA | "-"

*********************** Rank 1028 [Score  7.273400] GBROWSE*******************

 CG31858 in-situ | CG31858 | - | -9834 | -10484 | UPSTREAM | CG31858-RA | "-"
 CG31857 in-situ | CG31857 | - | 9971 | 9309 | DOWNSTREAM | CG31857-RA | "-"

*********************** Rank 1029 [Score  7.269300] GBROWSE*******************

 CG17348 in-situ | drl | + | -19571 | -1302 | DOWNSTREAM | CG17348-RA | "-"
 CG31797 in-situ | CG31797 | - | 52216 | 49610 | DOWNSTREAM | CG31797-RA | "-"

*********************** Rank 1030 [Score  7.268500] GBROWSE*******************

 CG10633 in-situ | CG10633 | - | -48621 | -52656 | UPSTREAM | CG10633-RA | "-"
 CG4814 in-situ | CG4814 | + | 1565 | 2835 | UPSTREAM | CG4814-RA | "-"

*********************** Rank 1031 [Score  7.265800] GBROWSE*******************

 CG13438 in-situ | CG13438 | + | -29068 | -28329 | DOWNSTREAM | CG13438-RA | "-"
 CG13442 in-situ | CG13442 | - | 8672 | 5886 | DOWNSTREAM | CG13442-RA | "-"

*********************** Rank 1032 [Score  7.263900] GBROWSE*******************

 CG2861 in-situ | CG2861 | - | -7617 | -14026 | UPSTREAM | CG2861-RA | "-" | CG2861-RB | "-"
 CG12682 in-situ | CG12682 | + | 14617 | 15330 | UPSTREAM | CG12682-RA | "-"

*********************** Rank 1033 [Score  7.262700] GBROWSE*******************

 CG14441 in-situ | CG14441 | + | -10104 | -2845 | DOWNSTREAM | CG14441-RA | "-"
insitu CG3168 in-situ | CG3168 | - | 20162 | 1728 | DOWNSTREAM | CG3168-RC | "-" | CG3168-RA | "-" | CG3168-RB | "-"

*********************** Rank 1034 [Score  7.262100] GBROWSE*******************

 CG5361 in-situ | CG5361 | - | -22844 | -24196 | UPSTREAM | CG5361-RA | "-"
 CG6203 in-situ | Fmr1 | - | 11912 | 3204 | DOWNSTREAM | CG6203-RB | "-" | CG6203-RA | "-" | CG6203-RC | "-" | CG6203-RD | "-" | CG6203-RE | "-"

*********************** Rank 1035 [Score  7.261700] GBROWSE*******************

 CG17843 in-situ | CG17843 | + | -8665 | -6866 | DOWNSTREAM | CG17843-RA | "-"
 CG31171 in-situ | CG31171 | - | 10406 | 9751 | DOWNSTREAM | CG31171-RA | "-"

*********************** Rank 1036 [Score  7.261700] GBROWSE*******************

 CG10366 in-situ | CG10366 | + | -18708 | -16527 | DOWNSTREAM | CG10366-RA | "-"
 CG10443 in-situ | Lar | + | 8443 | 128595 | UPSTREAM | CG10443-RA | "-"


*********************** Rank 1037 [Score  7.260600] GBROWSE*******************

 CG12673 in-situ | olf413 | + | -68607 | -9565 | DOWNSTREAM | CG12673-RA | "-"
 CG9063 in-situ | BcDNA:GH03694 | - | 10523 | 5375 | DOWNSTREAM | CG9063-RA | "-"

*********************** Rank 1038 [Score  7.260200] GBROWSE*******************

 CG3977 in-situ | CG3977 | + | -9377 | -3973 | DOWNSTREAM | CG3977-RA | "-"
 CG3198 in-situ | CG3198 | - | 2945 | 173 | DOWNSTREAM | CG3198-RA | "-"

*********************** Rank 1039 [Score  7.260000] GBROWSE*******************

 CG3931 in-situ | CG3931 | - | -431 | -1614 | UPSTREAM | CG3931-RA | "-"
 CG5372 in-situ | alphaPS5 | + | 178 | 3565 | UPSTREAM | CG5372-RA | "-"

*********************** Rank 1040 [Score  7.259500] GBROWSE*******************

insitu highlight CG10325 in-situ | abd-A | - | -5862 | -28288 | UPSTREAM | CG10325-RA | "-" | CG10325-RB | "-"
 CG10349 in-situ | CG10349 | + | 43815 | 49197 | UPSTREAM | CG10349-RA | "-" | CG10349-RB | "-"

*********************** Rank 1041 [Score  7.256700] GBROWSE*******************

 CG6633 in-situ | Ugt86Dd | - | -520 | -2260 | UPSTREAM | CG6633-RA | "-"
 CG4706 in-situ | CG4706 | + | 8217 | 10790 | UPSTREAM | CG4706-RA | "-"

*********************** Rank 1042 [Score  7.256200] GBROWSE*******************

 CG18405 in-situ | Sema-1a | + | -78873 | 24760 | INTRAGENIC | intron:CG18405-RA:3 | CG18405-RA | "-"
 CG9280 in-situ | Glt | + | 29374 | 34142 | UPSTREAM | CG9280-RC | "-" | CG9280-RB | "-" | CG9280-RA | "-"

*********************** Rank 1043 [Score  7.251500] GBROWSE*******************

 CG1130 in-situ | scrt | + | -13105 | -8432 | DOWNSTREAM | CG1130-RA | "-"
 CG14985 in-situ | CG14985 | + | 6451 | 9605 | UPSTREAM | CG14985-RA | "-"

*********************** Rank 1044 [Score  7.251200] GBROWSE*******************

insitu CG15828 in-situ | CG15828 | + | -52571 | -37892 | DOWNSTREAM | CG15828-RA | "-"
 CG4379 in-situ | Pka-C1 | - | 5372 | -9265 | INTRAGENIC | intron:CG4379-RB:2 | CG4379-RA | "-" | CG4379-RB | "-"

*********************** Rank 1045 [Score  7.250500] GBROWSE*******************

insitu CG11453 in-situ | CG11453 | + | -5588 | -3581 | DOWNSTREAM | CG11453-RA | "-"
insitu CG4608 in-situ | bnl | - | 47706 | 4962 | DOWNSTREAM | CG4608-RA | "-" | CG4608-RB | "-"

*********************** Rank 1046 [Score  7.250300] GBROWSE*******************

 CG5583 in-situ | Ets98B | + | -8323 | 2212 | INTRAGENIC | intron:CG5583-RA:3 | CG5583-RA | "-"
 CG31308 in-situ | CG31308 | - | 22570 | 21863 | DOWNSTREAM | CG31308-RA | "-"

*********************** Rank 1047 [Score  7.249500] GBROWSE*******************

 CG15061 in-situ | CG15061 | + | -7762 | -6763 | DOWNSTREAM | CG15061-RA | "-"
 CG5993 in-situ | os | - | 7484 | 5003 | DOWNSTREAM | CG5993-RA | "-"


*********************** Rank 1048 [Score  7.249100] GBROWSE*******************

 CG4279 in-situ | CG4279 | - | -111 | -641 | UPSTREAM | CG4279-RA | "-"
 CG9433 in-situ | Xpd | + | 297 | 3798 | UPSTREAM | CG9433-RA | "-" | CG9433-RB | "-"


*********************** Rank 1049 [Score  7.245100] GBROWSE*******************

 CG5020 in-situ | CLIP-190 | + | -5127 | 19832 | INTRAGENIC | intron:CG5020-RB:3 | intron:CG5020-RA:2 | intron:CG5020-RD:1 | intron:CG5020-RC:1 | CG5020-RB | "-" | CG5020-RA | "-" | CG5020-RD | "-" | CG5020-RC | "-"
 CG6840 in-situ | Rpb11 | - | 20501 | 19829 | DOWNSTREAM | CG6840-RA | "-"

*********************** Rank 1050 [Score  7.243700] GBROWSE*******************

 CG14277 in-situ | CG14277 | + | -7302 | -6677 | DOWNSTREAM | CG14277-RA | "-"
 CG8086 in-situ | CG8086 | - | 7533 | -5424 | INTRAGENIC | intron:CG8086-RA:3 | intron:CG8086-RB:3 | CG8086-RA | "-" | CG8086-RB | "-"

*********************** Rank 1051 [Score  7.242300] GBROWSE*******************

 CG9834 in-situ | endoB | - | -8388 | -11640 | UPSTREAM | CG9834-RA | "-" | CG9834-RB | "-"
insitu CG9811 in-situ | CG9811 | - | 6409 | -6938 | INTRAGENIC | intron:CG9811-RA:2 | CG9811-RA | "-"

*********************** Rank 1052 [Score  7.241800] GBROWSE*******************

insitu CG17752 in-situ | CG17752 | + | -2686 | -474 | DOWNSTREAM | CG17752-RA | "-"
 CG16727 in-situ | CG16727 | + | 470 | 2617 | UPSTREAM | CG16727-RA | "-"

*********************** Rank 1053 [Score  7.241000] GBROWSE*******************

 CG31630 in-situ | CG31630 | + | -2452 | 2348 | INTRAGENIC | intron:CG31630-RA:1 | CG31630-RA | "-"
 CG13778 in-situ | Mnn1 | + | 11387 | 16802 | UPSTREAM | CG13778-RA | "-" | CG13778-RB | "-"

*********************** Rank 1054 [Score  7.239800] GBROWSE*******************

insitu highlight CG16738 in-situ | slp1 | + | -8001 | -6544 | DOWNSTREAM | CG16738-RA | "-"
insitu highlight CG2939 in-situ | slp2 | + | 3161 | 5520 | UPSTREAM | CG2939-RA | "-"

*********************** Rank 1055 [Score  7.239100] GBROWSE*******************

 CG4060 in-situ | CG4060 | - | -5965 | -7165 | UPSTREAM | CG4060-RA | "-"
 CG11896 in-situ | CG11896 | - | 1044 | -2911 | INTRAGENIC | intron:CG11896-RA:1 | CG11896-RA | "-"

*********************** Rank 1056 [Score  7.238800] GBROWSE*******************

 CG1976 in-situ | RhoGAP100F | + | -36185 | -23803 | DOWNSTREAM | CG1976-RA | "-"
insitu CG2003 in-situ | CG2003 | + | 109068 | 121854 | UPSTREAM | CG2003-RA | "-" | CG2003-RB | "-"

*********************** Rank 1057 [Score  7.237900] GBROWSE*******************

 CG32655 in-situ | CG32655 | - | -69011 | -70069 | UPSTREAM | CG32655-RA | "-"
 CG2577 in-situ | CG2577 | + | 68471 | 69711 | UPSTREAM | CG2577-RA | "-"

*********************** Rank 1058 [Score  7.236600] GBROWSE*******************

 CG13131 in-situ | CG13131 | - | -528 | -4720 | UPSTREAM | CG13131-RA | "-"
 CG13127 in-situ | CG13127 | + | 8749 | 11308 | UPSTREAM | CG13127-RA | "-"

*********************** Rank 1059 [Score  7.236300] GBROWSE*******************

insitu CG17065 in-situ | CG17065 | - | -4422 | -6862 | UPSTREAM | CG17065-RA | "-"
 CG17063 in-situ | inx6 | - | 7947 | 6428 | DOWNSTREAM | CG17063-RA | "-"

*********************** Rank 1060 [Score  7.234400] GBROWSE*******************

 CG11755 in-situ | CG11755 | + | -6028 | -5564 | DOWNSTREAM | CG11755-RA | "-"
insitu highlight CG9786 in-situ | hb | - | 13505 | 7003 | DOWNSTREAM | CG9786-RB | "-" | CG9786-RA | "-"

*********************** Rank 1061 [Score  7.234300] GBROWSE*******************

insitu CG6417 in-situ | CG6417 | + | -643 | 2924 | INTRAGENIC | intron:CG6417-RA:2 | CG6417-RA | "-"
 CG5418 in-situ | CG5418 | - | 4101 | 2932 | DOWNSTREAM | CG5418-RA | "-"

*********************** Rank 1062 [Score  7.234100] GBROWSE*******************

 CG5685 in-situ | Calx | + | -12835 | 23349 | INTRAGENIC | intron:CG5685-RA:1 | intron:CG5685-RB:2 | intron:CG5685-RC:1 | CG5685-RA | "-" | CG5685-RB | "-" | CG5685-RC | "-"
 CG10827 in-situ | CG10827 | - | 15876 | 14048 | DOWNSTREAM | CG10827-RA | "-"

*********************** Rank 1063 [Score  7.233200] GBROWSE*******************

 CG8427 in-situ | SmD3 | + | -2611 | -1328 | DOWNSTREAM | CG8427-RA | "-"
 CG16747 in-situ | guf | - | 2925 | -4765 | INTRAGENIC | intron:CG16747-RA:1 | CG16747-RA | "-" | CG16747-RC | "-" | CG16747-RB | "-"


*********************** Rank 1064 [Score  7.232700] GBROWSE*******************

insitu CG31116 in-situ | CG31116 | - | -3699 | -13016 | UPSTREAM | CG31116-RD | "-" | CG31116-RA | "-" | CG31116-RC | "-"
 CG14724 in-situ | CoVa | + | 9099 | 10048 | UPSTREAM | CG14724-RA | "-" | CG14724-RB | "-"

*********************** Rank 1065 [Score  7.232400] GBROWSE*******************

insitu highlight CG14080 in-situ | Mkp3 | - | -3499 | -22189 | UPSTREAM | CG14080-RB | "-" | CG14080-RA | "-"
 CG6818 in-situ | MESR6 | + | 2751 | 4839 | UPSTREAM | CG6818-RA | "-"

*********************** Rank 1066 [Score  7.231900] GBROWSE*******************

 CG2331 in-situ | TER94 | + | -8356 | -3950 | DOWNSTREAM | CG2331-RA | "-" | CG2331-RB | "-"
 CG15862 in-situ | Pka-R2 | - | 28518 | -1713 | INTRAGENIC | intron:CG15862-RA:9 | intron:CG15862-RB:9 | intron:CG15862-RC:8 | CG15862-RA | "-" | CG15862-RB | "-" | CG15862-RC | "-"

*********************** Rank 1067 [Score  7.228900] GBROWSE*******************

 CG13896 in-situ | CG13896 | - | -1547 | -2179 | UPSTREAM | CG13896-RA | "-"
 CG13897 in-situ | CG13897 | - | 25999 | 24556 | DOWNSTREAM | CG13897-RA | "-"


*********************** Rank 1068 [Score  7.227200] GBROWSE*******************

 CG3301 in-situ | CG3301 | - | -3610 | -5618 | UPSTREAM | CG3301-RA | "-" | CG3301-RB | "-"
 CG17298 in-situ | CG17298 | + | 3336 | 4194 | UPSTREAM | CG17298-RA | "-"

*********************** Rank 1069 [Score  7.226600] GBROWSE*******************

 CG17181 in-situ | CG17181 | - | -3399 | -8687 | UPSTREAM | CG17181-RA | "-"
 CG12031 in-situ | Trap170 | + | 575 | 6571 | UPSTREAM | CG12031-RA | "-" | CG12031-RB | "-"


*********************** Rank 1070 [Score  7.225500] GBROWSE*******************

 CG32390 in-situ | CG32390 | - | -6109 | -6695 | UPSTREAM | CG32390-RA | "-"
 CG32386 in-situ | corn | - | 6602 | -5947 | INTRAGENIC | intron:CG32386-RA:1 | CG32386-RA | "-"

*********************** Rank 1071 [Score  7.225100] GBROWSE*******************

 CG14581 in-situ | CG14581 | + | -7499 | -7203 | DOWNSTREAM | CG14581-RA | "-"
 CG32521 in-situ | CG32521 | - | 18384 | -57477 | INTRAGENIC | intron:CG32521-RA:1 | intron:CG32521-RB:1 | intron:CG32521-RC:1 | CG32521-RA | "-" | CG32521-RB | "-" | CG32521-RC | "-"

*********************** Rank 1072 [Score  7.223700] GBROWSE*******************

 CG6527 in-situ | CG6527 | - | -33272 | -34156 | UPSTREAM | CG6527-RA | "-"
 CG14151 in-situ | CG14151 | - | 36163 | 35359 | DOWNSTREAM | CG14151-RA | "-"

*********************** Rank 1073 [Score  7.222800] GBROWSE*******************

 CG12636 in-situ | BG:DS07721.6 | - | -6749 | -10789 | UPSTREAM | CG12636-RA | "-"
 CG31835 in-situ | CG31835 | + | 356 | 1405 | UPSTREAM | CG31835-RA | "-"

*********************** Rank 1074 [Score  7.222500] GBROWSE*******************

insitu CG6822 in-situ | rhea | + | -19038 | -15972 | DOWNSTREAM | CG6822-RA | "-" | CG6822-RB | "-"
 CG6831 in-situ | Talin | + | 7267 | 20410 | UPSTREAM | CG6831-RA | "-"

*********************** Rank 1075 [Score  7.220000] GBROWSE*******************

 CG9113 in-situ | AP-1gamma | + | -19223 | -11400 | DOWNSTREAM | CG9113-RB | "-" | CG9113-RD | "-" | CG9113-RA | "-" | CG9113-RC | "-"
 CG12664 in-situ | ld14 | - | 7102 | -2784 | INTRAGENIC | intron:CG12664-RB:1 | CG12664-RB | "-"

*********************** Rank 1076 [Score  7.219000] GBROWSE*******************

 CG6414 in-situ | CG6414 | - | -90086 | -92436 | UPSTREAM | CG6414-RA | "-"
 CG32790 in-situ | CG32790 | + | 16482 | 17780 | UPSTREAM | CG32790-RA | "-"


*********************** Rank 1077 [Score  7.216800] GBROWSE*******************

 CG12756 in-situ | CG12756 | - | -802 | -1883 | UPSTREAM | CG12756-RA | "-"
insitu highlight CG5249 in-situ | CG5249 | + | 21941 | 39907 | UPSTREAM | CG5249-RA | "-"

*********************** Rank 1078 [Score  7.216100] GBROWSE*******************

 CG15883 in-situ | Obp18a | - | -8722 | -9510 | UPSTREAM | CG15883-RA | "-"
 CG7502 in-situ | CG7502 | - | 10363 | 6104 | DOWNSTREAM | CG7502-RA | "-"

*********************** Rank 1079 [Score  7.215900] GBROWSE*******************

 CG9169 in-situ | FucTD | + | -4740 | -3037 | DOWNSTREAM | CG9169-RA | "-"
 CG9173 in-situ | CG9173 | - | 2500 | 902 | DOWNSTREAM | CG9173-RA | "-"

*********************** Rank 1080 [Score  7.215600] GBROWSE*******************

 CG32632 in-situ | CG32632 | + | -2611 | 10550 | INTRAGENIC | intron:CG32632-RB:1 | CG32632-RB | "-"
insitu CG7107 in-situ | up | - | 22462 | 13528 | DOWNSTREAM | CG7107-RA | "-" | CG7107-RB | "-" | CG7107-RD | "-"

*********************** Rank 1081 [Score  7.215400] GBROWSE*******************

 CG17974 in-situ | CG17974 | - | -1705 | -3494 | UPSTREAM | CG17974-RA | "-"
 CG15671 in-situ | cv-2 | - | 14392 | 3313 | DOWNSTREAM | CG15671-RA | "-"

*********************** Rank 1082 [Score  7.212000] GBROWSE*******************

 CG14941 in-situ | esc | - | -9627 | -11849 | UPSTREAM | CG14941-RA | "-"
 CG31704 in-situ | CG31704 | + | 1683 | 1889 | UPSTREAM | CG31704-RA | "-"

*********************** Rank 1083 [Score  7.211900] GBROWSE*******************

 CG31438 in-situ | CG31438 | + | -11937 | -11204 | DOWNSTREAM | CG31438-RA | "-"
 CG6570 in-situ | lbl | - | 13059 | -11087 | INTRAGENIC | intron:CG6570-RA:1 | CG6570-RA | "-"

*********************** Rank 1084 [Score  7.211500] GBROWSE*******************

 CG6154 in-situ | CG6154 | + | -37251 | -29725 | DOWNSTREAM | CG6154-RA | "-" | CG6154-RB | "-"
 CG14559 in-situ | CG14559 | + | 18445 | 31734 | UPSTREAM | CG14559-RA | "-"


*********************** Rank 1085 [Score  7.211000] GBROWSE*******************

 CG5290 in-situ | CG5290 | - | -29564 | -32642 | UPSTREAM | CG5290-RA | "-"
 CG32193 in-situ | CG32193 | + | 19539 | 23559 | UPSTREAM | CG32193-RA | "-"


*********************** Rank 1086 [Score  7.209200] GBROWSE*******************

 CG30470 in-situ | CG30470 | + | -964 | -317 | DOWNSTREAM | CG30470-RA | "-"
 CG8180 in-situ | CG8180 | - | 20958 | 10590 | DOWNSTREAM | CG8180-RA | "-"


*********************** Rank 1087 [Score  7.209000] GBROWSE*******************

 CG15321 in-situ | CG15321 | - | -6544 | -7251 | UPSTREAM | CG15321-RA | "-"
insitu highlight CG12653 in-situ | btd | + | 3972 | 7357 | UPSTREAM | CG12653-RA | "-"

note: overlaps known module btd_head by 500 bases (module coords: 9429057-9430856)

*********************** Rank 1088 [Score  7.208400] GBROWSE*******************

 CG31381 in-situ | BEST:LD10347 | + | -2450 | -886 | DOWNSTREAM | CG31381-RA | "-"
insitu CG31121 in-situ | CG31121 | - | 14934 | 1787 | DOWNSTREAM | CG31121-RA | "-" | CG31121-RB | "-"

*********************** Rank 1089 [Score  7.208400] GBROWSE*******************

 CG8181 in-situ | CG8181 | - | -14643 | -17506 | UPSTREAM | CG8181-RA | "-"
 CG8172 in-situ | CG8172 | - | 1005 | -4932 | INTRAGENIC | intron:CG8172-RA:1 | CG8172-RA | "-"

*********************** Rank 1090 [Score  7.206100] GBROWSE*******************

insitu CG31619 in-situ | CG31619 | + | -6593 | 37922 | INTRAGENIC | intron:CG31619-RA:2 | intron:CG31619-RB:2 | CG31619-RA | "-" | CG31619-RB | "-"
 CG11628 in-situ | CG11628 | - | 66332 | 50122 | DOWNSTREAM | CG11628-RA | "-"


*********************** Rank 1091 [Score  7.204700] GBROWSE*******************

insitu CG6560 in-situ | CG6560 | - | -16313 | -17988 | UPSTREAM | CG6560-RA | "-"
 CG6690 in-situ | CG6690 | + | 8152 | 9840 | UPSTREAM | CG6690-RA | "-"

*********************** Rank 1092 [Score  7.203600] GBROWSE*******************

 CG1139 in-situ | CG1139 | + | -55059 | -52436 | DOWNSTREAM | CG1139-RA | "-"
 CG32308 in-situ | CG32308 | - | 11473 | 10712 | DOWNSTREAM | CG32308-RA | "-" | CG32308-RB | "-"

*********************** Rank 1093 [Score  7.203600] GBROWSE*******************

 CG7910 in-situ | CG7910 | + | -6111 | -3795 | DOWNSTREAM | CG7910-RA | "-"
 CG7900 in-situ | CG7900 | + | 6661 | 8445 | UPSTREAM | CG7900-RA | "-"

*********************** Rank 1094 [Score  7.201800] GBROWSE*******************

insitu highlight CG4889 in-situ | wg | + | -3508 | 5586 | INTRAGENIC | intron:CG4889-RA:2 | intron:CG4889-RB:1 | CG4889-RA | "-" | CG4889-RB | "-"
 CG4969 in-situ | Wnt6 | + | 40229 | 41611 | UPSTREAM | CG4969-RA | "-"

*********************** Rank 1095 [Score  7.200600] GBROWSE*******************

 CG9456 in-situ | Spn1 | + | -2245 | -375 | DOWNSTREAM | CG9456-RA | "-"
insitu CG9460 in-situ | CG9460 | + | 1157 | 3018 | UPSTREAM | CG9460-RA | "-"

*********************** Rank 1096 [Score  7.199200] GBROWSE*******************

 CG31909 in-situ | CG31909 | + | -6948 | -6404 | DOWNSTREAM | CG31909-RA | "-"
insitu CG4698 in-situ | Wnt4 | - | 12649 | -9059 | INTRAGENIC | intron:CG4698-RA:1 | CG4698-RA | "-"


*********************** Rank 1097 [Score  7.197900] GBROWSE*******************

 CG8671 in-situ | CG8671 | + | -8145 | 10469 | INTRAGENIC | intron:CG8671-RA:2 | intron:CG8671-RB:2 | CG8671-RA | "-" | CG8671-RB | "-"
 CG31622 in-situ | Gr39a | + | 19628 | 26580 | UPSTREAM | CG31622-RB | "-" | CG31622-RC | "-" | CG31622-RD | "-" | CG31622-RA | "-"

*********************** Rank 1098 [Score  7.197300] GBROWSE*******************

insitu CG9468 in-situ | CG9468 | - | -14318 | -17909 | UPSTREAM | CG9468-RA | "-"
insitu highlight CG18024 in-situ | SoxN | + | 30056 | 34101 | UPSTREAM | CG18024-RA | "-"

*********************** Rank 1099 [Score  7.194600] GBROWSE*******************

 CG1695 in-situ | CG1695 | + | -11161 | -6910 | DOWNSTREAM | CG1695-RA | "-" | CG1695-RB | "-"
 CG32506 in-situ | CG32506 | + | 8423 | 15086 | UPSTREAM | CG32506-RA | "-"

*********************** Rank 1100 [Score  7.194400] GBROWSE*******************

 CG31010 in-situ | CG31010 | - | -1219 | -2553 | UPSTREAM | CG31010-RA | "-"
 CG1340 in-situ | CG1340 | + | 18775 | 20705 | UPSTREAM | CG1340-RA | "-"

*********************** Rank 1101 [Score  7.192000] GBROWSE*******************

 CG11086 in-situ | CG11086 | + | -4135 | -3295 | DOWNSTREAM | CG11086-RA | "-"
 CG1850 in-situ | CG1850 | - | 7953 | 5795 | DOWNSTREAM | CG1850-RA | "-"

*********************** Rank 1102 [Score  7.191300] GBROWSE*******************

 CG13702 in-situ | AlCR2 | - | -3267 | -5342 | UPSTREAM | CG13702-RB | "-" | CG13702-RA | "-"
 CG32198 in-situ | CG32198 | + | 34920 | 35330 | UPSTREAM | CG32198-RB | "-"

*********************** Rank 1103 [Score  7.190500] GBROWSE*******************

 CG15006 in-situ | CG15006 | - | -338 | -1688 | UPSTREAM | CG15006-RA | "-"
 CG15007 in-situ | CG15007 | - | 2029 | 1477 | DOWNSTREAM | CG15007-RA | "-"

*********************** Rank 1104 [Score  7.186100] GBROWSE*******************

 CG18492 in-situ | Tak1 | - | -4576 | -13597 | UPSTREAM | CG18492-RA | "-"
 CG32504 in-situ | CG32504 | - | 3517 | 2933 | DOWNSTREAM | CG32504-RA | "-"

*********************** Rank 1105 [Score  7.185200] GBROWSE*******************

 CG12114 in-situ | CG12114 | + | -870 | 654 | INTRAGENIC | intron:CG12114-RA:1 | CG12114-RA | "-"
 CG3669 in-situ | CG3669 | + | 2937 | 4020 | UPSTREAM | CG3669-RA | "-"

*********************** Rank 1106 [Score  7.182600] GBROWSE*******************

 CG6023 in-situ | CG6023 | - | -12626 | -19668 | UPSTREAM | CG6023-RA | "-"
 CG6123 in-situ | CG6123 | - | 22297 | -9552 | INTRAGENIC | intron:CG6123-RA:2 | CG6123-RA | "-"


*********************** Rank 1107 [Score  7.180200] GBROWSE*******************

 CG14762 in-situ | CG14762 | - | -14645 | -19288 | UPSTREAM | CG14762-RA | "-"
insitu highlight CG18455 in-situ | Optix | + | 3080 | 13983 | UPSTREAM | CG18455-RA | "-" | CG18455-RB | "-"

*********************** Rank 1108 [Score  7.179200] GBROWSE*******************

 CG6098 in-situ | Lrr47 | + | -3404 | -1750 | DOWNSTREAM | CG6098-RA | "-"
insitu CG6113 in-situ | CG6113 | + | 8676 | 10820 | UPSTREAM | CG6113-RA | "-"

*********************** Rank 1109 [Score  7.178400] GBROWSE*******************

 CG32113 in-situ | CG32113 | + | -1007 | 12408 | INTRAGENIC | intron:CG32113-RA:2 | CG32113-RA | "-"
 CG10971 in-situ | CG10971 | - | 17416 | 12279 | DOWNSTREAM | CG10971-RA | "-" | CG10971-RB | "-"

*********************** Rank 1110 [Score  7.178000] GBROWSE*******************

insitu highlight CG5393 in-situ | apt | + | -27385 | 7418 | INTRAGENIC | intron:CG5393-RB:1 | intron:CG5393-RD:2 | intron:CG5393-RE:2 | intron:CG5393-RC:1 | intron:CG5393-RA:1 | CG5393-RB | "-" | CG5393-RD | "-" | CG5393-RE | "-" | CG5393-RC | "-" | CG5393-RA | "-"
 CG30182 in-situ | CG30182 | - | 8472 | 7241 | DOWNSTREAM | CG30182-RA | "-"

*********************** Rank 1111 [Score  7.176000] GBROWSE*******************

 CG13260 in-situ | CG13260 | + | -39587 | -37665 | DOWNSTREAM | CG13260-RA | "-"
 CG31815 in-situ | CG31815 | + | 4197 | 9561 | UPSTREAM | CG31815-RA | "-"

*********************** Rank 1112 [Score  7.175400] GBROWSE*******************

 CG9879 in-situ | CG9879 | + | -3673 | -2588 | DOWNSTREAM | CG9879-RA | "-"
 CG15396 in-situ | Gr23a | + | 1001 | 3376 | UPSTREAM | CG15396-RA | "-" | CG15396-RB | "-"

*********************** Rank 1113 [Score  7.174900] GBROWSE*******************

 CG11994 in-situ | Ada | + | -38734 | -37661 | DOWNSTREAM | CG11994-RA | "-"
 CG11997 in-situ | CG11997 | + | 73650 | 74960 | UPSTREAM | CG11997-RA | "-"

*********************** Rank 1114 [Score  7.174000] GBROWSE*******************

 CG13884 in-situ | CG13884 | - | -6533 | -7307 | UPSTREAM | CG13884-RA | "-"
insitu CG6883 in-situ | trh | - | 4346 | -6447 | INTRAGENIC | intron:CG6883-RA:2 | CG6883-RA | "-"

*********************** Rank 1115 [Score  7.172800] GBROWSE*******************

 CG2849 in-situ | Rala | - | -15552 | -30209 | UPSTREAM | CG2849-RB | "-" | CG2849-RA | "-" | CG2849-RC | "-"
 CG12462 in-situ | CG12462 | + | 11477 | 11779 | UPSTREAM | CG12462-RA | "-"


*********************** Rank 1116 [Score  7.170200] GBROWSE*******************

 CG4733 in-situ | CG4733 | + | -4312 | 1479 | INTRAGENIC | intron:CG4733-RA:3 | CG4733-RA | "-"
insitu CG4538 in-situ | CG4538 | - | 6628 | 1765 | DOWNSTREAM | CG4538-RA | "-" | CG4538-RB | "-"

*********************** Rank 1117 [Score  7.170000] GBROWSE*******************

 CG11282 in-situ | caps | + | -1164 | 47907 | INTRAGENIC | intron:CG11282-RA:1 | intron:CG11282-RB:1 | CG11282-RA | "-" | CG11282-RB | "-"
 CG32119 in-situ | CG32119 | - | 18849 | 17010 | DOWNSTREAM | CG32119-RA | "-"

*********************** Rank 1118 [Score  7.169800] GBROWSE*******************

 CG11634 in-situ | CG11634 | + | -17263 | -16176 | DOWNSTREAM | CG11634-RA | "-"
 CG2528 in-situ | CG2528 | - | 59309 | 57207 | DOWNSTREAM | CG2528-RA | "-"

*********************** Rank 1119 [Score  7.168900] GBROWSE*******************

 CG13479 in-situ | CG13479 | - | -13490 | -13839 | UPSTREAM | CG13479-RA | "-"
insitu highlight CG13475 in-situ | HGTX | - | 17948 | 1595 | DOWNSTREAM | CG13475-RA | "-"

*********************** Rank 1120 [Score  7.168000] GBROWSE*******************

 CG18404 in-situ | CG18404 | + | -14180 | -13264 | DOWNSTREAM | CG18404-RA | "-"
 CG15532 in-situ | hdc | + | 10572 | 94807 | UPSTREAM | CG15532-RA | "-" | CG15532-RC | "-" | CG15532-RB | "-"

*********************** Rank 1121 [Score  7.166600] GBROWSE*******************

 CG3262 in-situ | CG3262 | + | -4804 | -3841 | DOWNSTREAM | CG3262-RA | "-" | CG3262-RB | "-"
 CG12775 in-situ | CG12775 | - | 3111 | 2389 | DOWNSTREAM | CG12775-RA | "-"

*********************** Rank 1122 [Score  7.165300] GBROWSE*******************

 CG2652 in-situ | EG:155E2.5 | - | -2496 | -3441 | UPSTREAM | CG2652-RA | "-"
 CG2647 in-situ | per | + | 152 | 7352 | UPSTREAM | CG2647-RA | "-"

*********************** Rank 1123 [Score  7.164900] GBROWSE*******************

 CG12217 in-situ | PpV | - | -45233 | -47015 | UPSTREAM | CG12217-RA | "-"
 CG3367 in-situ | CG3367 | - | 50754 | 48960 | DOWNSTREAM | CG3367-RA | "-"

*********************** Rank 1124 [Score  7.163500] GBROWSE*******************

 CG14316 in-situ | CG14316 | + | -4743 | -3221 | DOWNSTREAM | CG14316-RA | "-"
 CG14315 in-situ | CG14315 | + | 1907 | 2560 | UPSTREAM | CG14315-RA | "-"


*********************** Rank 1125 [Score  7.160600] GBROWSE*******************

 CG3332 in-situ | CG3332 | - | -1748 | -6487 | UPSTREAM | CG3332-RB | "-" | CG3332-RA | "-"
 CG9664 in-situ | CG9664 | - | 12409 | 8964 | DOWNSTREAM | CG9664-RB | "-" | CG9664-RC | "-" | CG9664-RA | "-"


*********************** Rank 1126 [Score  7.160300] GBROWSE*******************

 CG31031 in-situ | CG31031 | + | -15684 | -15220 | DOWNSTREAM | CG31031-RA | "-"
 CG18682 in-situ | CG18682 | - | 45973 | 43292 | DOWNSTREAM | CG18682-RA | "-"

*********************** Rank 1127 [Score  7.160200] GBROWSE*******************

 CG7415 in-situ | CG7415 | + | -3110 | -45 | DOWNSTREAM | CG7415-RB | "-" | CG7415-RC | "-" | CG7415-RA | "-" | CG7415-RD | "-"
 CG7352 in-situ | CG7352 | + | 251 | 1783 | UPSTREAM | CG7352-RA | "-"

*********************** Rank 1128 [Score  7.160100] GBROWSE*******************

 CG14064 in-situ | beat-VI | + | -1608 | 53397 | INTRAGENIC | intron:CG14064-RA:1 | CG14064-RA | "-"
 CG1894 in-situ | CG1894 | + | 64840 | 66186 | UPSTREAM | CG1894-RA | "-"

*********************** Rank 1129 [Score  7.159800] GBROWSE*******************

insitu highlight CG10798 in-situ | dm | + | -8029 | 4804 | INTRAGENIC | intron:CG10798-RA:2 | CG10798-RA | "-"
 CG12535 in-situ | CG12535 | - | 12202 | 11417 | DOWNSTREAM | CG12535-RB | "-" | CG12535-RA | "-"

*********************** Rank 1130 [Score  7.159600] GBROWSE*******************

 CG4478 in-situ | Mst35Bb | - | -21951 | -23565 | UPSTREAM | CG4478-RA | "-"
 CG15277 in-situ | CG15277 | - | 4101 | 2855 | DOWNSTREAM | CG15277-RA | "-"

*********************** Rank 1131 [Score  7.157000] GBROWSE*******************

 CG12433 in-situ | CG12433 | + | -41770 | -40296 | DOWNSTREAM | CG12433-RA | "-"
 CG8949 in-situ | CG8949 | - | 10757 | 5551 | DOWNSTREAM | CG8949-RA | "-"

*********************** Rank 1132 [Score  7.151600] GBROWSE*******************

 CG2702 in-situ | CG2702 | + | -13939 | -11155 | DOWNSTREAM | CG2702-RA | "-"
 CG2723 in-situ | ImpE3 | - | 2682 | 447 | DOWNSTREAM | CG2723-RA | "-"

*********************** Rank 1133 [Score  7.150000] GBROWSE*******************

insitu highlight CG4807 in-situ | ab | + | -37810 | 9052 | INTRAGENIC | intron:CG4807-RB:7 | intron:CG4807-RA:7 | CG4807-RB | "-" | CG4807-RA | "-"
 CG32830 in-situ | CG32830 | + | 2545 | 4548 | UPSTREAM | CG32830-RA | "-"

*********************** Rank 1134 [Score  7.146500] GBROWSE*******************

 CG32653 in-situ | CG32653 | - | -846 | -3666 | UPSTREAM | CG32653-RA | "-"
 CG2555 in-situ | CG2555 | + | 20290 | 21401 | UPSTREAM | CG2555-RA | "-"

*********************** Rank 1135 [Score  7.146500] GBROWSE*******************

 CG14925 in-situ | CG14925 | - | -50426 | -51443 | UPSTREAM | CG14925-RA | "-"
 CG14926 in-situ | CG14926 | - | 7234 | 6284 | DOWNSTREAM | CG14926-RA | "-"

*********************** Rank 1136 [Score  7.146100] GBROWSE*******************

 CG2022 in-situ | CG2022 | - | -49179 | -51266 | UPSTREAM | CG2022-RA | "-"
insitu highlight CG2530 in-situ | corto | - | 48755 | 45690 | DOWNSTREAM | CG2530-RA | "-"

*********************** Rank 1137 [Score  7.146100] GBROWSE*******************

insitu CG7023 in-situ | CG7023 | + | -530 | 24954 | INTRAGENIC | intron:CG7023-RA:1 | intron:CG7023-RB:1 | CG7023-RA | "-" | CG7023-RB | "-"
 CG4725 in-situ | CG4725 | - | 9649 | 7424 | DOWNSTREAM | CG4725-RA | "-"

*********************** Rank 1138 [Score  7.145300] GBROWSE*******************

 CG5620 in-situ | CG5620 | - | -3549 | -12122 | UPSTREAM | CG5620-RA | "-"
 CG32098 in-situ | CG32098 | - | 39867 | 37388 | DOWNSTREAM | CG32098-RA | "-"

*********************** Rank 1139 [Score  7.145100] GBROWSE*******************

 CG30085 in-situ | CG30085 | - | -17630 | -22471 | UPSTREAM | CG30085-RA | "-"
 CG8355 in-situ | sli | - | 4079 | -14104 | INTRAGENIC | intron:CG8355-RA:1 | intron:CG8355-RC:1 | intron:CG8355-RB:1 | CG8355-RA | "-" | CG8355-RC | "-" | CG8355-RB | "-"

*********************** Rank 1140 [Score  7.143900] GBROWSE*******************

 CG30282 in-situ | CG30282 | - | -2750 | -5520 | UPSTREAM | CG30282-RA | "-"
 CG9847 in-situ | Fkbp13 | - | 3327 | -7428 | INTRAGENIC | intron:CG9847-RB:2 | intron:CG9847-RA:2 | CG9847-RB | "-" | CG9847-RA | "-"

*********************** Rank 1141 [Score  7.143500] GBROWSE*******************

 CG13353 in-situ | CG13353 | + | -34710 | -33359 | DOWNSTREAM | CG13353-RA | "-"
 CG30483 in-situ | Prosap | - | 15869 | -63607 | INTRAGENIC | intron:CG30483-RA:1 | CG30483-RA | "-"

*********************** Rank 1142 [Score  7.141100] GBROWSE*******************

 CG13285 in-situ | CG13285 | - | -10985 | -11817 | UPSTREAM | CG13285-RA | "-"
 CG10625 in-situ | CG10625 | - | 4515 | -8029 | INTRAGENIC | intron:CG10625-RA:2 | intron:CG10625-RD:1 | intron:CG10625-RC:2 | intron:CG10625-RB:2 | CG10625-RA | "-" | CG10625-RD | "-" | CG10625-RC | "-" | CG10625-RB | "-"

*********************** Rank 1143 [Score  7.139500] GBROWSE*******************

 CG31515 in-situ | CG31515 | - | -7550 | -7996 | UPSTREAM | CG31515-RA | "-"
 CG17379 in-situ | CG17379 | + | 5351 | 11063 | UPSTREAM | CG17379-RA | "-"

*********************** Rank 1144 [Score  7.138200] GBROWSE*******************

 CG8668 in-situ | CG8668 | + | -3045 | 3921 | INTRAGENIC | intron:CG8668-RA:1 | CG8668-RA | "-"
 CG31606 in-situ | CG31606 | + | 17561 | 18210 | UPSTREAM | CG31606-RB | "-" | CG31606-RA | "-"

*********************** Rank 1145 [Score  7.138100] GBROWSE*******************

 CG1438 in-situ | Cyp4c3 | + | -13251 | -6575 | DOWNSTREAM | CG1438-RA | "-"
 CG1447 in-situ | Ptx1 | + | 5676 | 23470 | UPSTREAM | CG1447-RA | "-" | CG1447-RB | "-"

*********************** Rank 1146 [Score  7.137900] GBROWSE*******************

 CG10160 in-situ | ImpL3 | - | -47628 | -50832 | UPSTREAM | CG10160-RA | "-"
 CG32401 in-situ | CG32401 | + | 10290 | 11777 | UPSTREAM | CG32401-RA | "-"

*********************** Rank 1147 [Score  7.137800] GBROWSE*******************

 CG7855 in-situ | timeout | + | -10558 | 64667 | INTRAGENIC | intron:CG7855-RA:6 | CG7855-RA | "-"
 CG17319 in-situ | CG17319 | - | 29340 | 26902 | DOWNSTREAM | CG17319-RA | "-"

*********************** Rank 1148 [Score  7.136500] GBROWSE*******************

 CG12607 in-situ | CG12607 | + | -7231 | -5742 | DOWNSTREAM | CG12607-RB | "-"
 CG11345 in-situ | CG11345 | - | 2577 | 1761 | DOWNSTREAM | CG11345-RA | "-"

*********************** Rank 1149 [Score  7.135200] GBROWSE*******************

 CG12626 in-situ | CG12626 | - | -4746 | -5130 | UPSTREAM | CG12626-RA | "-"
 CG11160 in-situ | CG11160 | + | 2297 | 7084 | UPSTREAM | CG11160-RA | "-" | CG11160-RB | "-"

*********************** Rank 1150 [Score  7.132100] GBROWSE*******************

insitu CG9403 in-situ | jing | + | -7869 | -2252 | DOWNSTREAM | CG9403-RB | "-" | CG9403-RA | "-"
 CG3161 in-situ | Vha16 | - | 11480 | 5265 | DOWNSTREAM | CG3161-RA | "-" | CG3161-RB | "-" | CG3161-RC | "-" | CG3161-RD | "-"

*********************** Rank 1151 [Score  7.131700] GBROWSE*******************

 CG10943 in-situ | CG10943 | - | -2835 | -3761 | UPSTREAM | CG10943-RA | "-"
 CG14120 in-situ | CG14120 | + | 19499 | 23614 | UPSTREAM | CG14120-RA | "-"

*********************** Rank 1152 [Score  7.130700] GBROWSE*******************

insitu CG32499 in-situ | CG32499 | - | -114096 | -163163 | UPSTREAM | CG32499-RA | "-"
 CG12446 in-situ | CG12446 | - | 65130 | 62482 | DOWNSTREAM | CG12446-RA | "-"

*********************** Rank 1153 [Score  7.130100] GBROWSE*******************

 CG12078 in-situ | CG12078 | - | -10411 | -11121 | UPSTREAM | CG12078-RA | "-"
 CG14959 in-situ | CG14959 | + | 3684 | 13285 | UPSTREAM | CG14959-RB | "-" | CG14959-RA | "-"

*********************** Rank 1154 [Score  7.128300] GBROWSE*******************

 CG6986 in-situ | CG6986 | + | -12219 | 9357 | INTRAGENIC | intron:CG6986-RB:2 | intron:CG6986-RA:2 | CG6986-RB | "-" | CG6986-RA | "-"
 CG12683 in-situ | CG12683 | - | 31486 | 31244 | DOWNSTREAM | CG12683-RA | "-"

*********************** Rank 1155 [Score  7.126600] GBROWSE*******************

 CG32064 in-situ | CG32064 | + | -5910 | -3836 | DOWNSTREAM | CG32064-RA | "-"
 CG32062 in-situ | CG32062 | + | 19234 | 96650 | UPSTREAM | CG32062-RB | "-" | CG32062-RD | "-"

*********************** Rank 1156 [Score  7.125100] GBROWSE*******************

 CG5499 in-situ | His2Av | + | -4432 | -2455 | DOWNSTREAM | CG5499-RA | "-"
insitu CG6378 in-situ | BM-40-SPARC | - | 653 | -2131 | INTRAGENIC | intron:CG6378-RA:1 | CG6378-RA | "-"

*********************** Rank 1157 [Score  7.124300] GBROWSE*******************

 CG32140 in-situ | CG32140 | + | -12325 | -5589 | DOWNSTREAM | CG32140-RA | "-" | CG32140-RB | "-"
 CG7924 in-situ | CG7924 | + | 1399 | 2540 | UPSTREAM | CG7924-RA | "-"

*********************** Rank 1158 [Score  7.124000] GBROWSE*******************

 CG32203 in-situ | CG32203 | - | -16830 | -25387 | UPSTREAM | CG32203-RA | "-"
insitu highlight CG9739 in-situ | fz2 | - | 17305 | -10423 | INTRAGENIC | intron:CG9739-RB:2 | CG9739-RB | "-" | CG9739-RA | "-"

*********************** Rank 1159 [Score  7.123800] GBROWSE*******************

insitu CG9403 in-situ | jing | + | -15969 | -10352 | DOWNSTREAM | CG9403-RB | "-" | CG9403-RA | "-"
 CG3161 in-situ | Vha16 | - | 3380 | -2835 | INTRAGENIC | intron:CG3161-RA:2 | intron:CG3161-RB:2 | intron:CG3161-RC:1 | intron:CG3161-RD:2 | CG3161-RA | "-" | CG3161-RB | "-" | CG3161-RC | "-" | CG3161-RD | "-"

*********************** Rank 1160 [Score  7.123200] GBROWSE*******************

 CG32193 in-situ | CG32193 | + | -10661 | -6641 | DOWNSTREAM | CG32193-RA | "-"
 CG32192 in-situ | CG32192 | + | 67996 | 68731 | UPSTREAM | CG32192-RA | "-" | CG32192-RB | "-"

*********************** Rank 1161 [Score  7.121000] GBROWSE*******************

 CG4788 in-situ | CG4788 | + | -16950 | -15561 | DOWNSTREAM | CG4788-RA | "-"
insitu highlight CG4807 in-situ | ab | + | 14690 | 61552 | UPSTREAM | CG4807-RB | "-" | CG4807-RA | "-"

*********************** Rank 1162 [Score  7.119900] GBROWSE*******************

 CG3837 in-situ | CG3837 | + | -91130 | -87505 | DOWNSTREAM | CG3837-RA | "-"
 CG14861 in-situ | CG14861 | + | 4960 | 6227 | UPSTREAM | CG14861-RA | "-"

*********************** Rank 1163 [Score  7.117300] GBROWSE*******************

insitu CG5359 in-situ | CG5359 | - | -4885 | -6391 | UPSTREAM | CG5359-RA | "-"
 CG31407 in-situ | CG31407 | - | 17448 | 16686 | DOWNSTREAM | CG31407-RA | "-"

*********************** Rank 1164 [Score  7.114500] GBROWSE*******************

 CG16970 in-situ | CG16970 | - | -2358 | -5253 | UPSTREAM | CG16970-RA | "-"
 CG16826 in-situ | CG16826 | + | 34611 | 35779 | UPSTREAM | CG16826-RA | "-"

*********************** Rank 1165 [Score  7.113400] GBROWSE*******************

 CG5308 in-situ | CG5308 | + | -8526 | -2555 | DOWNSTREAM | CG5308-RA | "-" | CG5308-RB | "-"
 CG12593 in-situ | CG12593 | - | 28385 | 25990 | DOWNSTREAM | CG12593-RA | "-"


*********************** Rank 1166 [Score  7.112900] GBROWSE*******************

 CG5060 in-situ | CG5060 | + | -678 | 37657 | INTRAGENIC | intron:CG5060-RA:1 | CG5060-RA | "-"
 CG10883 in-situ | CG10883 | - | 35352 | 34819 | DOWNSTREAM | CG10883-RA | "-"

*********************** Rank 1167 [Score  7.112000] GBROWSE*******************

 CG14116 in-situ | CG14116 | + | -19724 | -17674 | DOWNSTREAM | CG14116-RA | "-"
 CG17300 in-situ | CG17300 | - | 5686 | 4784 | DOWNSTREAM | CG17300-RA | "-"

*********************** Rank 1168 [Score  7.111500] GBROWSE*******************

 CG15631 in-situ | CG15631 | - | -20473 | -22522 | UPSTREAM | CG15631-RA | "-"
 CG15630 in-situ | CG15630 | - | 39111 | -19384 | INTRAGENIC | intron:CG15630-RA:1 | CG15630-RA | "-"

*********************** Rank 1169 [Score  7.111500] GBROWSE*******************

 CG14318 in-situ | CG14318 | - | -5065 | -11817 | UPSTREAM | CG14318-RA | "-"
insitu CG7713 in-situ | CG7713 | + | 6034 | 7254 | UPSTREAM | CG7713-RA | "-"

*********************** Rank 1170 [Score  7.111300] GBROWSE*******************

 CG12189 in-situ | Rev1 | + | -12560 | -8946 | DOWNSTREAM | CG12189-RA | "-"
 CG17129 in-situ | CG17129 | - | 13401 | 10951 | DOWNSTREAM | CG17129-RC | "-" | CG17129-RA | "-" | CG17129-RB | "-"

*********************** Rank 1171 [Score  7.111100] GBROWSE*******************

 CG17341 in-situ | BG:DS01523.1 | + | -22672 | -19923 | DOWNSTREAM | CG17341-RA | "-"
insitu CG32972 in-situ | BG:DS01523.2 | - | 1683 | -15492 | INTRAGENIC | intron:CG32972-RB:1 | CG32972-RB | "-"

*********************** Rank 1172 [Score  7.111100] GBROWSE*******************

 CG15899 in-situ | Ca-alpha1T | - | -15936 | -54271 | UPSTREAM | CG15899-RB | "-"
 CG32750 in-situ | CG32750 | - | 35093 | 33464 | DOWNSTREAM | CG32750-RA | "-"

*********************** Rank 1173 [Score  7.108900] GBROWSE*******************

 CG11522 in-situ | CG11522 | - | -1267 | -2725 | UPSTREAM | CG11522-RA | "-" | CG11522-RB | "-"
 CG1775 in-situ | Med | + | 13536 | 18244 | UPSTREAM | CG1775-RA | "-" | CG1775-RB | "-" | CG1775-RC | "-" | CG1775-RD | "-"

*********************** Rank 1174 [Score  7.107700] GBROWSE*******************

 CG7423 in-situ | CG7423 | - | -10312 | -10686 | UPSTREAM | CG7423-RA | "-"
 CG15882 in-situ | CG15882 | - | 30786 | 30310 | DOWNSTREAM | CG15882-RA | "-"

*********************** Rank 1175 [Score  7.107700] GBROWSE*******************

 CG10718 in-situ | neb | + | -2767 | 11183 | INTRAGENIC | intron:CG10718-RA:1 | CG10718-RA | "-"
insitu highlight CG10746 in-situ | fok | - | 7003 | 3765 | DOWNSTREAM | CG10746-RB | "-" | CG10746-RA | "-"

*********************** Rank 1176 [Score  7.107200] GBROWSE*******************

 CG5518 in-situ | sda | + | -11030 | 18550 | INTRAGENIC | intron:CG5518-RA:3 | CG5518-RA | "-"
 CG14252 in-situ | CG14252 | + | 18771 | 20448 | UPSTREAM | CG14252-RA | "-"

*********************** Rank 1177 [Score  7.107100] GBROWSE*******************

 CG10897 in-situ | tou | - | -556 | -37505 | UPSTREAM | CG10897-RA | "-" | CG10897-RC | "-" | CG10897-RD | "-" | CG10897-RB | "-"
insitu CG9006 in-situ | CG9006 | - | 2623 | 482 | DOWNSTREAM | CG9006-RA | "-"

*********************** Rank 1178 [Score  7.103900] GBROWSE*******************

 CG14247 in-situ | CG14247 | + | -6021 | -5494 | DOWNSTREAM | CG14247-RA | "-"
 CG5490 in-situ | Tl | + | 55180 | 98540 | UPSTREAM | CG5490-RB | "-" | CG5490-RA | "-"

*********************** Rank 1179 [Score  7.103600] GBROWSE*******************

 CG2045 in-situ | Ser7 | + | -2737 | -885 | DOWNSTREAM | CG2045-RA | "-"
 CG15245 in-situ | CG15245 | - | 988 | 231 | DOWNSTREAM | CG15245-RA | "-"

*********************** Rank 1180 [Score  7.103500] GBROWSE*******************

 CG7344 in-situ | CG7344 | + | -15999 | 1354 | INTRAGENIC | intron:CG7344-RA:1 | CG7344-RA | "-"
 CG6240 in-situ | CG6240 | - | 13262 | 12552 | DOWNSTREAM | CG6240-RA | "-"

*********************** Rank 1181 [Score  7.103100] GBROWSE*******************

insitu highlight CG10145 in-situ | mspo | - | -3303 | -44989 | UPSTREAM | CG10145-RA | "-"
 CG12865 in-situ | CG12865 | + | 21795 | 22364 | UPSTREAM | CG12865-RA | "-"

*********************** Rank 1182 [Score  7.102600] GBROWSE*******************

 CG7391 in-situ | Clk | - | -27068 | -38700 | UPSTREAM | CG7391-RA | "-" | CG7391-RB | "-"
insitu CG32369 in-situ | CG32369 | - | 1735 | -24262 | INTRAGENIC | intron:CG32369-RA:1 | CG32369-RA | "-" | CG32369-RB | "-"

*********************** Rank 1183 [Score  7.101900] GBROWSE*******************

 CG16970 in-situ | CG16970 | - | -20958 | -23853 | UPSTREAM | CG16970-RA | "-"
 CG16826 in-situ | CG16826 | + | 16011 | 17179 | UPSTREAM | CG16826-RA | "-"

*********************** Rank 1184 [Score  7.100300] GBROWSE*******************

 CG14792 in-situ | sta | - | -4700 | -6325 | UPSTREAM | CG14792-RA | "-" | CG14792-RB | "-" | CG14792-RD | "-"
 CG14793 in-situ | Nmdar2 | - | 5569 | 324 | DOWNSTREAM | CG14793-RA | "-" | CG14793-RB | "-"

*********************** Rank 1185 [Score  7.098600] GBROWSE*******************

 CG11405 in-situ | A3-3 | - | -8607 | -17523 | UPSTREAM | CG11405-RA | "-"
 CG32812 in-situ | EG:114D9.1 | + | 27783 | 28460 | UPSTREAM | CG32812-RA | "-"

*********************** Rank 1186 [Score  7.097000] GBROWSE*******************

 CG7499 in-situ | Rh50 | + | -27623 | -18965 | DOWNSTREAM | CG7499-RA | "-"
 CG32233 in-situ | CG32233 | + | 11442 | 12050 | UPSTREAM | CG32233-RA | "-"

*********************** Rank 1187 [Score  7.094400] GBROWSE*******************

 CG31806 in-situ | CG31806 | + | -10162 | -9599 | DOWNSTREAM | CG31806-RA | "-"
 CG6304 in-situ | CG6304 | - | 17838 | 16331 | DOWNSTREAM | CG6304-RA | "-"

*********************** Rank 1188 [Score  7.091100] GBROWSE*******************

 CG9850 in-situ | CG9850 | - | -15006 | -30368 | UPSTREAM | CG9850-RA | "-"
 CG15800 in-situ | CG15800 | - | 7110 | 6636 | DOWNSTREAM | CG15800-RA | "-"

*********************** Rank 1189 [Score  7.090500] GBROWSE*******************

 CG14547 in-situ | CG14547 | - | -13388 | -13783 | UPSTREAM | CG14547-RA | "-"
 CG14540 in-situ | CG14540 | + | 4463 | 6428 | UPSTREAM | CG14540-RA | "-"

*********************** Rank 1190 [Score  7.088900] GBROWSE*******************

 CG1470 in-situ | Gycbeta100B | + | -12793 | 24258 | INTRAGENIC | intron:CG1470-RA:3 | CG1470-RA | "-"
 CG31006 in-situ | CG31006 | - | 21841 | 10893 | DOWNSTREAM | CG31006-RB | "-" | CG31006-RA | "-"

*********************** Rank 1191 [Score  7.086900] GBROWSE*******************

 CG3250 in-situ | Os-C | + | -10826 | -9989 | DOWNSTREAM | CG3250-RA | "-"
 CG31466 in-situ | CG31466 | + | 5877 | 6446 | UPSTREAM | CG31466-RA | "-"

*********************** Rank 1192 [Score  7.083400] GBROWSE*******************

insitu CG6560 in-situ | CG6560 | - | -22813 | -24488 | UPSTREAM | CG6560-RA | "-"
 CG6690 in-situ | CG6690 | + | 1652 | 3340 | UPSTREAM | CG6690-RA | "-"


*********************** Rank 1193 [Score  7.082600] GBROWSE*******************

 CG17723 in-situ | CG17723 | + | -749 | 9009 | INTRAGENIC | intron:CG17723-RA:1 | intron:CG17723-RB:1 | intron:CG17723-RC:1 | intron:CG17723-RD:1 | CG17723-RA | "-" | CG17723-RB | "-" | CG17723-RC | "-" | CG17723-RD | "-" | CG17723-RE | "-"
 CG32279 in-situ | CG32279 | + | 9796 | 10116 | UPSTREAM | CG32279-RA | "-"


*********************** Rank 1194 [Score  7.082000] GBROWSE*******************

 CG31807 in-situ | CG31807 | - | -27450 | -27917 | UPSTREAM | CG31807-RA | "-"
 CG13263 in-situ | Cyt-c-d | + | 6042 | 9826 | UPSTREAM | CG13263-RA | "-"

*********************** Rank 1195 [Score  7.081900] GBROWSE*******************

 CG14793 in-situ | Nmdar2 | - | -3081 | -8326 | UPSTREAM | CG14793-RA | "-" | CG14793-RB | "-"
 CG14794 in-situ | CG14794 | - | 5517 | 4993 | DOWNSTREAM | CG14794-RA | "-"

*********************** Rank 1196 [Score  7.081600] GBROWSE*******************

insitu CG5254 in-situ | EG:BACR19J1.2 | + | -3025 | -490 | DOWNSTREAM | CG5254-RA | "-"
 CG5273 in-situ | EG:BACR19J1.3 | + | 382 | 2148 | UPSTREAM | CG5273-RA | "-" | CG5273-RC | "-" | CG5273-RB | "-"

*********************** Rank 1197 [Score  7.081200] GBROWSE*******************

 CG13123 in-situ | CG13123 | - | -25074 | -26209 | UPSTREAM | CG13123-RA | "-"
 CG31880 in-situ | CG31880 | + | 16349 | 17124 | UPSTREAM | CG31880-RA | "-"

*********************** Rank 1198 [Score  7.081100] GBROWSE*******************

 CG6873 in-situ | CG6873 | - | -2117 | -2563 | UPSTREAM | CG6873-RA | "-"
 CG12609 in-situ | CG12609 | - | 6465 | 4528 | DOWNSTREAM | CG12609-RB | "-" | CG12609-RA | "-"

*********************** Rank 1199 [Score  7.079900] GBROWSE*******************

 CG32773 in-situ | CG32773 | - | -14883 | -15302 | UPSTREAM | CG32773-RA | "-"
 CG3578 in-situ | bi | + | 9722 | 81330 | UPSTREAM | CG3578-RA | "-"

*********************** Rank 1200 [Score  7.079500] GBROWSE*******************

 CG13385 in-situ | CG13385 | + | -2106 | -933 | DOWNSTREAM | CG13385-RA | "-"
 CG13386 in-situ | CG13386 | + | 225 | 1153 | UPSTREAM | CG13386-RA | "-"

*********************** Rank 1201 [Score  7.079400] GBROWSE*******************

insitu CG4089 in-situ | CG4089 | + | -10819 | -8615 | DOWNSTREAM | CG4089-RA | "-"
 CG4655 in-situ | CG4655 | + | 11283 | 15941 | UPSTREAM | CG4655-RA | "-" | CG4655-RB | "-"

*********************** Rank 1202 [Score  7.079300] GBROWSE*******************

 CG12296 in-situ | klu | - | -6294 | -33392 | UPSTREAM | CG12296-RA | "-"
 CG7923 in-situ | Fad2 | + | 8421 | 9488 | UPSTREAM | CG7923-RA | "-"

*********************** Rank 1203 [Score  7.079300] GBROWSE*******************

 CG1462 in-situ | Aph-4 | + | -680 | 2934 | INTRAGENIC | intron:CG1462-RA:1 | CG1462-RA | "-" | CG1462-RB | "-"
insitu CG1715 in-situ | l(3)03670 | - | 4300 | 2999 | DOWNSTREAM | CG1715-RA | "-"

*********************** Rank 1204 [Score  7.078600] GBROWSE*******************

insitu highlight CG4125 in-situ | rst | - | -30547 | -52172 | UPSTREAM | CG4125-RA | "-"
 CG4116 in-situ | CG4116 | - | 63183 | 62389 | DOWNSTREAM | CG4116-RA | "-"

*********************** Rank 1205 [Score  7.076700] GBROWSE*******************

 CG13198 in-situ | CG13198 | + | -10089 | -9225 | DOWNSTREAM | CG13198-RA | "-"
insitu CG30035 in-situ | CG30035 | + | 767 | 9795 | UPSTREAM | CG30035-RA | "-" | CG30035-RB | "-"

*********************** Rank 1206 [Score  7.075100] GBROWSE*******************

insitu highlight CG31043 in-situ | gukh | + | -18986 | 19427 | INTRAGENIC | intron:CG31043-RA:1 | intron:CG31043-RB:1 | CG31043-RA | "-" | CG31043-RB | "-"
 CG6005 in-situ | CG6005 | - | 21011 | 19590 | DOWNSTREAM | CG6005-RA | "-"

*********************** Rank 1207 [Score  7.074900] GBROWSE*******************

insitu highlight CG1849 in-situ | run | + | -26444 | -23559 | DOWNSTREAM | CG1849-RA | "-"
 CG1324 in-situ | CG1324 | - | 15075 | 13859 | DOWNSTREAM | CG1324-RA | "-"

*********************** Rank 1208 [Score  7.072900] GBROWSE*******************

 CG7343 in-situ | CG7343 | - | -26088 | -29030 | UPSTREAM | CG7343-RA | "-"
 CG3610 in-situ | CG3610 | + | 8833 | 10527 | UPSTREAM | CG3610-RA | "-"

*********************** Rank 1209 [Score  7.072900] GBROWSE*******************

 CG11726 in-situ | CG11726 | + | -17747 | -16872 | DOWNSTREAM | CG11726-RA | "-"
 CG7560 in-situ | CG7560 | + | 12632 | 13877 | UPSTREAM | CG7560-RA | "-"

*********************** Rank 1210 [Score  7.070100] GBROWSE*******************

 CG7520 in-situ | CG7520 | + | -16515 | -5644 | DOWNSTREAM | CG7520-RA | "-"
 CG7515 in-situ | CG7515 | + | 6430 | 8013 | UPSTREAM | CG7515-RA | "-"

*********************** Rank 1211 [Score  7.069100] GBROWSE*******************

 CG6850 in-situ | Ugt | + | -9374 | -3869 | DOWNSTREAM | CG6850-RA | "-"
 CG3961 in-situ | CG3961 | - | 8361 | -3867 | INTRAGENIC | intron:CG3961-RA:3 | intron:CG3961-RC:4 | intron:CG3961-RB:2 | CG3961-RA | "-" | CG3961-RC | "-" | CG3961-RB | "-"

*********************** Rank 1212 [Score  7.069000] GBROWSE*******************

 CG11456 in-situ | CG11456 | + | -9623 | -7284 | DOWNSTREAM | CG11456-RA | "-"
insitu CG32432 in-situ | CG32432 | - | 33564 | -7299 | INTRAGENIC | intron:CG32432-RA:9 | CG32432-RA | "-"

*********************** Rank 1213 [Score  7.067700] GBROWSE*******************

 CG1447 in-situ | Ptx1 | + | -7474 | 10320 | INTRAGENIC | intron:CG1447-RA:2 | CG1447-RA | "-" | CG1447-RB | "-"
 CG15549 in-situ | CG15549 | - | 16145 | 15034 | DOWNSTREAM | CG15549-RA | "-"

*********************** Rank 1214 [Score  7.065400] GBROWSE*******************

insitu CG11453 in-situ | CG11453 | + | -37538 | -35531 | DOWNSTREAM | CG11453-RA | "-"
insitu CG4608 in-situ | bnl | - | 15756 | -26988 | INTRAGENIC | intron:CG4608-RA:2 | intron:CG4608-RB:2 | CG4608-RA | "-" | CG4608-RB | "-"

*********************** Rank 1215 [Score  7.064500] GBROWSE*******************

 CG14127 in-situ | CG14127 | + | -8618 | -6456 | DOWNSTREAM | CG14127-RA | "-"
 CG5897 in-situ | CG5897 | - | 670 | -2117 | INTRAGENIC | intron:CG5897-RA:1 | CG5897-RA | "-"


*********************** Rank 1216 [Score  7.064400] GBROWSE*******************

 CG12217 in-situ | PpV | - | -33633 | -35415 | UPSTREAM | CG12217-RA | "-"
 CG3367 in-situ | CG3367 | - | 62354 | 60560 | DOWNSTREAM | CG3367-RA | "-"

*********************** Rank 1217 [Score  7.063800] GBROWSE*******************

 CG18313 in-situ | CG18313 | + | -7236 | -6289 | DOWNSTREAM | CG18313-RA | "-"
 CG32596 in-situ | CG32596 | - | 47403 | 46652 | DOWNSTREAM | CG32596-RA | "-"

*********************** Rank 1218 [Score  7.063500] GBROWSE*******************

 CG6099 in-situ | m4 | - | -3320 | -3778 | UPSTREAM | CG6099-RA | "-"
insitu highlight CG6096 in-situ | HLHm5 | - | 1922 | 1037 | DOWNSTREAM | CG6096-RA | "-"

*********************** Rank 1219 [Score  7.063300] GBROWSE*******************

 CG6112 in-situ | CG6112 | - | -24431 | -28822 | UPSTREAM | CG6112-RA | "-"
 CG32151 in-situ | CG32151 | + | 7211 | 7825 | UPSTREAM | CG32151-RA | "-"

*********************** Rank 1220 [Score  7.063200] GBROWSE*******************

 CG17244 in-situ | CG17244 | + | -3851 | -3231 | DOWNSTREAM | CG17244-RA | "-"
 CG17241 in-situ | Or94a | + | 6159 | 7381 | UPSTREAM | CG17241-RA | "-"

*********************** Rank 1221 [Score  7.060800] GBROWSE*******************

 CG8379 in-situ | CG8379 | + | -30681 | -27828 | DOWNSTREAM | CG8379-RA | "-"
 CG9731 in-situ | CG9731 | - | 7669 | 6881 | DOWNSTREAM | CG9731-RA | "-"

*********************** Rank 1222 [Score  7.059600] GBROWSE*******************

 CG33197 in-situ | CG33197 | + | -64322 | 45994 | INTRAGENIC | intron:CG33197-RA:2 | intron:CG33197-RB:2 | intron:CG33197-RD:2 | CG33197-RA | "-" | CG33197-RB | "-" | CG33197-RC | "-" | CG33197-RD | "-"
 CG14477 in-situ | mm | + | 21219 | 45994 | UPSTREAM | CG14477-RA | "-"

*********************** Rank 1223 [Score  7.059200] GBROWSE*******************

insitu CG2092 in-situ | scra | + | -13530 | -8395 | DOWNSTREAM | CG2092-RA | "-" | CG2092-RB | "-"
 CG1358 in-situ | CG1358 | - | 10886 | -8384 | INTRAGENIC | intron:CG1358-RA:3 | intron:CG1358-RB:2 | intron:CG1358-RC:2 | CG1358-RA | "-" | CG1358-RB | "-" | CG1358-RC | "-"

*********************** Rank 1224 [Score  7.057900] GBROWSE*******************

 CG12729 in-situ | CG12729 | + | -16995 | -16169 | DOWNSTREAM | CG12729-RA | "-"
 CG15764 in-situ | CG15764 | + | 6821 | 7789 | UPSTREAM | CG15764-RA | "-"

*********************** Rank 1225 [Score  7.056800] GBROWSE*******************

 CG3162 in-situ | CG3162 | - | -7293 | -8707 | UPSTREAM | CG3162-RA | "-"
 CG3092 in-situ | CG3092 | + | 13794 | 15096 | UPSTREAM | CG3092-RA | "-"

*********************** Rank 1226 [Score  7.056500] GBROWSE*******************

 CG12516 in-situ | CG12516 | - | -922 | -1797 | UPSTREAM | CG12516-RA | "-"
insitu highlight CG14066 in-situ | larp | - | 22821 | 12712 | DOWNSTREAM | CG14066-RB | "-" | CG14066-RC | "-" | CG14066-RD | "-" | CG14066-RA | "-"

*********************** Rank 1227 [Score  7.054600] GBROWSE*******************

 CG12800 in-situ | Cyp6d4 | + | -797 | 1190 | INTRAGENIC | intron:CG12800-RA:1 | CG12800-RA | "-"
 CG4910 in-situ | Ccap | - | 2345 | 1636 | DOWNSTREAM | CG4910-RA | "-"

*********************** Rank 1228 [Score  7.054300] GBROWSE*******************

 CG1867 in-situ | Or98b | + | -53670 | -52292 | DOWNSTREAM | CG1867-RA | "-"
 CG14064 in-situ | beat-VI | + | 12292 | 67297 | UPSTREAM | CG14064-RA | "-"

*********************** Rank 1229 [Score  7.053000] GBROWSE*******************

 CG7342 in-situ | CG7342 | + | -1550 | 817 | INTRAGENIC | intron:CG7342-RA:4 | CG7342-RA | "-"
 CG17751 in-situ | CG17751 | + | 1979 | 3421 | UPSTREAM | CG17751-RA | "-"


*********************** Rank 1230 [Score  7.052900] GBROWSE*******************

 CG11994 in-situ | Ada | + | -46084 | -45011 | DOWNSTREAM | CG11994-RA | "-"
 CG11997 in-situ | CG11997 | + | 66300 | 67610 | UPSTREAM | CG11997-RA | "-"

*********************** Rank 1231 [Score  7.052700] GBROWSE*******************

 CG17368 in-situ | CG17368 | - | -7333 | -8975 | UPSTREAM | CG17368-RA | "-"
 CG8865 in-situ | Rgl | - | 25478 | 7121 | DOWNSTREAM | CG8865-RB | "-" | CG8865-RD | "-" | CG8865-RC | "-" | CG8865-RA | "-"

*********************** Rank 1232 [Score  7.052200] GBROWSE*******************

 CG32775 in-situ | BcDNA:GH05057 | + | -69638 | -68037 | DOWNSTREAM | CG32775-RA | "-"
 CG3665 in-situ | Fas2 | - | 5206 | -66512 | INTRAGENIC | intron:CG3665-RA:2 | intron:CG3665-RB:2 | intron:CG3665-RC:2 | CG3665-RA | "-" | CG3665-RB | "-" | CG3665-RC | "-"


*********************** Rank 1233 [Score  7.052000] GBROWSE*******************

 CG11913 in-situ | CG11913 | + | -20763 | -19142 | DOWNSTREAM | CG11913-RA | "-"
insitu CG11910 in-situ | CG11910 | - | 3729 | 1992 | DOWNSTREAM | CG11910-RA | "-"


*********************** Rank 1234 [Score  7.050700] GBROWSE*******************

 CG12680 in-situ | CG12680 | + | -16302 | -15850 | DOWNSTREAM | CG12680-RA | "-"
 CG6824 in-situ | ovo | + | 34261 | 55668 | UPSTREAM | CG6824-RB | "-" | CG6824-RC | "-" | CG6824-RA | "-"

*********************** Rank 1235 [Score  7.046400] GBROWSE*******************

 CG31031 in-situ | CG31031 | + | -52284 | -51820 | DOWNSTREAM | CG31031-RA | "-"
 CG18682 in-situ | CG18682 | - | 9373 | 6692 | DOWNSTREAM | CG18682-RA | "-"

*********************** Rank 1236 [Score  7.042400] GBROWSE*******************

insitu CG1147 in-situ | NPFR1 | + | -9378 | -1364 | DOWNSTREAM | CG1147-RA | "-"
insitu CG15589 in-situ | CG15589 | + | 2035 | 10903 | UPSTREAM | CG15589-RA | "-"

*********************** Rank 1237 [Score  7.041600] GBROWSE*******************

insitu CG7337 in-situ | CG7337 | + | -14177 | 19393 | INTRAGENIC | intron:CG7337-RA:2 | CG7337-RA | "-"
 CG15357 in-situ | CG15357 | - | 21504 | 21220 | DOWNSTREAM | CG15357-RA | "-"

*********************** Rank 1238 [Score  7.041300] GBROWSE*******************

 CG6154 in-situ | CG6154 | + | -3651 | 3875 | INTRAGENIC | intron:CG6154-RA:1 | CG6154-RA | "-" | CG6154-RB | "-"
 CG14559 in-situ | CG14559 | + | 52045 | 65334 | UPSTREAM | CG14559-RA | "-"

*********************** Rank 1239 [Score  7.041100] GBROWSE*******************

 CG4525 in-situ | CG4525 | + | -585 | 2682 | INTRAGENIC | intron:CG4525-RA:2 | CG4525-RA | "-"
 CG5614 in-situ | CG5614 | + | 4845 | 6026 | UPSTREAM | CG5614-RA | "-"

*********************** Rank 1240 [Score  7.041000] GBROWSE*******************

 CG32406 in-situ | CG32406 | + | -1978 | 27018 | INTRAGENIC | intron:CG32406-RA:1 | CG32406-RA | "-"
 CG10478 in-situ | CG10478 | - | 11759 | 10364 | DOWNSTREAM | CG10478-RA | "-"

*********************** Rank 1241 [Score  7.040900] GBROWSE*******************

 CG16918 in-situ | CG16918 | + | -8109 | -3375 | DOWNSTREAM | CG16918-RA | "-"
 CG11719 in-situ | Mst98Ca | + | 1521 | 2851 | UPSTREAM | CG11719-RA | "-"

*********************** Rank 1242 [Score  7.038800] GBROWSE*******************

 CG10013 in-situ | CG10013 | + | -24586 | -22895 | DOWNSTREAM | CG10013-RA | "-"
 CG10038 in-situ | CG10038 | - | 2536 | 875 | DOWNSTREAM | CG10038-RB | "-" | CG10038-RA | "-"

*********************** Rank 1243 [Score  7.038600] GBROWSE*******************

 CG6013 in-situ | CG6013 | + | -5732 | -4845 | DOWNSTREAM | CG6013-RA | "-"
 CG6026 in-situ | CG6026 | + | 2290 | 6982 | UPSTREAM | CG6026-RA | "-"

*********************** Rank 1244 [Score  7.037700] GBROWSE*******************

 CG12540 in-situ | CG12540 | + | -32049 | -30944 | DOWNSTREAM | CG12540-RA | "-"
insitu CG14414 in-situ | CG14414 | + | 52422 | 53988 | UPSTREAM | CG14414-RA | "-" | CG14414-RC | "-" | CG14414-RB | "-"

*********************** Rank 1245 [Score  7.037700] GBROWSE*******************

insitu highlight CG2851 in-situ | Gsc | - | -2348 | -13495 | UPSTREAM | CG2851-RA | "-"
 CG13689 in-situ | CG13689 | + | 6077 | 6304 | UPSTREAM | CG13689-RA | "-"

*********************** Rank 1246 [Score  7.037700] GBROWSE*******************

 CG11456 in-situ | CG11456 | + | -12023 | -9684 | DOWNSTREAM | CG11456-RA | "-"
insitu CG32432 in-situ | CG32432 | - | 31164 | -9699 | INTRAGENIC | intron:CG32432-RA:8 | CG32432-RA | "-"

*********************** Rank 1247 [Score  7.035200] GBROWSE*******************

 CG12650 in-situ | CG12650 | + | -27424 | -20129 | DOWNSTREAM | CG12650-RB | "-"
 CG15316 in-situ | CG15316 | - | 61005 | -17383 | INTRAGENIC | intron:CG15316-RB:2 | intron:CG15316-RA:2 | CG15316-RB | "-" | CG15316-RA | "-"

*********************** Rank 1248 [Score  7.033400] GBROWSE*******************

 CG3886 in-situ | Psc | - | -5947 | -20626 | UPSTREAM | CG3886-RA | "-"
 CG3905 in-situ | Su(z)2 | + | 10187 | 20878 | UPSTREAM | CG3905-RA | "-"

*********************** Rank 1249 [Score  7.031000] GBROWSE*******************

 CG11368 in-situ | CG11368 | + | -34715 | -34260 | DOWNSTREAM | CG11368-RA | "-"
 CG32719 in-situ | CG32719 | - | 25920 | 21968 | DOWNSTREAM | CG32719-RA | "-"

*********************** Rank 1250 [Score  7.031000] GBROWSE*******************

 CG10317 in-situ | CG10317 | - | -22427 | -23474 | UPSTREAM | CG10317-RA | "-"
insitu CG6993 in-situ | ss | - | 10723 | -18532 | INTRAGENIC | intron:CG6993-RA:2 | CG6993-RA | "-"

*********************** Rank 1251 [Score  7.030600] GBROWSE*******************

 CG14992 in-situ | Ack | + | -8669 | -2713 | DOWNSTREAM | CG14992-RA | "-"
 CG14996 in-situ | Chd64 | - | 5549 | -2585 | INTRAGENIC | intron:CG14996-RB:1 | CG14996-RB | "-"

*********************** Rank 1252 [Score  7.028600] GBROWSE*******************

 CG6304 in-situ | CG6304 | - | -3162 | -4669 | UPSTREAM | CG6304-RA | "-"
 CG15136 in-situ | CG15136 | - | 31765 | 31032 | DOWNSTREAM | CG15136-RA | "-"

*********************** Rank 1253 [Score  7.028300] GBROWSE*******************

 CG8127 in-situ | Eip75B | - | -23973 | -131608 | UPSTREAM | CG8127-RB | "-" | CG8127-RC | "-" | CG8127-RA | "-" | CG8127-RD | "-"
insitu CG32194 in-situ | CG32194 | - | 3487 | 1733 | DOWNSTREAM | CG32194-RB | "-"

*********************** Rank 1254 [Score  7.027700] GBROWSE*******************

insitu CG9876 in-situ | CG9876 | - | -5320 | -9479 | UPSTREAM | CG9876-RA | "-"
 CG9873 in-situ | CG9873 | - | 791 | 522 | DOWNSTREAM | CG9873-RA | "-"

*********************** Rank 1255 [Score  7.027600] GBROWSE*******************

 CG31394 in-situ | CG31394 | - | -42404 | -43142 | UPSTREAM | CG31394-RA | "-"
insitu highlight CG17117 in-situ | hth | - | 67104 | -61720 | INTRAGENIC | intron:CG17117-RB:6 | intron:CG17117-RC:6 | intron:CG17117-RA:5 | CG17117-RD | "-" | CG17117-RB | "-" | CG17117-RC | "-" | CG17117-RA | "-"

*********************** Rank 1256 [Score  7.027000] GBROWSE*******************

insitu CG6738 in-situ | CG6738 | + | -8984 | -7539 | DOWNSTREAM | CG6738-RA | "-"
 CG13830 in-situ | CG13830 | - | 4193 | -35900 | INTRAGENIC | intron:CG13830-RA:1 | CG13830-RA | "-"

*********************** Rank 1257 [Score  7.026700] GBROWSE*******************

 CG32594 in-situ | CG32594 | + | -22152 | 5254 | INTRAGENIC | intron:CG32594-RA:6 | intron:CG32594-RD:1 | CG32594-RA | "-" | CG32594-RB | "-" | CG32594-RC | "-" | CG32594-RD | "-" | CG32594-RE | "-"
 CG32592 in-situ | hiw | - | 16821 | -35903 | INTRAGENIC | intron:CG32592-RA:13 | CG32592-RA | "-"

*********************** Rank 1258 [Score  7.026600] GBROWSE*******************

 CG12637 in-situ | CG12637 | - | -3822 | -4700 | UPSTREAM | CG12637-RA | "-"
 CG32677 in-situ | CG32677 | + | 25264 | 32489 | UPSTREAM | CG32677-RA | "-"

*********************** Rank 1259 [Score  7.025300] GBROWSE*******************

 CG17362 in-situ | CG17362 | + | -1424 | -254 | DOWNSTREAM | CG17362-RA | "-"
 CG9040 in-situ | CG9040 | + | 529 | 1256 | UPSTREAM | CG9040-RA | "-"

*********************** Rank 1260 [Score  7.024500] GBROWSE*******************

 CG8183 in-situ | Khc-73 | - | -387 | -16847 | UPSTREAM | CG8183-RB | "-" | CG8183-RA | "-"
 CG30471 in-situ | CG30471 | + | 2570 | 5025 | UPSTREAM | CG30471-RA | "-"

*********************** Rank 1261 [Score  7.023700] GBROWSE*******************

insitu CG5799 in-situ | dve | + | -2636 | 39819 | INTRAGENIC | intron:CG5799-RA:1 | intron:CG5799-RD:1 | intron:CG5799-RB:1 | intron:CG5799-RC:1 | CG5799-RA | "-" | CG5799-RD | "-" | CG5799-RB | "-" | CG5799-RC | "-"
insitu CG5819 in-situ | CG5819 | + | 46860 | 50359 | UPSTREAM | CG5819-RA | "-" | CG5819-RB | "-"

*********************** Rank 1262 [Score  7.023100] GBROWSE*******************

 CG13783 in-situ | CG13783 | - | -11722 | -12962 | UPSTREAM | CG13783-RA | "-"
 CG4495 in-situ | CG4495 | + | 13817 | 17111 | UPSTREAM | CG4495-RA | "-"

*********************** Rank 1263 [Score  7.022800] GBROWSE*******************

 CG31909 in-situ | CG31909 | + | -15248 | -14704 | DOWNSTREAM | CG31909-RA | "-"
insitu CG4698 in-situ | Wnt4 | - | 4349 | -17359 | INTRAGENIC | intron:CG4698-RA:1 | CG4698-RA | "-"

*********************** Rank 1264 [Score  7.022600] GBROWSE*******************

 CG31483 in-situ | Pif2 | + | -7691 | -6807 | DOWNSTREAM | CG31483-RA | "-"
 CG1137 in-situ | CG1137 | - | 3624 | 1888 | DOWNSTREAM | CG1137-RA | "-"

*********************** Rank 1265 [Score  7.020200] GBROWSE*******************

 CG15465 in-situ | CG15465 | + | -1896 | -628 | DOWNSTREAM | CG15465-RA | "-"
 CG5062 in-situ | CG5062 | - | 5233 | 3045 | DOWNSTREAM | CG5062-RA | "-"

*********************** Rank 1266 [Score  7.019000] GBROWSE*******************

 CG31531 in-situ | CG31531 | + | -17146 | 28528 | INTRAGENIC | intron:CG31531-RA:3 | intron:CG31531-RC:3 | intron:CG31531-RB:2 | CG31531-RA | "-" | CG31531-RC | "-" | CG31531-RB | "-"
insitu CG31534 in-situ | CG31534 | + | 29417 | 35464 | UPSTREAM | CG31534-RA | "-" | CG31534-RB | "-"

*********************** Rank 1267 [Score  7.017800] GBROWSE*******************

 CG31807 in-situ | CG31807 | - | -9250 | -9717 | UPSTREAM | CG31807-RA | "-"
 CG13263 in-situ | Cyt-c-d | + | 24242 | 28026 | UPSTREAM | CG13263-RA | "-"

*********************** Rank 1268 [Score  7.016000] GBROWSE*******************

 CG2617 in-situ | CG2617 | + | -26988 | -25726 | DOWNSTREAM | CG2617-RA | "-"
 CG1864 in-situ | Hr38 | - | 5231 | -25898 | INTRAGENIC | intron:CG1864-RB:1 | CG1864-RB | "-" | CG1864-RC | "-"

*********************** Rank 1269 [Score  7.015400] GBROWSE*******************

 CG10160 in-situ | ImpL3 | - | -2228 | -5432 | UPSTREAM | CG10160-RA | "-"
 CG32401 in-situ | CG32401 | + | 55690 | 57177 | UPSTREAM | CG32401-RA | "-"

*********************** Rank 1270 [Score  7.014900] GBROWSE*******************

insitu CG12926 in-situ | CG12926 | - | -64084 | -66241 | UPSTREAM | CG12926-RA | "-"
 CG1794 in-situ | Mmp2 | - | 12297 | -62276 | INTRAGENIC | intron:CG1794-RA:2 | CG1794-RA | "-"

*********************** Rank 1271 [Score  7.014600] GBROWSE*******************

 CG32446 in-situ | CG32446 | + | -330 | 1227 | INTRAGENIC | intron:CG32446-RA:2 | CG32446-RA | "-"
 CG11250 in-situ | CG11250 | + | 3588 | 24648 | UPSTREAM | CG11250-RB | "-" | CG11250-RA | "-"

*********************** Rank 1272 [Score  7.014600] GBROWSE*******************

 CG32767 in-situ | CG32767 | - | -6863 | -13302 | UPSTREAM | CG32767-RA | "-"
 CG6789 in-situ | CG6789 | + | 43962 | 45053 | UPSTREAM | CG6789-RA | "-"

*********************** Rank 1273 [Score  7.013900] GBROWSE*******************

insitu highlight CG7952 in-situ | gt | - | -7965 | -9821 | UPSTREAM | CG7952-RB | "-"
 CG7925 in-situ | tko | - | 7048 | 5379 | DOWNSTREAM | CG7925-RB | "-"

*********************** Rank 1274 [Score  7.013600] GBROWSE*******************

 CG11608 in-situ | CG11608 | + | -1778 | -312 | DOWNSTREAM | CG11608-RA | "-"
 CG6753 in-situ | CG6753 | - | 2314 | 82 | DOWNSTREAM | CG6753-RA | "-"

*********************** Rank 1275 [Score  7.012500] GBROWSE*******************

insitu highlight CG5461 in-situ | bun | - | -31959 | -115952 | UPSTREAM | CG5461-RA | "-" | CG5461-RB | "-" | CG5461-RC | "-"
 CG15489 in-situ | CG15489 | + | 21258 | 22160 | UPSTREAM | CG15489-RA | "-"

*********************** Rank 1276 [Score  7.011000] GBROWSE*******************

 CG14853 in-situ | CG14853 | + | -6911 | 2427 | INTRAGENIC | intron:CG14853-RB:3 | intron:CG14853-RA:3 | CG14853-RB | "-" | CG14853-RA | "-"
 CG7904 in-situ | put | - | 9139 | 3764 | DOWNSTREAM | CG7904-RA | "-"


*********************** Rank 1277 [Score  7.010900] GBROWSE*******************

 CG18371 in-situ | CG18371 | + | -32318 | -31830 | DOWNSTREAM | CG18371-RA | "-"
 CG13353 in-situ | CG13353 | + | 29440 | 30791 | UPSTREAM | CG13353-RA | "-"

*********************** Rank 1278 [Score  7.009600] GBROWSE*******************

 CG7847 in-situ | sr | + | -35460 | 7486 | INTRAGENIC | intron:CG7847-RA:3 | intron:CG7847-RB:1 | CG7847-RA | "-" | CG7847-RB | "-"
 CG14316 in-situ | CG14316 | + | 25857 | 27379 | UPSTREAM | CG14316-RA | "-"

*********************** Rank 1279 [Score  7.009500] GBROWSE*******************

 CG14436 in-situ | CG14436 | - | -1873 | -2343 | UPSTREAM | CG14436-RA | "-"
 CG3126 in-situ | C3G | - | 1422 | -20727 | INTRAGENIC | intron:CG3126-RA:1 | intron:CG3126-RB:1 | CG3126-RA | "-" | CG3126-RB | "-"

*********************** Rank 1280 [Score  7.008700] GBROWSE*******************

 CG4481 in-situ | Glu-RIB | + | -28966 | 4071 | INTRAGENIC | intron:CG4481-RA:13 | CG4481-RA | "-"
 CG32042 in-situ | PGRP-LA | + | 52943 | 56548 | UPSTREAM | CG32042-RB | "-" | CG32042-RA | "-"

*********************** Rank 1281 [Score  7.006600] GBROWSE*******************

 CG2113 in-situ | CG2113 | + | -5391 | -4413 | DOWNSTREAM | CG2113-RA | "-"
 CG2114 in-situ | FR | + | 8175 | 9824 | UPSTREAM | CG2114-RA | "-"

*********************** Rank 1282 [Score  7.006300] GBROWSE*******************

 CG3837 in-situ | CG3837 | + | -79280 | -75655 | DOWNSTREAM | CG3837-RA | "-"
 CG14861 in-situ | CG14861 | + | 16810 | 18077 | UPSTREAM | CG14861-RA | "-"


*********************** Rank 1283 [Score  7.006100] GBROWSE*******************

 CG4285 in-situ | CG4285 | + | -9859 | -4893 | DOWNSTREAM | CG4285-RA | "-"
insitu CG6476 in-situ | Su(var)3-9 | - | 1514 | -4026 | INTRAGENIC | intron:CG6476-RA:2 | intron:CG6476-RB:2 | intron:CG6476-RC:2 | CG6476-RA | "-" | CG6476-RB | "-" | CG6476-RC | "-"

*********************** Rank 1284 [Score  7.005200] GBROWSE*******************

insitu CG2083 in-situ | CG2083 | - | -8841 | -20849 | UPSTREAM | CG2083-RA | "-"
 CG14952 in-situ | CG14952 | - | 38924 | 38541 | DOWNSTREAM | CG14952-RA | "-"

*********************** Rank 1285 [Score  7.005200] GBROWSE*******************

 CG9266 in-situ | CG9266 | - | -43365 | -46789 | UPSTREAM | CG9266-RB | "-"
insitu CG1762 in-situ | betaInt-nu | + | 40526 | 45738 | UPSTREAM | CG1762-RA | "-"

*********************** Rank 1286 [Score  7.004600] GBROWSE*******************

 CG14910 in-situ | CG14910 | + | -12205 | -11702 | DOWNSTREAM | CG14910-RA | "-"
 CG14911 in-situ | CG14911 | + | 10182 | 11185 | UPSTREAM | CG14911-RA | "-"

*********************** Rank 1287 [Score  7.003700] GBROWSE*******************

 CG31887 in-situ | CG31887 | - | -18476 | -19068 | UPSTREAM | CG31887-RA | "-"
 CG13108 in-situ | CG13108 | + | 15916 | 17106 | UPSTREAM | CG13108-RA | "-"

*********************** Rank 1288 [Score  7.002700] GBROWSE*******************

 CG14925 in-situ | CG14925 | - | -51526 | -52543 | UPSTREAM | CG14925-RA | "-"
 CG14926 in-situ | CG14926 | - | 6134 | 5184 | DOWNSTREAM | CG14926-RA | "-"

*********************** Rank 1289 [Score  7.002700] GBROWSE*******************

 CG15286 in-situ | BG:DS01068.11 | + | -5558 | -3718 | DOWNSTREAM | CG15286-RA | "-"
 CG18125 in-situ | BG:DS01068.10 | - | 2856 | 2137 | DOWNSTREAM | CG18125-RA | "-"

*********************** Rank 1290 [Score  7.002300] GBROWSE*******************

 CG1004 in-situ | rho | + | -5479 | -788 | DOWNSTREAM | CG1004-RA | "-"
 CG32319 in-situ | CG32319 | - | 2789 | 1997 | DOWNSTREAM | CG32319-RA | "-"


*********************** Rank 1291 [Score  7.001200] GBROWSE*******************

insitu CG18783 in-situ | Kr-h1 | + | -11927 | 2769 | INTRAGENIC | intron:CG18783-RA:2 | intron:CG18783-RB:2 | CG18783-RA | "-" | CG18783-RB | "-"
insitu CG9175 in-situ | CG9175 | - | 5229 | 2960 | DOWNSTREAM | CG9175-RA | "-" | CG9175-RB | "-"

*********************** Rank 1292 [Score  7.000200] GBROWSE*******************

 CG3578 in-situ | bi | + | -37378 | 34230 | INTRAGENIC | intron:CG3578-RA:2 | CG3578-RA | "-"
 CG12685 in-situ | CG12685 | + | 65380 | 65948 | UPSTREAM | CG12685-RA | "-"

*********************** Rank 1293 [Score  6.999400] GBROWSE*******************

 CG8524 in-situ | NK7.1 | - | -5508 | -48335 | UPSTREAM | CG8524-RA | "-" | CG8524-RB | "-"
 CG8489 in-situ | CG8489 | - | 3240 | 1924 | DOWNSTREAM | CG8489-RA | "-"

*********************** Rank 1294 [Score  6.999300] GBROWSE*******************

 CG32397 in-situ | CG32397 | - | -14008 | -23628 | UPSTREAM | CG32397-RA | "-"
 CG10129 in-situ | ndl | - | 16295 | 6867 | DOWNSTREAM | CG10129-RA | "-"

*********************** Rank 1295 [Score  6.998700] GBROWSE*******************

 CG12688 in-situ | CG12688 | + | -962 | 735 | INTRAGENIC | intron:CG12688-RA:2 | CG12688-RA | "-"
 CG32773 in-situ | CG32773 | - | 5817 | 5398 | DOWNSTREAM | CG32773-RA | "-"

*********************** Rank 1296 [Score  6.995300] GBROWSE*******************

insitu highlight CG31043 in-situ | gukh | + | -28586 | 9827 | INTRAGENIC | intron:CG31043-RA:3 | intron:CG31043-RB:3 | CG31043-RA | "-" | CG31043-RB | "-"
 CG6005 in-situ | CG6005 | - | 11411 | 9990 | DOWNSTREAM | CG6005-RA | "-"

*********************** Rank 1297 [Score  6.994700] GBROWSE*******************

 CG31912 in-situ | CG31912 | + | -10033 | -8846 | DOWNSTREAM | CG31912-RA | "-"
 CG31913 in-situ | CG31913 | + | 4246 | 5108 | UPSTREAM | CG31913-RA | "-"

*********************** Rank 1298 [Score  6.993700] GBROWSE*******************

 CG12964 in-situ | CG12964 | - | -7565 | -11414 | UPSTREAM | CG12964-RA | "-"
 CG12960 in-situ | CG12960 | + | 2281 | 3756 | UPSTREAM | CG12960-RA | "-"

*********************** Rank 1299 [Score  6.993200] GBROWSE*******************

 CG12535 in-situ | CG12535 | - | -15948 | -16733 | UPSTREAM | CG12535-RB | "-" | CG12535-RA | "-"
 CG14269 in-situ | CG14269 | - | 26405 | 25726 | DOWNSTREAM | CG14269-RA | "-"

*********************** Rank 1300 [Score  6.991900] GBROWSE*******************

 CG6103 in-situ | CrebB-17A | + | -5308 | -1390 | DOWNSTREAM | CG6103-RA | "-" | CG6103-RB | "-"
insitu CG6179 in-situ | CG6179 | + | 2109 | 4048 | UPSTREAM | CG6179-RA | "-"

*********************** Rank 1301 [Score  6.991300] GBROWSE*******************

 CG11041 in-situ | CG11041 | + | -47891 | -46809 | DOWNSTREAM | CG11041-RA | "-"
 CG18416 in-situ | CG18416 | + | 9986 | 10672 | UPSTREAM | CG18416-RA | "-"

*********************** Rank 1302 [Score  6.989900] GBROWSE*******************

insitu highlight CG2189 in-situ | Dfd | + | -36191 | -25596 | DOWNSTREAM | CG2189-RA | "-"
insitu highlight CG1030 in-situ | Scr | - | 20592 | -4907 | INTRAGENIC | intron:CG1030-RA:2 | CG1030-RA | "-"

*********************** Rank 1303 [Score  6.989900] GBROWSE*******************

insitu CG6305 in-situ | CG6305 | + | -4133 | 6300 | INTRAGENIC | intron:CG6305-RA:2 | CG6305-RA | "-"
 CG12295 in-situ | CG12295 | + | 6538 | 20639 | UPSTREAM | CG12295-RB | "-"

*********************** Rank 1304 [Score  6.989700] GBROWSE*******************

 CG8279 in-situ | CG8279 | - | -17130 | -33069 | UPSTREAM | CG8279-RA | "-"
 CG14854 in-situ | CG14854 | - | 14011 | 12301 | DOWNSTREAM | CG14854-RA | "-"

*********************** Rank 1305 [Score  6.988200] GBROWSE*******************

 CG3510 in-situ | CycB | - | -1715 | -5178 | UPSTREAM | CG3510-RA | "-" | CG3510-RD | "-" | CG3510-RB | "-" | CG3510-RC | "-"
 CG3622 in-situ | CG3622 | + | 2692 | 7303 | UPSTREAM | CG3622-RB | "-" | CG3622-RA | "-"

*********************** Rank 1306 [Score  6.986000] GBROWSE*******************

 CG11041 in-situ | CG11041 | + | -46391 | -45309 | DOWNSTREAM | CG11041-RA | "-"
 CG18416 in-situ | CG18416 | + | 11486 | 12172 | UPSTREAM | CG18416-RA | "-"


*********************** Rank 1307 [Score  6.985000] GBROWSE*******************

 CG14239 in-situ | CG14239 | - | -1875 | -2754 | UPSTREAM | CG14239-RA | "-"
 CG5432 in-situ | CG5432 | + | 9425 | 10726 | UPSTREAM | CG5432-RA | "-"

*********************** Rank 1308 [Score  6.985000] GBROWSE*******************

 CG17572 in-situ | CG17572 | - | -221 | -3175 | UPSTREAM | CG17572-RA | "-"
 CG10700 in-situ | CG10700 | - | 4628 | 3009 | DOWNSTREAM | CG10700-RA | "-"

*********************** Rank 1309 [Score  6.984700] GBROWSE*******************

 CG15631 in-situ | CG15631 | - | -14873 | -16922 | UPSTREAM | CG15631-RA | "-"
 CG15630 in-situ | CG15630 | - | 44711 | -13784 | INTRAGENIC | intron:CG15630-RA:1 | CG15630-RA | "-"

*********************** Rank 1310 [Score  6.984300] GBROWSE*******************

insitu CG4623 in-situ | CG4623 | + | -486 | 1878 | INTRAGENIC | intron:CG4623-RA:1 | CG4623-RA | "-"
insitu CG4633 in-situ | Aats-ala-m | + | 3664 | 7413 | UPSTREAM | CG4633-RA | "-"

*********************** Rank 1311 [Score  6.983900] GBROWSE*******************

 CG12662 in-situ | CG12662 | - | -31782 | -32297 | UPSTREAM | CG12662-RA | "-"
 CG32709 in-situ | CG32709 | + | 10250 | 11669 | UPSTREAM | CG32709-RA | "-"

*********************** Rank 1312 [Score  6.983800] GBROWSE*******************

 CG32206 in-situ | CG32206 | - | -14879 | -87450 | UPSTREAM | CG32206-RB | "-" | CG32206-RC | "-"
 CG33062 in-situ | CG33062 | - | 18612 | 14327 | DOWNSTREAM | CG33062-RA | "-"

*********************** Rank 1313 [Score  6.983600] GBROWSE*******************

 CG5308 in-situ | CG5308 | + | -28176 | -22205 | DOWNSTREAM | CG5308-RA | "-" | CG5308-RB | "-"
 CG12593 in-situ | CG12593 | - | 8735 | 6340 | DOWNSTREAM | CG12593-RA | "-"

*********************** Rank 1314 [Score  6.983200] GBROWSE*******************

 CG5194 in-situ | CG5194 | - | -4239 | -5466 | UPSTREAM | CG5194-RA | "-"
insitu highlight CG5187 in-situ | Doc2 | - | 5150 | -1405 | INTRAGENIC | intron:CG5187-RA:4 | CG5187-RA | "-"

*********************** Rank 1315 [Score  6.979900] GBROWSE*******************

 CG8985 in-situ | CG8985 | + | -9033 | -3470 | DOWNSTREAM | CG8985-RA | "-"
 CG13801 in-situ | CG13801 | + | 12745 | 14817 | UPSTREAM | CG13801-RA | "-"

*********************** Rank 1316 [Score  6.979900] GBROWSE*******************

 CG11958 in-situ | Cnx99A | - | -20368 | -24761 | UPSTREAM | CG11958-RA | "-" | CG11958-RB | "-"
 CG11516 in-situ | CG11516 | + | 86690 | 88743 | UPSTREAM | CG11516-RA | "-"

*********************** Rank 1317 [Score  6.978900] GBROWSE*******************

 CG32698 in-situ | CG32698 | + | -62984 | -1325 | DOWNSTREAM | CG32698-RA | "-"
 CG2045 in-situ | Ser7 | + | 14663 | 16515 | UPSTREAM | CG2045-RA | "-"

*********************** Rank 1318 [Score  6.978600] GBROWSE*******************

 CG12656 in-situ | CG12656 | + | -10686 | -10068 | DOWNSTREAM | CG12656-RA | "-"
 CG15322 in-situ | CG15322 | + | 1855 | 5231 | UPSTREAM | CG15322-RA | "-"

*********************** Rank 1319 [Score  6.978500] GBROWSE*******************

 CG6486 in-situ | CG6486 | + | -14916 | -13724 | DOWNSTREAM | CG6486-RA | "-"
insitu highlight CG6494 in-situ | h | + | 11519 | 14799 | UPSTREAM | CG6494-RA | "-"

note: overlaps known module h_rescue by 500 bases (module coords: 8620516-8642090)
note: overlaps known module h_stripe3_4_1991 by 399 bases (module coords: 8622071-8623548)
note: overlaps known module h_stripe3_4_1990 by 500 bases (module coords: 8622240-8623984)
note: overlaps known module h_stripe3 by 399 bases (module coords: 8623073-8623548)
note: overlaps known module h_stripe4 by 500 bases (module coords: 8623073-8623984)
note: overlaps known module h_stripe7_1990a by 102 bases (module coords: 8623548-8625021)

*********************** Rank 1320 [Score  6.977700] GBROWSE*******************

 CG14521 in-situ | CG14521 | - | -6008 | -51367 | UPSTREAM | CG14521-RA | "-"
 CG14520 in-situ | CG14520 | + | 1556 | 2153 | UPSTREAM | CG14520-RA | "-"

*********************** Rank 1321 [Score  6.975600] GBROWSE*******************

 CG11041 in-situ | CG11041 | + | -29241 | -28159 | DOWNSTREAM | CG11041-RA | "-"
 CG18416 in-situ | CG18416 | + | 28636 | 29322 | UPSTREAM | CG18416-RA | "-"

*********************** Rank 1322 [Score  6.974600] GBROWSE*******************

 CG2944 in-situ | CG2944 | - | -14868 | -25970 | UPSTREAM | CG2944-RB | "-" | CG2944-RA | "-" | CG2944-RC | "-" | CG2944-RD | "-" | CG2944-RE | "-" | CG2944-RF | "-"
insitu highlight CG3136 in-situ | CG3136 | + | 1517 | 11581 | UPSTREAM | CG3136-RA | "-" | CG3136-RB | "-"

*********************** Rank 1323 [Score  6.972700] GBROWSE*******************

insitu CG3127 in-situ | Pgk | - | -1859 | -3501 | UPSTREAM | CG3127-RA | "-"
insitu CG9894 in-situ | CG9894 | + | 2418 | 6225 | UPSTREAM | CG9894-RB | "-" | CG9894-RA | "-"

*********************** Rank 1324 [Score  6.972700] GBROWSE*******************

 CG4841 in-situ | CG4841 | + | -54388 | -48014 | DOWNSTREAM | CG4841-RA | "-"
 CG33179 in-situ | beat-IIIb | + | 19589 | 27976 | UPSTREAM | CG33179-RA | "-"

*********************** Rank 1325 [Score  6.970700] GBROWSE*******************

insitu CG3619 in-situ | Dl | - | -15898 | -39380 | UPSTREAM | CG3619-RA | "-" | CG3619-RB | "-"
 CG3581 in-situ | CG3581 | - | 32638 | 31646 | DOWNSTREAM | CG3581-RA | "-"


*********************** Rank 1326 [Score  6.970500] GBROWSE*******************

 CG7886 in-situ | CG7886 | - | -8747 | -24637 | UPSTREAM | CG7886-RA | "-"
 CG7832 in-situ | BEST:LD14744 | - | 4391 | -6641 | INTRAGENIC | intron:CG7832-RA:1 | CG7832-RA | "-"

*********************** Rank 1327 [Score  6.969000] GBROWSE*******************

 CG6641 in-situ | Pbprp5 | - | -11459 | -11952 | UPSTREAM | CG6641-RA | "-"
 CG6682 in-situ | Rapgap1 | - | 20577 | -11018 | INTRAGENIC | intron:CG6682-RA:1 | CG6682-RA | "-" | CG6682-RB | "-"

*********************** Rank 1328 [Score  6.969000] GBROWSE*******************

 CG9731 in-situ | CG9731 | - | -24931 | -25719 | UPSTREAM | CG9731-RA | "-"
insitu CG31349 in-situ | pyd | - | 43543 | -60235 | INTRAGENIC | intron:CG31349-RE:1 | CG31349-RE | "-" | CG31349-RA | "-" | CG31349-RC | "-" | CG31349-RB | "-" | CG31349-RF | "-"

*********************** Rank 1329 [Score  6.967900] GBROWSE*******************

insitu CG14356 in-situ | CG14356 | - | -5590 | -6981 | UPSTREAM | CG14356-RA | "-"
 CG14355 in-situ | CG14355 | - | 14340 | 10835 | DOWNSTREAM | CG14355-RA | "-"

*********************** Rank 1330 [Score  6.967800] GBROWSE*******************

 CG3199 in-situ | CG3199 | + | -6140 | -5234 | DOWNSTREAM | CG3199-RA | "-"
 CG9652 in-situ | DopR | - | 45828 | 8424 | DOWNSTREAM | CG9652-RA | "-"

*********************** Rank 1331 [Score  6.967300] GBROWSE*******************

 CG13003 in-situ | CG13003 | - | -5494 | -13925 | UPSTREAM | CG13003-RA | "-"
 CG4872 in-situ | CG4872 | + | 22640 | 24068 | UPSTREAM | CG4872-RA | "-"


*********************** Rank 1332 [Score  6.965800] GBROWSE*******************

 CG4054 in-situ | CG4054 | + | -12879 | -11738 | DOWNSTREAM | CG4054-RA | "-"
 CG13487 in-situ | CG13487 | + | 547 | 2077 | UPSTREAM | CG13487-RA | "-"

*********************** Rank 1333 [Score  6.965300] GBROWSE*******************

insitu CG6844 in-situ | nAcRalpha-96Ab | + | -9979 | -4268 | DOWNSTREAM | CG6844-RA | "-" | CG6844-RB | "-"
 CG6798 in-situ | nAcRbeta-96A | - | 19680 | 13793 | DOWNSTREAM | CG6798-RA | "-" | CG6798-RB | "-"

*********************** Rank 1334 [Score  6.964800] GBROWSE*******************

 CG17024 in-situ | CG17024 | + | -16997 | -15728 | DOWNSTREAM | CG17024-RA | "-"
 CG31858 in-situ | CG31858 | - | 26616 | 25966 | DOWNSTREAM | CG31858-RA | "-"

*********************** Rank 1335 [Score  6.963600] GBROWSE*******************

 CG9701 in-situ | CG9701 | + | -9196 | -3399 | DOWNSTREAM | CG9701-RA | "-"
 CG9704 in-situ | Nrt | + | 23304 | 34808 | UPSTREAM | CG9704-RB | "-" | CG9704-RA | "-"

*********************** Rank 1336 [Score  6.962700] GBROWSE*******************

 CG7492 in-situ | CG7492 | - | -4126 | -7005 | UPSTREAM | CG7492-RA | "-"
 CG32377 in-situ | CG32377 | - | 34984 | 7260 | DOWNSTREAM | CG32377-RA | "-"

*********************** Rank 1337 [Score  6.961200] GBROWSE*******************

 CG9138 in-situ | SP1070 | - | -14056 | -25539 | UPSTREAM | CG9138-RA | "-"
 CG13776 in-situ | CG13776 | + | 14427 | 15576 | UPSTREAM | CG13776-RA | "-"

*********************** Rank 1338 [Score  6.961100] GBROWSE*******************

 CG5465 in-situ | Trap95 | + | -2803 | -57 | DOWNSTREAM | CG5465-RA | "-"
 CG5625 in-situ | CG5625 | + | 29 | 3938 | UPSTREAM | CG5625-RB | "-" | CG5625-RA | "-"

*********************** Rank 1339 [Score  6.953400] GBROWSE*******************

 CG4881 in-situ | salr | + | -55324 | -47232 | DOWNSTREAM | CG4881-RA | "-" | CG4881-RB | "-"
insitu highlight CG6464 in-situ | salm | - | 24974 | 13682 | DOWNSTREAM | CG6464-RA | "-"

*********************** Rank 1340 [Score  6.952800] GBROWSE*******************

 CG12283 in-situ | kek1 | - | -32243 | -36100 | UPSTREAM | CG12283-RA | "-"
 CG5983 in-situ | ACXC | + | 57500 | 61744 | UPSTREAM | CG5983-RA | "-"

*********************** Rank 1341 [Score  6.950400] GBROWSE*******************

 CG31880 in-situ | CG31880 | + | -7601 | -6826 | DOWNSTREAM | CG31880-RA | "-"
 CG4128 in-situ | nAcRalpha-30D | - | 44093 | -45149 | INTRAGENIC | intron:CG4128-RA:2 | intron:CG4128-RC:2 | CG4128-RA | "-" | CG4128-RC | "-"

*********************** Rank 1342 [Score  6.950000] GBROWSE*******************

 CG3541 in-situ | CG3541 | + | -6760 | 9841 | INTRAGENIC | intron:CG3541-RB:2 | intron:CG3541-RC:2 | CG3541-RB | "-" | CG3541-RC | "-"
 CG13587 in-situ | CG13587 | + | 9963 | 11059 | UPSTREAM | CG13587-RA | "-"


*********************** Rank 1343 [Score  6.949700] GBROWSE*******************

insitu highlight CG17943 in-situ | comm | - | -14798 | -20897 | UPSTREAM | CG17943-RA | "-"
 CG13445 in-situ | CG13445 | + | 66277 | 66662 | UPSTREAM | CG13445-RA | "-"

*********************** Rank 1344 [Score  6.947900] GBROWSE*******************

 CG8298 in-situ | CG8298 | + | -15454 | -13146 | DOWNSTREAM | CG8298-RA | "-" | CG8298-RB | "-"
 CG8967 in-situ | otk | - | 15237 | -3109 | INTRAGENIC | intron:CG8967-RA:4 | CG8967-RA | "-"

*********************** Rank 1345 [Score  6.947800] GBROWSE*******************

 CG18553 in-situ | CG18553 | - | -11005 | -11907 | UPSTREAM | CG18553-RA | "-"
 CG31355 in-situ | CG31355 | + | 49655 | 50811 | UPSTREAM | CG31355-RA | "-"

*********************** Rank 1346 [Score  6.947700] GBROWSE*******************

 CG13251 in-situ | CG13251 | + | -47482 | -43917 | DOWNSTREAM | CG13251-RA | "-"
 CG32431 in-situ | CG32431 | + | 3030 | 3737 | UPSTREAM | CG32431-RA | "-"

*********************** Rank 1347 [Score  6.947600] GBROWSE*******************

 CG12814 in-situ | CG12814 | - | -7438 | -9993 | UPSTREAM | CG12814-RA | "-"
 CG3985 in-situ | Syn | + | 8428 | 39823 | UPSTREAM | CG3985-RB | "-" | CG3985-RD | "-" | CG3985-RE | "-" | CG3985-RF | "-" | CG3985-RC | "-"

*********************** Rank 1348 [Score  6.947000] GBROWSE*******************

 CG15186 in-situ | CG15186 | + | -35331 | -23094 | DOWNSTREAM | CG15186-RA | "-" | CG15186-RB | "-"
 CG15185 in-situ | CG15185 | + | 9402 | 10430 | UPSTREAM | CG15185-RA | "-"

*********************** Rank 1349 [Score  6.946900] GBROWSE*******************

 CG7320 in-situ | CG7320 | + | -10932 | -9205 | DOWNSTREAM | CG7320-RA | "-"
 CG7313 in-situ | CG7313 | + | 39326 | 40075 | UPSTREAM | CG7313-RA | "-"

*********************** Rank 1350 [Score  6.946900] GBROWSE*******************

 CG30442 in-situ | CG30442 | - | -3827 | -5136 | UPSTREAM | CG30442-RB | "-"
 CG2944 in-situ | CG2944 | - | 27732 | 16630 | DOWNSTREAM | CG2944-RB | "-" | CG2944-RA | "-" | CG2944-RC | "-" | CG2944-RD | "-" | CG2944-RE | "-" | CG2944-RF | "-"


*********************** Rank 1351 [Score  6.945800] GBROWSE*******************

 CG12217 in-situ | PpV | - | -39133 | -40915 | UPSTREAM | CG12217-RA | "-"
 CG3367 in-situ | CG3367 | - | 56854 | 55060 | DOWNSTREAM | CG3367-RA | "-"

*********************** Rank 1352 [Score  6.945700] GBROWSE*******************

 CG1867 in-situ | Or98b | + | -50520 | -49142 | DOWNSTREAM | CG1867-RA | "-"
 CG14064 in-situ | beat-VI | + | 15442 | 70447 | UPSTREAM | CG14064-RA | "-"

*********************** Rank 1353 [Score  6.945600] GBROWSE*******************

 CG3040 in-situ | CG3040 | - | -114 | -1200 | UPSTREAM | CG3040-RA | "-"
insitu CG3039 in-situ | ogre | - | 8174 | 492 | DOWNSTREAM | CG3039-RA | "-" | CG3039-RB | "-"

*********************** Rank 1354 [Score  6.945200] GBROWSE*******************

 CG30076 in-situ | CG30076 | - | -24115 | -25437 | UPSTREAM | CG30076-RA | "-"
 CG10119 in-situ | LamC | - | 5928 | 930 | DOWNSTREAM | CG10119-RA | "-"


*********************** Rank 1355 [Score  6.944600] GBROWSE*******************

 CG14141 in-situ | CG14141 | + | -4012 | -3168 | DOWNSTREAM | CG14141-RA | "-"
 CG6199 in-situ | CG6199 | - | 728 | -7152 | INTRAGENIC | intron:CG6199-RA:1 | intron:CG6199-RB:2 | CG6199-RA | "-" | CG6199-RB | "-"

*********************** Rank 1356 [Score  6.944200] GBROWSE*******************

 CG10293 in-situ | how | + | -32922 | 4213 | INTRAGENIC | intron:CG10293-RA:8 | CG10293-RA | "-" | CG10293-RB | "-"
 CG13408 in-situ | CG13408 | + | 5348 | 8024 | UPSTREAM | CG13408-RA | "-"

*********************** Rank 1357 [Score  6.940000] GBROWSE*******************

insitu highlight CG4029 in-situ | jumu | + | -11476 | -39 | DOWNSTREAM | CG4029-RA | "-"
 CG6312 in-situ | Rfx | - | 16408 | 851 | DOWNSTREAM | CG6312-RA | "-"

*********************** Rank 1358 [Score  6.939900] GBROWSE*******************

 CG4428 in-situ | CG4428 | + | -15321 | -12850 | DOWNSTREAM | CG4428-RA | "-"
insitu CG4629 in-situ | CG4629 | - | 6272 | -13144 | INTRAGENIC | intron:CG4629-RA:1 | intron:CG4629-RB:2 | CG4629-RA | "-" | CG4629-RB | "-"

*********************** Rank 1359 [Score  6.939600] GBROWSE*******************

 CG13801 in-situ | CG13801 | + | -3205 | -1133 | DOWNSTREAM | CG13801-RA | "-"
 CG1317 in-situ | CG1317 | - | 8189 | 4228 | DOWNSTREAM | CG1317-RB | "-"

*********************** Rank 1360 [Score  6.937700] GBROWSE*******************

 CG15159 in-situ | CG15159 | - | -3052 | -3921 | UPSTREAM | CG15159-RA | "-"
 CG10231 in-situ | CG10231 | - | 16005 | -14439 | INTRAGENIC | intron:CG10231-RA:1 | CG10231-RA | "-"

*********************** Rank 1361 [Score  6.935600] GBROWSE*******************

 CG9703 in-situ | Axs | - | -9735 | -13541 | UPSTREAM | CG9703-RA | "-"
insitu CG9699 in-situ | CG9699 | - | 1543 | -9419 | INTRAGENIC | intron:CG9699-RA:2 | intron:CG9699-RF:2 | intron:CG9699-RB:2 | intron:CG9699-RD:2 | intron:CG9699-RE:2 | intron:CG9699-RC:2 | CG9699-RA | "-" | CG9699-RF | "-" | CG9699-RB | "-" | CG9699-RD | "-" | CG9699-RE | "-" | CG9699-RC | "-"

*********************** Rank 1362 [Score  6.935200] GBROWSE*******************

insitu CG6393 in-situ | CG6393 | + | -3846 | 4960 | INTRAGENIC | intron:CG6393-RD:1 | intron:CG6393-RA:1 | intron:CG6393-RC:1 | CG6393-RD | "-" | CG6393-RA | "-" | CG6393-RC | "-" | CG6393-RB | "-"
 CG30327 in-situ | CG30327 | + | 2152 | 3375 | UPSTREAM | CG30327-RA | "-"

*********************** Rank 1363 [Score  6.934400] GBROWSE*******************

 CG4374 in-situ | CG4374 | - | -59164 | -63516 | UPSTREAM | CG4374-RA | "-"
 CG31225 in-situ | CG31225 | + | 10196 | 11770 | UPSTREAM | CG31225-RA | "-"

*********************** Rank 1364 [Score  6.932800] GBROWSE*******************

insitu highlight CG4922 in-situ | sala | + | -7090 | -6324 | DOWNSTREAM | CG4922-RA | "-"
 CG6488 in-situ | CG6488 | - | 5840 | 3515 | DOWNSTREAM | CG6488-RA | "-"

*********************** Rank 1365 [Score  6.928400] GBROWSE*******************

 CG13616 in-situ | CG13616 | + | -27348 | -26529 | DOWNSTREAM | CG13616-RA | "-"
 CG5610 in-situ | nAcRalpha-96Aa | - | 36658 | -19045 | INTRAGENIC | intron:CG5610-RA:3 | CG5610-RA | "-"

*********************** Rank 1366 [Score  6.928300] GBROWSE*******************

 CG15399 in-situ | CG15399 | - | -24396 | -25193 | UPSTREAM | CG15399-RA | "-"
insitu CG3139 in-situ | syt | - | 3839 | -15390 | INTRAGENIC | intron:CG3139-RA:2 | intron:CG3139-RB:2 | intron:CG3139-RC:1 | CG3139-RA | "-" | CG3139-RB | "-" | CG3139-RC | "-"

*********************** Rank 1367 [Score  6.925000] GBROWSE*******************

 CG13353 in-situ | CG13353 | + | -24010 | -22659 | DOWNSTREAM | CG13353-RA | "-"
 CG30483 in-situ | Prosap | - | 26569 | -52907 | INTRAGENIC | intron:CG30483-RA:1 | CG30483-RA | "-"

*********************** Rank 1368 [Score  6.924100] GBROWSE*******************

 CG8398 in-situ | CG8398 | + | -8346 | 514 | INTRAGENIC | intron:CG8398-RA:3 | intron:CG8398-RB:3 | intron:CG8398-RC:3 | CG8398-RA | "-" | CG8398-RB | "-" | CG8398-RC | "-"
insitu highlight CG8442 in-situ | Glu-RI | + | 6363 | 17395 | UPSTREAM | CG8442-RA | "-"

*********************** Rank 1369 [Score  6.923500] GBROWSE*******************

 CG4685 in-situ | CG4685 | - | -5392 | -7508 | UPSTREAM | CG4685-RA | "-"
insitu highlight CG17383 in-situ | CG17383 | + | 5514 | 17937 | UPSTREAM | CG17383-RA | "-" | CG17383-RB | "-" | CG17383-RD | "-" | CG17383-RC | "-"

*********************** Rank 1370 [Score  6.923500] GBROWSE*******************

 CG14943 in-situ | CG14943 | - | -30929 | -31453 | UPSTREAM | CG14943-RA | "-"
 CG14944 in-situ | CG14944 | - | 6283 | 5705 | DOWNSTREAM | CG14944-RA | "-"

*********************** Rank 1371 [Score  6.921500] GBROWSE*******************

insitu CG12701 in-situ | CG12701 | - | -29512 | -36453 | UPSTREAM | CG12701-RB | "-" | CG12701-RA | "-"
 CG12700 in-situ | skpD | + | 289 | 948 | UPSTREAM | CG12700-RA | "-"

*********************** Rank 1372 [Score  6.920800] GBROWSE*******************

 CG2212 in-situ | sws | - | -12024 | -23235 | UPSTREAM | CG2212-RA | "-" | CG2212-RB | "-"
 CG1543 in-situ | Tbh | + | 15643 | 46480 | UPSTREAM | CG1543-RA | "-" | CG1543-RB | "-"


*********************** Rank 1373 [Score  6.919900] GBROWSE*******************

 CG13024 in-situ | CG13024 | - | -23320 | -24569 | UPSTREAM | CG13024-RA | "-"
insitu CG9712 in-situ | TSG101 | + | 8079 | 10366 | UPSTREAM | CG9712-RA | "-"

*********************** Rank 1374 [Score  6.918200] GBROWSE*******************

 CG5718 in-situ | CG5718 | - | -895 | -3049 | UPSTREAM | CG5718-RA | "-"
 CG7260 in-situ | byn | + | 5729 | 10079 | UPSTREAM | CG7260-RA | "-"

*********************** Rank 1375 [Score  6.917400] GBROWSE*******************

 CG15109 in-situ | CG15109 | + | -5209 | -3560 | DOWNSTREAM | CG15109-RC | "-" | CG15109-RB | "-" | CG15109-RA | "-"
 CG16720 in-situ | 5-HT1A | + | 2529 | 57328 | UPSTREAM | CG16720-RB | "-" | CG16720-RA | "-"

*********************** Rank 1376 [Score  6.916300] GBROWSE*******************

 CG4096 in-situ | CG4096 | + | -838 | 8489 | INTRAGENIC | intron:CG4096-RA:1 | CG4096-RA | "-"
insitu CG33080 in-situ | CG33080 | - | 17753 | 8536 | DOWNSTREAM | CG33080-RA | "-" | CG33080-RB | "-"

*********************** Rank 1377 [Score  6.916300] GBROWSE*******************

 CG31741 in-situ | CG31741 | + | -1749 | -391 | DOWNSTREAM | CG31741-RA | "-"
 CG15153 in-situ | CG15153 | - | 6221 | 4924 | DOWNSTREAM | CG15153-RA | "-"

*********************** Rank 1378 [Score  6.915700] GBROWSE*******************

insitu CG3258 in-situ | ase | + | -6462 | -3776 | DOWNSTREAM | CG3258-RA | "-"
insitu CG3972 in-situ | Cyp4g1 | - | 2371 | 87 | DOWNSTREAM | CG3972-RA | "-"


*********************** Rank 1379 [Score  6.915500] GBROWSE*******************

insitu highlight CG31721 in-situ | CG31721 | + | -10960 | 71533 | INTRAGENIC | intron:CG31721-RA:2 | CG31721-RA | "-"
 CG6138 in-situ | CG6138 | + | 11449 | 19782 | UPSTREAM | CG6138-RB | "-" | CG6138-RA | "-"

*********************** Rank 1380 [Score  6.914600] GBROWSE*******************

 CG31749 in-situ | CG31749 | + | -7753 | -6963 | DOWNSTREAM | CG31749-RA | "-"
 CG10305 in-situ | RpS26 | - | 49170 | 48431 | DOWNSTREAM | CG10305-RA | "-" | CG10305-RB | "-" | CG10305-RC | "-"

*********************** Rank 1381 [Score  6.914600] GBROWSE*******************

 CG13935 in-situ | CG13935 | - | -1410 | -3935 | UPSTREAM | CG13935-RA | "-"
insitu CG1919 in-situ | CG1919 | - | 5509 | 3707 | DOWNSTREAM | CG1919-RA | "-"

*********************** Rank 1382 [Score  6.910100] GBROWSE*******************

 CG32632 in-situ | CG32632 | + | -511 | 12650 | INTRAGENIC | intron:CG32632-RB:1 | CG32632-RB | "-"
insitu CG7107 in-situ | up | - | 24562 | 15628 | DOWNSTREAM | CG7107-RA | "-" | CG7107-RB | "-" | CG7107-RD | "-"

*********************** Rank 1383 [Score  6.908800] GBROWSE*******************

 CG12408 in-situ | CG12408 | - | -63113 | -67543 | UPSTREAM | CG12408-RA | "-"
 CG17510 in-situ | CG17510 | - | 33849 | 32607 | DOWNSTREAM | CG17510-RA | "-" | CG17510-RB | "-"

*********************** Rank 1384 [Score  6.906900] GBROWSE*******************

 CG32726 in-situ | CG32726 | - | -21080 | -21599 | UPSTREAM | CG32726-RA | "-"
 CG11368 in-situ | CG11368 | + | 5385 | 5840 | UPSTREAM | CG11368-RA | "-"

*********************** Rank 1385 [Score  6.906600] GBROWSE*******************

 CG3289 in-situ | Ptpa | + | -2273 | -695 | DOWNSTREAM | CG3289-RA | "-"
 CG3254 in-situ | CG3254 | - | 28289 | 25179 | DOWNSTREAM | CG3254-RA | "-"

*********************** Rank 1386 [Score  6.905700] GBROWSE*******************

 CG10419 in-situ | CG10419 | - | -4737 | -5952 | UPSTREAM | CG10419-RA | "-"
 CG14082 in-situ | CG14082 | - | 5327 | 2204 | DOWNSTREAM | CG14082-RA | "-"

*********************** Rank 1387 [Score  6.905400] GBROWSE*******************

 CG9072 in-situ | CG9072 | - | -12053 | -12597 | UPSTREAM | CG9072-RA | "-"
 CG9081 in-situ | Cyp4s3 | - | 8453 | 5216 | DOWNSTREAM | CG9081-RA | "-"

*********************** Rank 1388 [Score  6.904300] GBROWSE*******************

 CG15381 in-situ | CG15381 | - | -12197 | -18641 | UPSTREAM | CG15381-RA | "-"
 CG4259 in-situ | CG4259 | - | 7178 | 6182 | DOWNSTREAM | CG4259-RA | "-"

*********************** Rank 1389 [Score  6.902000] GBROWSE*******************

insitu CG3401 in-situ | betaTub60D | + | -4350 | 2871 | INTRAGENIC | intron:CG3401-RA:1 | CG3401-RA | "-"
insitu CG4354 in-situ | slbo | - | 24074 | 21290 | DOWNSTREAM | CG4354-RA | "-"

*********************** Rank 1390 [Score  6.901700] GBROWSE*******************

 CG14521 in-situ | CG14521 | - | -3558 | -48917 | UPSTREAM | CG14521-RA | "-"
 CG14520 in-situ | CG14520 | + | 4006 | 4603 | UPSTREAM | CG14520-RA | "-"

*********************** Rank 1391 [Score  6.901600] GBROWSE*******************

 CG15747 in-situ | CG15747 | + | -7658 | -4972 | DOWNSTREAM | CG15747-RB | "-" | CG15747-RA | "-"
 CG10617 in-situ | CG10617 | + | 13259 | 22190 | UPSTREAM | CG10617-RA | "-"

*********************** Rank 1392 [Score  6.900500] GBROWSE*******************

 CG8129 in-situ | CG8129 | + | -11892 | -9546 | DOWNSTREAM | CG8129-RB | "-" | CG8129-RA | "-"
 CG8874 in-situ | Fps85D | - | 17231 | -9713 | INTRAGENIC | intron:CG8874-RB:3 | intron:CG8874-RA:3 | CG8874-RB | "-" | CG8874-RA | "-" | CG8874-RC | "-" | CG8874-RD | "-"

*********************** Rank 1393 [Score  6.900400] GBROWSE*******************

 CG5210 in-situ | Chit | + | -5477 | -3420 | DOWNSTREAM | CG5210-RA | "-"
 CG30463 in-situ | CG30463 | - | 34209 | -23714 | INTRAGENIC | intron:CG30463-RA:6 | intron:CG30463-RB:5 | CG30463-RA | "-" | CG30463-RB | "-"

*********************** Rank 1394 [Score  6.898300] GBROWSE*******************

 CG12063 in-situ | CG12063 | + | -1989 | 6136 | INTRAGENIC | intron:CG12063-RA:1 | CG12063-RA | "-"
insitu CG1499 in-situ | CG1499 | + | 42170 | 65305 | UPSTREAM | CG1499-RA | "-" | CG1499-RB | "-"

*********************** Rank 1395 [Score  6.897100] GBROWSE*******************

 CG6149 in-situ | CG6149 | - | -1448 | -7061 | UPSTREAM | CG6149-RA | "-"
 CG7557 in-situ | CG7557 | + | 3343 | 4753 | UPSTREAM | CG7557-RA | "-" | CG7557-RB | "-"

*********************** Rank 1396 [Score  6.894700] GBROWSE*******************

 CG5907 in-situ | Frq | + | -1734 | 13722 | INTRAGENIC | intron:CG5907-RA:1 | CG5907-RA | "-"
 CG5927 in-situ | Her | - | 17229 | 16652 | DOWNSTREAM | CG5927-RA | "-"

*********************** Rank 1397 [Score  6.894100] GBROWSE*******************

 CG30122 in-situ | CG30122 | - | -8162 | -15101 | UPSTREAM | CG30122-RB | "-"
 CG5473 in-situ | SP2637 | - | 10770 | -6407 | INTRAGENIC | intron:CG5473-RA:1 | intron:CG5473-RB:1 | CG5473-RA | "-" | CG5473-RB | "-"

*********************** Rank 1398 [Score  6.890900] GBROWSE*******************

 CG4701 in-situ | BG:DS06874.3 | + | -6458 | -5142 | DOWNSTREAM | CG4701-RA | "-"
 CG4650 in-situ | CG4650 | - | 2577 | 1448 | DOWNSTREAM | CG4650-RA | "-"

*********************** Rank 1399 [Score  6.889300] GBROWSE*******************

 CG9850 in-situ | CG9850 | - | -3606 | -18968 | UPSTREAM | CG9850-RA | "-"
 CG15800 in-situ | CG15800 | - | 18510 | 18036 | DOWNSTREAM | CG15800-RA | "-"

*********************** Rank 1400 [Score  6.889200] GBROWSE*******************

 CG7329 in-situ | CG7329 | - | -2088 | -3789 | UPSTREAM | CG7329-RA | "-"
 CG31872 in-situ | CG31872 | + | 6805 | 10351 | UPSTREAM | CG31872-RA | "-"

*********************** Rank 1401 [Score  6.888600] GBROWSE*******************

 CG12283 in-situ | kek1 | - | -56243 | -60100 | UPSTREAM | CG12283-RA | "-"
 CG5983 in-situ | ACXC | + | 33500 | 37744 | UPSTREAM | CG5983-RA | "-"


*********************** Rank 1402 [Score  6.888300] GBROWSE*******************

insitu highlight CG1849 in-situ | run | + | -29694 | -26809 | DOWNSTREAM | CG1849-RA | "-"
 CG1324 in-situ | CG1324 | - | 11825 | 10609 | DOWNSTREAM | CG1324-RA | "-"

*********************** Rank 1403 [Score  6.887800] GBROWSE*******************

 CG16837 in-situ | CG16837 | + | -756 | -161 | DOWNSTREAM | CG16837-RA | "-"
 CG13579 in-situ | CG13579 | + | 6865 | 13463 | UPSTREAM | CG13579-RA | "-"

*********************** Rank 1404 [Score  6.887800] GBROWSE*******************

insitu CG11584 in-situ | CG11584 | + | -3161 | -870 | DOWNSTREAM | CG11584-RB | "-"
 CG32603 in-situ | CG32603 | + | 350 | 1545 | UPSTREAM | CG32603-RA | "-"

*********************** Rank 1405 [Score  6.886200] GBROWSE*******************

 CG9887 in-situ | CG9887 | + | -2677 | 4715 | INTRAGENIC | intron:CG9887-RA:3 | CG9887-RA | "-"
 CG18641 in-situ | CG18641 | - | 9146 | 7901 | DOWNSTREAM | CG18641-RA | "-"

*********************** Rank 1406 [Score  6.884200] GBROWSE*******************

 CG13951 in-situ | l(2)k10201 | + | -6179 | -5514 | DOWNSTREAM | CG13951-RA | "-"
 CG8804 in-situ | wun | - | 8192 | -4721 | INTRAGENIC | intron:CG8804-RB:2 | intron:CG8804-RA:1 | CG8804-RB | "-" | CG8804-RA | "-"


*********************** Rank 1407 [Score  6.883200] GBROWSE*******************

 CG15275 in-situ | BG:DS01219.3 | - | -44220 | -44943 | UPSTREAM | CG15275-RA | "-"
 CG4482 in-situ | BG:DS01219.1 | - | 13225 | -5063 | INTRAGENIC | intron:CG4482-RA:4 | intron:CG4482-RB:3 | CG4482-RA | "-" | CG4482-RB | "-"

*********************** Rank 1408 [Score  6.883100] GBROWSE*******************

 CG31330 in-situ | CG31330 | - | -43380 | -49594 | UPSTREAM | CG31330-RA | "-"
 CG8464 in-situ | CG8464 | - | 35132 | 33334 | DOWNSTREAM | CG8464-RA | "-"

*********************** Rank 1409 [Score  6.883000] GBROWSE*******************

 CG4783 in-situ | CG4783 | + | -23141 | -22702 | DOWNSTREAM | CG4783-RA | "-"
 CG31213 in-situ | CG31213 | + | 1902 | 7401 | UPSTREAM | CG31213-RA | "-"

*********************** Rank 1410 [Score  6.882300] GBROWSE*******************

 CG12467 in-situ | CG12467 | - | -2472 | -15232 | UPSTREAM | CG12467-RA | "-"
 CG14635 in-situ | CG14635 | + | 20074 | 20433 | UPSTREAM | CG14635-RA | "-"

*********************** Rank 1411 [Score  6.880700] GBROWSE*******************

insitu CG11387 in-situ | ct | + | -30475 | 36400 | INTRAGENIC | intron:CG11387-RA:1 | intron:CG11387-RB:2 | CG11387-RA | "-" | CG11387-RB | "-"
 CG12690 in-situ | CHES-1-like | - | 57071 | 45264 | DOWNSTREAM | CG12690-RA | "-"

*********************** Rank 1412 [Score  6.880700] GBROWSE*******************

 CG12165 in-situ | Incenp | + | -3232 | -138 | DOWNSTREAM | CG12165-RA | "-"
 CG11101 in-situ | pwn | + | 13609 | 18703 | UPSTREAM | CG11101-RA | "-"

*********************** Rank 1413 [Score  6.880100] GBROWSE*******************

insitu CG17110 in-situ | CG17110 | + | -324 | 1232 | INTRAGENIC | intron:CG17110-RA:1 | CG17110-RA | "-"
insitu CG17109 in-situ | CG17109 | + | 1871 | 3358 | UPSTREAM | CG17109-RA | "-"

*********************** Rank 1414 [Score  6.879100] GBROWSE*******************

 CG14597 in-situ | CG14597 | - | -29318 | -29959 | UPSTREAM | CG14597-RA | "-"
 CG31146 in-situ | CG31146 | + | 25190 | 61259 | UPSTREAM | CG31146-RD | "-"

*********************** Rank 1415 [Score  6.878300] GBROWSE*******************

 CG7722 in-situ | CG7722 | - | -5955 | -7436 | UPSTREAM | CG7722-RA | "-"
 CG17326 in-situ | CG17326 | - | 43959 | 41784 | DOWNSTREAM | CG17326-RA | "-"

*********************** Rank 1416 [Score  6.877000] GBROWSE*******************

 CG32198 in-situ | CG32198 | + | -45630 | -45220 | DOWNSTREAM | CG32198-RB | "-"
 CG7285 in-situ | Drostar1 | + | 1267 | 2790 | UPSTREAM | CG7285-RA | "-"

*********************** Rank 1417 [Score  6.876600] GBROWSE*******************

 CG12998 in-situ | CG12998 | + | -959 | -347 | DOWNSTREAM | CG12998-RA | "-"
 CG5172 in-situ | CG5172 | + | 1601 | 2689 | UPSTREAM | CG5172-RA | "-"

*********************** Rank 1418 [Score  6.876500] GBROWSE*******************

 CG15498 in-situ | CG15498 | - | -2065 | -3695 | UPSTREAM | CG15498-RA | "-"
 CG18402 in-situ | InR | - | 9315 | 1285 | DOWNSTREAM | CG18402-RA | "-"

*********************** Rank 1419 [Score  6.876300] GBROWSE*******************

 CG13110 in-situ | CG13110 | + | -4438 | -3915 | DOWNSTREAM | CG13110-RA | "-"
 CG13111 in-situ | CG13111 | - | 18676 | 18110 | DOWNSTREAM | CG13111-RA | "-"

*********************** Rank 1420 [Score  6.874600] GBROWSE*******************

insitu highlight CG32306 in-situ | CG32306 | + | -3118 | 32428 | INTRAGENIC | intron:CG32306-RB:1 | CG32306-RB | "-" | CG32306-RA | "-" | CG32306-RC | "-"
 CG16762 in-situ | CG16762 | - | 43173 | 42346 | DOWNSTREAM | CG16762-RA | "-"

*********************** Rank 1421 [Score  6.874000] GBROWSE*******************

 CG6640 in-situ | CG6640 | - | -38640 | -43774 | UPSTREAM | CG6640-RA | "-" | CG6640-RB | "-"
 CG8072 in-situ | CG8072 | + | 12443 | 13235 | UPSTREAM | CG8072-RA | "-"

*********************** Rank 1422 [Score  6.873500] GBROWSE*******************

 CG31774 in-situ | fred | - | -121 | -41768 | UPSTREAM | CG31774-RA | "-"
 CG15422 in-situ | CG15422 | + | 57775 | 58089 | UPSTREAM | CG15422-RA | "-"

*********************** Rank 1423 [Score  6.872100] GBROWSE*******************

 CG32606 in-situ | CG32606 | - | -6887 | -7300 | UPSTREAM | CG32606-RA | "-"
 CG11068 in-situ | CG11068 | + | 33260 | 34600 | UPSTREAM | CG11068-RA | "-"

*********************** Rank 1424 [Score  6.869800] GBROWSE*******************

 CG12408 in-situ | CG12408 | - | -66063 | -70493 | UPSTREAM | CG12408-RA | "-"
 CG17510 in-situ | CG17510 | - | 30899 | 29657 | DOWNSTREAM | CG17510-RA | "-" | CG17510-RB | "-"

*********************** Rank 1425 [Score  6.868800] GBROWSE*******************

insitu highlight CG4702 in-situ | CG4702 | - | -15812 | -22432 | UPSTREAM | CG4702-RA | "-"
 CG10095 in-situ | CG10095 | - | 14586 | -5176 | INTRAGENIC | intron:CG10095-RA:5 | CG10095-RA | "-"

*********************** Rank 1426 [Score  6.867900] GBROWSE*******************

insitu highlight CG9015 in-situ | en | - | -18327 | -22533 | UPSTREAM | CG9015-RB | "-" | CG9015-RA | "-"
 CG10897 in-situ | tou | - | 69294 | 32345 | DOWNSTREAM | CG10897-RA | "-" | CG10897-RC | "-" | CG10897-RD | "-" | CG10897-RB | "-"

*********************** Rank 1427 [Score  6.867000] GBROWSE*******************

 CG1867 in-situ | Or98b | + | -8170 | -6792 | DOWNSTREAM | CG1867-RA | "-"
 CG14064 in-situ | beat-VI | + | 57792 | 112797 | UPSTREAM | CG14064-RA | "-"

*********************** Rank 1428 [Score  6.866700] GBROWSE*******************

insitu highlight CG2851 in-situ | Gsc | - | -398 | -11545 | UPSTREAM | CG2851-RA | "-"
 CG13689 in-situ | CG13689 | + | 8027 | 8254 | UPSTREAM | CG13689-RA | "-"

*********************** Rank 1429 [Score  6.866600] GBROWSE*******************

 CG10793 in-situ | CG10793 | - | -41910 | -43568 | UPSTREAM | CG10793-RA | "-"
insitu highlight CG10798 in-situ | dm | + | 14871 | 27704 | UPSTREAM | CG10798-RA | "-"

*********************** Rank 1430 [Score  6.864600] GBROWSE*******************

 CG31247 in-situ | tinc | + | -36439 | -18042 | DOWNSTREAM | CG31247-RB | "-" | CG31247-RA | "-" | CG31247-RC | "-" | CG31247-RD | "-"
 CG7321 in-situ | Rim | - | 6782 | -13787 | INTRAGENIC | intron:CG7321-RA:8 | CG7321-RA | "-"

*********************** Rank 1431 [Score  6.864400] GBROWSE*******************

 CG18285 in-situ | igl | + | -43636 | -8781 | DOWNSTREAM | CG18285-RA | "-" | CG18285-RB | "-"
 CG8090 in-situ | CG8090 | - | 2102 | 232 | DOWNSTREAM | CG8090-RA | "-"

*********************** Rank 1432 [Score  6.863500] GBROWSE*******************

 CG1907 in-situ | CG1907 | + | -8778 | -7054 | DOWNSTREAM | CG1907-RA | "-"
 CG18741 in-situ | DopR2 | - | 32172 | 2489 | DOWNSTREAM | CG18741-RA | "-" | CG18741-RB | "-"

*********************** Rank 1433 [Score  6.862900] GBROWSE*******************

 CG30384 in-situ | CG30384 | + | -6951 | -4645 | DOWNSTREAM | CG30384-RA | "-"
 CG1854 in-situ | Or43a | - | 44965 | 42891 | DOWNSTREAM | CG1854-RA | "-"


*********************** Rank 1434 [Score  6.862200] GBROWSE*******************

 CG13109 in-situ | tai | + | -24017 | 55571 | INTRAGENIC | intron:CG13109-RA:1 | CG13109-RA | "-"
 CG17009 in-situ | CG17009 | - | 60193 | 59477 | DOWNSTREAM | CG17009-RA | "-"

*********************** Rank 1435 [Score  6.862200] GBROWSE*******************

 CG9871 in-situ | CG9871 | - | -2395 | -3640 | UPSTREAM | CG9871-RA | "-"
 CG12782 in-situ | CG12782 | - | 15302 | 14037 | DOWNSTREAM | CG12782-RA | "-"


*********************** Rank 1436 [Score  6.862000] GBROWSE*******************

 CG9580 in-situ | Sdic | - | -5392 | -9784 | UPSTREAM | CG9580-RA | "-" | CG9580-RB | "-"
 CG32823 in-situ | CG32823 | - | 11164 | 6112 | DOWNSTREAM | CG32823-RB | "-"

*********************** Rank 1437 [Score  6.858400] GBROWSE*******************

 CG1976 in-situ | RhoGAP100F | + | -117385 | -105003 | DOWNSTREAM | CG1976-RA | "-"
insitu CG2003 in-situ | CG2003 | + | 27868 | 40654 | UPSTREAM | CG2003-RA | "-" | CG2003-RB | "-"


*********************** Rank 1438 [Score  6.858100] GBROWSE*******************

 CG7968 in-situ | BG:DS00941.15 | + | -48986 | -48071 | DOWNSTREAM | CG7968-RA | "-"
insitu CG8954 in-situ | CG8954 | - | 6261 | 955 | DOWNSTREAM | CG8954-RA | "-" | CG8954-RB | "-"

*********************** Rank 1439 [Score  6.857400] GBROWSE*******************

 CG11634 in-situ | CG11634 | + | -49563 | -48476 | DOWNSTREAM | CG11634-RA | "-"
 CG2528 in-situ | CG2528 | - | 27009 | 24907 | DOWNSTREAM | CG2528-RA | "-"

*********************** Rank 1440 [Score  6.856500] GBROWSE*******************

 CG31009 in-situ | Cad99C | + | -3058 | 6041 | INTRAGENIC | intron:CG31009-RA:3 | CG31009-RA | "-"
 CG18041 in-situ | CG18041 | - | 8807 | 6028 | DOWNSTREAM | CG18041-RA | "-"

*********************** Rank 1441 [Score  6.855300] GBROWSE*******************

 CG14271 in-situ | Gas8 | - | -6912 | -20754 | UPSTREAM | CG14271-RB | "-"
 CG13021 in-situ | CG13021 | + | 2553 | 4297 | UPSTREAM | CG13021-RA | "-" | CG13021-RB | "-" | CG13021-RC | "-"

*********************** Rank 1442 [Score  6.854900] GBROWSE*******************

 CG13871 in-situ | CG13871 | + | -2130 | -568 | DOWNSTREAM | CG13871-RA | "-"
 CG13867 in-situ | Arc32 | - | 7268 | 6303 | DOWNSTREAM | CG13867-RA | "-"

*********************** Rank 1443 [Score  6.854800] GBROWSE*******************

 CG3411 in-situ | bs | + | -23619 | 9143 | INTRAGENIC | intron:CG3411-RA:2 | CG3411-RA | "-"
 CG13578 in-situ | CG13578 | + | 10804 | 11287 | UPSTREAM | CG13578-RA | "-"

*********************** Rank 1444 [Score  6.854000] GBROWSE*******************

insitu highlight CG4717 in-situ | kni | - | -5449 | -8482 | UPSTREAM | CG4717-RA | "-"
insitu CG13253 in-situ | CG13253 | - | 18386 | 15983 | DOWNSTREAM | CG13253-RA | "-"

*********************** Rank 1445 [Score  6.853800] GBROWSE*******************

 CG15204 in-situ | CG15204 | - | -17760 | -18417 | UPSTREAM | CG15204-RA | "-"
 CG12626 in-situ | CG12626 | - | 50554 | 50170 | DOWNSTREAM | CG12626-RA | "-"

*********************** Rank 1446 [Score  6.853600] GBROWSE*******************

 CG1973 in-situ | CG1973 | + | -9636 | -5255 | DOWNSTREAM | CG1973-RA | "-"
 CG15507 in-situ | CG15507 | + | 8123 | 8755 | UPSTREAM | CG15507-RA | "-"

*********************** Rank 1447 [Score  6.852200] GBROWSE*******************

insitu highlight CG10619 in-situ | tup | - | -13461 | -35217 | UPSTREAM | CG10619-RA | "-" | CG10619-RB | "-"
 CG18397 in-situ | CG18397 | - | 49231 | 21808 | DOWNSTREAM | CG18397-RA | "-"

*********************** Rank 1448 [Score  6.851400] GBROWSE*******************

 CG10362 in-situ | CG10362 | - | -44342 | -48782 | UPSTREAM | CG10362-RA | "-"
insitu CG1522 in-situ | cac | - | 8691 | -41506 | INTRAGENIC | intron:CG1522-RA:9 | CG1522-RA | "-"

*********************** Rank 1449 [Score  6.851300] GBROWSE*******************

insitu CG6106 in-situ | CG6106 | + | -9008 | -5929 | DOWNSTREAM | CG6106-RA | "-"
 CG6103 in-situ | CrebB-17A | + | 4242 | 8160 | UPSTREAM | CG6103-RA | "-" | CG6103-RB | "-"

*********************** Rank 1450 [Score  6.851300] GBROWSE*******************

 CG8854 in-situ | CG8854 | - | -2141 | -4528 | UPSTREAM | CG8854-RA | "-"
 CG13170 in-situ | CG13170 | + | 11422 | 11775 | UPSTREAM | CG13170-RA | "-"

*********************** Rank 1451 [Score  6.850500] GBROWSE*******************

 CG17580 in-situ | CG17580 | + | -32859 | -31725 | DOWNSTREAM | CG17580-RA | "-"
 CG17577 in-situ | Cyp9h1 | - | 2985 | 1300 | DOWNSTREAM | CG17577-RA | "-"

*********************** Rank 1452 [Score  6.849300] GBROWSE*******************

insitu CG32499 in-situ | CG32499 | - | -141446 | -190513 | UPSTREAM | CG32499-RA | "-"
 CG12446 in-situ | CG12446 | - | 37780 | 35132 | DOWNSTREAM | CG12446-RA | "-"

*********************** Rank 1453 [Score  6.848100] GBROWSE*******************

 CG1690 in-situ | CG1690 | - | -2749 | -3894 | UPSTREAM | CG1690-RA | "-"
insitu CG1698 in-situ | CG1698 | + | 32526 | 37190 | UPSTREAM | CG1698-RA | "-"

*********************** Rank 1454 [Score  6.847800] GBROWSE*******************

 CG11958 in-situ | Cnx99A | - | -72168 | -76561 | UPSTREAM | CG11958-RA | "-" | CG11958-RB | "-"
 CG11516 in-situ | CG11516 | + | 34890 | 36943 | UPSTREAM | CG11516-RA | "-"

*********************** Rank 1455 [Score  6.846400] GBROWSE*******************

 CG15321 in-situ | CG15321 | - | -7394 | -8101 | UPSTREAM | CG15321-RA | "-"
insitu highlight CG12653 in-situ | btd | + | 3122 | 6507 | UPSTREAM | CG12653-RA | "-"

note: overlaps known module btd_head by 500 bases (module coords: 9429057-9430856)

*********************** Rank 1456 [Score  6.844700] GBROWSE*******************

 CG2750 in-situ | CG2750 | + | -55090 | -49036 | DOWNSTREAM | CG2750-RA | "-"
 CG1924 in-situ | CG1924 | - | 16317 | 14605 | DOWNSTREAM | CG1924-RA | "-"

*********************** Rank 1457 [Score  6.844700] GBROWSE*******************

insitu highlight CG1056 in-situ | 5-HT2 | + | -27104 | -6670 | DOWNSTREAM | CG1056-RA | "-" | CG1056-RB | "-"
 CG1057 in-situ | Trap18 | + | 1995 | 3318 | UPSTREAM | CG1057-RA | "-" | CG1057-RB | "-"

*********************** Rank 1458 [Score  6.844400] GBROWSE*******************

 CG32970 in-situ | CG32970 | + | -17991 | -15150 | DOWNSTREAM | CG32970-RA | "-"
 CG15287 in-situ | BG:DS01068.1 | + | 2281 | 7719 | UPSTREAM | CG15287-RA | "-"

*********************** Rank 1459 [Score  6.843800] GBROWSE*******************

 CG4090 in-situ | CG4090 | - | -41515 | -47943 | UPSTREAM | CG4090-RA | "-"
 CG31262 in-situ | CG31262 | - | 17969 | 15876 | DOWNSTREAM | CG31262-RA | "-"


*********************** Rank 1460 [Score  6.842500] GBROWSE*******************

insitu highlight CG4889 in-situ | wg | + | -29258 | -20164 | DOWNSTREAM | CG4889-RA | "-" | CG4889-RB | "-"
 CG4969 in-situ | Wnt6 | + | 14479 | 15861 | UPSTREAM | CG4969-RA | "-"

*********************** Rank 1461 [Score  6.842000] GBROWSE*******************

 CG12820 in-situ | CG12820 | - | -8 | -1354 | UPSTREAM | CG12820-RA | "-"
 CG30377 in-situ | CG30377 | + | 46204 | 60261 | UPSTREAM | CG30377-RA | "-"


*********************** Rank 1462 [Score  6.841900] GBROWSE*******************

 CG2904 in-situ | EG:EG0002.3 | + | -1101 | 5201 | INTRAGENIC | intron:CG2904-RA:2 | intron:CG2904-RB:2 | CG2904-RA | "-" | CG2904-RB | "-"
 CG2913 in-situ | yin | - | 16942 | 11579 | DOWNSTREAM | CG2913-RA | "-" | CG2913-RB | "-" | CG2913-RC | "-"

*********************** Rank 1463 [Score  6.841600] GBROWSE*******************

 CG12690 in-situ | CHES-1-like | - | -20579 | -32386 | UPSTREAM | CG12690-RA | "-"
 CG15478 in-situ | CG15478 | - | 655 | -4488 | INTRAGENIC | intron:CG15478-RA:1 | CG15478-RA | "-"

*********************** Rank 1464 [Score  6.841300] GBROWSE*******************

 CG8398 in-situ | CG8398 | + | -2196 | 6664 | INTRAGENIC | intron:CG8398-RA:1 | intron:CG8398-RB:1 | CG8398-RA | "-" | CG8398-RB | "-" | CG8398-RC | "-"
insitu highlight CG8442 in-situ | Glu-RI | + | 12513 | 23545 | UPSTREAM | CG8442-RA | "-"

*********************** Rank 1465 [Score  6.841100] GBROWSE*******************

 CG11634 in-situ | CG11634 | + | -18013 | -16926 | DOWNSTREAM | CG11634-RA | "-"
 CG2528 in-situ | CG2528 | - | 58559 | 56457 | DOWNSTREAM | CG2528-RA | "-"

*********************** Rank 1466 [Score  6.840800] GBROWSE*******************

 CG12758 in-situ | sano | + | -2636 | 77880 | INTRAGENIC | intron:CG12758-RB:2 | intron:CG12758-RA:1 | CG12758-RB | "-" | CG12758-RA | "-" | CG12758-RC | "-" | CG12758-RD | "-"
 CG30329 in-situ | CG30329 | - | 30684 | 27970 | DOWNSTREAM | CG30329-RA | "-"

*********************** Rank 1467 [Score  6.840700] GBROWSE*******************

 CG10501 in-situ | amd | + | -2558 | 1393 | INTRAGENIC | intron:CG10501-RB:2 | intron:CG10501-RA:1 | CG10501-RB | "-" | CG10501-RA | "-"
insitu CG10561 in-situ | CG10561 | + | 1684 | 3854 | UPSTREAM | CG10561-RA | "-"

*********************** Rank 1468 [Score  6.839600] GBROWSE*******************

 CG13116 in-situ | CG13116 | + | -4631 | -3622 | DOWNSTREAM | CG13116-RA | "-"
 CG13117 in-situ | CG13117 | + | 2074 | 2762 | UPSTREAM | CG13117-RA | "-"

*********************** Rank 1469 [Score  6.839500] GBROWSE*******************

 CG15661 in-situ | CG15661 | - | -14739 | -16380 | UPSTREAM | CG15661-RA | "-"
insitu CG18375 in-situ | CG18375 | - | 19054 | -14043 | INTRAGENIC | intron:CG18375-RA:2 | CG18375-RA | "-" | CG18375-RB | "-"

*********************** Rank 1470 [Score  6.839100] GBROWSE*******************

 CG31817 in-situ | BG:DS02740.18 | - | -109 | -7293 | UPSTREAM | CG31817-RA | "-"
 CG31818 in-situ | CG31818 | - | 925 | 160 | DOWNSTREAM | CG31818-RA | "-"

*********************** Rank 1471 [Score  6.838600] GBROWSE*******************

 CG6371 in-situ | hug | + | -25071 | -22183 | DOWNSTREAM | CG6371-RA | "-"
 CG6989 in-situ | CG6989 | - | 3627 | 1385 | DOWNSTREAM | CG6989-RA | "-"

*********************** Rank 1472 [Score  6.838300] GBROWSE*******************

insitu CG9986 in-situ | CG9986 | + | -23875 | -21638 | DOWNSTREAM | CG9986-RA | "-"
 CG10011 in-situ | CG10011 | - | 5051 | -22081 | INTRAGENIC | intron:CG10011-RA:1 | CG10011-RA | "-"

*********************** Rank 1473 [Score  6.837200] GBROWSE*******************

 CG5069 in-situ | croc | - | -4045 | -5856 | UPSTREAM | CG5069-RA | "-"
 CG7204 in-situ | CG7204 | - | 9479 | 8331 | DOWNSTREAM | CG7204-RA | "-"


*********************** Rank 1474 [Score  6.836900] GBROWSE*******************

 CG12898 in-situ | CG12898 | - | -22 | -492 | UPSTREAM | CG12898-RA | "-"
 CG12897 in-situ | CG12897 | - | 5425 | 4765 | DOWNSTREAM | CG12897-RA | "-"

*********************** Rank 1475 [Score  6.835400] GBROWSE*******************

 CG6758 in-situ | CG6758 | + | -3093 | -571 | DOWNSTREAM | CG6758-RA | "-"
 CG30279 in-situ | CG30279 | + | 1182 | 1529 | UPSTREAM | CG30279-RA | "-"

*********************** Rank 1476 [Score  6.835100] GBROWSE*******************

insitu CG15251 in-situ | CG15251 | - | -1684 | -5120 | UPSTREAM | CG15251-RA | "-"
insitu CG15252 in-situ | CG15252 | - | 8185 | 5763 | DOWNSTREAM | CG15252-RA | "-"

*********************** Rank 1477 [Score  6.834500] GBROWSE*******************

 CG31690 in-situ | CG31690 | + | -2158 | 42454 | INTRAGENIC | intron:CG31690-RB:3 | CG31690-RB | "-"
 CG15398 in-situ | CG15398 | - | 19337 | 18395 | DOWNSTREAM | CG15398-RA | "-"

*********************** Rank 1478 [Score  6.833100] GBROWSE*******************

 CG7423 in-situ | CG7423 | - | -1762 | -2136 | UPSTREAM | CG7423-RA | "-"
 CG15882 in-situ | CG15882 | - | 39336 | 38860 | DOWNSTREAM | CG15882-RA | "-"

*********************** Rank 1479 [Score  6.831500] GBROWSE*******************

 CG12676 in-situ | ed | + | -65252 | 18428 | INTRAGENIC | intron:CG12676-RA:5 | CG12676-RA | "-"
 CG31962 in-situ | Sr-CIII | + | 23732 | 24811 | UPSTREAM | CG31962-RA | "-"

*********************** Rank 1480 [Score  6.831400] GBROWSE*******************

 CG4438 in-situ | CG4438 | - | -11201 | -11788 | UPSTREAM | CG4438-RA | "-"
 CG3759 in-situ | CG3759 | + | 7628 | 17379 | UPSTREAM | CG3759-RA | "-"

*********************** Rank 1481 [Score  6.829700] GBROWSE*******************

 CG5079 in-situ | CG5079 | + | -2758 | -2245 | DOWNSTREAM | CG5079-RA | "-"
 CG5071 in-situ | CG5071 | + | 1851 | 4101 | UPSTREAM | CG5071-RB | "-" | CG5071-RA | "-"

*********************** Rank 1482 [Score  6.828800] GBROWSE*******************

 CG32973 in-situ | CG32973 | - | -3109 | -4093 | UPSTREAM | CG32973-RA | "-"
 CG16879 in-situ | BG:DS01759.1 | + | 656 | 2686 | UPSTREAM | CG16879-RA | "-"

*********************** Rank 1483 [Score  6.828100] GBROWSE*******************

 CG4054 in-situ | CG4054 | + | -2529 | -1388 | DOWNSTREAM | CG4054-RA | "-"
 CG13487 in-situ | CG13487 | + | 10897 | 12427 | UPSTREAM | CG13487-RA | "-"

*********************** Rank 1484 [Score  6.828000] GBROWSE*******************

 CG31257 in-situ | CG31257 | + | -20798 | -18751 | DOWNSTREAM | CG31257-RA | "-"
 CG31418 in-situ | CG31418 | + | 8932 | 9652 | UPSTREAM | CG31418-RA | "-"

*********************** Rank 1485 [Score  6.827400] GBROWSE*******************

 CG17453 in-situ | Cyp317a1 | + | -10229 | -8673 | DOWNSTREAM | CG17453-RA | "-"
 CG10249 in-situ | BcDNA:GH03482 | - | 2493 | -8249 | INTRAGENIC | intron:CG10249-RC:3 | intron:CG10249-RA:1 | CG10249-RC | "-" | CG10249-RA | "-" | CG10249-RB | "-"

*********************** Rank 1486 [Score  6.826400] GBROWSE*******************

 CG17090 in-situ | CG17090 | + | -14401 | 18594 | INTRAGENIC | intron:CG17090-RB:2 | intron:CG17090-RA:2 | CG17090-RB | "-" | CG17090-RA | "-"
 CG12169 in-situ | CG12169 | + | 23866 | 25339 | UPSTREAM | CG12169-RA | "-"

*********************** Rank 1487 [Score  6.826300] GBROWSE*******************

insitu CG32954 in-situ | CG32954 | + | -4143 | -796 | DOWNSTREAM | CG32954-RA | "-" | CG32954-RB | "-" | CG32954-RC | "-" | CG32954-RG | "-" | CG32954-RH | "-" | CG32954-RF | "-" | CG32954-RD | "-" | CG32954-RE | "-"
insitu CG3479 in-situ | osp | - | 69616 | -19922 | INTRAGENIC | intron:CG3479-RA:2 | CG3479-RA | "-"

*********************** Rank 1488 [Score  6.825900] GBROWSE*******************

 CG8532 in-situ | lqf | + | -8017 | 1458 | INTRAGENIC | intron:CG8532-RB:8 | intron:CG8532-RA:9 | intron:CG8532-RD:7 | intron:CG8532-RC:5 | CG8532-RB | "-" | CG8532-RA | "-" | CG8532-RD | "-" | CG8532-RC | "-"
 CG16998 in-situ | CG16998 | - | 3015 | 2239 | DOWNSTREAM | CG16998-RA | "-"

*********************** Rank 1489 [Score  6.825700] GBROWSE*******************

insitu CG31163 in-situ | CG31163 | + | -24156 | 140057 | INTRAGENIC | intron:CG31163-RB:3 | intron:CG31163-RC:2 | CG31163-RB | "-" | CG31163-RC | "-" | CG31163-RA | "-"
 CG5732 in-situ | CG5732 | + | 53810 | 58682 | UPSTREAM | CG5732-RA | "-"

*********************** Rank 1490 [Score  6.825500] GBROWSE*******************

insitu CG7998 in-situ | CG7998 | + | -4271 | -1956 | DOWNSTREAM | CG7998-RA | "-"
insitu CG31240 in-situ | repo | + | 5532 | 8942 | UPSTREAM | CG31240-RA | "-"

*********************** Rank 1491 [Score  6.825400] GBROWSE*******************
[truncated: 388,994 more chars]
